# Stereodivergent entry to β-branched β-trifluoromethyl α-amino acid derivatives by sequential catalytic asymmetric reactions†

**DOI:** 10.1039/d1sc01442k

**Published:** 2021-06-29

**Authors:** Vasco Corti, Riccardo Riccioli, Ada Martinelli, Sofia Sandri, Mariafrancesca Fochi, Luca Bernardi

**Affiliations:** Department of Industrial Chemistry “Toso Montanari” and INSTM RU Bologna, Alma Mater Studiorum – University of Bologna V. Risorgimento 4 40136 Bologna Italy mariafrancesca.fochi@unibo.it luca.bernardi2@unibo.it

## Abstract

Currently, conventional reductive catalytic methodologies do not guarantee general access to enantioenriched β-branched β-trifluoromethyl α-amino acid derivatives. Herein, a one-pot approach to these important α-amino acids, grounded on the reduction – ring opening of Erlenmeyer–Plöchl azlactones, is presented. The configurations of the two chirality centers of the products are established during each of the two catalytic steps, enabling a stereodivergent process.

## Abstract 2

A one-pot approach to β-branched β-trifluoromethyl α-amino acids, grounded on the reduction – ring opening of Erlenmeyer–Plöchl azlactones, and complementary to conventional catalytic asymmetric hydrogenation, is presented.

## Graphical abstract

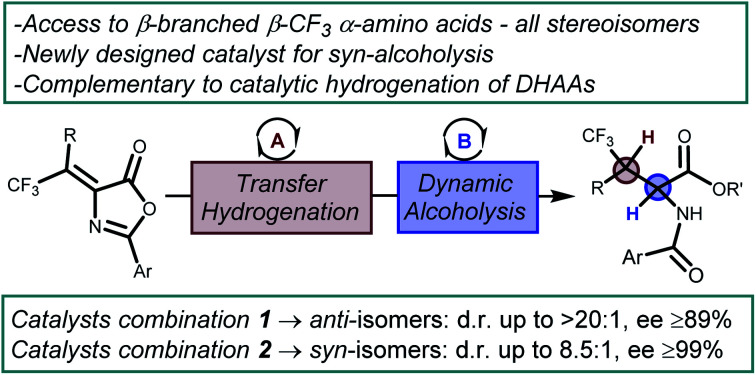

## Introduction

**Scheme 1:**
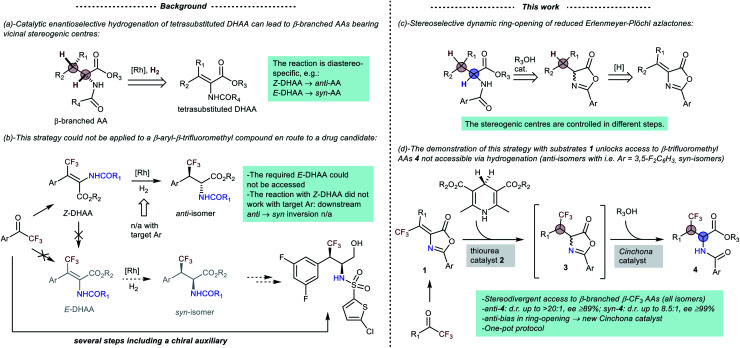
(Left) (a) catalytic asymmetric hydrogenation approach to β-branched AAs, and (b) its failure with a β-trifluoromethyl drug candidate. (Right) (c) the underlying concept of this work and (d) its demonstration with β-trifluoromethyl substrates **1**.

β-Branched α-amino acids (AAs) carrying different β-substituents – thus bearing two vicinal chirality centres, at the α- and β-carbons – are important yet challenging synthetic targets.^1^ The stereocontrolled preparation of these compounds has been tackled and realised with different (catalytic) methods.^2–7^ A non-comprehensive list of examples includes enantioselective conjugate additions and alkylations of glycinate imines,^2^ palladium catalysed β-C(sp^3^)–H alkylation and arylation of AAs,^3^ aziridine ring-opening,^4^ an engineered tryptophan synthase,^5^ multi-enzymatic β-methylation of AAs,^6^ and catalytic asymmetric hydrogenation of α,β-dehydro-amino acids (DHAAs), in its implementation with tetra-substituted substrates.^7^ This latter approach (Scheme 1(a)) has disclosed diastereo- and enantioselective entries to various classes of β-branched AAs, including^7*d*^ less common yet relevant β-trifluoromethyl AA derivatives.^8,9^ Given the common stereospecificity of the hydrogenation, the *E*/*Z* geometry of the substrate dictates the relative configuration of the product (Scheme 1(a)). Thus, a diastereoisomer is obtainable only if the parent olefin isomer can be prepared. This constraint can have negative implications. For example, a straightforward synthesis of a preclinical drug candidate *via* asymmetric hydrogenation of the corresponding β-aryl-β-trifluoromethyl DHAA was envisioned (Scheme 1(b)).^9*a*^ However, the required *E*-olefin isomer could not be accessed with sufficient selectivity. Furthermore, enantioselective hydrogenation of tetrasubstituted olefins can occasionally be challenging. In fact, the β-trifluoromethyl *Z*-DHAAs required for the target was found to be reluctant to asymmetric hydrogenation,^9*a*^ preventing application of Turner's formal stereodivergent reduction of DHAAs.^10^ This ingenious chemo-enzymatic approach, which unlocks access to the isomer not obtainable by hydrogenation *via* downstream inversion of the α-amino chirality centre in the reduced DHAA, is in fact established only for β-aryl-β-methyl AAs. Eventually, to obtain the target *syn*-trifluoromethylated amino alcohol,§§All through the paper, to identify the diastereomers of the β-branched β-trifluoromethyl AA derivatives, we have used Masamune's *syn* and *anti* descriptors, arbitrarily setting the β-aryl/alkyl group of these compounds in the main chain. Using CIP descriptors for the relative configuration (e.g. R*, S*), although more rigorous, would result in a less clear identification of the diastereoisomeric pairs. Alimardanov *et al.* resorted to a longer, yet effective and scalable, route, encompassing a chiral auxiliary and introduction of the amino functionality at a late stage.^9*a*^

With this background in mind, we envisioned an original stereodivergent^11^ entry to β-branched AAs grounded on the dynamic stereoselective ring-opening of enantioselectively reduced Erlenmeyer–Plöchl azlactones (Scheme 1(c)). In contrast with catalytic hydrogenation, this formal hydrogenation of the azlactone olefin (a DHAA derivative) fixes the configurations of the two hydrogenated centres in different steps, lending itself to stereodivergency. Herein, we present the first demonstration of this strategy by its application to the one-pot preparation of β-trifluoromethyl AA derivatives **4** (Scheme 1(d)). In more detail, enantioselective transfer hydrogenation of readily available substrates **1** (ref. 9a) with Hantzsch esters^12^ sets the absolute configuration of the trifluoromethylated β-centre.^13^ Subsequent dynamic alcoholytic ring-opening^14^ of intermediates **3** fixes the absolute configuration of the α-carbon. In the ring-opening reaction, the substrates **3** feature a considerable bias towards the formation of *anti*-isomers **4**. Such bias was readily leveraged with conventional *Cinchona* catalysts to obtain a range of *anti*-**4** products with very high stereoselectivities (including compounds not suited for hydrogenation). Conversely, the development of the *syn*-selective process was less straightforward, requiring a peculiar ammonia-derived squaramide catalyst to afford the *syn*-**4** isomers with variable diastereoselectivities.

**Fig. 1:**
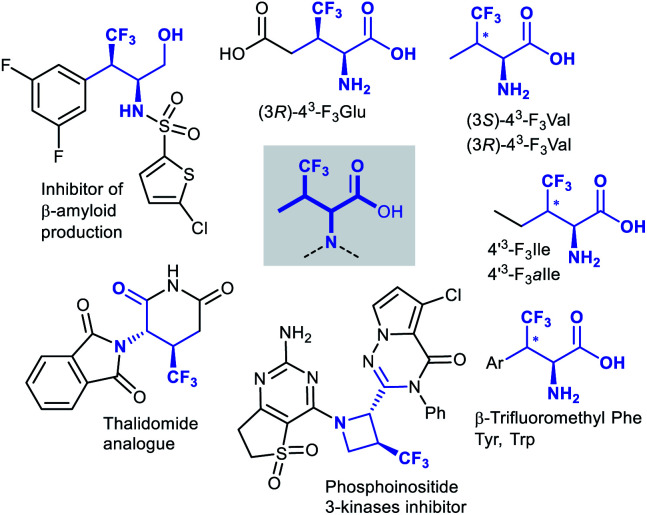
Medicinal chemistry relevant β-branched β-trifluoromethyl AAs.

It is worth stressing that β-branched β-trifluoromethyl AAs, and derivatives thereof, have found widespread interest in medicinal chemistry (Fig. 1). β-Trifluoromethylated analogues of natural AAs have been incorporated into peptides, wherein the trifluoromethyl group can give unique effects on stability, acidicy/basicity, folding behaviour, hydrophobicity, and ultimately biological activity.^8*a*^ The β-trifluoromethyl AA framework can also be found in less canonical structures. Besides the drug candidate mentioned in Scheme 1(b), which showcases inhibition of β-amyloid production,^15^ another β-trifluoromethyl AA structure of medicinal interest is an analogue of thalidomide,^2*i*^ which feature enhanced configurational stability compared to thalidomide. An additional example is represented by the heterocyclic compound derived from a 3-(trifluoromethyl)azetidine carboxylic acid, shown in Fig. 1. This compound is a member of a library of related heterocycles, investigated for their activity as inhibitors of phosphoinositide 3-kinases.^16^

## Results and discussion

At the outset, with transfer hydrogenation to β,β-disubstituted nitroalkenes promoted by Jacobsen-type catalysts as a lead,^17^ we explored different variables in the reduction of β-phenyl-β-trifluoromethyl Erlenmeyer–Plöchl substrates **1** with HEs.¶¶For a more comprehensive list of screening results, see ESI.† Preliminary screening of different catalysts and HEs identified the conditions outlined in Table 1, entry 1, as promising starting point. In more detail, using 20 mol% of catalyst **2a** in combination with the isobutyl HE, in dichloromethane (reagent grade) as solvent at low temperature (−30 °C), the 2-phenyl azlactone **1a** could be reduced with full conversion and promising enantioselectivity (70% ee). Products **3** of the transfer hydrogenation reaction are relatively unstable. Thus, for CSP HPLC analysis they are converted to the ultimate products **4** by ring-opening with allyl alcohol using achiral tertiary amine promoters, furnishing the two diastereoisomers **4**. These two isomers feature comparable ees, validating this method for the evaluation of the enantioselectivity of the reduction step. Thus, a systematic variation of the modules of this type of Jacobsen catalyst^18^ (AA portion, double H-bond donor, amide portion, terminal N-group), of C2 substituents of the azlactone, and of reaction conditions, was undertaken.¶ Exploration of catalysts based on double hydrogen bond donors other than thiourea (ureas and squaramides), and another AA portion (l-valine), confirmed the l-*tert*-leucine derived thiourea as the most efficient catalyst core. Whereas variation of solvent and/or dilution was not fruitful, investigation of different terminal N-groups in the thiourea (**2a–f**) indicated that the 4-(trifluoromethyl)phenyl residue (**2b**) could provide a distinct improvement, compared to the common 3,5-bis(trifluoromethyl)phenyl (**2a**) and other aryl/alkyl groups (**2c–f**) (entries 1–6). With catalyst **2b**, three differently C2 substituted Erlenmeyer–Plöchl azlactones **1b–d** were screened (entries 7–9). The 4-methoxyphenyl (PMP) derivative **1d** gave better results, and was thus selected for further catalyst development, which focussed on exploring various *N*-substituents on the amide (entries 10–14). Amongst catalysts **2g–k**, the *N*-benzyl-*N*-methyl amide derivative **2k** performed best, affording the transfer hydrogenation product **3d** with a respectable 85% ee, measured on the corresponding ring-opened derivative **4d**.

**Table 1:** Screening of azlactones **1** and catalysts **2** in the transfer hydrogenation reaction. Representative results^a^

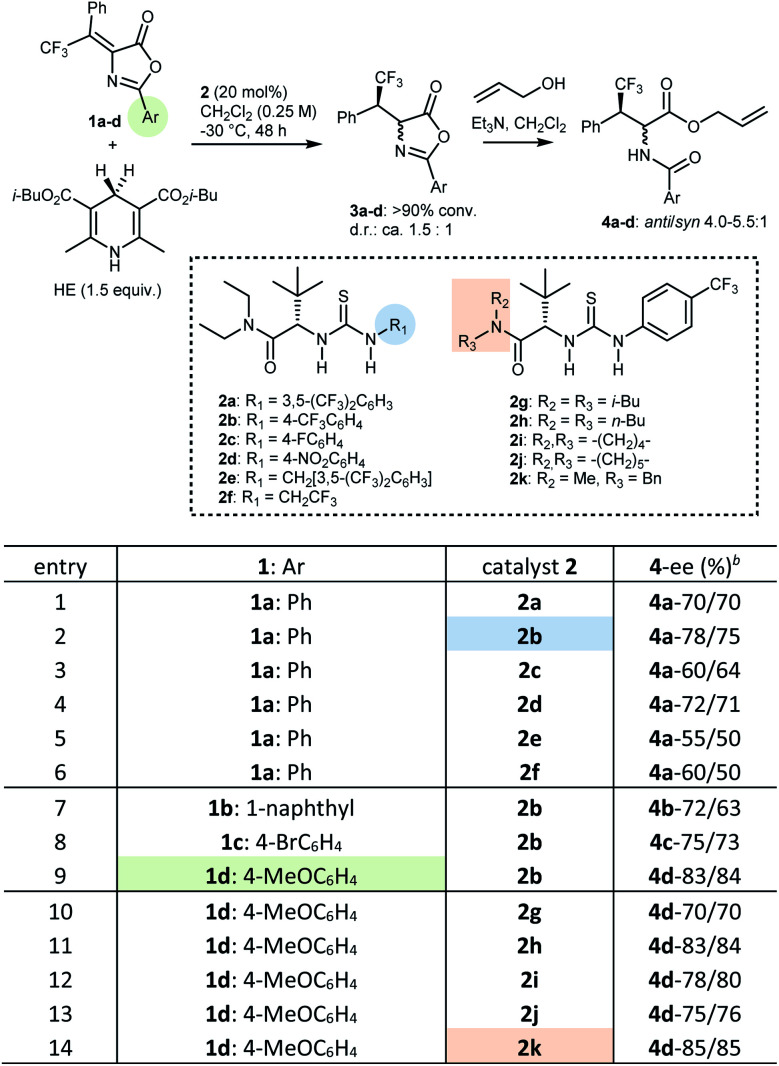

a Conditions: substrate **1** (0.05 mmol), catalyst **2** (0.01 mmol, 20 mol%), HE (0.075 mmol), CH_2_Cl_2_ (200 μL), −30 °C, 48 h. All reactions gave >90% conversion of **1** (^19^F NMR). Filtration on silica, evaporation, then CH_2_Cl_2_ (0.5 mL), allyl alcohol (0.1 mmol), Et_3_N (1 drop), RT, 24–48 h.

b Enantiomeric excess of *anti*-**4** and *syn*-**4**, respectively, determined by CSP HPLC after chromatographic purification on silica gel.

A closer inspection at the alcoholytic ring-opening step of compounds **3** promoted by achiral tertiary amines, indicated that the ring-opened products **4** were obtained with higher *anti*/*syn* ratios (5.5 : 1 for **4d**) than the parent azlactones **3** (*ca.* 1.5 : 1). Thus, the alcoholytic process was dynamic, and biased towards the *anti*-isomer. We initially surmised that such substrate-bias, hindering access to *syn*-**4**,^19^ would be circumscribed to the tertiary amine promoted alcoholytic ring-opening. Since a variety of DKRs of azlactones by ring-opening reactions, using different nucleophiles and catalytic approaches (Lewis bases, Lewis acids, Brønsted acids, enzymes), are available in the literature,^14^ we hoped that one of these could be subdued to our aims.

**Scheme 2:**
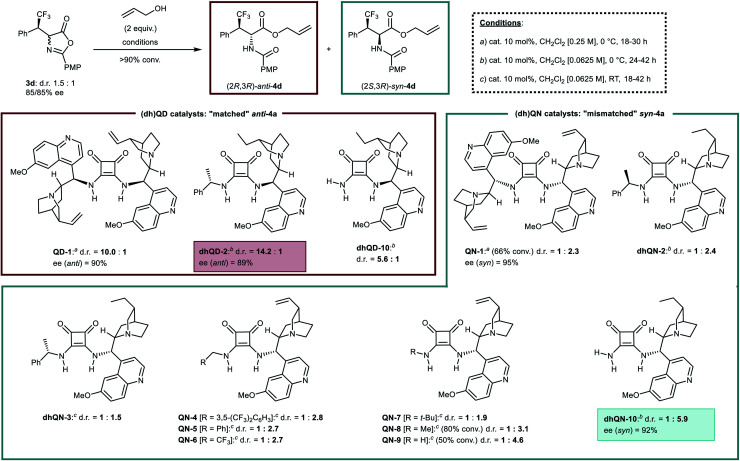
Optimization of the alcoholytic ring-opening step: selected results (d.r. values refer to *anti*/*syn* ratios).

However, a preliminary screening of many of these methods suggested the squaramide *Cinchona* catalyzed alcoholytic ring opening^20^ as most promising option, despite its resemblance with the biased achiral amine promoted reaction. State-of-the-art *Cinchona* squaramide dimeric catalysts derived from quinidine (**QD-1**) and quinine (**QN-1**) (Scheme 2) were initially employed with enantioenriched azlactone **3d** under standard conditions (dichloromethane, allyl alcohol, 0 °C). While, unsurprisingly, “matched” **QD-1** increased the *anti*/*syn* ratio to a high 10.0 : 1 value, compared to an achiral tertiary amine (*ca.* 5.5 : 1), we were pleased to observe that the corresponding “mismatched” **QN-1** could reverse the selectivity of the process, forcing the ring-opening reaction towards a moderate preference (1 : 2.3) for *syn*-**4d**. The products **4d** displayed a higher enantiomeric excess than **3d**, in accordance with the Horeau effect.^21^ Adjusting the reaction conditions and testing additional **QD** and dihydroquinidine (**dhQD**) derived structures led to a highly *anti*-selective protocol. Catalyst **dhQD-2** improved in fact the diastereomeric ratio of the product **4d** up to 14.2 : 1 in favour of the *anti*-isomer. However, application of its quasi-enantiomeric derivative **dhQN-2** did not result in the expected improvement in the *syn*-selectivity, providing a result similar to **QN-1** (1 : 2.4 *vs.* 1 : 2.3). This result emphasized that the transition states leading to the *anti* and *syn*-products are “intrinsically” diastereomeric,^19*b*^ due to the presence of the chiral (*R*)-configured β-branched chain of azlactone **3d**. On these grounds, different (*i.e.* non quasi-enantiomeric) catalyst structures may be required for *anti*- and *syn*-selective processes. Thus, a range of **(dh)QN** derived squaramide catalysts and reaction conditions were examined (see also ESI†). The diastereomeric catalyst **dhQN-3** (from (*S*)-α-methylbenzylamine instead of (*R*)-α-methylbenzylamine of **dhQN-2**) was tested first, giving however a poor result. Subsequent catalyst screening, performed at RT, suggested that the main factor affecting the stereoselectivity is the bulkiness of the squaramide portion. While catalysts **QN-4**, **5**, **6**, wherein the squaramide bears a methylene group, gave slight improvements compared to **dhQN-2** (1 : 2.7–2.8 *vs.* 1 : 2.4), the more bulky *tert*-butyl substituted **QN-7** provided a lower d.r. (1 : 1.9). The similar performances of the prototypical^22^ 3,5-bis(trifluoromethyl)benzyl catalyst **QN-4** and the simple benzyl derivative **QN-5** point to a negligible influence of the electronics of this group on selectivity. Thus, aiming at reducing bulkiness, catalyst **QN-8** derived from methylamine was applied, providing indeed a rewarding improvement (*anti*/*syn* = 1 : 3.1). A further reduction in bulkiness could be achieved only by entirely removing the *N*-substituent, which was finalized preparing and testing the ammonia derived catalyst **QN-9**. Pleasingly, this peculiar and unprecedented structure was able to afford *syn*-**4d** with a notable 1 : 4.6 selectivity. The very poor solubility of **QN-9** in CH_2_Cl_2_ resulted however in sluggish reactivity, with only 50% conversion after 42 h at RT. Such shortcoming was overcome by switching to the more soluble dihydroquinine derivative **dhQN-10**, which gave **4d** with >90% conversion, even by performing the reaction at 0 °C, and in a 1 : 5.9 diastereomeric ratio favouring the *syn*-isomer.^23^ Enantiomeric excess was found to be good (92%), as expected. At this stage, additional experiments indicated the unique requirements of the *syn*-selective reaction: catalyst **dhQD-10**, quasi-enantiomeric of **dhQN-10**, gave an *anti*-selectivity in the process comparable to an achiral tertiary amine (*ca.* 5 : 1).

Aiming at streamlining the overall process (**1d** → **4d**) by implementing a one pot procedure, thus circumventing the problematic purification of azlactone intermediate **3d**, it was found that an excess of HE in the transfer hydrogenation reaction has to be avoided, since this species inhibits the basic squaramide catalyst used in the ring-opening step (see ESI†). In contrast, the other components of the transfer hydrogenation (thiourea catalyst **2k** and pyridine co-product) do not interfere with the second step. Fortunately, it was possible to drive the transfer hydrogenation reaction to completion even by using just 1.1 equiv. of HE. Ultimately, this modification was sufficient to develop an efficient one-pot procedure.

**Scheme 3:**
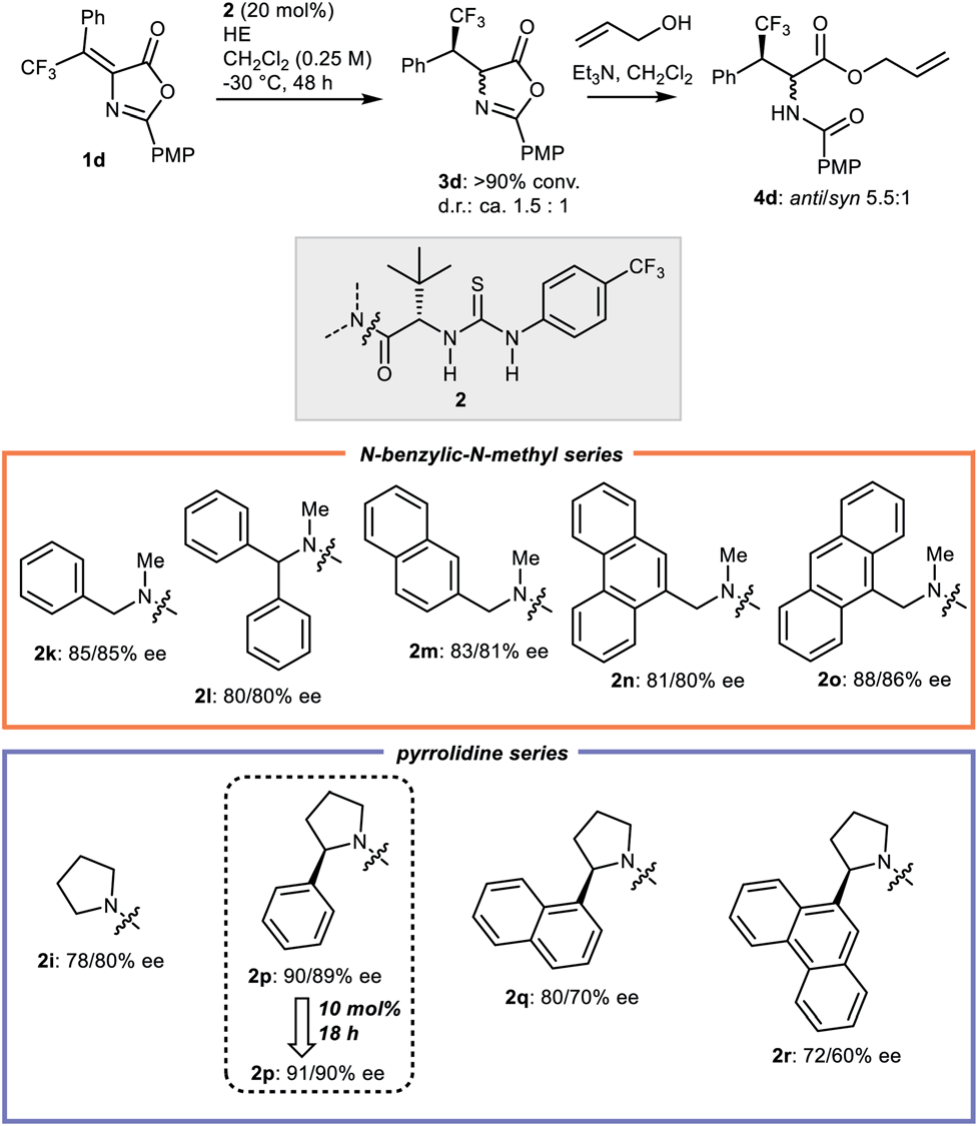
Second round of catalyst optimisation for the hydrogen transfer step: identification of optimal catalyst **2p**.

Then, in line with the notion that enhancing the enantiopurity of **3d** would result in additional improvement of *syn*-selectivity,^19^ an additional round of optimization of the catalyst used in the hydrogen transfer step was undertaken (Scheme 3). Different *N*-benzylic derivatives **2l–o** related to **2k**, and more elaborated Jacobsen catalysts bearing chiral 2-aryl pyrrolidin-1-yl amides^24^**2p–r** were applied, promptly leading to improvements. Indeed, compared to *N*-benzyl catalyst **2k**, the related 9-anthracenylmethyl derived structure **2o** and the (2*R*)-2-phenylpyrrolidine amide **2p** afforded azlactone **3a** with higher enantioselectivities, with catalyst **2p** providing better results (91/90% ee), even at lower catalyst loading (10 *vs.* 20 mol%), and in shorter reaction time (18 *vs.* 48 h).

**Fig. 2:**
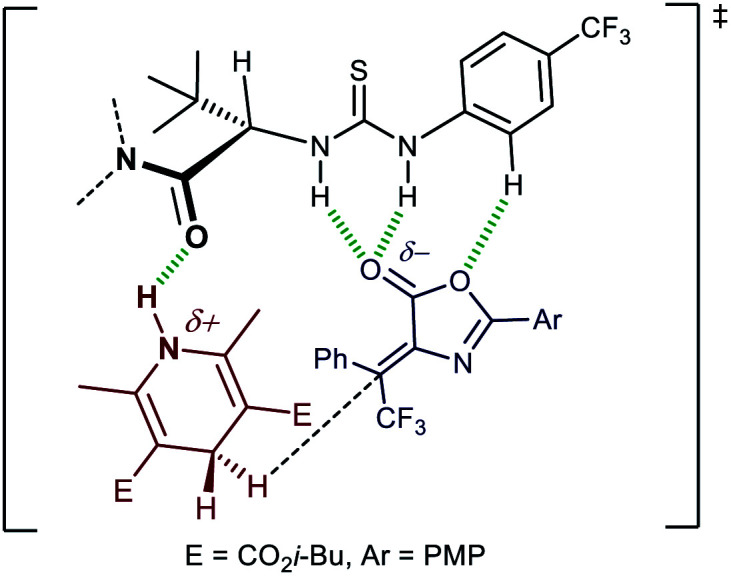
Tentative transition state model for the transfer hydrogenation reaction.

A tentative transition state picture can be built from a computationally validated model for the transfer hydrogenation of nitroalkenes with HEs catalysed by Jacobsen-type thiourea catalysts (**2**),^17*c*^ complemented with recognition studies of lactones by a thiourea.^25^ Coordination of the acidic thiourea hydrogens to the lactone moiety, possibly assisted by its *N*-aryl group, and simultaneous stabilisation of the positive charge on the HE by the amide oxygen, are the key interactions between catalyst and substrates (Fig. 2). The *tert*-butyl group serves to “lock” the conformation of the catalyst as shown, thus leading to a match between the catalyst polar functionalities and a transition state leading to (3*R*)-**3d**. While this model does not help rationalizing the subtle effects of the amide and the thiourea aryl groups on the enantioselectivity of the reaction, it reconciles with the observed comparably high, but opposite, sense of enantioinduction exerted by catalyst **2p** on the two isomeric olefins (*i.e. Z*-**1** → (3*R*)-**3**, and *E*-**1** → (3*S*)-**3**, see ESI†). From the experimental results shown in Scheme 3 (compare **2p** with **2q** and **2r**), the often encountered positive relationship between the extension of the π-system of the 2-substituent of the pyrrolidine and the enantioselectivity^24*b*^ is not apparent. Stabilising cation-π interactions might not be helpful to selectivity in this case.

**Scheme 4:**
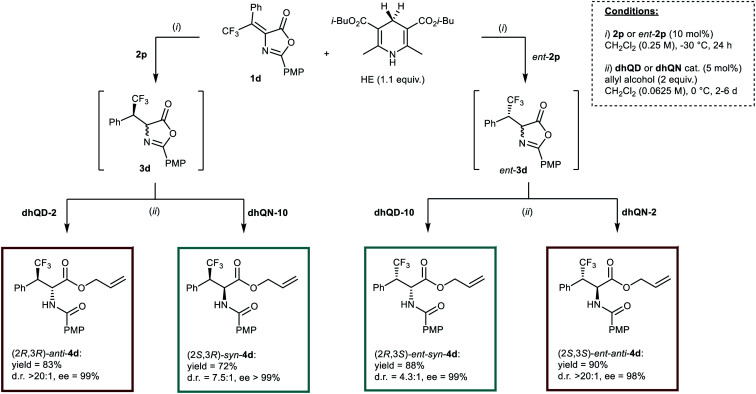
Diastereodivergent, enantioselective synthesis of the whole set of stereoisomers of **4d** by applying different catalysts combinations in the one-pot process.

The stage was thus set for the full unravelment of the stereodivergent methodology (Scheme 4). It is clear from the results reported that the improvement in enantioselectivity provided by catalyst **2p** in the first step was indeed beneficial to the diastereoselectivities of the whole processes. Its combination with catalyst **dhQD-2** furnished *anti*-**4d** in good yield and in essentially diastereo- and enantiopure form, while use of “mismatched” **dhQN-10** in the second step afforded *syn*-**4d** in 72% yield and >99% ee, with a notable 7.5 : 1 diastereomeric ratio. These results are to be compared with the 14.2 : 1 d.r. and 89% ee for *anti*-**4d**, and the 5.9 : 1 d.r. and 92% ee for *syn*-**4d** obtained when catalyst **2k** was used in the first step (Scheme 2). Scheme 4 shows also how the different combinations of catalysts (**2p** and *ent*-**2p**, **dhQD-2** and **dhQN-2**, **dhQD-10** and **dhQN-10**) could permit the obtainment of the full set of stereoisomeric products **4d** with moderate to excellent results. Moreover, although the one pot protocol required longer reaction times (2–6 days instead of 24 h), for the ring opening step, the *Cinchona* loading could be lowered to 5 mol% without affecting the selectivity of the process.

The scope of the one-pot procedure was then studied (Table 2), by first applying a range of β-trifluoromethyl Erlenmeyer–Plöchl azlactones **1d–j**, bearing electron-donating (**1g**, **i**) or electron-withdrawing (**1e**, **f**, **h**, **j**) groups at the β-aryl ring, and β-heteroaromatic substituents (**1k**, **l**). Entries 1–9 show that these substrates behaved very well in the *anti*-selective reaction, providing the corresponding *anti*-**4d-l** with results comparable to the parent *anti*-**4d**, that is, in good yields and outstanding diastereo- and enantioselectivities. The *syn*-selective processes provided variable results in terms of diastereoselectivities, ranging from a fully satisfactory 8.5 : 1 value for product *syn*-**4g** to less pleasing *ca.* 2 : 1 results for the β-heteroaromatic derivatives *syn*-**4k** and *syn*-**4l**. The latter results can be ascribed to a very high substrate bias towards *anti*-**4k**, **l** in the ring-opening process (>10 : 1 employing Et_3_N), rather than to poor catalyst **dhQN-10** efficiency. *Syn*-**4k** and *syn*-**4l** were also obtained in lower yields compared to the other compounds. Nevertheless, the enantiomeric excesses of the major *syn*-**4** isomers were found to be excellent in all cases examined (≥99% ee). Substrate **1m** bearing a β-perfluoro residue rendered results similar to the β-heteroaromatic derivatives **1k** and **1l**, that is, excellent selectivity in the *anti*-**4m** isomer, and moderate yield and diastereoselectivity, but with >99% ee, for the *syn*-**4m** diastereoisomer (entry 10). The application of β-aliphatic substrates **1n–p** required an adjustment to the conditions used in the transfer hydrogenation step, which was performed with higher (20 mol%) catalyst loading and at higher temperatures (−20 °C for the ethyl and methyl derivatives **1o** and **1p**, 0 °C for the more hindered cyclohexyl counterpart **1n**). With these adjustments, it was possible to obtain *anti*-**4n–p** with good selectivities, while the results for *syn*-**4n–p** vary from the satisfactory level of *syn*-**4n** to the less pleasing *syn*-selectivity for **4p** (entries 11–13). The latter result was ascribed to the deleterious combination of moderate enantioselectivity in the transfer hydrogenation step (*ca.* 70% ee) with high substrate bias in the ring-opening step (*ca.* 10 : 1). The last three entries 14–16 of Table 2 display the results obtained by applying alcohols other than allyl in the alcoholytic process with substrate **1d**. The peculiarity of the present reaction system makes the tolerance to different primary alcohols, known for the DKR of simple azlactones,^20^ less than obvious, especially in the case of the *syn*-selective protocol. However, it was pleasing to observe that results in line with the allyl derivatives **4d** were obtained for the products of methyl, benzyl and isobutyl alcohols **4q–s**, although lower yields were observed in the latter case.||||For limitations in terms of substrate variations, see ESI.†

**Table 2:** Reaction scope of the *anti*-**4** and *syn*-**4** selective processes

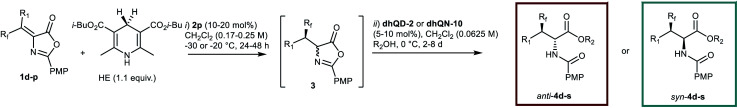										
Entry	**1**: R_1_, R_f_	R_2_	*Anti*-**4** selective process^a^				*Syn*-**4** selective process^a^			
			*Anti*-**4**	Yield^b^ (%)	*Anti*/*syn*^c^	ee^d^ (%)	*Syn*-**4**	Yield^b^ (%)	*Syn/anti* c	ee^d^ (%)
1	**1d**: C_6_H_5_, CF_3_	Allyl	*anti*-**4d**	83	>20 : 1	99	*syn*-**4d**	72	7.5 : 1	>99
2	**1e**: 4-BrC_6_H_4_, CF_3_	Allyl	*anti*-**4e**	93	>20 : 1	98	*syn*-**4e**	72	3.6 : 1	99
3	**1f**: 4-ClC_6_H_4_, CF_3_	Allyl	*anti*-**4f**	90	>20 : 1	99	*syn*-**4f**	77	4.4 : 1	99
4	**1g**: 3-MeC_6_H_4_, CF_3_	Allyl	*anti*-**4g**	96	>20 : 1	98	*syn*-**4g**	84	8.5 : 1	99
5	**1h**: 4-FC_6_H_4_, CF_3_	Allyl	*anti*-**4h**	88	>20 : 1	98	*syn*-**4h**^e^	78	4.4 : 1	99
6	**1i**: 4-MeOC_6_H_4_, CF_3_	Allyl	*anti*-**4i**^e^^,^^g^	90	>20 : 1	98	*syn*-**4i**^e^^,^^f^	60	3.7 : 1	>99
7	**1j**: 3,5-F_2_C_6_H_3_, CF_3_	Allyl	*anti*-**4j**	85	>20 : 1	99	*syn*-**4j**^e^	87	4.0 : 1	>99
8	**1k**: 2-thienyl, CF_3_	Allyl	*anti*-**4k**	78	>20 : 1	97	*syn*-**4k**^g^	37	2.1 : 1	99
9	**1l**: N-Ts-indol-3-yl, CF_3_	Allyl	*anti*-**4l**	82	>20 : 1	96	*syn*-**4l**^g^	53	2.3 : 1	>99
10	**1m**: C_6_H_5_, CF_3_CF_2_CF_2_	Allyl	*anti*-**4m**	69	>20 : 1	98	*syn*-**4m**^g^^,^^h^	50	2.0 : 1	>99
11^i^	**1n**: cyclohexyl, CF_3_	Allyl	*anti*-**4n**^e^	98	16.7 : 1	99	*syn*-**4n**^e^^,^^f^	65	3.6 : 1	>99
12^i^	**1o**: Et, CF_3_	Allyl	*anti*-**4o**	98	15.3 : 1	97	*syn*-**4o**^e^	78	2.6 : 1	>99
13^i^	**1p**: Me, CF_3_	Allyl	*anti*-**4p**	97	10.1 : 1	89	*syn*-**4p**^e^	85	1.3 : 1	99
14	**1d**: C_6_H_5_, CF_3_	Me	*anti*-**4q**	91	>20 : 1	97	*syn*-**4q**	77	5.3 : 1	>99
15	**1d**: C_6_H_5_, CF_3_	Bn	*anti*-**4r**	81	18 : 1	98	*syn*-**4r**^e^	70	5.9 : 1	99
16	**1d**: C_6_H_5_, CF_3_	*i*-Bu	*anti*-**4s**^ee^	50	>20 : 1	96	*syn*-**4s**^e^^,^^g^	50	6.7 : 1	99

a Conditions: **1** (0.15 mmol), HE (0.165 mmol, 1.1 equiv.), **2p** (0.015 mmol, 10 mol%), CH_2_Cl_2_ (0.60 mL), −30 °C, 24–48 h, then CH_2_Cl_2_ (1.8 mL), **dhQD-2** for *anti*-**4** or **dhQN-10** for *syn*-**4** (0.0075 mmol, 5 mol%), R^2^OH (0.30 mmol, 2 equiv.), 0 °C, 2–8 d.

b Isolated yield of combined diastereoisomers **4** after chromatography on silica gel.

c Determined on the crude mixtures by ^19^F NMR spectroscopy.

d Enantiomeric excess of major diastereoisomer, determined by CSP HPLC.

e In the ring-opening step, after 2–5 d, additional catalyst **dhQD-2** for *anti*-**4** or **dhQN-10** for *syn*-**4** (0.0075 mmol) and R^2^OH (0.15 mmol, 1–2 equiv.), were added.

f Ring-opening step warmed to RT after 2 d.

g Two step reaction performed by isolating intermediate **3** by a rapid filtration on silica gel.

h Reduction step performed at 0 °C.

i Conditions: **1** (0.05 mmol), HE (0.055 mmol, 1.1 equiv.), **2p** (0.01 mmol, 20 mol%), CH_2_Cl_2_ (0.300 mL), −20 or 0 °C, 24–48 h, then CH_2_Cl_2_ (0.60 mL), **dhQD-2** for *anti*-**4** or **dhQN-10** for *syn*-**4** (0.01 mmol, 10 mol%), allyl alcohol (0.1 mmol, 2 equiv.), 0 °C, 3–6 d.

**Scheme 5:**
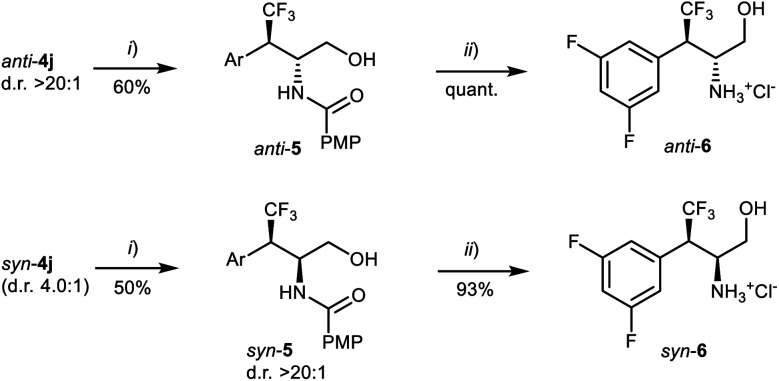
Conversion of the catalytic products **4j** to the corresponding amino alcohol hydrochlorides *anti*-**6** and *syn*-**6.** (i) NaBH_4_, THF/H_2_O, 0 °C → RT, then column chromatography on silica gel. (ii) HCl, MeOH/H_2_O, reflux, then work up and evaporation.

Compounds **4j** were separately subjected to a two-step reduction-hydrolysis sequence (Scheme 5), delivering the corresponding aminoalcohol hydrochlorides **6**, *via* amides **5**. It is worth stressing that neither *syn*-**6** – intermediate *en route* to the drug candidate (see Scheme 1(b))^9*a*^ – nor *anti*-**6** can be easily accessed by conventional asymmetric hydrogenation.^7*d*^

## Conclusions

We have proved that the conceptually new combination of two catalytic processes (transfer hydrogenation – dynamic ring-opening) on Erlenmeyer–Plöchl azlactones can provide a new stereodivergent strategy to enantioenriched β-branched AAs. The realization of this tactic with trifluoromethylated substrates has disclosed a one-pot entry to β-aryl-β-trifluoromethyl AA derivatives. Using the appropriate catalyst combination, the *anti*-bias of the ring-opening reaction was leveraged, giving *anti*-products with excellent stereoselectivities (d.r. up to >20 : 1, ee ≥ 89%). The scope of this reaction includes substrates reluctant to enantioselective hydrogenation. A newly designed ammonia derived squaramide catalyst afforded the *syn*-isomers, not obtainable by hydrogenation, with variable diastereoselectivities (d.r. up to 8.5 : 1) and high enantioselectivities (ee ≥ 99%).

## Author contributions

Conceptualization and supervision: VC and LB. Investigation and methodology: all authors. Writing - original draft: LB. Writing - review and editing: VC, MF, LB. Funding acquisition, project administration and resources: MF and LB.

## Conflicts of interest

There are no conflicts to declare.

## Supplementary Material

SC-012-D1SC01442K-s001
