# Supplementary material for: Stereodivergent entry to β-branched β-trifluoromethyl α-amino acid derivatives by sequential catalytic asymmetric reactions
Source: Chem Sci. 2021 Jun 29;12(30):10233–41. doi: 10.1039/d1sc01442k (PMC8336586; doi:10.1039/d1sc01442k)

Electronic Supplementary Material (ESI) for Chemical Science.
This journal is © The Royal Society of Chemistry 2021

# Electronic Supplementary Information

# Stereodivergent entry to β-branched β-trifluoromethyl α-amino acid derivatives by sequential catalytic asymmetric reactions

*Vasco Corti,† Riccardo Riccioli, Ada Martinelli, Sofia Sandri, Mariafrancesca Fochi,* and Luca Bernardi**

Department of Industrial Chemistry "Toso Montanari" and INSTM RU Bologna
Alma Mater Studiorum – University of Bologna
V. Risorgimento 4, 40136 Bologna (Italy)
E-mail: mariafrancesca.fochi@unibo.it; luca.bernardi2@unibo.it
†Department of Chemistry, Aarhus University, 8000 Aarhus (Denmark).

# Table of contents

Optimization of the transfer hydrogenation step – representative results........................................................1

Optimization of the ring-opening step – additional results ..................................................................................6

Ring-opening step – additional discussion ...........................................................................................................8

One pot procedure – control results ..................................................................................................................20

Olefin geometry and enantioinduction in the transfer hydrogenation reaction.................................................21

Ring-opening step – tentative reaction models..................................................................................................23

Limitations of the methodology ..........................................................................................................................26

Experimental section ..........................................................................................................................................28

General Methods...............................................................................................................................................28

Materials:...........................................................................................................................................................28

Synthesis and characterization of Erlenmeyer-Plöchl azlactone derivatives **1**. ........................................30

Copies of $^{1}H$, $^{19}F$, and $^{13}C$ NMR spectra of substrates **1** (in $CDCl_3$) ......................................................36

Synthesis[19] and characterization of 4-(trifluoromethyl)phenyl thiourea catalysts **2k** and **2p**.......................62

Copies of $^{1}H$, $^{19}F$, and $^{13}C$ NMR spectra of catalysts **2k**, **2p** (in $CDCl_3$)................................................64

Synthesis[22] and characterization of **dhQN** and **dhQD** catalysts. ...............................................................68

Copies of $^{1}H$ and $^{13}C$ NMR spectra of catalysts **dhQD-2**, **dhQD-10**, **dhQN-2**, **dhQN-10** (in DMSO-$d_6$ at 60 °C)...........................................................................................................................................72

Synthesis and characterization of catalytic products **4**, amidoalcohols **5** and aminoalcohols **6**..................76

General procedure A for the one pot synthesis of products **4**..............................................................76

General procedure B for the synthesis of products **4**.............................................................................76

General procedure C for the synthesis of products **4**.............................................................................77

General procedure for the synthesis of racemic products **4**..................................................................78

Reduction of the ester moiety of products *anti*-**4j** and *syn*-**4j**..............................................................98

Hydrolysis of the amide moiety of products *anti*-**5** and *syn*-**5**, and assignment of the relative and absolute configuration of the products.........................................................................................................100

Copies of $^{1}H$ and $^{13}C$ NMR spectra of products **4**, **5**, **6** (in $CDCl_3$ unless otherwise stated) .................102

Copies of HPLC Traces of products **4** and **5**........................................................................................174

# Optimization of the transfer hydrogenation step – representative results

**Scheme S1**. Preliminary catalyst screening:

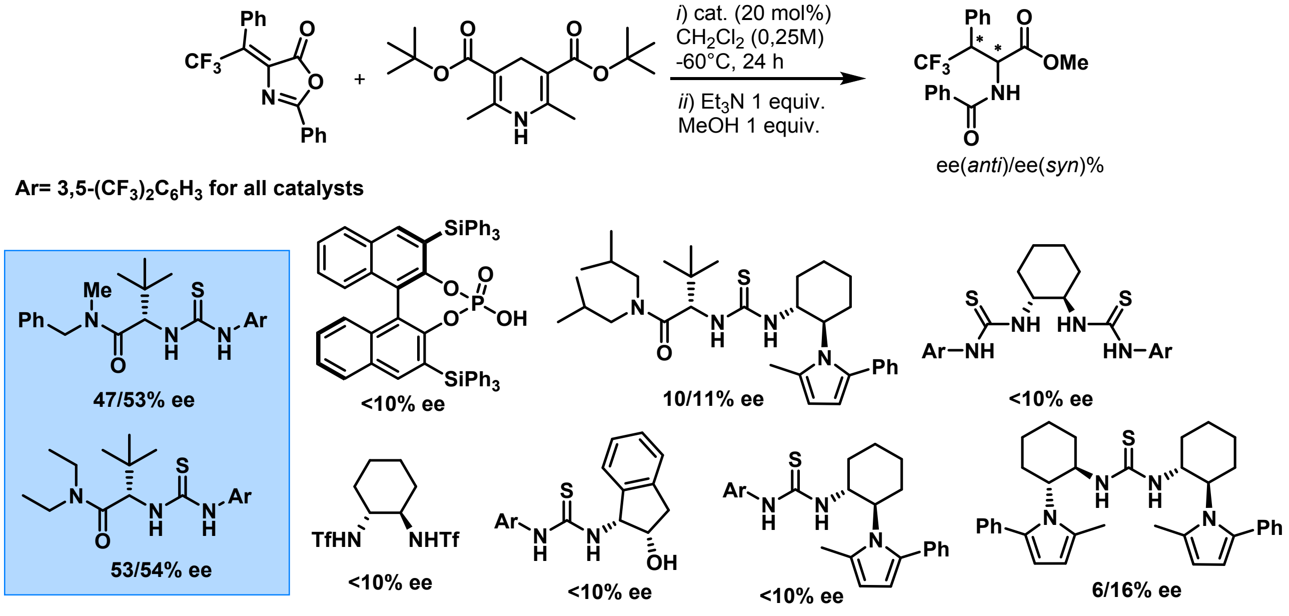


**Scheme S2**. First screening of reductants (Hantzsch esters and benzothiazoline):

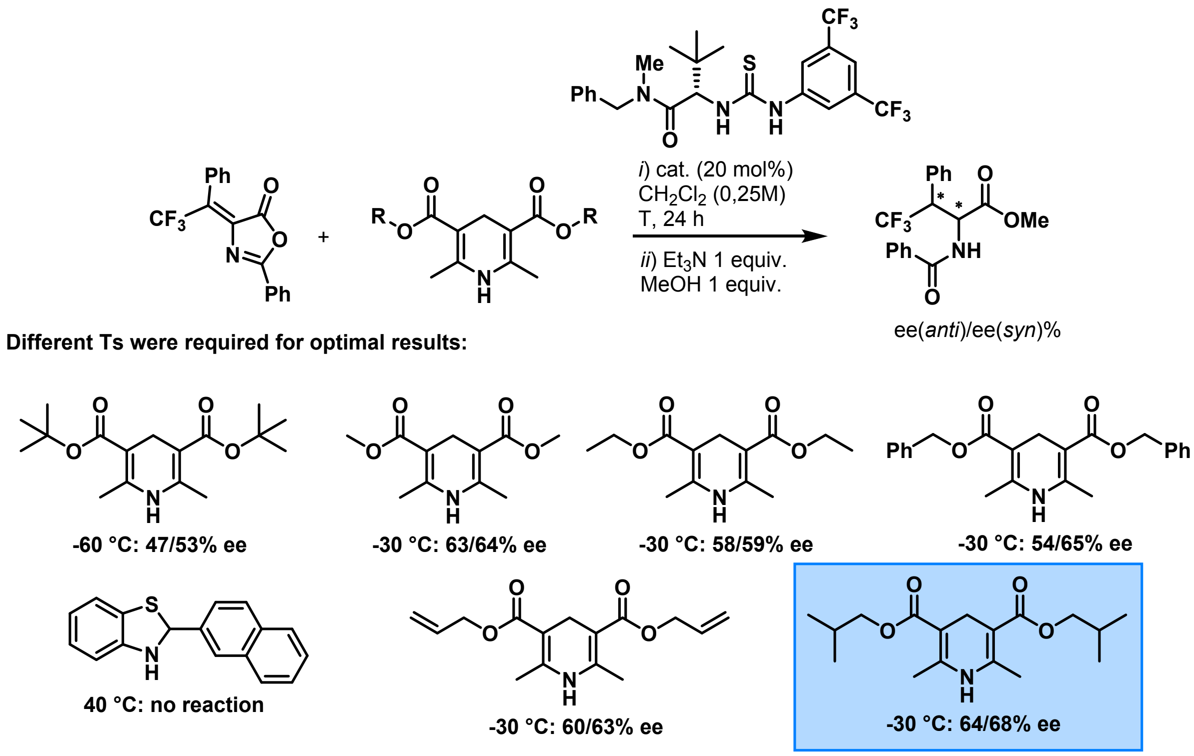

**Scheme S3**. Screening of double H-bond donors and amino acid residues of the catalyst:

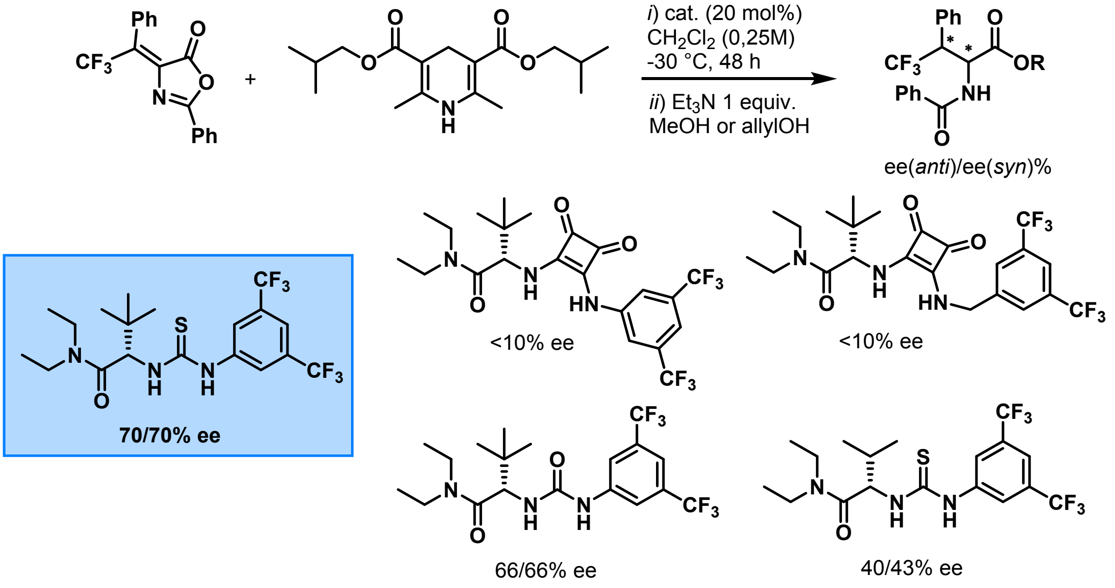


**Scheme S4**. Screening of solvents including dilution:

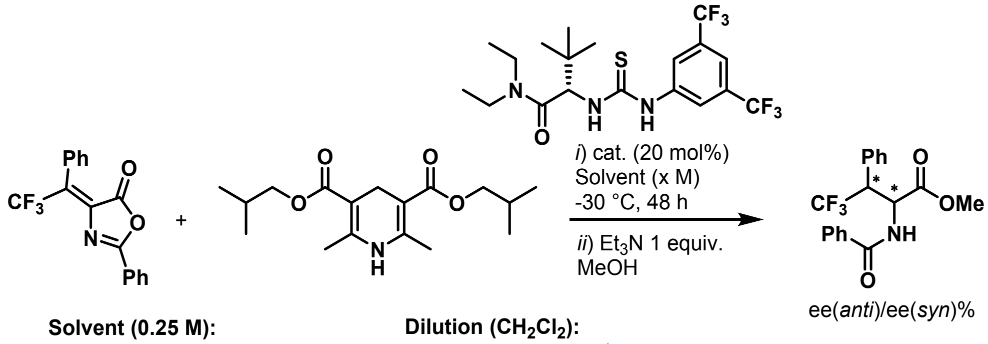


**Solvent (0.25 M):**

$CH_2Cl_2$: 70/70%ee
$CHCl_3$: 41/43% ee
Toluene: 46/43% ee
MTBE: <10% ee
EtOAc: 6/14% ee
$CH_3CN$: 16/20% ee

**Dilution ($CH_2Cl_2$):**

0.25 M: 70/70% ee
0.125 M: 67/69% ee
0.5 M: 67/65% ee

**Scheme S5**. Screening of terminal N-groups of the catalyst:

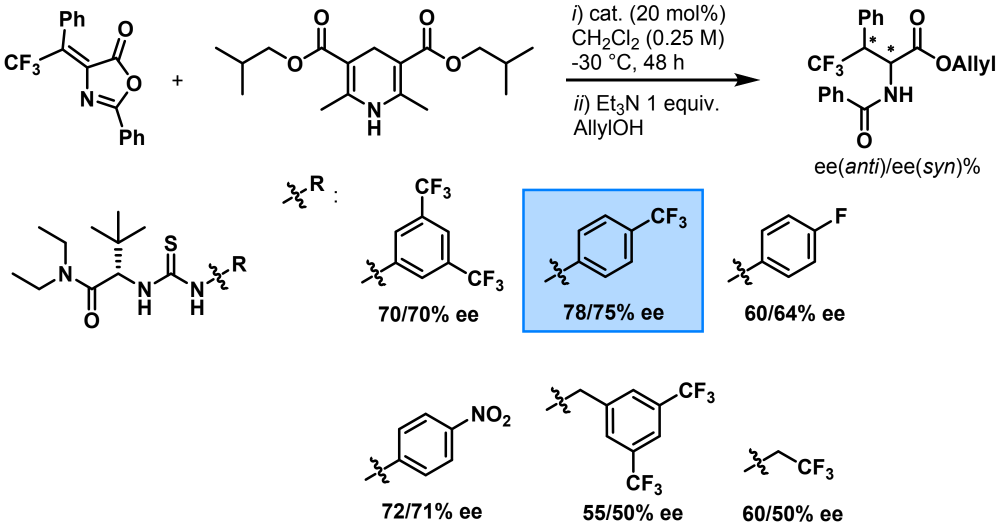


**Scheme S6**. Screening of C5-residues of azlactone:

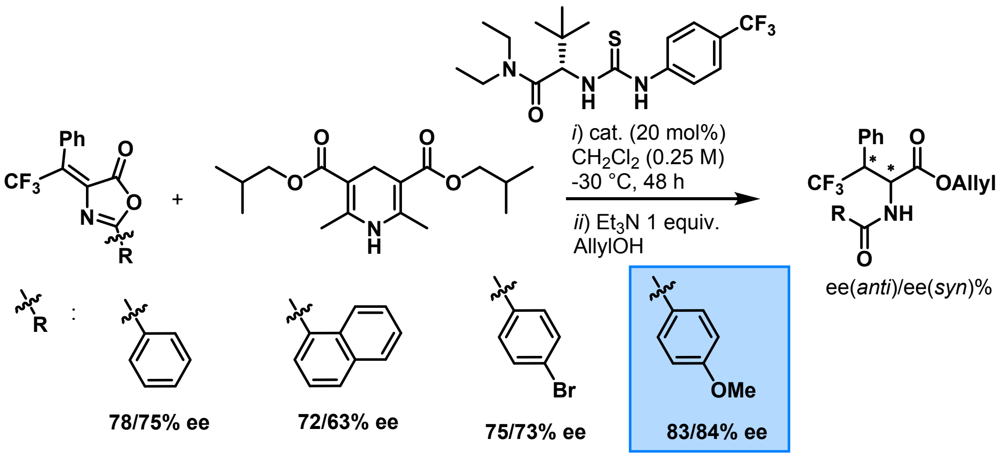

**Scheme S7**. First screening of amide residues of the catalyst:

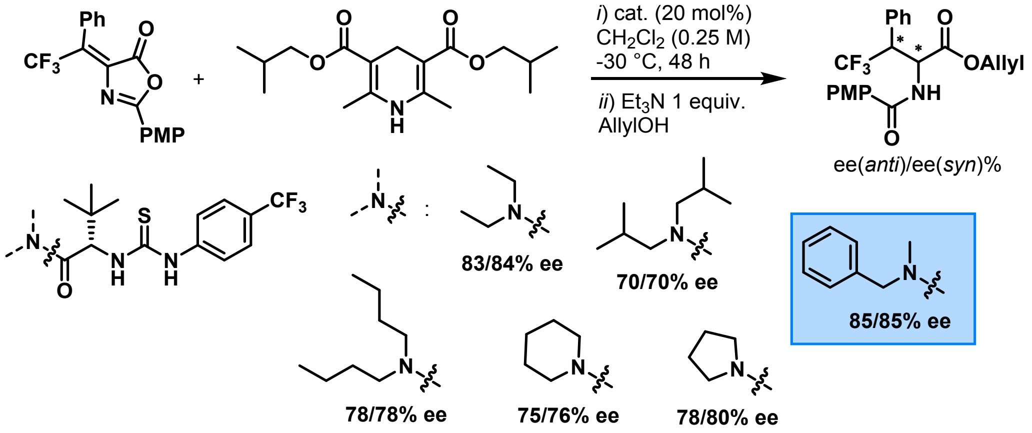


**Scheme S8**. Second screening of Hantzsch esters:

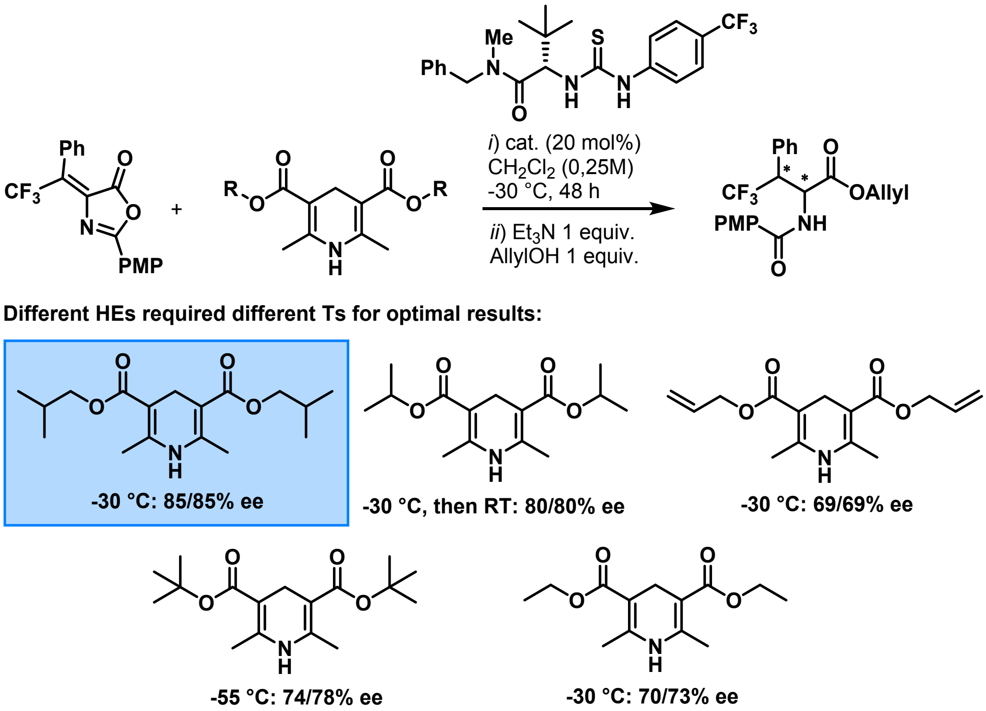

**Scheme S9**. Second screening of amide residues of the catalyst:

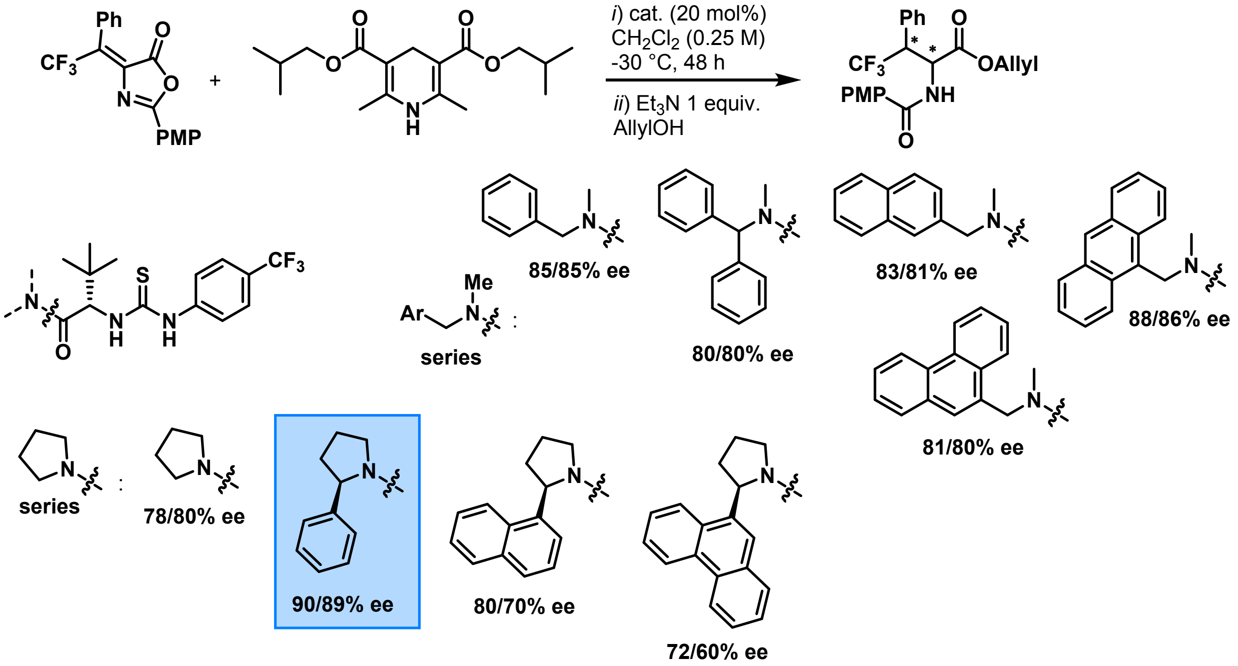


**Scheme S10**. Catalyst loading, reaction temperature and time: optimized conditions.

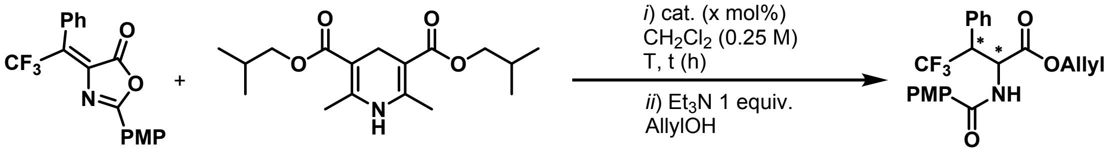


| Conditions | Results |
|---|---|
| 20 mol% cat.<br>-30 °C, 18 h | 90% conv., 90/89% ee |
| 20 mol% cat.<br>0 °C, 15 h | >95% conv., 82/83% ee |
| 10 mol% cat.<br>-30 °C, 18 h | >95% conv., 91/90% ee |
| 5 mol% cat.<br>-30 °C, 40 h | >95% conv., 88/86% ee |

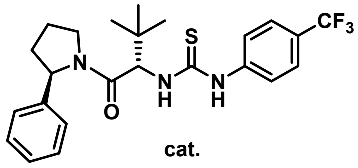


cat.

# Optimization of the ring-opening step – additional results

**Chart S1**. Representative **QN** catalysts ("mismatched") screened (Ar = 3,5-$(CF_3)_2C_6H_3$).

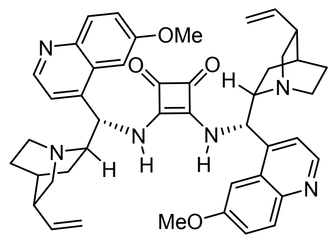

**I**

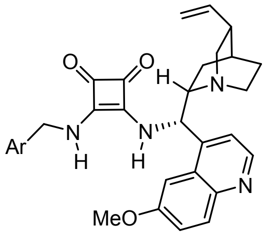

**II**

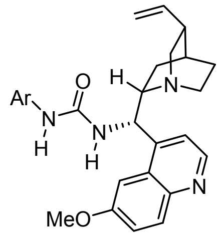

**III**

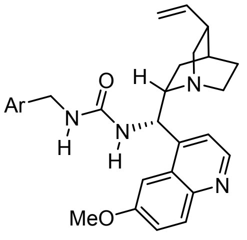

**IV**

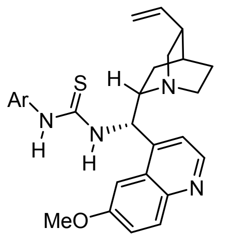

**V**

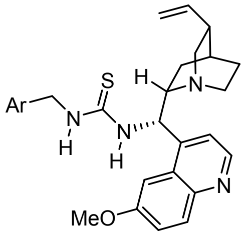

**VI**

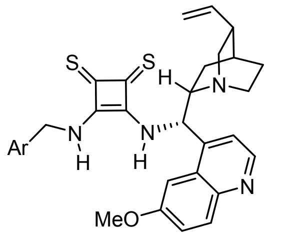

**VII**

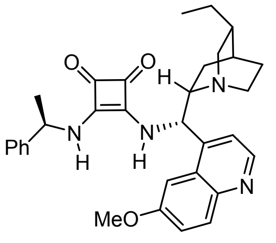

**VIII**

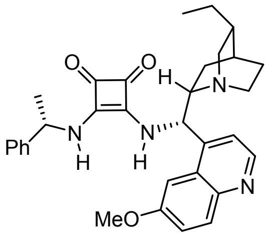

**IX**

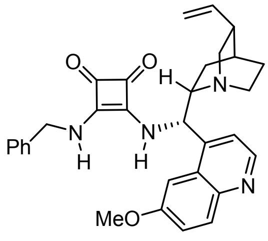

**X**

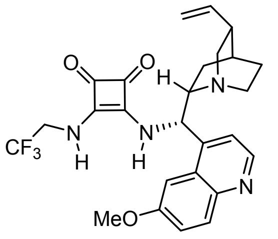

**XI**

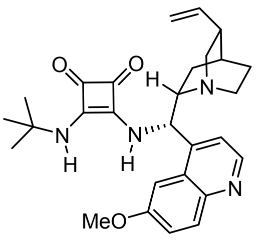

**XII**

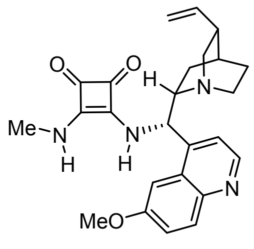

**XIII**

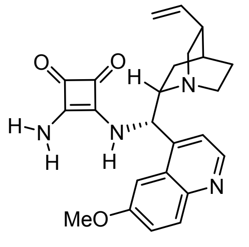

**XIV**

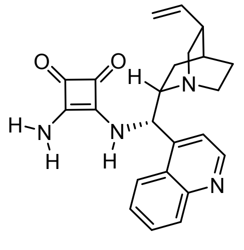

**XV**

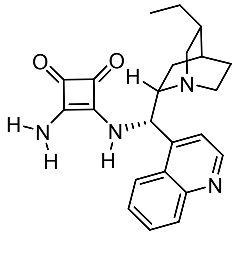

**XVI**

**Table S1**: These experiments were carried out on the isolated enantioenriched (85% ee of both diastereomers) **3d** obtained from the catalytic asymmetric hydrogen transfer of substrate **1d** and catalyst **2k**.

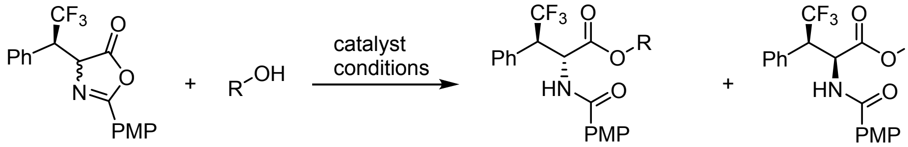


**3d**: d.r.: 1.5 : 1
85%/85% ee

*anti*-**4d**

*syn*-**4d**

| Entry[a] | Cat. | R | Solvent (M) | T (°C) | t (h) | Conv. (%)[b] | d.r. (*syn*/*anti*-**4**)[b] | ee major *syn*-**4** (%)[c] |
|---|---|---|---|---|---|---|---|---|
| 1 | **I** | Me | $CH_2Cl_2$ (0.25) | 0 | 30 | 56 | 1.4:1 | - |
| 2 | **I** | Allyl | $CH_2Cl_2$ (0.25) | 0 | 30 | 66 | 2.3:1 | 95 |
| 3 | **I** | Bn | $CH_2Cl_2$ (0.25) | 0 | 18 | 91 | 2.0:1 | - |
| 4 | **I** | *i*-Pr | $CH_2Cl_2$ (0.25) | r.t. | 30 | - | - | - |
| 6 | **II** | Allyl | $CH_2Cl_2$ (0.25) | 0 | 18 | >95 | 2.4:1 | - |
| 7 | **II** | Allyl | $CHCl_3$ (0.25) | r.t. | 18 | >95 | 1.7:1 | - |
| 8 | **II** | Allyl | DCE (0.25) | r.t. | 18 | >95 | 2.0:1 | - |
| 9 | **II** | Allyl | PhMe (0.25) | r.t. | 18 | >95 | 1.3:1 | - |
| 10 | **II** | Allyl | THF (0.25) | r.t. | 18 | 74 | 1.4:1 | - |
| 11 | **II** | Allyl | $CH_3CN$ (0.25) | r.t. | 18 | 80 | 1.1:1 | - |
| 12 | **II** | Allyl | $CH_2Cl_2$ (0.5) | r.t. | 18 | >95 | 2.2:1 | - |
| 13 | **II** | Allyl | $CH_2Cl_2$ (0.125) | r.t. | 18 | >95 | 2.5:1 | - |
| 14 | **II** | Allyl | $CH_2Cl_2$ (0.0625) | r.t | 18 | >95 | 2.8:1 | - |
| 15[d] | **II** | Allyl | $CH_2Cl_2$ (0.0625) | r.t. | 18 | >95 | 2.8:1 | - |
| 16[e] | **II** | Allyl | $CH_2Cl_2$ (0.0625) | r.t. | 24 | 73 | 2.1:1 | - |
| 17[d] | **III** | Allyl | $CH_2Cl_2$ (0.0625) | r.t. | 68 | 90 | 1.8:1 | |
| 18[d] | **IV** | Allyl | $CH_2Cl_2$ (0.0625) | r.t. | 68 | >95 | 1.1:1 | |
| 19[d] | **V** | Allyl | $CH_2Cl_2$ (0.0625) | r.t. | 96 | 94 | 1.5:1 | |
| 20[d] | **VI** | Allyl | $CH_2Cl_2$ (0.0625) | r.t. | 96 | >95 | 1.6:1 | |
| 21 | **VII** | Allyl | $CH_2Cl_2$ (0.0625) | r.t. | 19 | 63 | 1:1.3 | - |
| 21 | **VIII** | Allyl | $CH_2Cl_2$ (0.0625) | r.t. | 42 | >95 | 2.4 : 1 | - |
| 22[d] | **IX** | Allyl | $CH_2Cl_2$ (0.0625) | r.t. | 42 | >95 | 1.5 : 1 | - |
| 23 | **X** | Allyl | $CH_2Cl_2$ (0.0625) | r.t. | 42 | >95 | 2.7 : 1 | - |
| 24 | **XI** | Allyl | $CH_2Cl_2$ (0.0625) | r.t. | 42 | >95 | 2.7 : 1 | - |
| 25 | **XII** | Allyl | $CH_2Cl_2$ (0.0625) | r.t. | 42 | >95 | 1.9 : 1 | - |
| 26 | **XIII** | Allyl | $CH_2Cl_2$ (0.0625) | r.t. | 42 | 80 | 3.1 : 1 | - |
| 27 | **XIV** | Allyl | $CH_2Cl_2$ (0.0625) | r.t. | 42 | 50 | 4.6 : 1 | - |
| 28[d,f] | **XV** | Allyl | $CH_2Cl_2$ (0.0625) | r.t. | 22 | 59 | 4.2 : 1 | - |
| 29 | **XVI** | Allyl | $CH_2Cl_2$ (0.0625) | r.t. | 24 | >95 | 4.9 : 1 | - |
| 30 | **XVI** | Allyl | $CH_2Cl_2$ (0.0625) | 0 | 24 | >95 | 5.9 : 1 | 92 |

[a] Conditions: **3d** (1 equiv), ROH (2 equiv), solvent, catalyst (10 mol%). [b] Determined on the crude mixture by $^{19}F$ NMR analysis. [c] Determined by CSP-HPLC on the purified product. [d] Reaction carried out with 5 mol% catalyst. [e] Reaction carried out with 2.5 mol% catalyst. [f] $NaHCO_3$ (1 equiv.) was used as additive.

## Ring-opening step – additional discussion

During the optimization of the transfer hydrogenation step, the reduced Erlenmeyer-Plöchl azlactones (**3d**) were ring-opened by using alcohols (methanol or allyl alcohol) under the action of achiral tertiary amines (trimethylamine, *N*,*N*-diisopropylethylamine). These reactions gave ca. 5.5 : 1 diastereomeric ratio in favor of the *anti*-isomers of **4d**. The first set of optimized conditions is reported in Scheme S11.

**Scheme S11**. First set of optimized conditions for the transfer hydrogenation step and ensuing ring-opening with achiral tertiary amines.

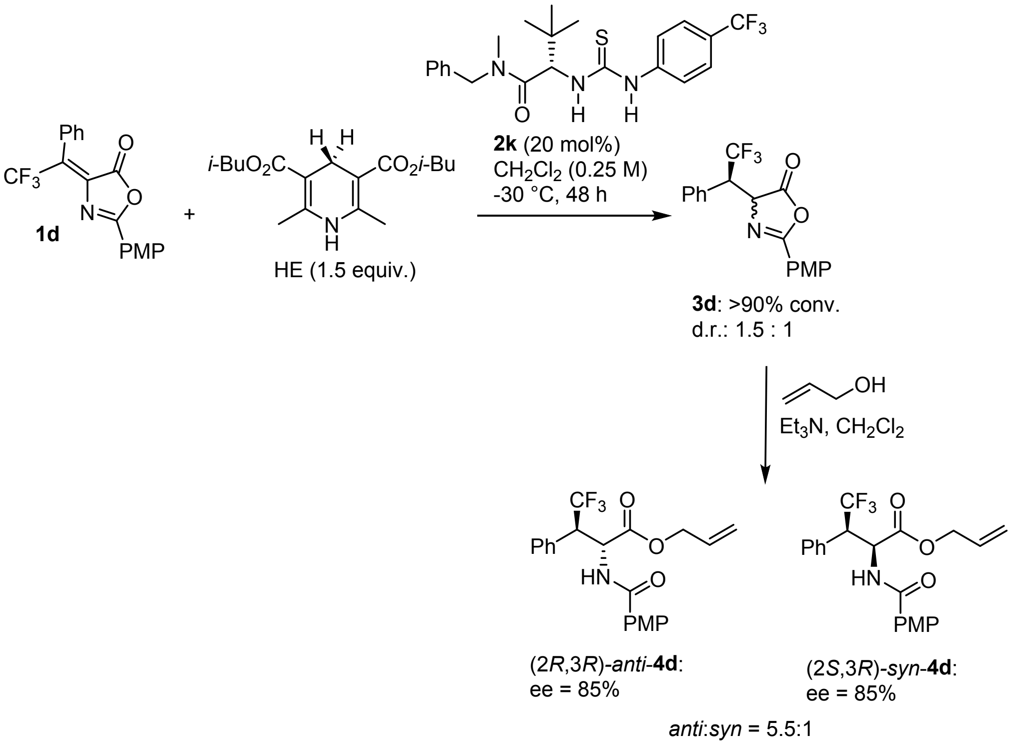


Since compound **3d** has ca. 1.5 : 1 diastereomeric ratio, it can be surmised that the ring-opening process is dynamic, and that substrate **3d** has a non-negligible tendency towards forming the *anti*-isomer **4d** in alcoholytic ring-opening promoted by tertiary amines. While a 5.5 : 1 substrate bias would make the development of an efficient stereodivergent process using homochiral tertiary amines[1] challenging, it was initially hypothesized that such bias

[1] (a) A. Berkessel, F. Cleemann, S. Mukherjee, T. N. Müller and J. Lex, *Angew. Chem. Int. Ed.*, 2005, **44**, 807. (b) A. Berkessel, S. Mukherjee, F. Cleemann, T. N. Müller and J. Lex, *Chem. Commun.*, 2005, 1898. (c) A. Berkessel, S. Mukherjee, T. N. Müller, F. Cleemann, K. Roland, M. Brandenburg, J.-N. Neudörfl and J. Lex, *Org. Biomol. Chem.*, 2006, **4**, 4319. (d) J. W. Lee, T. H. Ryu, J. S. Oh, H. Y. Bae and C. E. Song, *Chem. Commun.*, 2009, 7224. (e) J.-S. Oh, J.-W. Lee, T. H. Ryu, J. H. Lee and C. E. Song, *Org. Biomol. Chem.*, 2012, **10**, 1052. (f) S. Tallon, F. Manoni and S. J. Connon, *Angew. Chem. Int. Ed.*, 2015, **54**, 813.

may have been circumscribed to a tertiary amine promoted process. A variety of mechanistically distinct catalytic approaches (Lewis base, Lewis acid, Brønsted acid, enzyme catalysis) are available in the literature for the DKR of simple azlactones with different nucleophiles (alcohols, N-derivatives, thiols).[2] Approaches that were attempted in our case in the reaction with (*R*)-**3d** (ca. 85% ee) include: i) alcoholytic ring-opening using Lewis bases (DMAP in combination with different alcohols), ii) Brønsted acids (chiral phosphoric acids and various alcohols), iii) isothioureas (benzotetramisoles and benzylic and benzhydrylic alcohols); iv) thiolysis with thiophenol and *Cinchona* derivatives, v) the reaction with oxime. However, either these reactions showed a similarly high substrate bias (DMAP catalyzed reaction), or it proved not to be possible to achieve stereocontrol using chiral catalysts, or simply the reactions proceeded sluggishly (Brønsted acid alcoholytic reaction). Ultimately, the *Cinchona* alkaloid catalyzed alcoholysis reaction[1d-f] proved the most promising reaction platform for developing a stereodivergent, two-step process, despite its resemblance with the biased tertiary amine promoted reaction.

The following paragraphs will try to put numbers on the results that can be expected using chiral amine catalysts featuring different, arbitrarily chosen, selectivities in model ring-opening of simpler azlactones, in combination with such substrate bias towards the formation of *anti*-isomers. Admittedly resulting from a simplification of the catalysts-substrates interactions and ensuing transition states, which actually govern the selectivity of the overall process, these numbers are to be appreciated in a truly qualitative manner. They may serve to increase awareness on *i*) the challenging nature of the *syn*-selective amine catalyzed process, thus highlighting the performance of the new catalyst **dhQN-10**; *ii*) the mandatory requirement of high enantioselectivity in the first step, to reach good

[2] (a) J. Liang, J. C. Ruble and G. C. Fu, *J. Org. Chem.*, 1998, **63**, 3154. (b) A. J. Metrano and S. J. Miller, *J. Org. Chem.*, 2014, **79**, 1542. (c) X. Yang, G. Lu and V. B. Birman, *Org. Lett.*, 2010, **12**, 892. (d) A. L. Fuentes de Arriba, O. H. Rubio, L. Simón, V. Alcázar, L. M. Monleón, F. Sanz and J. R. Morán, *Tetrahedron: Asymm.*, 2017, **28**, 819. (e) Y.-C. Zhang, Q. Yang, X. Yang, Q.-N. Zhu and F. Shi, *Asian J. Org. Chem.*, 2016, **5**, 914. (f) K. Yu, X. Liu, X. Lin and X. Feng, *Chem. Commun.*, 2015, **51**, 14897. (g) D. A. Chaplin, M. E. Fox and S. H. B. Kroll, *Chem. Commun.*, 2014, **50**, 5858. (h) C. Palacio and S. J. Connon, *Eur. J. Org. Chem.*, 2013, 5398. (i) P. Liu, X. Yang, V. B. Birman and K. N. Houk, *Org. Lett.*, 2012, **14**, 3288. (j) Z. Rodríguez-Docampo, C. Quigley, S. Tallon and S. J. Connon, *J. Org. Chem.*, 2012, **77**, 2407. (k) C. Wang, H.-W. Luo and L.-Z. Gong, *Synlett*, 2011, 992. (l) G. Lu and V. B. Birman, *Org. Lett.*, 2011, **13**, 356. (m) S. A. Brown, M.-C. Parker and N. J. Turner, *Tetrahedron: Asymm.*, 2000, **11**, 1687. (n) N. J. Turner, J. R. Winterman, R. McCague, J. S. Parratt and S. J. C. Taylor, *Tetrahedron Lett.*, 1995, **36**, 1113. (o) J. Z. Crich, R. Brieva, P. Marquart, R.-L. Gu, S. Flemming and C. J. Sih, *J. Org. Chem.*, 1993, **58**, 3252. (p) R.-L. Gu, I.-S. Lee and C. J. Sih, *Tetrahedron Lett.*, 1992, **33**, 1953. (q) D. Seebach, G. Jaeschke, K. Gottwald, K. Matsuda, R. Formisano, D. A. Chaplin, M. Breuning and G. Bringmann, *Tetrahedron*, 1997, **53**, 7539. (r) K. Gottwald and D. Seebach, *Tetrahedron*, 1999, **55**, 723. (s) L. Xie, W. Hua, A. S. C. Chan and Y.-C. Leung, *Tetrahedron: Asymm.*, 1999, **10**, 4715. (t) C. Quigley, Z. Rodríguez-Docampo and S. J. Connon, *Chem. Commun.*, 2012, **48**, 1443.

diastereoselectivity in the overall transformation; *iii*) the uniqueness of the transition state leading to *syn*-**4d**.

The discussion can start by stressing that the application of chiral amine catalysts in the ring-opening reaction of (*R*)-**3d** implies a double asymmetric synthesis scenario,[3] in which two stereoinducing elements (the substrate **3d** with its C3 chirality center, and the chiral amine catalyst) control the configuration of the α-amino C2 center of product **4d**.

According to Masamune,[4] two situations are possible in double asymmetric synthesis: *matched*, in which the two stereo-inducing elements conspire in the same direction, and *mismatched*, in which the two elements head for opposite selectivities. Masamune proposed that double asymmetric induction can be analyzed in terms of the single asymmetric reactions of each stereoinducing element, by considering similar "model" reactions. Such analysis led to a rule of thumb (*multiplicity rule*) to predict qualitatively the stereochemical outcome of double asymmetric transformations: in the *matched* case, the selectivity can be expected to be the product of the selectivities displayed by the single model reactions, while, in the *mismatched* case, the selectivity can be predicted to be roughly the ratio between the two.

In the ring-opening under study, the first model reaction to be considered is the alcoholytic ring-opening of azlactone **3d** in the presence of an achiral tertiary amine catalyst, which has a 5.5 : 1 selectivity towards *anti*-**4d** (Scheme S12). That is, the 3*R* chirality center induces 2*R* configuration and, likewise, 3*S* chirality induces 2*S* selectivity. The second model reaction is the catalytic dynamic kinetic resolution of simpler azlactones by homochiral tertiary amine catalysts (Scheme S12). This reaction has ample literature precedents, with squaramide *Cinchona* alkaloid derivatives being the catalysts of choice[1d-f] furnishing selectivites up to 65:1.

[3] A. Horeau, H. B. Kagan and J. P. Vigneron, *Bull. Soc. Chim. Fr.*, 1968, 3795.
[4] S. Masamune, W. Choy, J. S. Petersen and L. R. Sita, *Angew. Chem. Int. Ed.*, 1985, **24**, 1.

**Scheme S12**. Model "single" reactions for the double asymmetric synthesis under investigation.

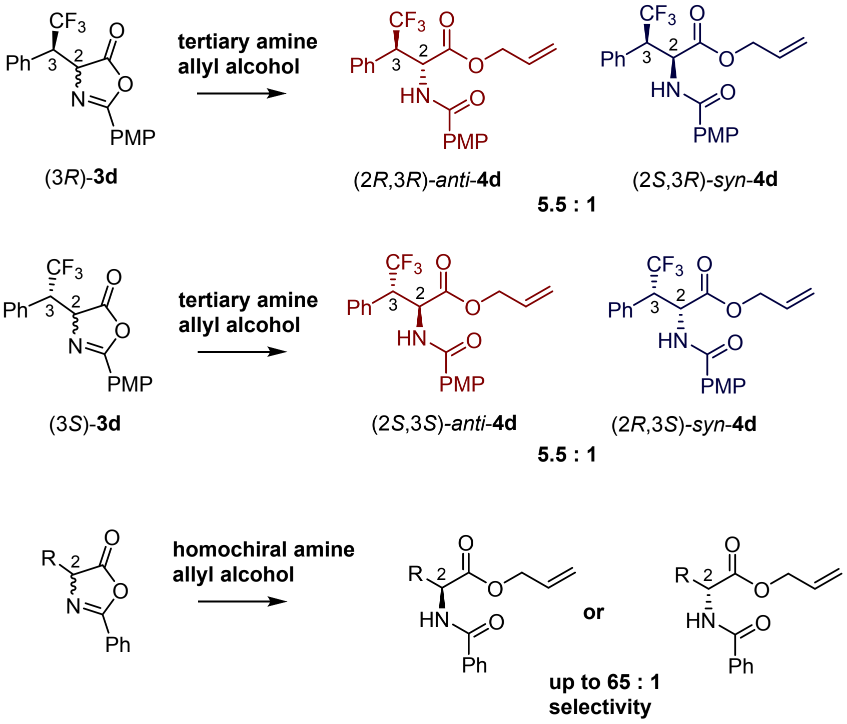


In the ring-opening of azlactone (3*R*)-**3d**, it can be intuitively recognized, and approximated using the multiplicity rule (Scheme S13), that even a homochiral amine catalyst displaying moderate "matched" 2*R*-selectivity in the model ring-opening of simpler azlactones (*e.g.* 3 : 1), would be sufficient for a highly (2*R*,3*R*)-*anti*-**4d** *vs* (2*S*,3*R*)-*syn*-**4d** selective reaction (3 x 5.5 → 16.5 : 1 d.r.). While, even a catalyst featuring high "mismatched" 2*S*-selectivity in the ring-opening (*e.g.* 20 : 1), would result in moderate (2*S*,3*R*)-*syn*-**4d** vs (2*R*,3*R*)-*anti*-**4d** selectivity (20 / 5.5 → 3.6 : 1).

**Scheme S13**. Stereoselectivities calculated by the multiplicity rule in the ring-opening of (*R*)-**3d** with amine catalysts featuring different "intrinsic" selectivites in the model ring opening of simpler azlactones.

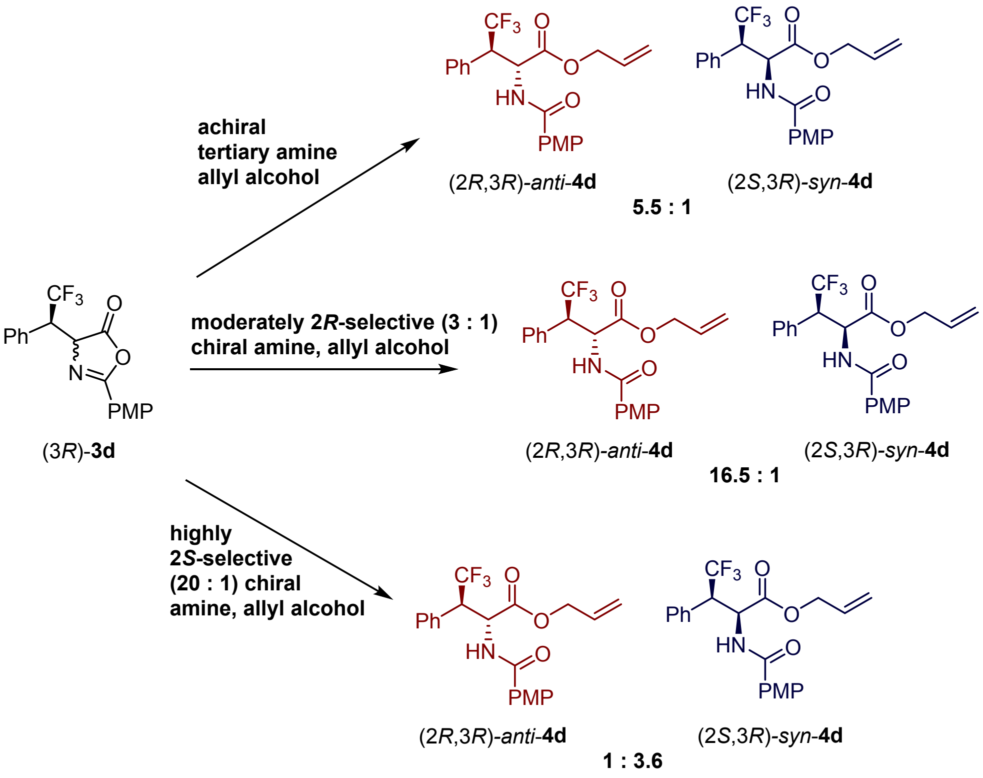


However, the multiplicity rule applies to enantiopure materials. Thus, in our case these numbers have to be adjusted taking into consideration that the reduced azlactone **3d** is not enantiopure, but is itself the result of the enantioselectivity offered by the catalyst **2** used in the first step.

In fact, for such type of two-step catalytic processes, the relationship between the selectivities of the two steps and the stereochemical results (diastereomeric ratios and enantiomeric excesses) can be obtained by the equations reported in Scheme S14, elaborated from the literature.[5] In this Scheme, ***s*1** is the selectivity offered by catalyst **A** in the first reaction (that is, the enantiomer ratio of **3d**), and ***s*2** and ***s*2'** are the two selectivities at the C2 chirality center (2*R vs* 2*S*) observed in the second step. It is worth to recall that the latter selectivities ***s*2** and ***s*2'** are the result of the double induction by substrate **3d** *and* catalyst **B**. Furthermore, since catalyst **B** is homochiral, its combination with (*R*)-**3d** and (*S*)-

[5] (a) A. M. Harned, *Tetrahedron*, 2008, **74**, 3797. (b) S. E. Baba, K. Sartor, J. Poulin and H. Kagan, *Bull. Soc. Chim. Fr.*, 1994, **131**, 525.

**3d** leads to different results, thus ***s*2 ≠ *s*2'**. Last, a 2*R*-selective process for the second step leads to an *anti*-isomer of **4d** when applied to (*R*)-**3d**, and to a *syn*-isomer of **4d** from (*S*)-**3d** (the opposite holds for a 2*S*-selective reaction).

**Scheme S14**. Reaction scheme and equations for calculating d.r. and ee from selectivity values.

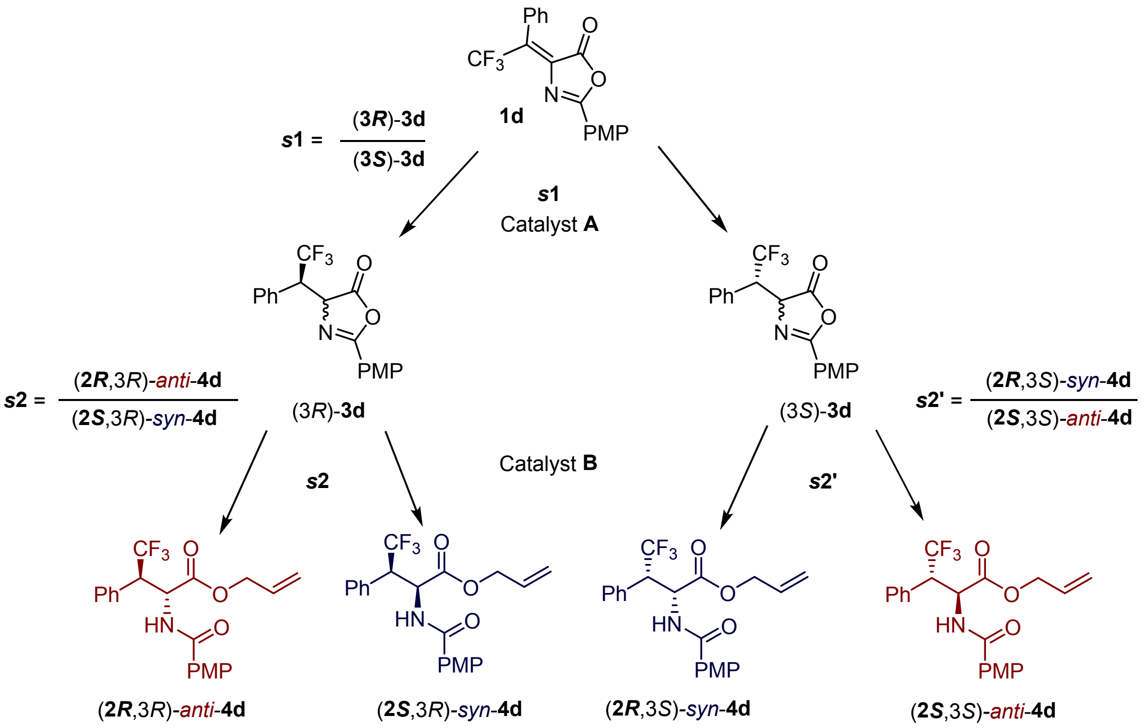


$$\text{d.r.} = \frac{anti}{syn} = \frac{(2R,3R)\text{-}anti\text{-}\mathbf{4d} + (2S,3S)\text{-}anti\text{-}\mathbf{4d}}{(2S,3R)\text{-}syn\text{-}\mathbf{4d} + (2R,3S)\text{-}syn\text{-}\mathbf{4d}} = \frac{\frac{s1 \times s2}{s2 + 1} + \frac{1}{s2' + 1}}{\frac{s2'}{s2' + 1} + \frac{s1}{s2 + 1}}$$

$$\text{e.e.}\ (anti) = \frac{(2R,3R)\text{-}anti\text{-}\mathbf{4d} - (2S,3S)\text{-}anti\text{-}\mathbf{4d}}{(2R,3R)\text{-}anti\text{-}\mathbf{4d} + (2S,3S)\text{-}anti\text{-}\mathbf{4d}} = \frac{\frac{s1 \times s2}{s2 + 1} - \frac{1}{s2' + 1}}{\frac{s1 \times s2}{s2 + 1} + \frac{1}{s2' + 1}}$$

$$\text{e.e.}\ (syn) = \frac{(2S,3R)\text{-}syn\text{-}\mathbf{4d} - (2R,3S)\text{-}syn\text{-}\mathbf{4d}}{(2S,3R)\text{-}syn\text{-}\mathbf{4d} + (2R,3S)\text{-}syn\text{-}\mathbf{4d}} = \frac{\frac{s2'}{s2' + 1} - \frac{s1}{s2 + 1}}{\frac{s2'}{s2' + 1} + \frac{s1}{s2 + 1}}$$

The application of these equations to the reaction under study, using Masamune's multiplicative rule, arbitrarily chosen selectivities for catalysts **A** and **B**, and a 5.5 : 1 *anti*-selectivity given by the 3C chirality center of **3d**, leads to the results reported in Table S2.

**Table S2**. Calculations of diastereo- and enantioselectivities in the two-step process via the equations shown in Scheme S14, using arbitrarily chosen/experimental selectivities for cat. **A** and **B**, 5.5 : 1 *anti*-selectivity given by substrate **3d**, Masamune multiplicative rule (matched case: ***s*2** or ***s*2'** = ***s***(cat. **B**) * 5.5 : 1; mismatched case: ***s*2** or ***s*2'** = ***s***(cat. **B**) / 5.5 : 1).

| Entry | ***s*1** (Cat. **A** selectivity) | ***s***(cat. B) "intrinsic" selectivity | ***s*2** (*R*)-**3d** → (**2*R***,3*R*)-***anti*-4d** / (**2*S***,3*R*)-***syn*-4d** | ***s*2'** (*S*)-**3d** → (**2*R***,3*S*)-***syn*-4d** / (**2*S***,3*S*)-***anti*-4d** | *anti*/*syn*-**4d** | ee % (*anti*) | ee % (*syn*) |
|---|---|---|---|---|---|---|---|
| 1 | 12.3:1 (85% ee for (3*R*)-**3d**) | 3:1-(**2*R***) | 16.5 : 1 (matched) | 1 : 1.8 (mismatched) | **11.9** : 1 | **89** | 31 |
| 2 | 12.3:1 (85% ee for (3*R*)-**3d**) | 20:1-(**2*S***) | 1 : 3.6 (mismatched) | 1 : 110 (matched) | 1 : **2.6** | 45 | **99** |
| 3 | 200:1 (99% ee for (3*R*)-**3d**) | 20:1-(**2*S***) | 1 : 3.6 (mismatched) | 1 : 110 (matched) | 1 : **3.6** | 96 | **100** |
| 4 | 21.2:1 (91% ee for (3*R*)-**3d**) | 67:1-(**2*S***) | 1 : 12.2 (mismatched) | 1 : 368 (matched) | 1 : **7.5** | 23 | **100** |

Entry 1 reports the case in which ***s*1** is 12.3:1 (that is, 85% ee as experimentally observed with catalyst **2k**), combined with a catalyst **B** having a moderate "intrinsic" 2*R*-selectivity (3:1) in the model ring-opening of simple azlactones (see Schemes S12 and S13). According to the multiplicative rule, ***s*2** (matched case between catalyst **B** and substrate (3*R*)-**3d**) is evaluated as 16.5:1, while ***s*2'** (in which catalyst **B** heads for (2*R*,3*S*)-*syn*-**4d** and the substrate for (2*S*,3*S*)-*anti*-**4d**, mismatched case) is evaluated to be 1:1.8. It can be noted that in the mismatched case a catalyst with moderate "intrinsic" selectivity is unable to invert the substrate induced *anti*-diastereoselectivity of the reaction. Overall, the application of the above equations results in a high 11.9:1 *anti*/*syn* selectivity in the two-step reaction, despite the low selectivity of catalyst **B** in the model ring-opening.

Conversely, entry 2 reports the case of a highly 2*S*-selective catalyst **B**. Due to the opposite bias of (*R*)-**3d**, ***s*2** is just 1:3.6, while catalyst **B** cooperates with (*S*)-**3d** towards giving the corresponding (2*S*,3*S*)-*anti*-**4d**, thus ***s*2'** reaches a 1:110 value. The overall result is a moderate 2.6:1 diastereomeric ratio for *syn*-**4d**, despite the high "intrinsic" 2*S*-selectivity of catalyst **B**.

In entry 3, the same catalyst **B** (*i.e.* same "intrinsic" 2*S*-selectivity) is considered, but this time with nearly enantiopure (*R*)-**3d** (***s*1** = 200:1, that is 99% ee for (*R*)-**3d**). In this case, the diastereoselectivity improved to a 3.6:1 value. This result can be understood considering that catalyst **B** converts (*R*)-**3d** to *syn*-**4d** but also, and even more efficiently, (*S*)-**3d** to *anti*-**4d**. Therefore, the less (*S*)-**3d** is present, the higher the *syn*/*anti* selectivity of **4d** if the same catalyst **B** is used.

In the last entry 4, the results in terms of diastereoselectivity and enantioselectivity of first step (***s*1** = 21.2:1 corresponding to 91% ee) and second step (*syn*/*anti* = 7.5:1) obtained under the fully optimized conditions for the *syn*-selective protocol, are reported. By applying the above equations, it results that the catalyst **B** able to impart the experimentally observed 7.5:1 *syn*/*anti* ratio under these conditions would be expected to have an "intrinsic" very high 68:1 2*S*-selectivity.

As an additional comment, the above equations predict also higher enantioenrichment of the major diastereomers of **4d**, compared to intermediate **3d**, according to the Horeau principle.[6] Such increase, which can be observed in entries 1,2 and 4 of Table S2, can be rationalized considering that a chiral catalyst **B** tends to convert (*R*)-**3d** and (*S*)-**3d** to opposite diastereoisomers (Scheme S14). In other words, catalyst **B** "removes" the minor enantiomer of **3d** by converting it into the minor diastereoisomer of **4d**. As a result, the unbalance between enantiomers is higher in the major diasteromeric product **4d** than in the intermediate **3d**.

As stressed by Masamune, the multiplicity rule holds true only if the transition states of the double and single ("model") asymmetric reactions are very similar. Does the reaction under study meet this requirement?

A first significant observation is given by the results obtained with the two catalysts reported in Scheme S15 for the ring-opening of (*R*)-**3d** and a model reaction. Their selectivities in the reaction with **3d** reflect very well their order of "intrinsic" 2*S*-selectivity, extracted from the reported values for the model reaction.[1d] Such results are representative of a large number of experiments performed during the optimization of the *syn*-selective process. In fact, it was found that several other catalysts (e.g. Takemoto catalyst, amino *Cinchona* derived ureas, etc.) which have been reported to be less stereoselective than the dimeric squaramide **QN-1**

[6] V. Rautenstrauch, *Bull. Soc. Chim. Fr.*, 1994, **131**, 515.

in the model reaction, were indeed less efficient in the *syn*-selective process too. Scheme S15 reports also a good agreement between observed and "predicted" (using the multiplicity rule and the equations shown in Scheme S14) *anti*/*syn* values.

**Scheme S15**. Comparison of **QN-1** and a thiourea catalyst: selectivities of the model reaction, and predicted/observed values for the reaction under study.

| | | |
|---|---|---|
| | 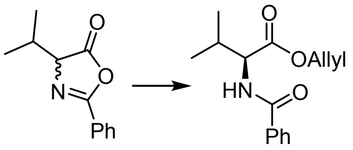  | 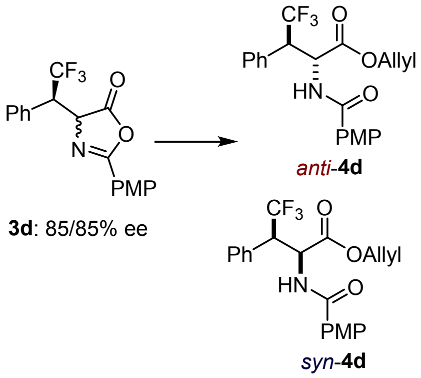  |
| 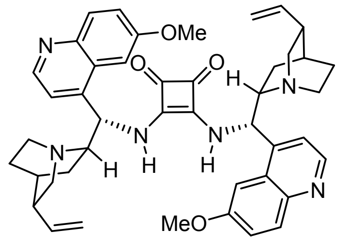  **QN-1** | **2S-selectivity: 27 : 1 (93% ee)** | **Predicted:** *anti/syn* = 1 : **3.3**<br>**Observed:** *anti*/*syn* = 1 : **2.3** |
| 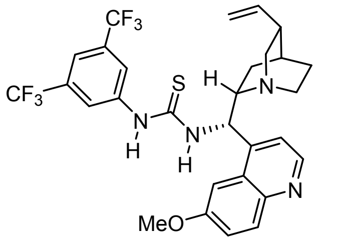  | **2S-selectivity: 7 : 1 (75% ee)** | **Predicted**: *anti/syn* = 1 : **1.1**<br>**Observed**: *anti/syn* = 1 : **1.0** |

Moving to inspect the squaramide series, Scheme S16 shows the selectivities displayed by catalysts **QN-1**, **dhQN-2**, **dhQN-3** and **dhQN-10** in the model and the double asymmetric reactions. A minor discrepancy is observed with catalysts **QN-1** and **dh-QN2**, while the selectivity order is followed very well by moving from the more 2*S*-selective catalyst **dhQN-2** to its less 2*S*-selective diastereoisomer **dhQN-3**.[7] The ammonia derived optimal catalyst **dhQN-10** shows instead a dramatically distinct behavior. It clearly stands out as the most *syn*-selective catalyst for the ring opening of (*R*)-**3d**, while being only moderately 2*S*-selective, at least compared to **QN-1** and **dhQN-2**, in the model reaction.

[7] Similar conclusions can be drawn for quindine-derived catalysts used in the *anti*-selective reaction.

**Scheme S16**. Comparison between a model and the double asymmetric reaction for catalysts **QN-1**, **dhQN-2**, **dhQN-3** and **dhQN-10**.

| | | |
|---|---|---|
| | 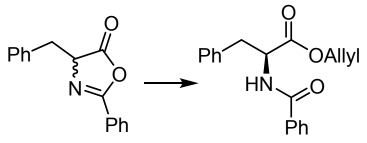 | 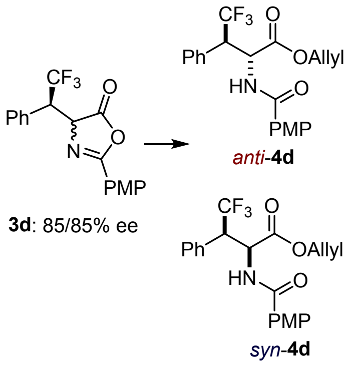 |
| 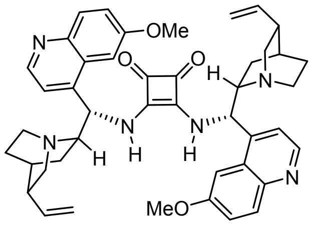<br>**QN-1** | **2S-selectivity: 24 : 1 (92% ee)** | **Predicted:** *anti/syn* = 1 : **3.1**<br>**Observed:** *anti/syn* = 1 : **2.3** |
| 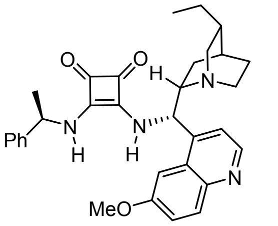<br>**dhQN-2** | **2S-selectivity: 19 : 1 (90% ee)** | **Predicted**: *anti/syn* = 1 : **2.6**<br>**Observed**: *anti/syn* = 1 : **2.4** |
| 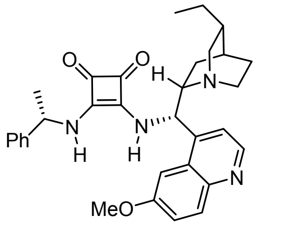<br>**dhQN-3** | **2S-selectivity: 11.5 : 1 (84% ee)** | **Predicted**: *anti/syn* = 1 : **1.7**<br>**Observed**: *anti/syn* = 1 : **1.5** |
| 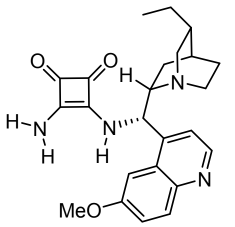<br>**dhQN-10** | **2S-selectivity: 13.4 : 1 (87% ee)** | **Predicted**: *anti/syn* = 1 : **1.9**<br>**Observed**: *anti/syn* = 1 : **5.9** |

Another interesting observation is that, while such ammonia-derived catalysts are well versed for the *syn*-selective reaction, they are not apt for the *anti*-selective process. For example (Scheme S17), **dhQD-10** gives an *anti*/*syn*-**4d** selectivity comparable to the one

offered by the substrate itself. Such result is not due to the fact that **dhQD-10** is pseudo-enantiomeric (and not enantiomeric) to **dhQN-10**, since experiments in the development of the *syn*-selective process with *ent*-**3d** confirmed ammonia derived **dhQD-10** as the *syn*-selective catalyst of choice.

**Scheme S17**. Ring-opening of (*R*)-**3d** catalysed by **dhQD-10**.

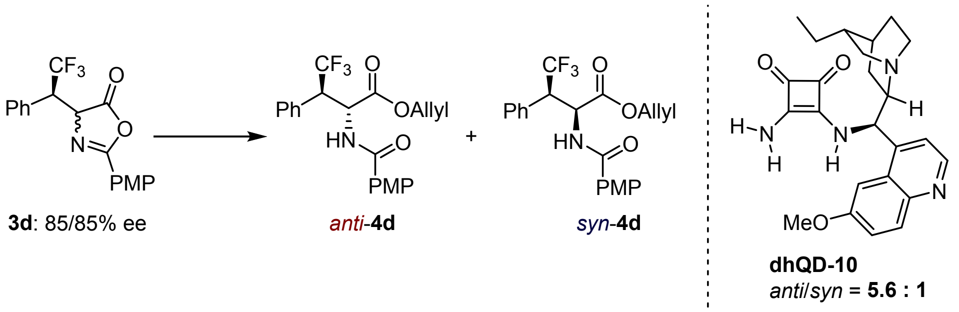


Summarizing the observations and the deductions of this section:

-The alcoholytic ring-opening of β-trifluoromethyl azlactone (*R*)-**3d** promoted by tertiary amines was approached as a double asymmetric synthesis case, wherein Masamune's multiplicity rules apply.

-In this scenario, due to a relatively high substrate bias towards *anti*-**4d** for tertiary amine promoted alcoholysis, it is relatively straightforward to achieve high *anti*-**4d** selectivities using chiral tertiary amines having 2*R*-selectivity in the ring-opening of simple azlactones.

-On the contrary, two requirements have to be met to realize a moderate to high *syn*-**4d** selective process:

i) **3d** must be highly enantioenriched, that is, a highly enantioselective transfer hydrogenation of **1d** must be in place.

ii) a very high 2*S*-selective catalyst in the ring-opening of simple azlactones must be used. In fact, experimentally, the 2S selectivity order of many catalysts tested is reflected in the *syn*/*anti* ratio of **4d**, which in some cases can be even predicted approximately by the multiplicity rule, adjusted by including the e.r. of **3d** in the calculations.

*however*

The influence of the steric hindrance at the squaramide portion of the catalyst on *syn*/*anti* selectivity (see main text) appears a distinctive feature of the ring-opening process towards *syn*-**4d**. In this process, ammonia-derived catalyst **dhQN-10** clearly stands out as being the most selective catalyst, while being only moderately 2*S*-selective with simple azlactones. Therefore, the transition state leading to *syn*-**4d** is probably unique and geometrically different from the ring-opening of simpler azlactones, and also from the transition state leading to the *anti*-**4d** product. Alternatively, stabilization of such transition state requires unique catalyst features.

Overall, the effectiveness of catalyst **dhQN-10** in “forcing” the ring-opening towards the mismatched *syn*-**4d** is remarkable. The catalyst appears specifically purposed for this reaction.

## One pot procedure – control results

Initial attempts to implement a one-pot procedure by simply adding additional $CH_2Cl_2$, allyl alcohol and the appropriate squaramide catalyst, and placing the mixture at 0 °C or RT once the transfer hydrogenation was judged complete were not successful. The ring-opening step proceeded sluggishly, reaching only a moderate conversion after prolonged reaction time. Diastereoselectivity was reduced too. In order to understand which element interfered with the catalytic ring-opening process, the control experiments reported in Scheme S18 were performed. These experiments were performed in parallel with the optimization of the reactions (i.e. catalysts are not optimal ones). It was found that while the thiourea catalyst **2k** and the oxidized pyridine were innocuous, the HE - which was used in excess (1.5 equiv.) in the transfer hydrogenation step - inhibited the *Cinchona* squaramide catalyst promoted process.

**Scheme S18**. Control experiments.

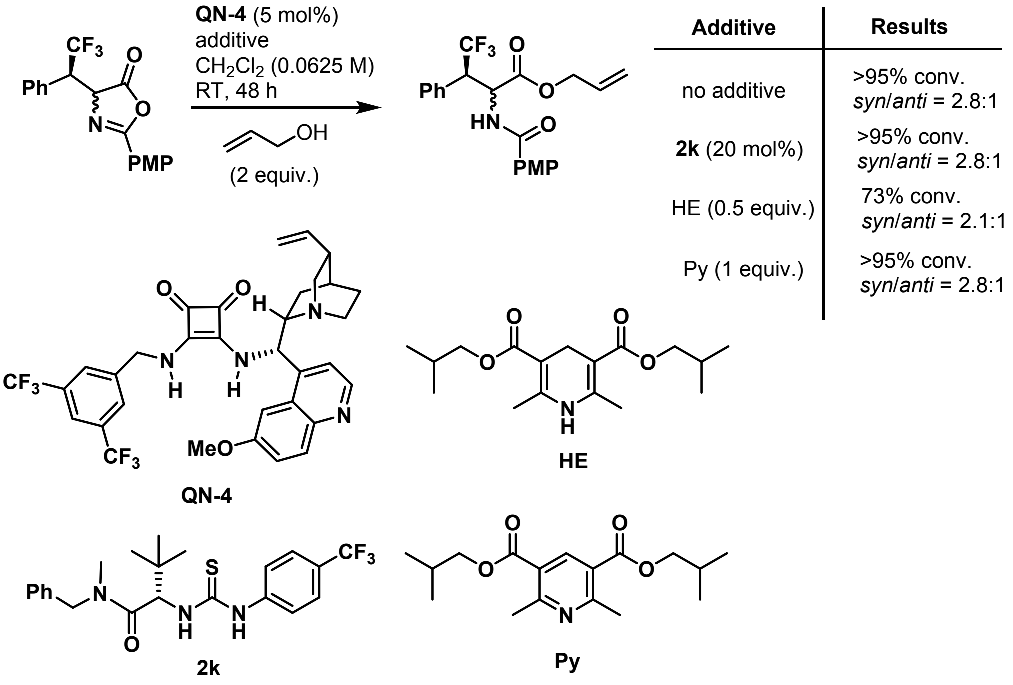


| Additive | Results |
|---|---|
| no additive | >95% conv. *syn*/*anti* = 2.8:1 |
| **2k** (20 mol%) | >95% conv. *syn*/*anti* = 2.8:1 |
| HE (0.5 equiv.) | 73% conv. *syn*/*anti* = 2.1:1 |
| Py (1 equiv.) | >95% conv. *syn*/*anti* = 2.8:1 |

Ultimately, it was sufficient to reduce the amount of HE to 1.1 equiv. (instead of 1.5 equiv.) in the transfer hydrogenation step, to avoid its interference in the catalytic ring-opening process thus permitting the development of an efficient one pot protocol.

# Olefin geometry and enantioinduction in the transfer hydrogenation reaction

According to the simplified transition state model for the transfer hydrogenation reaction, the geometry of the olefin dictates the configuration of the C3 chirality centre of product **3** which is obtained in the reaction. *Z*-configured Erlenmeyer-Plöchl substrates **1**, obtained in pure *Z*-form by crystallization, are used in this study, leading to (3*R*)-**3** as major products when L-*tert*-leucine catalysts **2** are employed (Scheme S19).

**Scheme S19**. Transition state model for the transfer hydrogenation reaction.

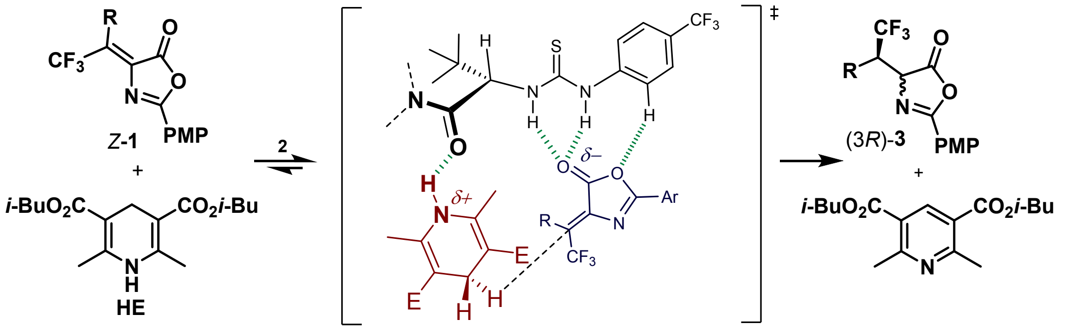


In order to check the correctness of this assumption, as well as possible match/mismatch effects which could simplify the preparation of the substrates (*i.e.* if the minor olefin isomer would react very slowly), the transfer hydrogenation reaction of substrate **1h** was studied in more detail. As shown in Scheme S20, the reaction with pure *Z*-**1h**, under the optimized conditions, leads to the corresponding azlactone (3*R*)-**3h** with 90% ee (measured on the corresponding ring-opened product **4h**). In contrast, using substrate **1h** as a *Z*/*E* 3.1 : 1 mixture (obtained from the mother liquors of the crystallization performed to obtain *Z*-**1h**) product (3*R*)-**3h** is obtained with only 42% ee. Furthermore, a sample of this reaction after 5 hours (73% conversion) showed that the *Z*/*E* ratio of the remaining olefin slightly increased to 4.0 : 1. Thus, the minor *E*-**1h** isomer reacted slightly faster than the major *Z*-**1h**.

**Scheme S20**. Control experiments with substrate **1h**.

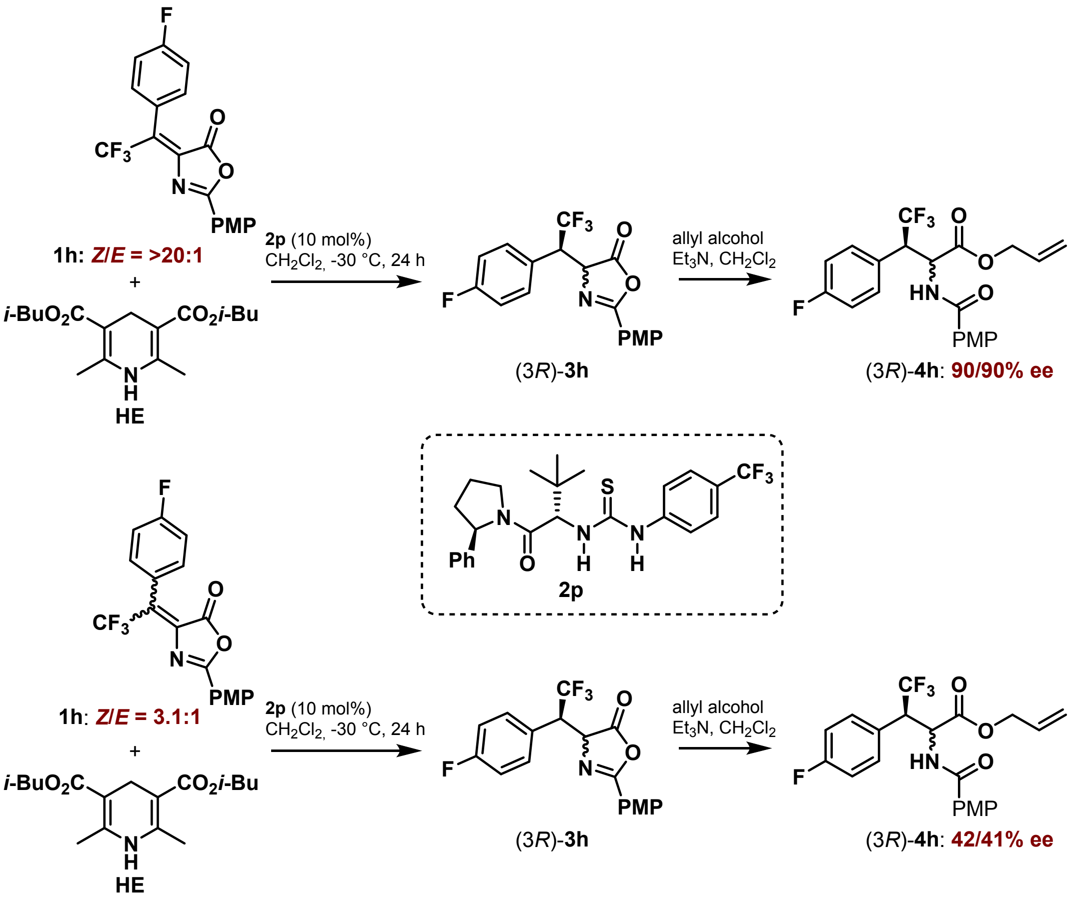


Thus, it can be concluded that to reach high enantioselectivity in the transfer hydrogenation step, it is necessary to use isomerically pure olefins **1**, since both *Z*-**1** and *E*-**1** take part in the reaction with comparable rates, but giving products **3** with opposite configuration at the C3 chirality centre. From the observed enantiomeric excesses of the two reactions, it can be determined that the (3*S*)-selectivity in the transfer hydrogenation reaction of substrate *E*-**1h** with catalyst **2p** is very high, thus comparable with the (3*R*)-selectivity of the reaction with substrate *Z*-**1h**.

## Ring-opening step – tentative reaction models

An attempt of rationalisation of the results obtained during the ring-opening step of azlactones **3** begins with considering the generally accepted pathway followed by alcoholytic dynamic kinetic resolution of simpler azlactones promoted by bifunctional catalysts, in which fast racemisation of the starting azlactone by a base[8] is combined with a catalytic resolution step. In an enlightening computational contribution, Berkessel and Etzenbach-Effers showed that attack of the alcohol to the azlactone carbonyl is the rate- and selectivity-determining step of the process.[9] As shown in Scheme S21 for the simplified model investigated computationally, (1*R*,2*R*)-diamine derived bifunctional catalysts promote selectively the ring-opening of the (*R*)-azlactone. Key interactions (not shown in the Scheme) are stabilisation of the negative charge on the oxygen atom by the thiourea group, and of the positively charged attacking alcohol by coordination of its proton with the tertiary amine portion. Although un-consequential for the configuration of the final product, it was shown that attack occurs preferentially to the *Re*-face of the lactone, that is, opposite to the C4-substituent, for steric reasons, which hinder considerably attack to the *Si*-face.

**Scheme S21**. Ring-opening of simple azlactones: computationally investigated simplified reaction.

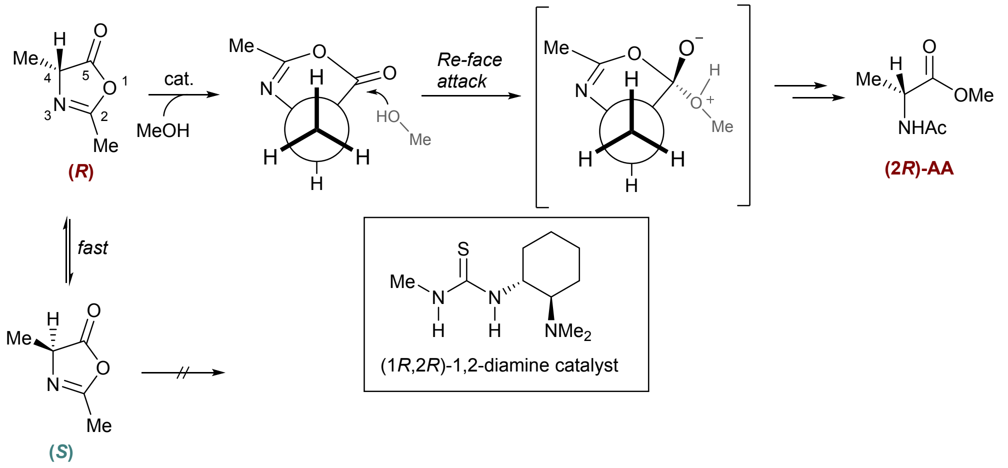


In the diastereoselective ring-opening of product (3*R*)-**3d**, both the chirality centre and the bifunctional catalyst exert an effect on the configuration at C2 of the ring-opened product **4d**. Also in this case, epimerization can be considered faster than ring-opening, since the

[8] P. P. de Castro, G. M. F. Batista, H. F. dos Santos and G. W. Amarante, *ACS Omega*, 2018, **3**, 3507.
[9] A. Berkessel and K. Etzenbach-Effers, Computational Studies of Organocatalytic Processes Based on Hydrogen Bonding, In *Hydrogen Bonding in Organic Synthesis*, Ed. P. M. Pihko, Ch. 3, pp. 15-42, Wiley-VCH, Weinheim, 2009.

diastereomeric ratio in the azlactone **3d** was found to be nearly constant during the reaction course. Furthermore, the two diastereoisomers have similar stability, being present in nearly equimolar amounts. Considering as a first approximation that attack of the alcohol is rate- and stereo- determining also in this case, selectivity is the result of a more favourable attack of the alcohol to one of the two diastereoisomers *anti*-**3d** or *syn*-**3d**. In line with the computational results of Berkessel and Etzenbach-Effers, it can be surmised that attack occurs on the face opposite to the chiral substituent of the azlactone, according to the reaction models shown in Scheme S22. In these models, an *anti*-arrangement between the trifluoromethyl group and the attacked carbonyl has been considered as most likely. Such conformation minimizes the overall dipole and repulsive electronic interactions[10] in the transitions state/intermediate. Experimentally, bifunctional catalysts derived from quinidine and dihydroquinidine (having a “local” 1*R*,2*R* configuration at the 1,2-diamine) delivers the (2*R*,3*R*)-**3d** isomer as major product, while quinine and dihydroquinine derivatives (having a “local” 1*S*,2*S* configuration at the 1,2-diamine) favour the formation of the (2*S*,3*R*)-**3d** isomer. While these results can be interpreted extrapolating Berkessel’s work into the models shown, with the conceivable inclusion of similar stabilizing interactions between the bifunctional catalyst portions and the lactone carbonyl oxygen, and the attacking alcohol, it is more difficult to devise the effect of the chirality centre on the stability of these transitions states/intermediates, and thus the selectivity observed towards (2*R*,3*R*)-*anti*-**3d** in the reactions catalysed by achiral amines. At a purely speculative level, it seems that attack to the lactone of *syn*-**3d** is hindered by the phenyl group, which instead does not hinder attack to the lactone of the *anti*-**3d**. Such hindrance present in the *syn*-pathway might justify the requirement of catalysts featuring a steric hindrance as low as possible at their squaramide portion, to channel the reaction preferentially through this pathway.

[10] T. Katagiri, S. Yamaji, M. Handa, M. Irie and K. Uneyama, *Chem. Commun.*, 2001, 2054.

**Scheme S22**. Reaction models for the ring opening of azlactones *anti*-**3d** and *syn*-**3d**.

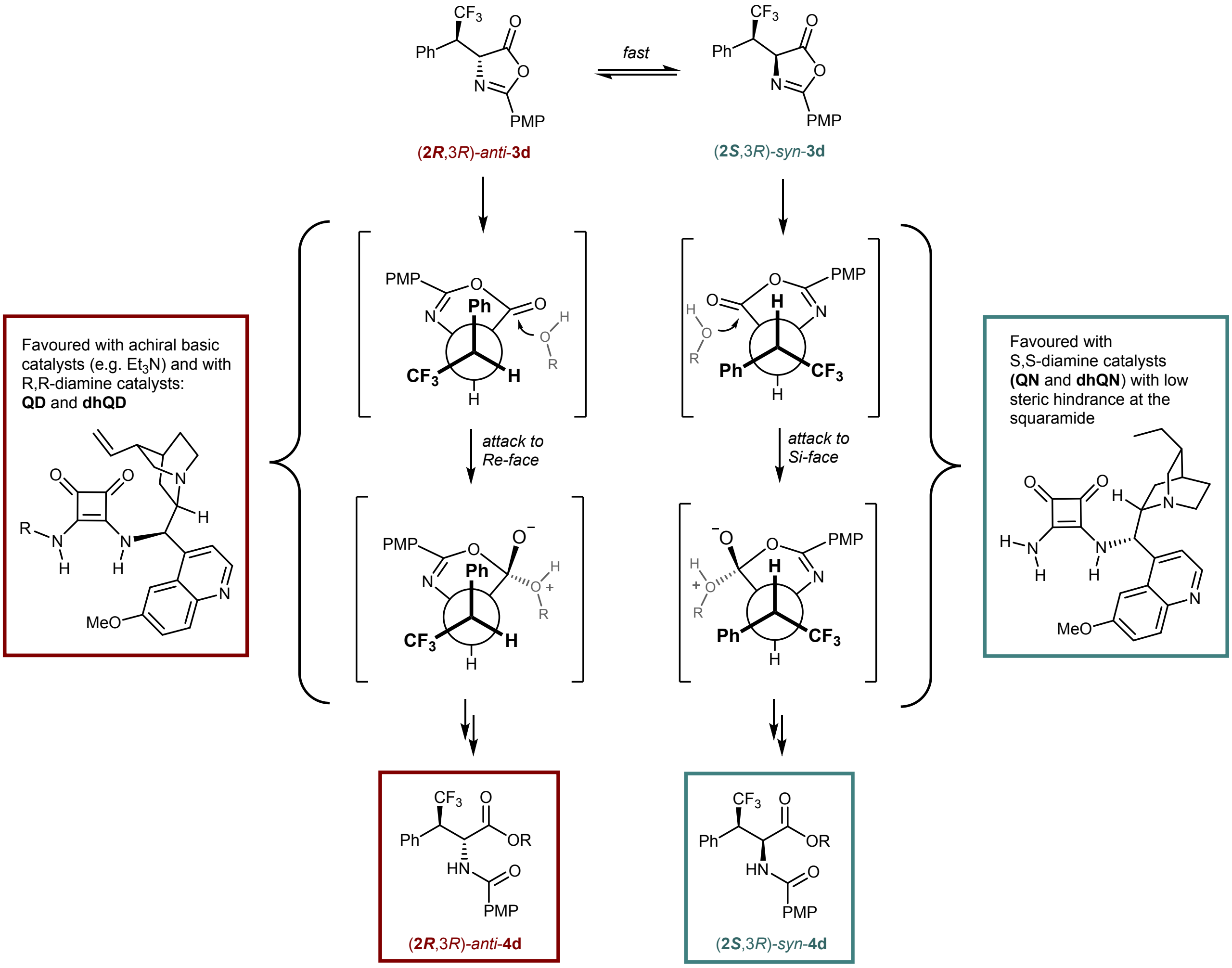

# Limitations of the methodology

During the study of the scope of the reaction, the following limitations became apparent.

i) The one-pot method used to prepare the substrates from α,α,α-trifluoroketones and *N*-4-methoxybenxoyl glycine was not successful with *ortho*-substituted acetophenones:

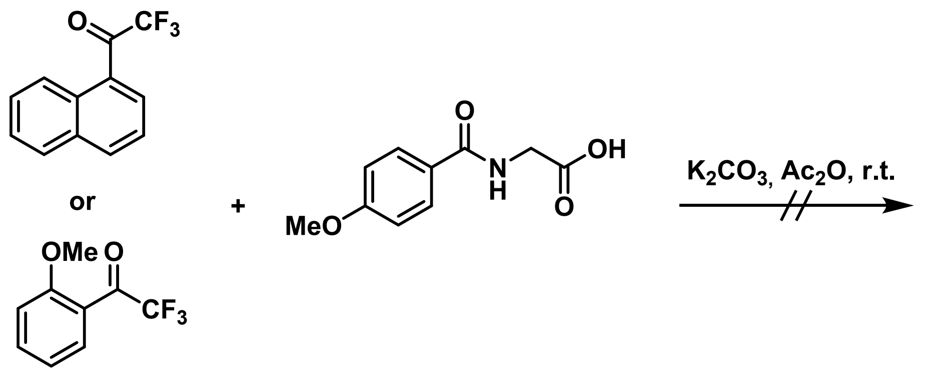


ii) A non-fluorinated derivative was found to be poorly reactive in the transfer hydrogenation with Hantzsch esters catalyzed by a representative thiourea (Schreiner's thiourea).

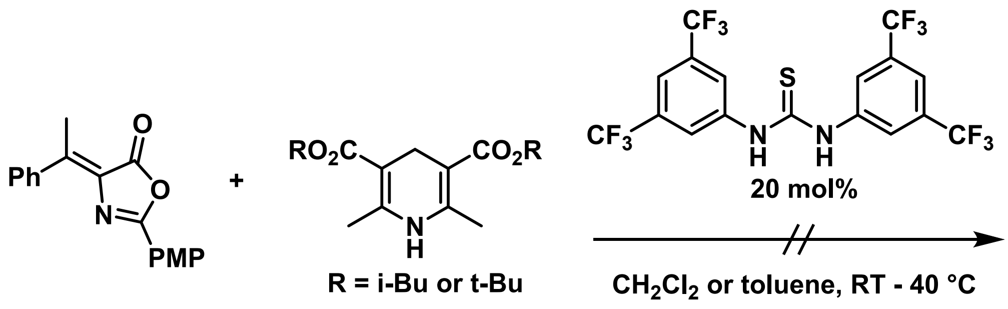

iii) A difluoromethyl derivative was found instead to be sufficiently reactive, leading to a clean and fast reaction at 0 °C. Unfortunately, enantioselectivity of the transfer hydrogenation step was found to be 40% using optimal catalyst **2p**, and could not be improved by using related catalysts.

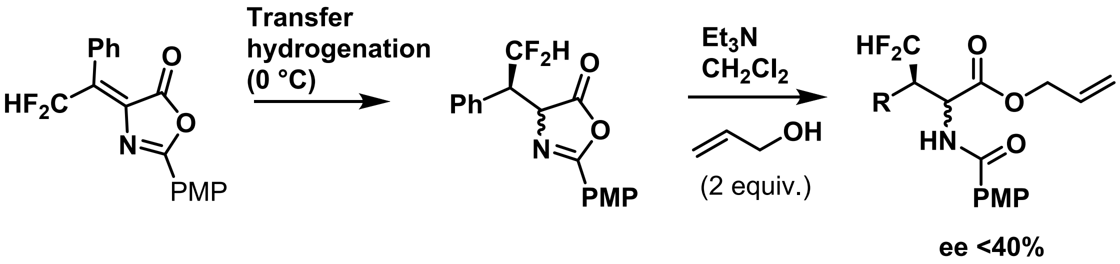


iv) Secondary alcohols cannot be used in the ring-opening step, as their reactivity is not sufficient.

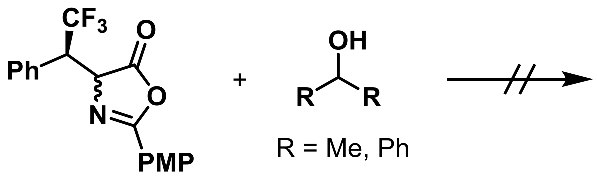

# Experimental section

## General Methods.

The $^{1}H$, $^{13}C$ and $^{19}F$ NMR spectra were recorded on Varian Inova 300, Mercury 400, or Inova 600 spectrometer. Chemical shifts (δ) are reported in ppm relative to residual solvents signals for $^{1}H$ and $^{13}C$ NMR,[11] and using $CF_3C_6H_5$ as external reference calibrated at -63.72 ppm for $^{19}F$ NMR. $^{13}C$ NMR spectra were acquired with $^{1}H$ broad-band decoupled mode. Chromatographic separations were carried out using 70 – 230 mesh silica. High Resolution Mass Spectra (HRMS) were recorded on a Waters Xevo G2-XS QTof spectrometer. Optical rotations were measured on a Perkin-Elmer 241 polarimeter provided with a sodium lamp and are reported as follows: $[\alpha]_{\lambda}^{T\,°C}$ (c = g/100 mL in solvent). The enantiomeric excess (ee) of the products was determined by chiral stationary phase HPLC (Daicel Chiralpak AD-H, IA, or Chiralcel OD-H), using a UV detector operating at 254 nm.

## Materials:

The two *α*,*α*,*α*-trifluoroacetophenones (2,2,2-trifluoro-1-(4-methoxyphenyl)ethan-1-one and 1-(4-chlorophenyl)-2,2,2-trifluoroethan-1-one) used for the synthesis of azlactones **1f,i** were prepared by reaction of the corresponding Grignard reagents with the trifluoromethyl Weinreb amide (2,2,2-trifluoro-*N*-methoxy-*N*-methylacetamide).[12] The trifluoromethylketone used for the synthesis of azlactone **1k** (2,2,2-trifluoro-1-(thiophen-2-yl)ethan-1-one) was prepared by reaction of commercial 2-thienyl lithium with ethyl 2,2,2-trifluoroacetate.[13] The 1-tosyl 3-trifluoroacetyl indole used for the synthesis of **1l** was prepared by trifluoroacetylation of indole with trifluoroacetic anhydride, followed by *N*-tosylation, as previously described by us.[14]

*N*-(4-Methoxybenzoyl) glycine was prepared via a two-step *N*-acylation – ester hydrolysis from glycine methyl ester and 4-methoxybenzoyl chloride, according to the literature procedure used for other amino acids.[15] This two-step procedure gave better results than

---

[11] H. E. Gottlieb, V. Kotlyar and A. Nudelman, *J. Org. Chem.*, 1997, **62**, 7512.
[12] C. J. Jiang, C. L. Cheng and S. F. Yuan, *Asian J. Chem.*, 2015, **27**, 2406.
[13] X. Creary, *J. Org. Chem.*, 1987, **52**, 5026.
[14] E. Martinelli, A. C. Vicini, M. Mancinelli, A. Mazzanti, P. Zani, L. Bernardi and M. Fochi, *Chem. Commun.*, 2015, **51**, 658.
[15] J. C. Ruble and G. C. Fu, *J. Am. Chem. Soc.*, 1998, **120**, 11532.

the direct amidation of the amino acid with the 4-methoxybenzoyl chloride in the presence of aqueous NaOH.

iso-Butyl Hantzsch ester HE was obtained as pale yellow crystals in 35-53% yield by applying a literature Hantzsch protocol[16] involving the reaction between iso-butyl acetoacetate, paraformaldehyde and ammonium acetate, followed by crystallization from MeOH [$^1$H NMR (300 MHz, $CDCl_3$) δ = 5.17 (s, 1H), 3.88 (d, *J* = 6.4 Hz, 4H), 3.31 (s, 2H), 2.19 (s, 6H), 1.96 (dq, *J* = 13.3, 6.6 Hz, 2H), 0.95 (d, *J* = 6.7 Hz, 12H)].

(*R*)-2-Phenyl pyrrolidine and (*S*)-2-phenyl pyrroldine, HCl salts, used for catalysts **2p** and *ent*-**2p** were prepared by (-)- or (+)-sparteine mediated enantioselective lithiation of *N*-Boc pyrrolidine, transmetallation with $ZnCl_2$ and Negishi coupling,[17] and Boc deprotection with HCl in dioxane. The *t*-leucine amide hydrochloride salts used for catalysts **2k,m** and *ent*-**2p** were prepared from the corresponding amine and *N*-Boc *tert*-leucine, following literature procedures (HBTU amide coupling in $CH_2Cl_2$ for **2k**,[18] or EDCI – HOBT amide coupling in DMF for **2p**,[19] followed by deprotection with HCl in dioxane).

9-Amino-9-deoxy *epi*-dihydroquinine used to prepare **dhQN** catalysts, and 9-amino-9-deoxy *epi*-dihydroquinidine used to prepare **dhQD** catalysts, were prepared[20] and purified as tri-hydrochloride salts[21] following literature procedures. The corresponding squaramide catalysts **dhQN-2,10** and **dhQD-2,10** were prepared by applying a typical literature procedure,[22] as outlined below.

---

[16] M.-H. Larraufie, R. Pellet, L. Fensterbank, J.-P. Goddard, E. Lacôte, M. Malacria and C. Ollivier, *Angew. Chem. Int. Ed.*, 2011, **50**, 4463.
[17] K. R. Campos, A. Klapars, J. H. Waldman, P. G. Dormer and C. Y. Chen, *J. Am. Chem. Soc.*, 2006, **128**, 3538.
[18] S. E. Reisman, A. G. Doyle and E. N. Jacobsen, *J. Am. Chem. Soc.*, 2008, **130**, 7198.
[19] R. R. Knowles, S. Lin and E. N. Jacobsen, *J. Am. Chem. Soc.*, 2010, **132**, 5030.
[20] Y. Wang, K. L. Milikiewicz, M. L. Kaufman, L. He, N. G. Landmesser, D. V. Levy, S. P. Allwein, M. A. Christie, M. A. Olsen, C. J. Nelville and K. Muthukumaran, *Org. Process Res. Dev.*, 2017, **21**, 408.
[21] C. Cassani, R. Martín-Rapún, E. Arceo, F. Bravo and P. Melchiorre, *Nat. Protoc.*, 2013, **8**, 325.
[22] V. H. Rawal, J. P. Malerich and H. Hagihara, *J. Am. Chem. Soc.*, 2008, **130**, 14416.

## Synthesis and characterization of Erlenmeyer-Plöchl azlactone derivatives 1.[23]

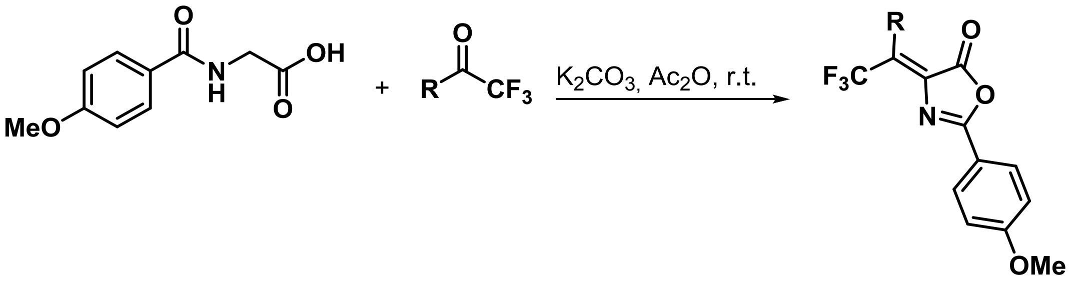

**General procedure**: To a suspension of *N*-(4-methoxybenzoyl)glycine (1 equiv., 3 mmol) in acetic anhydride (5.5 mL), $K_2CO_3$ (0.5 equiv., 1.5 mmol) was added, followed by the appropriate 2,2,2-trifluoroacetophenone (1 equiv., 3 mmol). The mixture was stirred overnight at room temperature, then 30 mL of water were added and the mixture stirred for additional 24 h. The precipitate was filtered, dried, suspended in $CH_2Cl_2$ and filtered through a short silica plug eluted with $CH_2Cl_2$. The resulting solid was recrystallized from hexanes to afford the desired arylidene azlactones **1** as single diastereoisomers. The olefinic E/Z configuration of an azlactone closely related to **1j** has been previously determined as Z.[23] The configuration of all azlactones **1** has been assigned as *Z* (**1k** features E configuration due to CIP priority rules) by analogy.

**(*Z*)-2-(4-methoxyphenyl)-4-(2,2,2-trifluoro-1-phenylethylidene)oxazol-5(4*H*)-one (1d).**

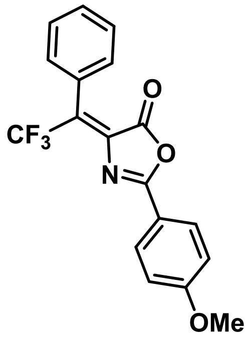

From 2,2,2-trifluoroacetophenone. Isolated as single diastereoisomer in 50% yield, as pale yellow crystals. **$^1$H NMR** (400 MHz, $CDCl_3$) δ = 8.15 – 8.09 (m, 2H), 7.52 – 7.43 (m, 3H), 7.38 – 7.33 (m, 2H), 7.04 – 6.97 (m, 2H), 3.89 (s, 3H). **$^{19}$F NMR** (376 MHz, $CDCl_3$) δ = -59.33 (s, 3F). **$^{13}$C NMR** (100 MHz, $CDCl_3$) δ = 165.2 (q, *J* = 2.2 Hz), 161.8, 163.7, 137.5 (q, *J* = 2.0 Hz), 132.1 (q, *J* = 31.0 Hz), 131.3, 129.9, 129.4, 129.2 (q, *J* = 1.6 Hz), 128.4, 122.3 (q, *J* = 276.3 Hz), 116.7, 114.6, 55.6. **HRMS**: calculated for $C_{18}H_{13}F_3NO_3$ [M + H$^+$] = 348.0848; found: 348.0848.

[23] Procedure adapted from: A. Alimardanov, A. Nikitenko, T. J. Connolly, G. Feigelson, A. W. Chan, Z. Ding, M. Ghosh, X. Shi, J. Ren, E. Hansen, R. Farr, M. MacEwan, S. Tadayon, D. M. Springer, A. F. Kreft, D. M. Ho and J. R. Potoski, *Org. Process Res. Dev.*, 2009, **13**, 1161.

**(*Z*)-4-(1-(4-bromophenyl)-2,2,2-trifluoroethylidene)-2-(4-methoxyphenyl)oxazol-5(4*H*)-one (1e).**

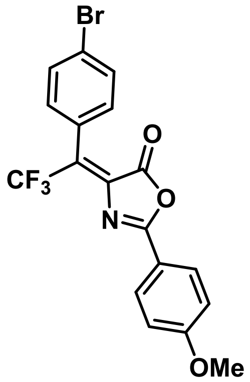

From 4'-bromo-2,2,2-trifluoroacetophenone. Isolated as single diastereoisomer in 48% yield, as orange crystals. **$^1$H NMR** (400 MHz, $CDCl_3$) δ = 8.16 – 8.11 (m, 2H), 7.63 – 7.57 (m, 2H), 7.25 – 7.21 (m, 2H), 7.05 – 6.99 (m, 2H), 3.92 (s, 3H). **$^{19}$F NMR** (376 MHz, $CDCl_3$) δ = -59.31 (s, 3F). **$^{13}$C NMR** (101 MHz, $CDCl_3$) δ = 165.7 (q, $J$ = 2.2 Hz), 165.2, 163.9, 137.9 (q, $J$ = 2.1 Hz), 131.7, 131.6, 131.2, 130.8 (q, $J$ = 32.6 Hz), 128.2 (q, $J$ = 1.6 Hz), 124.8, 122.2 (q, $J$ = 276.7 Hz), 116.6, 114.9, 55.8. **HRMS**: calculated for $C_{18}H_{12}BrF_3NO_3$ [M + H$^+$] = 425.9953; found: 425.9946.

**(*Z*)-4-(1-(4-chlorophenyl)-2,2,2-trifluoroethylidene)-2-(4-methoxyphenyl)oxazol-5(4*H*)-one (1f).**

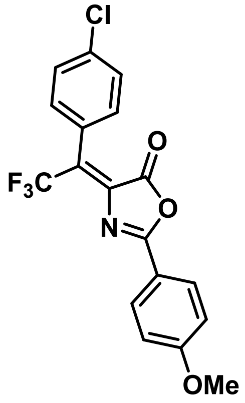

From 4'-chloro-2,2,2-trifluoroacetophenone. Isolated as single diastereoisomer in 53% yield, as orange crystals. **$^1$H NMR** (400 MHz $CDCl_3$) δ = 8.15-8.10 (m, 2H), 7.47-7.41 (m, 2H), 7.33-7.27 (m, 2H), 7.05-6.99 (m, 2H), 3.91 (s, 3H). **$^{19}$F NMR** (376 MHz, $CDCl_3$) δ = -59.34 (s, 3F). **$^{13}$C NMR** (100 MHz, $CDCl_3$) δ = 165.6 (q, $J$ = 2.1 Hz), 165.0, 163.7, 137.8 (q, $J$ = 2.2 Hz), 136.3, 131.5, 130.9, 130.6 (q, $J$ = 32.2 Hz), 128.9, 127.6 (q, $J$ = 1.5 Hz), 122.2 (q, $J$ = 276.0 Hz), 116.5, 114.7, 55.7. **HRMS**: calculated for $C_{18}H_{12}ClF_3NO_3$ [M + H$^+$] = 382.0458; found: 382.0452.

**(*Z*)-2-(4-methoxyphenyl)-4-(2,2,2-trifluoro-1-(*m*-tolyl)ethylidene)oxazol-5(4*H*)-one (1g).**

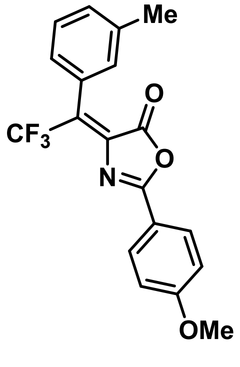

From 3'-methyl-2,2,2-trifluoroacetophenone. Isolated as single diastereoisomer in 49% yield, as yellow crystals. **$^1$H NMR** (400 MHz $CDCl_3$) δ = 8.20 – 8.08 (m, 2H), 7.42 – 7.34 (m, 1H), 7.34 – 7.27 (m, 1H), 7.21 – 7.12 (m, 2H), 7.05 – 6.98 (m, 2H), 3.91 (s, 3H), 2.42 (s, 3H). **$^{19}$F NMR** (376 MHz, $CDCl_3$) δ = -59.35 (s, 3F). **$^{13}$C NMR** (101 MHz, $CDCl_3$) δ = 165.3 (q, $J$ = 2.2 Hz), 165.0, 163.9, 138.3, 137.5 (q, $J$ = 2.2 Hz), 132.6 (q, $J$ = 32.1 Hz), 131.4, 130.9, 129.9, 129.3 (q, $J$ = 1.5 Hz), 128.5, 126.6, 122.5 (q, $J$ = 276.8 Hz), 116.8, 114.8, 55.8, 21.5. **HRMS**: calculated for $C_{19}H_{15}F_3NO_3$ [M + H$^+$] = 362.1004; found: 362.1001.

**(*Z*)-2-(4-methoxyphenyl)-4-(2,2,2-trifluoro-1-(4-fluorophenyl)ethylidene)oxazol-5(4*H*)-one (1h).**

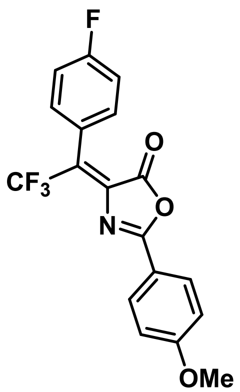


From 2,2,2,4'-tetrafluoroacetophenone. Isolated as single diastereoisomer in 51% yield, as yellow crystals. **$^{1}$H NMR** (400 MHz CDCl$_3$) δ = 8.22 – 8.05 (m, 2H), 7.40 – 7.31 (m, 2H), 7.20 – 7.10 (m, 2H), 7.06 – 6.99 (m, 2H), 3.92 (s, 3H). **$^{19}$F NMR** (376 MHz, CDCl$_3$) δ = -59.47 (s, 3F), -110.00 – -110.20 (m, 1F). **$^{13}$C NMR** (101 MHz, CDCl$_3$) δ = 165.6 (q, *J* = 2.1 Hz), 165.0, 163.8, 163.7 (d, *J* = 250.4 Hz), 137.8 (q, *J* = 2.2 Hz), 131.8 (d, *J* = 8.8 Hz), 131.6, 130.9 (q, *J* = 32.5 Hz), 125.33 – 125.07 (m), 122.4 (dq, *J* = 276.6, 1.0 Hz), 116.7, 115.9 (d, *J* = 22.0 Hz), 114.9, 55.8. **HRMS**: calculated for $C_{18}H_{12}F_4NO_3$ [M + H$^+$] = 366.0753; found: 348.0747.

**(*Z*)-2-(4-methoxyphenyl)-4-(2,2,2-trifluoro-1-(4-methoxyphenyl)ethylidene)oxazol-5(4*H*)-one (1i).**

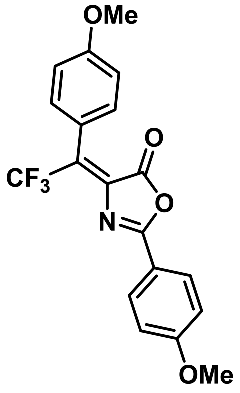


From 4'-methoxy-2,2,2-trifluoroacetophenone. Isolated as single diastereoisomer in 30% yield, as orange crystals. **$^{1}$H NMR** (400 MHz CDCl$_3$) δ = 8.12 (br d, *J* = 8.9 Hz, 2H), 7.33 (br d, *J* = 8.4 Hz, 1H), 7.04 – 6.95 (m, 4H), 3.90 (s, 3H), 3.86 (s, 3H). **$^{19}$F NMR** (376 MHz, CDCl$_3$) δ = -59.25 (s, 3F). **$^{13}$C NMR** (101 MHz, CDCl$_3$) δ = 164.8, 164.7 (q, *J* = 2.1 Hz), 164.2, 161.3, 137.0 (q, *J* = 2.1 Hz), 132.6 (q, *J* = 31.8 Hz), 131.5, 131.4, 122.6 (q, *J* = 277.1 Hz), 121.27 – 121.20 (m), 116.9, 114.8, 114.0, 55.8, 55.4. **HRMS**: calculated for $C_{19}H_{15}F_3NO_4$ [M + H$^+$] = 378.0953; found: 378.0949.

**(*Z*)-4-(1-(3,5-difluorophenyl)-2,2,2-trifluoroethylidene)-2-(4-methoxyphenyl)oxazol-5(4*H*)-one (1j).**

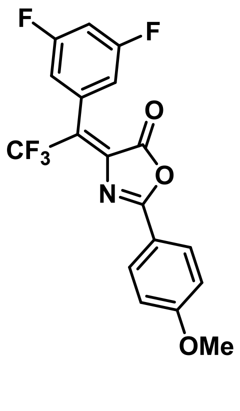


From 2,2,2,3',5'-pentafluoroacetophenone. Isolated as single diastereoisomer in 36% yield, as orange crystals. **$^{1}$H NMR** (400 MHz CDCl$_3$) δ = 8.18 – 8.09 (m, 2H), 7.08 – 7.00 (m, 2H), 6.94 (tt, *J* = 8.9, 2.3 Hz, 1H), 6.93 – 6.85 (m, 2H), 3.93 (s, 3H). **$^{19}$F NMR** (376 MHz, CDCl$_3$) δ = -59.47 (s, 3F), -108.62 – -108.74 (m, 2F). **$^{13}$C NMR** (101 MHz, CDCl$_3$) δ = 166.5 (q, *J* = 2.1 Hz), 165.4, 163.3, 162.9 (dd, *J* = 250.0, 12.9 Hz), 138.7 (q, *J* = 2.1 Hz), 132.39 – 132.02 (m), 131.8, 129.22 – 128.03 (m),

122.0 (q, *J* = 276.5 Hz), 116.4, 114.9, 113.57 – 112.59 (m), 105.7 (t, *J* = 25.0 Hz), 55.9. **HRMS**: calculated for $C_{18}H_{11}F_5NO_3$ [M + H$^+$] = 384.0659; found: 384.0650.

**(*E*)-2-(4-methoxyphenyl)-4-(2,2,2-trifluoro-1-(thiophen-2-yl)ethylidene)oxazol-5(4*H*)-one (1k).**

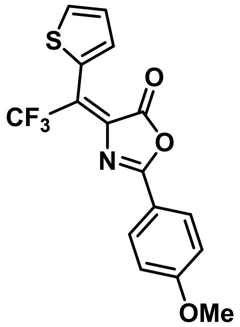


From 2-(trifluoroacetyl)thiophene. Isolated as single diastereoisomer in 41% yield as orange crystals. **$^1$H NMR** (400 MHz CDCl$_3$) δ = 8.17 – 8.07 (m, 2H), 7.62 (dd, *J* = 5.1, 1.2 Hz, 1H), 7.34 (ddd, *J* = 3.7, 1.3, 0.6 Hz, 1H), 7.16 (dd, *J* = 5.1, 3.7 Hz, 1H), 7.08 – 6.97 (m, 2H), 3.91 (s, 3H). **$^{19}$F NMR** (376 MHz, CDCl$_3$) δ = -59.85 (s, 3F). **$^{13}$C NMR** (101 MHz, CDCl$_3$) δ = 165.1, 165.0 (q, *J* = 2.2 Hz), 163.8, 138.1 (q, *J* = 1.8 Hz), 132.4, 131.6, 130.6, 128.9 (q, *J* = 1.6 Hz), 127.6, 125.4 (q, *J* = 33.3 Hz), 122.1 (q, *J* = 277.4 Hz), 116.7, 114.9, 55.8. **HRMS**: calculated for $C_{16}H_{11}F_3NO_3S$ [M + H$^+$] = 354.0412; found: 354.0407.

**(*Z*)-2-(4-methoxyphenyl)-4-(2,2,2-trifluoro-1-(1-tosyl-1*H*-indol-3-yl)ethylidene)oxazol-5(4H)-one (1l).**

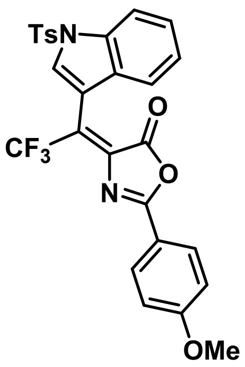


From 2,2,2-trifluoro-1-(1-tosyl-1*H*-indol-3-yl)ethan-1-one. Obtained as single diastereoisomer in 39% yield as bright yellow crystals. **$^1$H NMR** (400 MHz, CDCl$_3$) δ = 8.23-8.12 (m, 2H), 7.98-7.94 (m, 1H), 7.89-7.82 (m, 3H), 7.43-7.39 (m, 1H), 7.37-7.31 (m, 1H), 7.30-7.23 (m, 3H), 7.06-7.01 (m, 2H), 3.91 (s, 3H), 2.35 (s, 3H). **$^{19}$F NMR** (376 MHz, CDCl$_3$) δ = -59.33 (s, 3F). **$^{13}$C NMR** (101 MHz, CDCl$_3$) δ = 165.0, 164.9 (q, *J* = 2.2 Hz), 163.6, 145.4, 138.7 (q, *J* = 1.9 Hz), 134.7, 134.6, 131.4, 130.0, 129.1, 128.7, 127.1, 125.1, 123.8, 123.8 (q, *J* = 33.5 Hz), 122.3 (q, *J* = 280.1 Hz), 120.4, 116.4, 114.7, 113.6, 110.4, 55.6, 21.6. **HRMS**: calculated for $C_{27}H_{20}F_3N_2O_5S$ [M + H$^+$] = 541.1045; found: 541.1036.

**(*Z*)-4-(2,2,3,3,4,4,4-heptafluoro-1-phenylbutylidene)-2-(4-methoxyphenyl)oxazol-5(4*H*)-one (1m).**

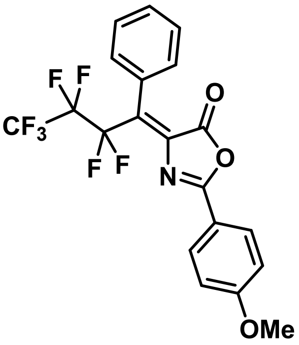

From 2,2,3,3,4,4,4-heptafluoro-1-phenylbutan-1-one. Obtained as single diastereoisomer in 7% yield as beige crystals. Low yield due to product losses during crystallization (high solubility in *n*-hexane). **$^1$H NMR** (400 MHz $CDCl_3$) δ = 8.14-8.09 (m, 2H), 7.52-7.44 (m, 3H), 7.34-7.30 (m, 2H), 7.05-7.00 (m, 2H), 3.89 (s, 3H). **$^{19}$F NMR** (376 MHz, $CDCl_3$) δ = -80.25 (t, *J* = 10.3 Hz, 3F), -106.29 - -106.46 (m, 2F), -123.27 - -123.41 (m, 2F). **$^{13}$C NMR** (101 MHz, $CDCl_3$) [$^{13}$C signals of the perfluoroalkyl chain not reported as they were weakly detected due to their multiplicity] δ = 165.4 (t, *J* = 3.0 Hz), 164.9, 163.5, 139.9 (t, *J* = 3.4 Hz), 131.4, 131.3 (t, *J* = 22.5 Hz), 129.8 (t, *J* = 3.1 Hz), 129.8, 129.7, 128.5, 116.7, 114.7, 55.6. **HRMS**: calculated for $C_{20}H_{13}F_7NO_3$ [M + H$^+$] = 448.0784; found: 448.0781.

**(*Z*)-4-(1-Cyclohexyl-2,2,2-trifluoroethylidene)-2-(4-methoxyphenyl)oxazol-5(4*H*)-one (1n).**

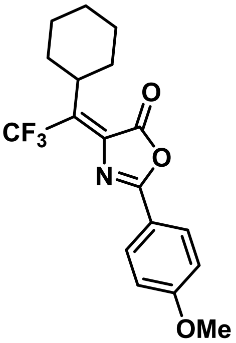

From 1-cyclohexyl-2,2,2-trifluoroethan-1-one, and performing the reaction at 60 °C for 48 h after stirring overnight at RT. Obtained as single diastereoisomer in 10% yield as white crystals. **$^1$H NMR** (400 MHz $CDCl_3$) δ = 8.10-8.05 (m, 2H), 7.03-6.97 (m, 2H), 3.93-3.85 (m, 1H), 3.90 (s, 3H), 1.88-1.80 (m, 2H), 1.79-1.65 (m, 5H), 1.48-1.35 (m, 2H), 1.25 (qt, *J* = 12.9, 3.2 Hz, 1H). **$^{19}$F NMR** (376 MHz, $CDCl_3$) δ = -56.75 (s, 3F). **$^{13}$C NMR** (101 MHz, $CDCl_3$) δ = 165.4, 164.4, 162.8 (q, *J* = 2.2 Hz), 140.8 (q, *J* = 27.8 Hz), 136.4 (q, *J* = 3.6 Hz), 130.9, 123.5 (q, *J* = 279.8 Hz), 116.9, 114.5, 55.6, 37.1, 30.0, 26.4, 25.6. **HRMS**: calculated for $C_{18}H_{19}F_3NO_3$ [M + H$^+$] = 354.1312; found: 354.1304.

**(*Z*)-2-(4-Methoxyphenyl)-4-(1,1,1-trifluorobutan-2-ylidene)oxazol-5(4H)-one (1o).**

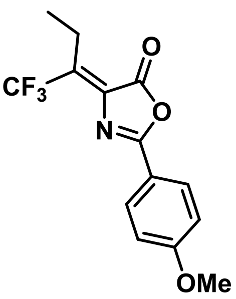

From 1,1,1-trifluorobutan-2-one, warming the reaction mixture for 5 h at 60 °C after stirring overnight at RT. Obtained as single diastereoisomer in 22% yield as white crystals. **$^1$H NMR** (400 MHz $CDCl_3$) δ = 8.10-8.05 (m, 2H), 7.02-6.98 (m, 2H), 3.90 (s, 3H), 2.93 (q, *J* = 7.5 Hz, 2H), 1.23 (t, *J* = 7.5 Hz, 3H). **$^{19}$F NMR** (376 MHz, $CDCl_3$) δ = -61.59 (s, 3F). **$^{13}$C NMR** (101 MHz, $CDCl_3$) δ = 165.2 (br s), 164.5, 163.5 (q, *J* = 2.3 Hz), 137.4 (q, *J* =

29.9 Hz), 136.4 (q, $J$ = 2.9 Hz), 131.0, 123.1 (q, $J$ = 276.2 Hz), 116.8, 114.6, 55.6, 19.8, 13.3. **HRMS**: calculated for $C_{14}H_{13}F_3NO_3$ [M + H$^+$] = 300.0842; found: 300.0834.

**(*Z*)-2-(4-Methoxyphenyl)-4-(1,1,1-trifluoropropan-2-ylidene)oxazol-5(4*H*)-one (1p).**

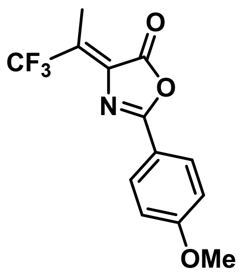


From 1,1,1-trifluoroetan-2-one. Obtained as single diastereoisomer in 11% yield as white crystals. Low yield due to the multiple crystallisations required to obtain Z-pure material. **$^1$H NMR** (400 MHz $CDCl_3$) δ = 8.10-8.04 (m, 2H), 7.03-6.97 (m, 2H), 3.90 (s, 3H), 2.46 (s, 3H). **$^{19}$F NMR** (376 MHz, $CDCl_3$) δ = -63.15 (s, 3F). **$^{13}$C NMR** (101 MHz, $CDCl_3$) δ = 165.6 (br s), 164.5, 163.3 (q, $J$ = 2.4 Hz), 136.3 (q, $J$ = 2.6 Hz), 131.3 (q, $J$ = 31.5 Hz), 131.0, 122.8 (q, $J$ = 275.9 Hz), 116.8, 114.6, 55.6, 12.1 (q, $J$ = 3.0 Hz). **HRMS**: calculated for $C_{13}H_{11}F_3NO_3$ [M + H$^+$] = 286.0686; found: 286.0678.

**Copies of $^{1}H$, $^{19}F$, and $^{13}C$ NMR spectra of substrates 1 (in CDCl3)**

**(*Z*)-2-(4-methoxyphenyl)-4-(2,2,2-trifluoro-1-phenylethylidene)oxazol-5(4*H*)-one (1d)**

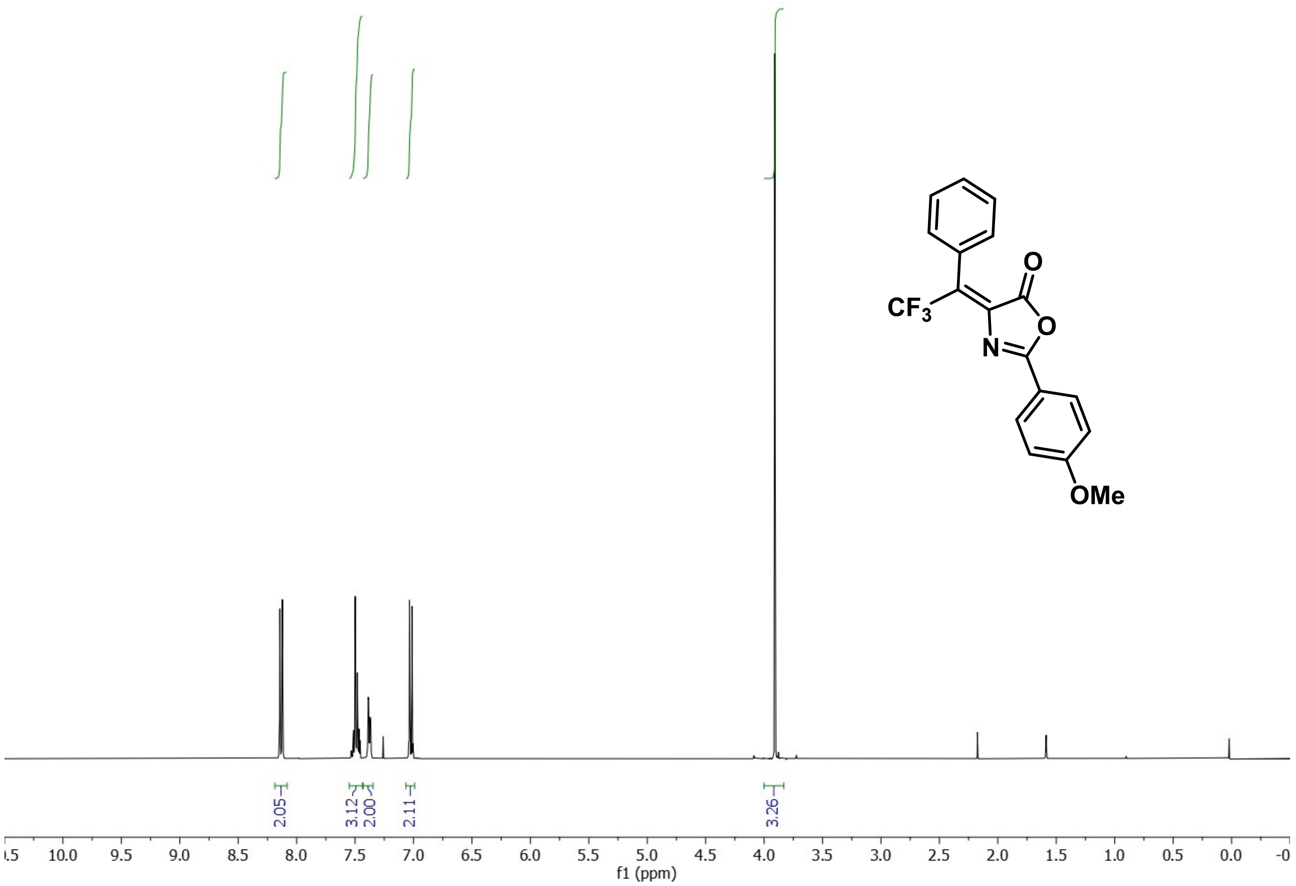

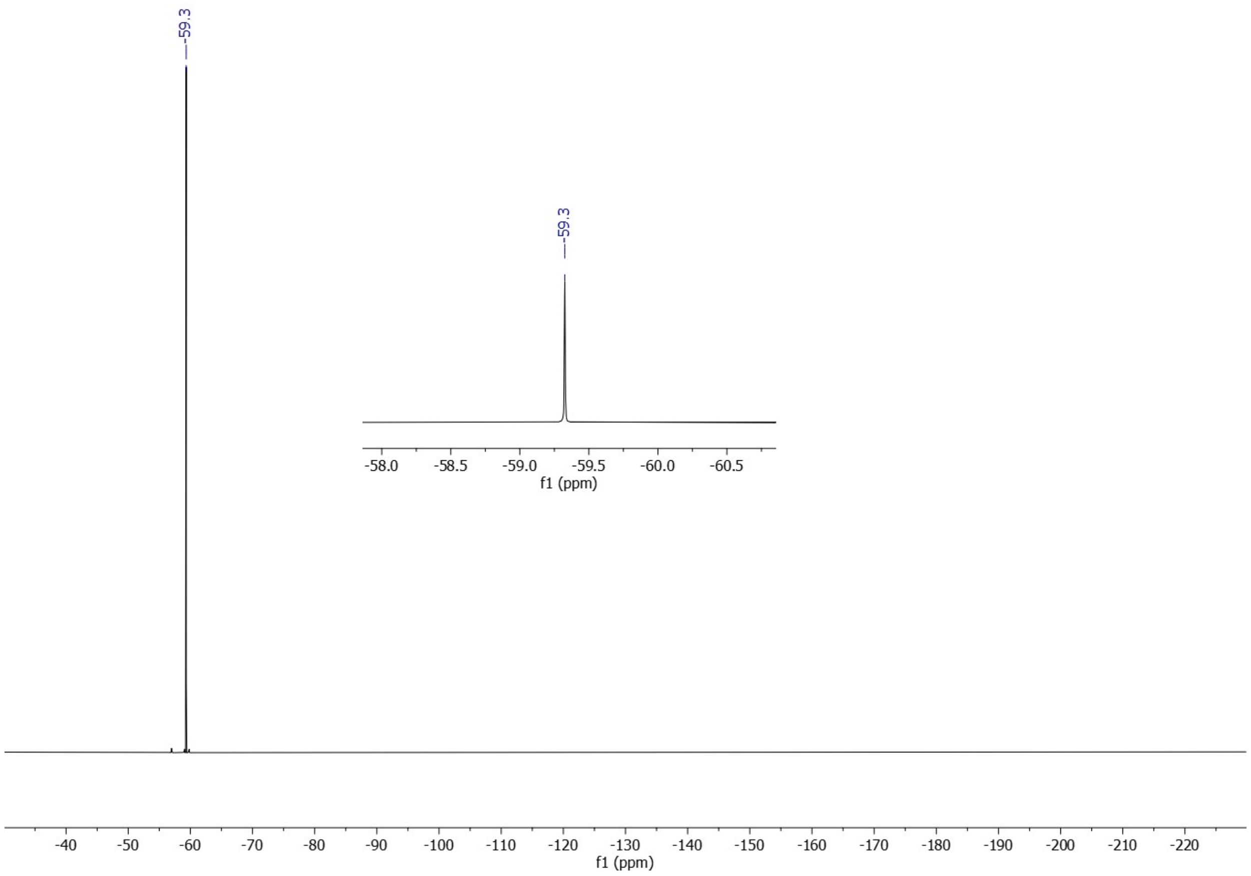
-59.3
-59.3
-58.0 -58.5 -59.0 -59.5 -60.0 -60.5
f1 (ppm)
-40 -50 -60 -70 -80 -90 -100 -110 -120 -130 -140 -150 -160 -170 -180 -190 -200 -210 -220
f1 (ppm)

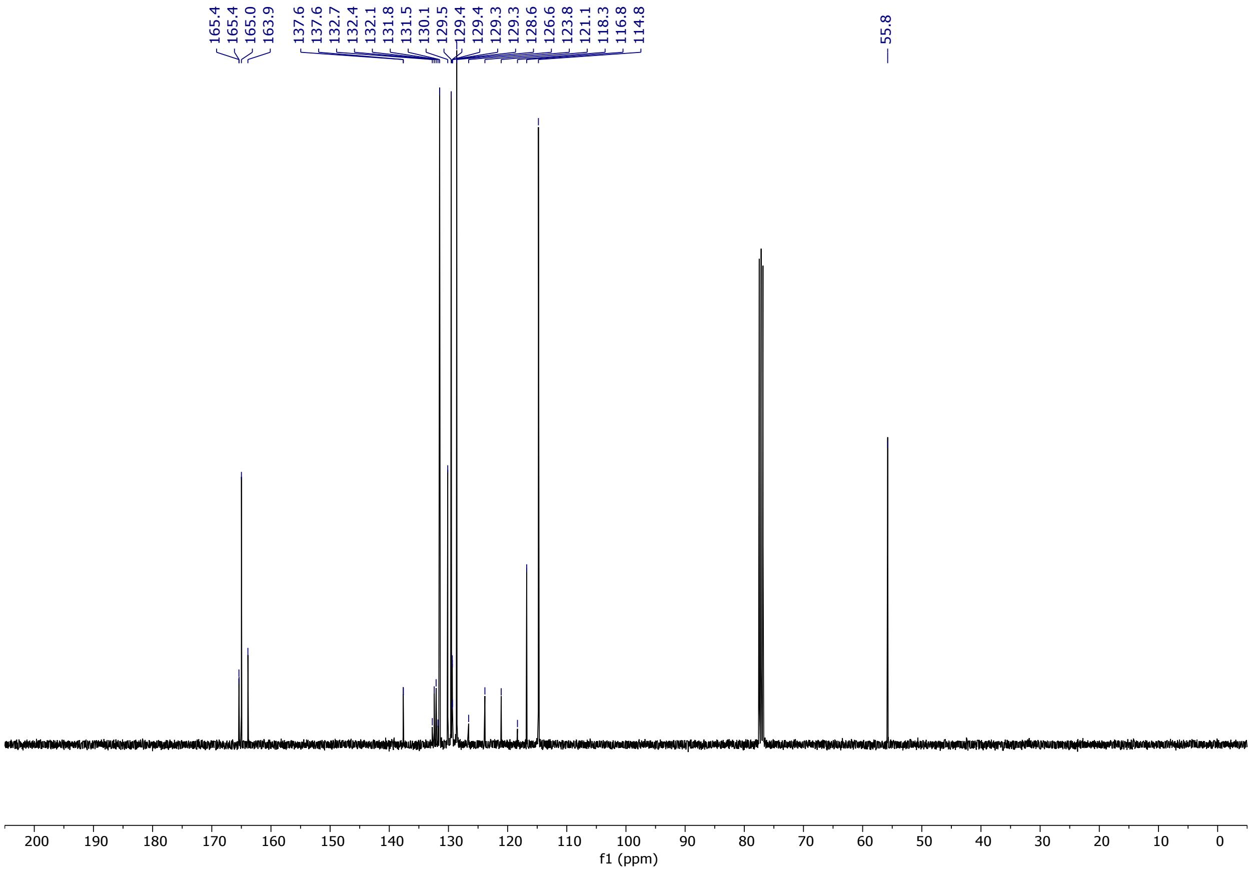
165.4
165.4
165.0
163.9
137.6
137.6
132.7
132.4
132.1
131.8
131.5
130.1
129.5
129.4
129.4
129.3
129.3
128.6
126.6
123.8
121.1
118.3
116.8
114.8
55.8
200 190 180 170 160 150 140 130 120 110 100 90 80 70 60 50 40 30 20 10 0
f1 (ppm)

**(*Z*)-4-(1-(4-bromophenyl)-2,2,2-trifluoroethylidene)-2-(4-methoxyphenyl)oxazol-5(4*H*)-one (1e).**

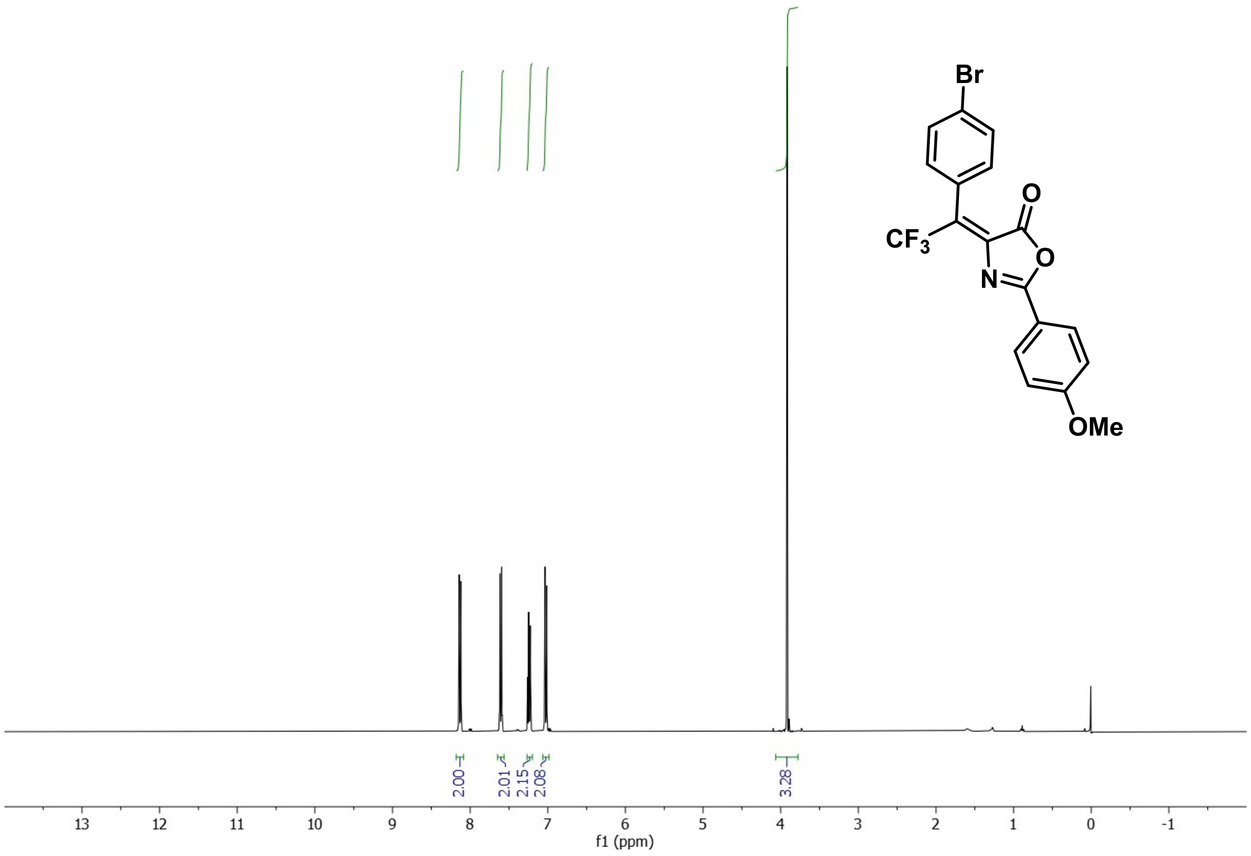

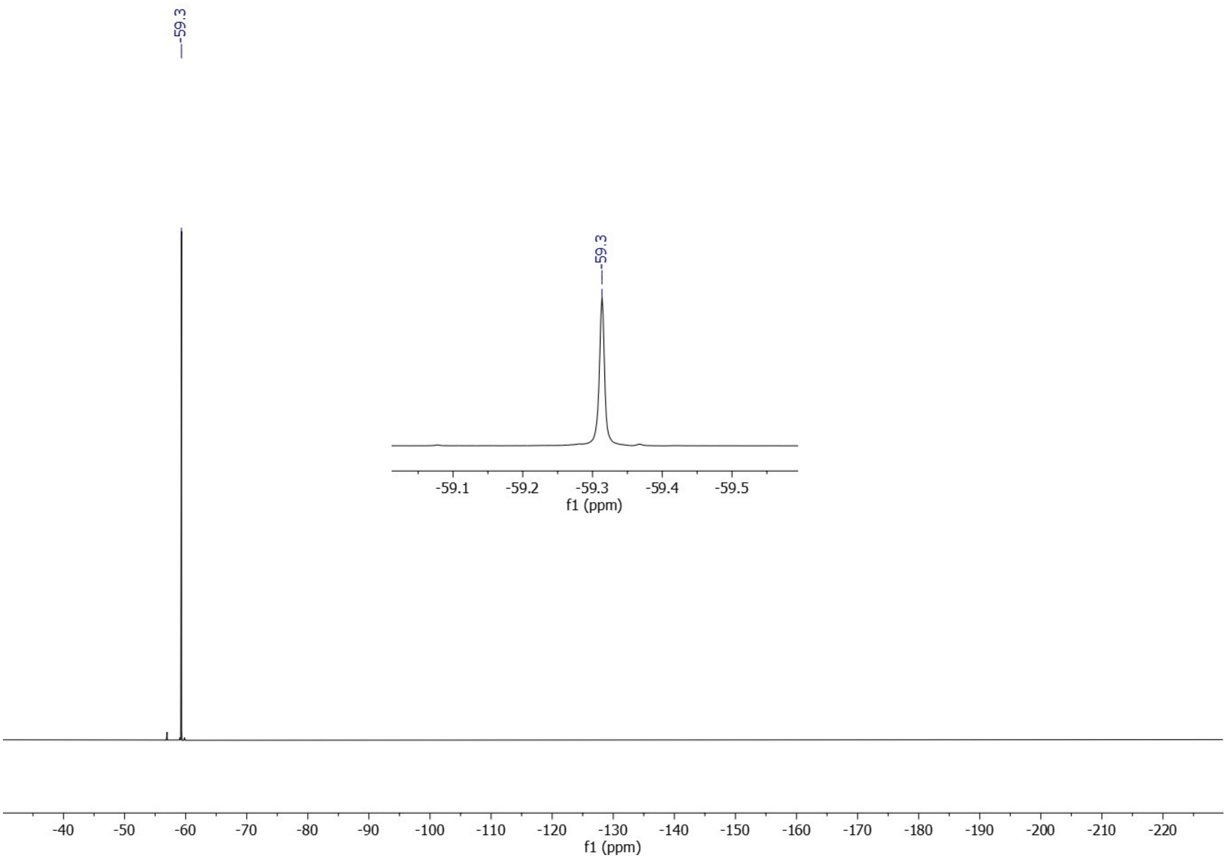

-59.3
-59.3
-59.1
-59.2
-59.3
-59.4
-59.5
f1 (ppm)
-40
-50
-60
-70
-80
-90
-100
-110
-120
-130
-140
-150
-160
-170
-180
-190
-200
-210
-220
f1 (ppm)


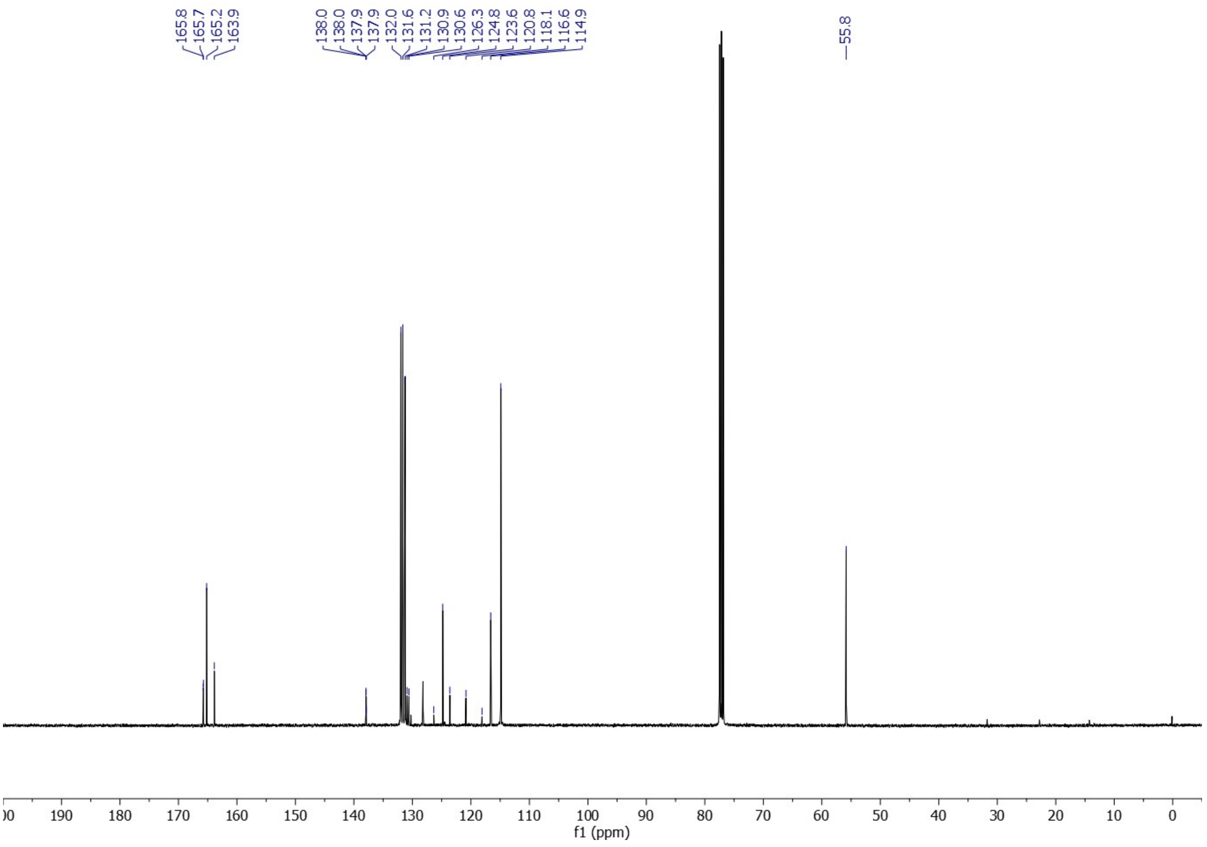

165.8
165.7
165.2
163.9
138.0
138.0
137.9
137.9
132.0
131.6
131.2
130.9
130.6
126.3
124.8
123.6
120.8
118.1
116.6
114.9
55.8
190
180
170
160
150
140
130
120
110
100
90
80
70
60
50
40
30
20
10
0
f1 (ppm)

**(*Z*)-4-(1-(4-chlorophenyl)-2,2,2-trifluoroethylidene)-2-(4-methoxyphenyl)oxazol-5(4*H*)-one (1f).**

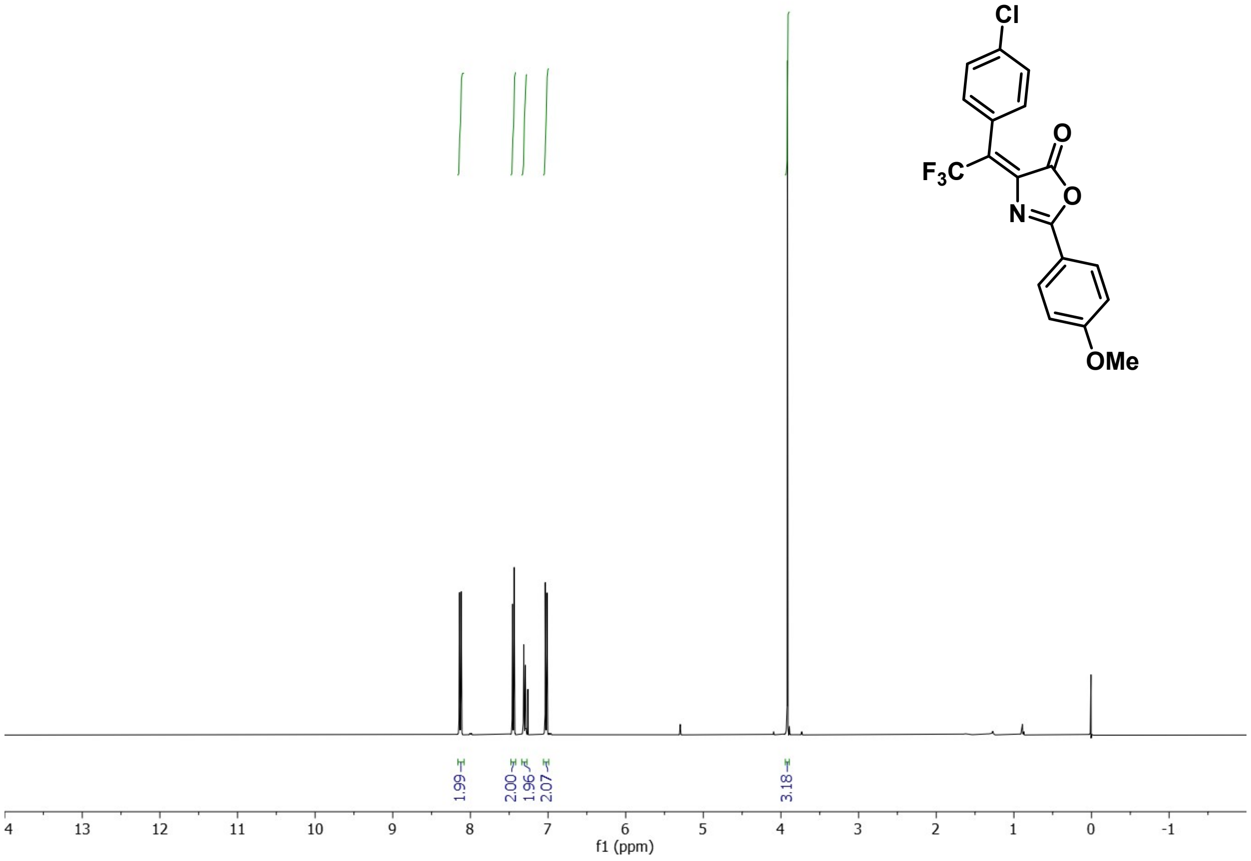

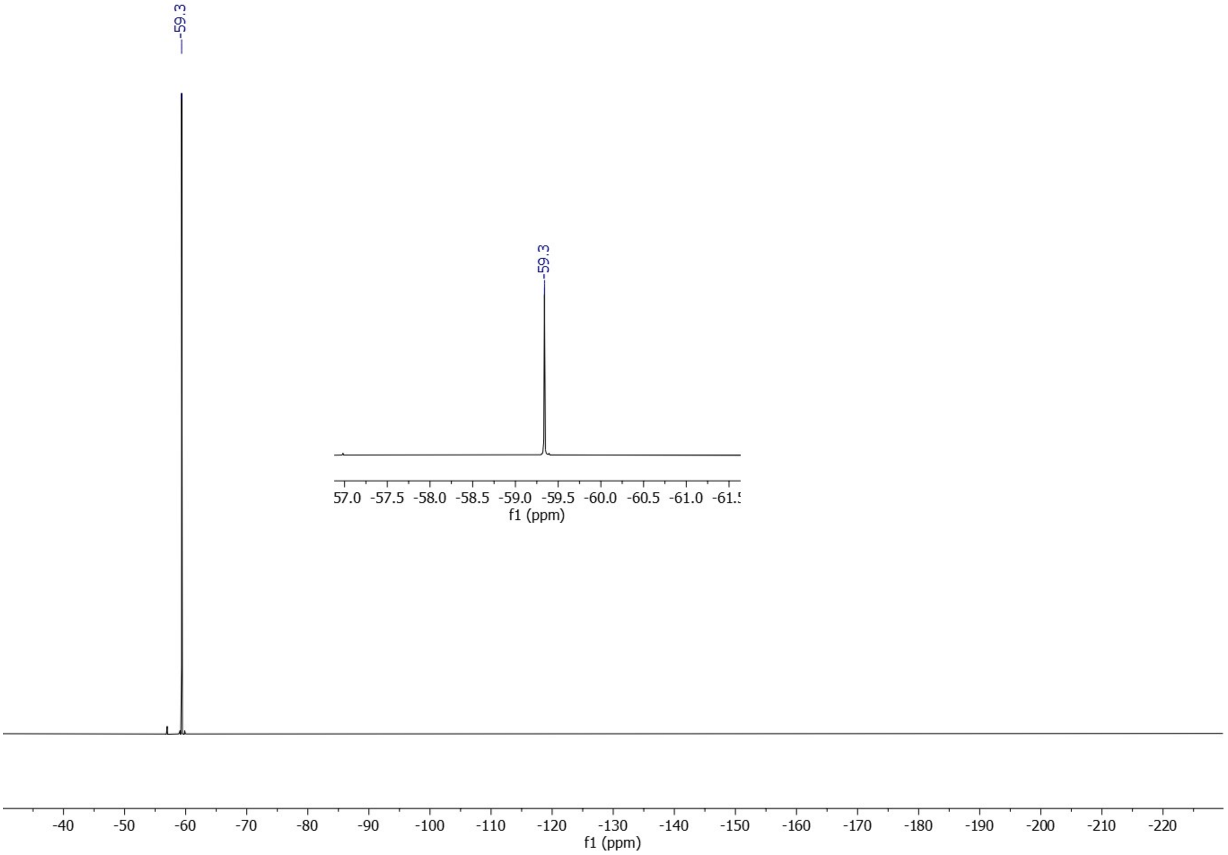
-59.3
-59.3
57.0 -57.5 -58.0 -58.5 -59.0 -59.5 -60.0 -60.5 -61.0 -61.5
f1 (ppm)
-40 -50 -60 -70 -80 -90 -100 -110 -120 -130 -140 -150 -160 -170 -180 -190 -200 -210 -220
f1 (ppm)

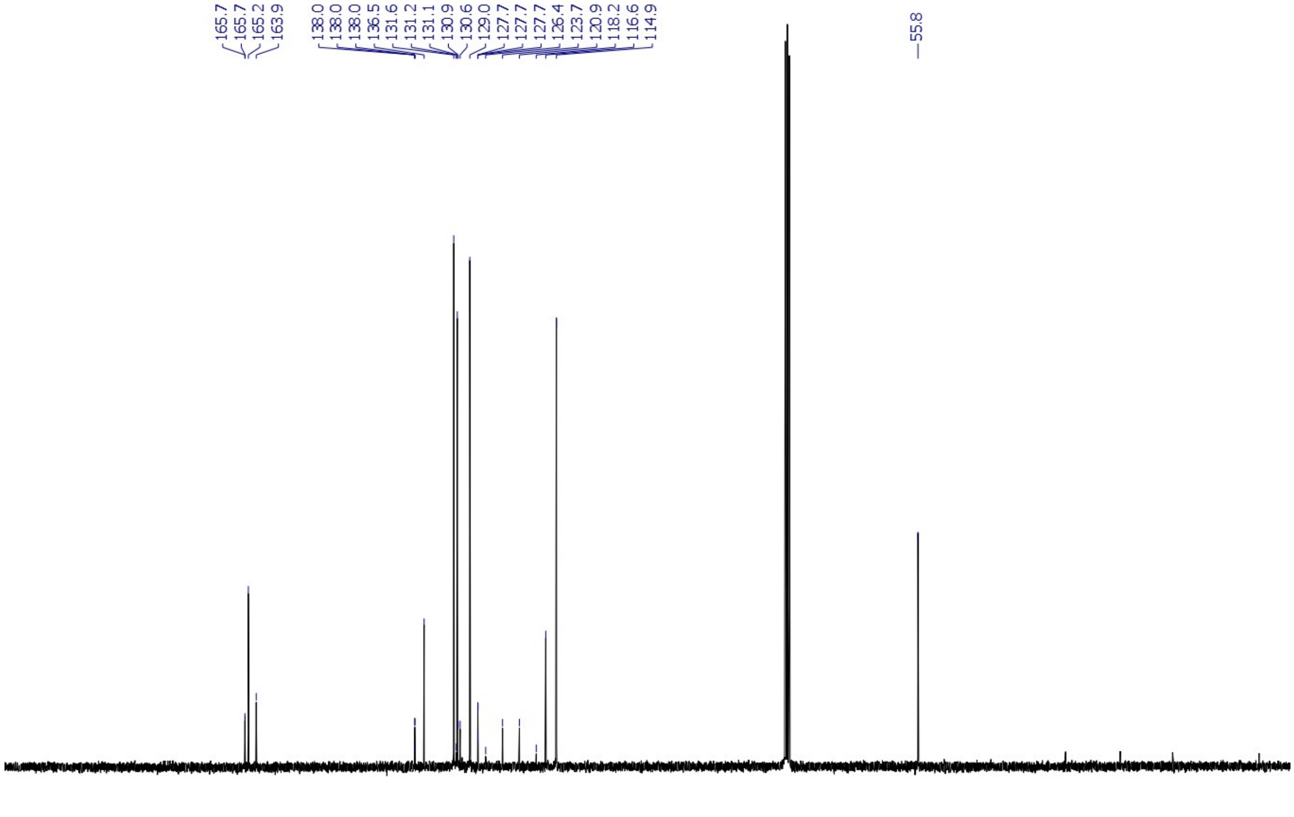
165.7
165.7
165.2
163.9
138.0
138.0
138.0
136.5
131.6
131.2
131.1
130.9
130.6
129.0
127.7
127.7
127.7
126.4
123.7
120.9
118.2
116.6
114.9
55.8
200 190 180 170 160 150 140 130 120 110 100 90 80 70 60 50 40 30 20 10 0
f1 (ppm)

**(*Z*)-2-(4-methoxyphenyl)-4-(2,2,2-trifluoro-1-(*m*-tolyl)ethylidene)oxazol-5(4*H*)-one (1g).**

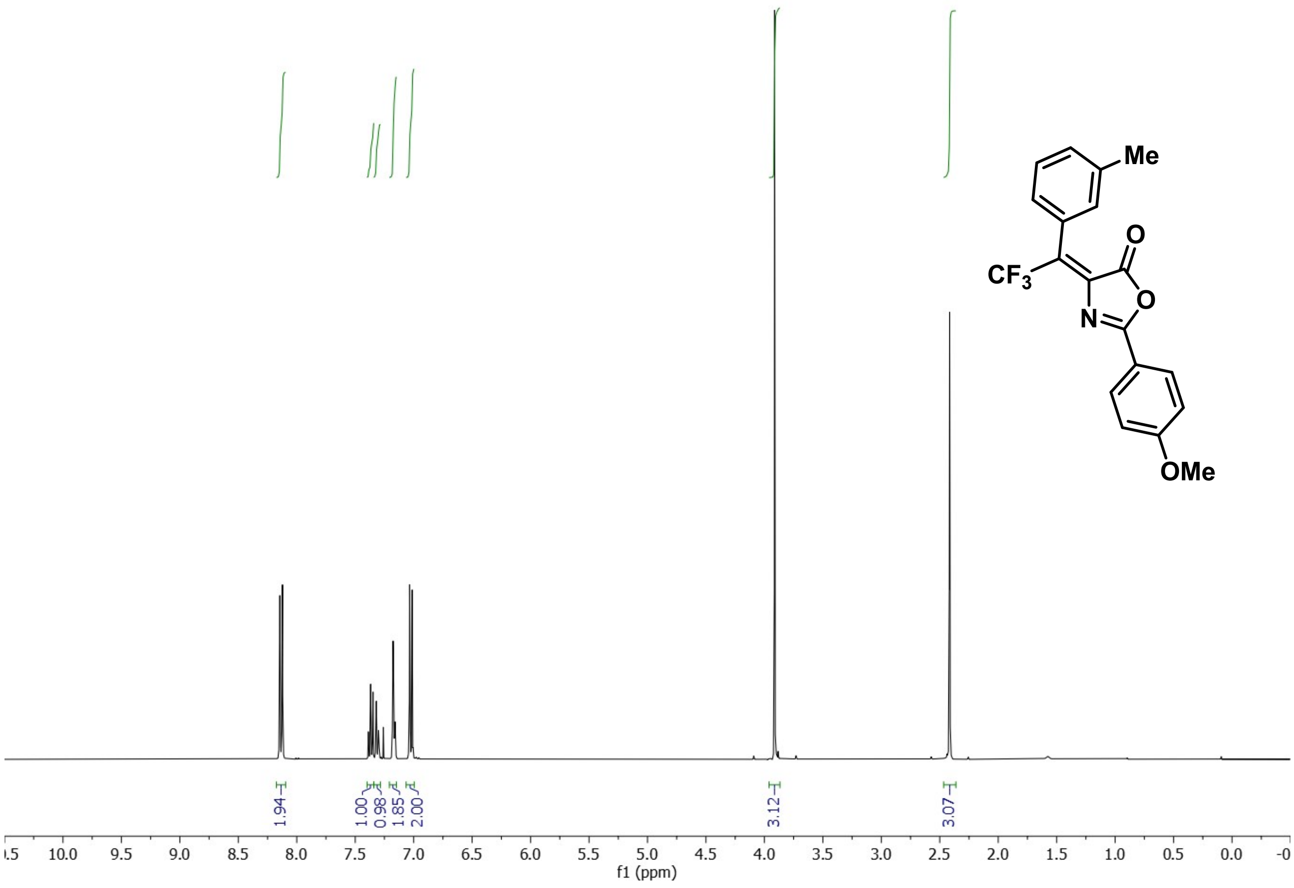

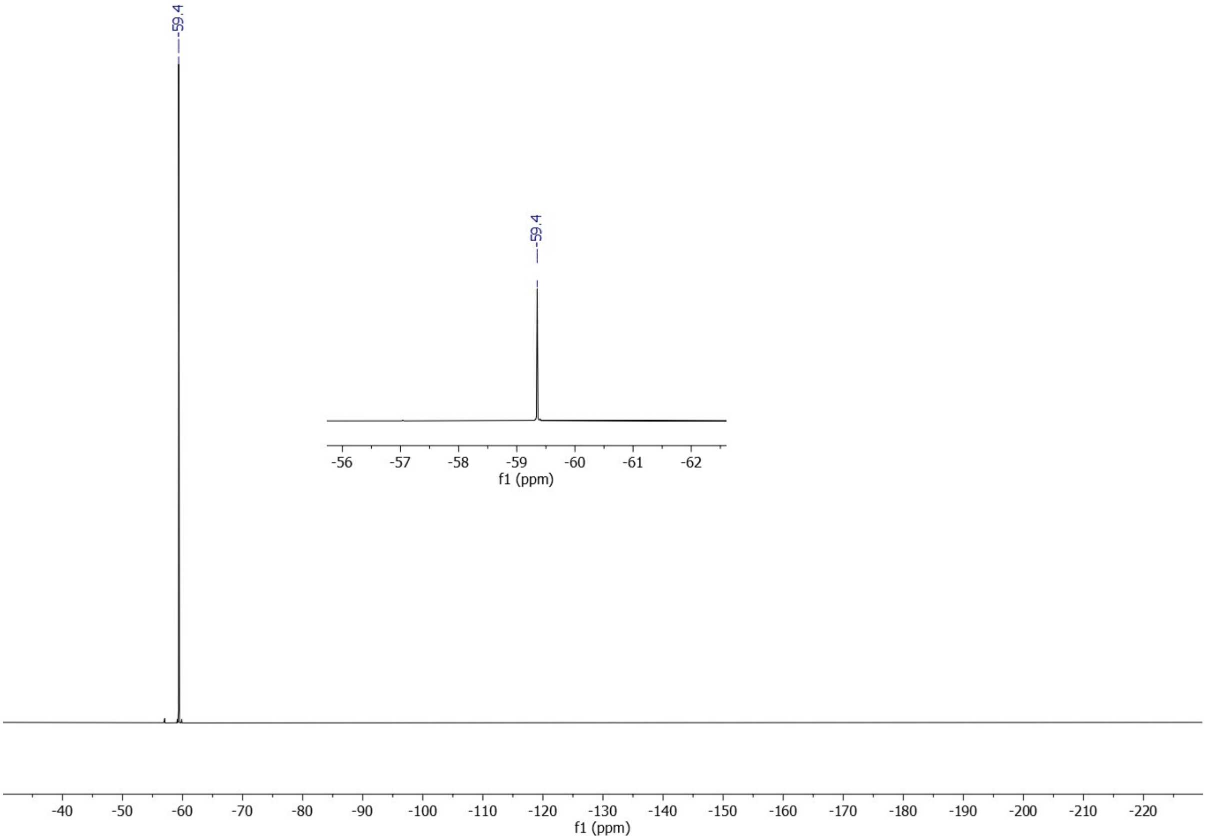
-59.4
-59.4
-56 -57 -58 -59 -60 -61 -62
f1 (ppm)
-40 -50 -60 -70 -80 -90 -100 -110 -120 -130 -140 -150 -160 -170 -180 -190 -200 -210 -220
f1 (ppm)

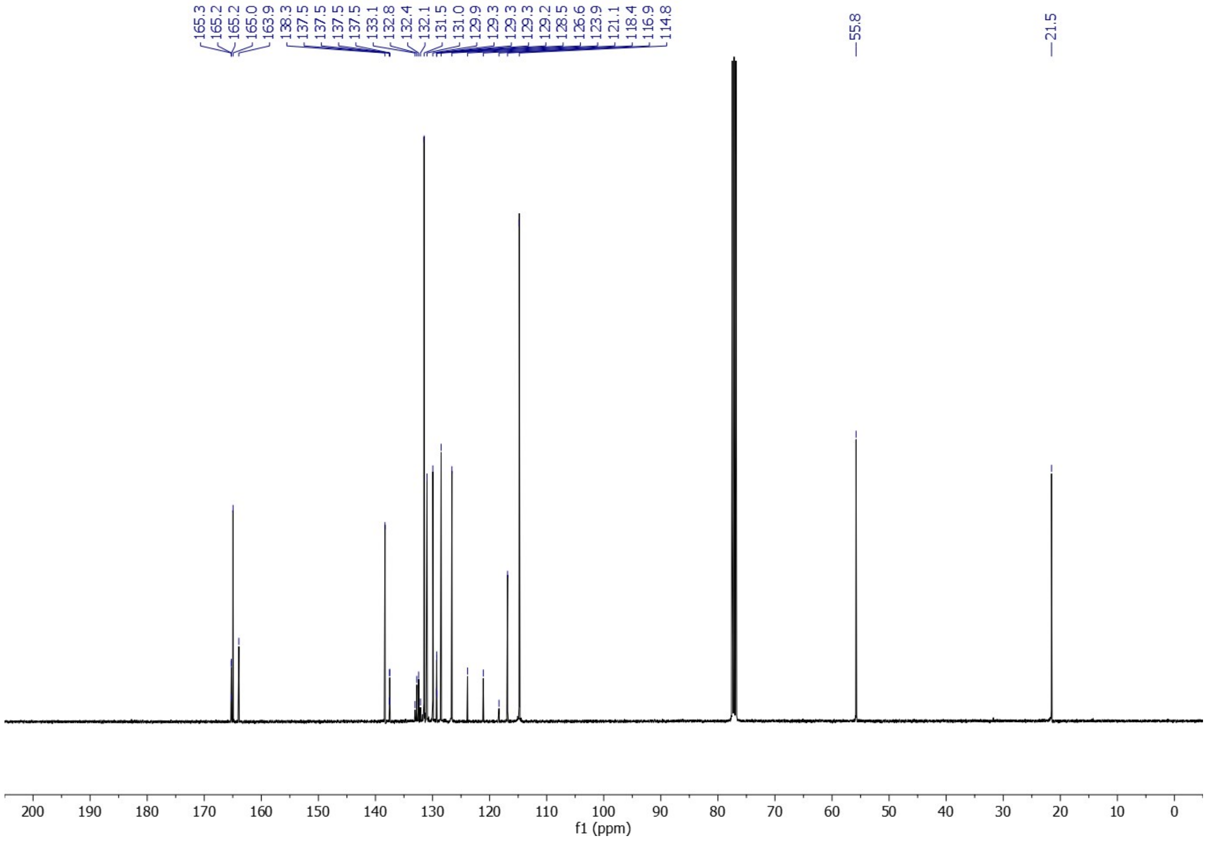
165.3
165.2
165.2
165.0
163.9
138.3
137.5
137.5
137.5
137.5
133.1
132.8
132.4
132.1
131.5
131.0
129.9
129.3
129.3
129.3
129.2
128.5
126.6
123.9
121.1
118.4
116.9
114.8
55.8
21.5
200 190 180 170 160 150 140 130 120 110 100 90 80 70 60 50 40 30 20 10 0
f1 (ppm)

**(*Z*)-2-(4-methoxyphenyl)-4-(2,2,2-trifluoro-1-(4-fluorophenyl)ethylidene)oxazol-5(4*H*)-one (1h).**

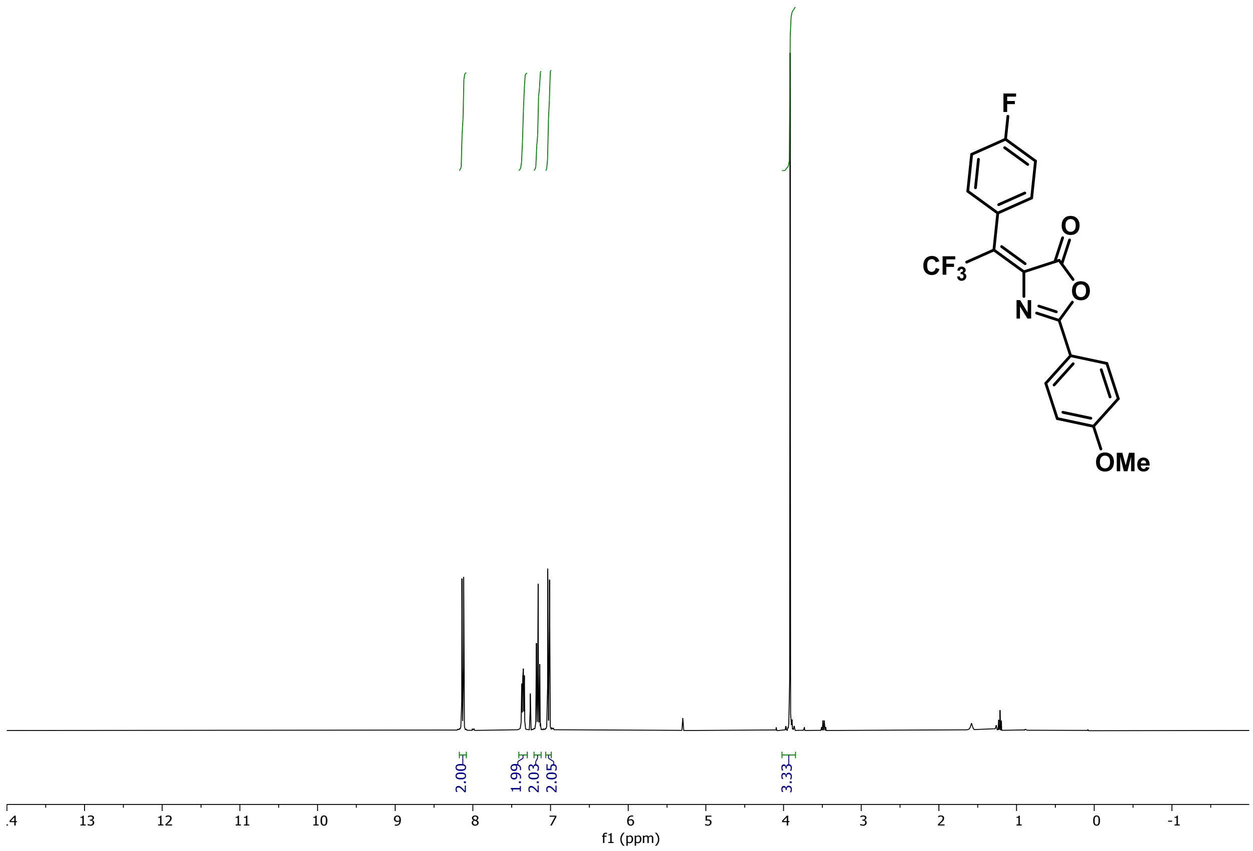

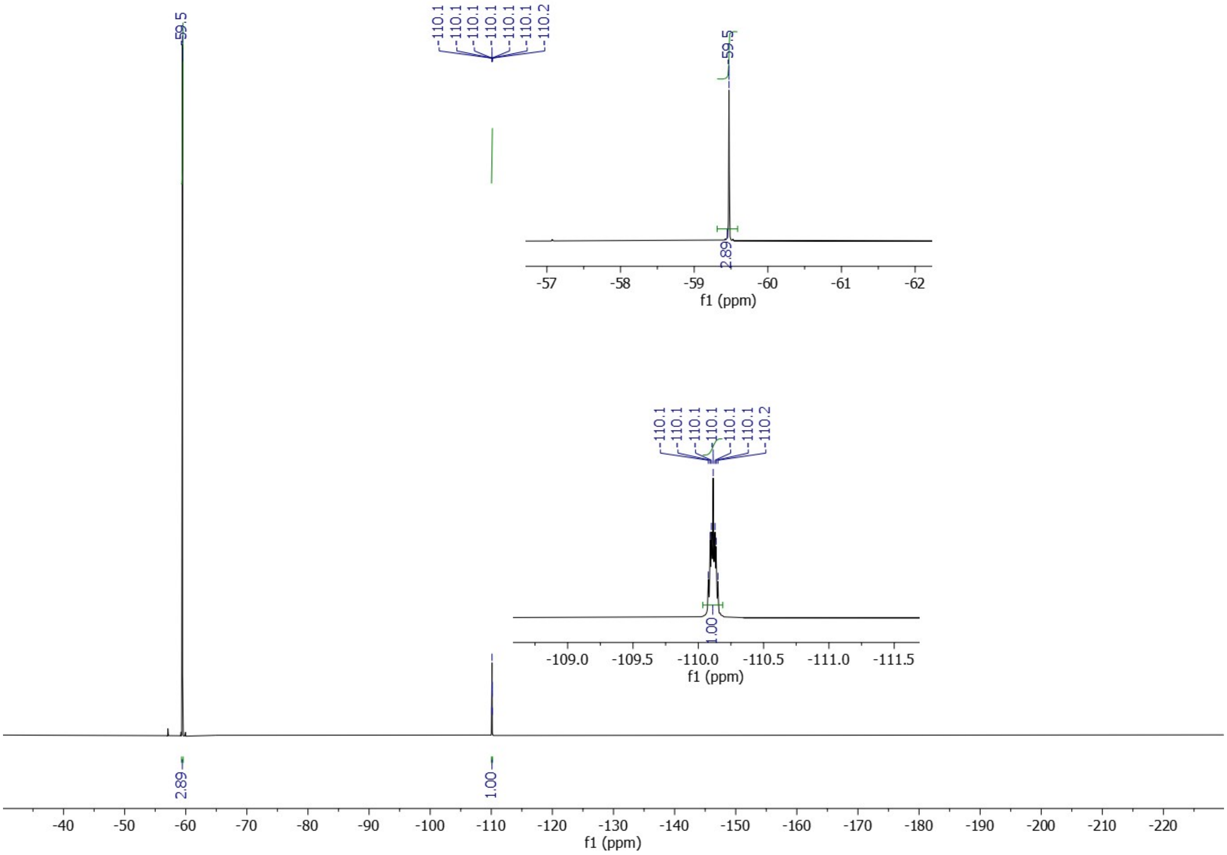
-59.5
-110.1
-110.2
2.89
1.00
f1 (ppm)

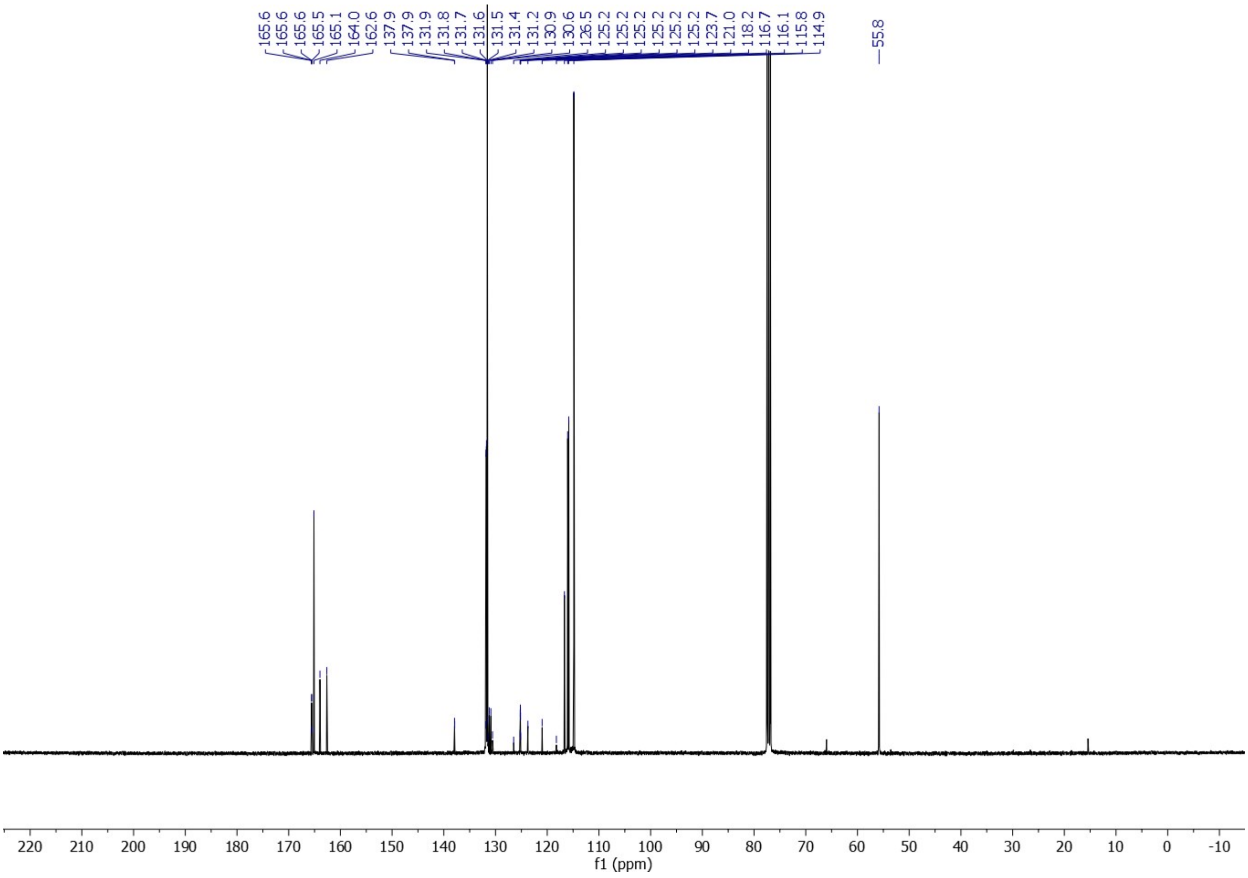
165.6
165.5
165.1
164.0
162.6
137.9
131.9
131.8
131.7
131.6
131.5
131.4
131.2
130.9
130.6
126.5
125.2
123.7
121.0
118.2
116.7
116.1
115.8
114.9
55.8
f1 (ppm)

**(*Z*)-2-(4-methoxyphenyl)-4-(2,2,2-trifluoro-1-(4-methoxyphenyl)ethylidene)oxazol-5(4*H*)-one (1i).**

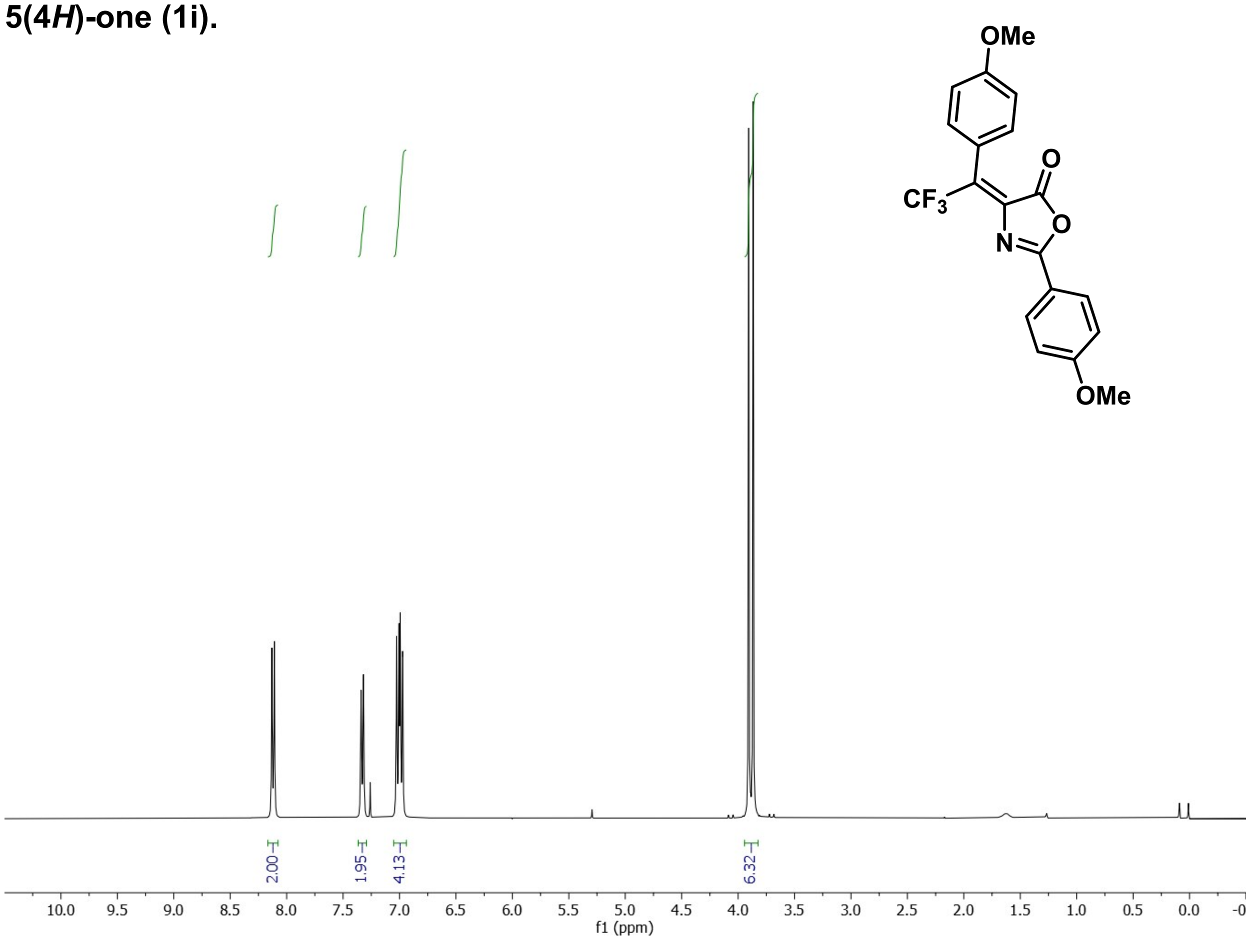

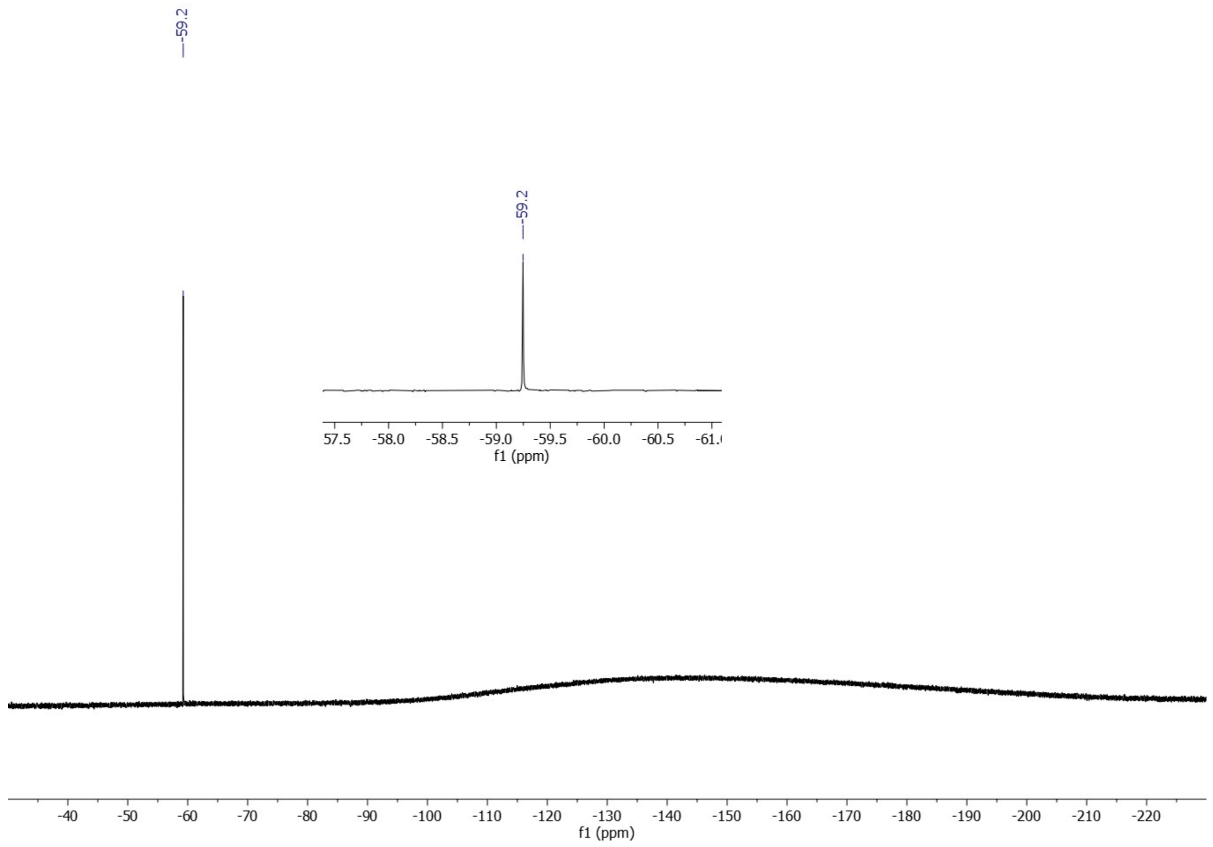

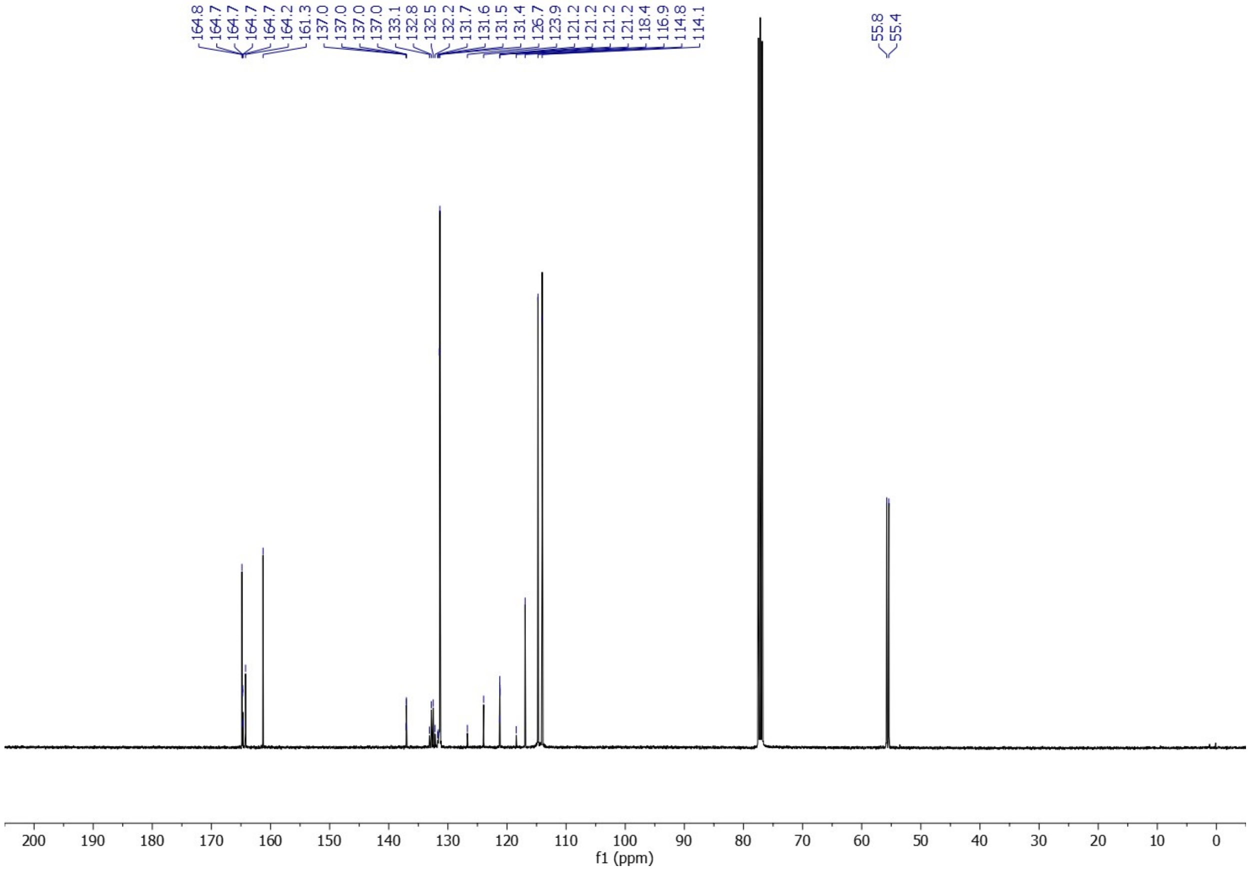

**(*Z*)-4-(1-(3,5-difluorophenyl)-2,2,2-trifluoroethylidene)-2-(4-methoxyphenyl)oxazol-5(4*H*)-one (1j).**

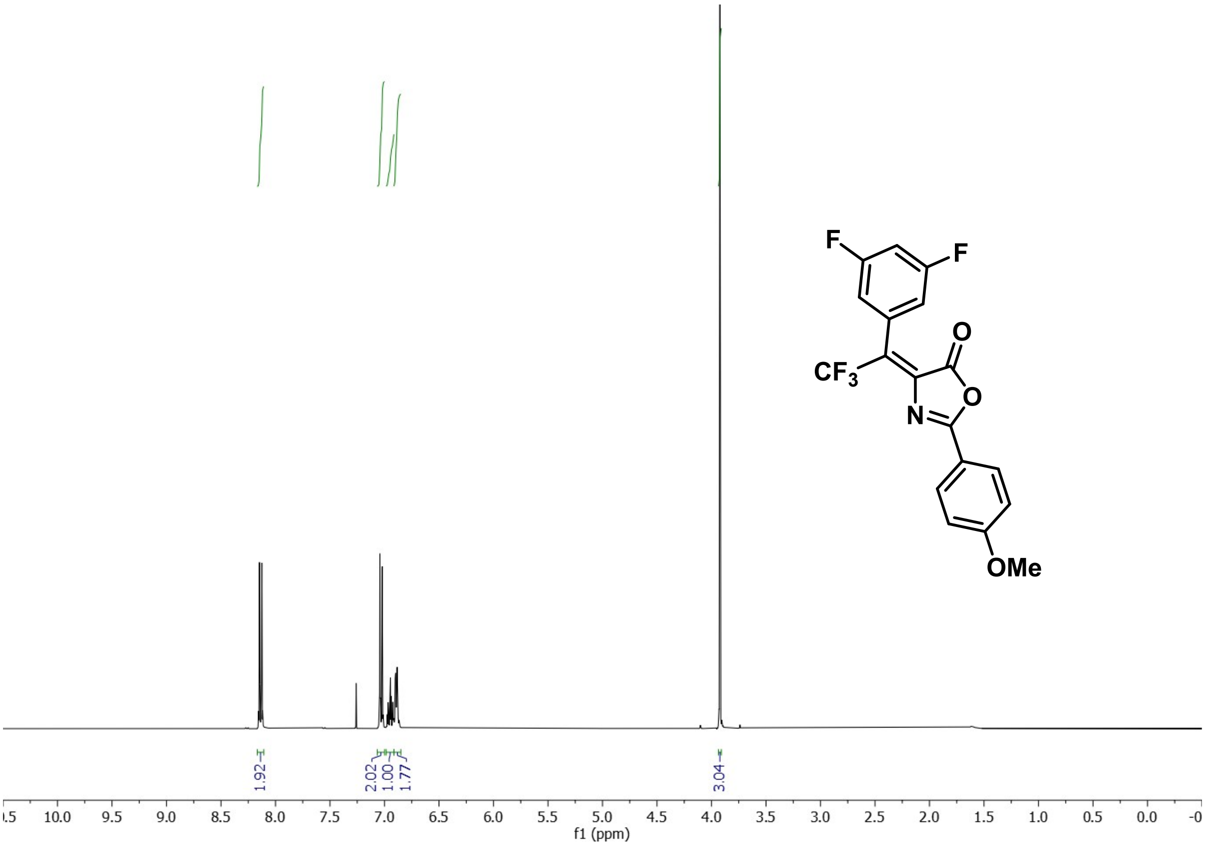

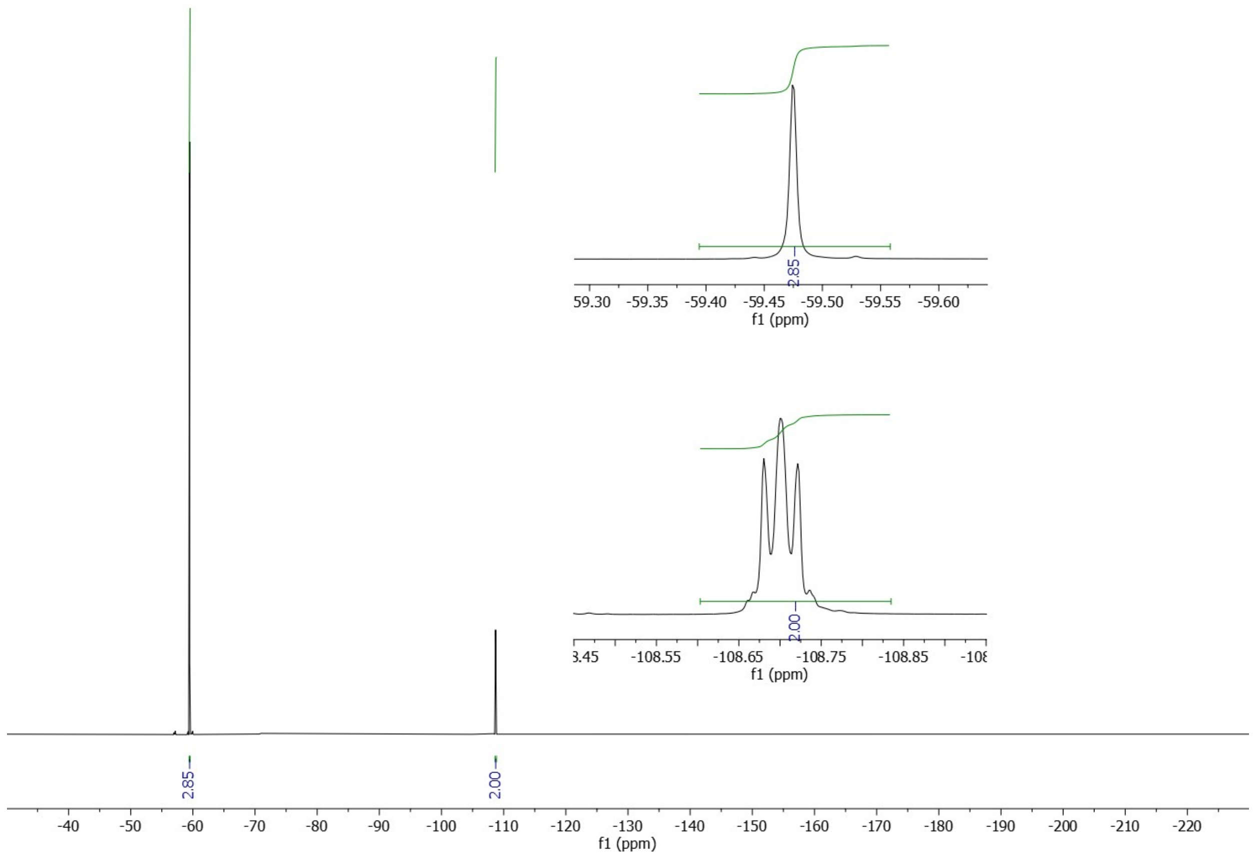

2.85
-59.30 -59.35 -59.40 -59.45 -59.50 -59.55 -59.60
f1 (ppm)
2.00
-108.55 -108.65 -108.75 -108.85
f1 (ppm)
2.85
2.00
-40 -50 -60 -70 -80 -90 -100 -110 -120 -130 -140 -150 -160 -170 -180 -190 -200 -210 -220
f1 (ppm)


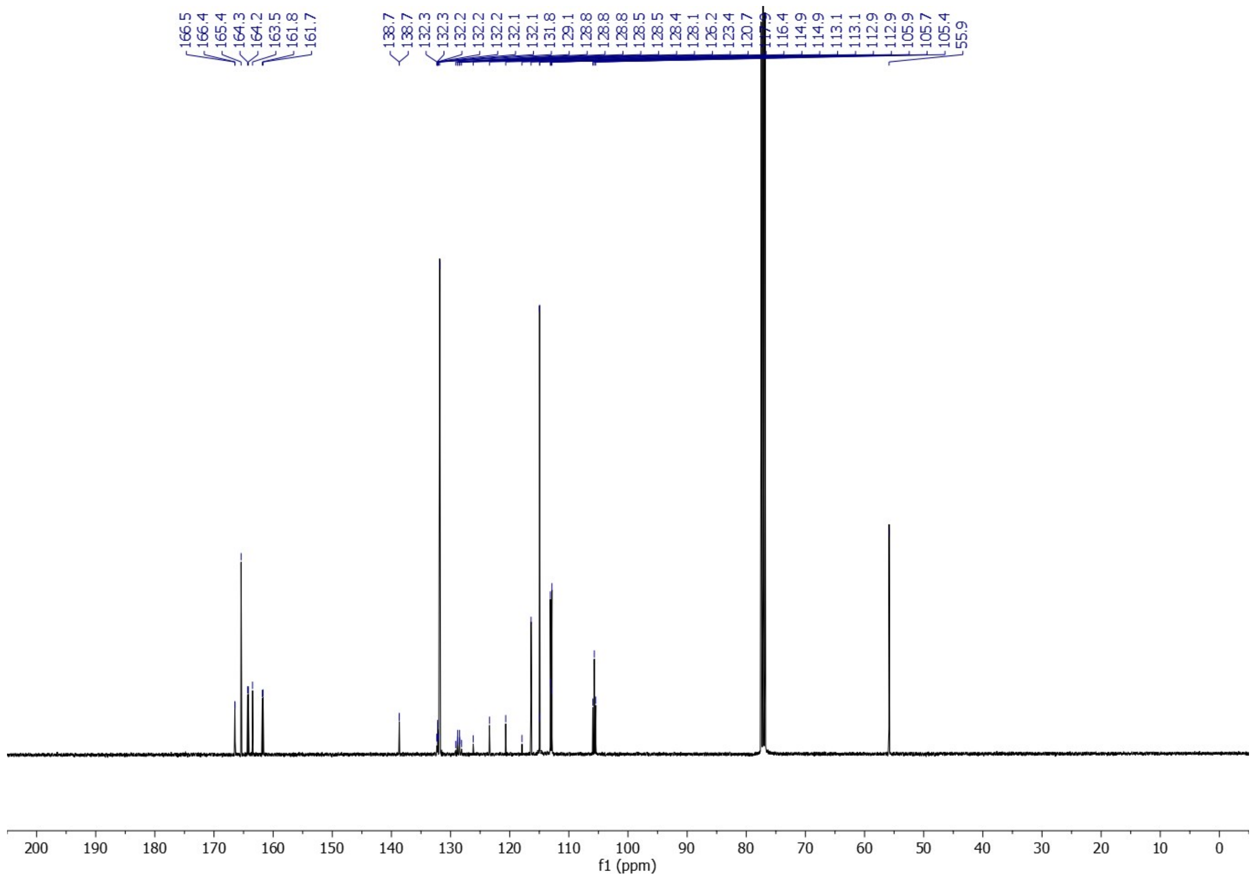

166.5 166.4 165.4 164.3 164.2 163.5 161.8 161.7
138.7 138.7 132.3 132.3 132.2 132.2 132.2 132.1 132.1 131.8 129.1 128.8 128.8 128.8 128.5 128.5 128.4 128.1 126.2 123.4 120.7 117.9 116.4 114.9 114.9 113.1 113.1 112.9 112.9 105.9 105.7 105.4 55.9
200 190 180 170 160 150 140 130 120 110 100 90 80 70 60 50 40 30 20 10 0
f1 (ppm)

**(*E*)-2-(4-methoxyphenyl)-4-(2,2,2-trifluoro-1-(thiophen-2-yl)ethylidene)oxazol-5(4*H*)-one (1k).**

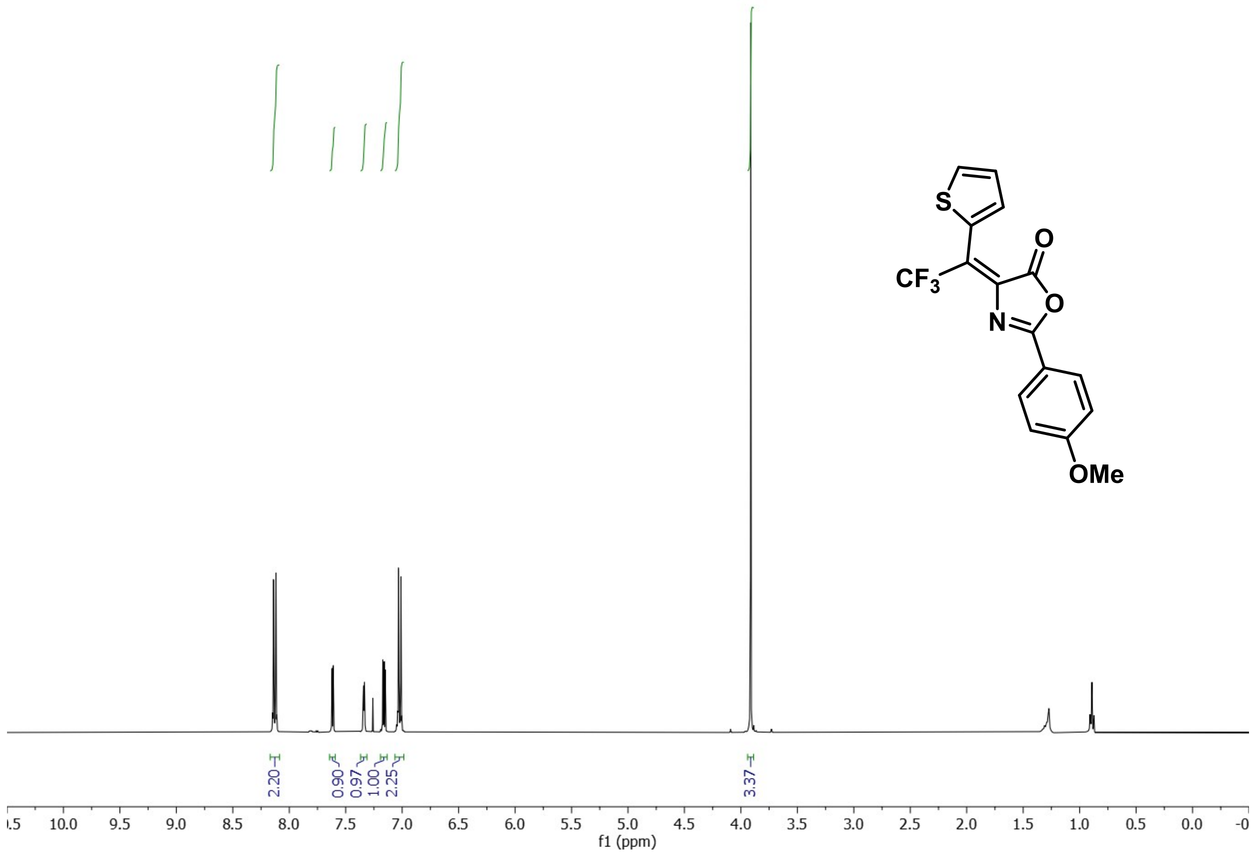

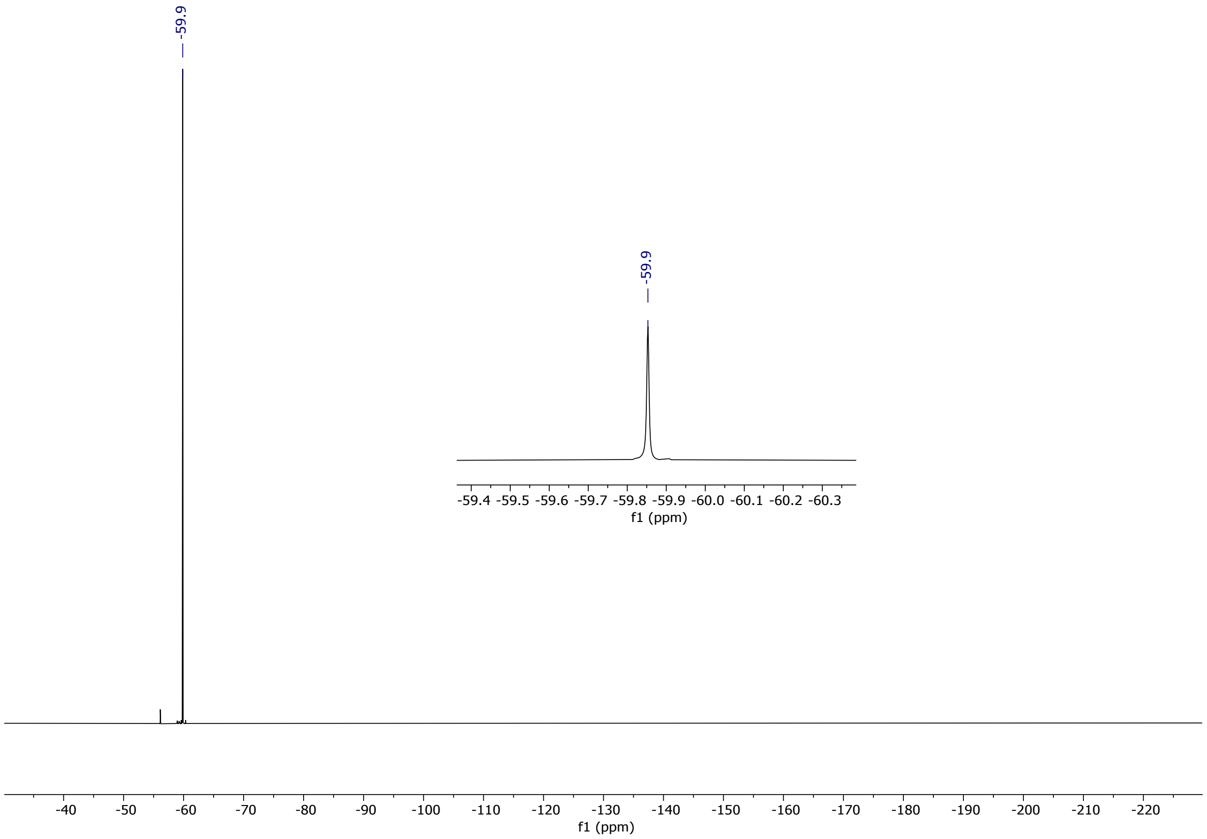

-59.9
-59.9
-59.4 -59.5 -59.6 -59.7 -59.8 -59.9 -60.0 -60.1 -60.2 -60.3
f1 (ppm)
-40 -50 -60 -70 -80 -90 -100 -110 -120 -130 -140 -150 -160 -170 -180 -190 -200 -210 -220
f1 (ppm)


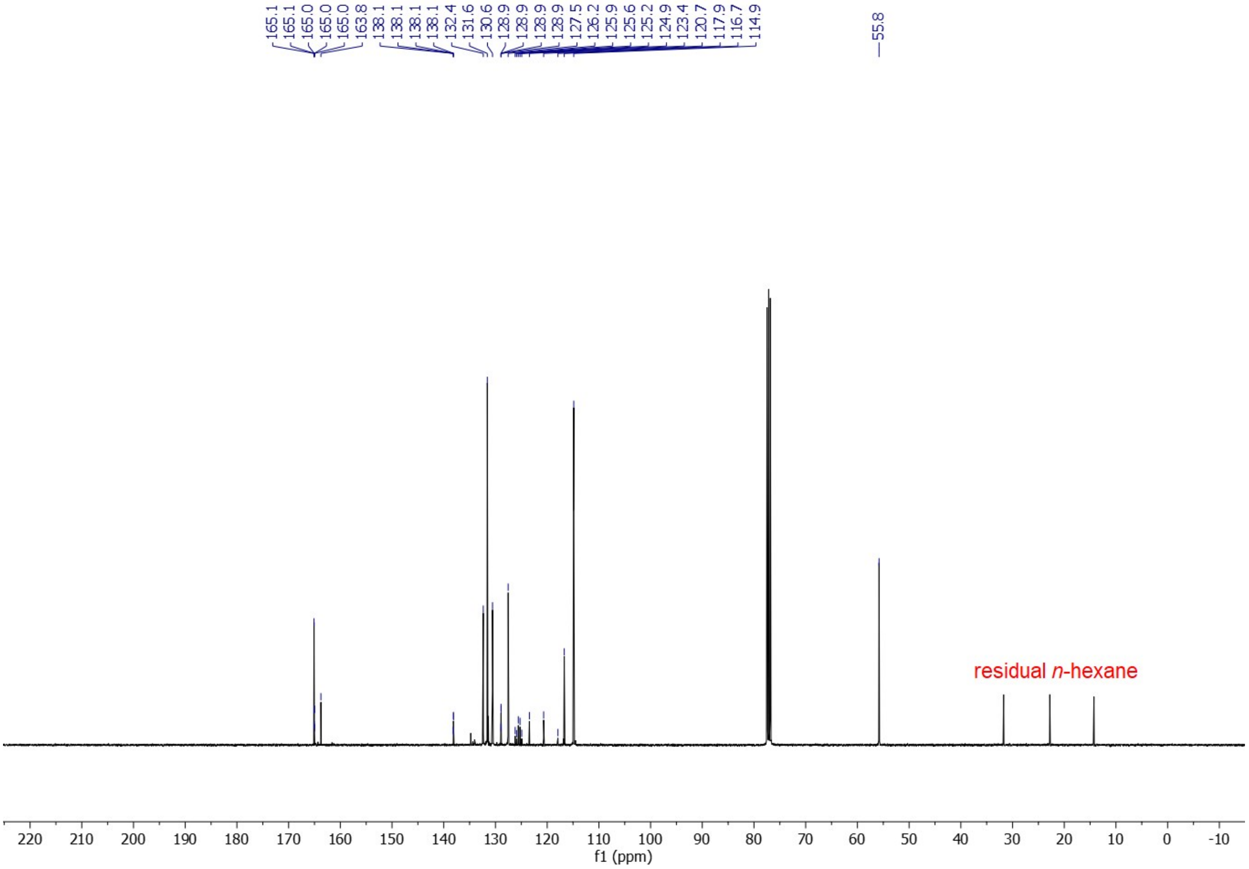

165.1
165.1
165.0
165.0
165.0
163.8
138.1
138.1
138.1
138.1
132.4
131.6
130.6
128.9
128.9
128.9
128.9
127.5
126.2
125.9
125.6
125.2
124.9
123.4
120.7
117.9
116.7
114.9
55.8
residual *n*-hexane
220 210 200 190 180 170 160 150 140 130 120 110 100 90 80 70 60 50 40 30 20 10 0 -10
f1 (ppm)

**(*Z*)-2-(4-methoxyphenyl)-4-(2,2,2-trifluoro-1-(1-tosyl-1H-indol-3-yl)ethylidene)oxazol-5(4H)-one (1l).**

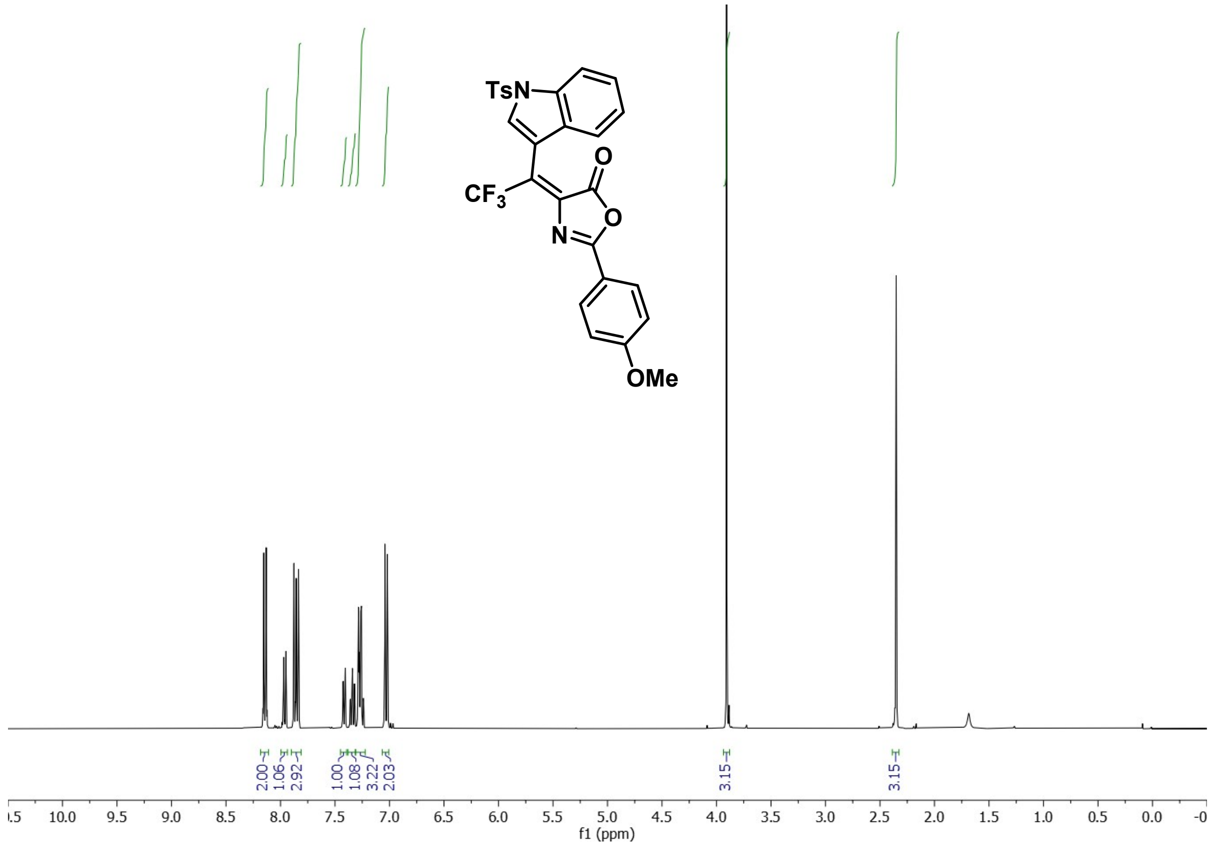

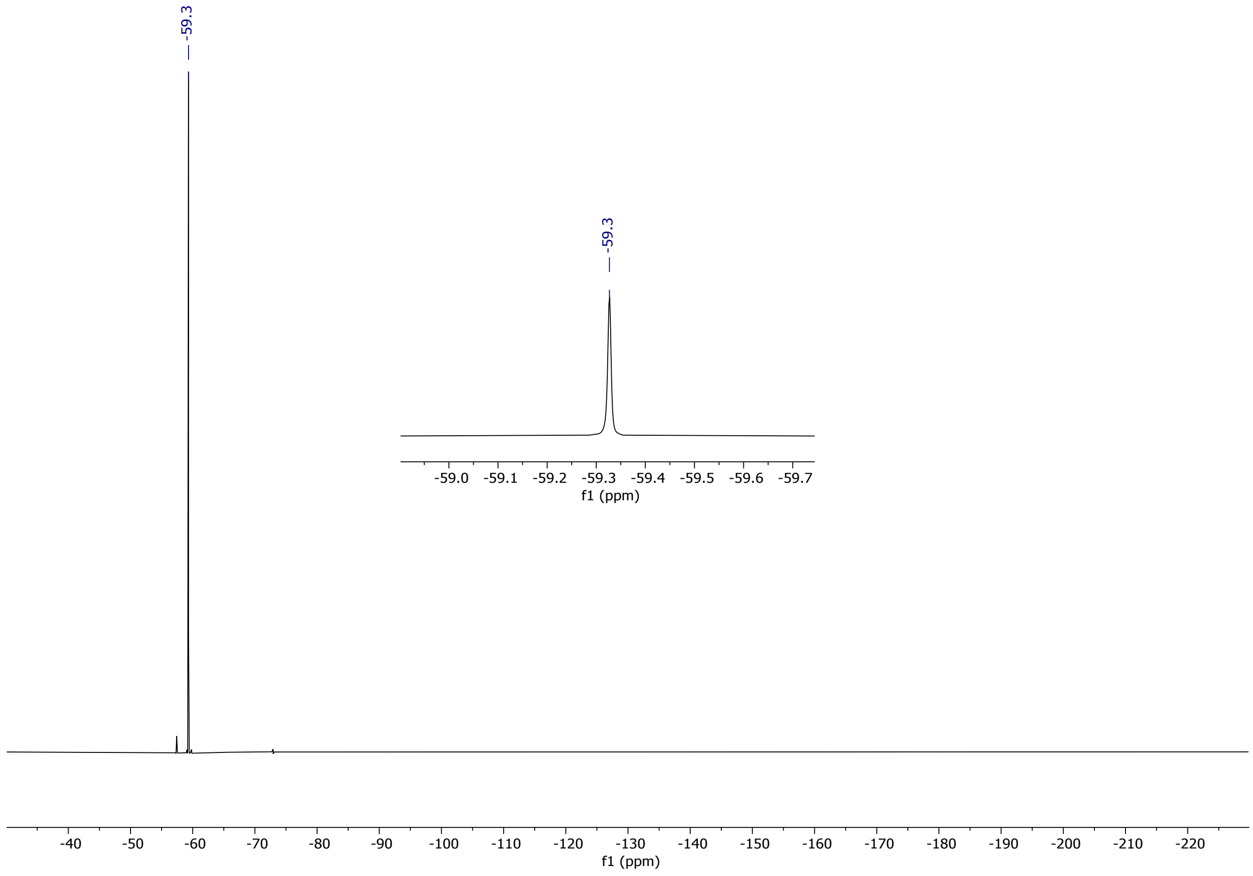
-59.3
-59.3
-59.0 -59.1 -59.2 -59.3 -59.4 -59.5 -59.6 -59.7
f1 (ppm)
-40 -50 -60 -70 -80 -90 -100 -110 -120 -130 -140 -150 -160 -170 -180 -190 -200 -210 -220
f1 (ppm)

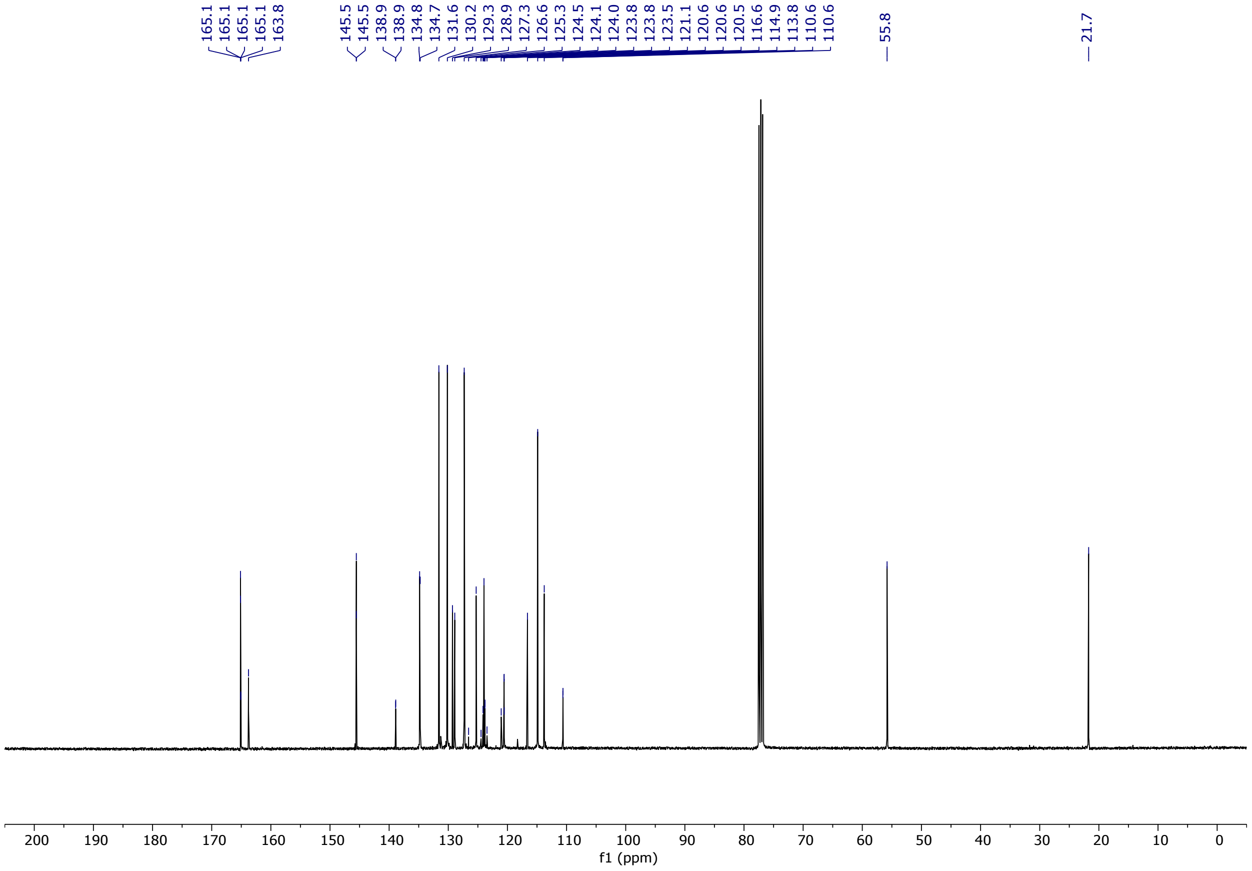
165.1
165.1
165.1
165.1
163.8
145.5
145.5
138.9
138.9
134.8
134.7
131.6
130.2
129.3
128.9
127.3
126.6
125.3
124.5
124.1
124.0
123.8
123.8
123.5
121.1
120.6
120.6
120.5
116.6
114.9
113.8
110.6
110.6
55.8
21.7
200 190 180 170 160 150 140 130 120 110 100 90 80 70 60 50 40 30 20 10 0
f1 (ppm)

**(*Z*)-4-(2,2,3,3,4,4,4-heptafluoro-1-phenylbutylidene)-2-(4-methoxyphenyl)oxazol-5(4*H*)-one (1m).**

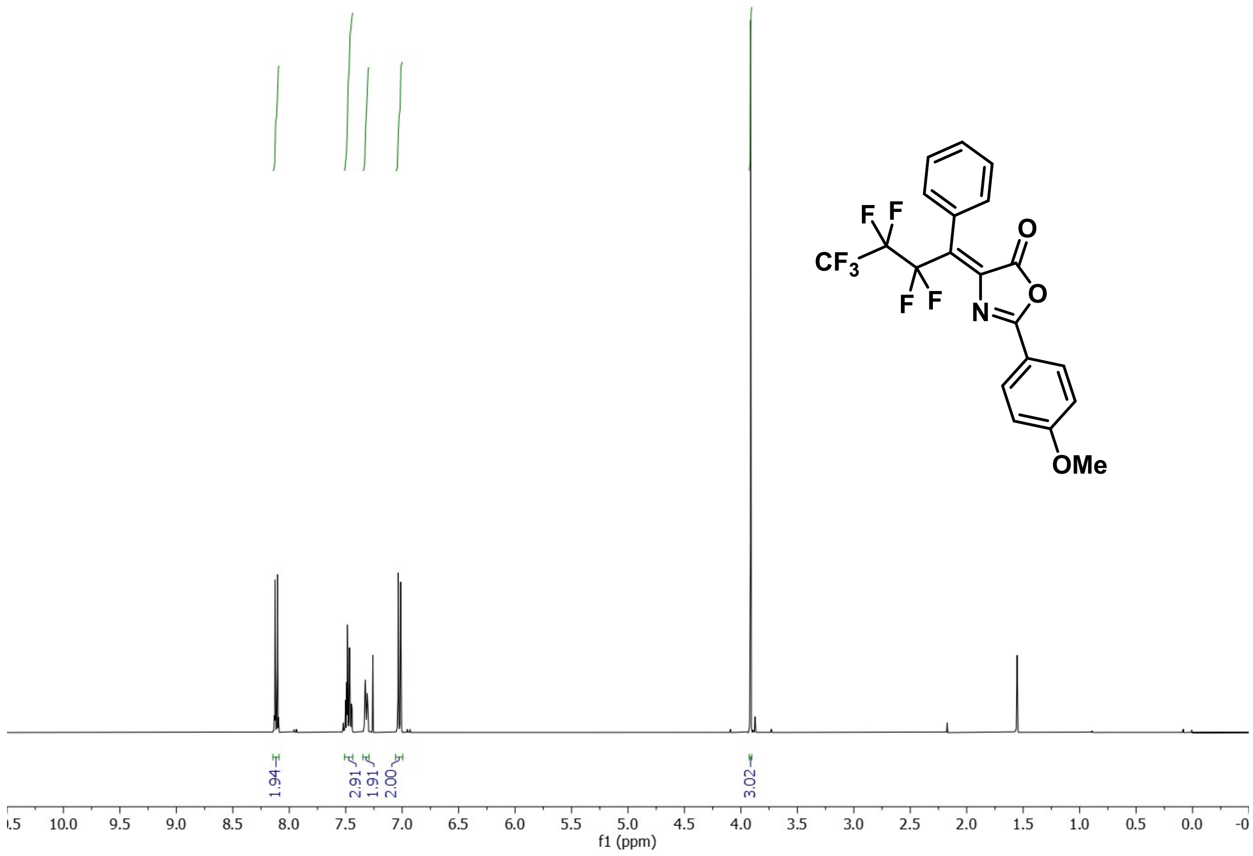

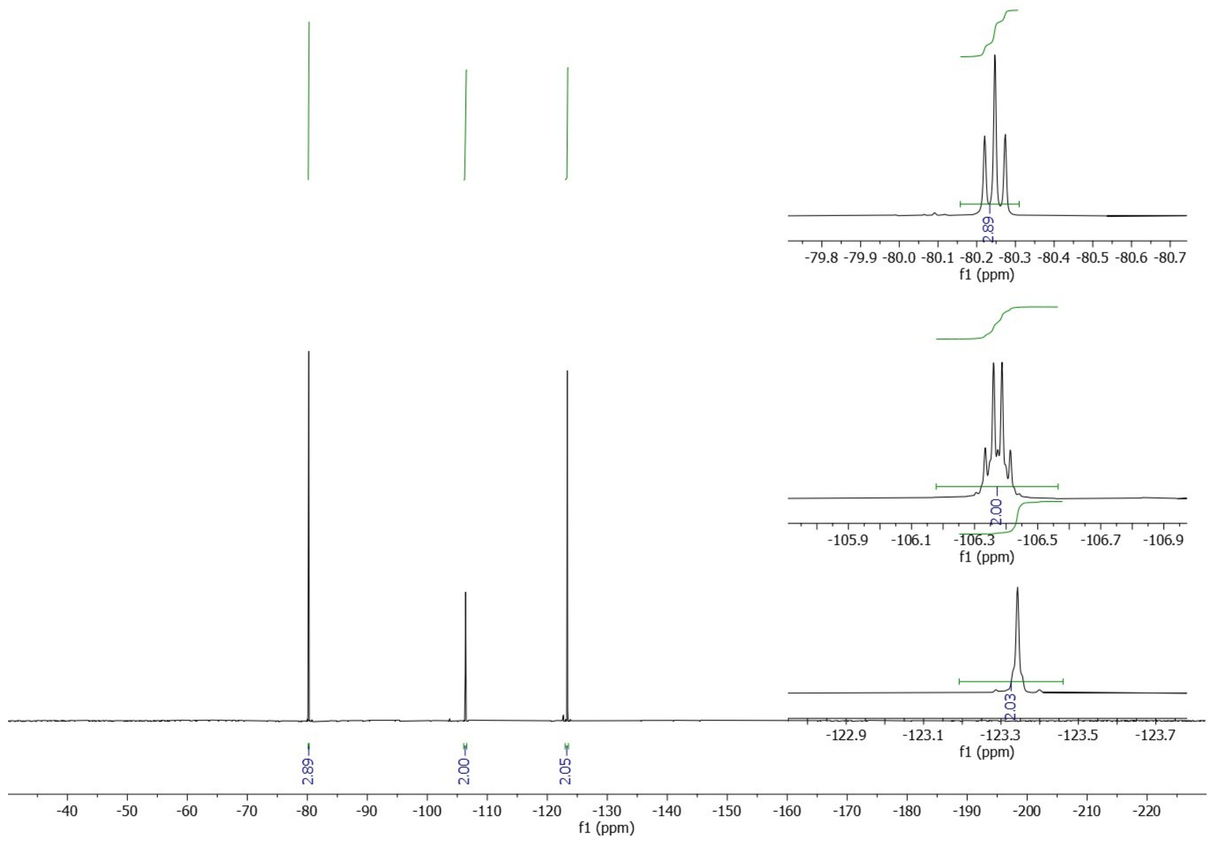
2.89
2.00
2.05
f1 (ppm)
-79.8 -79.9 -80.0 -80.1 -80.2 -80.3 -80.4 -80.5 -80.6 -80.7
-105.9 -106.1 -106.3 -106.5 -106.7 -106.9
2.03
-122.9 -123.1 -123.3 -123.5 -123.7

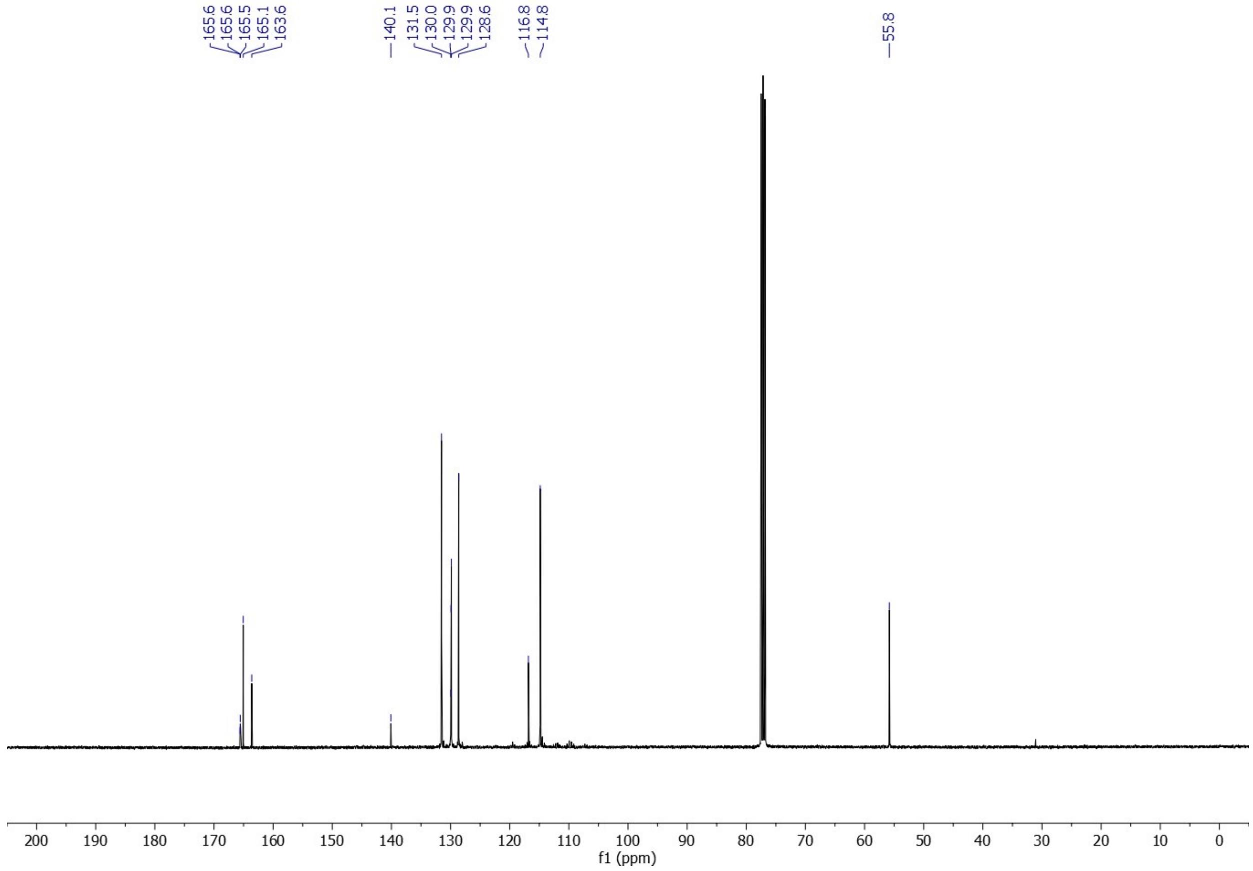
165.6
165.6
165.5
165.1
163.6
140.1
131.5
130.0
129.9
129.9
128.6
116.8
114.8
55.8
f1 (ppm)

**(*Z*)-4-(1-Cyclohexyl-2,2,2-trifluoroethylidene)-2-(4-methoxyphenyl)oxazol-5(4*H*)-one (1n).**

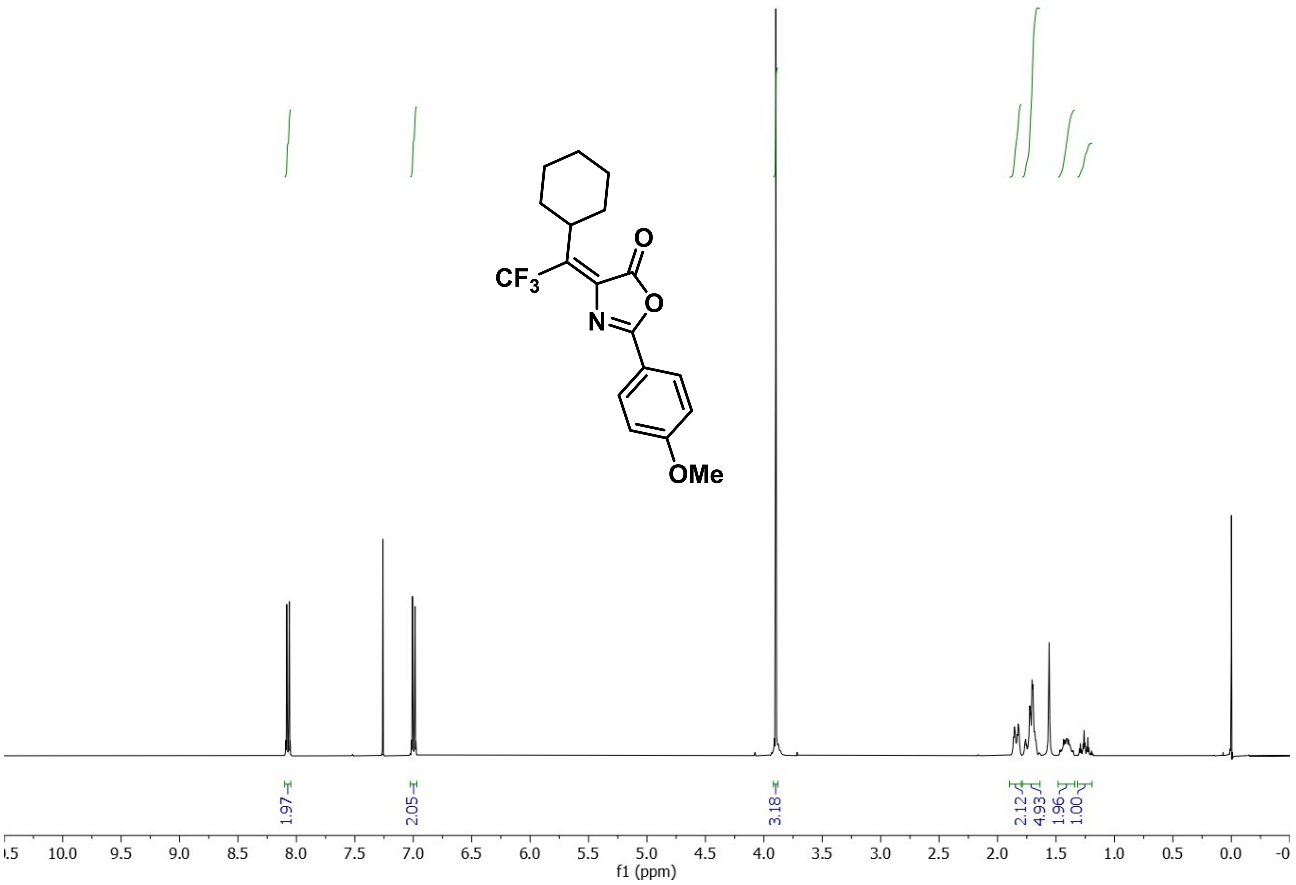

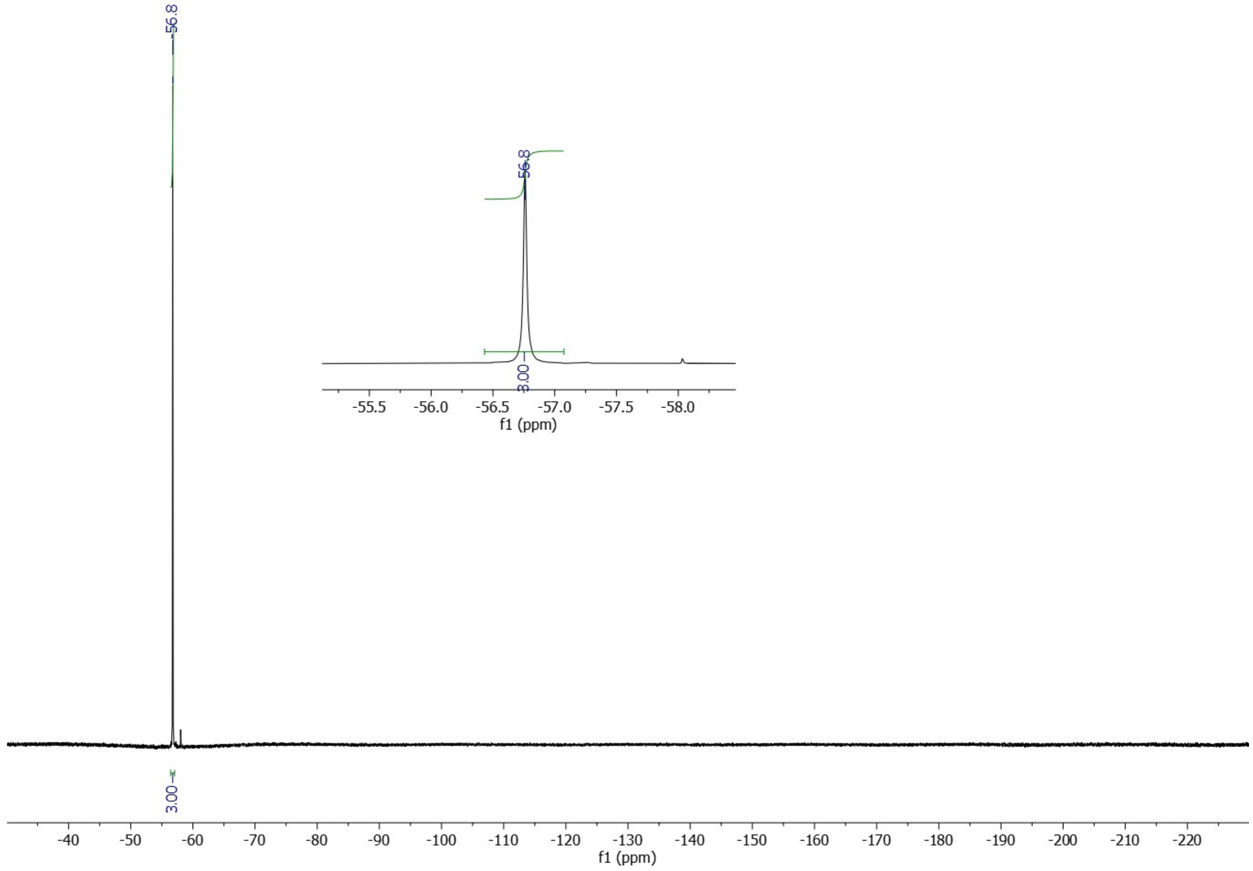
-56.8
3.00
f1 (ppm)

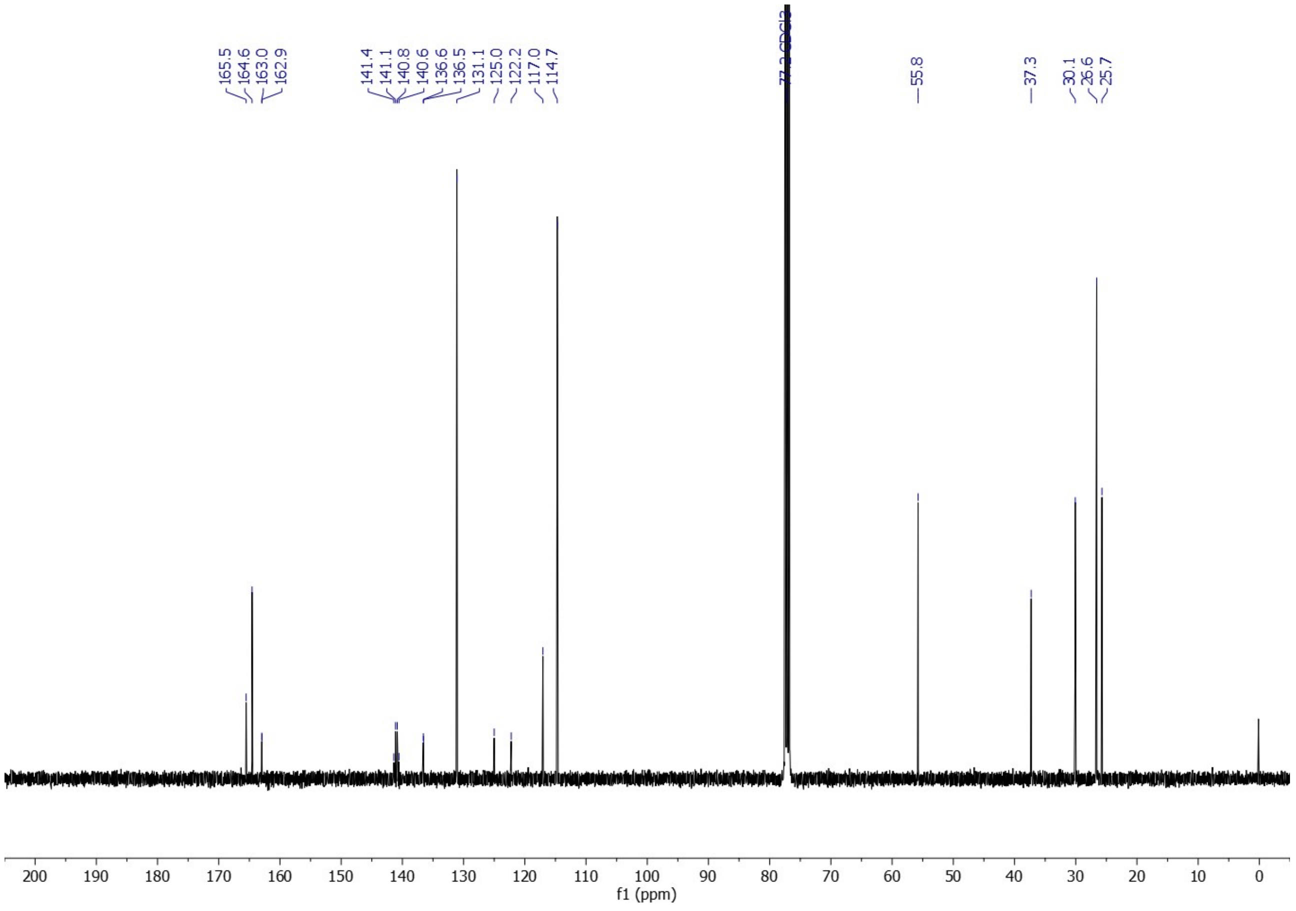
165.5
164.6
163.0
162.9
141.4
141.1
140.8
140.6
136.6
136.5
131.1
125.0
122.2
117.0
114.7
55.8
37.3
30.1
26.6
25.7
f1 (ppm)

**(*Z*)-2-(4-Methoxyphenyl)-4-(1,1,1-trifluorobutan-2-ylidene)oxazol-5(4H)-one (1o).**

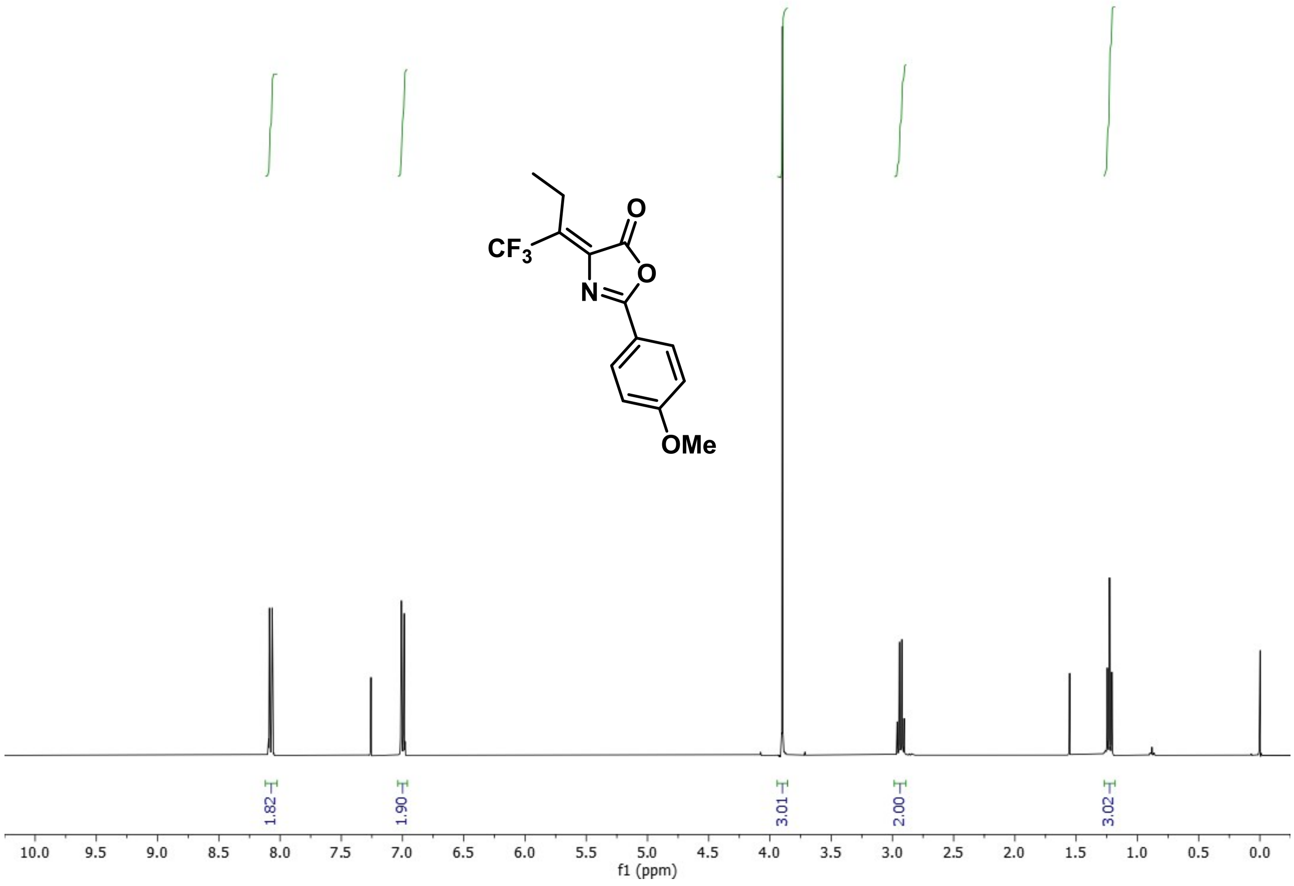

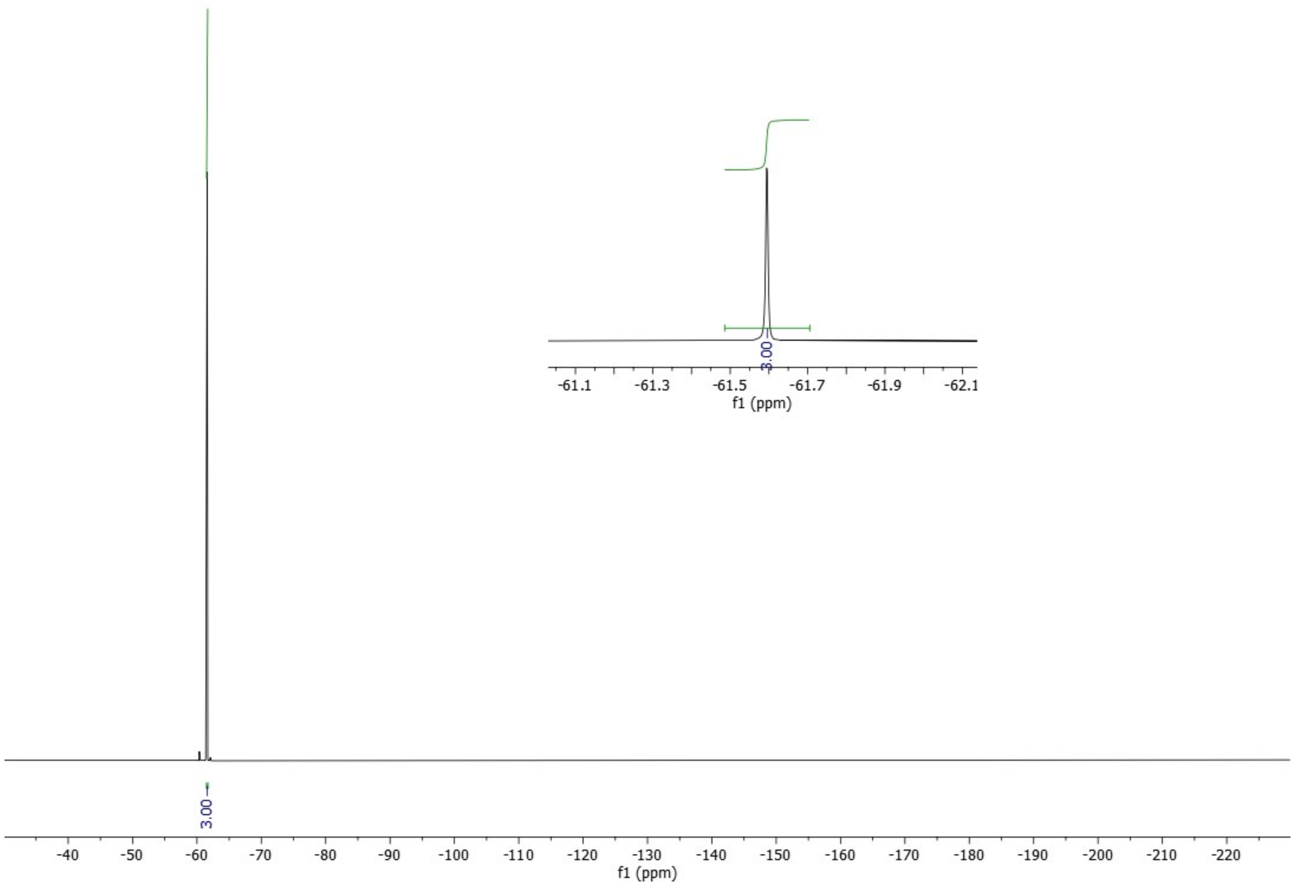
3.00
-61.1
-61.3
-61.5
-61.7
-61.9
-62.1
f1 (ppm)
3.00
-40
-50
-60
-70
-80
-90
-100
-110
-120
-130
-140
-150
-160
-170
-180
-190
-200
-210
-220
f1 (ppm)

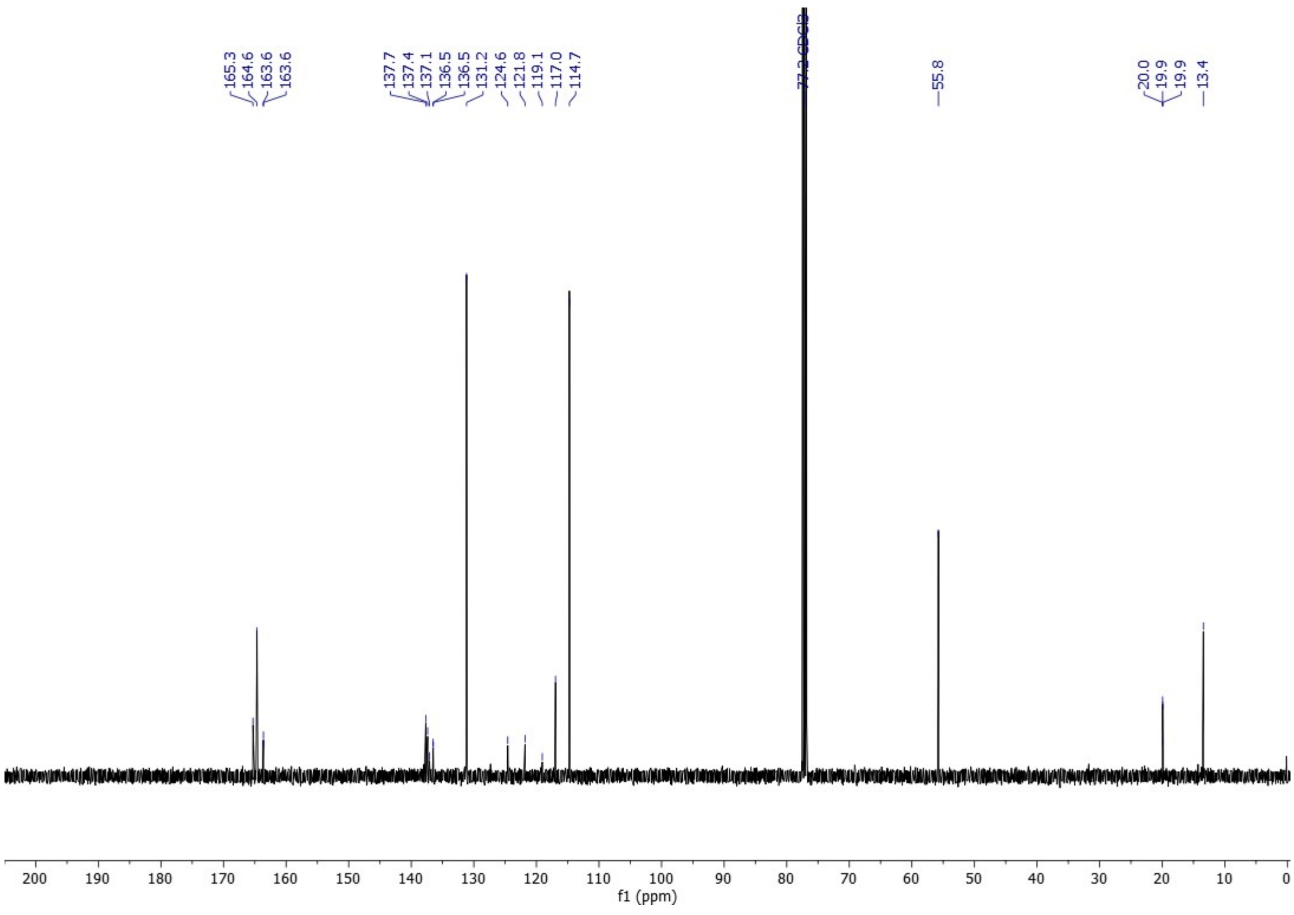
165.3
164.6
163.6
163.6
137.7
137.4
137.1
136.5
131.2
124.6
121.8
119.1
117.0
114.7
77.0 CDCl3
55.8
20.0
19.9
19.9
13.4
200
190
180
170
160
150
140
130
120
110
100
90
80
70
60
50
40
30
20
10
0
f1 (ppm)

**(*Z*)-2-(4-Methoxyphenyl)-4-(1,1,1-trifluoropropan-2-ylidene)oxazol-5(4*H*)-one (1p).**

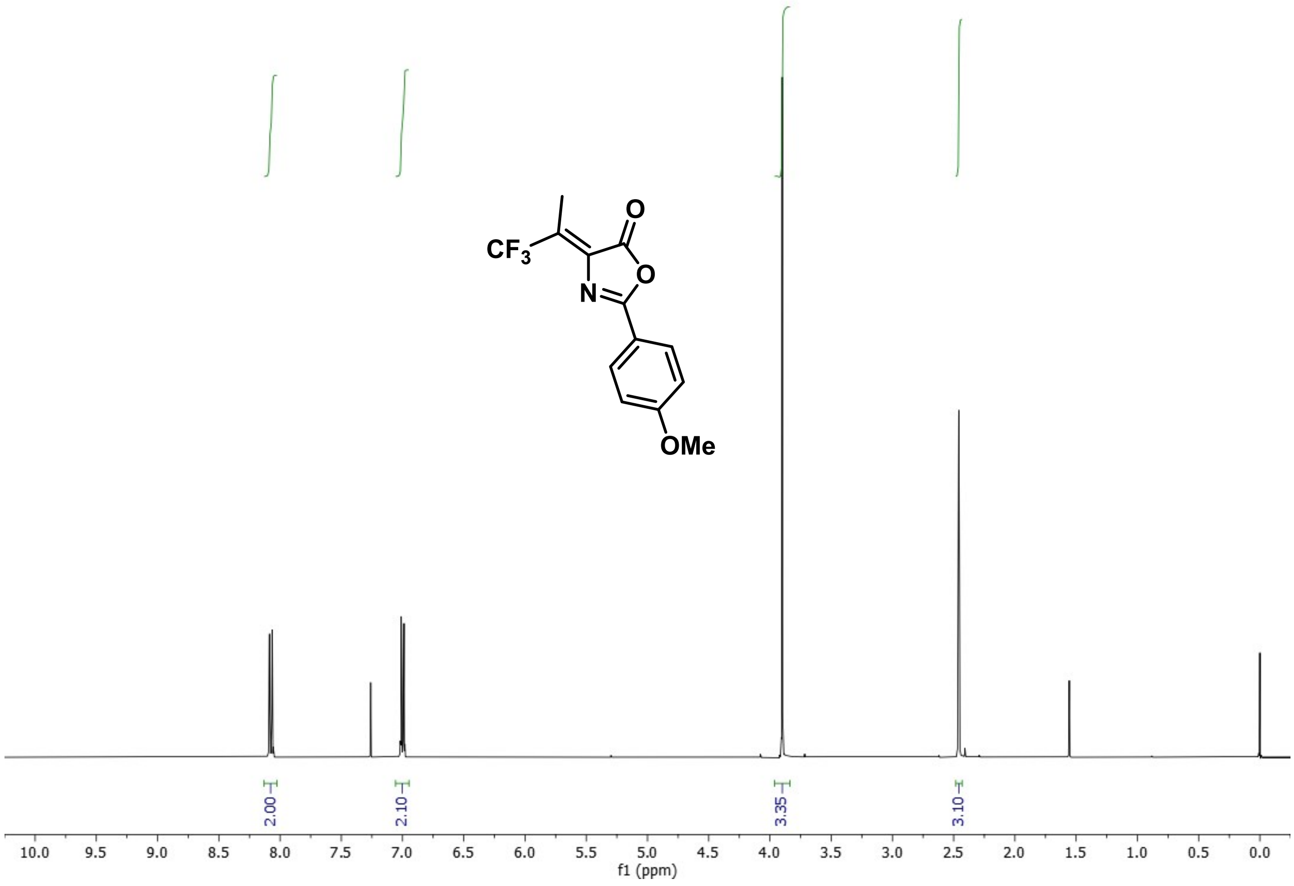

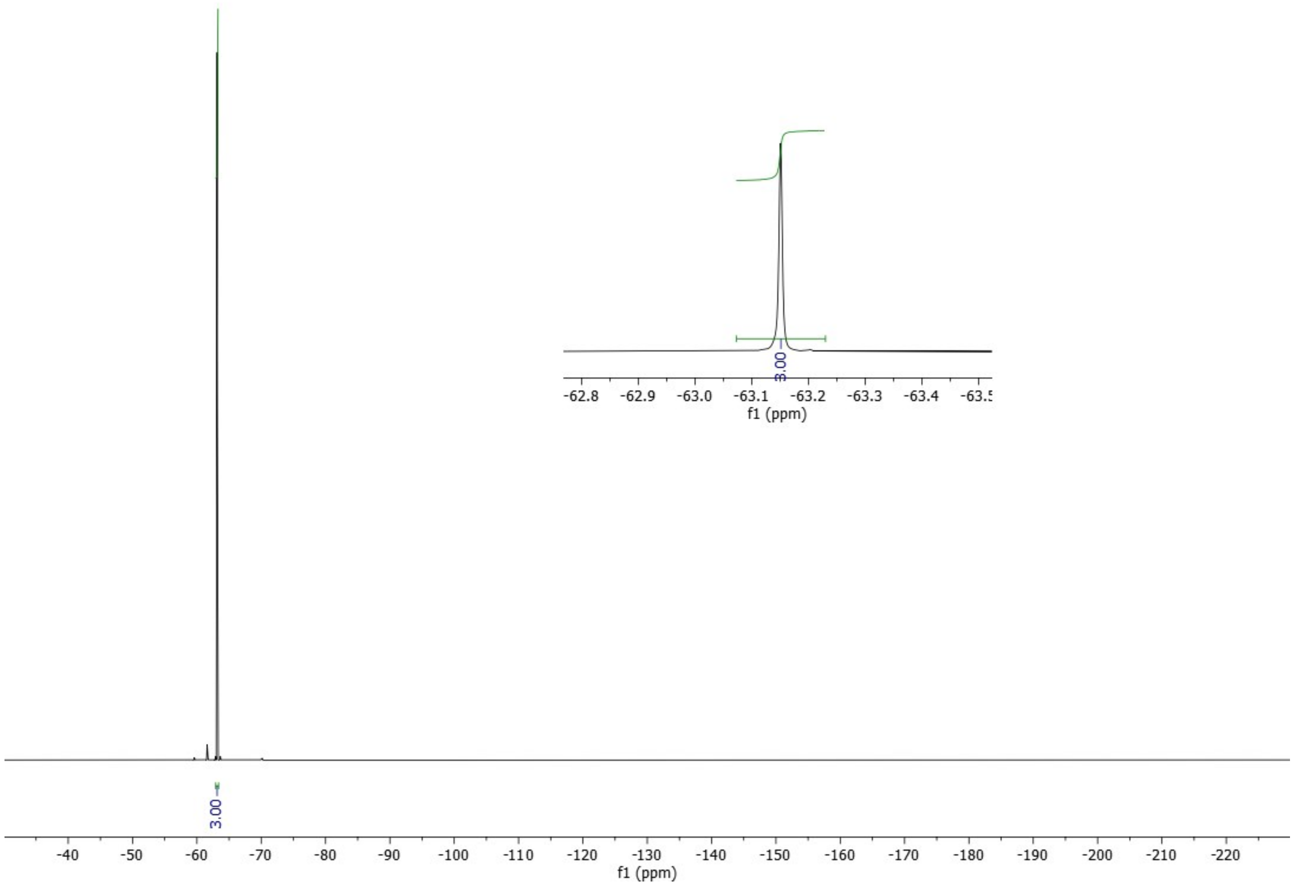
3.00
-62.8 -62.9 -63.0 -63.1 -63.2 -63.3 -63.4
f1 (ppm)
3.00
-40 -50 -60 -70 -80 -90 -100 -110 -120 -130 -140 -150 -160 -170 -180 -190 -200 -210 -220
f1 (ppm)

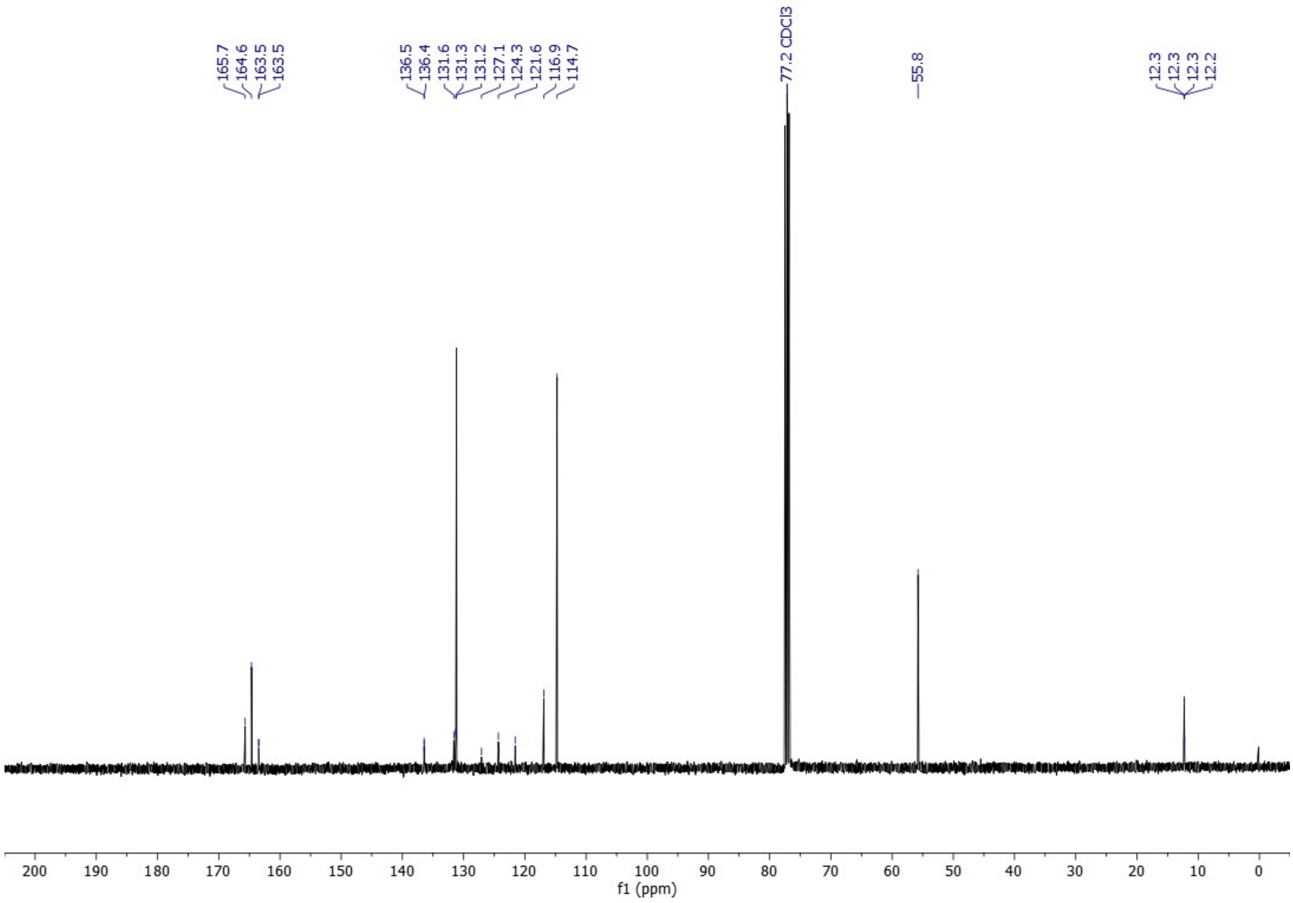
165.7
164.6
163.5
163.5
136.5
136.4
131.6
131.3
131.2
127.1
124.3
121.6
116.9
114.7
77.2 CDCl3
55.8
12.3
12.3
12.3
12.2
200 190 180 170 160 150 140 130 120 110 100 90 80 70 60 50 40 30 20 10 0
f1 (ppm)

## Synthesis[19] and characterization of 4-(trifluoromethyl)phenyl thiourea catalysts 2k and 2p.

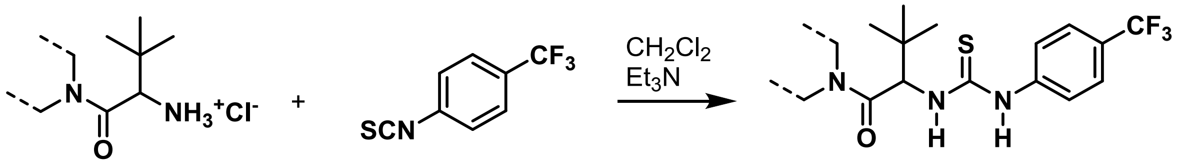

2

**General procedure**: To a stirred solution of crude α-amino amide hydrochloride (0.50 mmol) in $CH_2Cl_2$ (2.5 mL), $Et_3N$ (1.5 mmol) was added, followed by 4-trifluoromethylisothiocyanate (0.55 mmol). The resulting mixture was stirred overnight at RT, then the solvent was removed by rotary evaporation and the residue purified by chromatography on silica gel.

**(*S*)-*N*-Benzyl-*N*,3,3-trimethyl-2-(3-(4-(trifluoromethyl)phenyl)thioureido)butanamide (2k).**

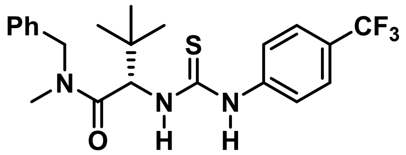

Following the general procedure (eluent for chromatography: $CH_2Cl_2$ then $CH_2Cl_2$ / $Et_2O$ 90:10), the title compound was obtained as a white solid in 90% yield. **$[\alpha]_D^{25}$** = +13 (c = 0.115 in $CHCl_3$). **$^1$H NMR** (400 MHz, $CDCl_3$) [two rotamers (major and minor) in 3.4 : 1 ratio] δ = 8.82 (br s, $1H_{maj}$, $1H_{min}$), 7.60-7.42 (m, $5H_{maj}$, $5H_{min}$), 7.39-7.17 (m, $5H_{maj}$, $5H_{min}$), 5.97 (d, *J* = 9.5 Hz, $1H_{min}$), 5.66 (d, *J* = 9.5 Hz, $1H_{maj}$), 5.17 (d, *J* = 14.9 Hz, $1H_{min}$), 4.81 (d, *J* = 14.5 Hz, $1H_{maj}$), 4.39 (d, *J* = 14.9 Hz, $1H_{min}$), 4.33 (d, *J* = 14.7 Hz, $1H_{maj}$), 3.20 (s, $3H_{maj}$), 2.79 (s, $3H_{min}$), 1.082 (s, $9H_{min}$), 1.078 (s, $9H_{maj}$). **$^{19}$F NMR** (376 MHz, $CDCl_3$) δ = -62.37 (s, 3F). **$^{13}$C NMR** (101 MHz, $CDCl_3$) [two rotamers, major and minor, signals tentatively assigned based on their intensity] δ = 180.92 (maj), 180.89 (min), 172.3 (min), 172.2 (maj), 140.7 (br s, min), 140.6 (br s, maj), 136.2 (maj), 135.4 (min), 128.8 (min), 128.7 (maj), 128.3 (min), 128.2 (min), 128.0 (maj), 127.62 (maj), 127.60 (q, *J* = 32.8 Hz, maj), 127.59 (q, *J* = 33.8 Hz, min), 126.6 (q, *J* = 3.7 Hz, maj), 126.5 (q, *J* = 3.7 Hz, min), 124.1 (min), 123.90 (q, *J* = 271.6 Hz, maj), 123.88 (q, *J* = 273.3 Hz, min), 123.8 (maj), 60.9 (maj), 60.7 (min), 54.7 (min), 51.6 (maj), 36.7 (min), 36.3 (maj), 36.2 (maj), 33.3 (min), 27.1 (min), 27.0 (maj). **HRMS**: calculated for $C_{22}H_{25}F_3N_3OS$ [M - H$^+$] = 436.1670; found: 436.1676.

**1-((*S*)-3,3-Dimethyl-1-oxo-1-((*R*)-2-phenylpyrrolidin-1-yl)butan-2-yl)-3-(4-(trifluoromethyl)phenyl)thiourea (2p).**

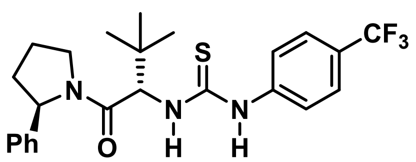

Following the general procedure (eluent for chromatography: n-hexane / EtOAc from 9:1 to 3:1), the title compound was obtained as a white solid in 52% yield. **[α]$_D$$^{25}$** = +34 (c = 0.165 in $CHCl_3$). **$^1$H NMR** (400 MHz, $CDCl_3$) [two rotamers (major and minor) in 2.4 : 1 ratio] δ = 8.93 (br s, $1H_{maj}$), 8.78 (br s, $1H_{min}$), 7.61-7.56 (m, $2H_{min}$), 7.51-7.45 (m, $2H_{maj}$), 7.43-7.28 (m, $4H_{maj}$, $4H_{min}$), 7.23-7.17 (m, $2H_{maj}$), 7.16-7.03 (m, $2H_{maj}$, $4H_{min}$), 5.91 (dd, *J* = 7.9, 1.8 Hz, $1H_{min}$), 5.45 (d, *J* = 8.6 Hz, $1H_{maj}$), 5.20 (d, *J* = 9.8 Hz, $1H_{min}$), 5.15 (dd, *J* = 7.9, 1.4 Hz, $1H_{maj}$), 4.50 (ddd, *J* = 10.6, 8.1, 3.4 Hz, $1H_{maj}$), 3.79 (td, *J* = 9.4, 7.4 Hz, $1H_{maj}$), 3.71-3.56 (m, $2H_{min}$), 2.42-2.30 (m, $1H_{min}$), 2.30-2.20 (m, $1H_{maj}$), 2.15-1.81 (m, $3H_{maj}$, $3H_{min}$), 1.09 (s, $9H_{maj}$), 0.62 (s, $9H_{min}$). **$^{19}$F NMR** (376 MHz, $CDCl_3$) δ = -62.34 (s, 3F). **$^{13}$C NMR** (101 MHz, $CDCl_3$) [two rotamers, major and minor, signals tentatively assigned based on their intensity] δ = 180.7 (maj), 180.2 (min), 171.7 (min), 170.0 (maj), 143.8 (min), 142.2 (maj), 140.7 (br s, maj), 140.5 (br s, min), 128.6 (min), 128.3 (maj), 127.60 (q, *J* = 32.4 Hz, min), 127.59 (min), 127.2 (q, *J* = 32.5 Hz, maj), 126.7 (maj), 126.63 (q, *J* = 3.6 Hz, min), 126.60 (min), 126.4 (q, *J* = 3.6 Hz, maj), 125.3 (maj), 123. 90 (q, *J* = 270.9 Hz, maj), 123.85 (q, *J* = 270.9 Hz, min), 123.7 (min), 123.3 (maj), 63.1 (maj), 62.1 (min), 61.9 (min), 61.2 (maj), 48.6 (maj), 47.4 (min), 35.7 (maj), 35.51 (min), 35.46 (maj), 34.4 (min), 26.9 (maj), 26.6 (min), 23.0 (maj), 21.7 (min). **HRMS**: calculated for $C_{24}H_{27}F_3N_3OS$ [M - H$^+$] = 462.1827; found: 462.1834.

**1-((*R*)-3,3-Dimethyl-1-oxo-1-((*S*)-2-phenylpyrrolidin-1-yl)butan-2-yl)-3-(4-(trifluoromethyl)phenyl)thiourea (*ent*-2p).**

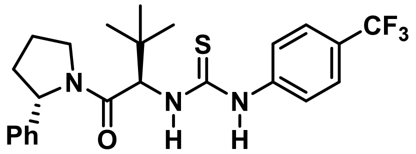

Following the general procedure (eluent for chromatography: n-hexane / EtOAc from 9:1 to 3:1), the title compound was obtained as a white solid in 56% yield. Spectral data were identical to compound **2p**. **[α]$_D$$^{25}$** = -33 (c = 0.10 in $CHCl_3$).

## Copies of $^{1}H$, $^{19}F$, and $^{13}C$ NMR spectra of catalysts 2k, 2p (in $CDCl_3$)

**(*S*)-*N*-Benzyl-*N*,3,3-trimethyl-2-(3-(4-(trifluoromethyl)phenyl)thioureido)butanamide (2k).**

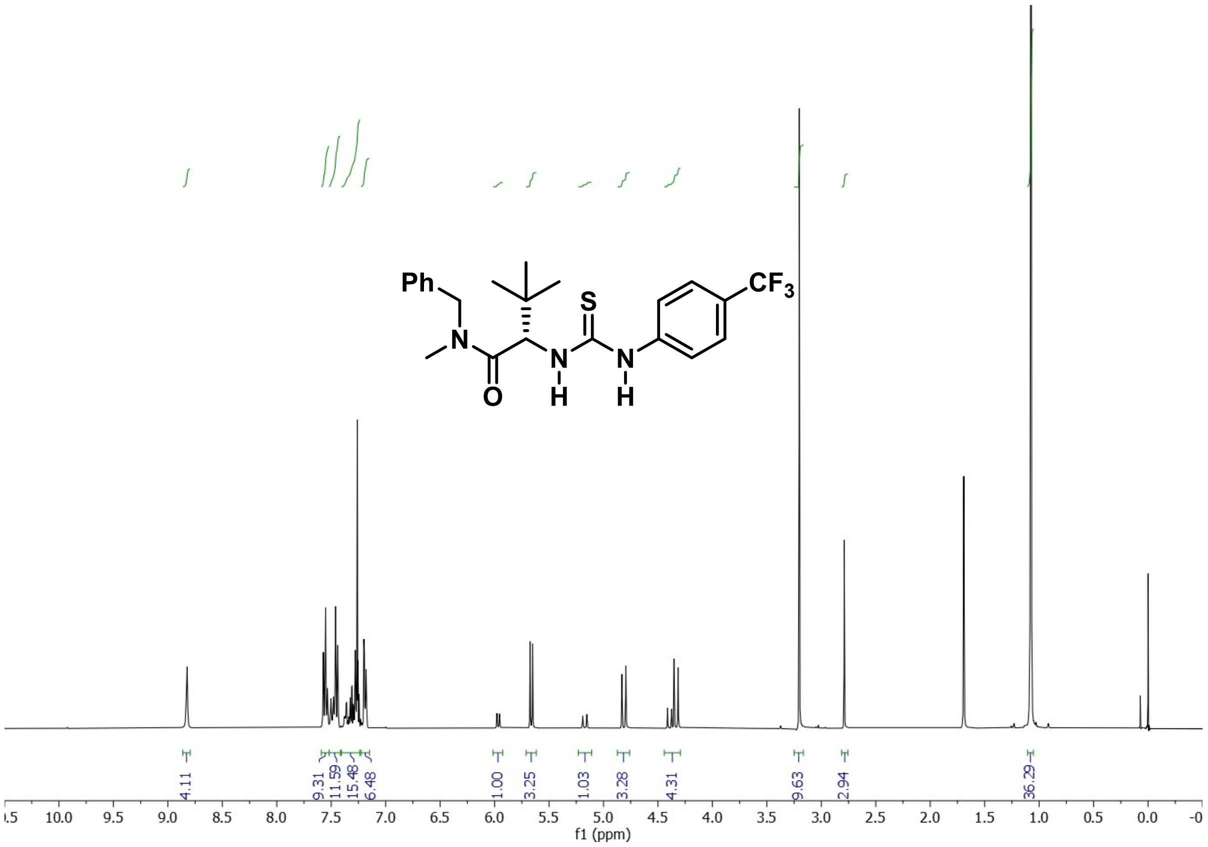


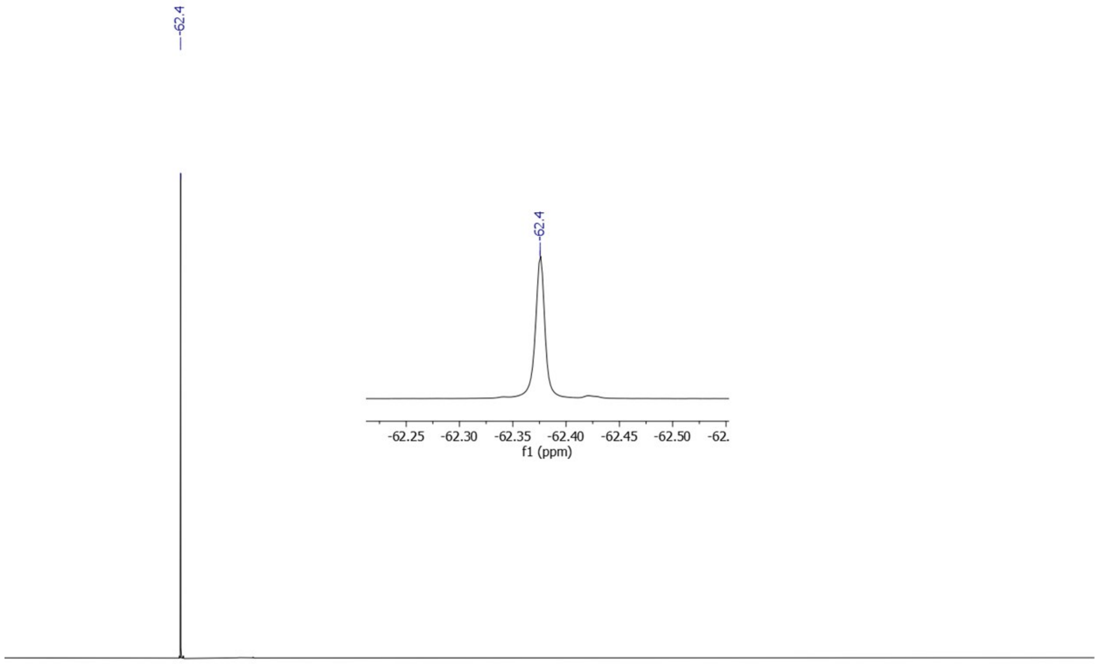

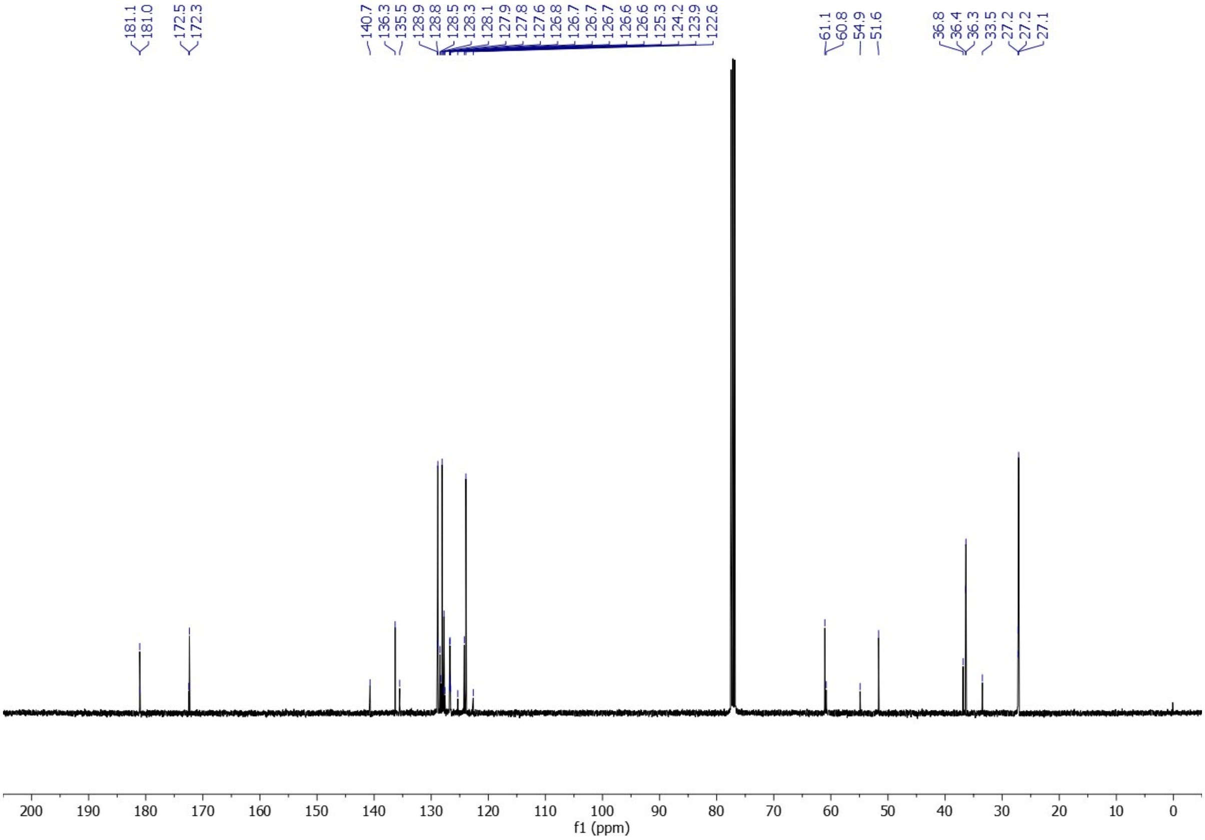

181.1
181.0
172.5
172.3
140.7
136.3
135.5
128.9
128.8
128.5
128.3
128.1
127.9
127.8
127.6
126.8
126.7
126.7
126.7
126.6
126.6
125.3
124.2
123.9
122.6
61.1
60.8
54.9
51.6
36.8
36.4
36.3
33.5
27.2
27.2
27.1
200
190
180
170
160
150
140
130
120
110
100
90
80
70
60
50
40
30
20
10
0
f1 (ppm)

**1-((*S*)-3,3-Dimethyl-1-oxo-1-((*R*)-2-phenylpyrrolidin-1-yl)butan-2-yl)-3-(4-(trifluoromethyl)phenyl)thiourea (2p).**

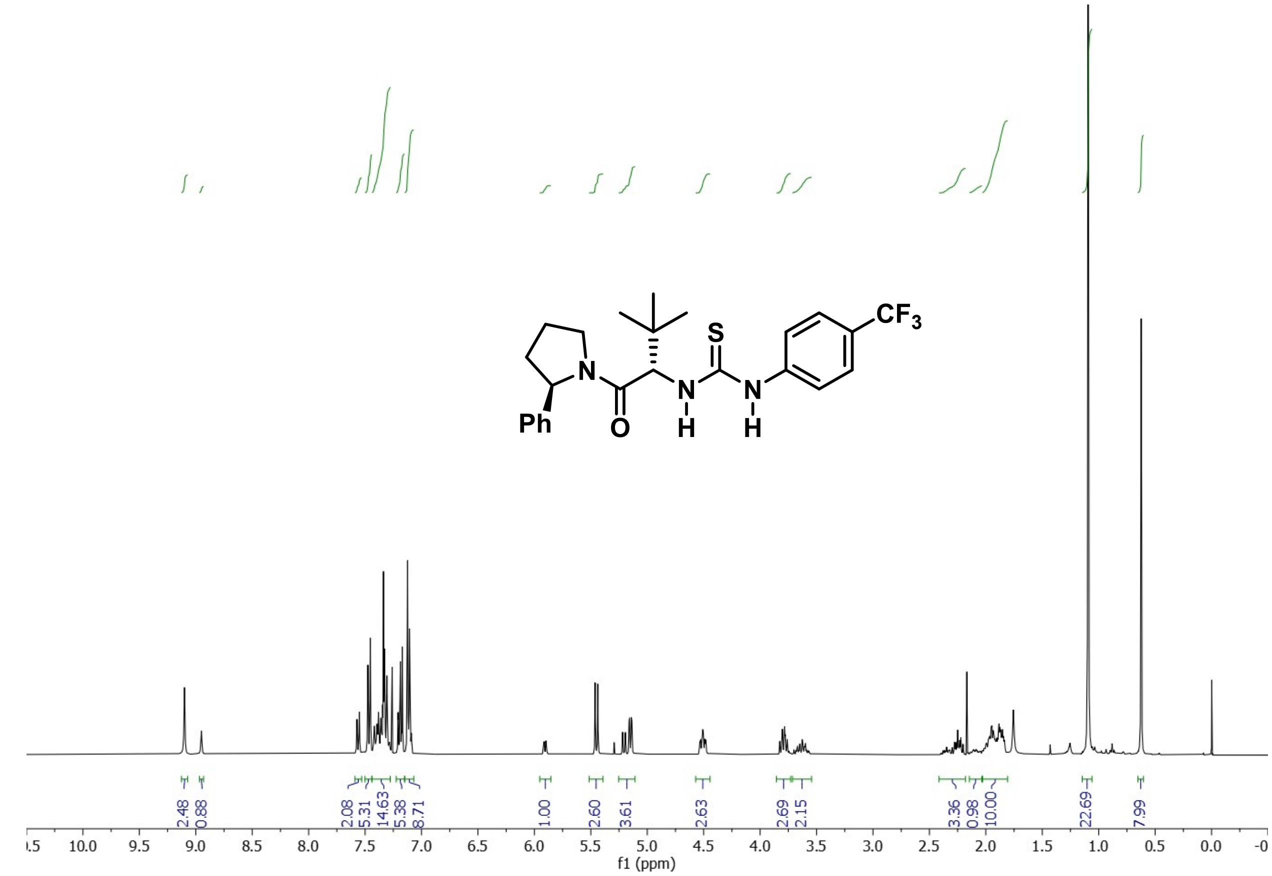


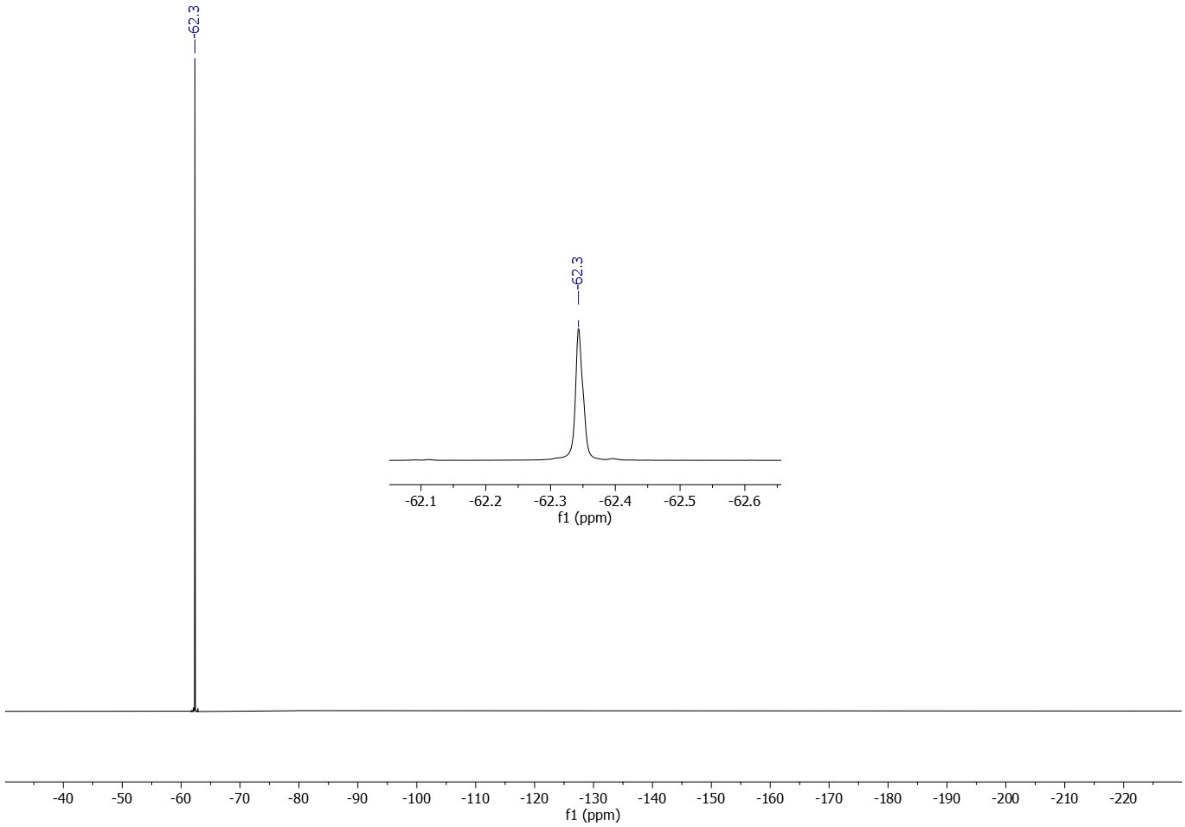

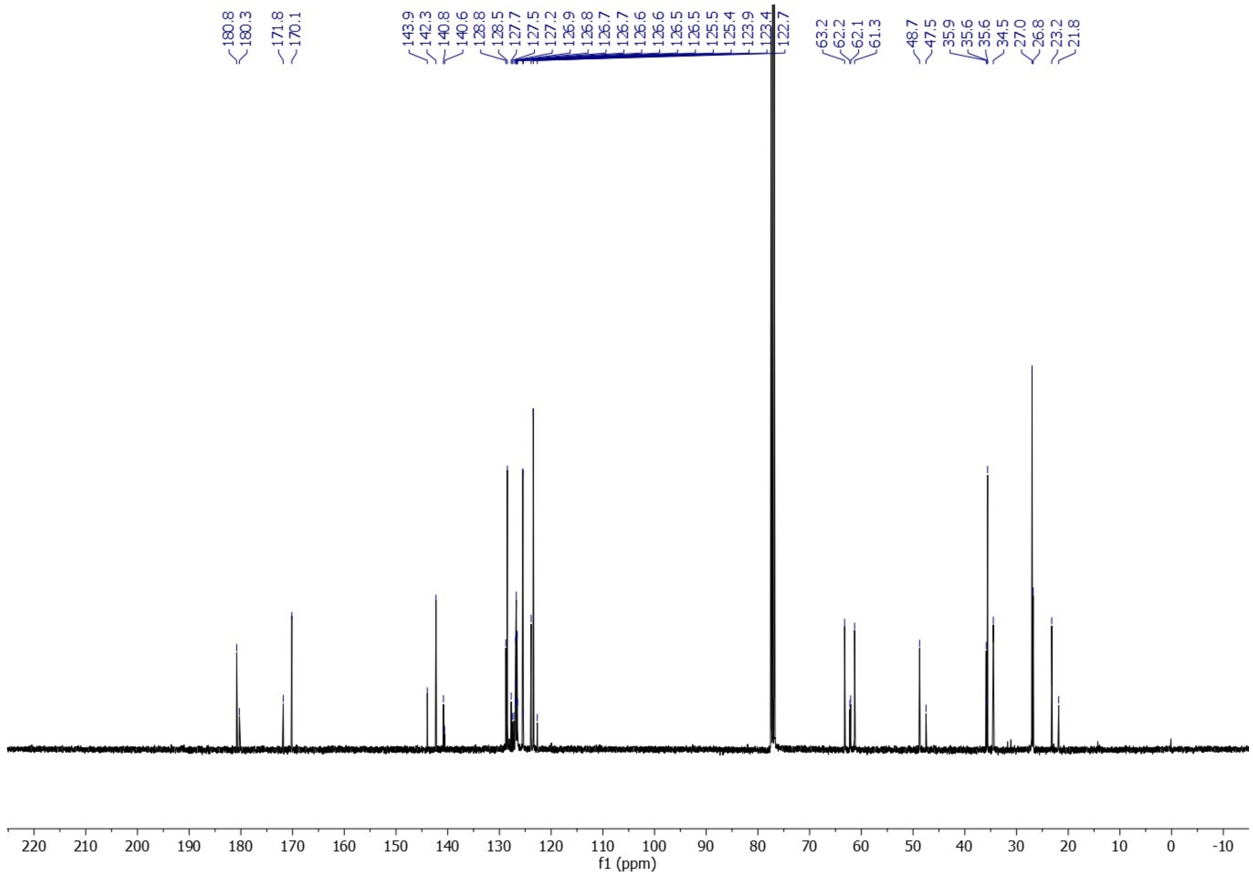

180.8
180.3
171.8
170.1
143.9
142.3
140.8
140.6
128.8
128.5
127.7
127.5
127.2
126.9
126.8
126.7
126.7
126.6
126.6
126.5
126.5
125.5
125.4
123.9
123.4
122.7
63.2
62.2
62.1
61.3
48.7
47.5
35.9
35.6
35.6
34.5
27.0
26.8
23.2
21.8
220 210 200 190 180 170 160 150 140 130 120 110 100 90 80 70 60 50 40 30 20 10 0 -10
f1 (ppm)

## Synthesis[22] and characterization of dhQN and dhQD catalysts.

### Preparation of catalyst dhQD-2.

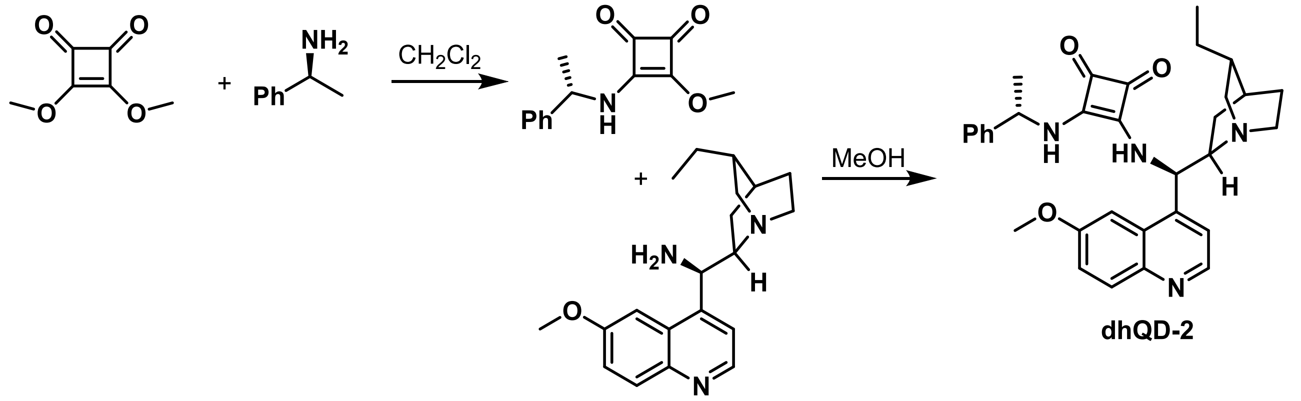


To a stirred solution of dimethylsquarate (3.46 mmol) in $CH_2Cl_2$ (4 mL), (*S*)-α-methylbenzylamine (3.63 mmol) was added, and the resulting mixture stirred for 72 h at RT. The reaction was filtered, and the filtrate washed with aqueous HCl (1 M), dried over $MgSO_4$, filtered and concentrated, giving intermediate monoamine squarate as a white solid. To a portion (0.67 mmol) of the thus obtained compound, dissolved in MeOH (5.6 mL), a solution of 9-amino-9-deoxy *epi*-dihydroquinidine (0.56 mmol) in MeOH (ca. 1.5 mL) was added, and the mixture stirred for 24 h at RT. The resulting precipitate was collected by Buchner filtration and washed with cold MeOH, affording the **dhQD-2** catalyst as a white solid in 70% yield. $[\alpha]_D^{25}$ = +134 ° (c = 0.11 in $CHCl_3$). **$^1$H NMR** (400 MHz, DMSO-$d_6$, 60 °C) δ = 8.78 (d, *J* = 4.6 Hz, 1H), 7.97 (d, *J* = 9.2 Hz, 1H), 7.81 (d, *J* = 2.5 Hz, 1H), 7.69 (d, *J* = 8.9 Hz, 1H), 7.63 (br s, 1H), 7.54 (d, *J* = 4.5 Hz, 1H), 7.43 (dd, *J* = 9.1, 2.6 Hz, 1H), 7.38-7.30 (m, 4H), 7.30-7.22 (m, 1H), 6.05 (t, *J* = 9.4 Hz, 1H), 5.14 (quint, *J* = 7.4 Hz, 1H), 3.94 (s, 3H), 3.27 (q, *J* = 9.4 Hz, 1H), 2.96-2.74 (m, 4H), 1.59-1.20 (m, 6H), 1.47 (d, *J* = 6.8 Hz, 3H), 1.06-0.96 (m, 1H), 0.93-0.89 (m, 1H), 0.83 (t, *J* = 6.8 Hz, 3H). **$^{13}$C NMR** (101 MHz, DMSO-$d_6$, 60 °C) δ = 183.0, 182.3, 167.5, 167.2, 158.3, 148.2, 144.8, 144.7, 143.8, 131.9, 129.1, 128.0, 127.9, 126.5, 122.3, 119.9, 102.1, 59.6, 56.0, 53.3 (br), 53.2, 49.5, 48.8, 37.4, 27.6, 26.2, 25.9, 25.5, 23.3, 12.1. **HRMS**: calculated for $C_{32}H_{37}N_4O_3$ [M + $H^+$] = 525.2866; found: 525.2868.

**Preparation of catalyst dhQN-2.**

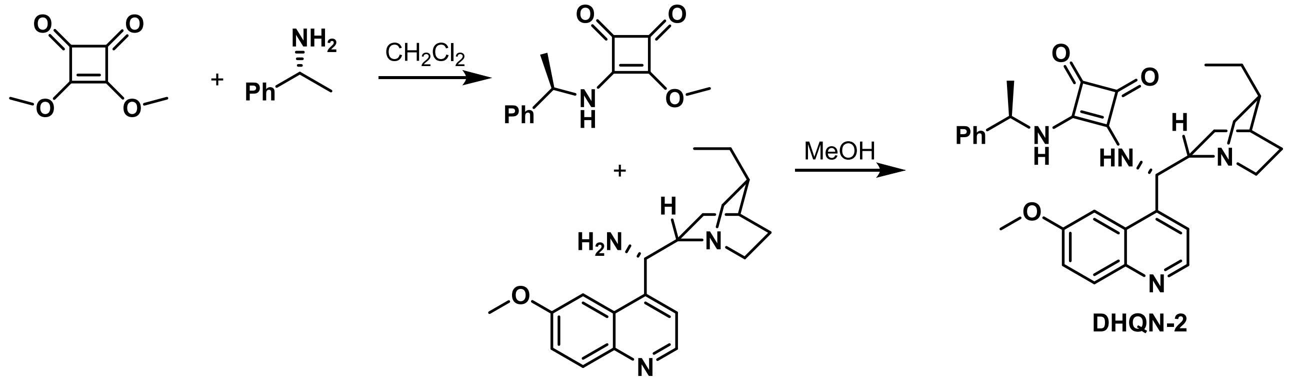


Following the procedure used for **dhQD-2**, but using (*R*)-α-methylbenzylamine and 9-amino-9-deoxy *epi*-dihydroquinine as starting compounds, catalyst **dhQN-2** was obtained in 46% yield as a white solid. $[\alpha]_D^{25}$ = -12 ° (c = 0.140 in $CHCl_3$). **$^1$H NMR** (400 MHz, DMSO-$d_6$, 60 °C) δ = 8.78-8.75 (m, 1H), 7.97-7.93 (m, 1H), 7.78 (br s, 1H), 7.68 (br d, *J* = 9.0 Hz, 1H), 7.63 (br s, 1H), 7.55-7.51 (m, 1H), 7.44-7.39 (m, 1H), 7.31 (br s, 4H), 7.28-7.21 (m, 1H), 5.93 (br t, *J* = 9.1 Hz, 1H), 5.13 (quint, *J* = 7.6 Hz, 1H), 3.93 (s, 3H), 3.36-3.20 (m, 2H), 3.14-3.09 (m, 1H, partially overlapped with residual $H_2O$ signal), 2.61-2.51 (m, 1H), 2.43-2.36 (m, 1H), 1.56-1.48 (m, 2H), 1.46 (d, *J* = 6.8 Hz, 3H), 1.43-1.23 (m, 5H), 0.79 (t, *J* = 6.9 Hz, 3H), 0.63-0.53 (m, 1H). **$^{13}$C NMR** (101 MHz, DMSO-$d_6$, 60 °C) δ = 183.0, 182.2, 167.4, 167.3, 158.3, 148.2, 144.8, 144.5, 143.8, 131.9, 129.1, 128.0, 127.9, 126.5, 122.2, 120.0, 102.4, 59.4, 57.8, 56.2, 54.2 (br), 53.3, 53.2, 37.4, 28.7, 27.3, 26.4, 25.6, 23.3, 12.3. **HRMS**: calculated for $C_{32}H_{37}N_4O_3$ [M + H$^+$] = 525.2866; found: 525.2862.

**Preparation of 3-amino-4-methoxycyclobut-3-ene-1,2-dione.**

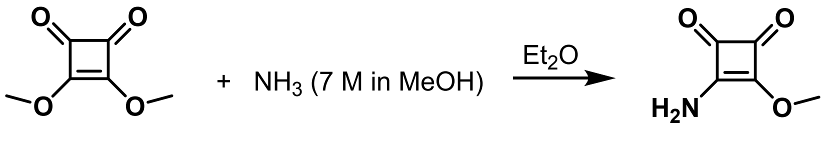


To a stirred, cooled (0 °C) suspension of dimethylsquarate (5.0 mmol) in $Et_2O$ (125 mL, untreated), a commercial solution of $NH_3$ in MeOH (7 M, 786 μL, 5.5 mmol) was added, resulting in the formation of a white fine precipitate. After stirring overnight at RT, the product was collected by Buchner filtration (65% yield, NMR data consistent with the literature[24]), and used in the next step without further purification.

[24] M. Lu, Q.-B. Lu and J. F. Honek, *Bioorg. Med. Chem. Lett.*, 2017, **27**, 282.

**Preparation of catalyst dhQD-10.**

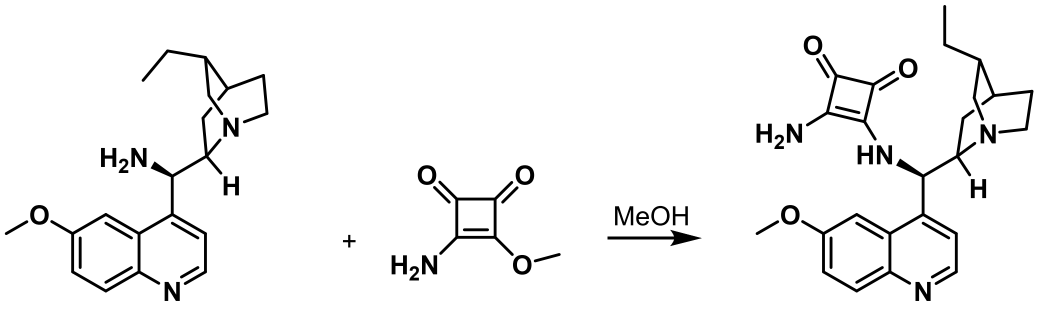

dhQD-10

A solution of 9-amino-9-deoxy *epi*-dihydroquinidine (0.85 mmol) in MeOH (ca. 1.5 mL) was added to a stirred suspension of 3-amino-4-methoxycyclobut-3-ene-1,2-dione (1.02 mmol) in MeOH (8.0 mL). After stirring the mixture at RT for 60 h, the resulting suspended solid was collected by Buchner filtration, and washed with cold MeOH, to afford catalyst **dhQD-10** in 52% yield as a white solid. **$[\alpha]_D^{25}$** = +75 ° (c = 0.11 in aq. HCl 1M). **$^1$H NMR** (400 MHz, DMSO-d$_6$, 60 °C) δ = 8.78 (d, *J* = 4.7 Hz, 1H), 7.96 (d, *J* = 9.1 Hz, 1H), 7.83 (d, *J* = 2.9 Hz, 1H), 7.79 (br s, 1H), 7.56 (d, *J* = 4.7 Hz, 1H), 7.43 (dd, *J* = 9.0, 2.7 Hz, 1H), 7.28 (br s, 2H), 6.04 (br d, *J* = 10.7 Hz, 1H), 3.95 (s, 3H), 3.30 (q, *J* = 9.2 Hz, 1H), 2.98-2.78 (m, 4H), 1.62-1.50 (m, 1H), 1.50-1.24 (m, 5H), 1.08-0.98 (m, 1H), 0.96-0.86 (m, 1H), 0.84 (t, *J* = 7.1 Hz, 3H). **$^{13}$C NMR** (101 MHz, DMSO-d$_6$, 60 °C) δ = 184.0, 183.0, 169.8, 168.4, 158.3, 148.1, 144.8, 144.7, 131.9, 128.0, 122.3, 119.9, 102.2, 59.7, 56.0, 53.3 (br), 49.6, 48.8, 37.4, 27.6, 26.2, 25.8, 25.6, 12.1. **HRMS**: calculated for $C_{24}H_{29}N_4O_3$ [M + H$^+$] = 421.2240; found: 421.2239.

**Preparation of catalyst dhQN-10.**

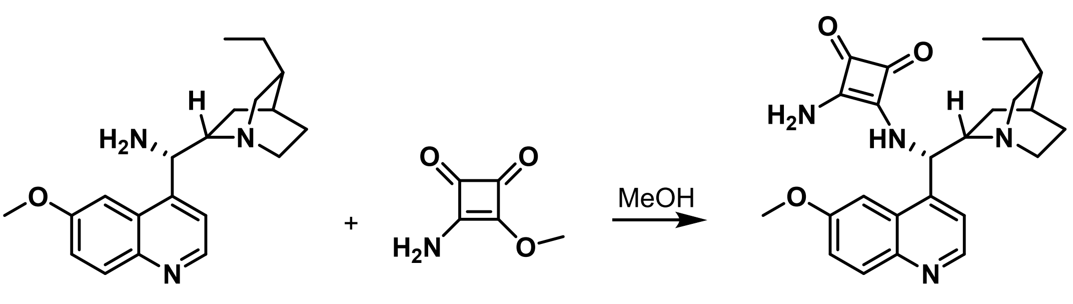

dhQN-10

Following the procedure used for **dhQD-10**, but using 9-amino-9-deoxy *epi*-dihydroquinine, catalyst **dhQN-10** was obtained in 62% yield as an off-white solid. **$[\alpha]_D^{25}$** = -65 (c = 0.11 in aq. HCl 1M). **$^1$H NMR** (400 MHz, DMSO-d$_6$, 60 °C) δ = 8.80-8.76 (m, 1H), 8.00-7.94 (m, 1H),

7.83 (br s, 1H), 7.82 (br s, 1H), 7.58-7.55 (m, 1H), 7.46-7.40 (m, 1H), 7.30 (br s, 2H), 5.93 (br d, *J* = 10.1 Hz, 1H), 3.95 (s, 3H), 3.40-3.26 (m, 2H), 3.17 (dd, *J* = 13.6, 9.8 Hz, 1H), 2.67-2.54 (m, 1H), 2.47-2.40 (m, 1H), 1.60-1.26 (m, 7H), 0.82 (t, *J* = 7.2 Hz, 3H), 0.68-0.58 (m, 1H). **$^{13}$C NMR** (101 MHz, DMSO-$d_6$, 60 °C) δ = 183.9, 183.0, 170.0, 168.2, 158.3, 148.2, 144.8, 144.5, 131.9, 128.0, 122.1, 120.1, 102.5, 59.5, 57.9, 56.2, 54.3 (br), 40.8, 37.4, 28.6, 27.3, 26.5, 25.6, 12.3. **HRMS**: calculated for $C_{24}H_{29}N_4O_3$ [M + H$^+$] = 421.2240; found: 421.2234.

**Copies of $^{1}H$ and $^{13}C$ NMR spectra of catalysts dhQD-2, dhQD-10, dhQN-2, dhQN-10 (in DMSO-$d_6$ at 60 °C)**

**dhQD-2**

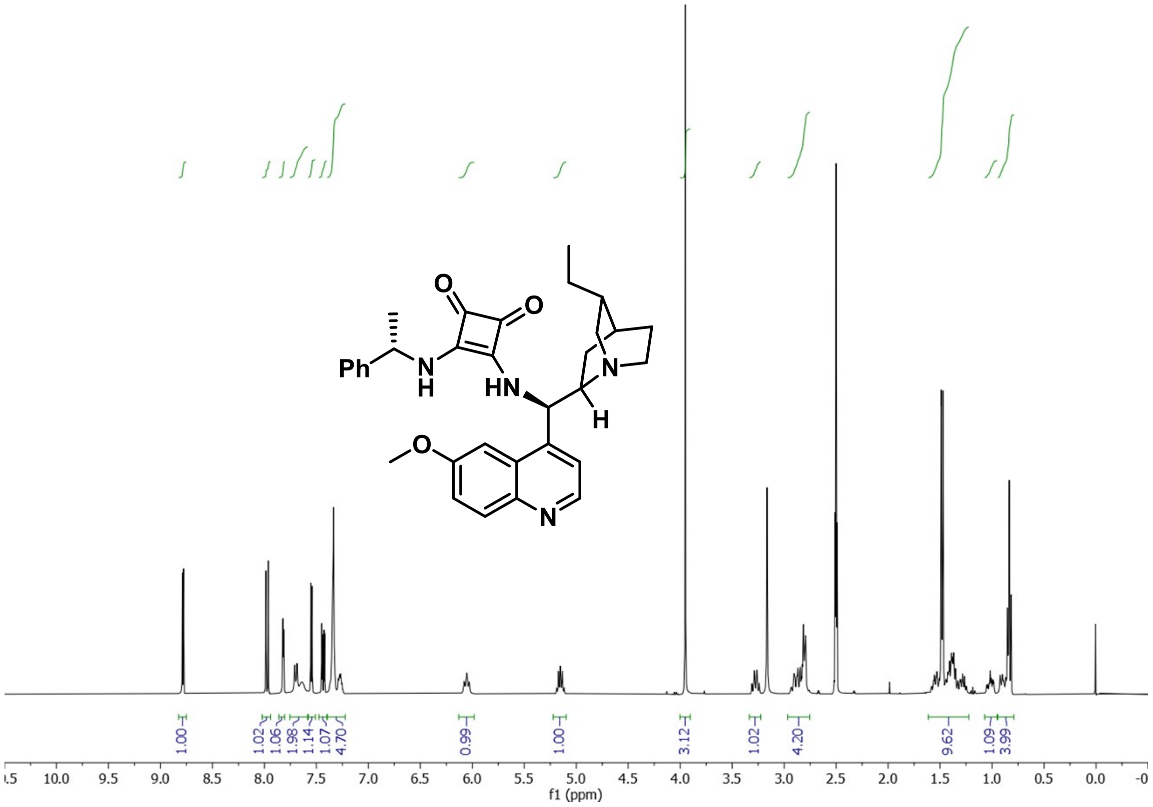


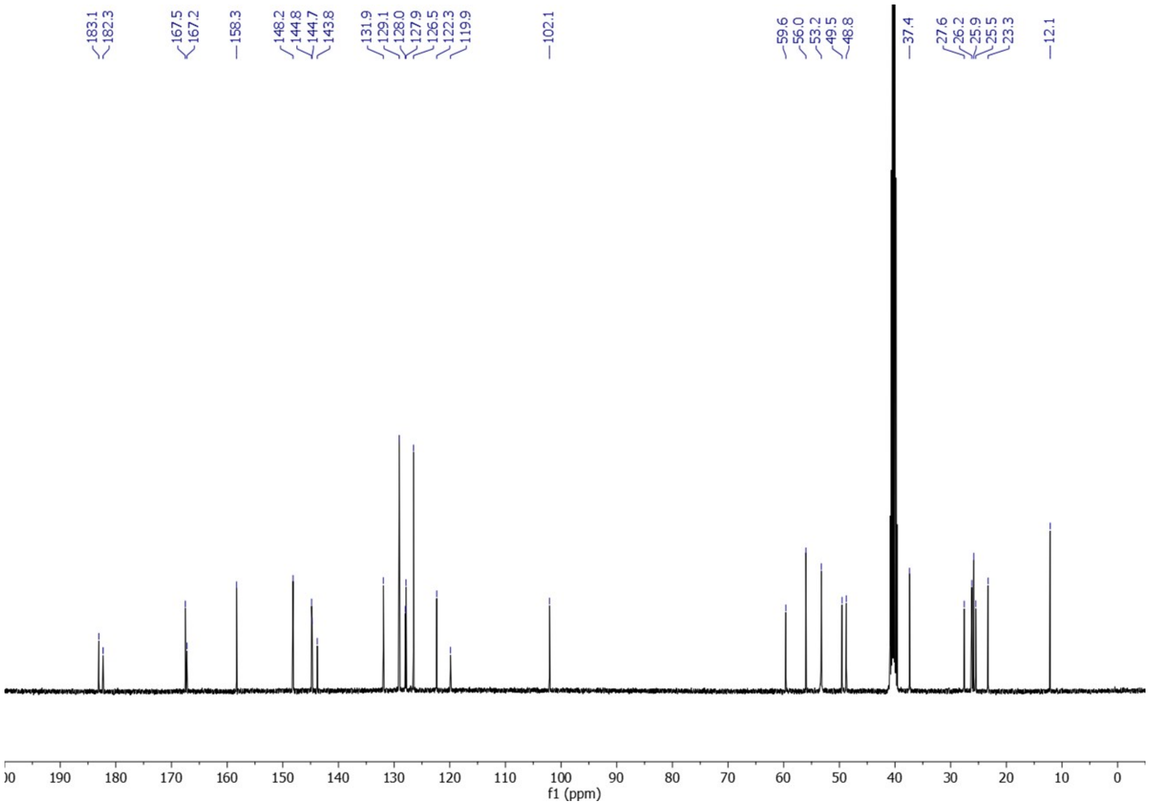

**dhQN-2**

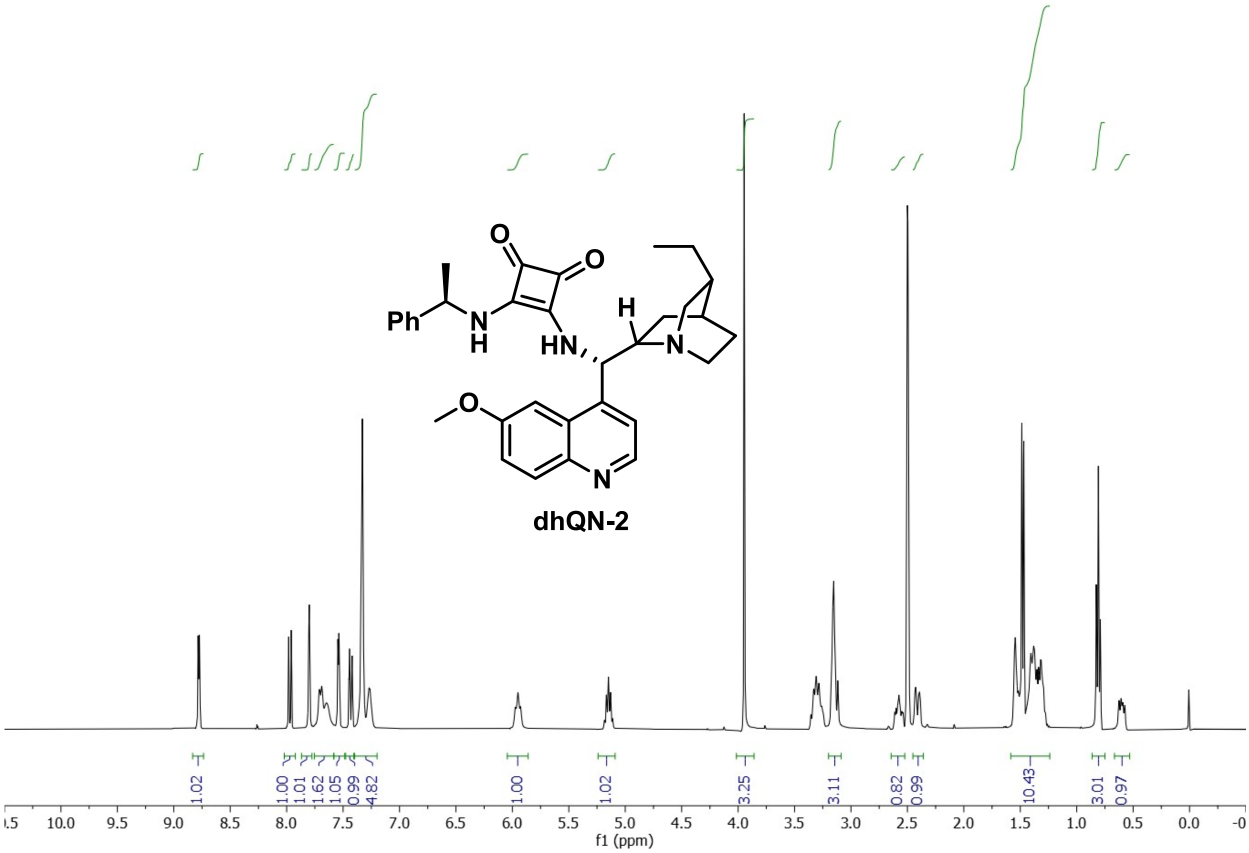

O
O
Ph
N
H
HN
H
N
O
N
dhQN-2
1.02
1.00
1.01
1.62
1.05
0.99
4.82
1.00
1.02
3.25
3.11
0.82
0.99
10.43
3.01
0.97
f1 (ppm)


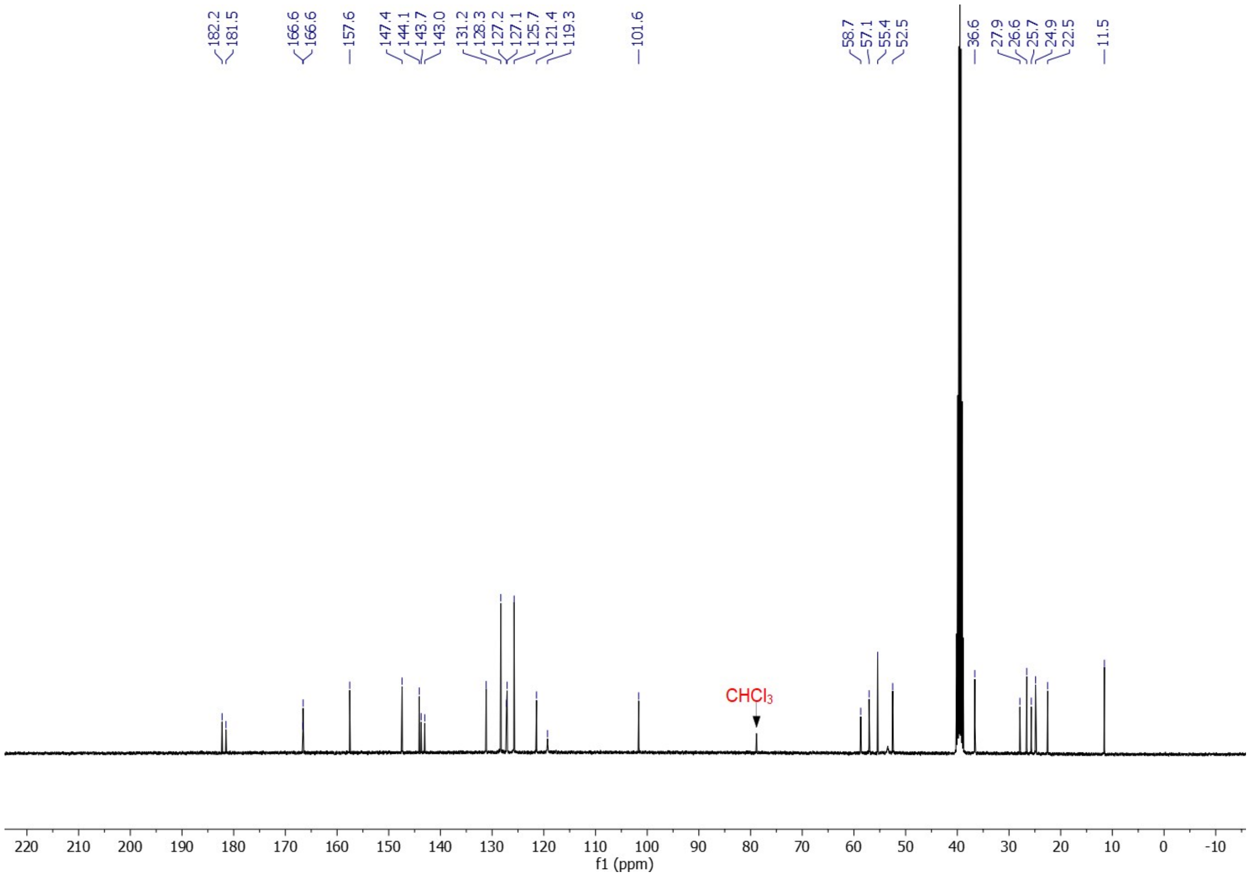

182.2
181.5
166.6
166.6
157.6
147.4
144.1
143.7
143.0
131.2
128.3
127.2
127.1
125.7
121.4
119.3
101.6
58.7
57.1
55.4
52.5
36.6
27.9
26.6
25.7
24.9
22.5
11.5
CHCl3
f1 (ppm)

**dhQD-10.**

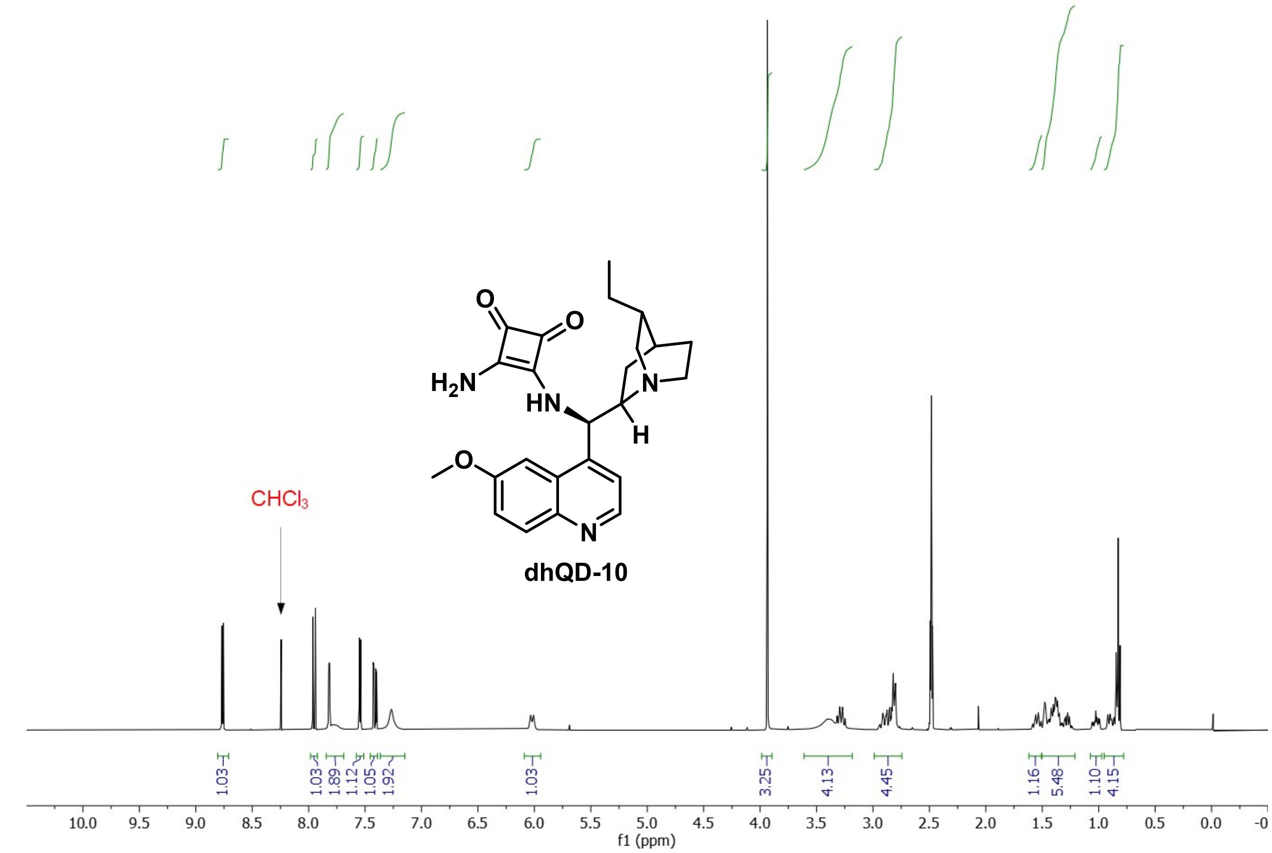
CHCl3
H2N
HN
O
N
H
dhQD-10
1.03
1.03
1.89
1.12
1.05
1.92
1.03
3.25
4.13
4.45
1.16
5.48
1.10
4.15
10.0 9.5 9.0 8.5 8.0 7.5 7.0 6.5 6.0 5.5 5.0 4.5 4.0 3.5 3.0 2.5 2.0 1.5 1.0 0.5 0.0 -0
f1 (ppm)

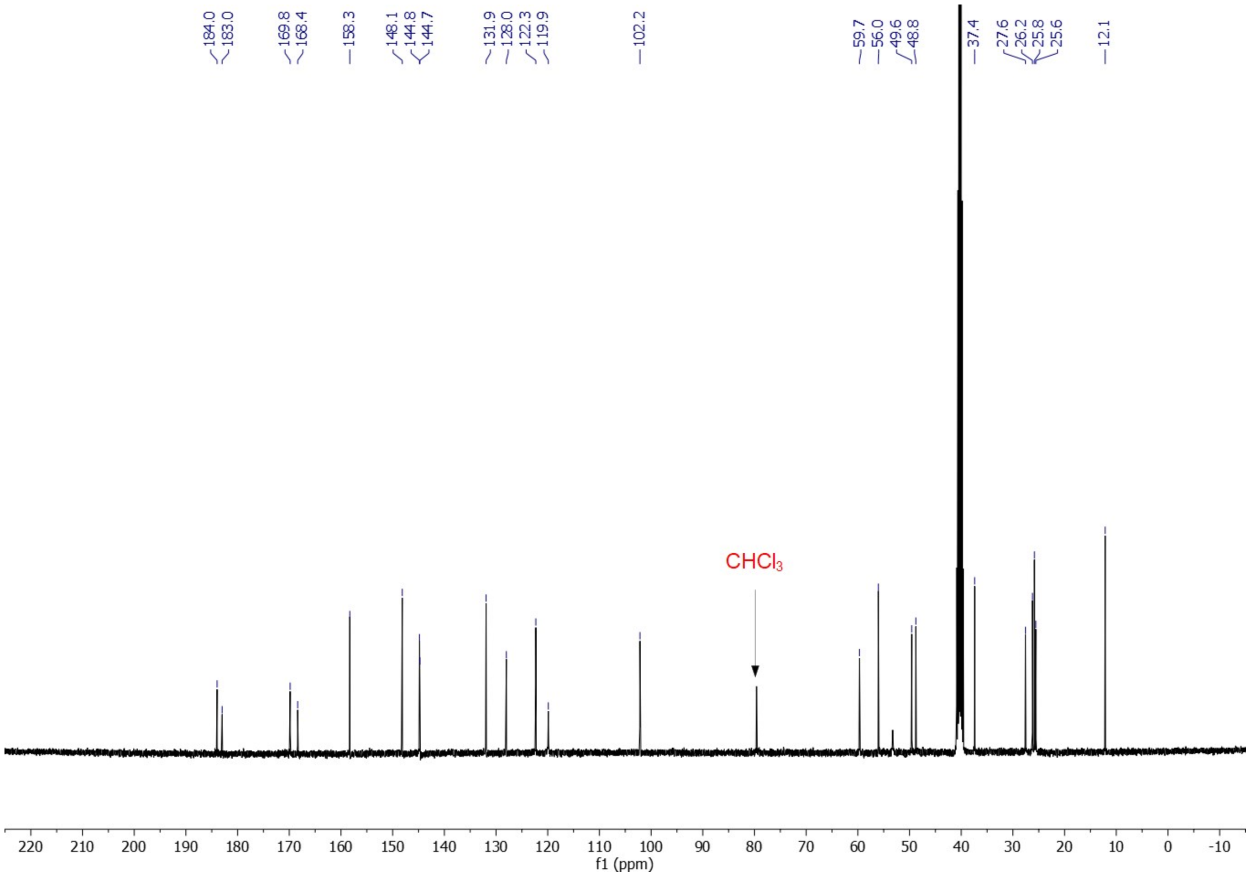
184.0
183.0
169.8
168.4
158.3
148.1
144.8
144.7
131.9
128.0
122.3
119.9
102.2
59.7
56.0
49.6
48.8
37.4
27.6
26.2
25.8
25.6
12.1
CHCl3
220 210 200 190 180 170 160 150 140 130 120 110 100 90 80 70 60 50 40 30 20 10 0 -10
f1 (ppm)

**dhQN-10**

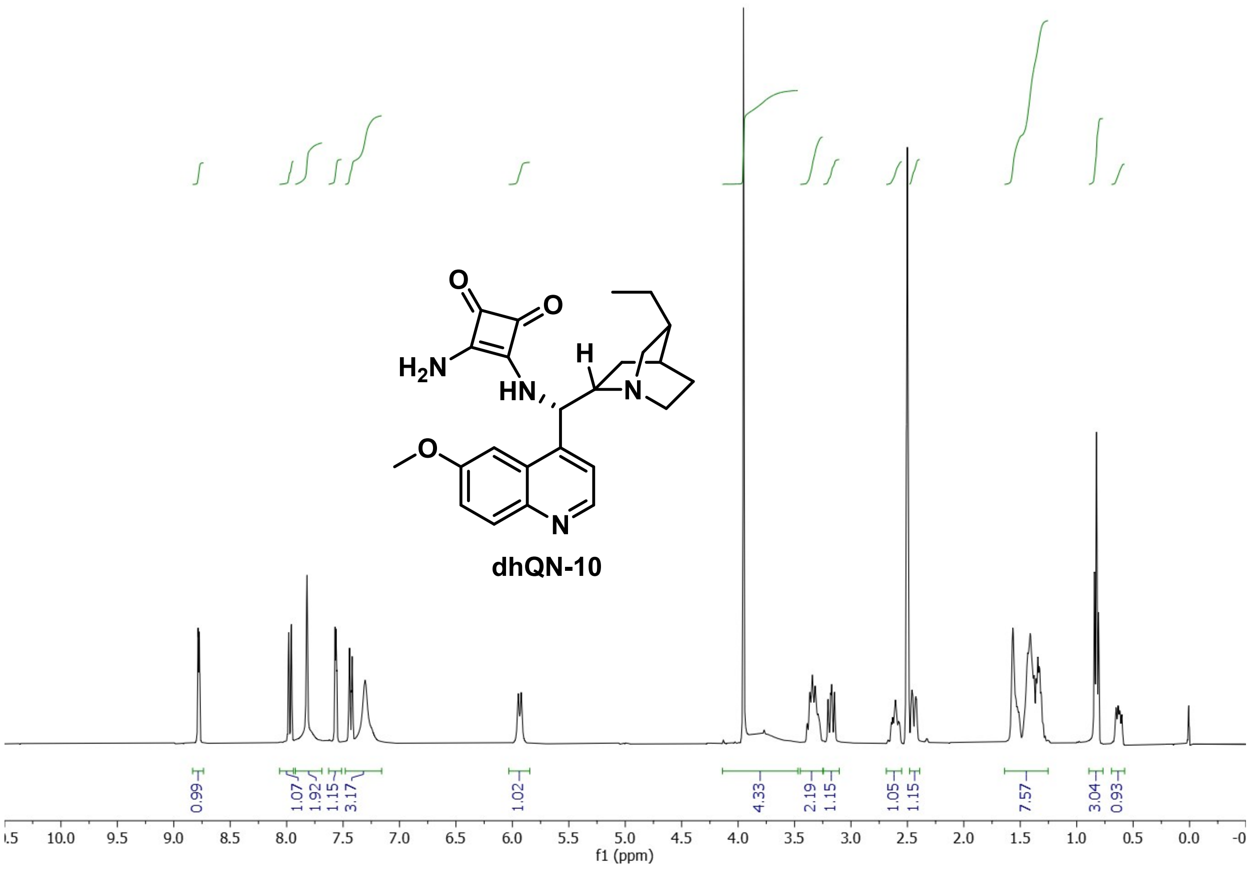
O
O
H2N
HN
H
N
O
N
dhQN-10
0.99
1.07
1.92
1.15
3.17
1.02
4.33
2.19
1.15
1.05
1.15
7.57
3.04
0.93
f1 (ppm)

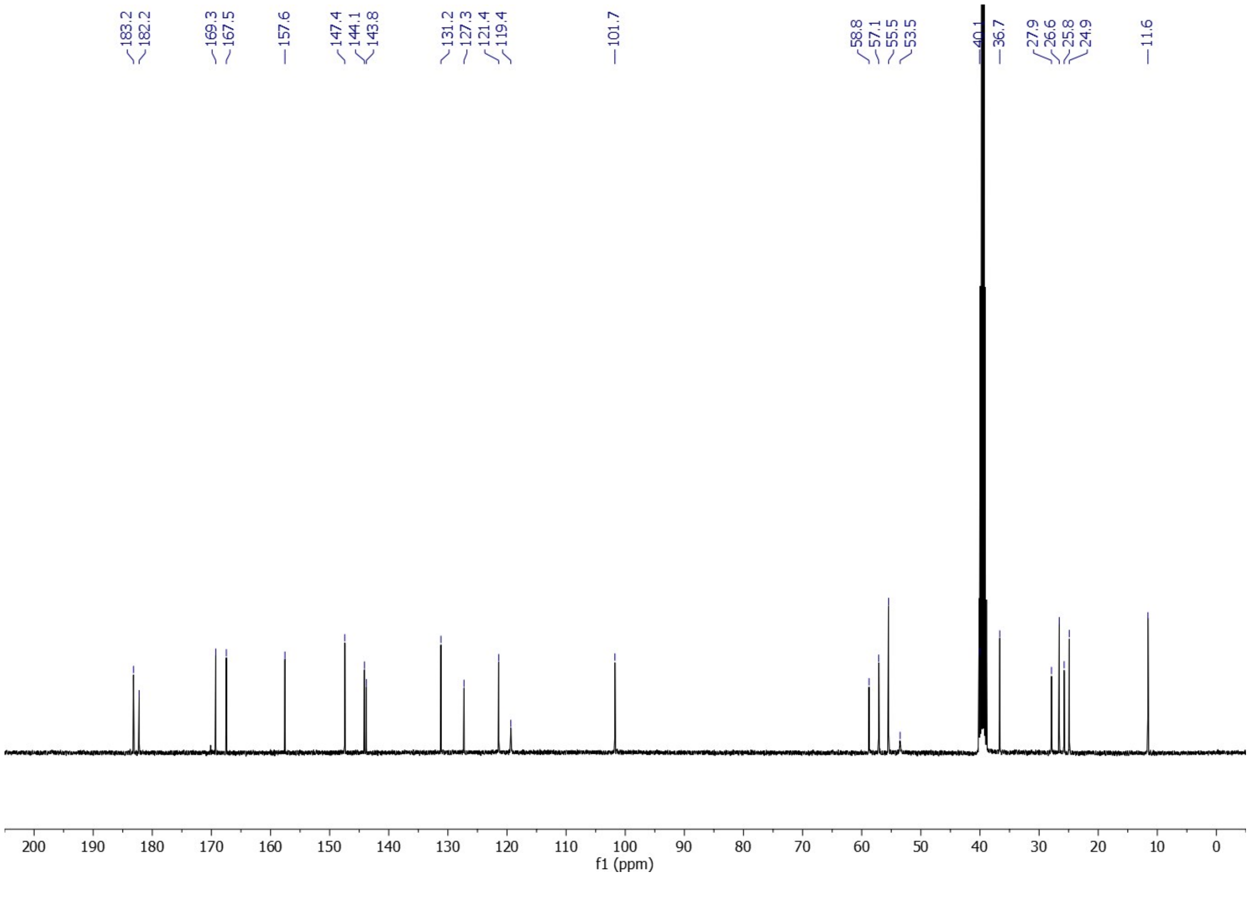
183.2
182.2
169.3
167.5
157.6
147.4
144.1
143.8
131.2
127.3
121.4
119.4
101.7
58.8
57.1
55.5
53.5
40.1
36.7
27.9
26.6
25.8
24.9
11.6
f1 (ppm)

## Synthesis and characterization of catalytic products 4, amidoalcohols 5 and aminoalcohols 6.

### General procedure A for the one pot synthesis of products 4.

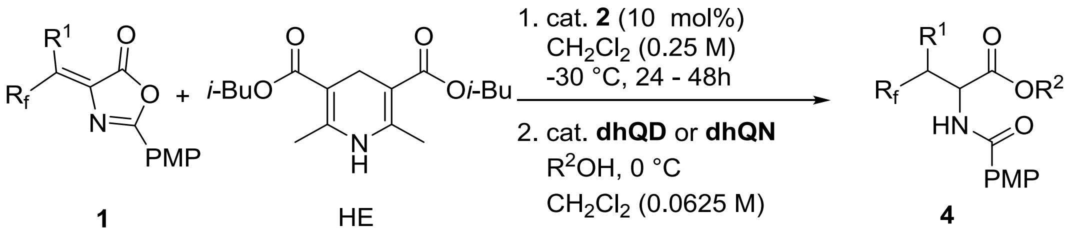


In a test tube equipped with a magnetic stirring bar, azlactone **1** (1 equiv, 0.15 mmol), catalyst **2p** (0.015 mmol, 10 mol%) and $CH_2Cl_2$ (0.60 mL) were added in this order. The resulting mixture was placed at -30 °C and stirred for 5 minutes after which Hantzsch ester HE (1.1 equiv, 0.165 mmol) was added and the solution stirred until full consumption of the starting azlactone **1** (24 - 48 h, as shown by $^{19}F$ NMR analysis). Afterwards, the test tube was moved to 0 °C and $CH_2Cl_2$ (1.8 mL), catalyst **dqQD** or **dhQN** (0.0075 mmol, 5 mol%), and the corresponding alcohol (2 equiv., 0.3 mmol) were added. The resulting reaction mixture was stirred at 0 °C until full consumption of the intermediate (as shown by $^{19}F$ NMR analysis). Then the reaction mixture was filtered through a short plug of silica gel, the test tube rinsed with $CH_2Cl_2$ (2x1 mL), the plug eluted with $Et_2O$ (3x1 mL), the solvents removed under vacuum, and the residue analyzed by $^{19}F$ NMR to determine the diastereoselectivity of the reaction. Purification by column chromatography on silica gel (mixtures of $Et_2O/CH_2Cl_2$) afforded the corresponding *N*-benzoyl α-amino ester derivatives **4**.

### General procedure B for the synthesis of products 4.

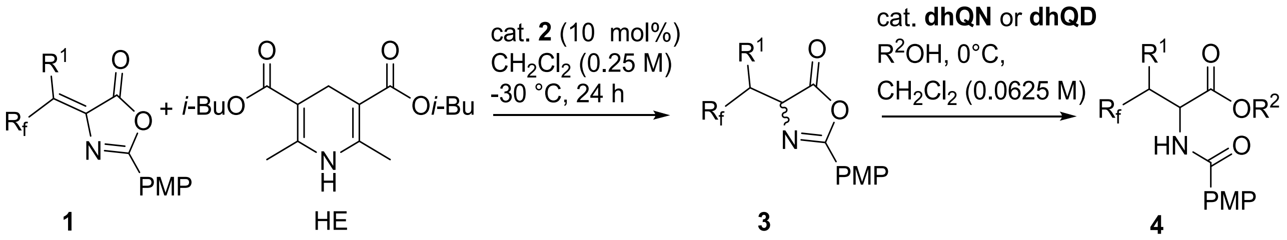


In a test tube equipped with a magnetic stirring bar, azlactone **1** (1 equiv, 0.15 mmol), catalyst **2p** (0.015 mmol 10 mol%) and $CH_2Cl_2$ (0.6 mL) were added in this order. The resulting mixture was placed at -30 °C and stirred for 5 minutes after which Hantzsch ester HE (1.5 equiv, 0.225 mmol) was added and the solution stirred until full consumption of the starting azlactone **1** (24 h, as shown by $^{19}F$ NMR analysis). Afterwards, the reaction mixture

was directly loaded onto a very short column of silica gel, eluted with $CH_2Cl_2$ and the fractions collected as fast as possible due to instability of the reduced azlactone **3** on silica, to isolate the desired product. In a test tube equipped with a magnetic stirring bar, the thus obtained azlactone **3** was dissolved in $CH_2Cl_2$ (2.4 mL). To this solution, cooled to 0 °C, catalyst **dqQD** or **dhQN** (0.0075 mmol, 5 mol%) were added, followed by the corresponding alcohol (2 equiv. 0.3 mmol). The resulting mixture was stirred at 0 °C until full consumption of the intermediate azlactone (as shown by $^{19}F$ NMR analysis). Then, the solution was filtered through a short plug of silica gel, the test tube was rinsed twice with $CH_2Cl_2$ (2x1 mL), the plug was eluted three times with (3x1 mL) $Et_2O$, the solvents removed under vacuum, and the residue analyzed by $^{19}F$ NMR to determine the diastereoselectivity of the reaction. Purification by column chromatography on silica gel (mixtures of $Et_2O/CH_2Cl_2$) afforded the corresponding *N*-benzoyl α-amino ester derivatives **4**.

## General procedure C for the synthesis of products 4.

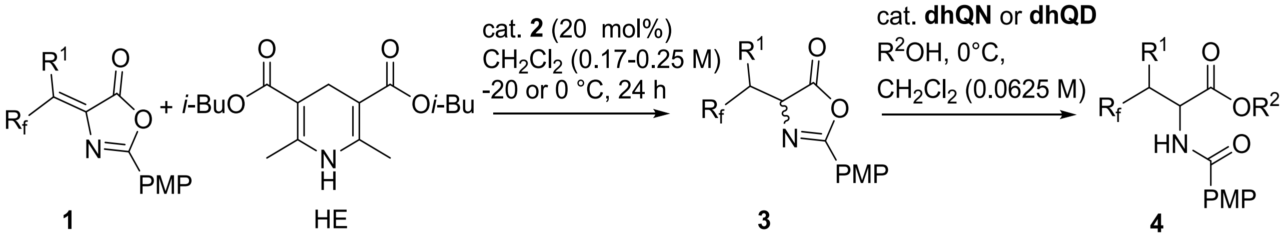


In a test tube equipped with a magnetic stirring bar, azlactone **1** (1 equiv, 0.05 mmol), catalyst **2p** (0.01 mmol, 20 mol%) and $CH_2Cl_2$ (0.30 mL) were added in this order. The resulting mixture was placed at -20 or 0 °C and stirred for 5 minutes after which Hantzsch ester HE (1.1 equiv, 0.055 mmol) was added and the solution stirred until full consumption of the starting azlactone **1** (24 h, as shown by $^{19}F$ NMR analysis). Afterwards, the test tube was moved to 0 °C and $CH_2Cl_2$ (0.60 mL), catalyst **dqQD** or **dhQN** (0.005 mmol, 10 mol%), and allyl alcohol (2 equiv., 0.01 mmol) were added. The resulting reaction mixture was stirred at 0 °C until full consumption of the intermediate (as shown by $^{19}F$ NMR analysis). Then the reaction mixture was filtered through a short plug of silica gel, the test tube rinsed with $CH_2Cl_2$ (2x1 mL), the plug eluted with $Et_2O$ (3x1 mL), the solvents removed under vacuum, and the residue analyzed by $^{19}F$ NMR to determine the diastereoselectivity of the reaction. Purification by column chromatography on silica gel (mixtures of $Et_2O/CH_2Cl_2$) afforded the corresponding *N*-benzoyl α-amino ester derivatives **4**.

## General procedure for the synthesis of racemic products 4.

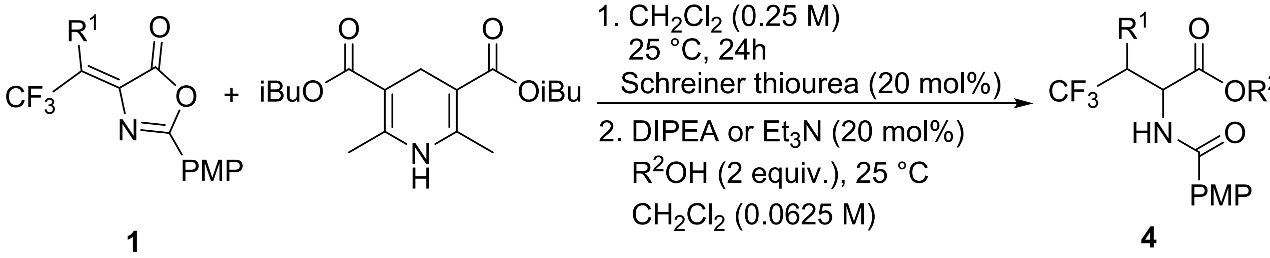

In a test tube equipped with a magnetic stirring bar, azlactone **1** (1 equiv, 0.05 mmol), $CH_2Cl_2$ (0.2 mL), Schreiner's thiourea (0.2 equiv, 0.01 mmol) and Hantzsch ester derivative (1.5 equiv, 0.075 mmol) were added in this order and stirred at r.t. until full consumption of the corresponding azlactone **1** (24 h, as shown by $^{19}F$ NMR analysis). Afterwards, $CH_2Cl_2$ (0.6 mL), DIPEA or $Et_3N$ (ca. 0.01 mmol, 20 mol%), and the corresponding alcohol (2 equiv. 0.1 mmol) were added. The resulting reaction mixture was stirred at r.t. until full consumption of the intermediate (as shown by $^{19}F$ NMR analysis, which also served to determine the d.r. values). Then the reaction mixture was filtered through a short plug of silica gel, the test tube rinsed with $CH_2Cl_2$ (2x1 mL), the plug eluted with $Et_2O$ (3x1 mL) and the solvents removed under vacuum. Purification by column chromatography on silica gel (mixtures of $Et_2O/CH_2Cl_2$) afforded the corresponding racemic *N*-benzoyl α-amino ester derivatives **4**.

**Allyl (2*R*,3*R*)-4,4,4-trifluoro-2-(4-methoxybenzamido)-3-phenylbutanoate** (*anti*-**4d**).

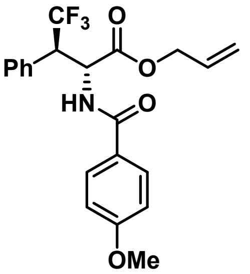

Following general procedure A from azlactone **1d**, cat. **2p**, allyl alcohol, and cat. **dhQD-2**, product *anti*-**4d** was obtained as a white solid in 83% yield (d.r. of the crude >20:1) and 99% ee after column chromatography on silica gel ($CH_2Cl_2/Et_2O$ = 100:1). **$[\alpha]_D^{25}$** = -30 (c = 0.27 in $CHCl_3$). **$^1$H NMR** (400 MHz) δ = 7.68-7.60 (m, 2H), 7.44-7.32 (m, 5H), 6.96-6.85 (m, 2H), 6.29 (d, *J* = 9.3 Hz, 1H), 5.89 (ddt, *J* = 17.3, 10.4, 6.0 Hz, 1H), 5.66 (dd, *J* = 9.3, 4.4 Hz, 1H), 5.38 – 5.26 (m, 2H), 4.69 – 4.60 (m, 2H), 4.14 (qd, *J* = 9.4, 4.4 Hz, 1H), 3.83 (s, 3H). **$^{19}$F NMR** (376 MHz, $CDCl_3$) δ = -71.02 (d, *J* = 9.3 Hz, 3F). **$^{13}$C NMR** (101 MHz, $CDCl_3$) δ = 169.8, 166.4, 162.8, 131.1, 130.5, 129.4, 129.31, 129.26, 129.0, 125.84, 125.79 (q, $^1J_{C\text{-}F}$ = 280.7 Hz), 119.9, 114.0, 67.0, 55.5, 52.4-50.5 (m, 2C). **HPLC**: Chiralpak AD-H (*n*-hexane/iPrOH 80:20, flow-rate 0.75 mL/min; $t_{major}$ = 11.4 min; $t_{minor}$ = 21.7 min).

**Allyl (2*S*,3*R*)-4,4,4-trifluoro-2-(4-methoxybenzamido)-3-phenylbutanoate** (*syn*-**4d**).

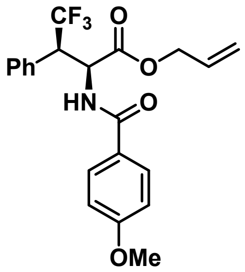

Following general procedure A from azlactone **1d**, cat. **2p**, allyl alcohol, and cat. **dhQN-10**, product *syn*-**4d** was obtained as an off-white solid in 72% yield (d.r. of the crude = 7.5:1) and >99% ee after column chromatography on silica gel ($CH_2Cl_2/Et_2O$ = 100:1). **$^1$H NMR** (400 MHz, $CDCl_3$) [signals of the *syn* isomer] δ = 7.78-7.69 (m, 2H), 7.34 (br s, 5H), 6.96-6.90 (m, 2H), 6.67 (d, *J* = 9.0 Hz, 1H), 5.70 (ddt, *J* = 17.3, 10.3, 6.0 Hz, 1H), 5.46 (dd, *J* = 9.0, 7.0 Hz, 1H), 5.25-5.14 (m, 2H), 4.56-4.44 (m, 2H), 4.12-3.98 (m, 1H), 3.84 (s, 3H). **$^{19}$F NMR** (376 MHz, $CDCl_3$) [signals of the *syn* isomer] δ = -64.07 (d, *J* = 9.5 Hz, 3F). **$^{13}$C NMR** (100 MHz, $CDCl_3$) [signals of the *syn* isomer] δ = 169.8, 166.6, 162.8, 131.3, 131.0, 129.5, 129.1, 129.0, 126.0 (q, $^1J_{C\text{-}F}$ = 281.1 Hz), 125.8, 119.4, 114.0, 66.7, 55.6, 53.1, 51.9 (q, $^2J_{C\text{-}F}$ = 26.5 Hz). **HPLC** [peaks of the *syn*-isomer]: Chiralpak AD-H (*n*-hexane/iPrOH 80:20, flow-rate 0.75 mL/min; $t_{major}$ = 24.7 min; $t_{minor}$ = 14.9 min).

**HRMS** [*syn*-**4d** and *anti*-**4d** isomers]: calculated for $C_{21}H_{21}F_3NO_4$ [M + $H^+$] = 408.1423; found: 408.1414.

**Allyl (2*S*,3*S*)-4,4,4-trifluoro-2-(4-methoxybenzamido)-3-phenylbutanoate** (*ent-anti*-**4d**).

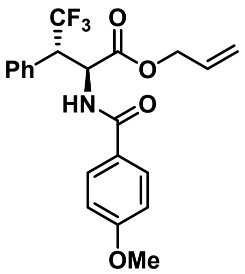

Following general procedure A from azlactone **1d**, cat. *ent*-**2p**, allyl alcohol, and cat. **dhQN-2**, product *ent-anti*-**4d** was obtained as a white solid in 90% yield (d.r. of the crude > 20:1) and 98% ee after column chromatography on silica gel ($CH_2Cl_2/Et_2O$ = 100:1). $[\alpha]_D^{25}$ = +33 (c = 0.27 in $CHCl_3$). Spectral data were identical to *anti*-**4d**. **HPLC**: Chiralpak AD-H (*n*-hexane/iPrOH 80:20, flow-rate 0.75 mL/min; $t_{major}$ = 20.5 min; $t_{minor}$ = 10.9 min).

**Allyl (2*R*,3*S*)-4,4,4-trifluoro-2-(4-methoxybenzamido)-3-phenylbutanoate** (*ent-syn*-**4d**).

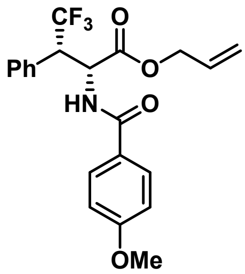

Following general procedure A from azlactone **1d**, cat. *ent*-**2p**, allyl alcohol, and cat. **dhQD-10**, product *ent-syn*-**4d** was obtained as an off-white solid in 88% yield (d.r. of the crude = 4.3:1) and 99% ee after column chromatography on silica gel ($CH_2Cl_2/Et_2O$ = 100:1). Spectral data were identical to *syn*-**4d. HPLC** [peaks of the *syn*-isomer]: Chiralpak AD-H (*n*-hexane/iPrOH 80:20, flow-rate 0.75 mL/min; $t_{major}$ = 14.2 min; $t_{minor}$ = 23.9 min).

**Allyl (2*R*,3*R*)-3-(4-bromophenyl)-4,4,4-trifluoro-2-(4-methoxybenzamido)butanoate** ***anti*-4e.**

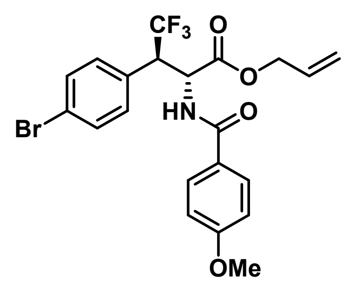

Following general procedure A from azlactone **1e**, cat. **2p**, allyl alcohol, and cat. **dhQD-2**, product *anti*-**4e** was obtained as a white solid in 93% yield (d.r. of the crude > 20:1) and 98% ee after column chromatography on silica gel ($CH_2Cl_2/Et_2O$ = 100:1). **$[\alpha]_D^{25}$** = -33 (c = 0.27 in $CHCl_3$). **$^1$H NMR** (400 MHz, Chloroform-*d*) δ = 7.66-7.60 (m, 2H), 7.54-7.49 (m, 2H), 7.25 (br d, *J* = 8.5 Hz, 2H), 6.95-6.84 (m, 2H), 6.38 (d, *J* = 9.2 Hz, 1H), 5.88 (ddt, *J* = 17.2, 10.3, 6.0 Hz, 1H), 5.64 (dd, *J* = 9.2, 4.7 Hz, 1H), 5.37-5.26 (m, 2H), 4.69-4.57 (m, 2H), 4.12 (qd, *J* = 9.3, 4.7 Hz, 1H), 3.82 (s, 3H). **$^{19}$F NMR** (376 MHz, $CDCl_3$) δ = -66.29 (d, *J* = 9.2 Hz, 3F). **$^{13}$C NMR** (100 MHz, $CDCl_3$) δ = 169.6, 166.4, 162.8, 132.3, 131.1, 130.9, 129.7, 129.0, 125.6, 125.5 (q, $^1J_{C\text{-}F}$ = 280.7 Hz), 123.6, 120.1, 114.0, 67.1, 55.5, 51.8-50.6 (m, 2C). **HPLC**: Chiralpak AD-H (*n*-hexane/iPrOH 80:20, flow-rate 0.75 mL/min; $t_{major}$ = 12.7 min; $t_{minor}$ = 40.3 min).

**Allyl (2*S*,3*R*)-3-(4-bromophenyl)-4,4,4-trifluoro-2-(4-methoxybenzamido)butanoate** ***syn*-4e.**

Following general procedure A from azlactone **1e**, cat. **2p**, allyl alcohol, and cat. **dhQN-10**,

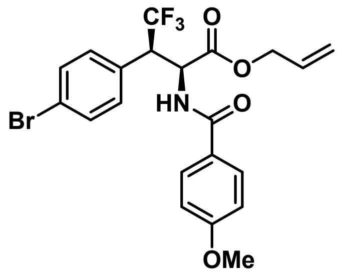

product *syn*-**4e** was obtained as a white solid in 72% yield (d.r. of the crude = 3.6:1) and 99% ee after column chromatography on silica gel ($CH_2Cl_2/Et_2O$ = 100:1). **$^1$H NMR** (400 MHz, $CDCl_3$) [signals of the *syn* isomer] δ = 7.77-7.70 (m, 2H), 7.50-7.44 (m, 2H), 7.24-7.18 (m, 2H), 6.98-6.91 (m, 2H), 6.66 (d, *J* = 9.0 Hz, 1H), 5.73 (ddt, *J* = 17.2, 10.3, 6.0 Hz, 1H), 5.44 (dd, *J* = 9.0, 6.7 Hz, 1H), 5.28-5.18 (m, 2H), 4.58 – 4.49 (m, 2H), 4.05 (qd, *J* = 9.4, 6.8 Hz, 1H), 3.85 (s, 3H). **$^{19}$F NMR** (376 MHz, $CDCl_3$) [signals of the *syn* isomer] δ = -64.22 (d, *J* = 9.3 Hz, 3F). **$^{13}$C NMR** (101 MHz, $CDCl_3$) [signals of the *syn* isomer] δ = 169.5, 166.6, 162.9, 132.2, 131.1, 130.9, 130.4, 129.2, 125.6, 125.7 (q, $^1J_{C\text{-}F}$ = 281.3 Hz),123.5, 119.8, 114.1, 66.9, 55.6, 52.8, 51.5 (q, $^2J_{C\text{-}F}$ = 26.7 Hz). **HPLC** [peaks of the *syn*-isomer]: Chiralpak AD-H (*n*-hexane/iPrOH 80:20, flow-rate 0.75 mL/min; $t_{major}$ = 19.6 min; $t_{minor}$ = 18.5 min).

**HRMS** [*syn*-**4e** and *anti*-**4e** isomers]: calculated for $C_{21}H_{20}BrF_3NO_4$ [M + H$^+$] = 486.0528; found: 486.0518.

**Allyl (2*R*,3*R*)-3-(4-chlorophenyl)-4,4,4-trifluoro-2-(4-methoxybenzamido)butanoate** *anti*-**4f**.

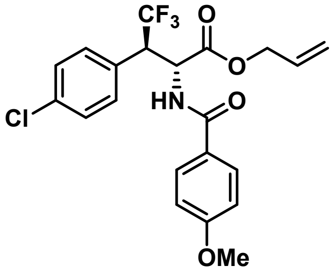

Following general procedure A from azlactone **1f**, cat. **2p**, allyl alcohol, and cat. **dhQD-2**, product *anti*-**4f** was obtained as a white solid in 90% yield (d.r. of the crude > 20:1) and 99% ee after column chromatography on silica gel ($CH_2Cl_2/Et_2O$ = 100:1). **$[\alpha]_D^{25}$** = -29 (c = 0.27 in $CHCl_3$). **$^1$H NMR** (400 MHz, $CDCl_3$) δ = 7.67 - 7.62 (m, 2H), 7.41-7.36 (m, 2H), 7.34-7.28 (m, 2H), 6.96-6.87 (m, 2H), 6.31 (d, *J* = 8.7 Hz, 1H), 5.89 (ddt, *J* = 17.2, 10.4, 6.1 Hz, 1H), 5.64 (dd, *J* = 9.2, 4.5 Hz, 1H), 5.40-5.26 (m, 2H), 4.67-4.62 (m, 2H), 4.12 (qd, *J* = 9.3, 4.5 Hz, 1H), 3.84 (s, 3H). **$^{19}$F NMR** (376 MHz, $CDCl_3$) δ = -66.32 (d, *J* = 9.3 Hz, 3F). **$^{13}$C NMR** (101 MHz, $CDCl_3$) δ = 169.7, 166.4, 162.9, 135.5, 131.0, 130.8, 129.4, 129.2, 129.0, 125.6, 125.5 (d, $^1J_{C\text{-}F}$ = 280.8 Hz) 120.1, 114.1, 67.2, 55.6, 51.6-50.6 (m, 2C). **HPLC**: Chiralpak AD-H (*n*-hexane/iPrOH 80:20, flow-rate 0.75 mL/min; $t_{major}$ = 11.8 min; $t_{minor}$ = 34.7 min).

**Allyl (2*S*,3*R*)-3-(4-chlorophenyl)-4,4,4-trifluoro-2-(4-methoxybenzamido)butanoate** *syn*-**4f**.

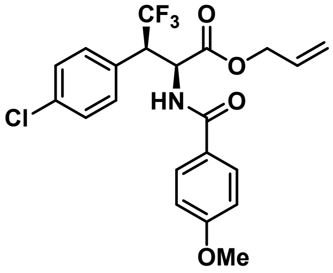

Following general procedure A from azlactone **1f**, cat. **2p**, allyl alcohol, and cat. **dhQN-10**, product *syn*-**4f** was obtained as a white solid in 77% yield (d.r. of the crude = 4.4:1) and 99% ee after column chromatography on silica gel ($CH_2Cl_2/Et_2O$ = 100:1). **$^1$H NMR** (400 MHz, $CDCl_3$) [signals of the *syn* isomer] δ = 7.78-7.71 (m, 2H), 7.33-7.27 (m, 4H), 6.98-6.90 (m, 2H), 6.67 (d, *J* = 9.0 Hz, 1H), 5.73 (ddt, *J* = 17.2, 10.3, 6.0 Hz, 1H), 5.44 (dd, *J* = 9.0, 6.7 Hz, 1H), 5.28-5.20 (m, 2H), 4.56 – 4.51 (m, 2H), 4.06 (qd, *J* = 9.4, 6.8 Hz, 1H), 3.85 (s, 3H). **$^{19}$F NMR** (376 MHz, $CDCl_3$) [signals of the *syn* isomer] δ = -64.24 (d, *J* = 9.2 Hz, 3F). **$^{13}$C NMR** (101 MHz, $CDCl_3$) [signals of the *syn* isomer] δ = 169.5, 166.6, 162.9, 135.3, 130.9, 130.8, 129.8, 129.2, 129.1, 127.2, 125.8 (q, $^1J_{C\text{-}F}$ = 281.6 Hz), 119.8, 114.1, 66.9, 55.6, 52.9, 51.2 (q, $^2J_{C\text{-}F}$ = 26.7 Hz). **HPLC** [peaks of the *syn* isomer]: Chiralpak AD-H (*n*-hexane/iPrOH 80:20, flow-rate 0.75 mL/min; $t_{major}$ = 20.1 min; $t_{minor}$ = 19.4 min).

**HRMS** [*syn*-**4f** and *anti*-**4f** isomers]: calculated for $C_{21}H_{20}ClF_3NO_4$ [M + H$^+$] = 442.1033; found: 442.1027.

**Allyl (2*R*,3*R*)-4,4,4-trifluoro-2-(4-methoxybenzamido)-3-(m-tolyl)butanoate *anti*-4g.**

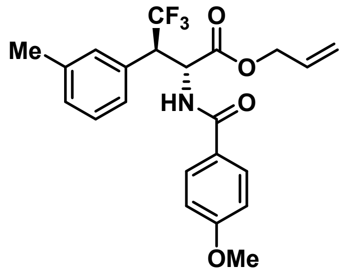

Following general procedure A from azlactone **1g**, cat. **2p**, allyl alcohol, and cat. **dhQD-2**, product *anti*-**4g** was obtained as a white solid in 96% yield (d.r. of the crude > 20:1) and 98% ee after column chromatography on silica gel ($CH_2Cl_2/Et_2O$ = 100:1). **$[\alpha]_D^{25}$** = -37 (c = 0.27 in $CHCl_3$). **$^1$H NMR** (400 MHz, $CDCl_3$) δ = 7.70 – 7.61 (m, 2H), 7.36 – 7.25 (m, 1H), 7.23 – 7.12 (m, 3H), 6.98 – 6.77 (m, 2H), 6.31 (d, *J* = 9.3 Hz, 1H), 5.89 (ddt, *J* = 17.2, 10.4, 6.0 Hz, 1H), 5.64 (dd, *J* = 9.3, 4.2 Hz, 1H), 5.40 – 5.23 (m, 2H), 4.70 – 4.59 (m, 2H), 4.11 (qd, *J* = 9.5, 4.3 Hz, 1H), 3.83 (s, 3H), 2.36 (s, 3H). **$^{19}$F NMR** (376 MHz, $CDCl_3$) δ = -66.22 (d, *J* = 9.5 Hz, 3F). **$^{13}$C NMR** (101 MHz, $CDCl_3$) δ = 169.8, 166.3, 162.7, 139.0, 131.2, 130.4, 130.2, 130.0, 129.1, 129.0, 126.3, 125.9, 125.72 (q, *J* = 280.6 Hz), 119.8, 114.0, 67.0, 55.5, 51.76 – 50.78 (m, 2C), 21.5. **HPLC**: Chiralpak AD-H (*n*-hexane/iPrOH 80:20, flow-rate 0.75 mL/min; $t_{major}$ = 10.6 min; $t_{minor}$ = 17.6 min).

**Allyl (2*S*,3*R*)-4,4,4-trifluoro-2-(4-methoxybenzamido)-3-(m-tolyl)butanoate *syn*-4g.**

Following general procedure A from azlactone **1g**, cat. **2p**, allyl alcohol, and cat. **dhQN-10**,

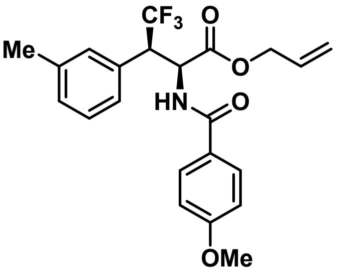

product *syn*-**4g** was obtained as a white solid in 84% yield (d.r. of the crude = 8.5:1) and 99% ee after column chromatography on silica gel ($CH_2Cl_2/Et_2O$ = 100:1). **$^1$H NMR** (400 MHz, $CDCl_3$) [signals of the *syn* isomer] δ = 7.78 – 7.71 (m, 2H), 7.23 – 7.11 (m, 4H), 6.97 – 6.90 (m, 2H), 6.68 (d, *J* = 9.0 Hz, 1H), 5.69 (ddt, *J* = 17.2, 10.4, 6.0 Hz, 1H), 5.45 (dd, *J* = 9.0, 7.2 Hz, 1H), 5.25 – 5.15 (m, 2H), 4.56 – 4.44 (m, 2H), 3.99 (qd, *J* = 9.5, 7.0 Hz, 1H), 3.84 (s, 3H), 2.31 (s, 3H). **$^{19}$F NMR** (376 MHz, $CDCl_3$) δ [signals of the *syn* isomer] = -69.21 (d, *J* = 9.5 Hz, 3F). **$^{13}$C NMR** (101 MHz, $CDCl_3$) [signals of the *syn* isomer] δ = 169.8, 166.6, 162.8, 138.6, 131.1, 131.0, 130.2, 129.9, 129.1, 128.8, 126.5, 126.0 (q, $^1J_{C\text{-}F}$ = 281.5 Hz), 125.8, 119.3, 114.0, 66.7, 55.5, 53.2, 51.8 (q, $^2J_{C\text{-}F}$ = 26.3 Hz), 21.5. **HPLC** [peaks of the *syn* isomer]: Chiralpak AD-H (*n*-hexane/iPrOH 80:20, flow-rate 0.75 mL/min; $t_{major}$ = 25.7 min; $t_{minor}$ = 12.4 min).

**HRMS** [*syn*-**4g** and *anti*-**4g** isomers]: calculated for $C_{22}H_{23}F_3NO_4$ [M + $H^+$] = 422.1529; found: 422.1574.

**Allyl (2*R*,3*R*)-4,4,4-trifluoro-3-(4-fluorophenyl)-2-(4-methoxybenzamido)butanoate** *anti*-**4h**.

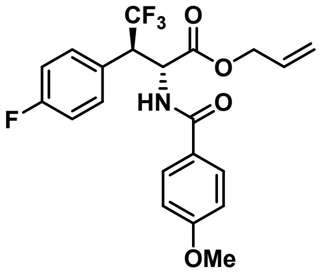

Following general procedure A from azlactone **1h**, cat. **2p**, allyl alcohol, and cat. **dhQD-2**, product *anti*-**4h** was obtained as a white solid in 88% yield (d.r. of the crude > 20:1) and 98% ee after column chromatography on silica gel ($CH_2Cl_2/Et_2O$ = 100:1). $[\alpha]_D^{25}$ = -29 (c = 0.27 in $CHCl_3$). **$^1$H NMR** (400 MHz, $CDCl_3$) δ = 7.70 – 7.58 (m, 2H), 7.47 – 7.29 (m, 2H), 7.16 – 7.05 (m, 2H), 6.94 – 6.86 (m, 2H), 6.32 (d, *J* = 9.2 Hz, 1H), 5.89 (ddt, *J* = 17.2, 10.3, 6.0 Hz, 1H), 5.63 (dd, *J* = 9.2, 4.7 Hz, 1H), 5.41 – 5.24 (m, 2H), 4.71 – 4.58 (m, 2H), 4.13 (qd, *J* = 9.3, 4.7 Hz, 1H), 3.83 (s, 3H). **$^{19}$F NMR** (376 MHz, $CDCl_3$) δ = -66.52 (d, *J* = 9.2 Hz, 3F), -112.03 - -112.12 (m, 1F). **$^{13}$C NMR** (101 MHz, $CDCl_3$) δ = 169.8, 166.4, 163.2 (d, $^1J_{C-F}$ = 249.2 Hz), 162.8, 131.3 (d, $^3J_{C-F}$ = 8.4 Hz), 131.0, 129.0, 126.5 (br s), 125.7, 125.6 (q, $^1J_{C-F}$ = 280.8 Hz), 120.0, 116.3 (d, $^2J_{C-F}$ = 21.5 Hz), 114.0, 67.1, 55.5, 51.5 (br s), 51.0 (q, $^2J_{C-F}$ = 27.8 Hz). **HPLC**: Chiralpak AD-H (*n*-hexane/iPrOH 80:20, flow-rate 0.75 mL/min; $t_{major}$ = 11.0 min; $t_{minor}$ = 25.3 min).

**Allyl (2*S*,3*R*)-4,4,4-trifluoro-3-(4-fluorophenyl)-2-(4-methoxybenzamido)butanoate** *syn*-**4h**.

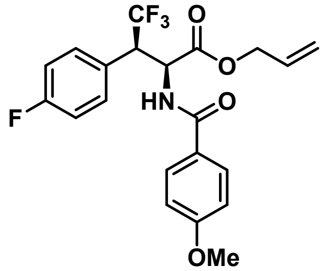

Following general procedure A from azlactone **1h**, cat. **2p**, allyl alcohol, and cat. **dhQN-10**, product *syn*-**4h** was obtained as a white solid in 78% yield (d.r. of the crude = 4.4:1) and 99% ee after column chromatography on silica gel ($CH_2Cl_2/Et_2O$ = 100:1). In order to reach full conversion of intermediate **3h**, further 5 mol% of cat **dhQN-10** and 1 equivalent of allyl alcohol were added after 4 days (6 days of reaction time). **$^1$H NMR** (400 MHz, $CDCl_3$) [signals of the *syn* isomer] δ = 7.76 – 7.70 (m, 2H), 7.37 – 7.28 (m, 2H), 7.08 – 6.98 (m, 2H), 6.97 – 6.91 (m, 2H), 6.69 (d, *J* = 9.0 Hz, 1H), 5.73 (ddt, *J* = 17.2, 10.3, 6.0 Hz, 1H), 5.44 (dd, *J* = 9.0, 6.9 Hz, 1H), 5.27 – 5.14 (m, 2H), 4.57 – 4.47 (m, 2H), 4.07 (qd, *J* = 9.4, 6.9 Hz, 1H), 3.84 (s, 3H). **$^{19}$F NMR** (376 MHz, $CDCl_3$) [signals of the *syn* isomer] δ = -64.42 (d, *J* = 9.3 Hz, 3F), -112.36 - -112.46 (m, 1F). **$^{13}$C NMR** (101 MHz, $CDCl_3$) δ [signals of the *syn* isomer] = 169.2, 166.6, 163.1 (d, $^1J_{C-F}$ = 248.8 Hz), 162.9, 131.3 (d, $^3J_{C-F}$ = 8.4 Hz), 130.9, 129.1, 127.2 (br s), 125.9 (q, $^1J_{C-F}$ = 281.0 Hz), 125.6, 119.6, 116.02 (d, $^2J_{C-F}$ = 21.6 Hz), 114.1, 66.8, 55.9, 53.0 (br s), 51.0 (q, $^3J_{C-F}$ = 26.8 Hz). **HPLC** [peaks of the *syn* isomer]: Chiralpak AD-H (*n*-hexane/iPrOH 80:20, flow-rate 0.75 mL/min; $t_{major}$ = 19.4 min; $t_{minor}$ = 16.8 min).

**HRMS** [*syn*-**4h** and *anti*-**4h** isomers]: calculated for $C_{21}H_{20}F_4NO_4$ [M + H$^+$] = 426.1328; found: 426.1325.

**Allyl (2*R*,3*R*)-4,4,4-trifluoro-2-(4-methoxybenzamido)-3-(4-methoxyphenyl)butanoate *anti*-4i.**

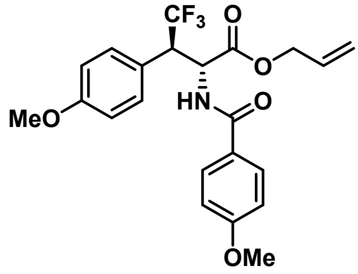


Following general procedure A from azlactone **1i**, cat. **2p**, allyl alcohol, and cat. **dhQD-2**, product *anti*-**4i** was obtained as a white solid in 90% yield (d.r. of the crude > 20:1) and 98% ee after column chromatography on silica gel ($CH_2Cl_2/Et_2O$ = 100:1). **[α]$_D$$^{25}$** = -37 (c = 0.27 in $CHCl_3$). In order to reach full conversion of intermediate **3i** the reaction was moved, after 2 days, from 0°C to r.t., then, after 3 days, additional 5 mol% of cat. **dhQD-2** and 1 equivalent of allyl alcohol were added (6 days of reaction time). **$^1$H NMR** (400 MHz, $CDCl_3$) δ = 7.69 – 7.62 (m, 2H), 7.33 – 7.24 (m, 2H), 6.96 – 6.87 (m, 4H), 6.32 (d, *J* = 9.3 Hz, 1H), 5.90 (ddt, *J* = 17.2, 10.4, 6.0 Hz, 1H), 5.63 (dd, *J* = 9.2, 4.4 Hz, 1H), 5.39 – 5.23 (m, 2H), 4.69 – 4.58 (m, 2H), 4.09 (qd, *J* = 9.4, 4.4 Hz, 1H), 3.83 (s, 3H), 3.81 (s, 3H). **$^{19}$F NMR** (376 MHz, $CDCl_3$) δ = -66.67 (d, *J* = 9.4 Hz, 3F). **$^{13}$C NMR** (101 MHz, $CDCl_3$) δ = 169.9, 166.4, 162.7, 160.2, 131.1, 130.6, 129.0, 125.85 (q, $^1J_{C-F}$ = 280.5 Hz), 125.85, 122.3, 119.8, 114.6, 114.0, 66.9, 55.5, 55.4, 51.5, 50.75 (q, $^2J_{C-F}$ = 27.5 Hz). **HPLC**: Chiralpak AD-H (*n*-hexane/iPrOH 80:20, flow-rate 0.75 mL/min; $t_{major}$ = 17.1 min; $t_{minor}$ = 35.1 min).

**Allyl (2S,3*R*)-4,4,4-trifluoro-2-(4-methoxybenzamido)-3-(4-methoxyphenyl)butanoate *syn*-4i.**

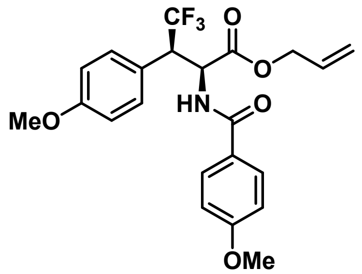


Following general procedure A from azlactone **1i**, cat. **2p**, allyl alcohol, and cat. **dhQN-10**, product *syn*-**4i** was obtained as a white solid in 60% yield (d.r. of the crude 3.7:1) and >99% ee after column chromatography on silica gel ($CH_2Cl_2/Et_2O$ = 100:1). In order to reach full conversion of intermediate **3i**, the reaction was moved, after 2 days, from 0°C to r.t., then, after 3 days, additional 5 mol% of cat. **dhQN-10** and 1 equivalent of allyl alcohol were added (8 days of reaction time). **$^1$H NMR** (400 MHz, $CDCl_3$) [signals of the *syn* isomer] δ = 7.76 – 7.70 (m, 2H), 7.30 – 7.20 (m, 2H), 6.98 – 6.89 (m, 2H), 6.88 – 6.84 (m, 2H), 6.64 (d, *J* = 9.0 Hz, 1H), 5.73 (ddt, *J* = 17.3, 10.4, 6.0 Hz, 1H), 5.43 (dd, *J* = 9.0, 6.9 Hz, 1H), 5.31 – 5.14 (m, 2H),

4.60 – 4.45 (m, 2H), 4.00 (qd, *J* = 9.5, 6.9 Hz, 1H), 3.85 (s, 3H), 3.78 (s, 3H). $^{19}$**F NMR** (376 MHz, $CDCl_3$) [signals of the *syn* isomer] δ = -64.51 (d, *J* = 9.4 Hz, 3F). $^{13}$**C NMR** (101 MHz, $CDCl_3$) [signals of the *syn* isomer] δ = 169.8, 166.6, 162.8, 160.1, 131.1, 130.7, 129.1, 126.1 (q, $^{1}J_{C\text{-}F}$ = 281.1 Hz), 125.8, 123.1, 119.4, 114.4, 114.0, 66.7, 55.6, 55.3, 53.2, 51.1 (q, $^{2}J_{C\text{-}F}$ = 27.1, 26.5 Hz). **HPLC** [peaks of the *syn* isomer]: Chiralpak AD-H (*n*-hexane/iPrOH 80:20, flow-rate 0.75 mL/min; $t_{major}$ = 23.3 min; $t_{minor}$ = 27.8 min).

**HRMS** [*syn*-**4i** and *anti*-**4i** isomers]: calculated for $C_{22}H_{22}F_3NNaO_5$ [M + Na$^+$] = 460.1348; found: 460.1343.

**Allyl (2*R*,3*R*)-3-(3,5-difluorophenyl)-4,4,4-trifluoro-2-(4-methoxybenzamido)butanoate** *anti*-**4j**.

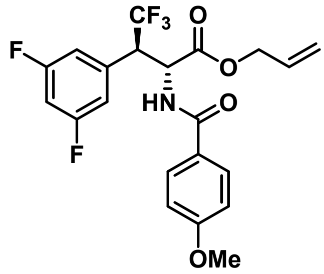


Following general procedure A from azlactone **1j**, cat. **2p**, allyl alcohol, and cat. **dhQD-2**, product *anti*-**4j** was obtained as a white solid in 85% yield (d.r. of the crude > 20:1) and 99% ee after column chromatography on silica gel ($CH_2Cl_2/Et_2O$ = 100:1). **[α]$_D^{25}$** = -13 (c = 0.27 in $CHCl_3$). $^{1}$**H NMR** (400 MHz, $CDCl_3$) δ = 7.74 – 7.62 (m, 2H), 7.00 – 6.89 (m, 5H), 6.85 (tt, *J* = 8.7, 2.3 Hz, 1H), 6.45 (d, *J* = 9.0 Hz, 1H), 5.89 (ddt, *J* = 17.2, 10.3, 6.1 Hz, 1H), 5.62 (dd, *J* = 9.0, 4.4 Hz, 1H), 5.41 – 5.26 (m, 2H), 4.73 – 4.58 (m, 2H), 4.13 (qd, *J* = 9.1, 4.5 Hz, 1H), 3.84 (s, 3H). $^{19}$**F NMR** (376 MHz, $CDCl_3$) δ = -66.09 (d, *J* = 9.1 Hz, 3F), -107.80 – -107.89 (m, 2F). $^{13}$**C NMR** (101 MHz, $CDCl_3$) δ = 169.4, 166.5, 163.2 (dd, $^{1}J_{C\text{-}F}$ = 250.3, $^{3}J_{C\text{-}F}$ = 12.9 Hz), 162.9, 134.3 (t, $^{3}J_{C\text{-}F}$ = 9.5 Hz), 130.8, 129.1, 125.5, 125.3 (q, $^{1}J_{C\text{-}F}$ = 280.9 Hz),120.5, 114.1, 113.20 – 112.54 (m), 104.94 (t, $^{2}J_{C\text{-}F}$ = 25.0 Hz), 67.3, 55.6, 51.8 – 50.7 (m, 2C). **HPLC**: Chiralpak AD-H (*n*-hexane/iPrOH 95:5, flow-rate 0.75 mL/min; $t_{major}$ = 24.7 min; $t_{minor}$ = 46.5 min).

**Allyl (2*S*,3*R*)-3-(3,5-difluorophenyl)-4,4,4-trifluoro-2-(4-methoxybenzamido)butanoate** *syn*-**4j**.

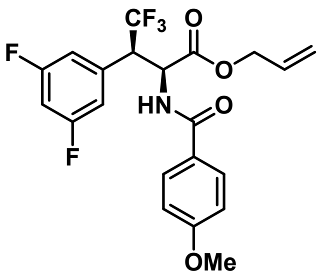

Following general procedure A from azlactone **1j**, cat. **2p**, allyl alcohol, and cat. **dhQN-10**, product *syn*-**4j** was obtained as a white solid in 87% yield (d.r. of the crude = 4.0:1) and >99% ee after column chromatography on silica gel ($CH_2Cl_2/Et_2O$ = 100:1). In order to reach full conversion of the intermediate **3j**, additional 5 mol% of cat **dhQN-10** and 1 equivalent of allyl alcohol were added after 6 days (8 days of reaction time). **$^1$H NMR** (400 MHz, $CDCl_3$) [signals of the *syn* isomer] δ = 7.76 – 7.71 (m, 2H), 6.97 – 6.89 (m, 4H), 6.81 (tt, *J* = 8.7, 2.3 Hz, 1H), 6.71 (d, *J* = 9.0 Hz, 1H), 5.77 (ddt, *J* = 16.4, 10.3, 6.1 Hz, 1H), 5.43 (dd, *J* = 9.0, 6.5 Hz, 1H), 5.34 – 5.21 (m, 2H), 4.59 – 4.54 (m, 2H), 4.09 (qd, *J* = 9.1, 6.2 Hz, 1H), 3.85 (s, 3H). **$^{19}$F NMR** (376 MHz, $CDCl_3$) [signals of the *syn* isomer] δ = -64.14 (d, *J* = 9.1 Hz, 3F), -108.25 – -108.36 (m, 2F). **$^{13}$C NMR** (101 MHz, $CDCl_3$) [signals of the *syn* isomer] δ = 169.2, 166.8, 163.1 (dd, $^1J_{C\text{-}F}$ = 249.8, $^3J_{C\text{-}F}$ = 12.9 Hz),163.0, 134.8 (t, $^3J_{C\text{-}F}$ = 9.6 Hz), 130.8, 129.2, 125.5, 125.4 (q, $^1J_{C\text{-}F}$ = 281.3 Hz), 120.0, 114.1, 113.0 – 112.6 (m), 104.8 (t, $^2J_{C\text{-}F}$ = 25.0 Hz), 67.1, 55.6, 52.8. 51.6 – 50.7 (m). **HPLC** [peaks of the *syn* isomer]: Chiralpak AD-H (*n*-hexane/iPrOH 95:5, flow-rate 0.75 mL/min; $t_{major}$ = 83.9 min; $t_{minor}$ = 57.9 min).

**HRMS** [*syn*-**4j** and *anti*-**4j** isomers]: calculated for $C_{21}H_{19}F_5NO_4$ [M + H$^+$] = 444.1234; found: 444.1232.

**Allyl (2*R*,3*R*)-4,4,4-trifluoro-2-(4-methoxybenzamido)-3-(thiophen-2-yl)butanoate** *anti*-**4k**.

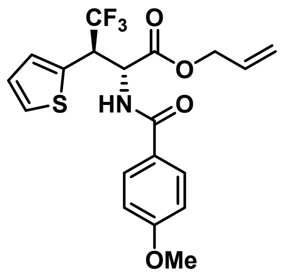

Following general procedure A from azlactone **1k**, cat. **2p**, allyl alcohol, and cat. **dhQD-2**, product *anti*-**4k** was obtained as a white solid in 78% yield (d.r. of the crude > 20:1) and 97% ee after column chromatography on silica gel ($CH_2Cl_2/Et_2O$ = 100:1). **$[\alpha]_D^{25}$** = -30 (c = 0.27 in $CHCl_3$). **$^1$H NMR** (400 MHz, $CDCl_3$) δ = 7.85 – 7.70 (m, 2H), 7.38 (dd, *J* = 5.1, 1.3, Hz, 1H), 7.16 – 7.02 (m, 2H), 6.99 – 6.90 (m, 2H), 6.50 (d, *J* = 9.4 Hz, 1H), 5.88 (ddt, *J* = 17.2, 10.3, 6.0 Hz, 1H), 5.73 (dd, *J* = 9.4, 3.0 Hz, 1H), 5.39 – 5.26 (m, 2H), 4.63 (dt, *J* = 6.0, 1.3 Hz, 2H), 4.54 (qd, *J* = 8.9, 3.0 Hz, 1H), 3.85 (s, 3H). **$^{19}$F NMR** (376 MHz, $CDCl_3$) δ = -67.90 (d, *J* = 8.9 Hz, 3F). **$^{13}$C NMR** (101 MHz,

$CDCl_3$) δ = 169.0, 166.4, 162.7, 130.9, 130.3, 129.2, 129.1, 127.4, 127.1, 125.7, 124.9 (q, $^1J_{C-F}$ = 280.8 Hz), 119.9, 113.9, 67.0, 55.4, 51.3, 47.10 (q, $^2J_{C-F}$ = 28.8 Hz). **HPLC**: Chiralcel OD-H (*n*-hexane/iPrOH 90:10, flow-rate 0.75 mL/min; $t_{major}$ = 14.9 min; $t_{minor}$ = 42.7 min).

**Allyl (2*S*,3*R*)-4,4,4-trifluoro-2-(4-methoxybenzamido)-3-(thiophen-2-yl)butanoate *syn*-4k**.

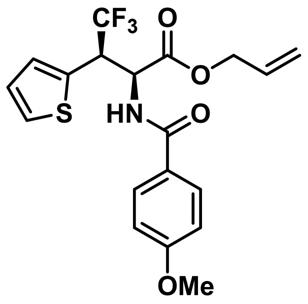


Following general procedure B from azlactone **1k**, cat. **2p**, allyl alcohol, and cat. **dhQN-10**, product *syn*-**4k** was obtained as a white solid in 37% yield (d.r. of the crude = 2.1:1) and 99% ee after column chromatography on silica gel ($CH_2Cl_2/Et_2O$ = 100:1). **$^1$H NMR** (400 MHz, $CDCl_3$) [signals of the *syn* isomer] δ = 7.78 – 7.74 (m, 2H), 7.30 (ddd, *J* = 5.1, 1.2, 0.4 Hz, 1H), 7.10 (dd, *J* = 3.6, 1.2 Hz, 1H), 6.99 (dd, *J* = 5.2, 3.6 Hz, 1H), 6.95 (dd, *J* = 8.9, 1.4 Hz, 2H), 6.64 (d, *J* = 9.3 Hz, 1H), 5.92 – 5.82 (m, 1H), 5.45 (dd, *J* = 9.3, 4.6 Hz, 1H), 5.38 – 5.25 (m, 2H), 4.70 – 4.64 (m, 2H), 4.59 – 4.49 (m, 1H), 3.86 (s, 3H). **$^{19}$F NMR** (376 MHz, $CDCl_3$) [signals of the *syn* isomer] δ = -65.50 (d, *J* = 9.1 Hz, 3F). **$^{13}$C NMR** (101 MHz, $CDCl_3$) [signals of the *syn* isomer] δ = 169.1, 166.9, 162.9, 131.6, 131.1, 129.2, 129.1, 127.1, 127.0, 125.8, 125.4 (q, $^1J_{C-F}$ = 281.3 Hz), 119.8, 114.1, 67.1, 55.6, 53.1, 47.1 (q, $^2J_{C-F}$ = 27.8 Hz). **HPLC** [peaks of the *syn* isomer]: Chiralcel OD-H (*n*-hexane/iPrOH 90:10, flow-rate 0.75 mL/min; $t_{major}$ = 52.4 min; $t_{minor}$ = 16.4 min).

**HRMS** [*syn*-**4k** and *anti*-**4k** isomers]: calculated for $C_{19}H_{19}F_3NO_4S$ [M + H$^+$] = 414.0987; found: 414.0982.

**Allyl (2*R*,3*R*)-4,4,4-trifluoro-2-(4-methoxybenzamido)-3-(1-tosyl-1*H*-indol-3-yl)butanoate *anti*-4l**.

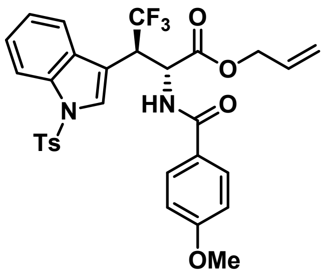


Following general procedure A from azlactone **1l**, cat. **2p**, allyl alcohol, and cat. **dhQD-2**, product *anti*-**4l** was obtained as a white solid in 82% yield (d.r. of the crude > 20:1) and 96% ee after column chromatography on silica gel ($CH_2Cl_2/Et_2O$ = 100:1). **$[\alpha]_D^{25}$** = -42 (c = 0.27 in $CHCl_3$). **$^1$H NMR** (400 MHz, $CDCl_3$) δ = 7.98 (d, *J* = 8.2 Hz, 1H), 7.78 – 7.69 (m, 5H), 7.66 (d, *J* = 8.2 Hz, 1H), 7.41 – 7.32 (m, 1H), 7.33 – 7.25 (m, 1H), 7.17 (d, *J* = 7.9 Hz, 2H), 6.97 – 6.90 (m, 2H), 6.58 (d, *J* = 8.8 Hz, 1H), 5.79 (ddt, *J* = 17.2, 10.3, 6.1 Hz, 1H), 5.63 (dd, *J* = 8.8, 3.5 Hz, 1H),

5.31 – 5.20 (m, 2H), 4.60 – 4.45 (m, 3H), 3.85 (s, 3H), 2.31 (s, 3H). **$^{19}$F NMR** (376 MHz, $CDCl_3$) δ = -66.91 (d, $J$ = 9.2 Hz, 3F). **$^{13}$C NMR** (101 MHz, $CDCl_3$) δ = 169.3, 166.5, 162.9, 145.5, 134.9, 134.7, 130.8, 130.1, 129.8, 129.1, 126.9, 125.8, 125.71 (q, $^{1}J_{C-F}$ = 280.8 Hz), 125.70, 125.6, 123.8, 120.2, 119.4, 114.1, 113.8, 111.6, 67.2, 55.6, 51.0, 43.3 (q, $^{2}J_{C-F}$ = 28.4 Hz), 21.7. **HPLC**: Chiralpak IA (*n*-hexane/iPrOH 75:25, flow-rate 1.0 mL/min; $t_{major}$ = 8.8 min; $t_{minor}$ = 12.3 min).

**Allyl (2*S*,3*R*)-4,4,4-trifluoro-2-(4-methoxybenzamido)-3-(1-tosyl-1*H*-indol-3-yl)butanoate *syn*-4l.**

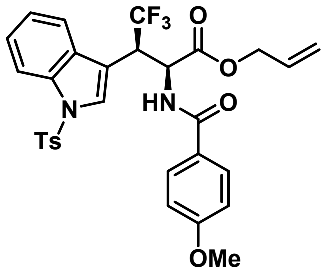

Following general procedure B from azlactone **1l**, cat. **2p**, allyl alcohol, and cat. **dhQN-10**, product *syn*-**4l** was obtained as a white solid in 53% yield (d.r. of the crude = 2.3:1) and >99% ee after column chromatography on silica gel (**$^{1}$H NMR** (400 MHz, $CDCl_3$) [signals of the *syn* isomer] δ = 7.93 (d, $J$ = 8.3, 1H), 7.75 – 7.63 (m, 5H), 7.50 (d, $J$ = 7.9, Hz, 1H), 7.35 – 7.29 (m, 1H), 7.27 – 7.19 (m, 1H), 7.16 – 7.12 (m, 2H), 6.96 – 6.89 (m, 2H), 6.60 (d, $J$ = 8.9 Hz, 1H), 5.86 – 5.72 (m, 1H), 5.46 (dd, $J$ = 9.0, 5.0 Hz, 1H), 5.33 – 5.20 (m, 2H), 4.66 – 4.44 (m, 3H), 3.85 (s, 3H), 2.30 (s, 3H). **$^{19}$F NMR** (376 MHz, $CDCl_3$) [signals of the *syn* isomer] δ = -65.00 (d, $J$ = 9.2 Hz, 3F). **$^{13}$C NMR** (101 MHz, $CDCl_3$) [signals of the *syn* isomer] δ = 169.3, 166.8, 162.9, 145.3, 134.9, 134.5, 130.9, 130.04, 129.99, 129.1, 127.0, 125.9 (q, $^{1}J_{C-F}$ = 280.9 Hz), 125.8, 125.6, 125.5, 123.8, 120.0, 118.9, 114.1, 112.2, 67.1, 55.6, 52.3, 42.9 (q, $^{2}J_{C-F}$ = 27.6 Hz), 21.7. **HPLC** [peaks of the *syn* isomer]: Chiralpak IA (*n*-hexane/iPrOH 75:25, flow-rate 1.0 mL/min; $t_{major}$ = 34.9 min; $t_{minor}$ = 14.1 min).

**HRMS** [*syn*-**4l** and *anti*-**4l** isomers]: calculated for $C_{30}H_{27}F_3N_2NaO_6S$ [M + Na$^+$] = 623.1440; found: 623.1432.

**Allyl (2*R*,3*R*)-4,4,5,5,6,6,6-heptafluoro-2-(4-methoxybenzamido)-3-phenylhexanoate *anti*-4m.**

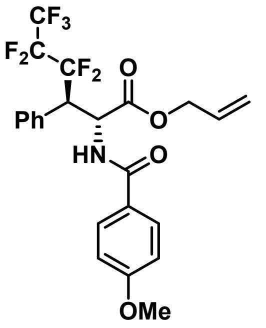

Following general procedure A from azlactone **1m**, cat. **2p**, allyl alcohol, and cat. **dhQD-2**, product *anti*-**4m** was obtained as a white solid in 69% yield (d.r. of the crude > 20:1) and 98% ee after column chromatography on silica gel ($CH_2Cl_2$). **$[\alpha]_D^{25}$** = -25 (c = 0.27 in $CHCl_3$). **$^1$H NMR** (400 MHz, $CDCl_3$) δ = 7.69 – 7.61 (m, 2H), 7.41 – 7.34 (m, 5H), 7.02 – 6.81 (m, 2H), 6.30 (d, *J* = 9.6 Hz, 1H), 5.94 – 5.77 (m, 2H), 5.39 – 5.25 (m, 2H), 4.66 – 4.57 (m, 2H), 4.30 – 4.16 (m, 1H), 3.83 (s, 3H). **$^{19}$F NMR** (376 MHz, $CDCl_3$) δ = -80.50 (dd, *J* = 12.9, 8.8 Hz, 3F), -107.22 – -109.99 (m, 1F), -117.82 (dddt, *J* = 280.5, 25.8, 17.5, 9.2 Hz, 1F), -122.21 (ddd, *J* = 289.2, 14.3, 10.3 Hz, 1F), -125.28 (dd, *J* = 289.2, 15.3 Hz, 1F). **$^{13}$C NMR** (101 MHz, $CDCl_3$) [$^{13}$C signals of the perfluoroalkyl chain not reported as they were weakly detected due to their multiplicity] δ = 169.8, 166.2, 162.8, 131.1, 130.03, 129.96, 129.4, 129.2, 129.0, 125.9, 119.8, 114.0, 67.0, 55.5, 50.7, 48.6 (t, $^2J_{C-F}$ = 20.0 Hz). **HPLC**: Chiralcel OD-H (*n*-hexane/iPrOH 95:5, flow-rate 0.75 mL/min; $t_{major}$ = 12.7 min; $t_{minor}$ = 20.2 min).

**Allyl (2*S*,3*R*)-4,4,5,5,6,6,6-heptafluoro-2-(4-methoxybenzamido)-3-phenylhexanoate** *syn*-**4m**.

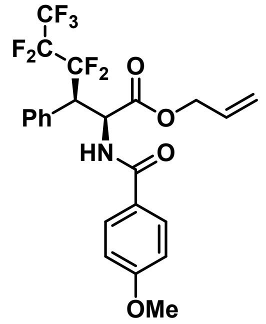

Following general procedure B, but carrying out the hydrogen transfer reaction at 0 °C, from azlactone **1m**, cat. **2p**, allyl alcohol, and cat. **dhQN-10**, product *syn*-**4m** was obtained as a white solid in 50% yield (d.r. of the crude = 2.0:1) and >99% ee after column chromatography on silica gel ($CH_2Cl_2$). **$^1$H NMR** (600 MHz, $CDCl_3$) [signals of the *syn* isomer] δ = 7.94-7.71 (m, 2H), 7.34-7.30 (m, 5H), 6.95-6.93 (m, 2H), 6.59 (d, *J* = 9.0 Hz, 1H), 5.92-5.82 (m, 1H), 5.53 (dd, *J* = 8.9, 5.4 Hz, 1H), 5.35-5.22 (m, 2H), 4.65-4.54 (m, 2H), 4.29-4.18 (m, 1H), 3.85 (s, 3H). **$^{19}$F NMR** (376 MHz, $CDCl_3$) [signals of the *syn* isomer] δ = -80.39 (dd, *J* = 12.7, 9.1 Hz, 3F), -107.50 – -108.49 (m, 1F), -114.08 – -115.29 (m, 1F), -122.33 (ddd, *J* = 289.7, 14.2, 10.8 Hz, 1F), -125.27 (br dd, *J* = 289.2, 15.6 Hz, 1F). **$^{13}$C NMR** (150 MHz, $CDCl_3$) [signals of the *syn* isomer, $^{13}$C signals of the perfluoroalkyl chain not reported as they were weakly detected due to their multiplicity] δ = 169.6, 166.3, 162.7, 130.8, 130.6-130.5 (m), 130.0 (br), 129.1, 129.0, 128.7, 125.6, 119.5, 113.9, 66.8, 55.4, 53.0-52.8 (m), 48.9 (t, $^2J_{C-F}$ = 19.4 Hz). **HPLC** [peaks of the

*syn* isomer]: Chiralcel OD-H (*n*-hexane/iPrOH 95:5, flow-rate 0.75 mL/min; $t_{major}$ = 62.8 min; $t_{minor}$ = 14.0 min).

**HRMS** [*syn*-**4m** and *anti*-**4m** isomers]: calculated for $C_{23}H_{21}F_7NO_4$ [M + H$^+$] = 508.1359; found: 508.1355.

**Allyl (2*R*,3*R*)-3-cyclohexyl-4,4,4-trifluoro-2-(4-methoxybenzamido)butanoate *anti*-4n.**

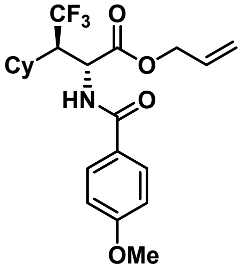


Following general procedure C from azlactone **1n**, cat. **2p**, allyl alcohol, and cat. **dhQD-2**, product *anti*-**4n** was obtained as a white waxy solid in 98% yield (d.r. of the crude = 16.7:1) and 99% ee after column chromatography on silica gel ($CH_2Cl_2/Et_2O$ = 200:1). In order to reach full conversion of intermediate **3n**, further 10 mol% of cat **dhQD-2** and 2 equivalents of allyl alcohol were added after 3 days (6 days of reaction time). **$^1$H NMR** (400 MHz, $CDCl_3$) [signals of the *anti*-isomer] δ = 7.79 – 7.72 (m, 2H), 6.98 – 6.92 (m, 2H), 6.79 (d, *J* = 8.4 Hz, 1H), 5.93 (ddt, *J* = 17.2, 10.4, 5.9 Hz, 1H), 5.44 – 5.28 (m, 3H), 4.72 – 4.66 (m, 2H), 3.86 (s, 3H), 2.63 (qdd, *J* = 10.0, 7.0, 3.3 Hz, 1H), 2.11 – 2.01 (m, 1H), 1.97 – 1.88 (m, 2H), 1.86 – 1.75 (m, 2H), 1.71 – 1.64 (m, 1H), 1.40 – 1.16 (m, 5H).**$^{19}$F NMR** (376 MHz, $CDCl_3$) [signal of the *anti*-isomer] δ = -62.12 (d, *J* = 9.7 Hz, 3F). **$^{13}$C NMR** (101 MHz, $CDCl_3$) [signals of the *anti*-isomer] δ = 170.1, 166.2, 162.8, 131.2, 129.1, 127.2 (q, $^1J_{C\text{-}F}$ = 283.9 Hz), 126.0, 119.6, 114.1, 66.9, 55.6, 52.4 (q, $^2J_{C\text{-}F}$ = 23.1 Hz), 50.3 (br s), 36.2, 31.8, 29.8, 26.7, 26.5, 26.1. **HPLC** [peaks of the *anti*-isomer]: Chiralpak AD-H (*n*-hexane/iPrOH 90:10, flow-rate 0.75 mL/min; $t_{major}$ = 14.1 min; $t_{minor}$ = 24.7 min).

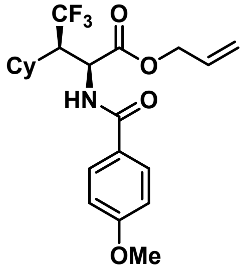


**Allyl (2*S*,3*R*)-3-cyclohexyl-4,4,4-trifluoro-2-(4-methoxybenzamido)butanoate *syn*-4n.**

Following general procedure C from azlactone **1n**, cat. **2p**, allyl alcohol, and cat. **dhQN-10**, product *syn*-**4n** was obtained as a colourless oil in 65% yield (d.r. of the crude = 3.6:1) and >99% ee after column chromatography on silica gel ($CH_2Cl_2/Et_2O$ = 200:1). In order to reach full conversion of the intermediate **3n**, additional 10 mol% of cat **dhQN-10** and 2 equivalents of allyl alcohol were added after 3 days, and the reaction was left stirring at RT for additional 5 days (8 days of reaction time). **$^1$H NMR** (400 MHz, $CDCl_3$) [signals of the *syn* isomer] δ = 7.79-7.74 (m, 2H), 6.98-6.93 (m, 2H), 6.43 (d, *J* = 9.9 Hz,

1H), 5.94-5.84 (m, 1H), 5.40-5.28 (m, 2H), 5.26 (dq, $J$ = 10.5, 1.2 Hz, 1H), 4.66 (dt, $J$ = 5.8, 1.3 Hz, 2H), 3.86 (s, 3H), 2.97-2.85 (m, 1H), 2.11-2.01 (m, 1H), 1.98-1.61 (m, 5H), 1.39-1.11 (m, 5H). **$^{19}$F NMR** (376 MHz, $CDCl_3$) [signal of the *syn* isomer] δ = -60.50 (d, $J$ = 9.9 Hz, 3F). **$^{13}$C NMR** (101 MHz, $CDCl_3$) [signals of the *syn* isomer] δ = 170.8, 167.2, 162.7, 131.3, 129.0, 127.4 (q, $^1J_{C\text{-}F}$ = 284.3 Hz), 125.9, 119.2, 114.0, 66.7, 55.5, 50.9 (q, $^2J_{C\text{-}F}$ = 22.9 Hz), 49.3 (br s), 35.7, 30.9, 30.6, 26.3, 26.2, 25.9. **HPLC** [peaks of the *syn* isomer]: Chiralpak AD-H (*n*-hexane/iPrOH 90:10, flow-rate 0.75 mL/min; $t_{major}$ = 16.7 min; $t_{minor}$ = 19.8 min).

**HRMS** [*syn*-**4n** and *anti*-**4n** isomers]: calculated for $C_{21}H_{27}F_3NO_4$ [M + H$^+$] = 414.1887; found: 444.1879.

**Allyl (2*R*,3*R*)-2-(4-methoxybenzamido)-3-(trifluoromethyl)pentanoate** *anti*-**4o**.

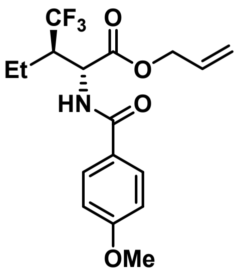

Following general procedure C from azlactone **1o**, cat. **2p**, allyl alcohol, and cat. **dhQD-2**, product *anti*-**4o** was obtained as a white waxy solid in 98% yield (d.r. of the crude = 15.3:1) and 97% ee after column chromatography on silica gel ($CH_2Cl_2/Et_2O$ = 100:1). **$^1$H NMR** (400 MHz, $CDCl_3$) [signals of the *anti* isomer] δ = 7.81 – 7.71 (m, 2H), 6.99 – 6.88 (m, 2H), 6.75 (d, $J$ = 8.3 Hz, 1H), 5.93 (ddt, $J$ = 17.2, 10.4, 6.0 Hz, 1H), 5.37 (dq, $J$ = 17.2, 1.4 Hz, 1H), 5.30 (dq, $J$ = 10.4, 1.2 Hz, 1H), 5.21 (dd, $J$ = 8.4, 3.0 Hz, 1H), 4.72 – 4.68 (m, 2H), 3.85 (s, 3H), 2.81 – 2.68 (m, 1H), 1.92 – 1.73 (m, 2H), 1.17 (d, $J$ = 7.6 Hz, 3H). **$^{19}$F NMR** (376 MHz, $CDCl_3$) [signal of the *anti* isomer] δ = -67.64 (d, $J$ = 9.0 Hz, 3F). **$^{13}$C NMR** (101 MHz, $CDCl_3$) [signals of the *anti* isomer] δ = 169.9, 166.4, 162.8, 131.2, 129.1, 127.1 (q, $^1J_{C\text{-}F}$ = 281.4 Hz), 126.0, 119.8, 114.1, 67.0, 55.6, 50.5, 47.9 (q, $^2J_{C\text{-}F}$ = 24.7 Hz), 18.8 (q, $^3J_{C\text{-}F}$ = 2.2 Hz), 12.0. **HPLC** [peaks of the *anti*-isomer]: Chiralpak AD-H (*n*-hexane/iPrOH 90:10, flow-rate 0.75 mL/min; $t_{major}$ = 18.6 min; $t_{minor}$ = 30.9 min).

**Allyl (2*S*,3*R*)-2-(4-methoxybenzamido)-3-(trifluoromethyl)pentanoate** *syn*-**4o**.

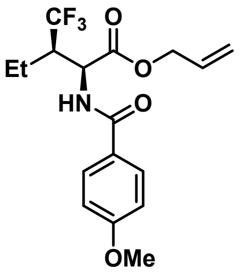

Following general procedure C from azlactone **1o**, cat. **2p**, allyl alcohol, and cat. **dhQN-10**, product *syn*-**4o** was obtained as a white waxy solid in 78% yield (d.r. of the crude = 2.6:1) and >99% ee after column chromatography on silica gel ($CH_2Cl_2/Et_2O$ = 100:1). In order to reach full conversion of the intermediate **3o**, additional 10 mol% of cat **dhQN-10** and 2 equivalents of allyl alcohol were added after 3 days (6 days of reaction time). **$^1$H NMR**

(400 MHz, $CDCl_3$) [signals of the *syn* isomer] δ = 7.81 – 7.75 (m, 2H), 6.98 – 6.93 (m, 2H), 6.49 (d, *J* = 9.6 Hz, 1H), 5.99 – 5.86 (m, 1H), 5.40 – 5.26 (m, 3H), 4.70 – 4.66 (m, 2H), 3.86 (s, 3H), 2.99 – 2.84 (m, 1H), 1.83 – 1.73 (m, 1H), 1.70 – 1.60 (m, 1H), 1.15 (t, *J* = 7.2 Hz, 3H). **$^{19}$F NMR** (376 MHz, $CDCl_3$) [signal of the *syn* isomer] δ = -66.07 (d, *J* = 9.1 Hz). **$^{13}$C NMR** (101 MHz, $CDCl_3$) [signals of the *syn* isomer] δ = 170.5, 167.4, 162.9, 131.4, 129.2, 127.4 (q, $^1J_{C\text{-}F}$ = 281.3 Hz) 125.9, 119.6, 114.1, 66.9, 55.6, 49.6, 47.7 (q, $^2J_{C\text{-}F}$ = 24.1 Hz), 18.8 (q, $^3J_{C\text{-}F}$ = 2.2 Hz), 11.7. **HPLC** [peaks of the *syn* isomer]: Chiralpak AD-H (*n*-hexane/iPrOH 90:10, flow-rate 0.75 mL/min; $t_{major}$ = 23.0 min; $t_{minor}$ = 16.6 min).

**HRMS** [*syn*-**4o** and *anti*-**4o** isomers]: calculated for $C_{17}H_{21}F_3NO_4$ [M + H$^+$] = 360.1417; found: 360.1410.

**Allyl (2*R*,3*R*)-4,4,4-trifluoro-2-(4-methoxybenzamido)-3-methylbutanoate *anti*-4p**.

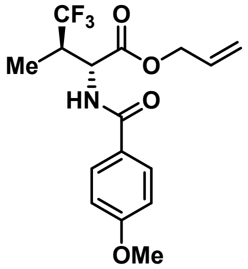


Following general procedure C from azlactone **1p**, cat. **2p**, allyl alcohol, and cat. **dhQD-2**, product *anti*-**4p** was obtained as in 97% yield (d.r. of the crude = 10.1:1) and 89% ee after column chromatography on silica gel ($CH_2Cl_2/Et_2O$ = 100:1). **$^1$H NMR** (400 MHz, $CDCl_3$) [signals of the *anti* isomer] δ = 7.81 – 7.73 (m, 2H), 6.97 – 6.91 (m, 2H), 6.63 (d, *J* = 8.5 Hz, 1H), 5.92 (ddt, *J* = 17.2, 10.4, 6.0 Hz, 1H), 5.37 (dq, *J* = 17.2, 1.4 Hz, 1H), 5.30 (dq, *J* = 10.4, 1.1 Hz, 1H), 5.19 (dd, *J* = 8.6, 3.4 Hz, 1H), 4.72 – 4.68 (m, 2H), 3.85 (s, 3H), 3.01 – 2.90 (m, 1H), 1.30 (d, *J* = 7.2 Hz, 3H). **$^{19}$F NMR** (376 MHz, $CDCl_3$) [signal of the *anti* isomer] δ = -70.16 (d, *J* = 8.7 Hz, 3F). **$^{13}$C NMR** (101 MHz, $CDCl_3$) [signals of the *anti* isomer] δ = 169.8, 166.5, 162.7, 131.0, 129.0, 126.8 (d, $^1J_{C\text{-}F}$ = 280.2 Hz), 125.9, 119.7, 113.9, 66.8, 55.5, 51.7 (q, $^3J_{C\text{-}F}$ = 2.2 Hz), 40.7 (q, $^2J_{C\text{-}F}$ = 26.6 Hz), 9.8 (q, $^3J_{C\text{-}F}$ = 2.7 Hz). **HPLC**: Chiralpak AD-H (*n*-hexane/iPrOH 80:20, flow-rate 0.75 mL/min; $t_{major}$ = 10.6 min; $t_{minor}$ = 16.2 min).

**Allyl (2*S*,3*R*)-4,4,4-trifluoro-2-(4-methoxybenzamido)-3-methylbutanoate *syn*-4p**.

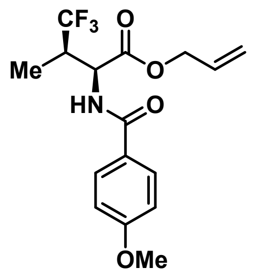

Following general procedure C from azlactone **1p**, cat. **2p**, allyl alcohol, and cat. **dhQN-10**, product *syn*-**4p** was obtained as a white waxy solid in 85% yield (d.r. of the crude = 1.3:1) and 99% ee after column chromatography on silica gel ($CH_2Cl_2/Et_2O$ = 100:1). In order to reach full conversion of the intermediate **3p**, additional 10 mol% of cat **dhQN-10** and 2 equivalents of allyl alcohol were added after 3 days (6 days of reaction time). **$^1$H NMR** (400 MHz, $CDCl_3$) [signals of the *syn* isomer] δ = 7.93 – 7.69 (m, 2H), 7.07 – 6.70 (m, 2H), 6.56 (d, $J$ = 9.1 Hz, 1H), 5.98 – 5.86 (m, 1H), 5.36 (dq, $J$ = 17.2, 1.4 Hz, 1H), 5.29 (dq, $J$ = 10.4, 1.2 Hz, 1H), 5.19 – 5.15 (m, 1H), 4.70 – 4.67 (m, 2H), 3.86 (s, 3H), 3.20 – 3.07 (m, 1H), 1.28 (d, $J$ = 5.8 Hz, 3H). **$^{19}$F NMR** (376 MHz, $CDCl_3$) [signal of the *syn* isomer] δ = -68.33 (d, $J$ = 9.1 Hz, 3F). **$^{13}$C NMR** (101 MHz, $CDCl_3$) [signals of the *syn* isomer] δ = 170.0, 167.2, 162.9, 131.3, 129.1, 127.3 (q, $^1J_{C\text{-}F}$ = 280.8 Hz), 125.8, 119.7, 114.1, 66.9, 55.6, 52.2 (q, $^3J_{C\text{-}F}$ = 2.0 Hz), 40.9 (q, $^2J_{C\text{-}F}$ = 25.5 Hz), 11.2 (q, $^3J_{C\text{-}F}$ = 2.7 Hz). **HPLC** [peaks of the *syn* isomer]: Chiralpak AD-H (*n*-hexane/iPrOH 80:20, flow-rate 0.75 mL/min; $t_{major}$ = 14.8 min; $t_{minor}$ = 11.4 min).

**HRMS** [*syn*-**4p** and *anti*-**4p** isomers]: calculated for $C_{16}H_{19}F_3NO_4$ [M + H$^+$] = 346.1261; found: 346.1252.

**Methyl (2*R*,3*R*)-4,4,4-trifluoro-2-(4-methoxybenzamido)-3-phenylbutanoate *anti*-4q**.

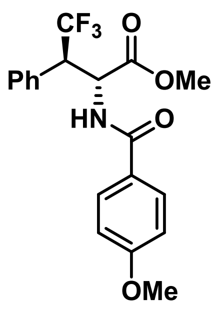

Following general procedure A from azlactone **1d**, cat. **2p**, methanol, and cat. **dhQD-2**, product *anti*-**4q** was obtained as a white solid in 91% yield (d.r. of the crude > 20:1) and 97% ee after column chromatography on silica gel ($CH_2Cl_2/Et_2O$ = 120:1). **$[\alpha]_D^{25}$** = -29 (c = 0.27 in $CHCl_3$). **$^1$H NMR** (400 MHz, $CDCl_3$) δ = 7.65 – 7.56 (m, 2H), 7.46 – 7.30 (m, 5H), 6.93 – 6.82 (m, 2H), 6.32 (d, $J$ = 9.3 Hz, 1H), 5.64 (dd, $J$ = 9.3, 4.8 Hz, 1H), 4.14 (qd, $J$ = 9.4, 4.8 Hz, 1H), 3.82 (s, 3H), 3.76 (s, 3H). **$^{19}$F NMR** (376 MHz, $CDCl_3$) δ = -66.39 (d, $J$ = 9.2 Hz). **$^{13}$C NMR** (101 MHz, $CDCl_3$) δ = 170.6, 166.4, 162.7, 130.6, 129.4, 129.28, 129.26, 129.0, 125.8, 125.7 (q, $^1J_{C\text{-}F}$ = 280.7 Hz), 114.0, 55.5, 53.1, 52.22 – 50.92 (m, 2C). **HPLC**: Chiralpak AD-H (*n*-hexane/iPrOH 80:20, flow-rate 0.75 mL/min; $t_{major}$ = 10.9 min; $t_{minor}$ = 17.9 min).

**Methyl (2*S*,3*R*)-4,4,4-trifluoro-2-(4-methoxybenzamido)-3-phenylbutanoate *syn*-4q**.

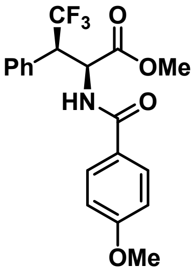

Following general procedure A from azlactone **1d**, cat. **2p**, methanol, and cat. **dhQN-10**, product *syn*-**4q** was obtained as a white solid in 77% yield (d.r. of the crude = 5.3:1) and >99% ee after column chromatography on silica gel ($CH_2Cl_2/Et_2O$ = 100:1). **$^1$H NMR** (400 MHz, $CD_2Cl_2$) [signals of the *syn* isomer] δ = 7.77 – 7.68 (m, 2H), 7.39 – 7.30 (m, 5H), 6.97 – 6.91 (m, 2H), 6.67 (d, *J* = 9.1 Hz, 1H), 5.40 (dd, *J* = 9.1, 6.9 Hz, 1H), 4.07 (qd, *J* = 9.6, 6.9 Hz, 1H), 3.83 (s, 3H), 3.60 (s, 3H). **$^{19}$F NMR** (376 MHz, $CD_2Cl_2$) [signals of the *syn* isomer] δ = -62.64 (d, *J* = 9.4 Hz). **$^{13}$C NMR** (101 MHz, $CD_2Cl_2$) [signals of the *syn* isomer] δ = 172.4, 168.6, 165.1, 133.8, 133.7, 131.7, 131.34, 131.32, 131.2, 128.4 (q, $^1J_{C\text{-}F}$ = 281.1 Hz), 116.2, 57.8, 55.3, 55.0, 53.95 (q, $^2J_{C\text{-}F}$ = 26.2 Hz). **HPLC** [peaks of the *syn* isomer]: Chiralpak AD-H (*n*-hexane/iPrOH 80:20, flow-rate 0.75 mL/min; $t_{major}$ = 22.2 min; $t_{minor}$ = 14.0 min).

**HRMS** [*syn*-**4q** and *anti*-**4q** isomers]: calculated for $C_{19}H_{19}F_3NO_4$ [M + H$^+$] = 382.1266; found: 382.1264.

**Benzyl (2*R*,3*R*)-4,4,4-trifluoro-2-(4-methoxybenzamido)-3-phenylbutanoate *anti*-4r**.

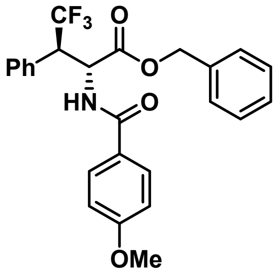

Following general procedure A from azlactone **1d**, cat. **2b**, benzyl alcohol, and cat. **dhQD-2**, product *anti*-**4r** was obtained as a white solid in 81% yield (d.r. of the crude 18:1) and 98% ee after column chromatography on silica gel ($CH_2Cl_2/Et_2O$ = 100:1). **$^1$H NMR** (400 MHz, $CDCl_3$) [signals of the *anti* isomer] δ = 7.74 – 7.57 (m, 2H), 7.45 – 7.29 (m, 8H), 7.22 – 7.18 (m, 2H), 6.97 – 6.84 (m, 2H), 6.32 (d, *J* = 9.3 Hz, 1H), 5.70 (dd, *J* = 9.3, 4.1 Hz, 1H), 5.25 (d, *J* = 12.0 Hz, 1H), 5.13 (d, *J* = 12.0 Hz, 1H), 4.10 (qd, *J* = 9.4, 4.1 Hz, 1H), 3.83 (s, 3H). **$^{19}$F NMR** (376 MHz, $CDCl_3$) [signals of the *anti* isomer] δ = -66.24 (d, *J* = 9.4 Hz). **$^{13}$C NMR** (101 MHz, $CDCl_3$) [signals of the *anti* isomer] δ = 169.9, 166.4, 162.7, 134.8, 130.3, 129.3, 129.2, 129.02, 129.00, 128.9, 128.8, 125.8, 125.7 (q, $^1J_{C\text{-}F}$ = 280.8 Hz), 114.0, 68.2, 55.5, 51.93 – 50.85 (m, 2C). **HPLC** [peaks of the *anti* isomer]: Chiralpak AD-H (*n*-hexane/iPrOH 80:20, flow-rate 0.75 mL/min; $t_{major}$ = 12.9 min; $t_{minor}$ = 22.6 min).

**Benzyl (2*S*,3*R*)-4,4,4-trifluoro-2-(4-methoxybenzamido)-3-phenylbutanoate *syn*-4r**.

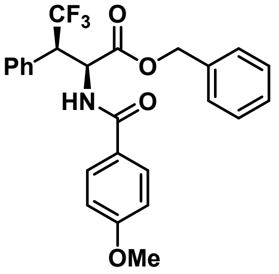

Following general procedure A from azlactone **1d**, cat. **2p**, benzyl alcohol, and cat. **dhQN-10**, product *syn*-**4r** was obtained as a white solid in 70% yield (d.r. of the crude = 5.9:1) and 99% ee after column chromatography on silica gel ($CH_2Cl_2/Et_2O$ = 100:1). In order to reach full conversion of the intermediate **3r**, further 5 mol% of cat **dhQN-10** and 1 equivalent of benzyl alcohol were added after 2 days (7 days of reaction time). **$^{1}$H NMR** (400 MHz, $CDCl_3$) [signals of the *syn* isomer] δ = 7.76 – 7.71 (m, 2H), 7.36 – 7.26 (m, 8H), 7.21 – 7.13 (m, 2H), 6.96 – 6.90 (m, 2H), 6.70 (d, *J* = 9.0 Hz, 1H), 5.51 (dd, *J* = 9.0, 7.1 Hz, 1H), 5.07 (d, *J* = 12.1 Hz, 1H), 5.01 (d, *J* = 12.1 Hz, 1H), 4.05 (qd, *J* = 9.4, 7.2 Hz, 1H), 3.85 (s, 3H). **$^{19}$F NMR** (376 MHz, $CDCl_3$) [signals of the *syn* isomer] δ = -64.06 (d, *J* = 9.4 Hz, 3F).**$^{13}$C NMR** (101 MHz, $CDCl_3$) [signals of the *syn* isomer] δ = 169.9, 166.6, 162.8, 134.6, 131.2, 129.5, 129.14, 129.12, 129.0, 128.7, 128.64, 128.61, 126.0 (q, $^{1}J_{C\text{-}F}$ = 281.1 Hz), 125.8, 114.0, 68.0, 55.5, 53.1, 51.8 (q, $^{2}J_{C\text{-}F}$ = 26.5 Hz). **HPLC** [peaks of the *syn* isomer]: Chiralpak AD-H (*n*-hexane/iPrOH 80:20, flow-rate 0.75 mL/min; $t_{major}$ = 21.1 min; $t_{minor}$ = 17.0 min).

**HRMS** [*syn*-**4r** and *anti*-**4r** isomers]: calculated for $C_{25}H_{22}F_3NNaO_4$ [M + H$^+$] = 480.1399; found: 480.1389.

**Isobutyl (2*R*,3*R*)-4,4,4-trifluoro-2-(4-methoxybenzamido)-3-phenylbutanoate *anti*-4s**.

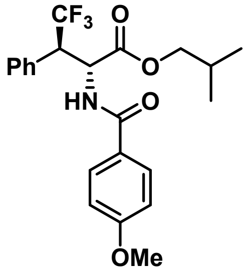

Following general procedure A from azlactone **1d**, cat. **2p**, isobutyl alcohol, and cat. **dhQD-2**, product *anti*-**4s** was obtained as a white solid in 50% yield (d.r. of the crude > 20:1) and 96% ee after column chromatography on silica gel ($CH_2Cl_2/Et_2O$ = 100:1). In order to reach full conversion of intermediate **3s**, further 5 mol% of cat **dhQD-2** and 1 equivalent of isobutyl alcohol were added after 2 days (3 days of reaction time). **$[\alpha]_D^{25}$** = -42 (c = 0.27 in $CHCl_3$). **$^{1}$H NMR** (400 MHz, $CDCl_3$) δ = 7.67 – 7.61 (m, 2H), 7.44 – 7.33 (m, 5H), 6.93 – 6.86 (m, 2H), 6.28 (d, *J* = 9.2 Hz, 1H), 5.64 (dd, *J* = 9.3, 4.6 Hz, 1H), 4.11 (qd, *J* = 9.4, 4.6 Hz, 1H), 3.95 (dd, *J* = 10.6, 6.6 Hz, 1H), 3.88 (dd, *J* = 10.6, 6.7 Hz, 1H), 3.83 (s, 3H), 1.95 (hept, *J* = 6.7 Hz, 1H), 0.93 (d, *J* = 6.7 Hz, 3H), 0.91 (d, *J* = 6.7 Hz, 3H). **$^{19}$F NMR** (376 MHz, $CDCl_3$) δ = -66.30 (d, *J* = 9.4 Hz, 3F). **$^{13}$C NMR** (101 MHz, $CDCl_3$) δ = 170.2, 166.4, 162.7, 130.6, 129.4, 129.28, 129.26, 129.0, 125.9, 125.8 (q, $^{1}J_{C\text{-}F}$

= 280.6 Hz), 114.0, 72.5, 55.5, 52.6 – 50.3 (m, 2C), 27.7, 19.1. **HPLC**: Chiralcel OD-H (*n*-hexane/iPrOH 95:5, flow-rate 0.75 mL/min; $t_{major}$ = 14.3 min; $t_{minor}$ = 21.1 min).

**Isobutyl (2*S*,3*R*)-4,4,4-trifluoro-2-(4-methoxybenzamido)-3-phenylbutanoate** *syn*-**4s**.

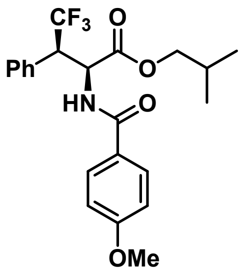


Following general procedure B from azlactone **1d**, cat. **2p**, isobutyl alcohol, and cat. **dhQN-10**, product *syn*-**4s** was obtained as a white solid in 50% yield (d.r. of the crude = 6.7:1) and 99% ee after column chromatography on silica gel ($CH_2Cl_2/Et_2O$ = 100:1). In order to reach full conversion of intermediate **3s**, further 5 mol% of cat **dhQN-10** and 1 equivalent of isobutyl alcohol were added after 3 days (6 days of reaction time). **$^{1}$H NMR** (400 MHz, $CDCl_3$) [signals of the *syn* isomer] δ = 7.78 – 7.68 (m, 2H), 7.35 (s, 5H), 7.01 – 6.89 (m, 2H), 6.64 (d, *J* = 9.0 Hz, 1H), 5.45 (dd, *J* = 9.1, 7.0 Hz, 1H), 4.11 – 4.00 (m, 1H), 3.88 – 3.82 (m, 4H), 3.74 (dd, *J* = 10.5, 6.6 Hz, 1H), 1.80 (hept, *J* = 6.7 Hz, 1H), 0.84 (d, *J* = 6.7 Hz, 3H), 0.82 (d, *J* = 6.7 Hz, 3H). **$^{19}$F NMR** (376 MHz, $CDCl_3$) [signals of the *syn* isomer] δ = -64.08 (d, *J* = 9.7 Hz, 3F). **$^{13}$C NMR** (101 MHz, $CDCl_3$) [signals of the *syn* isomer] δ = 170.2, 166.5, 162.8, 131.4, 131.4, 129.5, 129.1, 129.0, 126.0 (q, $^{1}J_{C\text{-}F}$ = 281.3 Hz), 125.9, 114.0, 72.3, 55.6, 53.1, 51.9 (q, $^{2}J_{C\text{-}F}$ = 26.5 Hz), 27.5, 19.1, 19.0. **HPLC** [peaks of the *syn* isomer]: Chiralcel OD-H (*n*-hexane/iPrOH 95:5, flow-rate 0.75 mL/min; $t_{major}$ = 33.7 min; $t_{minor}$ = 19.3 min). **HRMS** [*syn*-**4s** and *anti*-**4s** isomers]: calculated for $C_{22}H_{25}F_3NO_4$ [M + H$^+$] = 424.1736; found: 424.1729.

## Reduction of the ester moiety of products *anti*-4j and *syn*-4j.

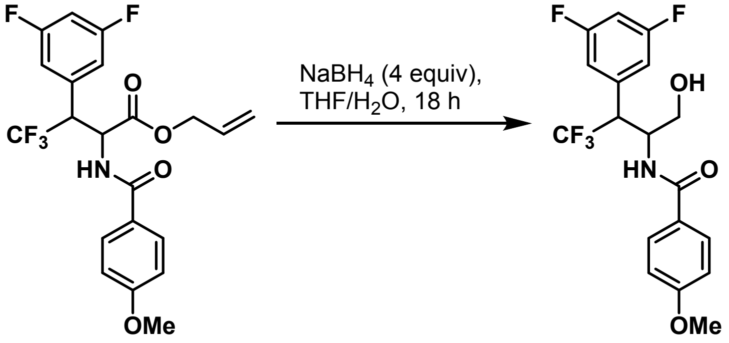


**4j** **5**

To a stirred and cooled (0 °C) solution of compound *anti*-**4j** or *syn*-**4j** (66.5 mg, 0.15 mmol) in a THF/$H_2O$ mixture (12 : 1, 1.5 mL), $NaBH_4$ was added (23 mg, 0.6 mmol). The resulting mixture was allowed to warm to RT, and stirred overnight, then diluted with EtOAc and quenched with a saturated aqueous $NH_4Cl$ solution. The phases were separated, and the aqueous phase extracted twice with EtOAc. The combined organic phases were dried with $MgSO_4$, filtered and evaporated under reduced pressure. Chromatographic purification furnished compounds *anti*-**5** or *syn*-**5**.

***N*-((2*R*,3*R*)-3-(3,5-difluorophenyl)-4,4,4-trifluoro-1-hydroxybutan-2-yl)-4-methoxybenzamide *anti*-5.**

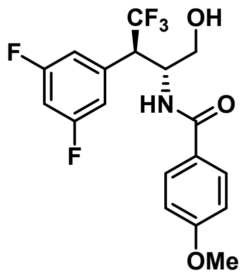

Following the above procedure from *anti*-**4j** (d.r. > 20:1), product *anti*-**5** was obtained as a white solid in 60% yield (d.r. > 20:1) and >99% ee after column chromatography on silica gel ($CH_2Cl_2/Et_2O$ 5:1). **$[\alpha]_D^{25}$** = +10 (c = 0.27 in $CHCl_3$). **$^1$H NMR** (400 MHz, $CD_3OD$) δ = 7.62 – 7.51 (m, 2H), 7.10 – 6.99 (m, 2H), 6.97 – 6.88 (m, 3H), 4.90-4.83 (m, 1H, this signal is overlapping with the signal of water in $CD_3OD$), 4.06 (qd, $J$ = 9.6, 7.5 Hz, 1H), 3.81 (s, 3H), 3.74 (dd, $J$ = 11.5, 5.6 Hz, 1H), 3.70 (dd, $J$ = 11.4, 5.4 Hz, 1H). **$^{19}$F NMR** (376 MHz, $CD_3OD$) δ = -62.73 (d, $J$ = 9.5 Hz, 3F), 107.36 – 107.45 (m, 2F). **$^{13}$C NMR** (100 MHz, $CD_3OD$) δ = 169.5, 164.3 (dd, $^1J_{C\text{-}F}$ = 247.4, $^3J_{C\text{-}F}$ = 12.9 Hz), 164.0, 138.2 – 137.8 (m), 130.1, 127.5 (q, $^1J_{C\text{-}F}$ = 279.6 Hz), 127.4, 114.6, 114.4 – 113.5 (m), 104.7 (t, $^2J_{C\text{-}F}$ = 25.6 Hz), 62.7, 55.9, 51.4, 50.6 (q, $^2J_{C\text{-}F}$ = 26.4 Hz). **HPLC**: Chiralpak AD-H (*n*-hexane/iPrOH 95:5, flow-rate 0.75 mL/min; $t_{major}$ = 23.1 min; $t_{minor}$ = 26.9 min). **HRMS**: calculated for $C_{18}H_{16}F_5NNaO_3$ [M + $Na^+$] = 412.0943; found: 412.0939.

***N*-((2*S*,3*R*)-3-(3,5-difluorophenyl)-4,4,4-trifluoro-1-hydroxybutan-2-yl)-4-methoxybenzamide** *syn*-**5**.

Following the above procedure from *syn*-**4j** (d.r. = 4.0:1) product *syn*-**5** was obtained as a white solid in 50% yield (d.r. > 20:1) and >99% ee after column chromatography on silica gel ($CH_2Cl_2/Et_2O$ 5:1). **$[\alpha]_D^{25}$** = -12 (c = 0.27 in $CHCl_3$). **$^1$H NMR** (400 MHz, $CD_3OD$) δ = 7.88-7.82 (m, 2H), 7.12 – 7.05 (m, 2H), 7.05-7.02 (m, 1H), 7.02-6.97 (m, 2H), 4.80-4.73 (m, 1H), 4.03 (quint, *J* = 9.1 Hz, 1H), 3.85 (s, 3H), 3.51 (dd, *J* = 11.5, 2.8 Hz, 1H), 3.33 (dd, *J* = 11.1, 4.7 Hz, 1H). **$^{19}$F NMR** (376 MHz, $CD_3OD$) δ = -61.71 (d, *J* = 9.4 Hz, 3F), -106.77 (t, *J* = 8.2 Hz, 2F). **$^{13}$C NMR** (100 MHz, $CD_3OD$) δ = 169.9, 164.6 (dd, $^1J_{C-F}$ = 248.4, $^3J_{C-F}$ = 13.7 Hz), 164.0, 139.9 – 138.7 (m), 130.3, 127.70, 127.68 (q, $^1J_{C-F}$ = 281.6 Hz), 114.7, 113.8 – 113.5 (m), 104.9 (t, $^2J_{C-F}$ = 26.0 Hz), 62.7, 55.9, 52.5, 50.8 (q, $^2J_{C-F}$ = 23.6 Hz). **HPLC**: Chiralpak AD-H (*n*-hexane/iPrOH 95:5, flow-rate 0.75 mL/min; $t_{major}$ = 32.8 min; $t_{minor}$ = 41.4 min). **HRMS**: calculated for $C_{18}H_{16}F_5NNaO_3$ [M + Na$^+$] = 412.0943; found: 412.0945.

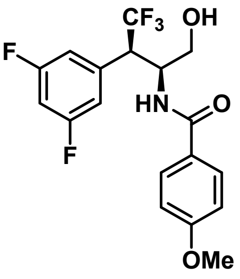

**Hydrolysis of the amide moiety of products *anti*-5 and *syn*-5,[25] and assignment of the relative and absolute configuration of the products.**

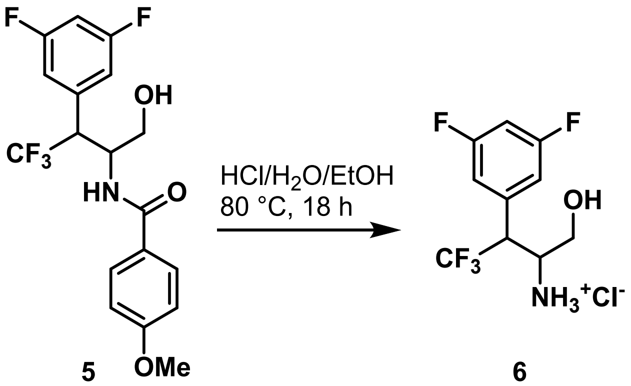


5      6

EtOH (1 mL), $H_2O$ (1 mL), and conc. aqueous HCl (2 mL) were sequentially added to a vial containing compound *anti*-**5** or *syn*-**5** (0.06 mmol). The resulting mixture was heated to 80 °C with stirring overnight. The solvents were then evaporated, the residue dissolved in $H_2O$ and washed three times with $Et_2O$. The aqueous phase was evaporated to dryness under reduced pressure, affording amino alcohol hydrochlorides *anti*-**6** or *syn*-**6**.

**(2*R*,3*R*)-3-(3,5-Difluorophenyl)-4,4,4-trifluoro-1-hydroxybutan-2-aminium chloride *anti*-6.**

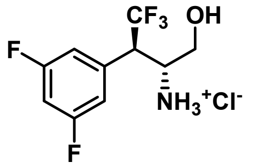

Following the above procedure from *anti*-**5** (d.r. > 20:1), product *anti*-**6** was obtained as a white solid in quantitative yield (d.r. > 20:1). **$[\alpha]_D^{25}$** = +32 (c = 0.58 in MeOH). **$^1$H NMR** (400 MHz, $CD_3OD$) δ = 7.12-6.97 (m, 3H), 4.03 (quint, *J* = 8.8 Hz, 1H), 3.96-3.83 (m, 2H), 3.78 (dd, *J* = 11.3, 4.5 Hz, 1H). **$^{19}$F NMR** (376 MHz, $CD_3OD$) δ = -66.26 (d, *J* = 8.8 Hz, 3F), -109.30 (t, *J* = 7.6 Hz, 2F). **$^{13}$C NMR** (100 MHz, $CD_3OD$) [benzylic $^{13}$C signal overlapped with $CD_3OD$ signal] δ = 165.0 (dd, $^1J_{C\text{-}F}$ = 249.2, $^3J_{C\text{-}F}$ = 12.7 Hz), 135.6-135.2 (m), 126.5 (q, $^1J_{C\text{-}F}$ = 280.4 Hz), 114.5-113.9 (m), 106.3 (t, $^2J_{C\text{-}F}$ = 24.2 Hz), 60.4 (br s), 53.7. **HRMS**: calculated for $C_{10}H_{11}F_5NO$ [$M^+$] = 256.0755; found: 256.0758.

[25] Procedure adapted from: N. P. Peet, J. P. Burkhart, M. R. Angelastro, E. L. Giroux, S. Mehdi, P. Bey, M. Kolb, B. Neises and D. Schirlin, *J. Med. Chem.*, 1990, **33**, 394.

**(2*S*,3*R*)-3-(3,5-Difluorophenyl)-4,4,4-trifluoro-1-hydroxybutan-2-aminium chloride *syn*-6.**

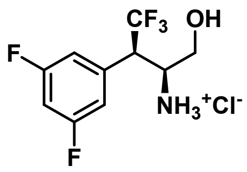

Following the above procedure from *syn*-**5** (d.r. > 20:1), product *syn*-**6** was obtained as a white solid in 93% yield (d.r. > 20:1). **$[\alpha]_D^{25}$** = +30 (c = 1.0 in MeOH). **$^1$H NMR** (400 MHz, $CD_3OD$) δ = 7.08-6.94 (m, 3H), 4.00 (quint, *J* = 8.5 Hz, 1H), 3.97-3.88 (m, 1H), 3.56 (br d, *J* = 11.1 Hz, 1H), 3.17 (dd, *J* = 11.9, 3.4 Hz, 1H). **$^{19}$F NMR** (376 MHz, $CD_3OD$) δ = -66.57 (d, *J* = 8.9 Hz, 3F), -109.87 (t, *J* = 8.0 Hz, 2F). **$^{13}$C NMR** (100 MHz, $CD_3OD$) δ = 164.6 (dd, $^1J_{C\text{-}F}$ = 249.0, $^3J_{C\text{-}F}$ = 13.1 Hz), 136.2-135.9 (m), 127.2 (q, $^1J_{C\text{-}F}$ = 281.1 Hz), 114.3-113.6 (m), 105.9 (t, $^2J_{C\text{-}F}$ = 25.3 Hz), 60.4 (br s), 52.6, 49.8 (q, $^2J_{C\text{-}F}$ = 26.1 Hz, partially overlapped with $CD_3OD$ signal). **HRMS**: calculated for $C_{10}H_{11}F_5NO$ [$M^+$] = 256.0755; found: 256.0757.

The relative configuration of amino alcohol hydrochloride *syn*-**6** was determined as *syn* by comparing its $^1$H NMR spectrum with the reported one.[23] Its absolute configuration was assigned as 2*S*,3*R* by comparing its **$[\alpha]_D^{25}$** = +30 (c = 1.0 in MeOH) with the reported value (**$[\alpha]_D^{25}$** = +40.4 (c = 1.0 in MeOH)).[23]

The correctness of this assignment was reinforced by the different $^1$H NMR spectrum displayed by its diastereomeric counterpart *anti*-**6**. The relative configuration of compound *anti*-**6** could be thus assigned as *anti*, while its absolute configuration as 2*R*,3*R*, considering that the same catalyst **2p** which is controlling the C3 chirality center was used. Furthermore, such assignment is fully in line with the notion that (dihydro)quinidine derived squaramide catalysts ((**dh**)**QD**) favor the formation of (2*R*)-amino acid derivatives, and (dihydro)quinine derived squaramide catalysts ((**dh**)**QN**) favor the formation of (2*S*)-amino acid derivatives, when applied to the alcoholytic DKR of azlactones.[1d-f] Indeed, based on the above assignment, in our case **dhQD-2** catalyst furnished the (2*R*)-product *anti*-**4j** (→ *anti*-**6**), while **dhQN-10** catalyst its (2*S*)-counterpart *syn*-**4j** (→ *syn*-**6**).

The configuration of all remaining compounds **4** was assigned by analogy.

Copies of $^{1}H$ and $^{13}C$ NMR spectra of products 4, 5, 6 (in $CDCl_3$ unless otherwise stated)

**Allyl (2*R*,3*R*)-4,4,4-trifluoro-2-(4-methoxybenzamido)-3-phenylbutanoate *anti*-4d.**

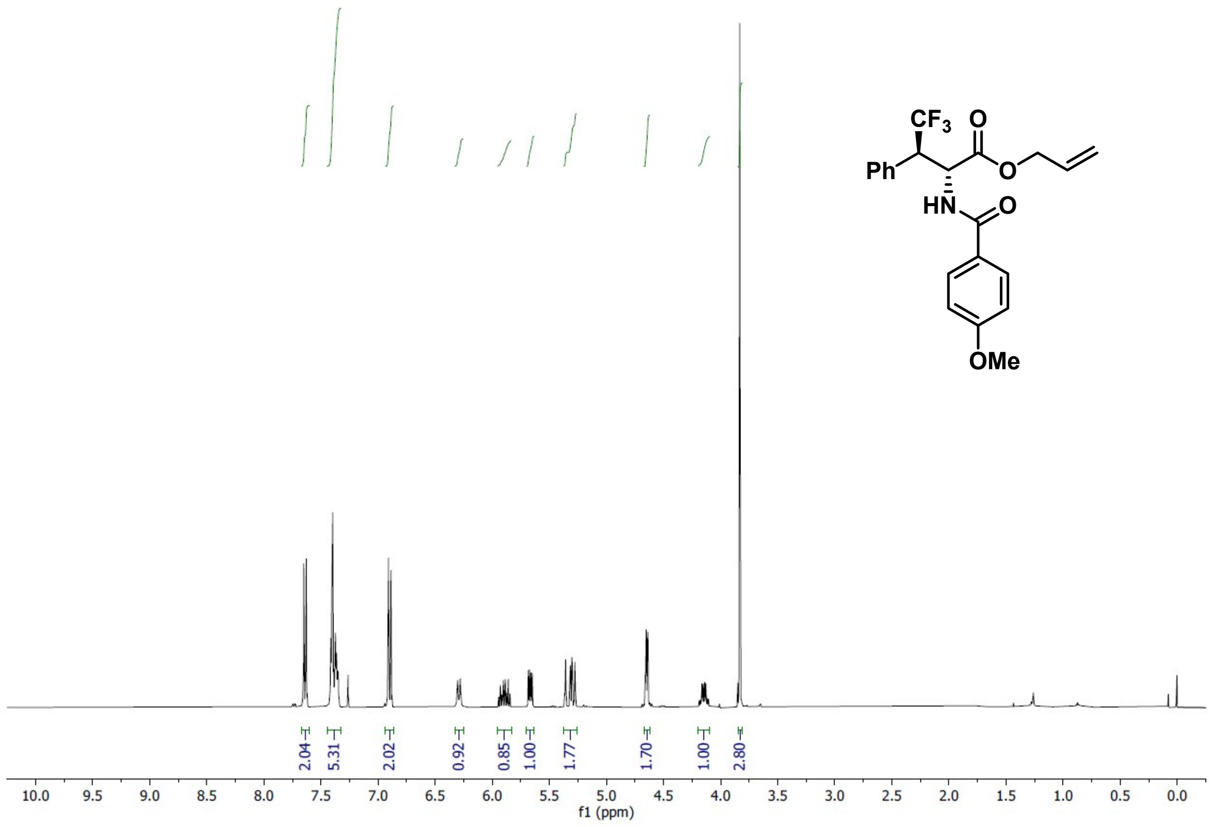

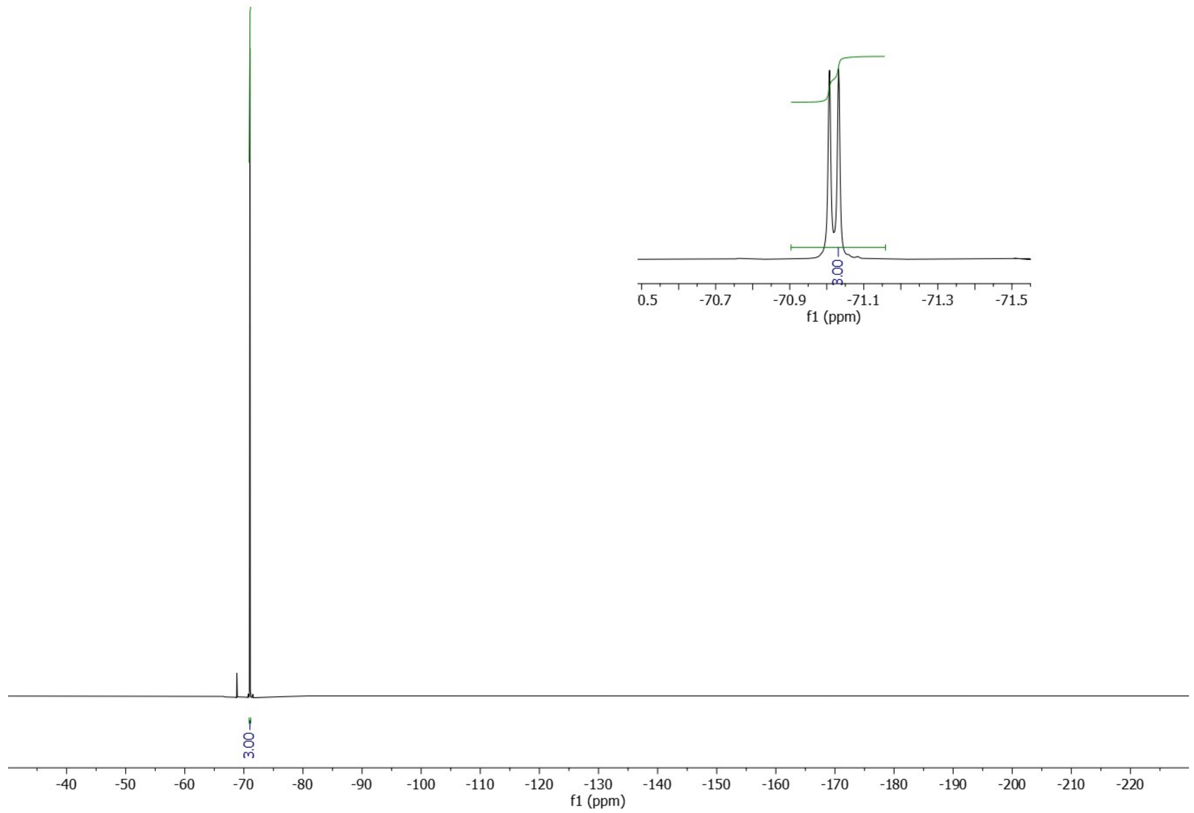

3.00
-40 -50 -60 -70 -80 -90 -100 -110 -120 -130 -140 -150 -160 -170 -180 -190 -200 -210 -220
f1 (ppm)
3.00
0.5 -70.7 -70.9 -71.1 -71.3 -71.5
f1 (ppm)


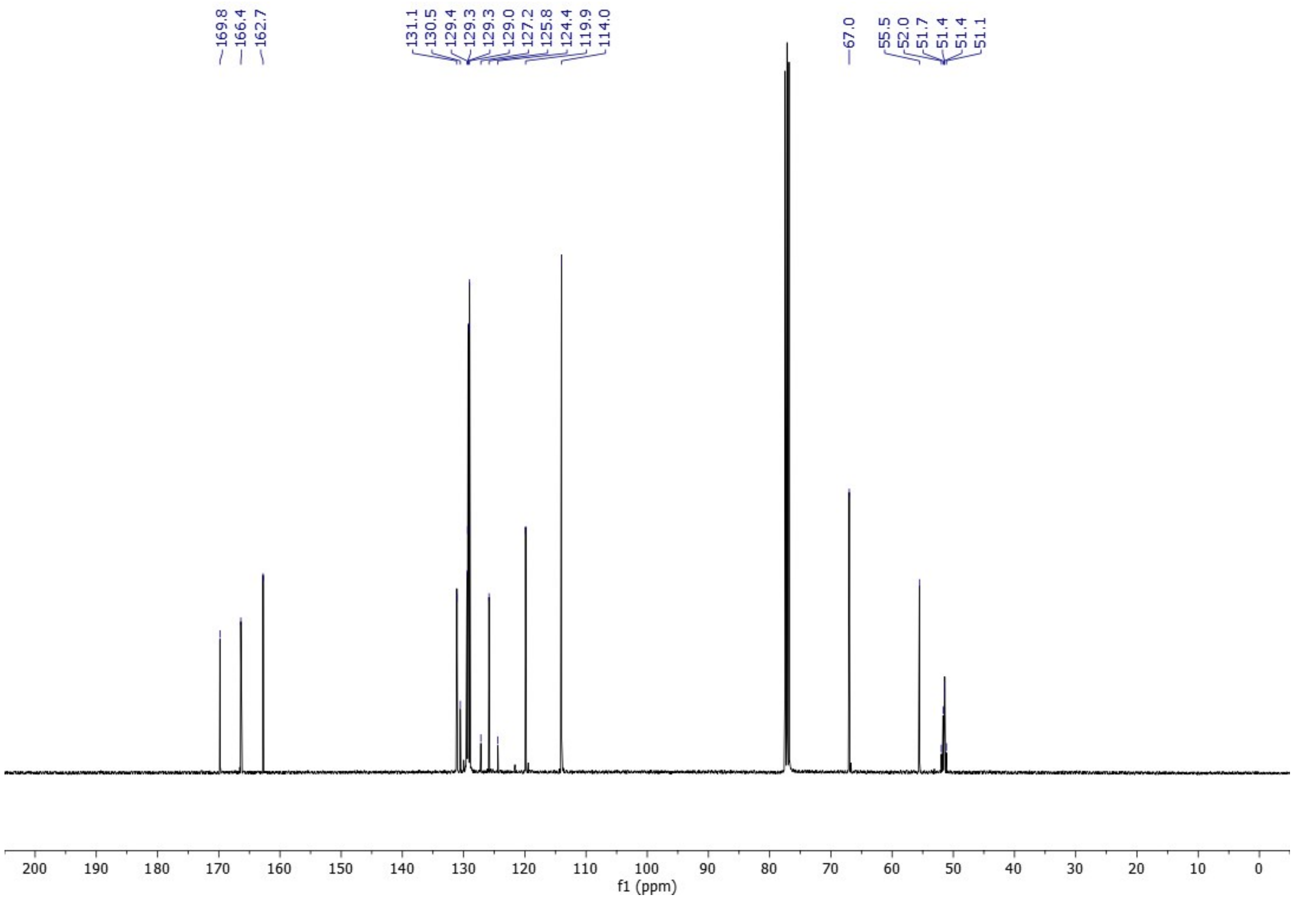

169.8
166.4
162.7
131.1
130.5
129.4
129.3
129.3
129.0
127.2
125.8
124.4
119.9
114.0
67.0
55.5
52.0
51.7
51.4
51.4
51.1
200 190 180 170 160 150 140 130 120 110 100 90 80 70 60 50 40 30 20 10 0
f1 (ppm)

**Allyl (2*S*,3*R*)-4,4,4-trifluoro-2-(4-methoxybenzamido)-3-phenylbutanoate *syn*-4d.**

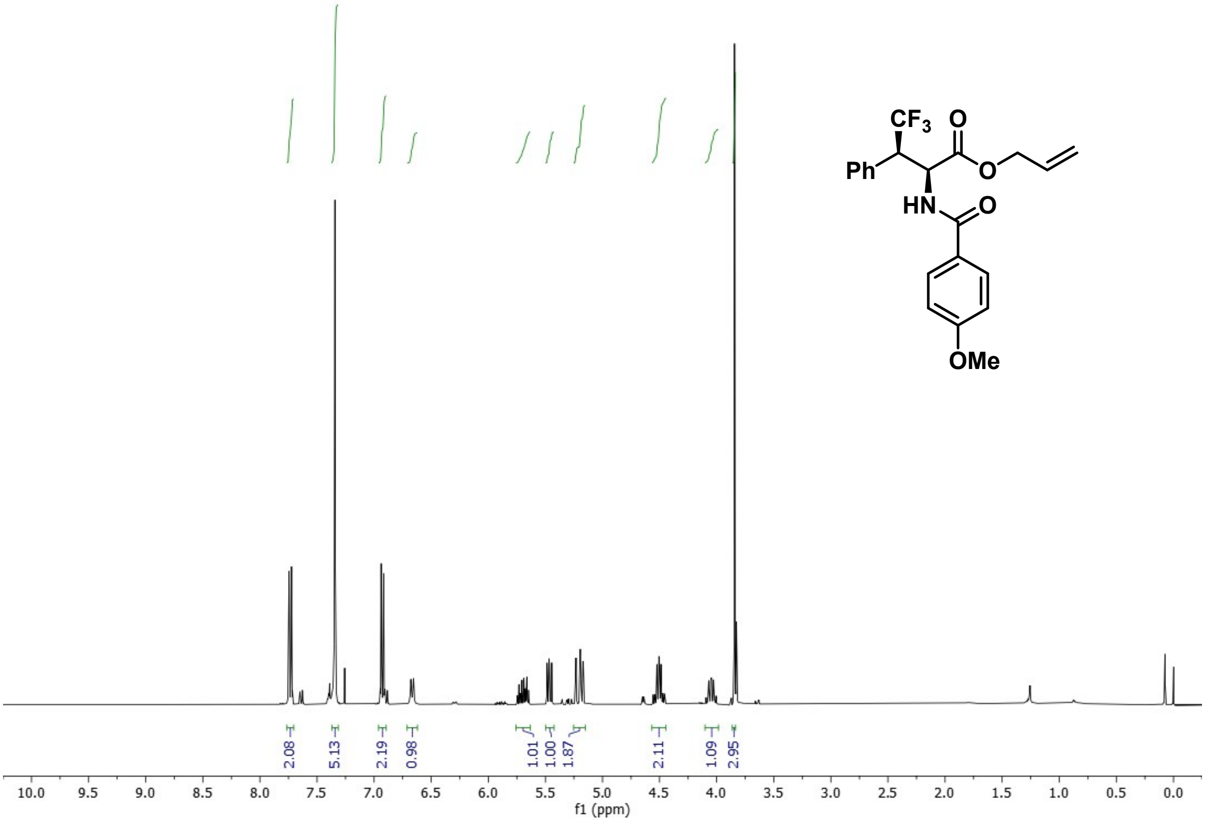

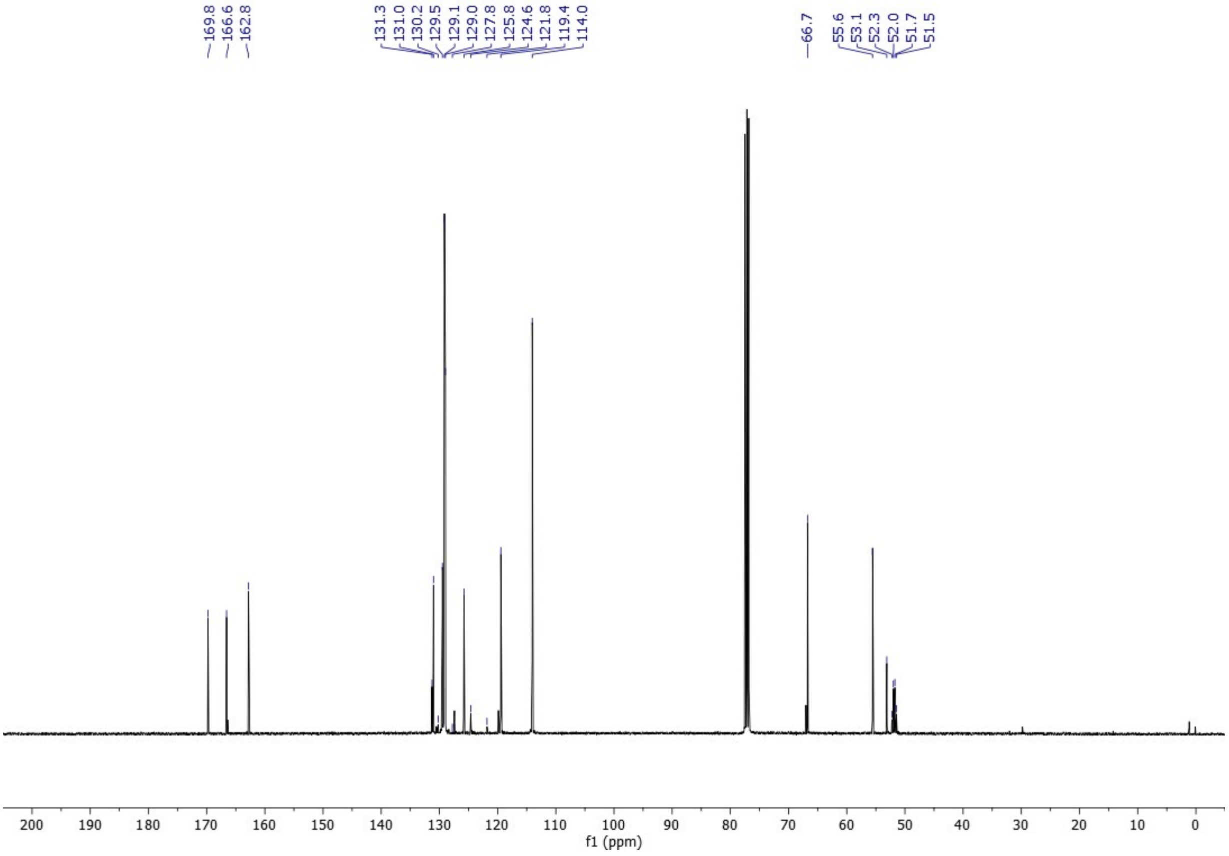
169.8
166.6
162.8
131.3
131.0
130.2
129.5
129.1
129.0
127.8
125.8
124.6
121.8
119.4
114.0
66.7
55.6
53.1
52.3
52.0
51.7
51.5
200 190 180 170 160 150 140 130 120 110 100 90 80 70 60 50 40 30 20 10 0
f1 (ppm)

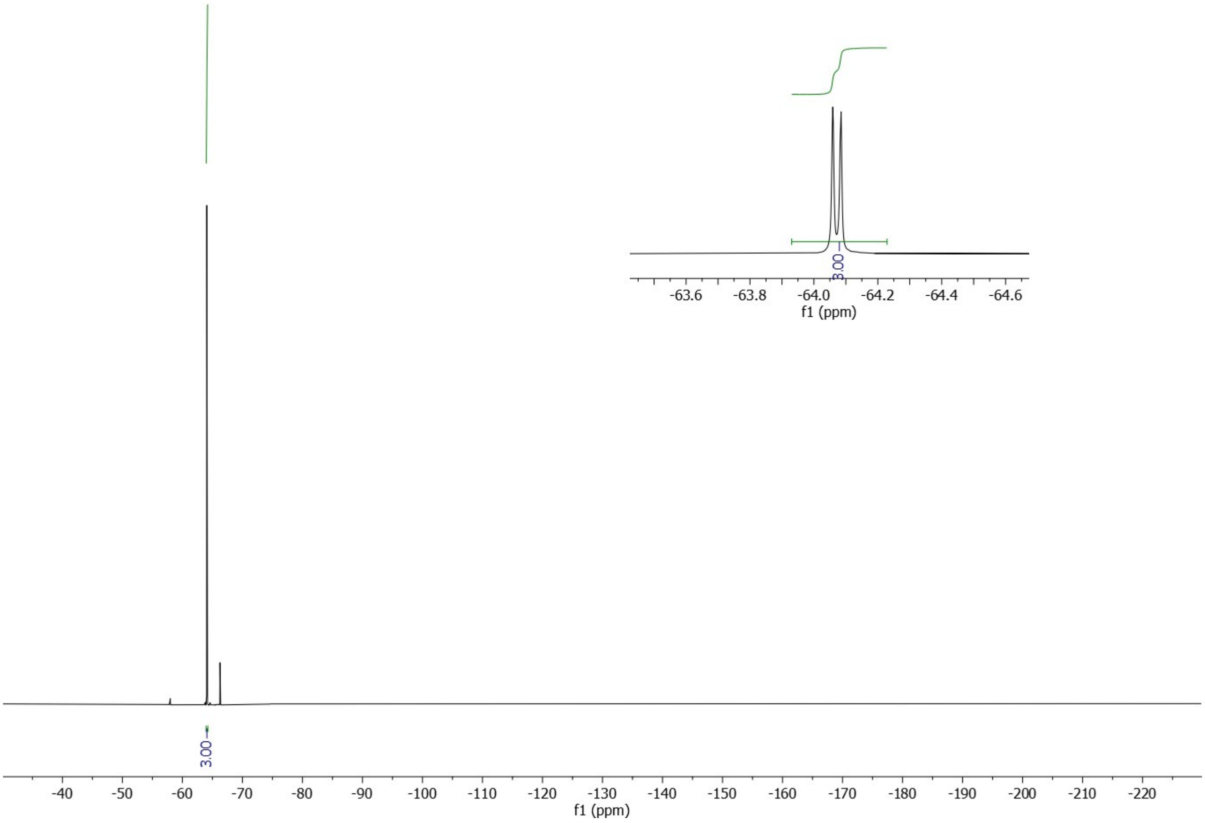
3.00
-63.6 -63.8 -64.0 -64.2 -64.4 -64.6
f1 (ppm)
3.00
-40 -50 -60 -70 -80 -90 -100 -110 -120 -130 -140 -150 -160 -170 -180 -190 -200 -210 -220
f1 (ppm)

**Allyl (2*R*,3*R*)-3-(4-bromophenyl)-4,4,4-trifluoro-2-(4-methoxybenzamido)butanoate** *anti*-**4e**.

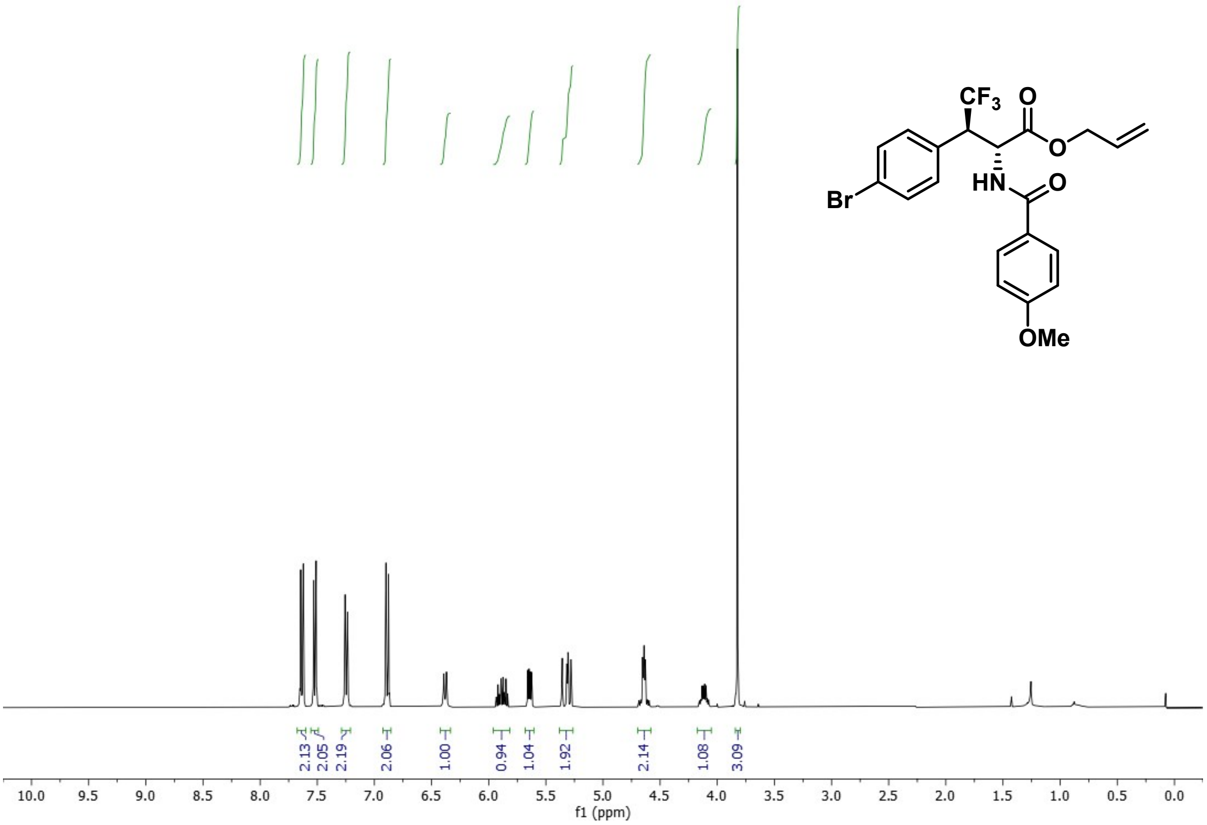

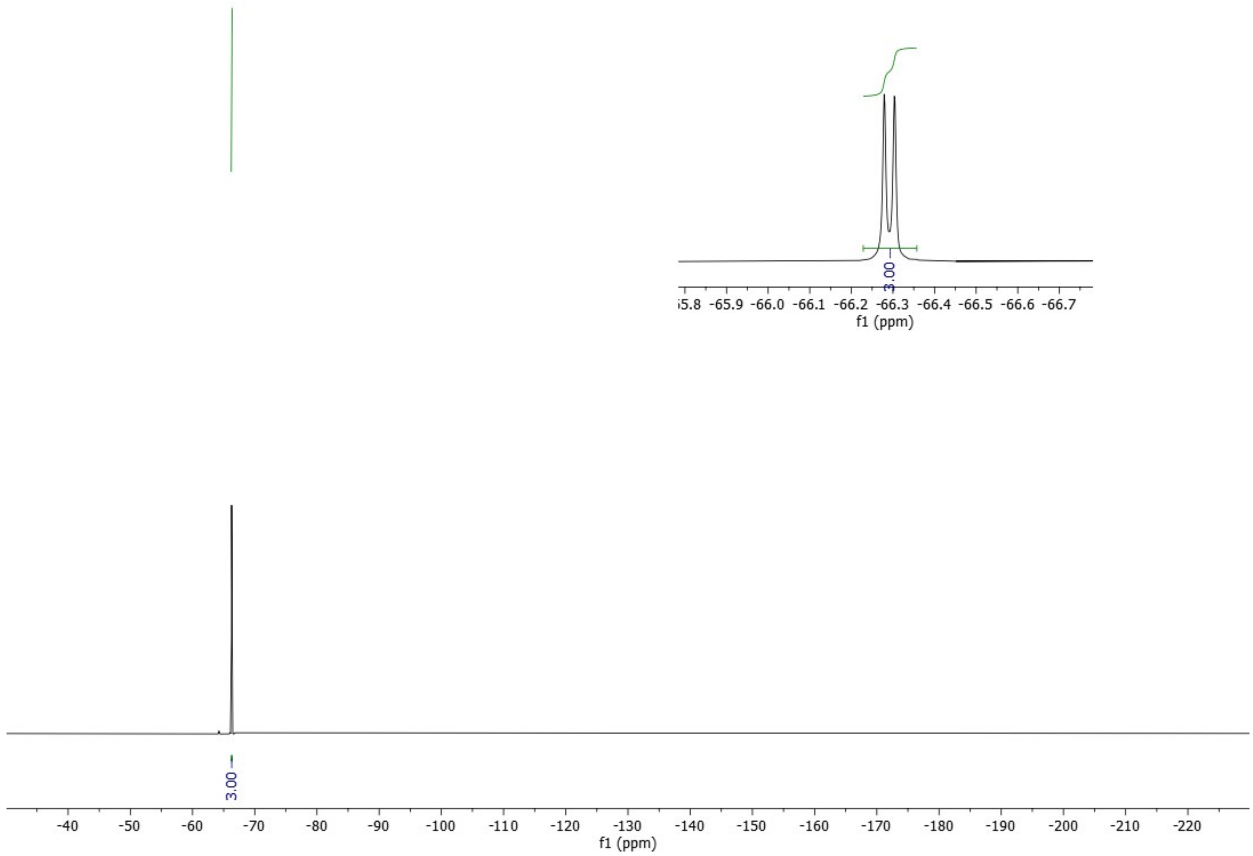
3.00
5.8 -65.9 -66.0 -66.1 -66.2 -66.3 -66.4 -66.5 -66.6 -66.7
f1 (ppm)
3.00
-40 -50 -60 -70 -80 -90 -100 -110 -120 -130 -140 -150 -160 -170 -180 -190 -200 -210 -220
f1 (ppm)

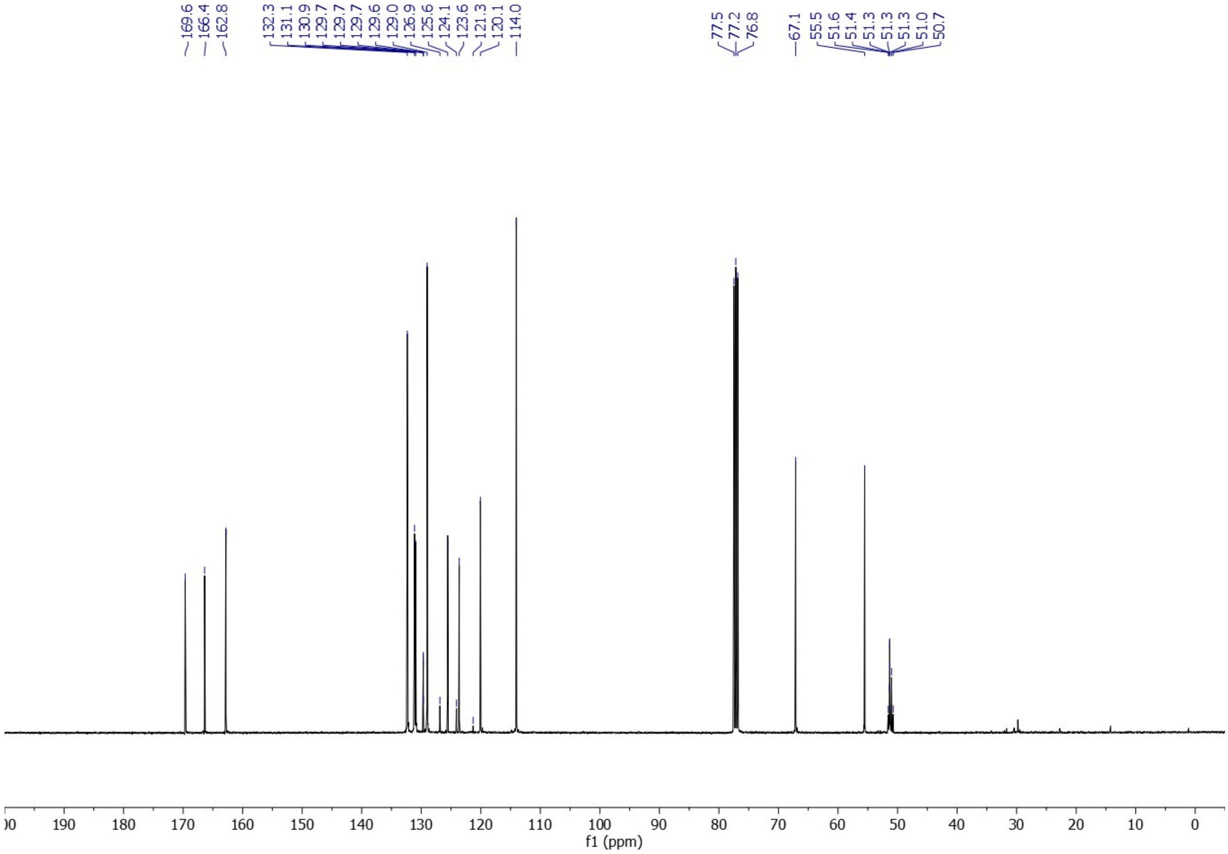
169.6
166.4
162.8
132.3
131.1
130.9
129.7
129.7
129.7
129.6
129.0
126.9
125.6
124.1
123.6
121.3
120.1
114.0
77.5
77.2
76.8
67.1
55.5
51.6
51.4
51.3
51.3
51.0
50.7
00 190 180 170 160 150 140 130 120 110 100 90 80 70 60 50 40 30 20 10 0
f1 (ppm)

**Allyl (2*S*,3*R*)-3-(4-bromophenyl)-4,4,4-trifluoro-2-(4-methoxybenzamido)butanoate** *syn*-**4e.**

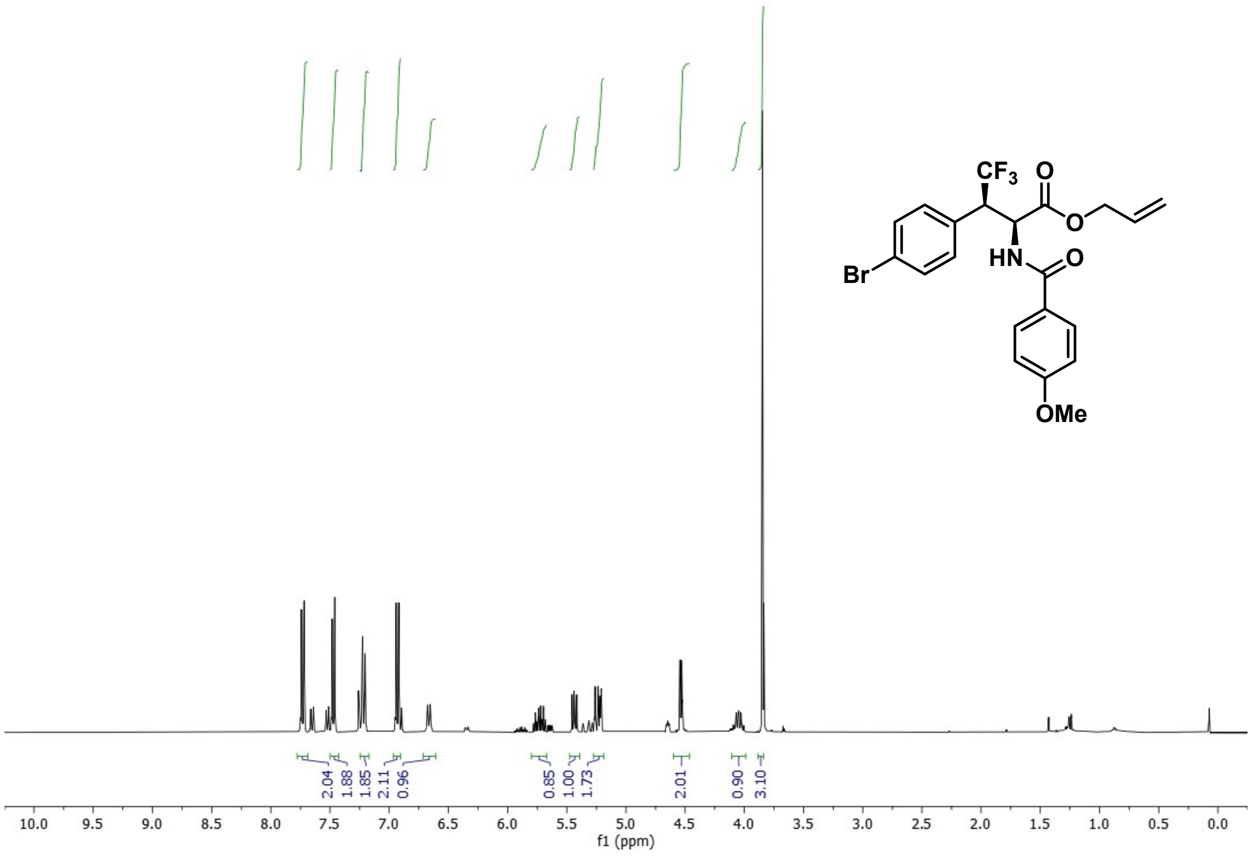


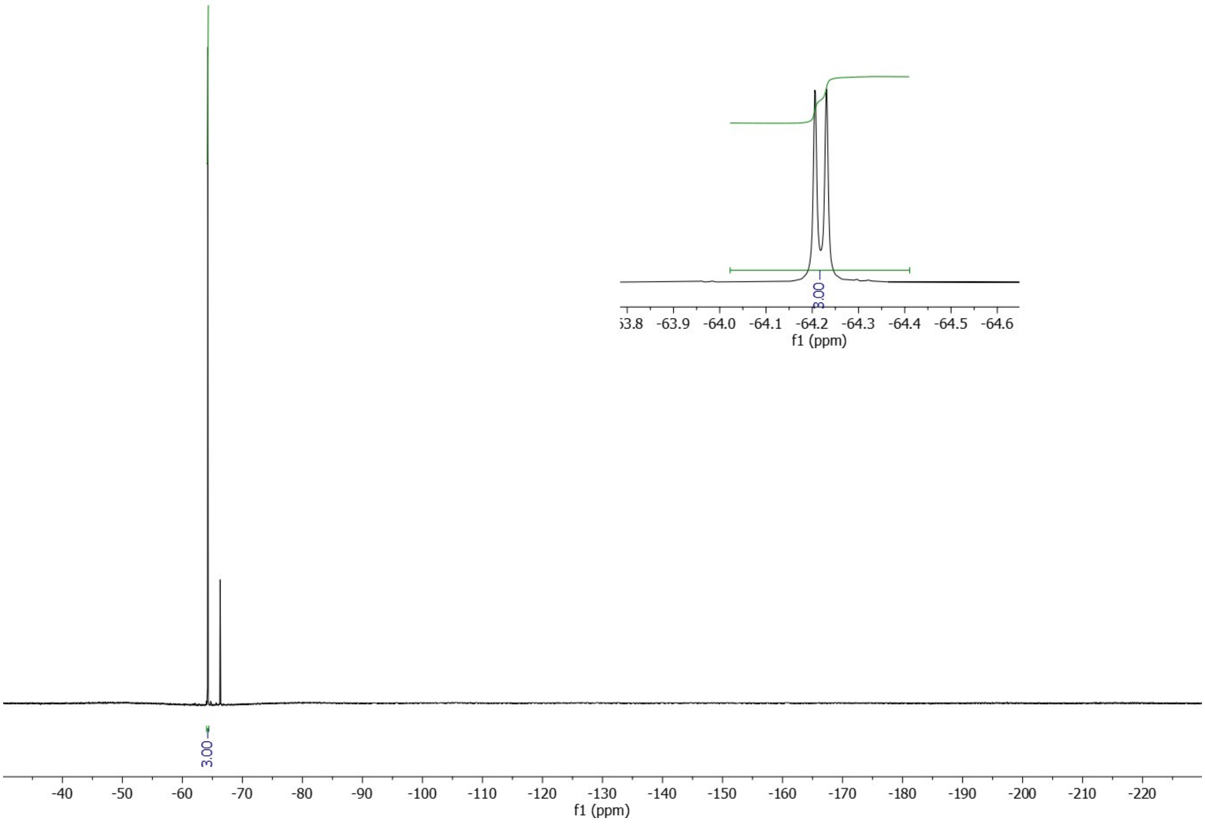

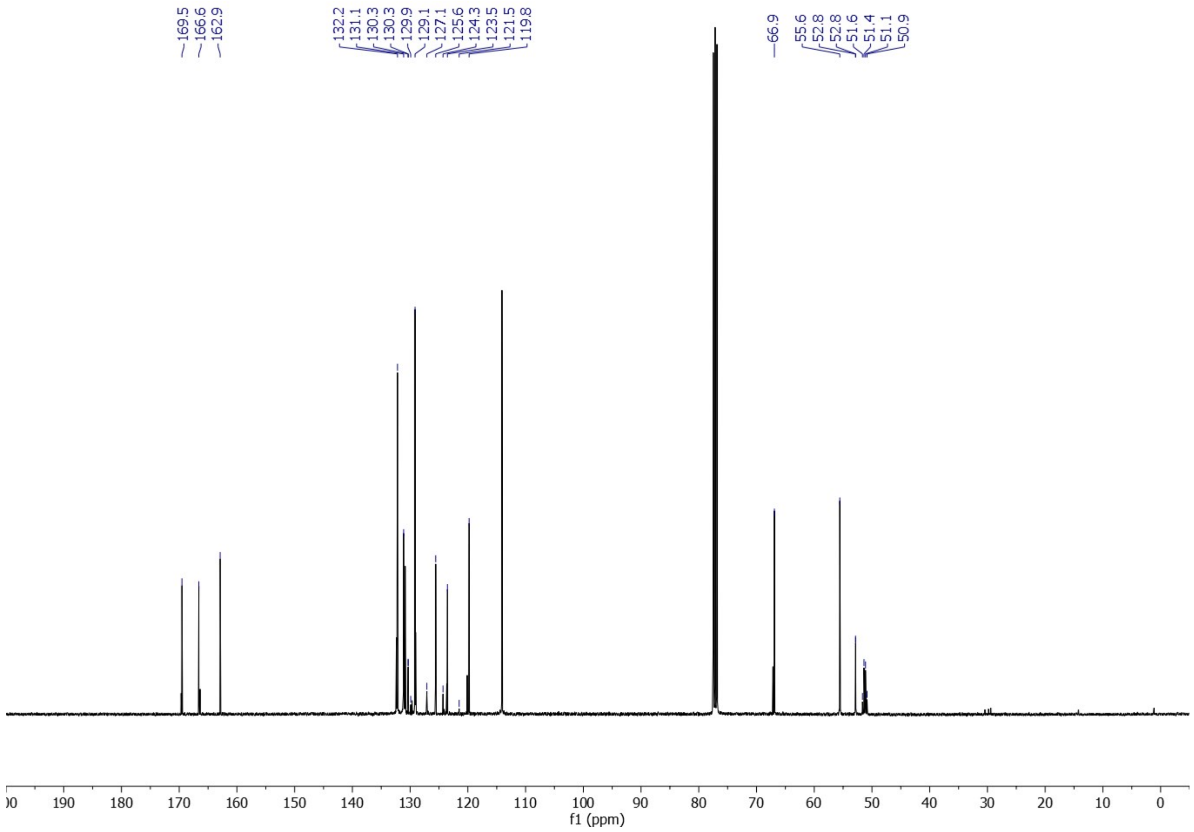
169.5
166.6
162.9
132.2
131.1
130.3
130.3
129.9
129.1
127.1
125.6
124.3
123.5
121.5
119.8
66.9
55.6
52.8
52.8
51.6
51.4
51.1
50.9
190
180
170
160
150
140
130
120
110
100
90
80
70
60
50
40
30
20
10
0
f1 (ppm)

**Allyl(2*R*,3*R*)-3-(4-chlorophenyl)-4,4,4-trifluoro-2-(4-methoxybenzamido)butanoate** *anti*-**4f**.

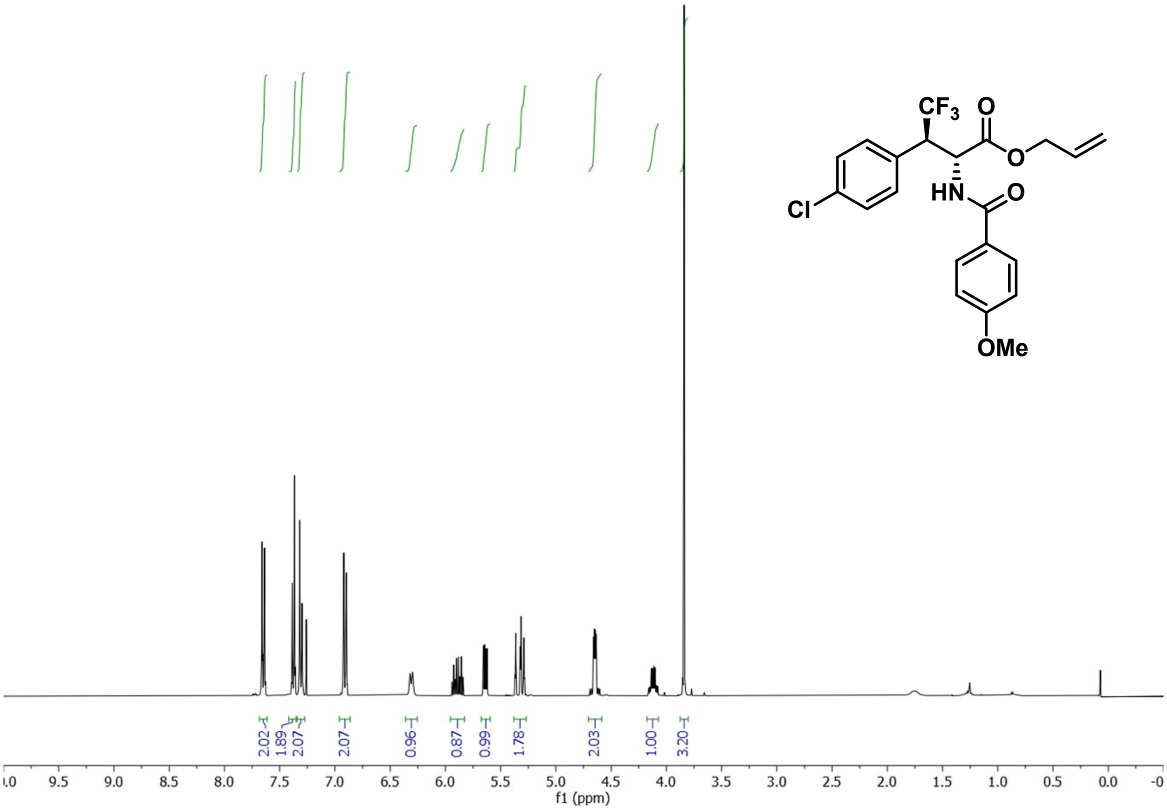

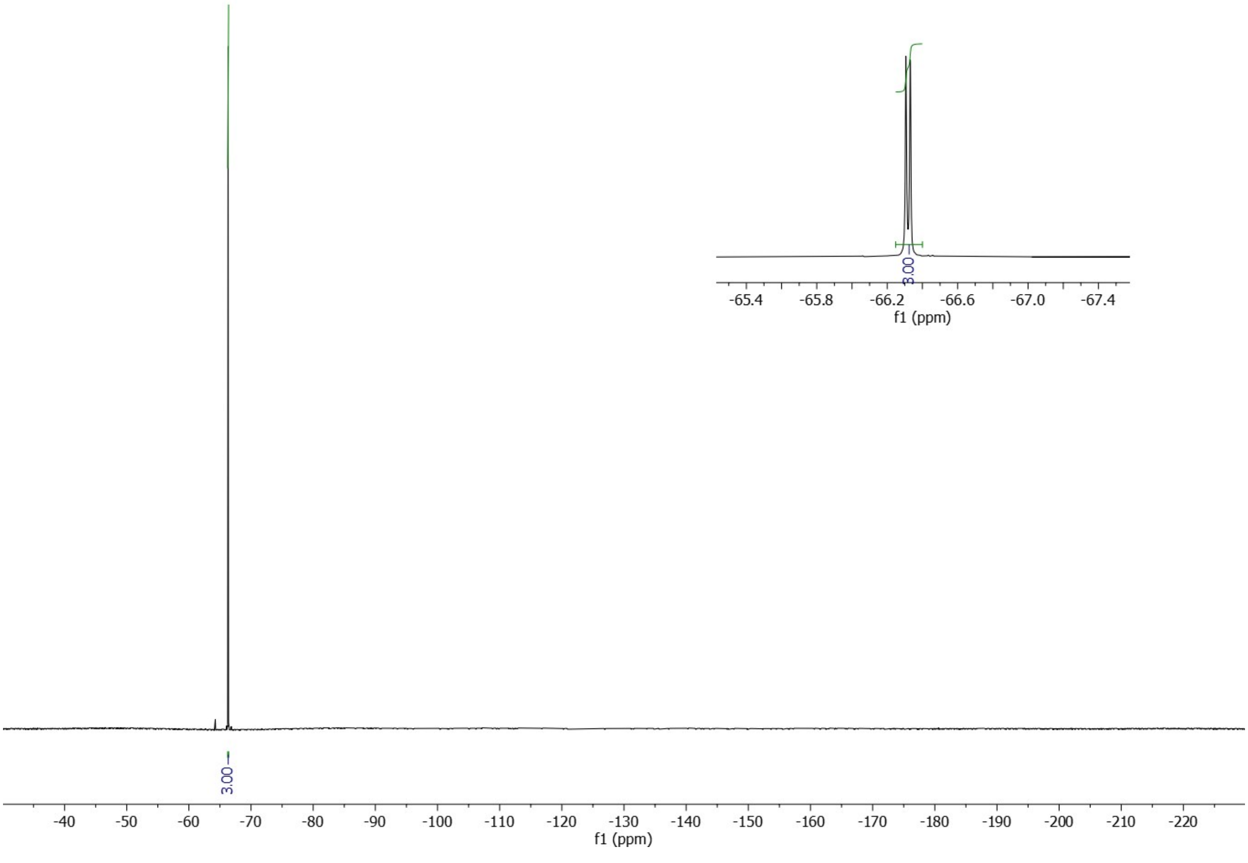

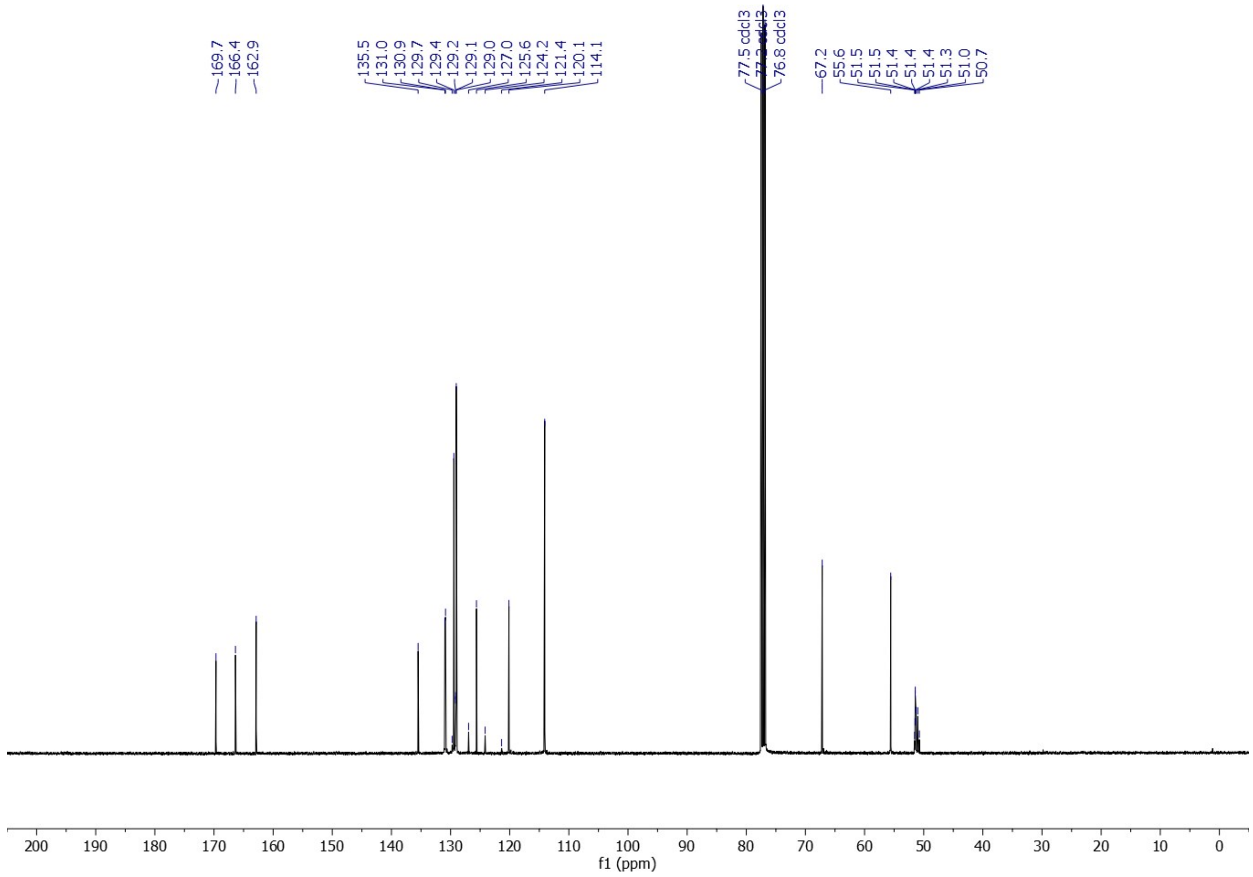
169.7
166.4
162.9
135.5
131.0
130.9
129.7
129.4
129.2
129.1
129.0
127.0
125.6
124.2
121.4
120.1
114.1
77.5 cdcl3
76.8 cdcl3
67.2
55.6
51.5
51.5
51.4
51.4
51.4
51.3
51.0
50.7
f1 (ppm)

**Allyl (2*S*,3*R*)-3-(4-chlorophenyl)-4,4,4-trifluoro-2-(4-methoxybenzamido)butanoate *syn*-4f.**

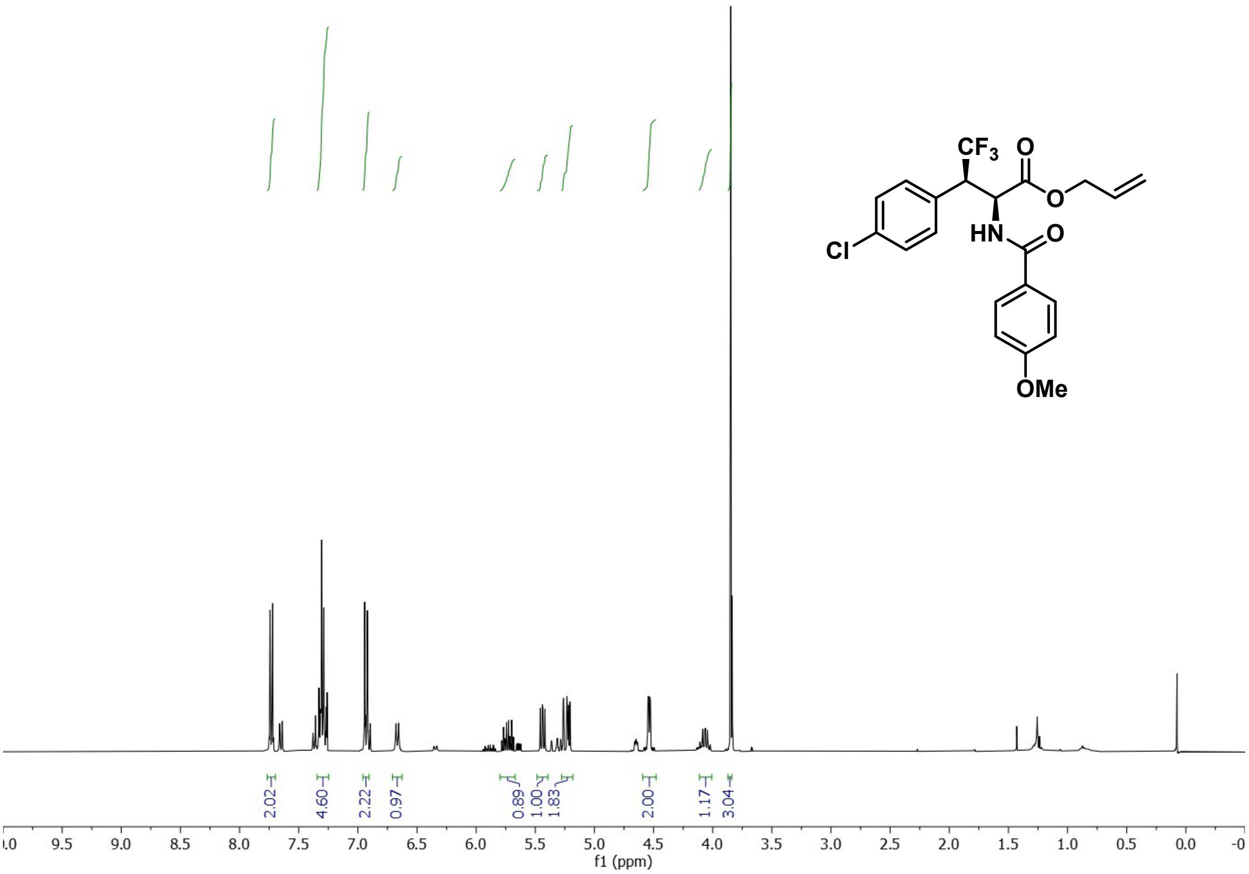

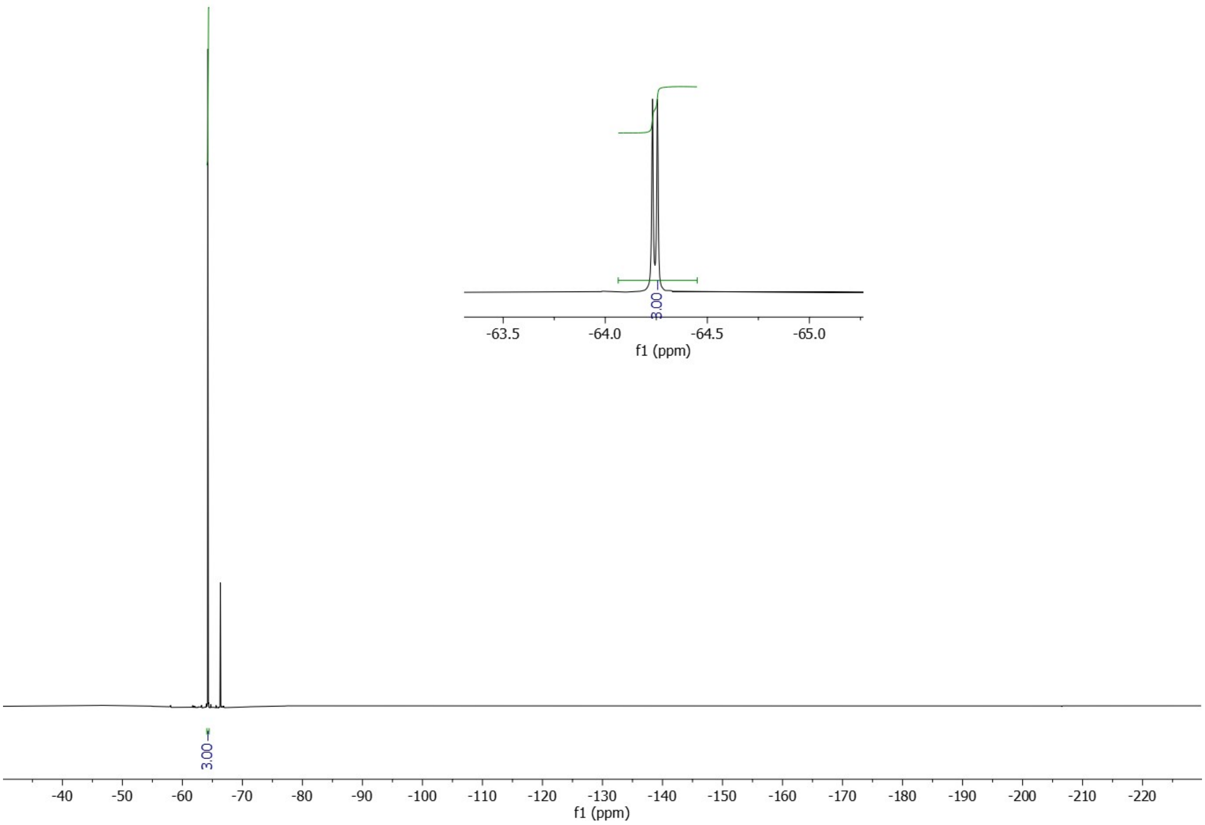
3.00
-63.5
-64.0
-64.5
-65.0
f1 (ppm)
3.00
-40
-50
-60
-70
-80
-90
-100
-110
-120
-130
-140
-150
-160
-170
-180
-190
-200
-210
-220
f1 (ppm)

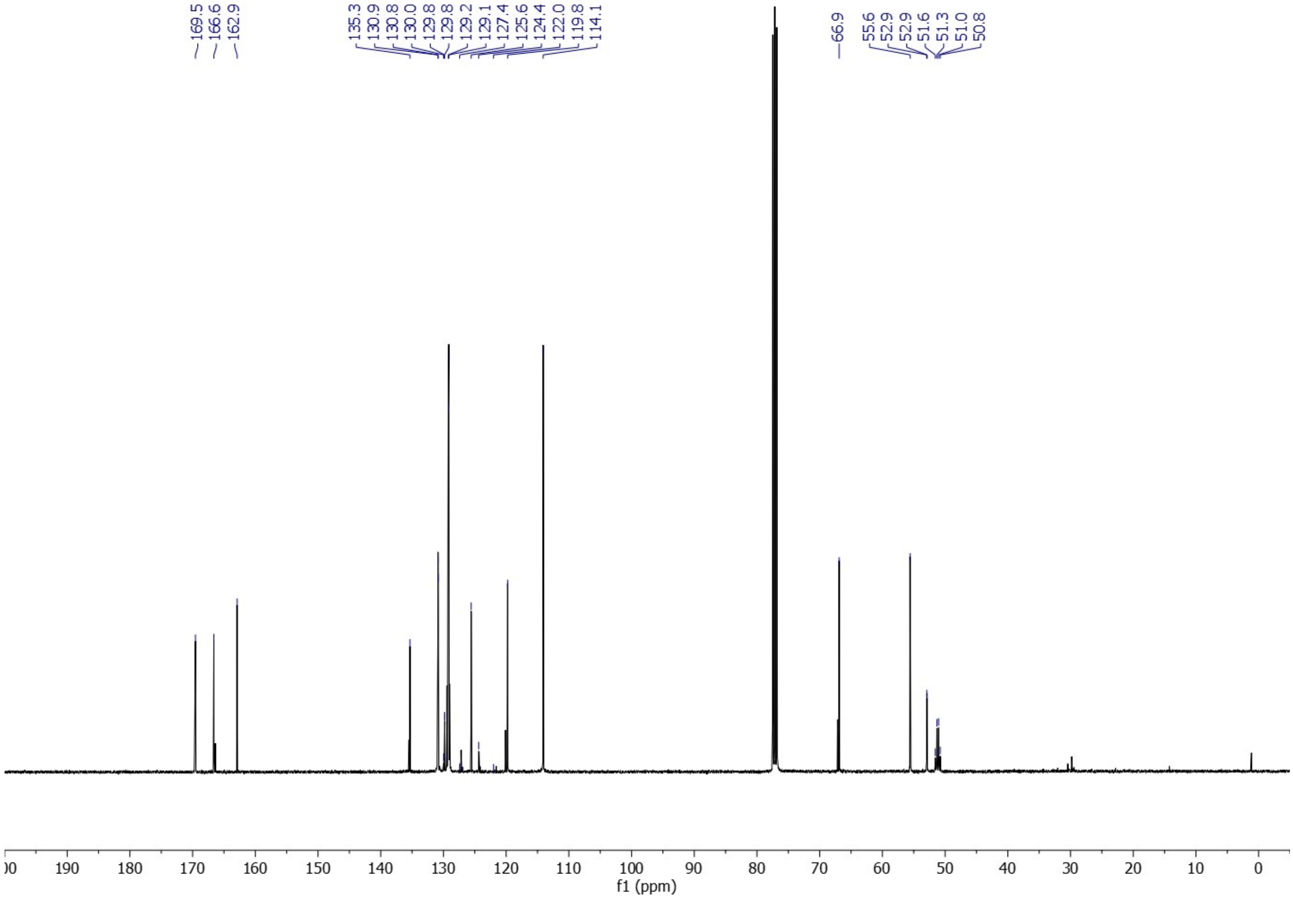
169.5
166.6
162.9
135.3
130.9
130.8
130.0
129.8
129.8
129.2
129.1
127.4
125.6
124.4
122.0
119.8
114.1
66.9
55.6
52.9
52.9
51.6
51.3
51.0
50.8
190
180
170
160
150
140
130
120
110
100
90
80
70
60
50
40
30
20
10
0
f1 (ppm)

**Allyl (2*R*,3*R*)-4,4,4-trifluoro-2-(4-methoxybenzamido)-3-(m-tolyl)butanoate *anti*-4g.**

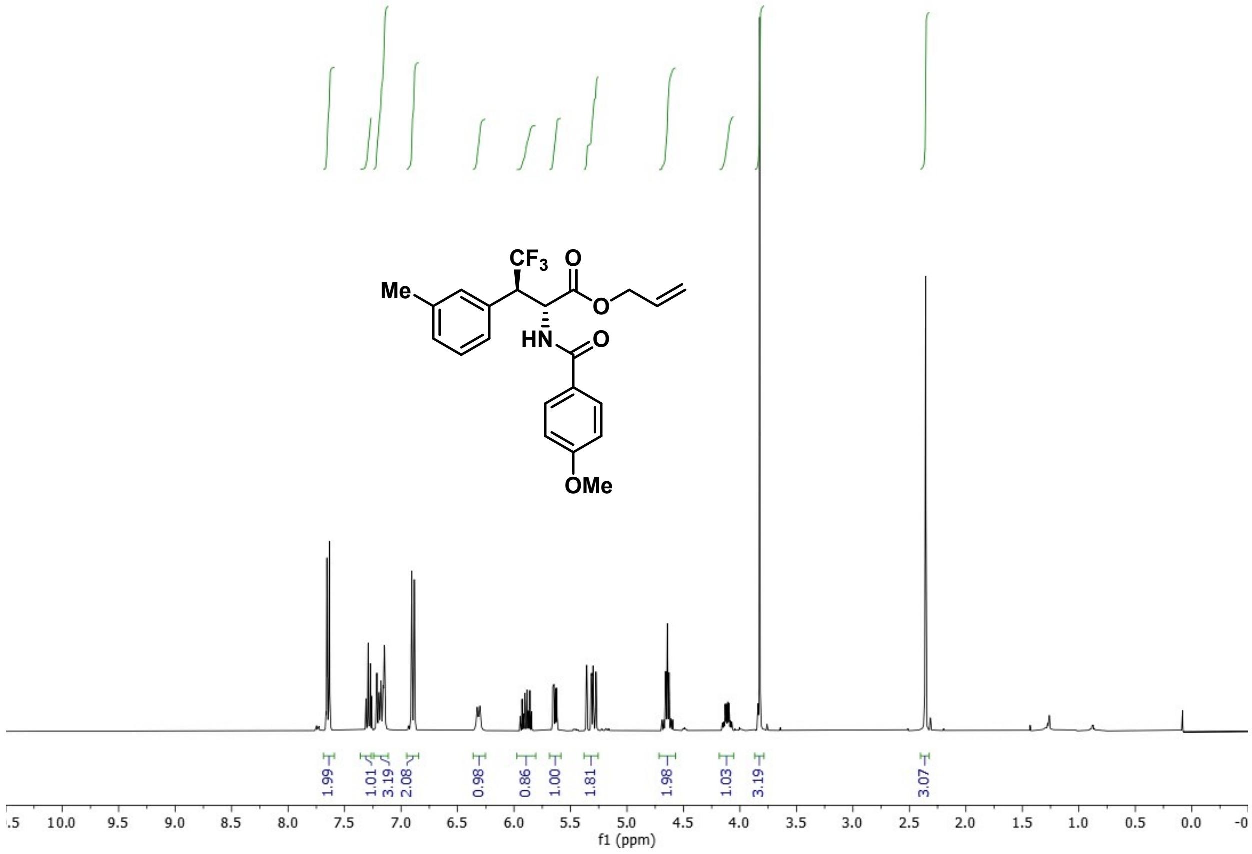


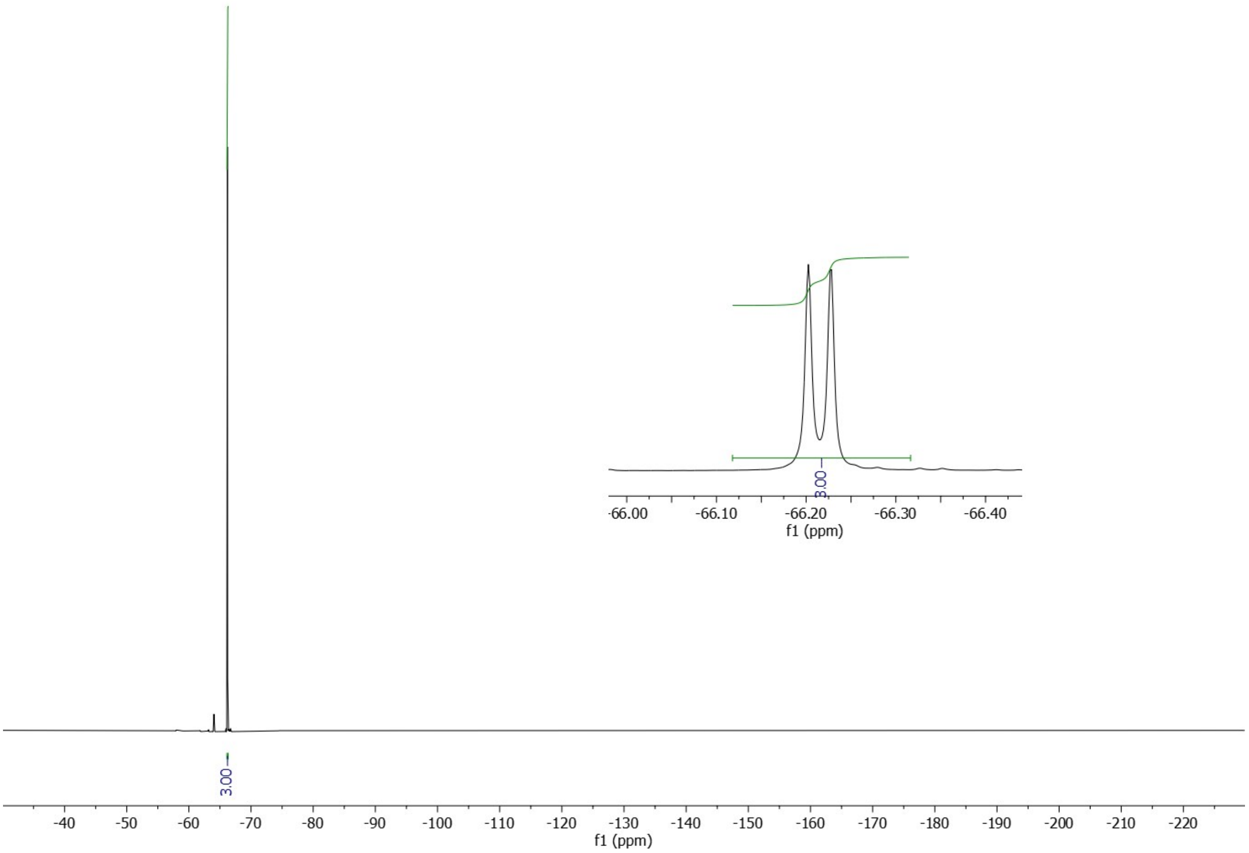

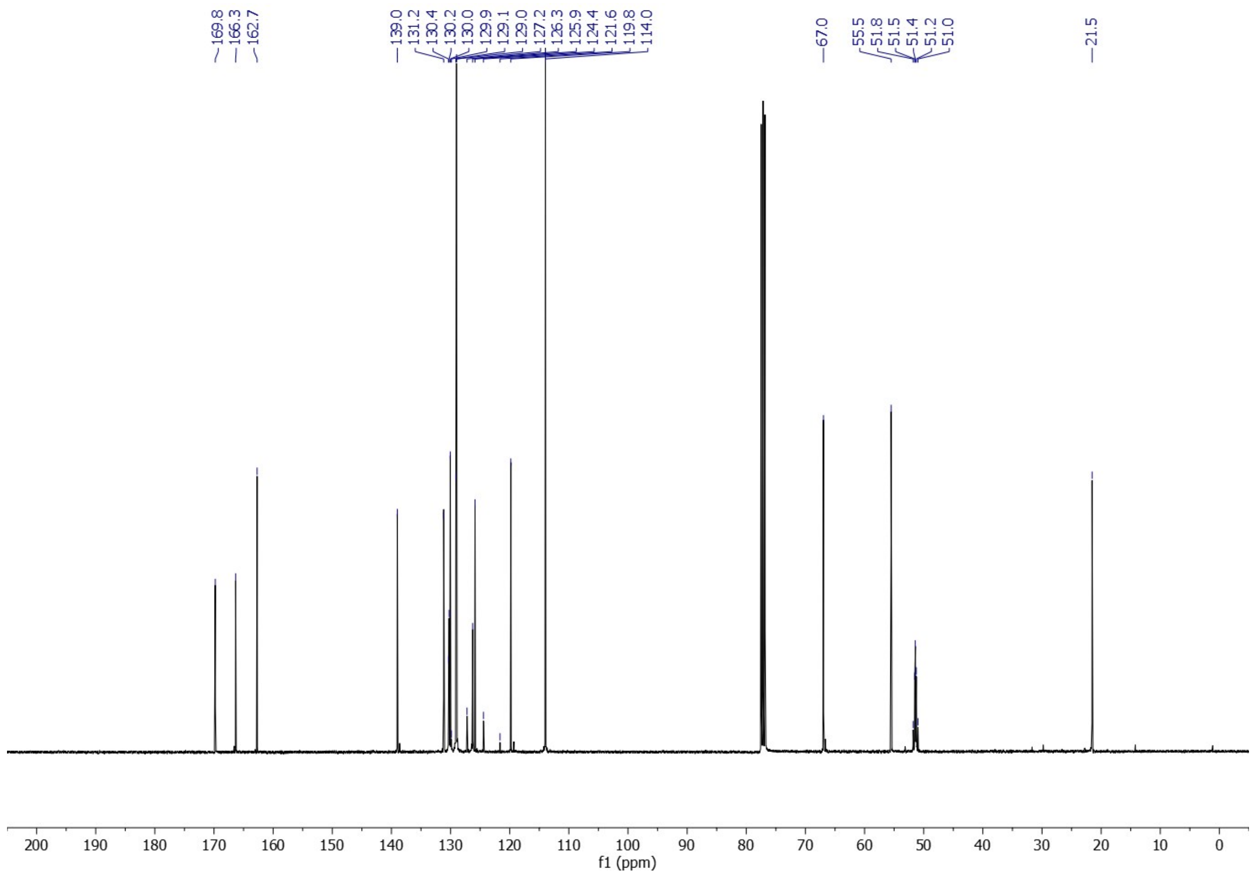
169.8
166.3
162.7
139.0
131.2
130.4
130.2
130.0
129.9
129.1
129.0
127.2
126.3
125.9
124.4
121.6
119.8
114.0
67.0
55.5
51.8
51.5
51.4
51.2
51.0
21.5
200
190
180
170
160
150
140
130
120
110
100
90
80
70
60
50
40
30
20
10
0
f1 (ppm)

**Allyl (2*S*,3*R*)-4,4,4-trifluoro-2-(4-methoxybenzamido)-3-(m-tolyl)butanoate *syn*-4g.**

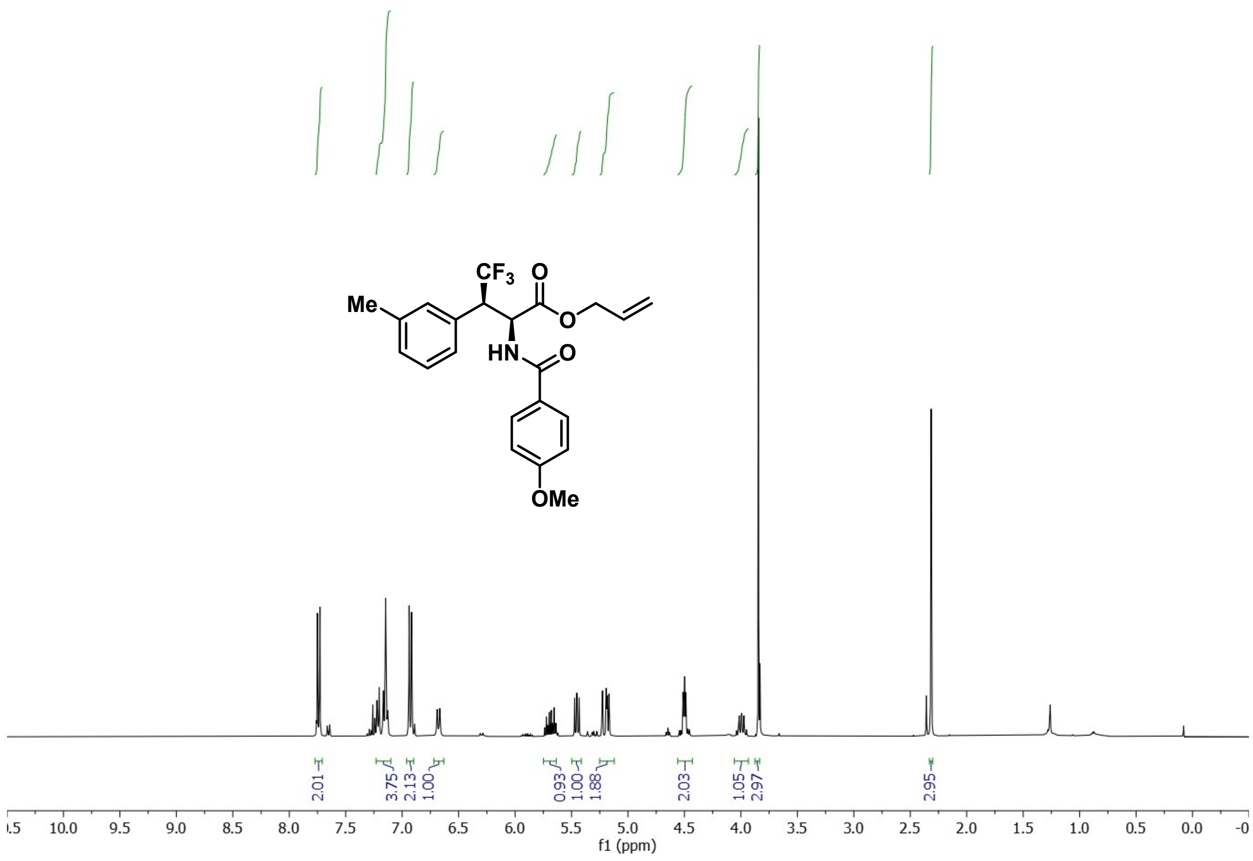

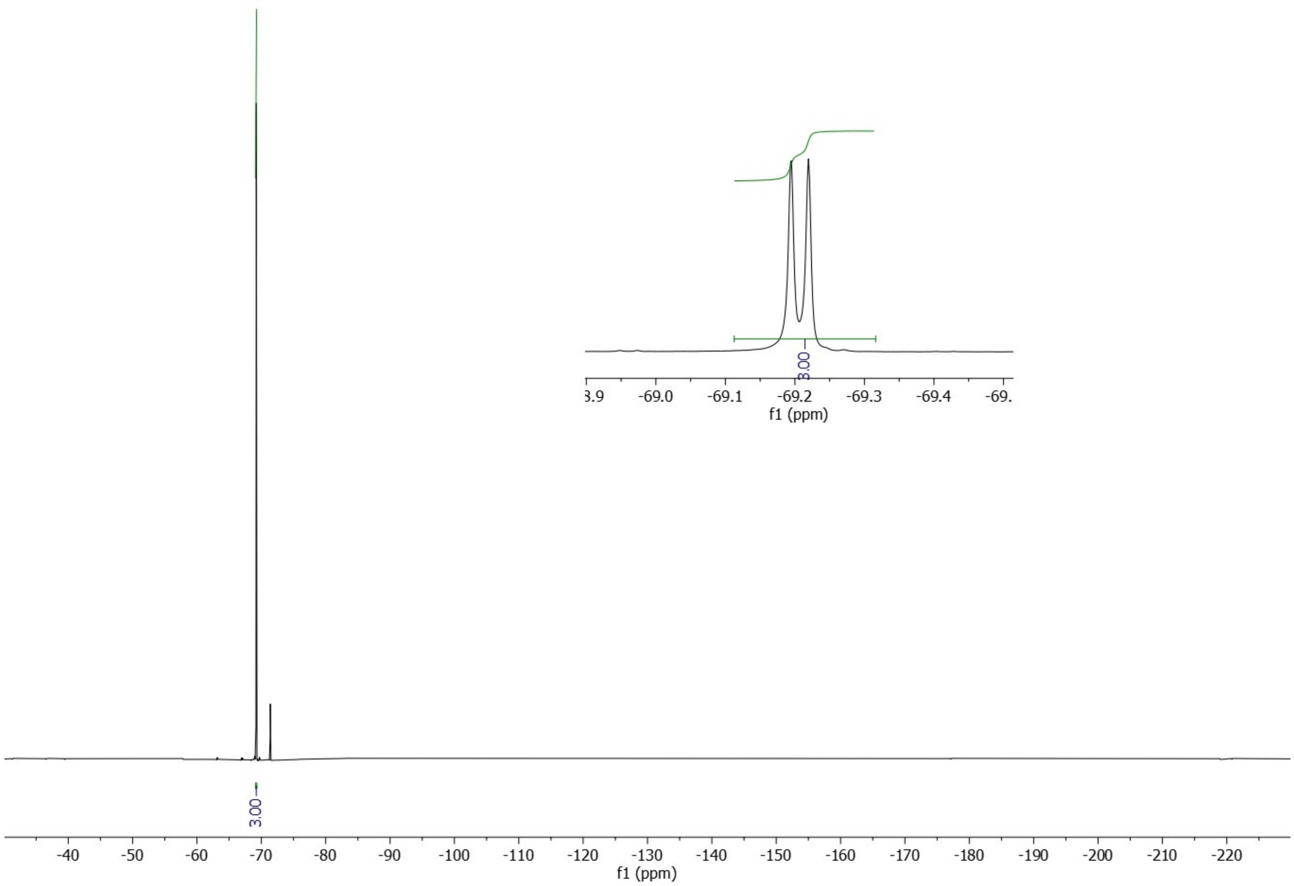
3.00
-69.0
-69.1
-69.2
-69.3
-69.4
f1 (ppm)
3.00
-40
-50
-60
-70
-80
-90
-100
-110
-120
-130
-140
-150
-160
-170
-180
-190
-200
-210
-220
f1 (ppm)

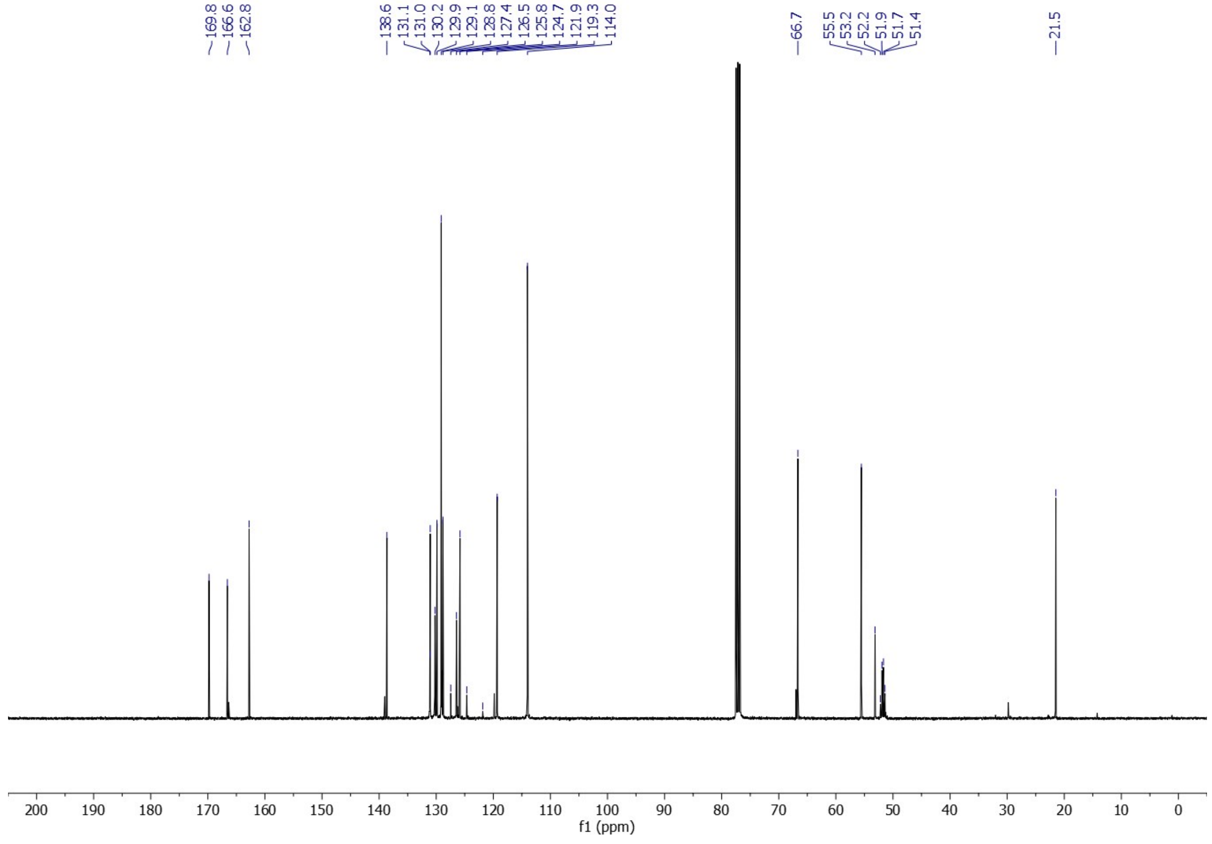
169.8
166.6
162.8
138.6
131.1
131.0
130.2
129.9
129.1
128.8
127.4
126.5
125.8
124.7
121.9
119.3
114.0
66.7
55.5
53.2
52.2
51.9
51.7
51.4
21.5
200
190
180
170
160
150
140
130
120
110
100
90
80
70
60
50
40
30
20
10
0
f1 (ppm)

**Allyl (2*R*,3*R*)-4,4,4-trifluoro-3-(4-fluorophenyl)-2-(4-methoxybenzamido)butanoate** *anti*-**4h**.

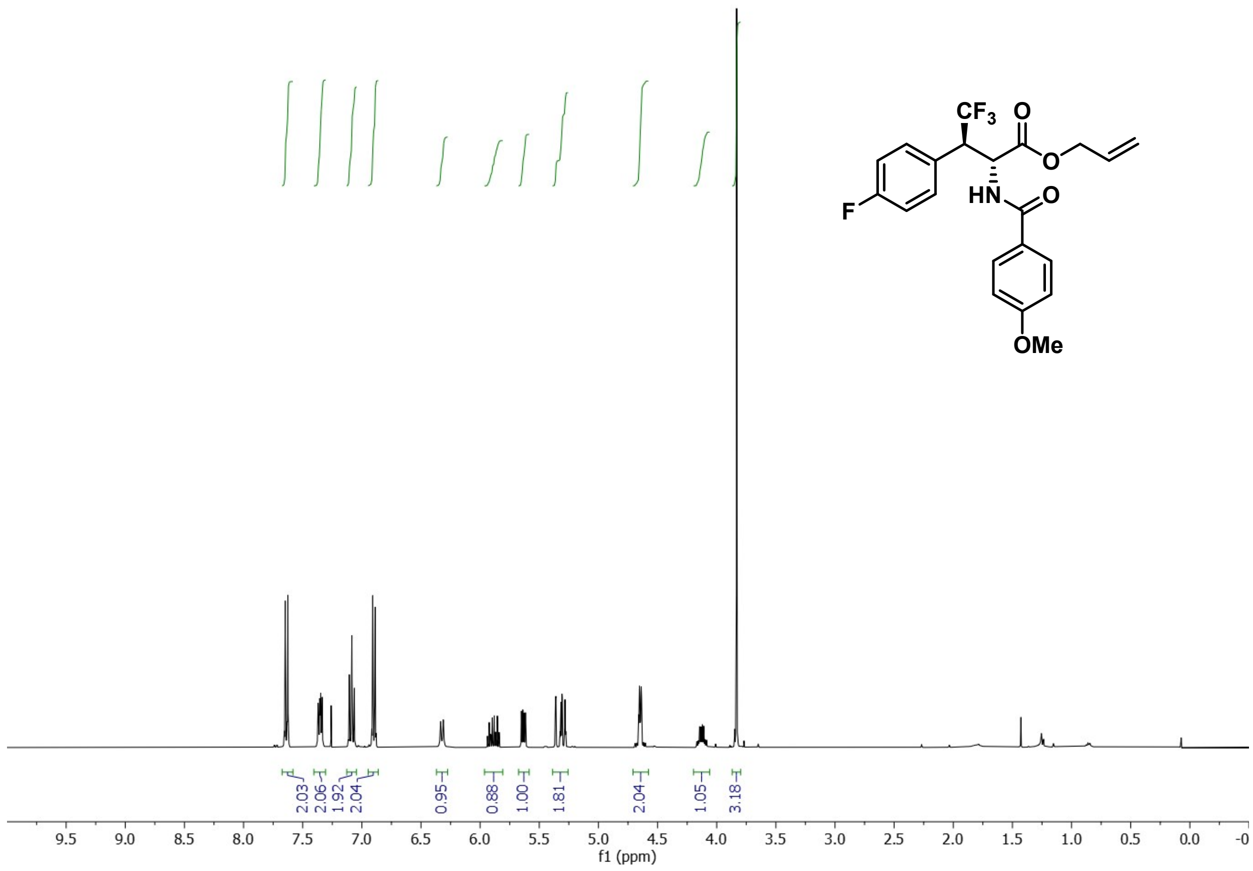

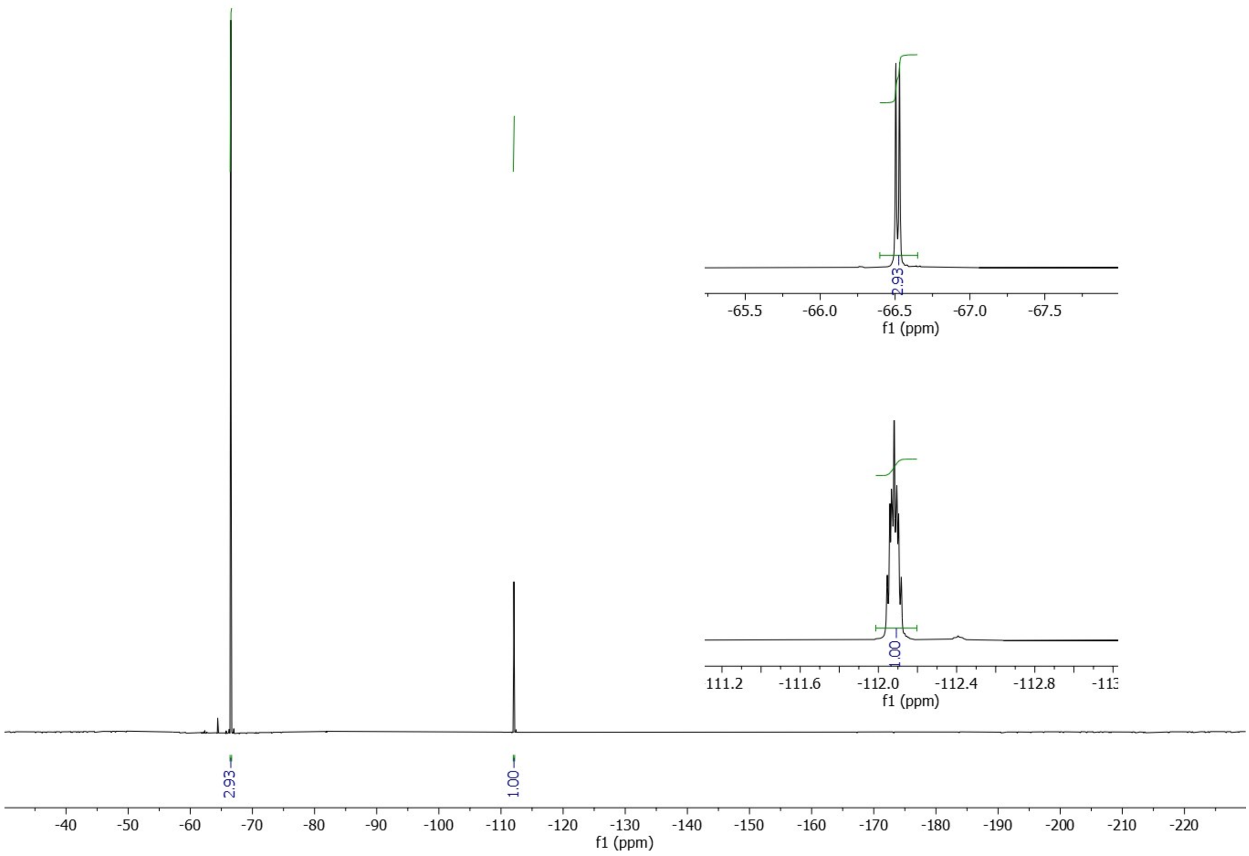

2.93
-65.5 -66.0 -66.5 -67.0 -67.5
f1 (ppm)
1.00
111.2 -111.6 -112.0 -112.4 -112.8
f1 (ppm)
2.93
1.00
-40 -50 -60 -70 -80 -90 -100 -110 -120 -130 -140 -150 -160 -170 -180 -190 -200 -210 -220
f1 (ppm)


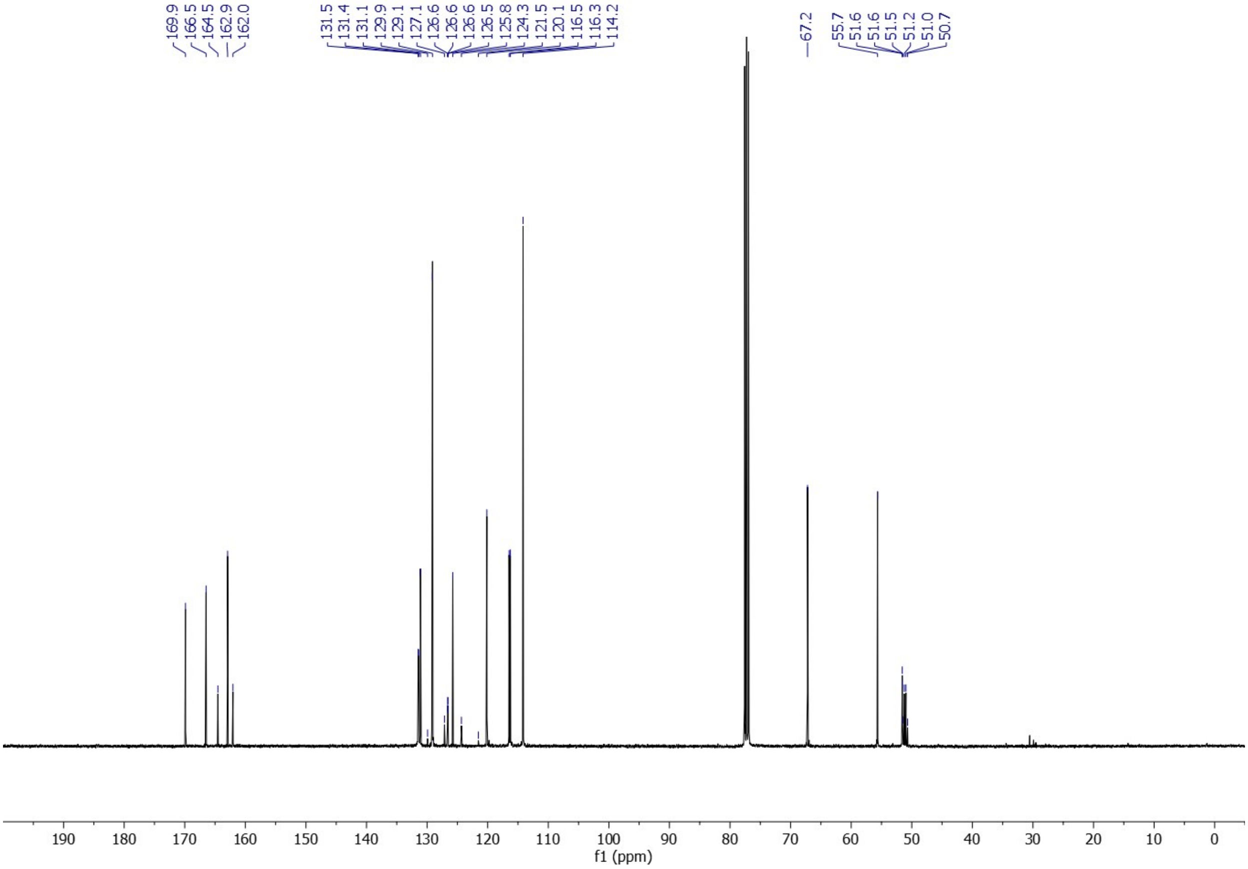

169.9
166.5
164.5
162.9
162.0
131.5
131.4
131.1
129.9
129.1
127.1
126.6
126.6
126.6
126.5
125.8
124.3
121.5
120.1
116.5
116.3
114.2
67.2
55.7
51.6
51.6
51.5
51.2
51.0
50.7
190 180 170 160 150 140 130 120 110 100 90 80 70 60 50 40 30 20 10 0
f1 (ppm)

**Allyl (2*S*,3*R*)-4,4,4-trifluoro-3-(4-fluorophenyl)-2-(4-methoxybenzamido)butanoate** *syn*-**4h**.

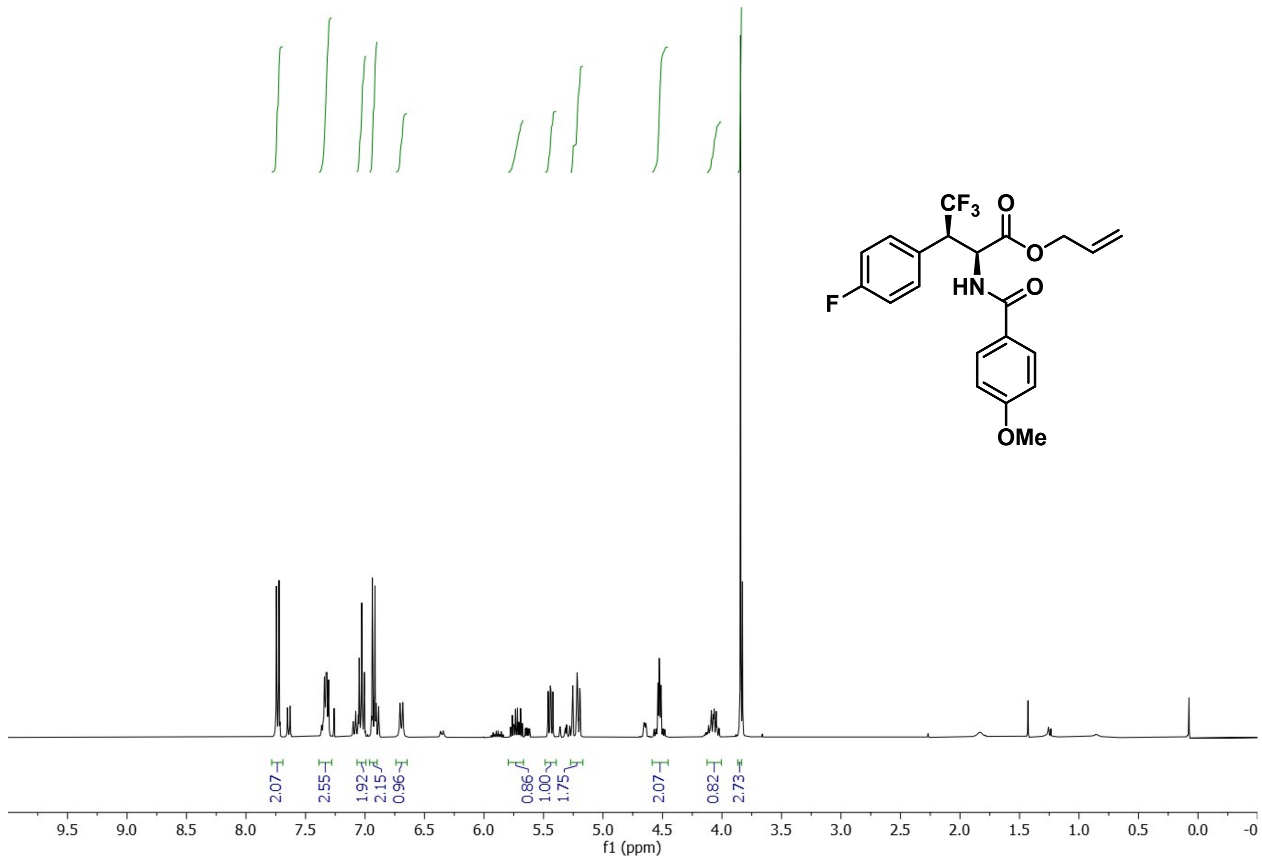

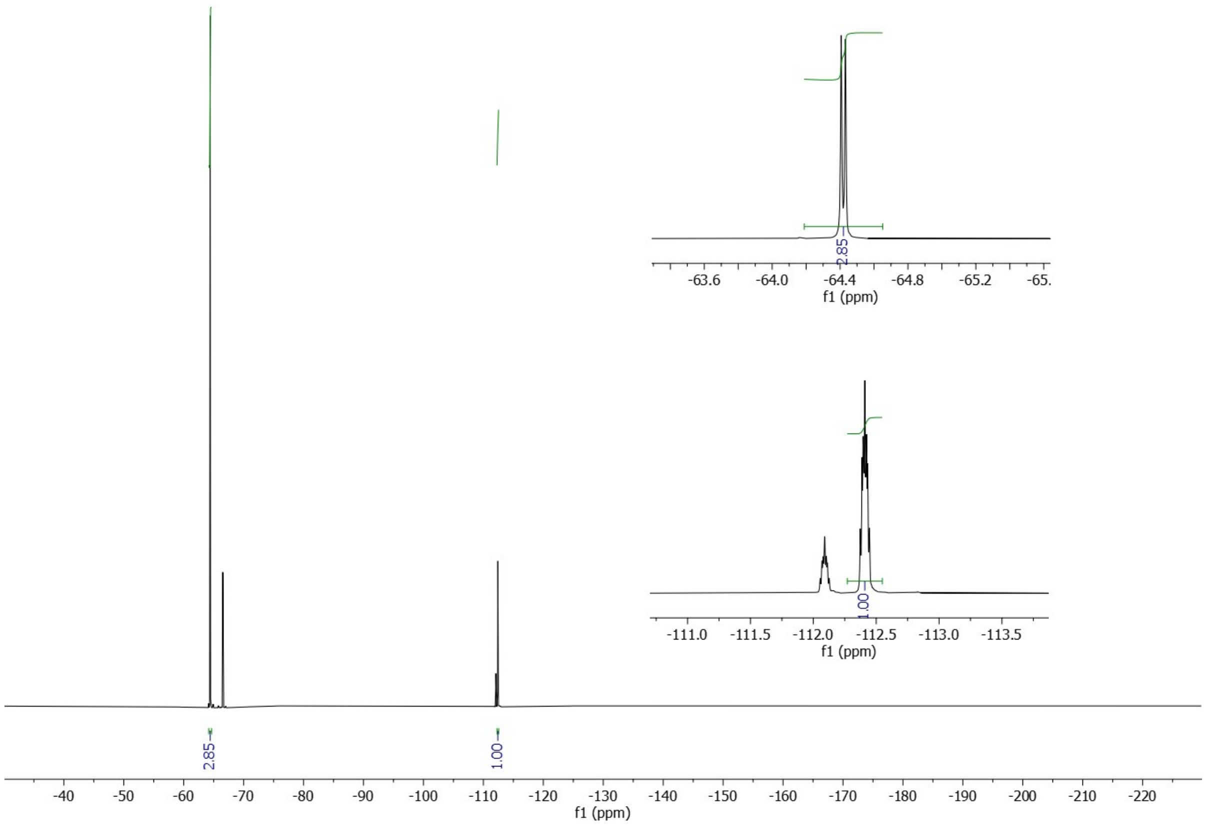
2.85
1.00
-40 -50 -60 -70 -80 -90 -100 -110 -120 -130 -140 -150 -160 -170 -180 -190 -200 -210 -220
f1 (ppm)
-63.6 -64.0 -64.4 -64.8 -65.2
f1 (ppm)
-111.0 -111.5 -112.0 -112.5 -113.0 -113.5
f1 (ppm)

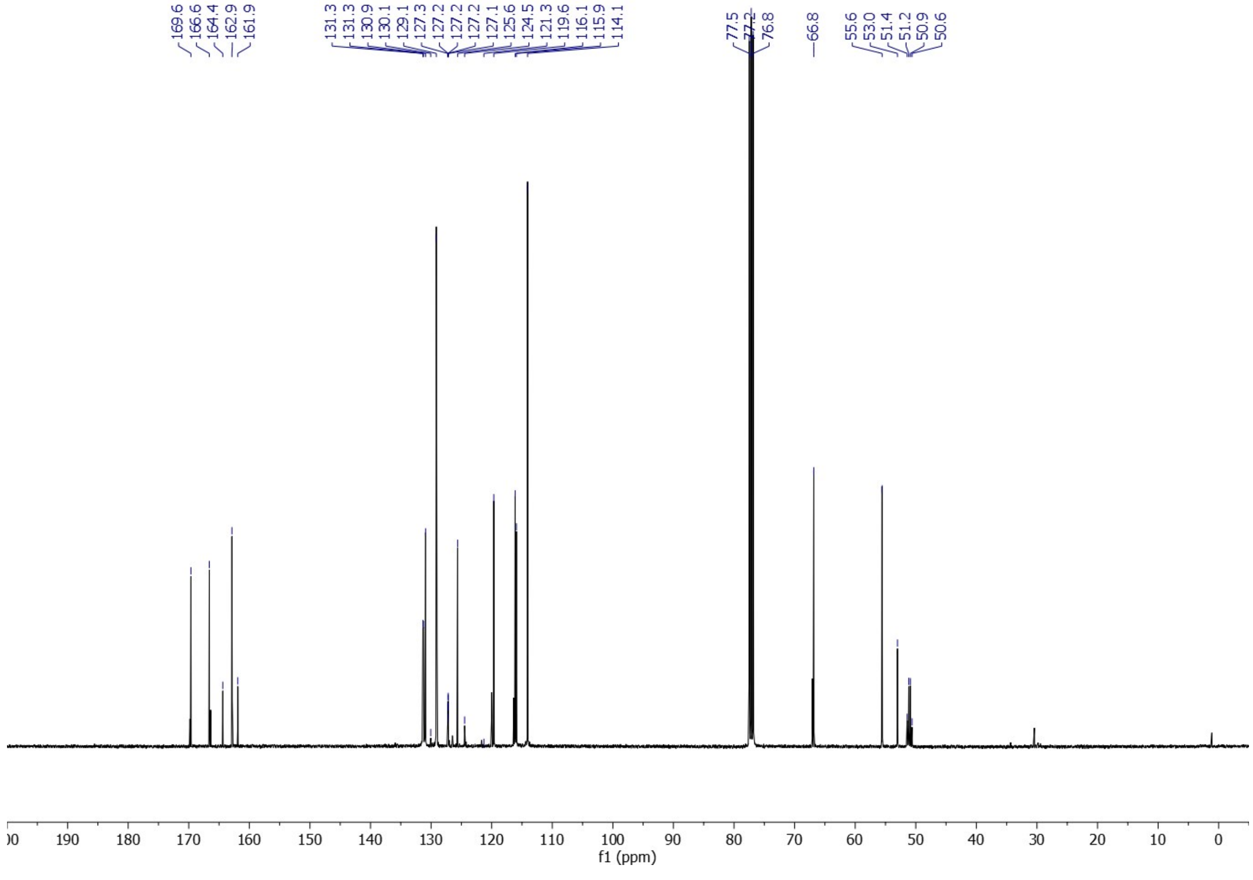
169.6
166.6
164.4
162.9
161.9
131.3
131.3
130.9
130.1
129.1
127.3
127.2
127.2
127.2
127.1
125.6
124.5
121.3
119.6
116.1
115.9
114.1
77.5
76.8
66.8
55.6
53.0
51.4
51.2
50.9
50.6
190 180 170 160 150 140 130 120 110 100 90 80 70 60 50 40 30 20 10 0
f1 (ppm)

**Allyl (2*R*,3*R*)-4,4,4-trifluoro-2-(4-methoxybenzamido)-3-(4-methoxyphenyl)butanoate** *anti*-**4i**.

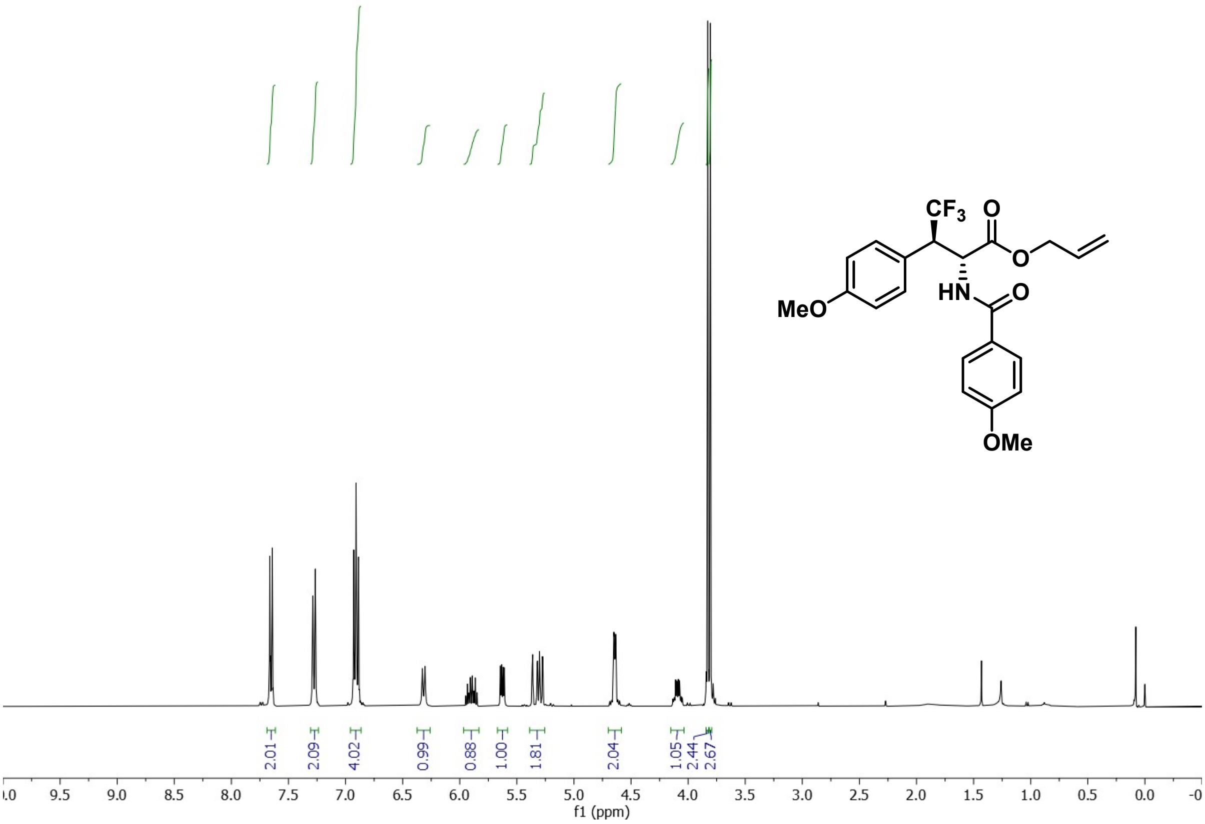

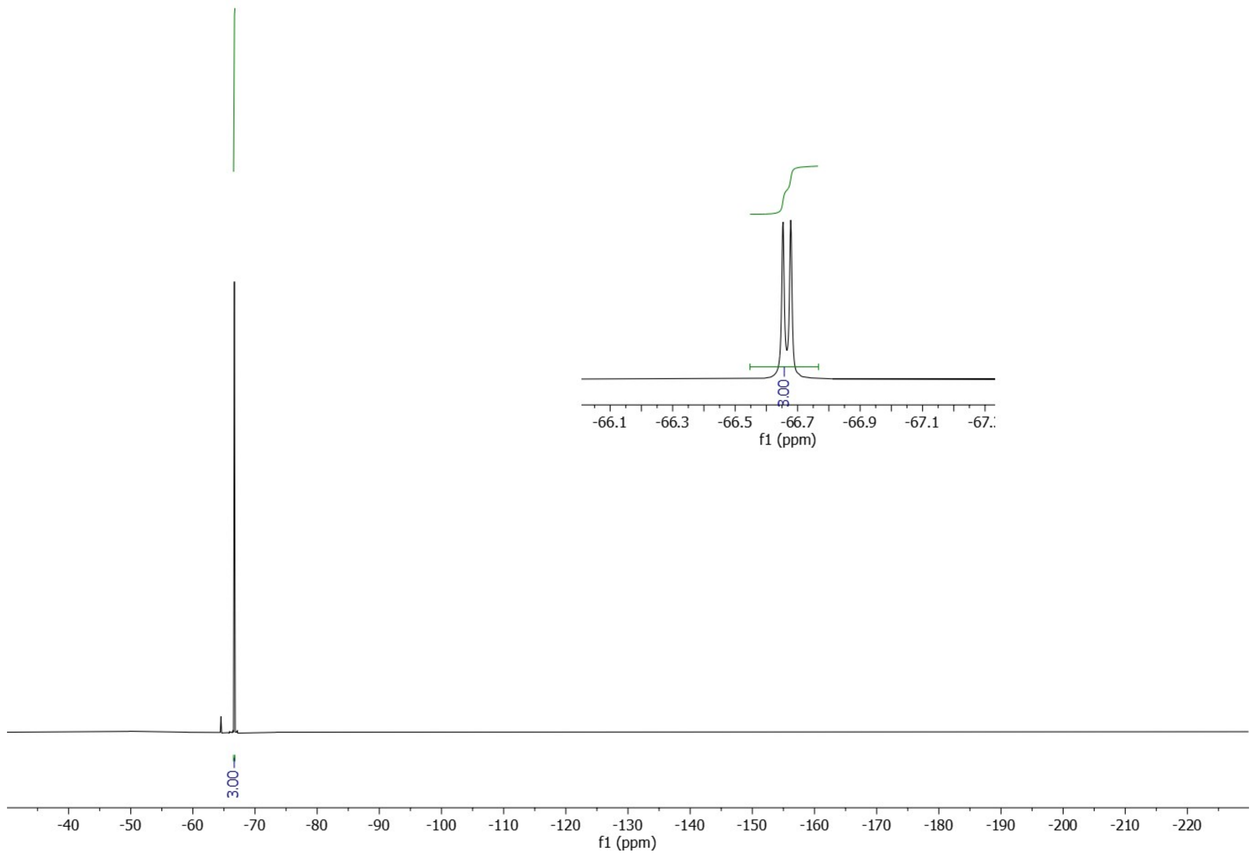
3.00
-66.1
-66.3
-66.5
-66.7
-66.9
-67.1
f1 (ppm)
-40
-50
-60
-70
-80
-90
-100
-110
-120
-130
-140
-150
-160
-170
-180
-190
-200
-210
-220

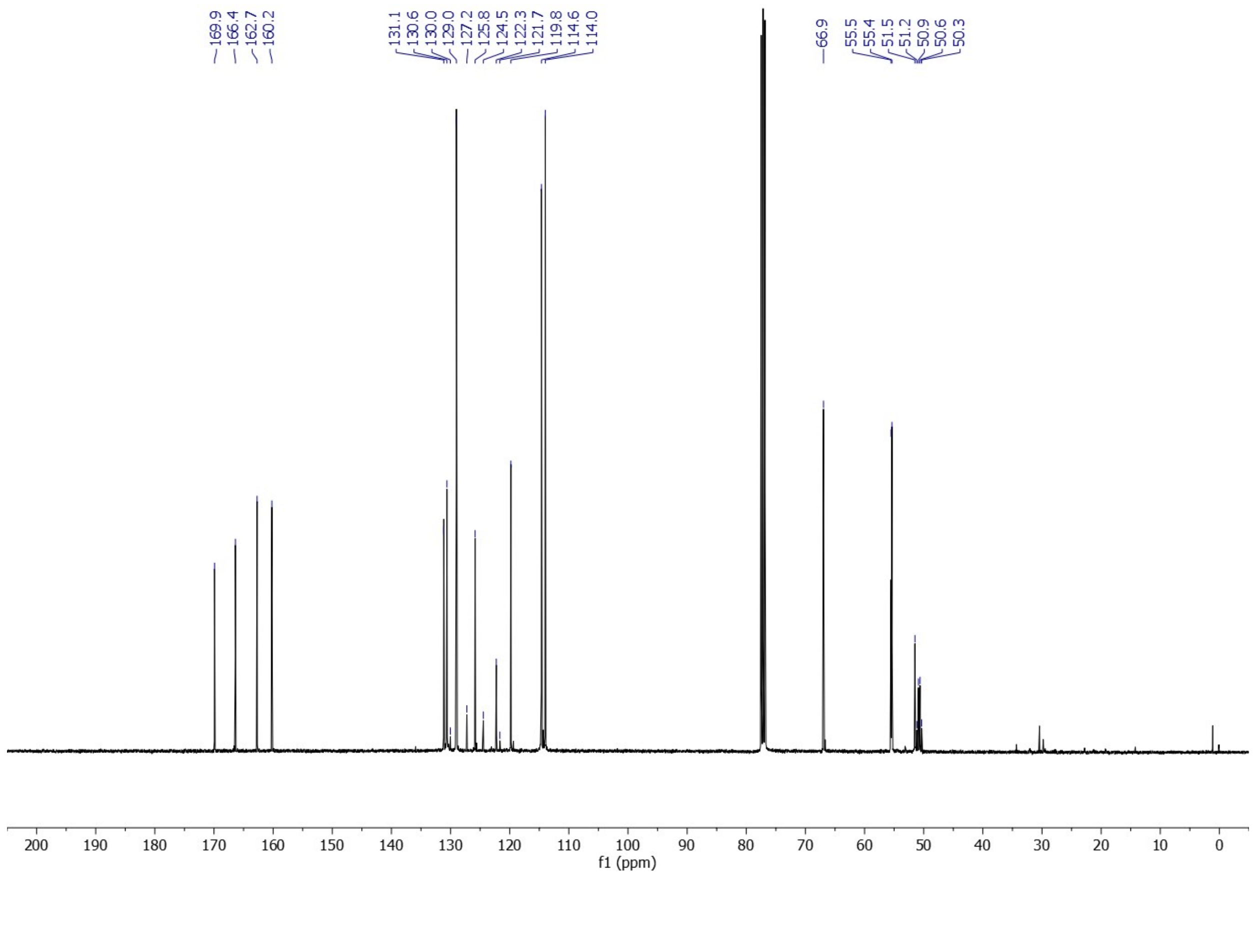
169.9
166.4
162.7
160.2
131.1
130.6
130.0
129.0
127.2
125.8
124.5
122.3
121.7
119.8
114.6
114.0
66.9
55.5
55.4
51.5
51.2
50.9
50.6
50.3
200
190
180
170
160
150
140
130
120
110
100
90
80
70
60
50
40
30
20
10
0
f1 (ppm)

**Allyl (2*S*,3*R*)-4,4,4-trifluoro-2-(4-methoxybenzamido)-3-(4-methoxyphenyl)butanoate *syn*-4i.**

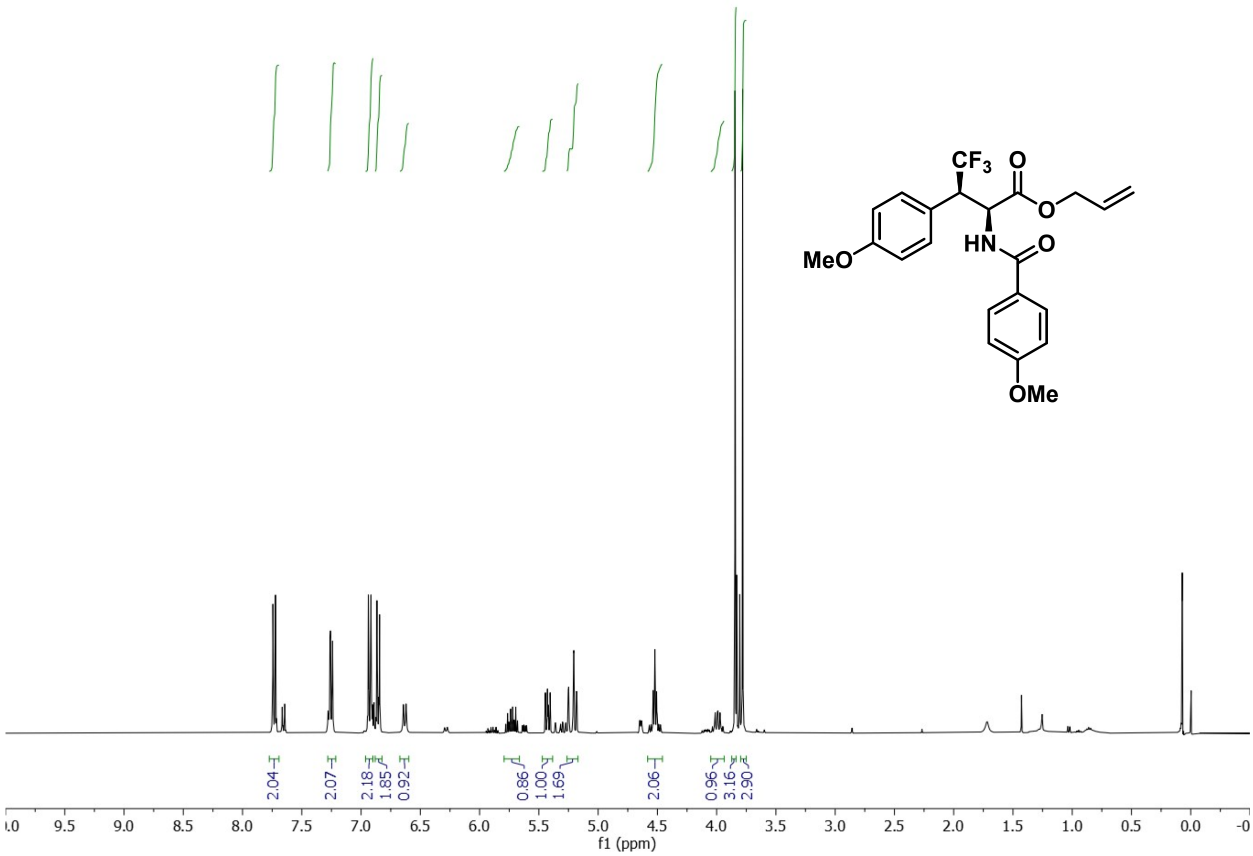

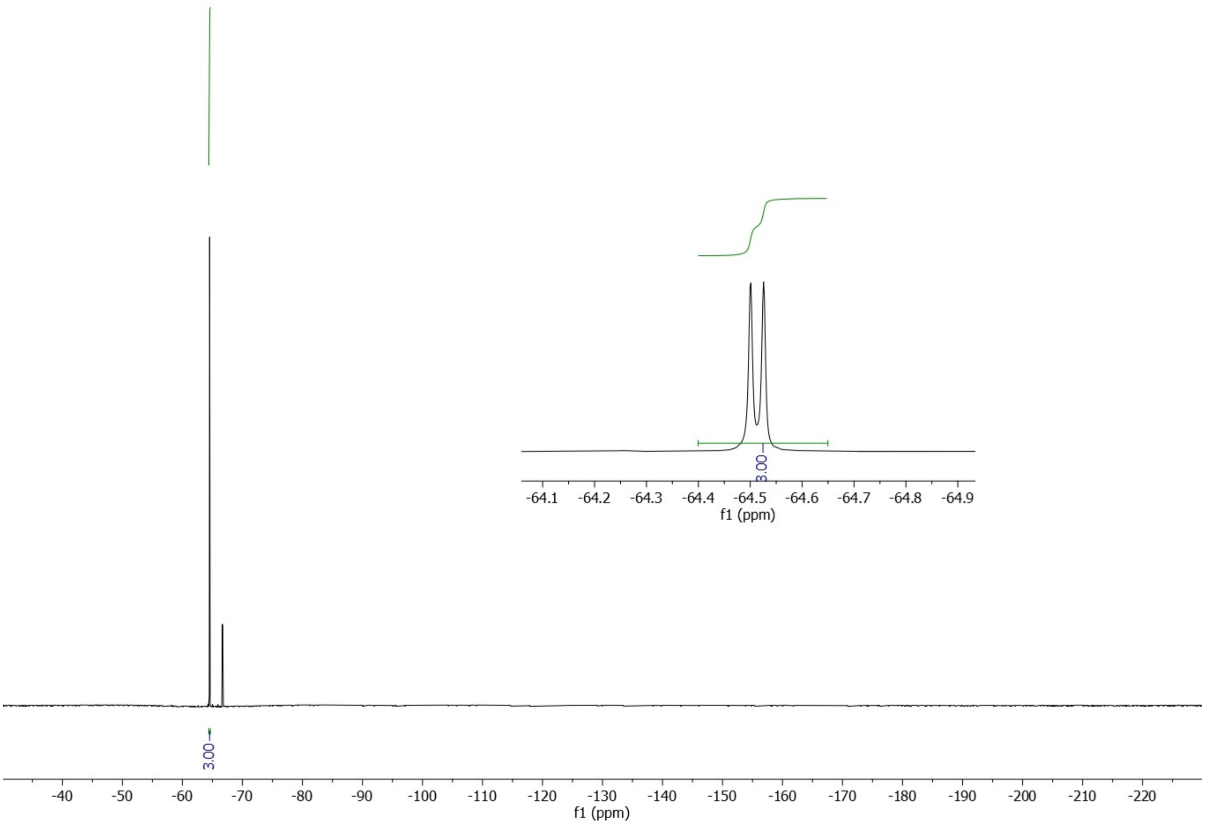
3.00
-64.1 -64.2 -64.3 -64.4 -64.5 -64.6 -64.7 -64.8 -64.9
f1 (ppm)
3.00
-40 -50 -60 -70 -80 -90 -100 -110 -120 -130 -140 -150 -160 -170 -180 -190 -200 -210 -220
f1 (ppm)

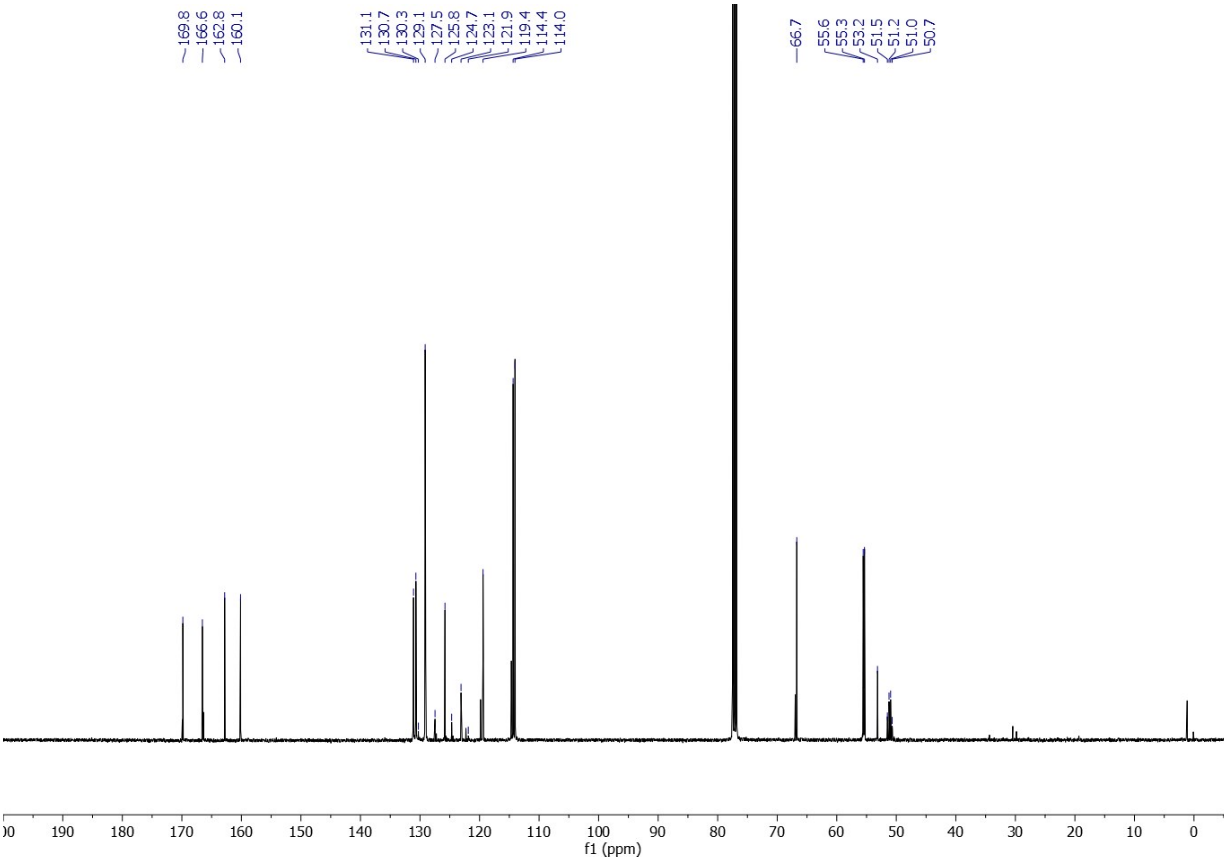
169.8
166.6
162.8
160.1
131.1
130.7
130.3
129.1
127.5
125.8
124.7
123.1
121.9
119.4
114.4
114.0
66.7
55.6
55.3
53.2
51.5
51.2
51.0
50.7
190 180 170 160 150 140 130 120 110 100 90 80 70 60 50 40 30 20 10 0
f1 (ppm)

**Allyl (2*R*,3R)-3-(3,5-difluorophenyl)-4,4,4-trifluoro-2-(4-methoxybenzamido)butanoate** *anti*-**4j**.

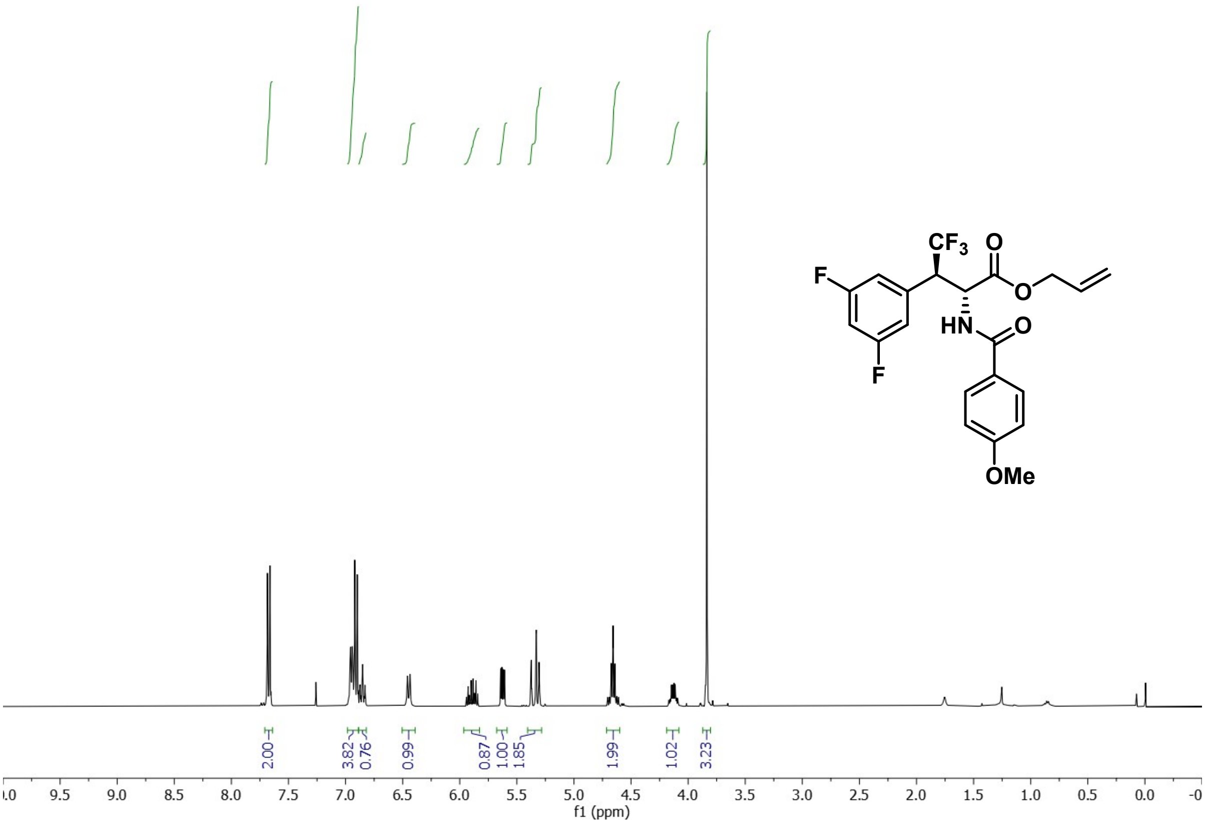

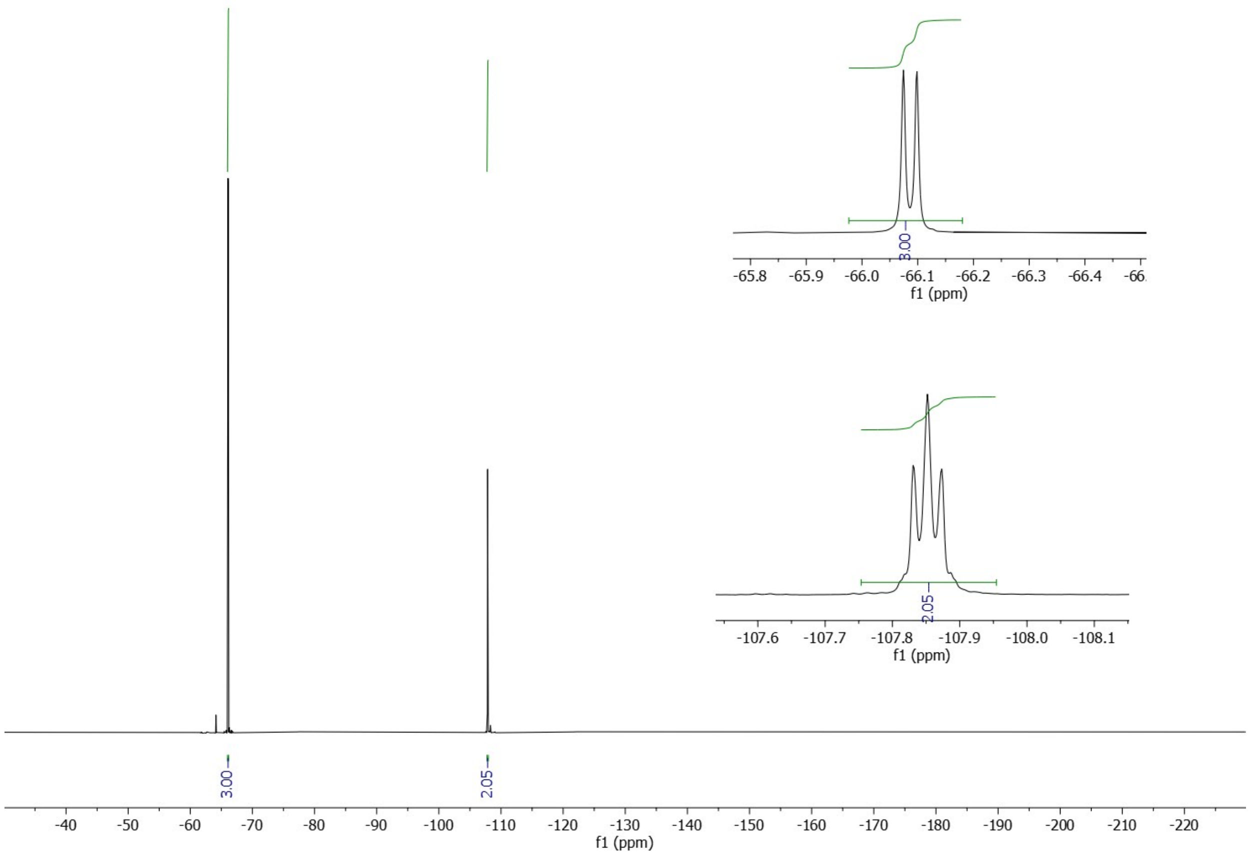
3.00
2.05
-40 -50 -60 -70 -80 -90 -100 -110 -120 -130 -140 -150 -160 -170 -180 -190 -200 -210 -220
f1 (ppm)
-65.8 -65.9 -66.0 -66.1 -66.2 -66.3 -66.4
-107.6 -107.7 -107.8 -107.9 -108.0 -108.1

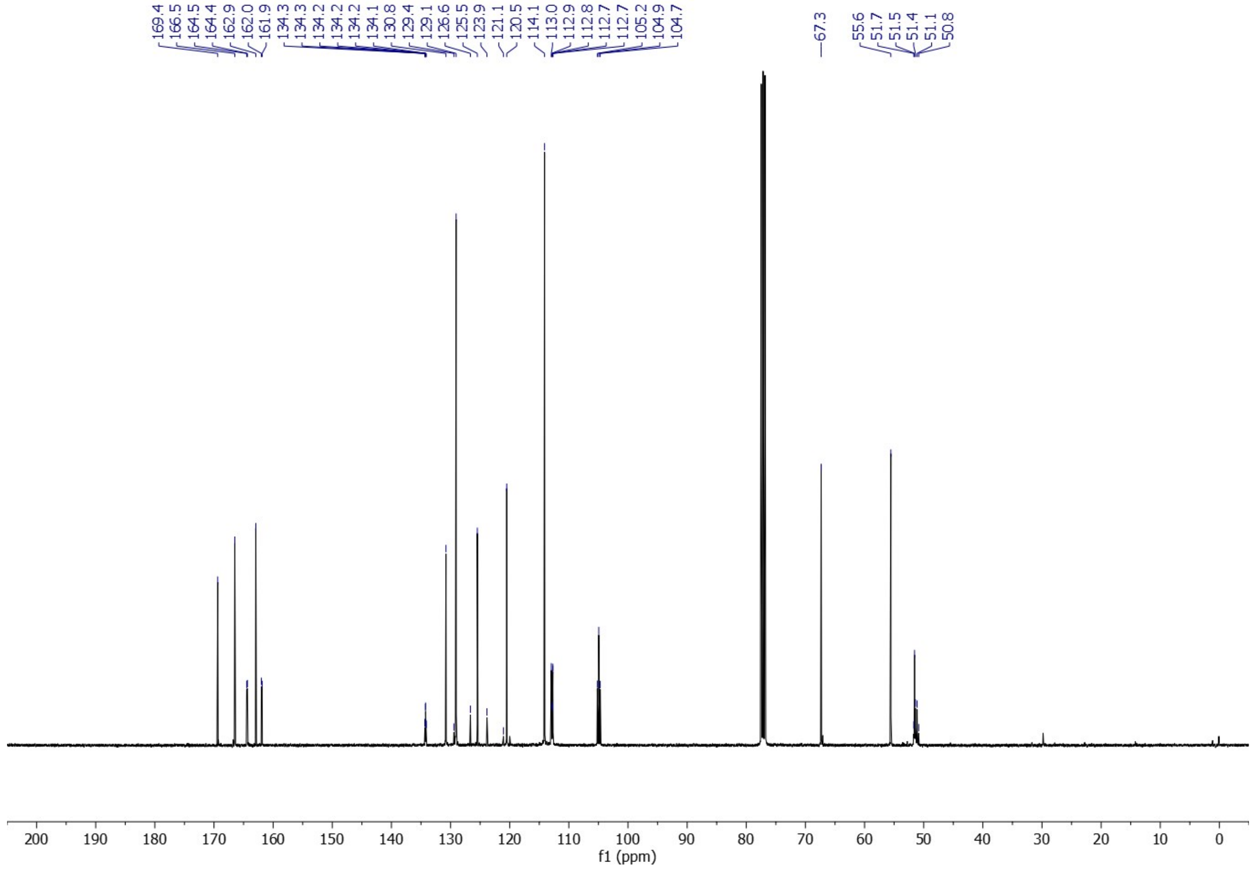
169.4 166.5 164.5 164.4 162.9 162.0 161.9 134.3 134.3 134.2 134.2 134.2 134.1 130.8 129.4 129.1 126.6 125.5 123.9 121.1 120.5 114.1 113.0 112.9 112.8 112.7 112.7 105.2 104.9 104.7
67.3
55.6 51.7 51.5 51.4 51.1 50.8
200 190 180 170 160 150 140 130 120 110 100 90 80 70 60 50 40 30 20 10 0
f1 (ppm)

**Allyl (2*S*,3*R*)-3-(3,5-difluorophenyl)-4,4,4-trifluoro-2-(4-methoxybenzamido)butanoate** *syn*-**4j**.

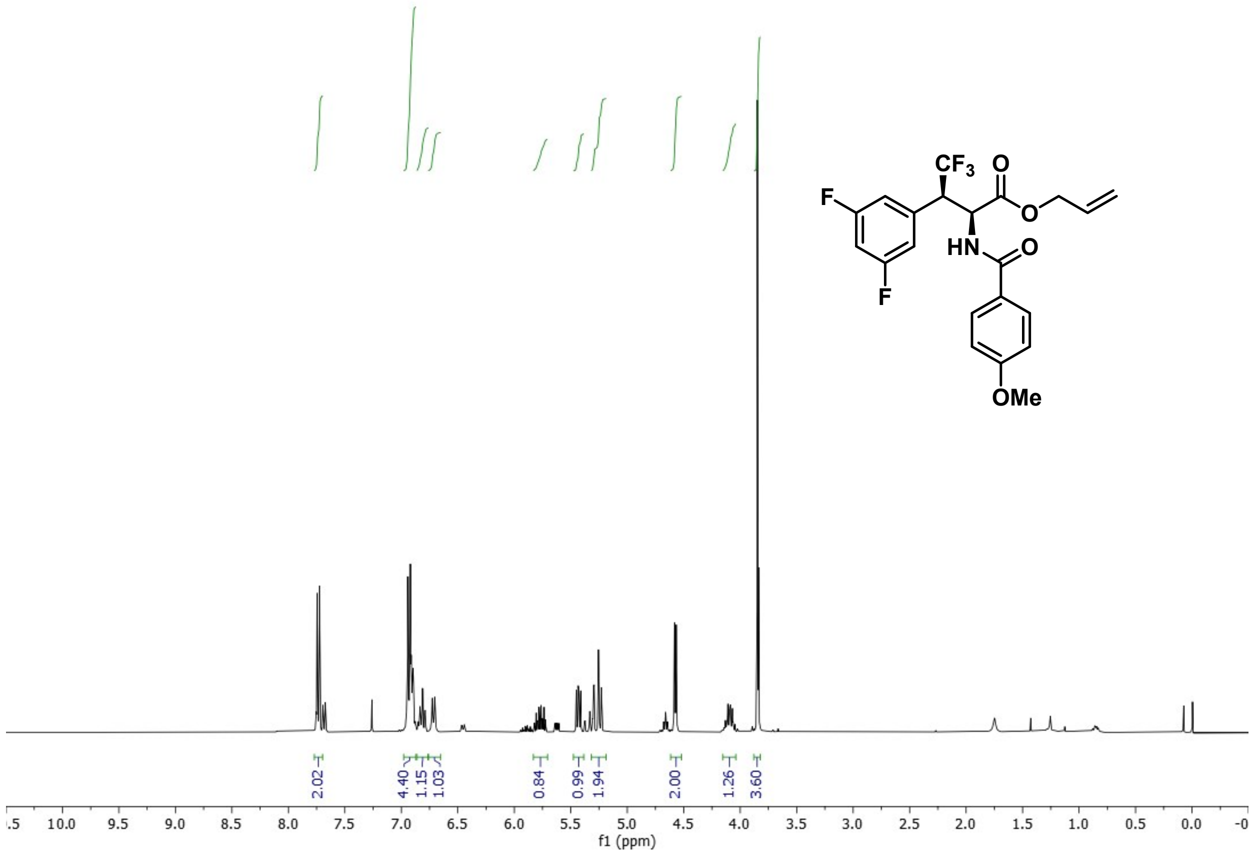

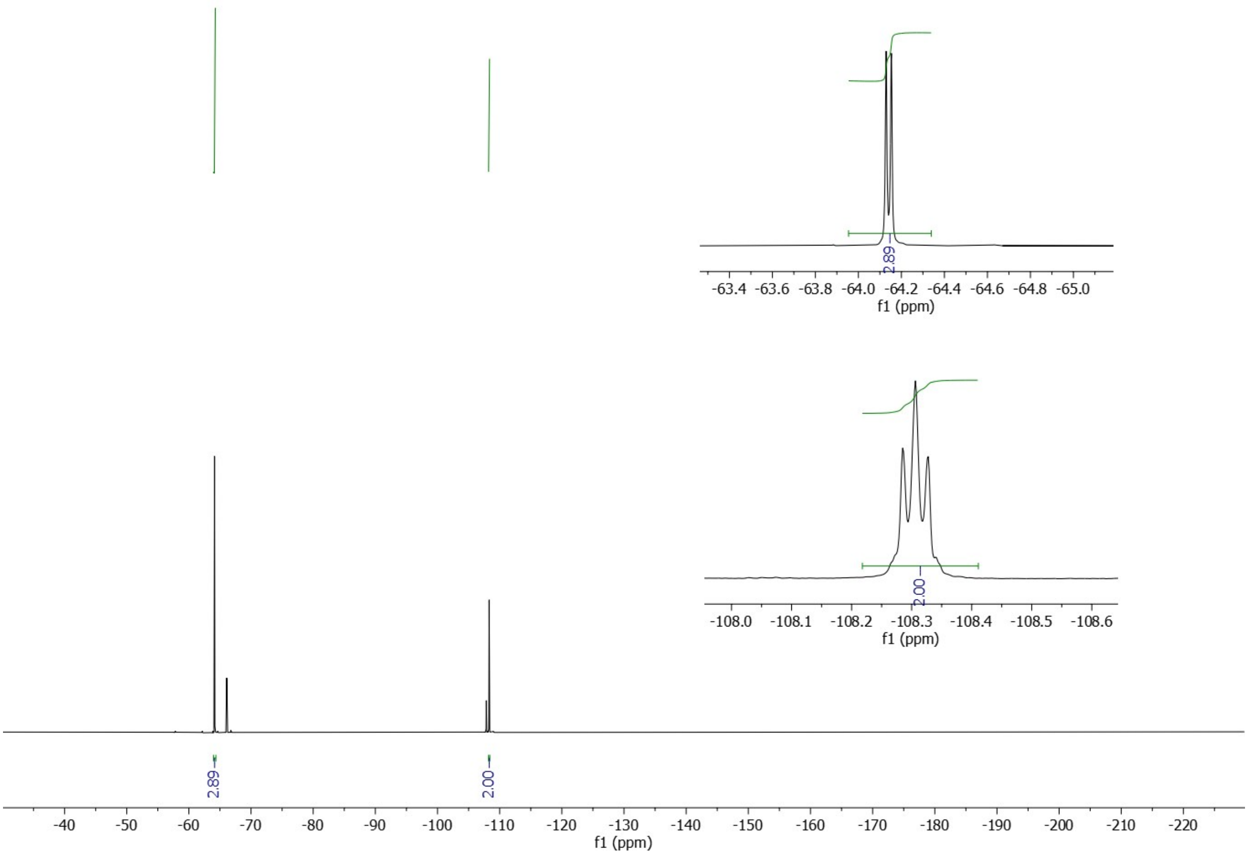
-63.4 -63.6 -63.8 -64.0 -64.2 -64.4 -64.6 -64.8 -65.0
f1 (ppm)
2.89
-108.0 -108.1 -108.2 -108.3 -108.4 -108.5 -108.6
f1 (ppm)
2.00
-40 -50 -60 -70 -80 -90 -100 -110 -120 -130 -140 -150 -160 -170 -180 -190 -200 -210 -220
f1 (ppm)

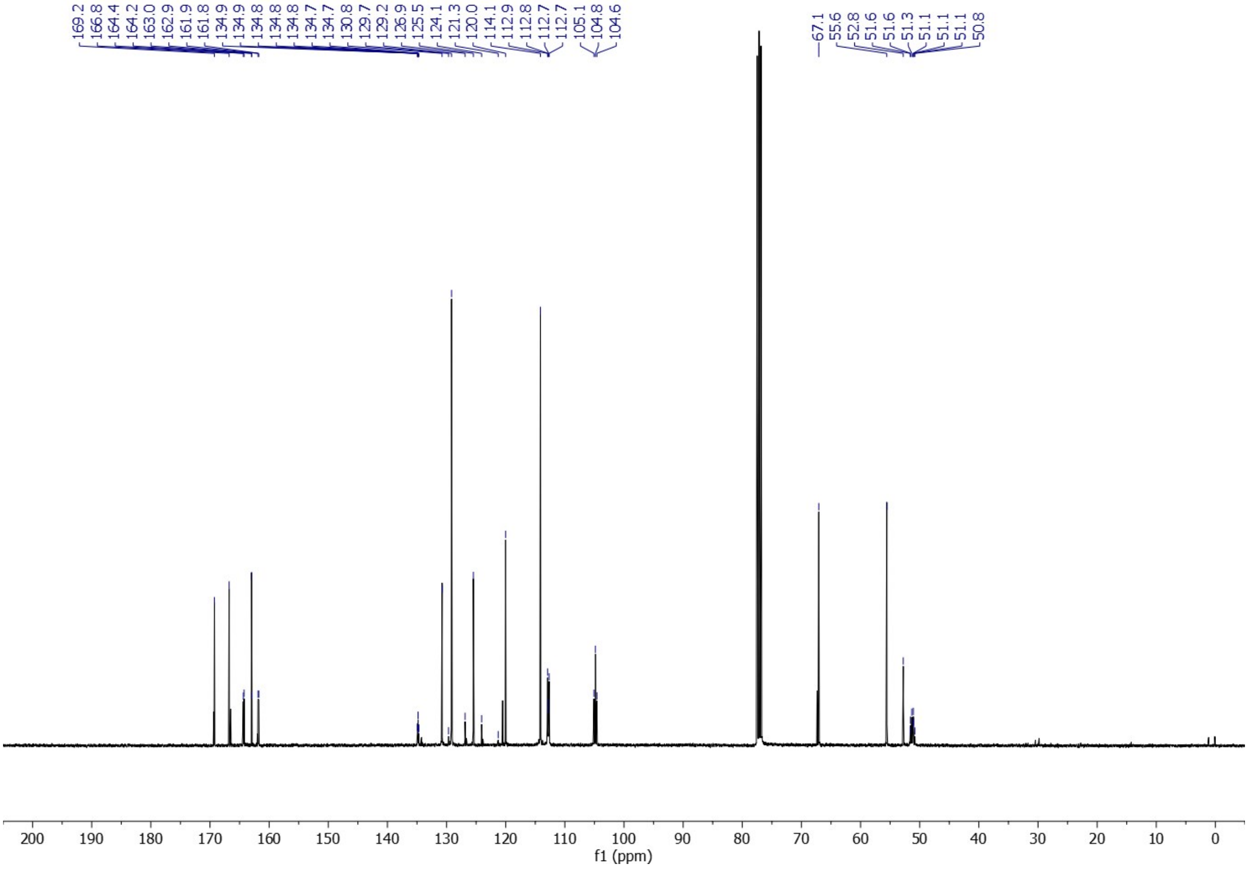
169.2 166.8 164.4 164.2 163.0 162.9 161.9 161.8 134.9 134.9 134.8 134.8 134.8 134.7 134.7 130.8 129.7 129.2 126.9 125.5 124.1 121.3 120.0 114.1 112.9 112.8 112.7 112.7 105.1 104.8 104.6
67.1 55.6 52.8 51.6 51.6 51.3 51.1 51.1 51.1 50.8
200 190 180 170 160 150 140 130 120 110 100 90 80 70 60 50 40 30 20 10 0
f1 (ppm)

**Allyl (2*R*,3*R*)-4,4,4-trifluoro-2-(4-methoxybenzamido)-3-(thiophen-2-yl)butanoate *anti*-4k**.

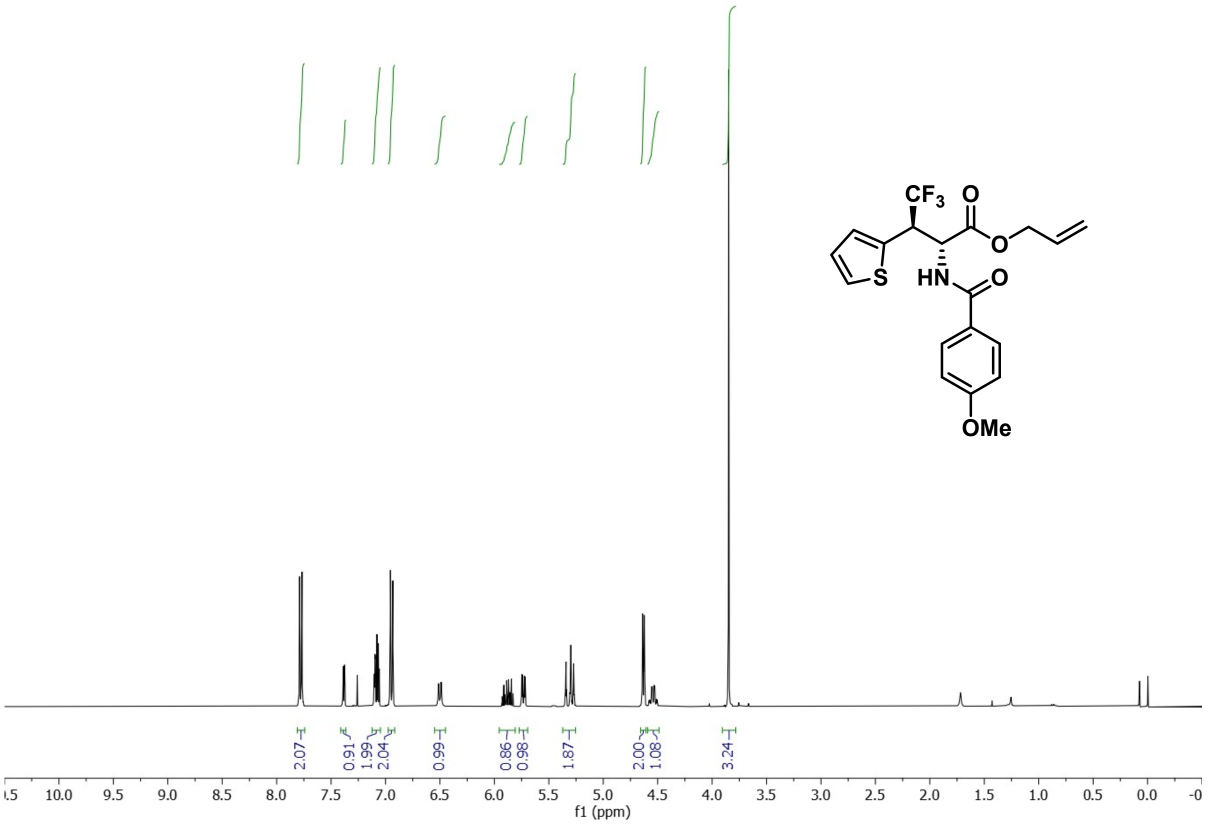

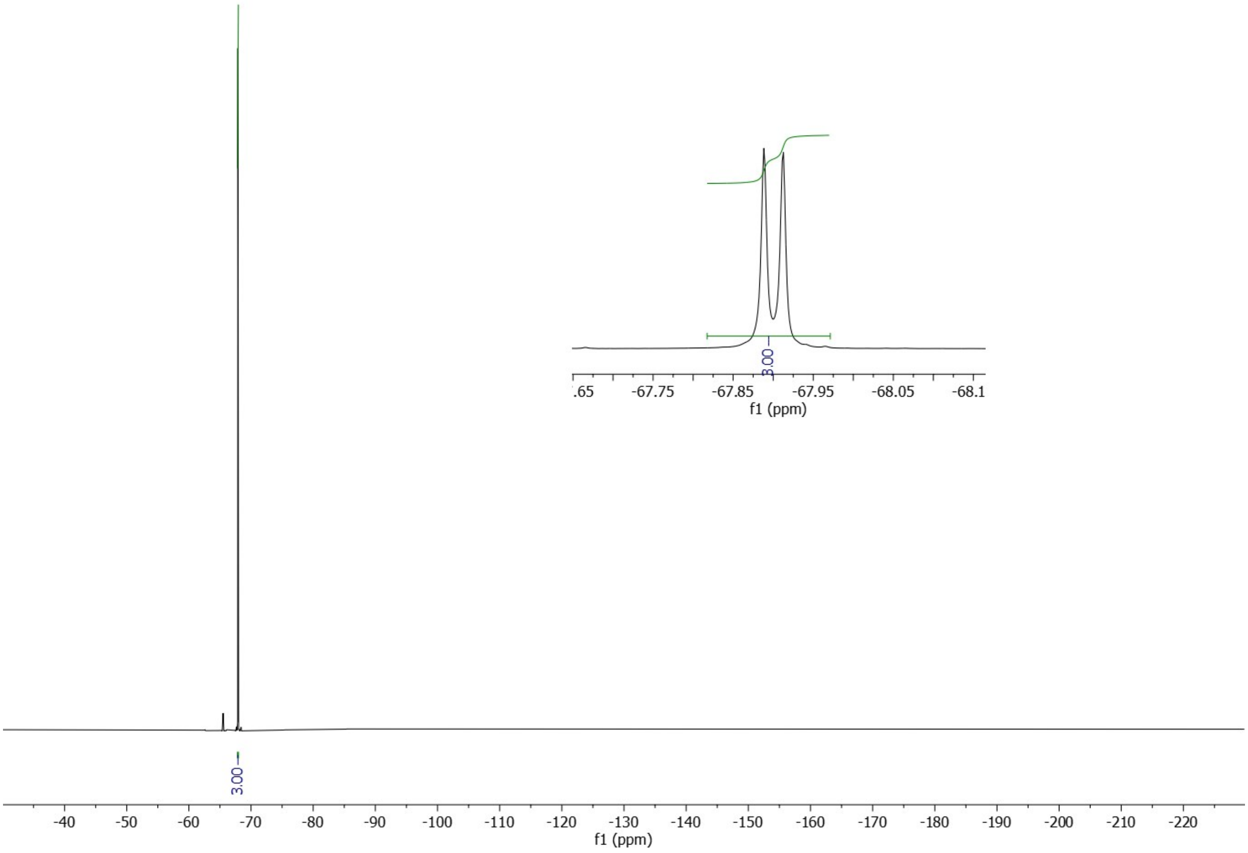

3.00
.65
-67.75
-67.85
-67.95
-68.05
-68.1
f1 (ppm)
3.00
-40 -50 -60 -70 -80 -90 -100 -110 -120 -130 -140 -150 -160 -170 -180 -190 -200 -210 -220
f1 (ppm)


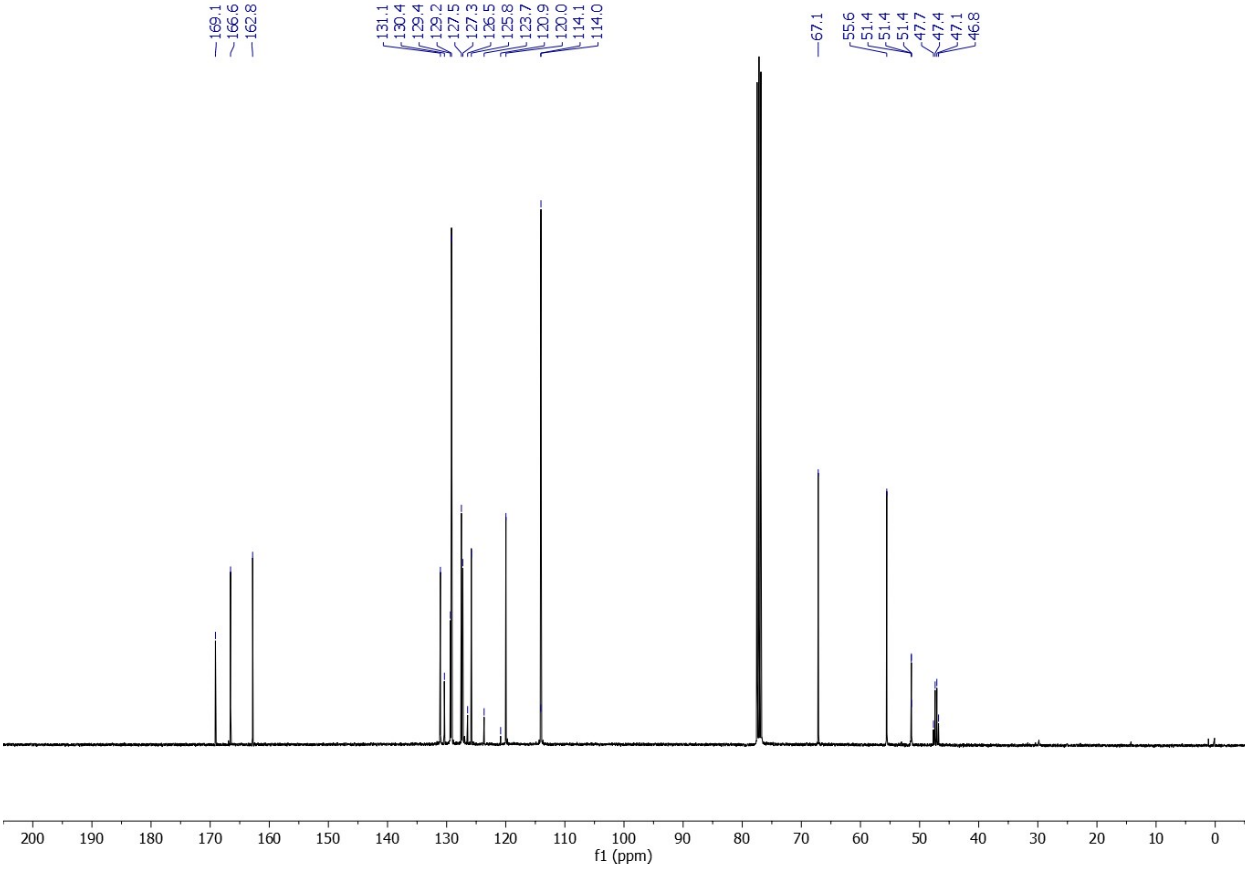

169.1
166.6
162.8
131.1
130.4
129.4
129.2
127.5
127.3
126.5
125.8
123.7
120.9
120.0
114.1
114.0
67.1
55.6
51.4
51.4
51.4
47.7
47.4
47.1
46.8
200 190 180 170 160 150 140 130 120 110 100 90 80 70 60 50 40 30 20 10 0
f1 (ppm)

**Allyl (2*S*,3*R*)-4,4,4-trifluoro-2-(4-methoxybenzamido)-3-(thiophen-2-yl)butanoate *syn*-4k.**

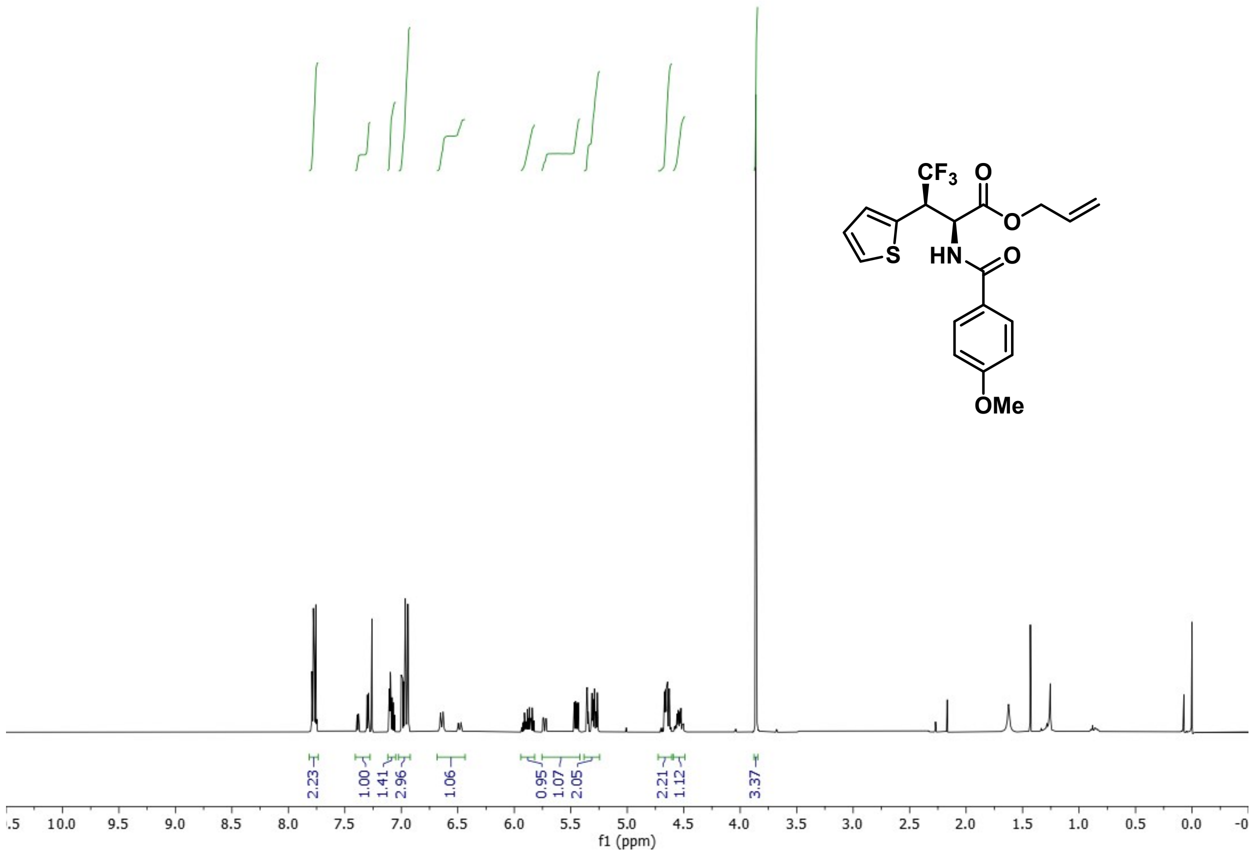

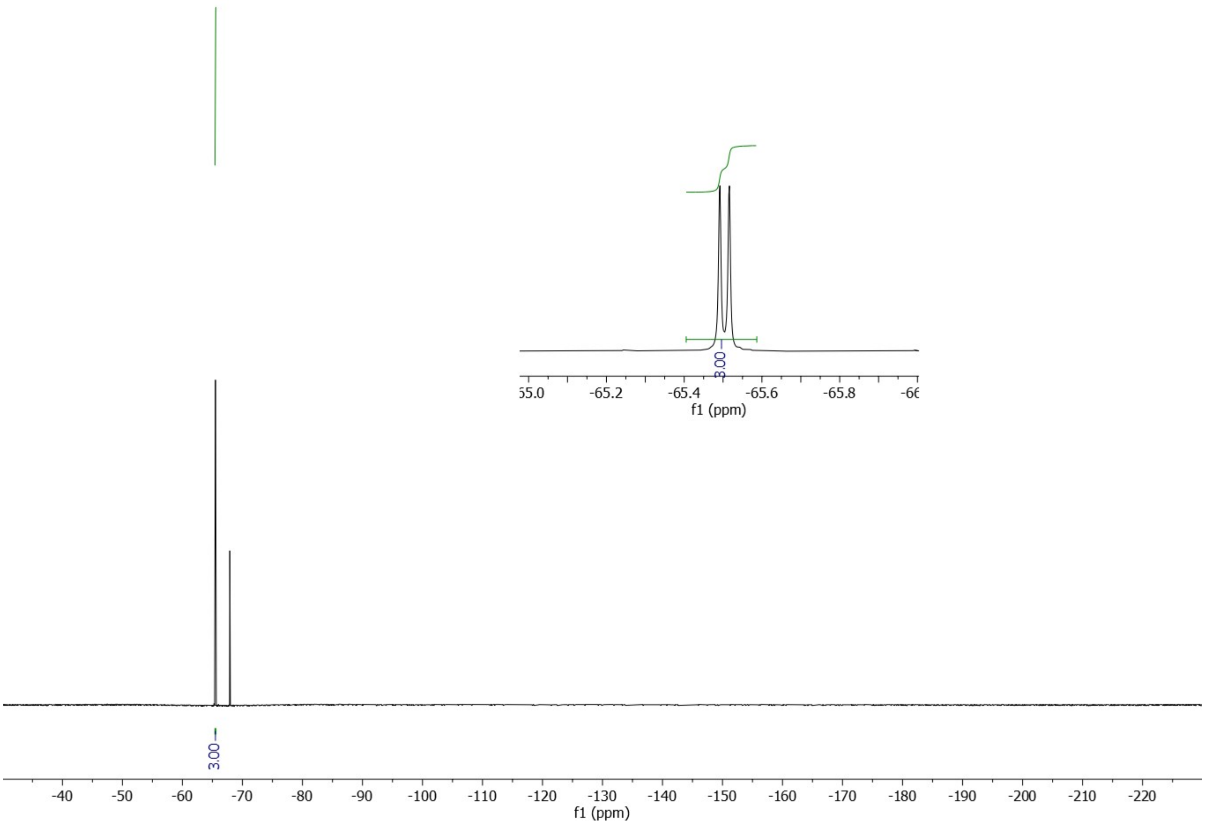
3.00
-65.0
-65.2
-65.4
-65.6
-65.8
-66
f1 (ppm)
3.00
-40
-50
-60
-70
-80
-90
-100
-110
-120
-130
-140
-150
-160
-170
-180
-190
-200
-210
-220
f1 (ppm)

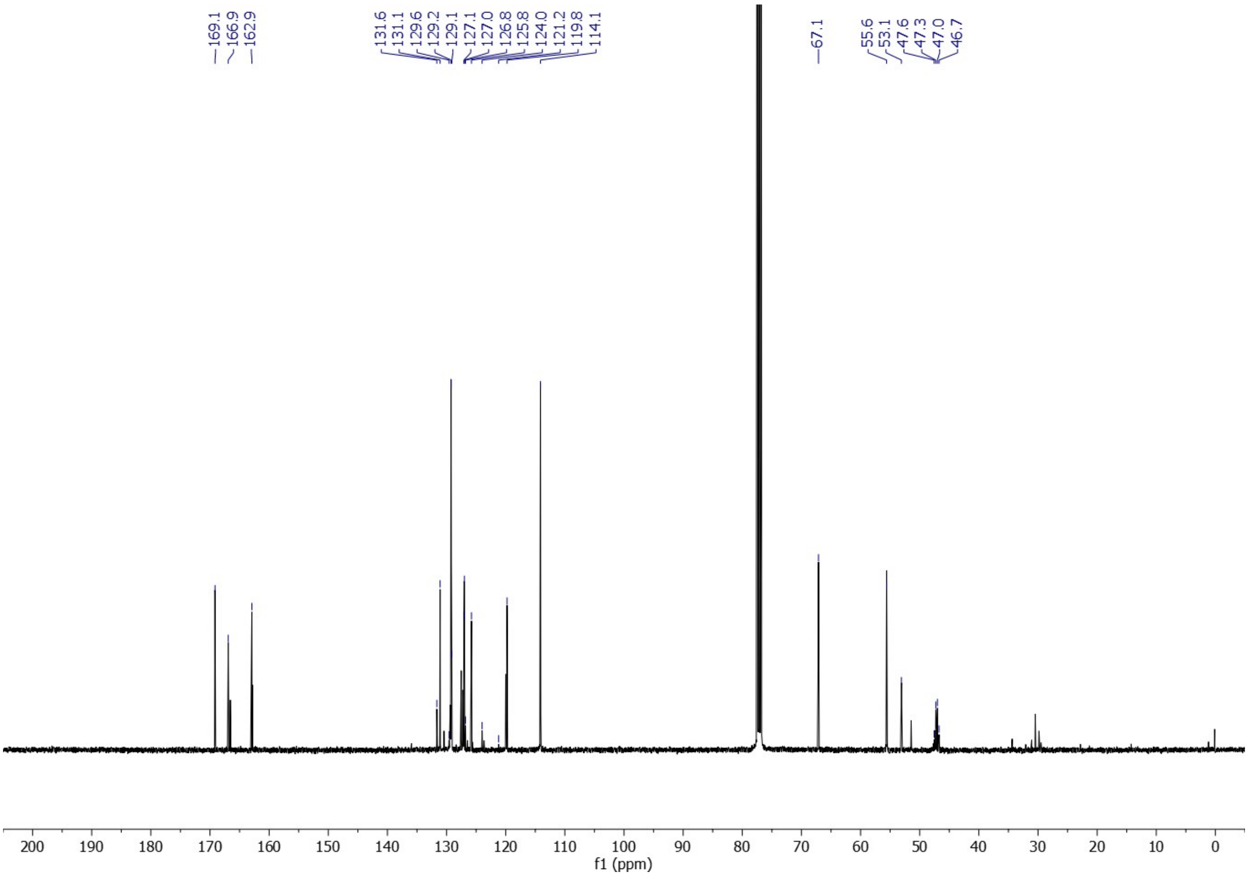
169.1
166.9
162.9
131.6
131.1
129.6
129.2
129.1
127.1
127.0
126.8
125.8
124.0
121.2
119.8
114.1
67.1
55.6
53.1
47.6
47.3
47.0
46.7
200
190
180
170
160
150
140
130
120
110
100
90
80
70
60
50
40
30
20
10
0
f1 (ppm)

**Allyl (2*R*,3*R*)-4,4,4-trifluoro-2-(4-methoxybenzamido)-3-(1-tosyl-1H-indol-3-yl)butanoate *anti*-4l**.

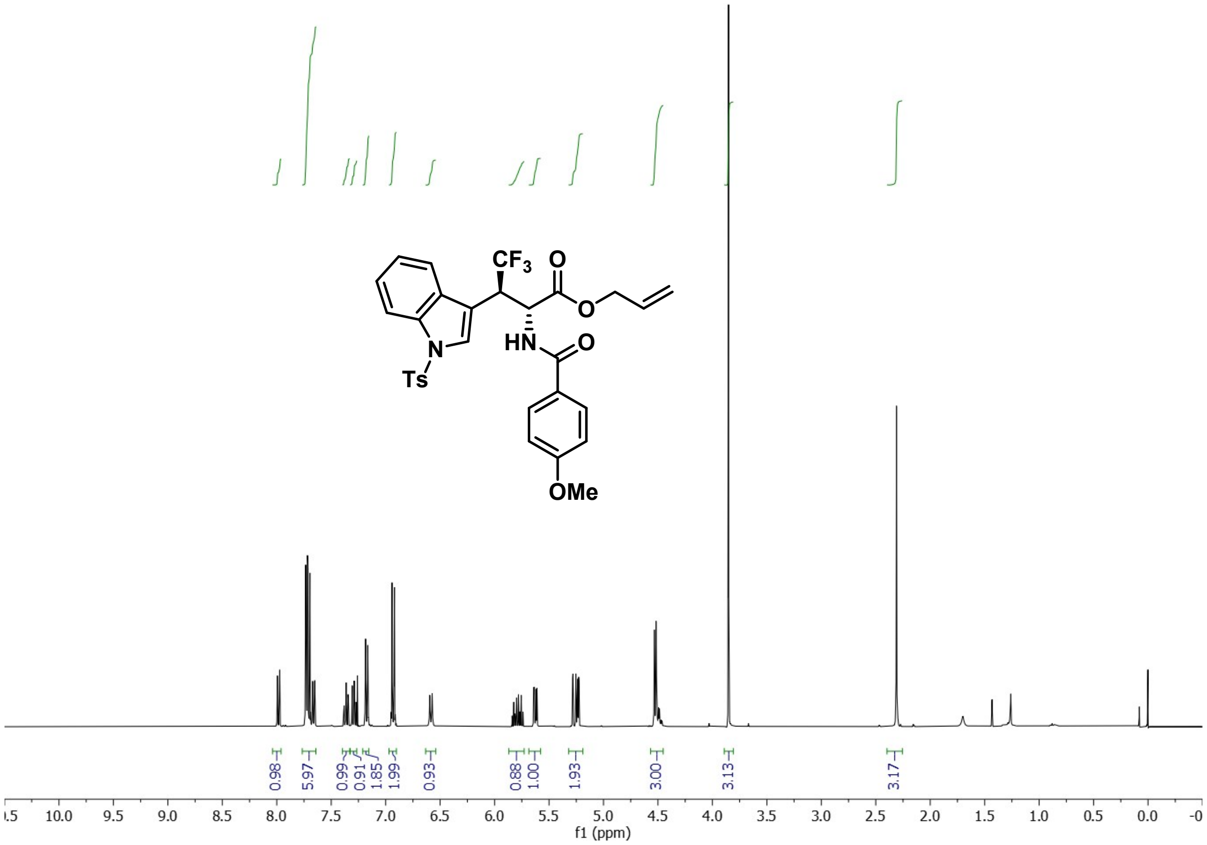

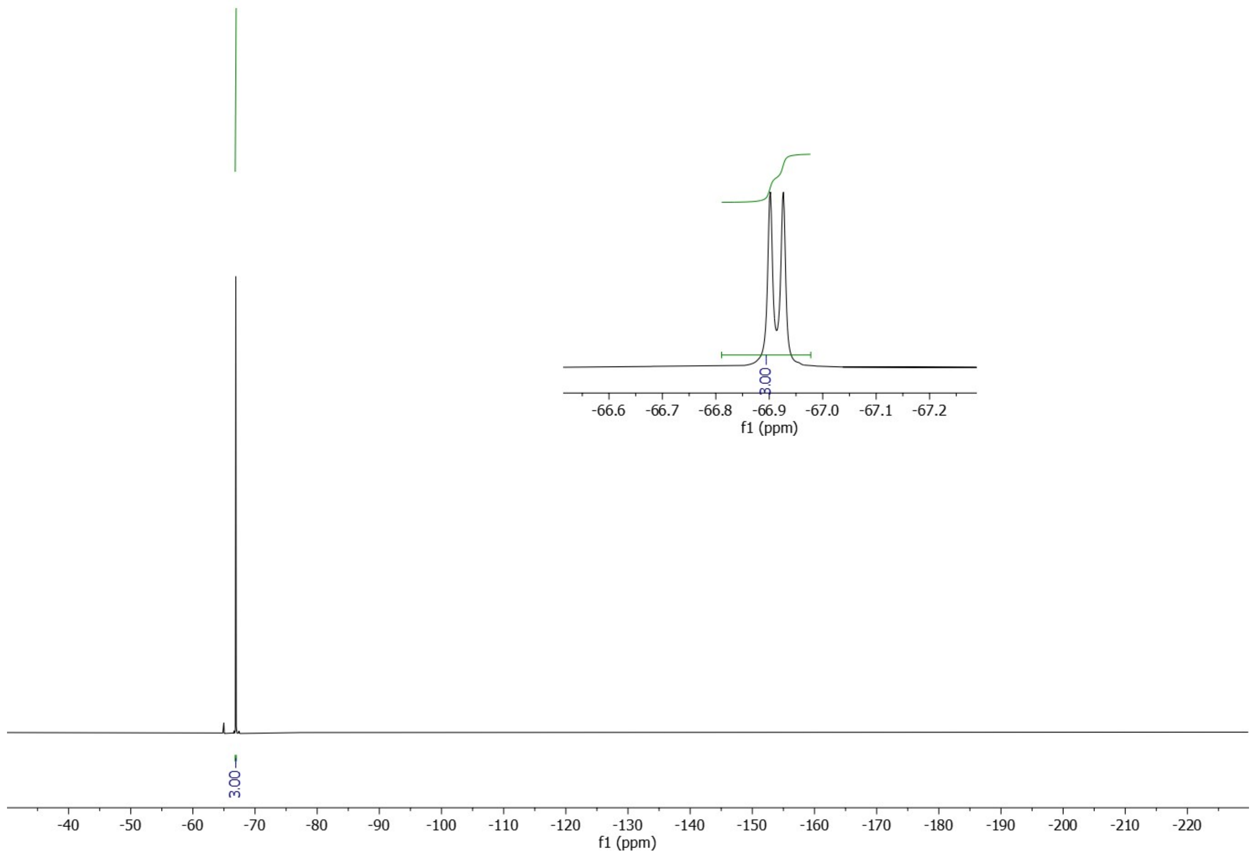
3.00
-66.6
-66.7
-66.8
-66.9
-67.0
-67.1
-67.2
f1 (ppm)
3.00
-40
-50
-60
-70
-80
-90
-100
-110
-120
-130
-140
-150
-160
-170
-180
-190
-200
-210
-220
f1 (ppm)

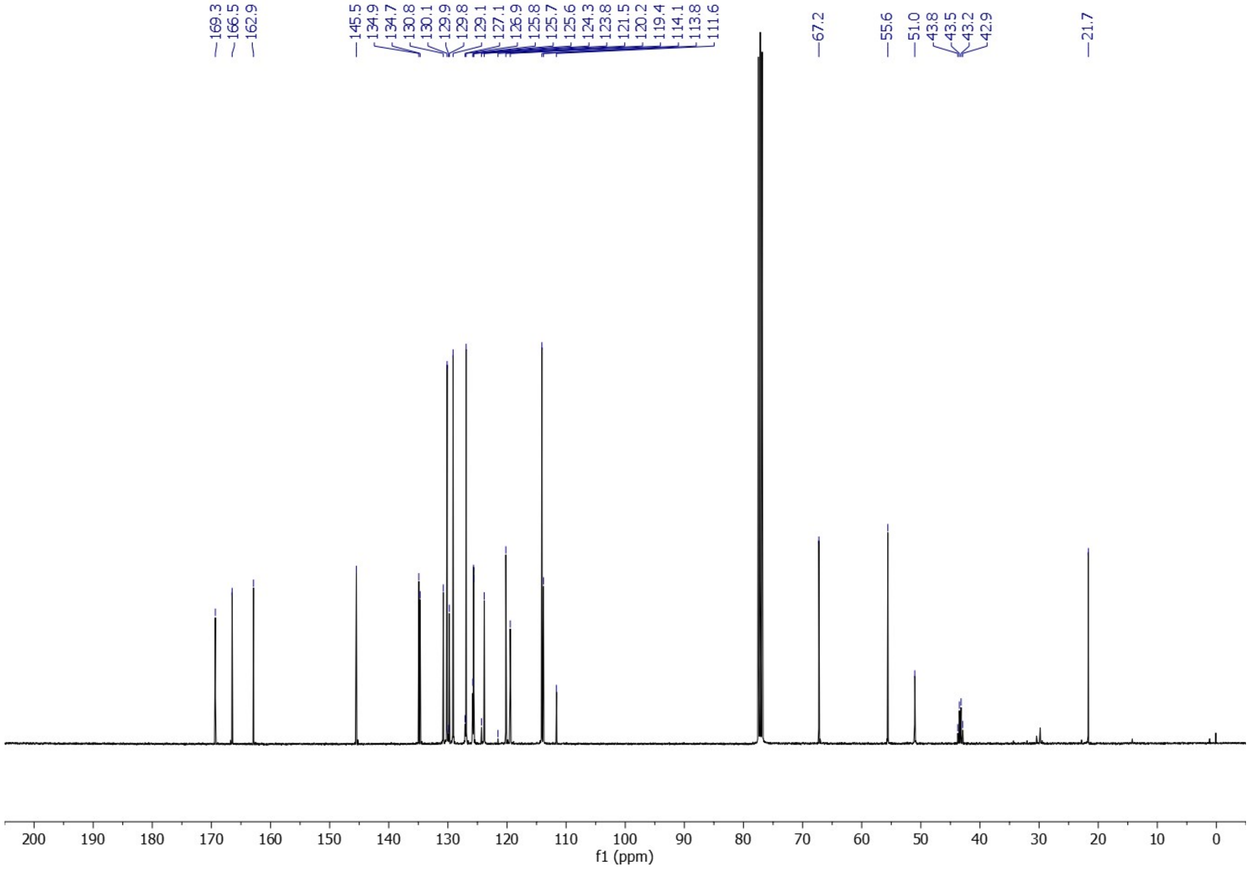
169.3
166.5
162.9
145.5
134.9
134.7
130.8
130.1
129.9
129.8
129.1
127.1
126.9
125.8
125.7
125.6
124.3
123.8
121.5
120.2
119.4
114.1
113.8
111.6
67.2
55.6
51.0
43.8
43.5
43.2
42.9
21.7
f1 (ppm)

**Allyl (2*S*,3*R*)-4,4,4-trifluoro-2-(4-methoxybenzamido)-3-(1-tosyl-1H-indol-3-yl)butanoate *syn*-4l.**

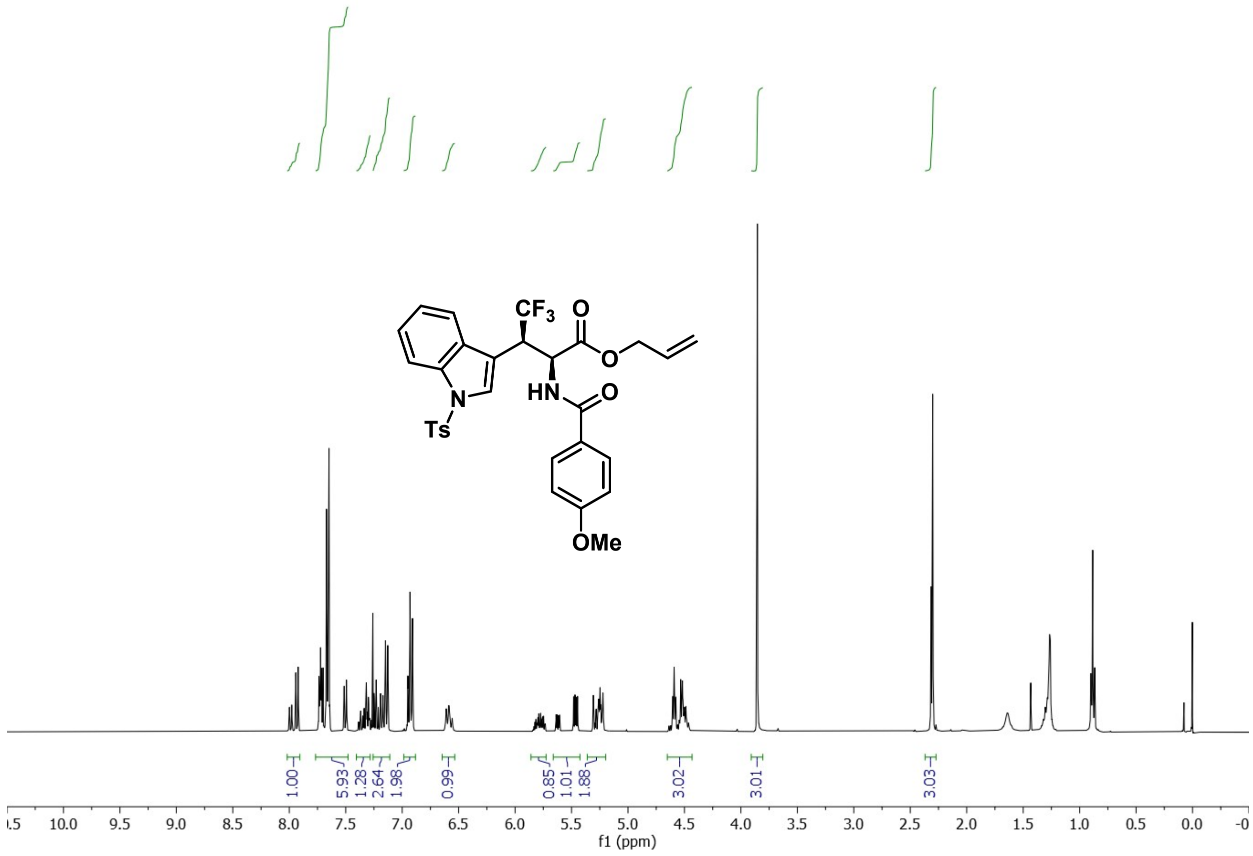

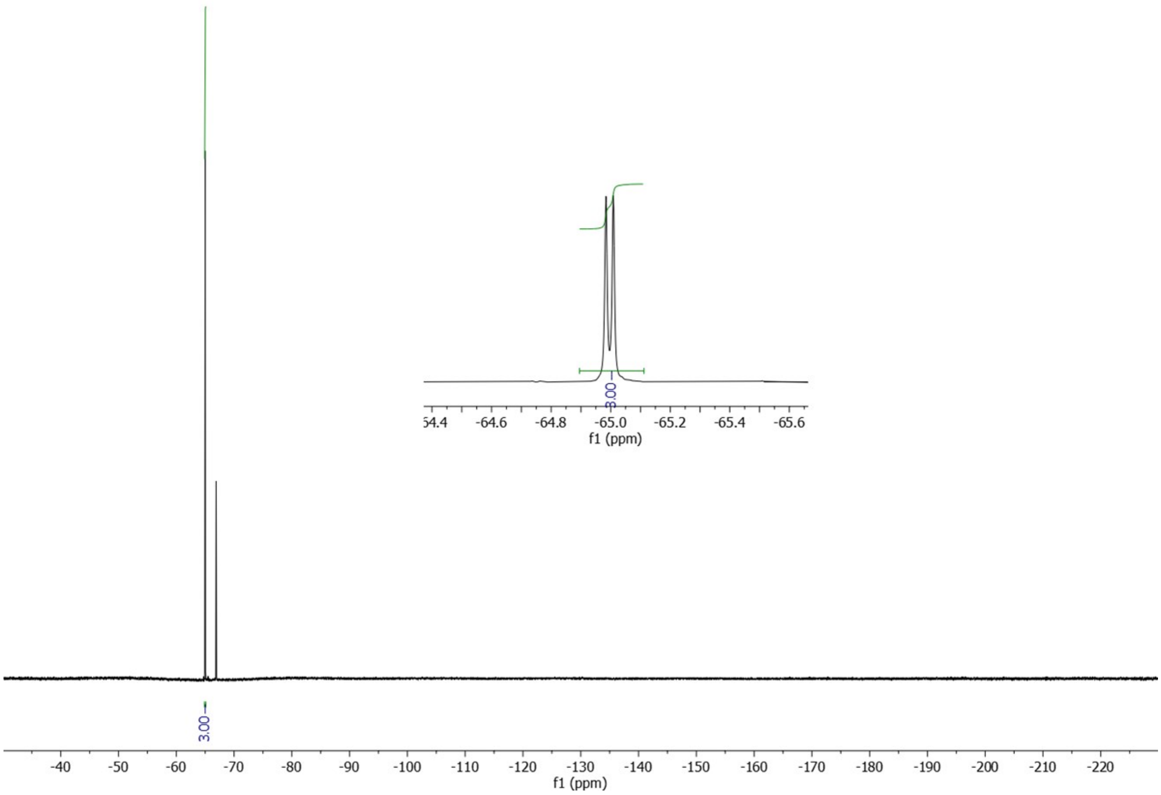

3.00
-64.6
-64.8
-65.0
-65.2
-65.4
-65.6
f1 (ppm)
-40
-50
-60
-70
-80
-90
-100
-110
-120
-130
-140
-150
-160
-170
-180
-190
-200
-210
-220


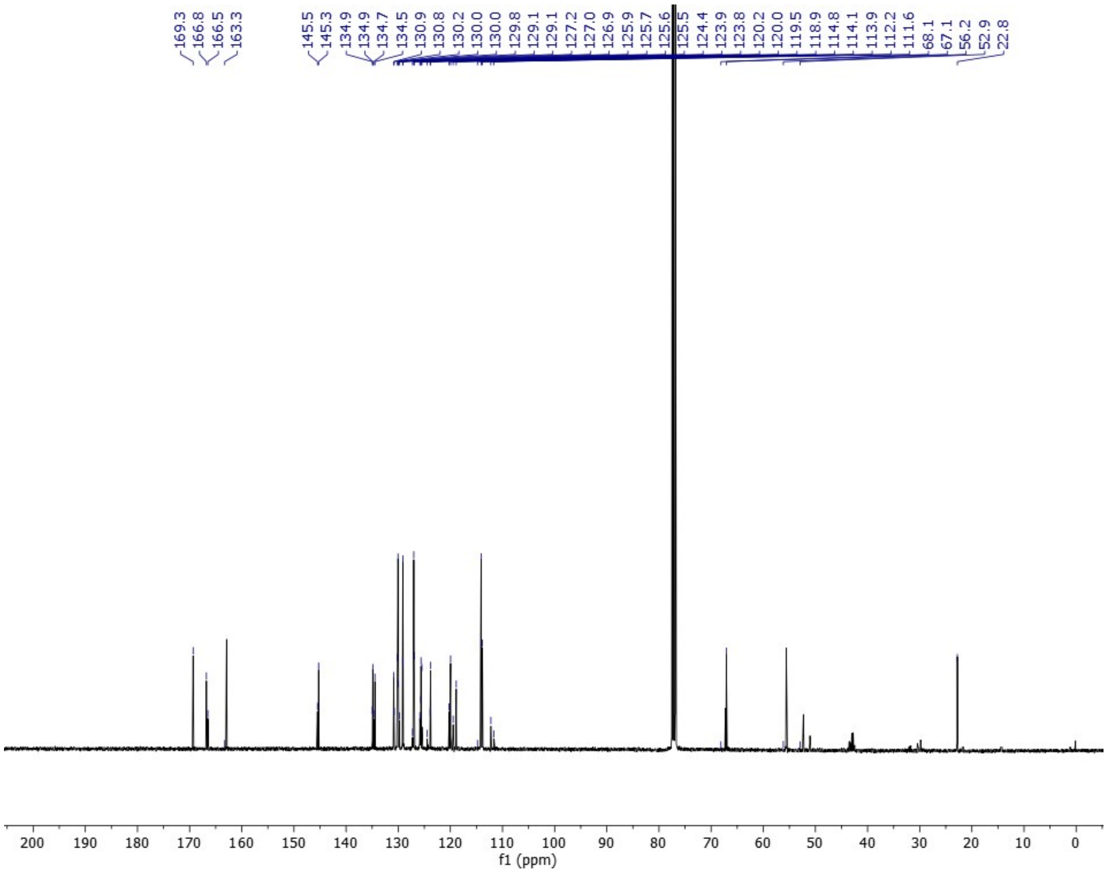

169.3
166.8
166.5
163.3
145.5
145.3
134.9
134.9
134.7
134.5
130.9
130.8
130.2
130.0
130.0
129.8
129.1
129.1
127.2
127.0
126.9
125.9
125.7
125.6
125.5
124.4
123.9
123.8
120.2
120.0
119.5
118.9
114.8
114.1
113.9
112.2
111.6
68.1
67.1
56.2
52.9
22.8
200
190
180
170
160
150
140
130
120
110
100
90
80
70
60
50
40
30
20
10
0
f1 (ppm)

**Allyl (2*R*,3*R*)-4,4,5,5,6,6,6-heptafluoro-2-(4-methoxybenzamido)-3-phenylhexanoate** *anti*-**4m**.

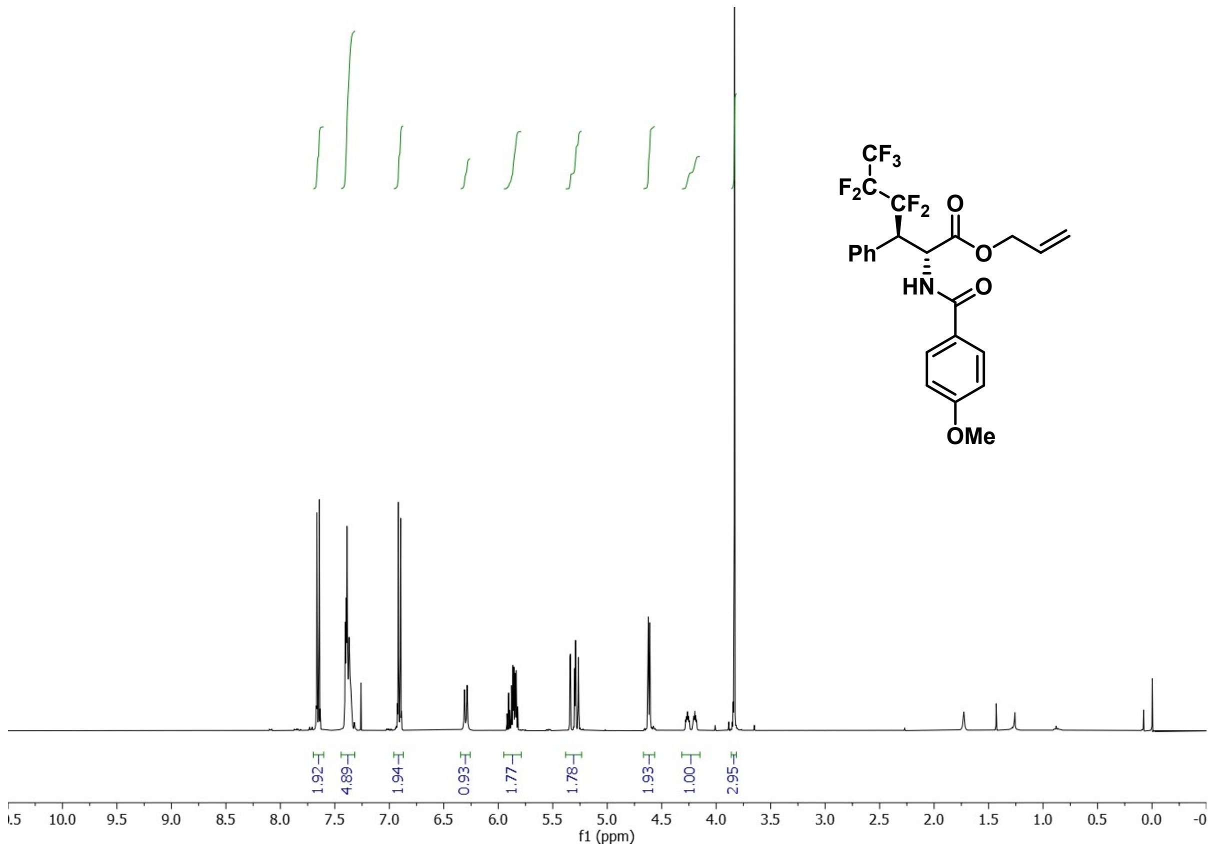

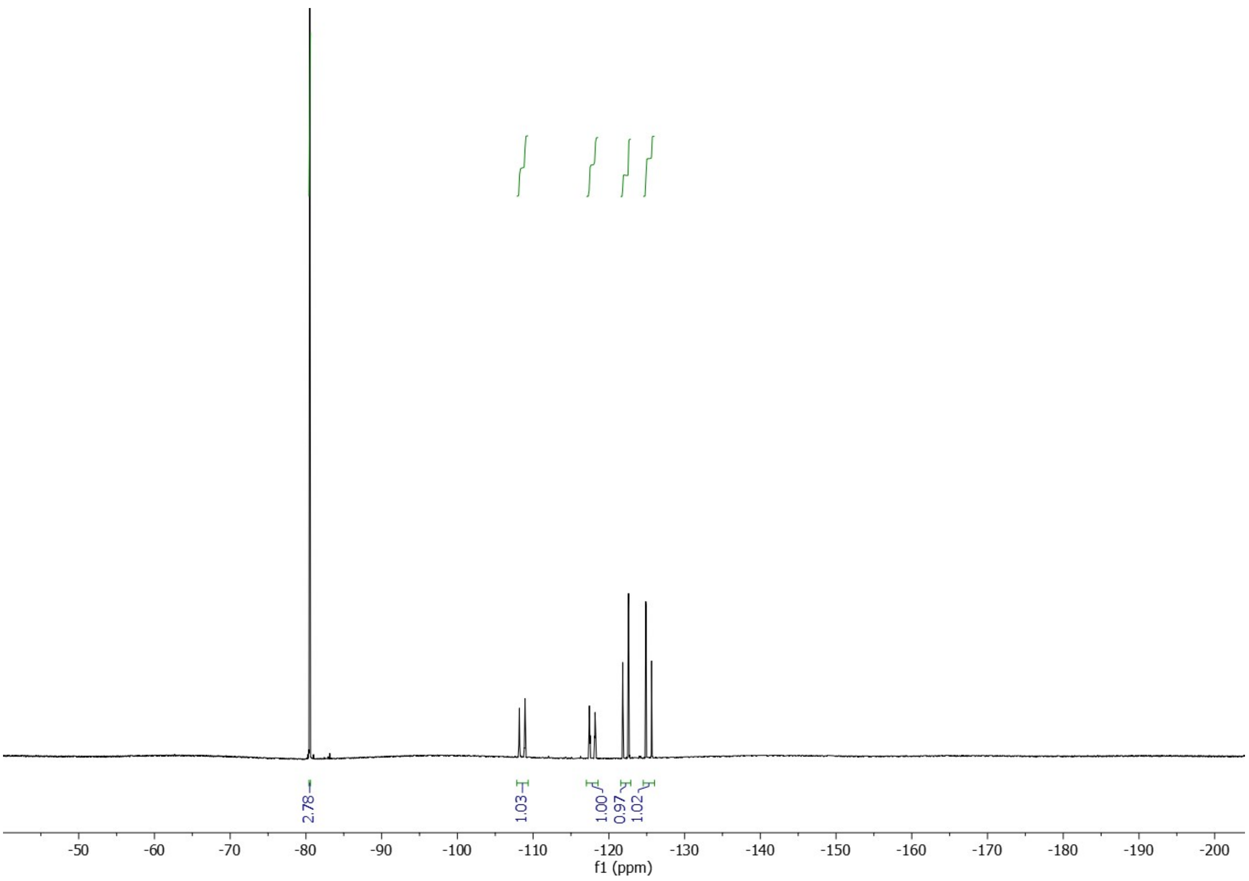
2.78
1.03
1.00
0.97
1.02
-50
-60
-70
-80
-90
-100
-110
-120
-130
-140
-150
-160
-170
-180
-190
-200
f1 (ppm)

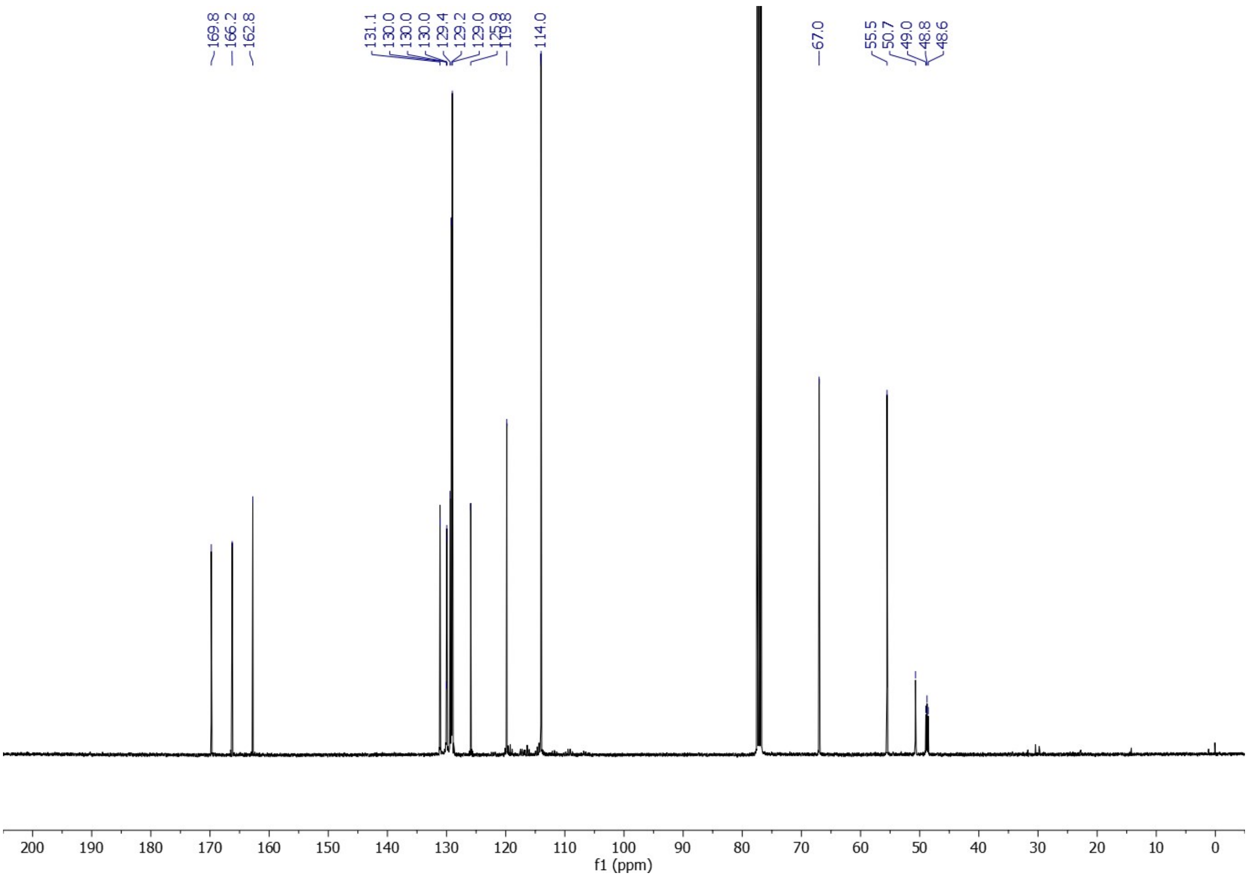
169.8
166.2
162.8
131.1
130.0
130.0
130.0
129.4
129.2
129.0
125.9
119.8
114.0
67.0
55.5
50.7
49.0
48.8
48.6
200
190
180
170
160
150
140
130
120
110
100
90
80
70
60
50
40
30
20
10
0
f1 (ppm)

**Allyl (2*S*,3*R*)-4,4,5,5,6,6,6-heptafluoro-2-(4-methoxybenzamido)-3-phenylhexanoate** *syn*-**4m**.

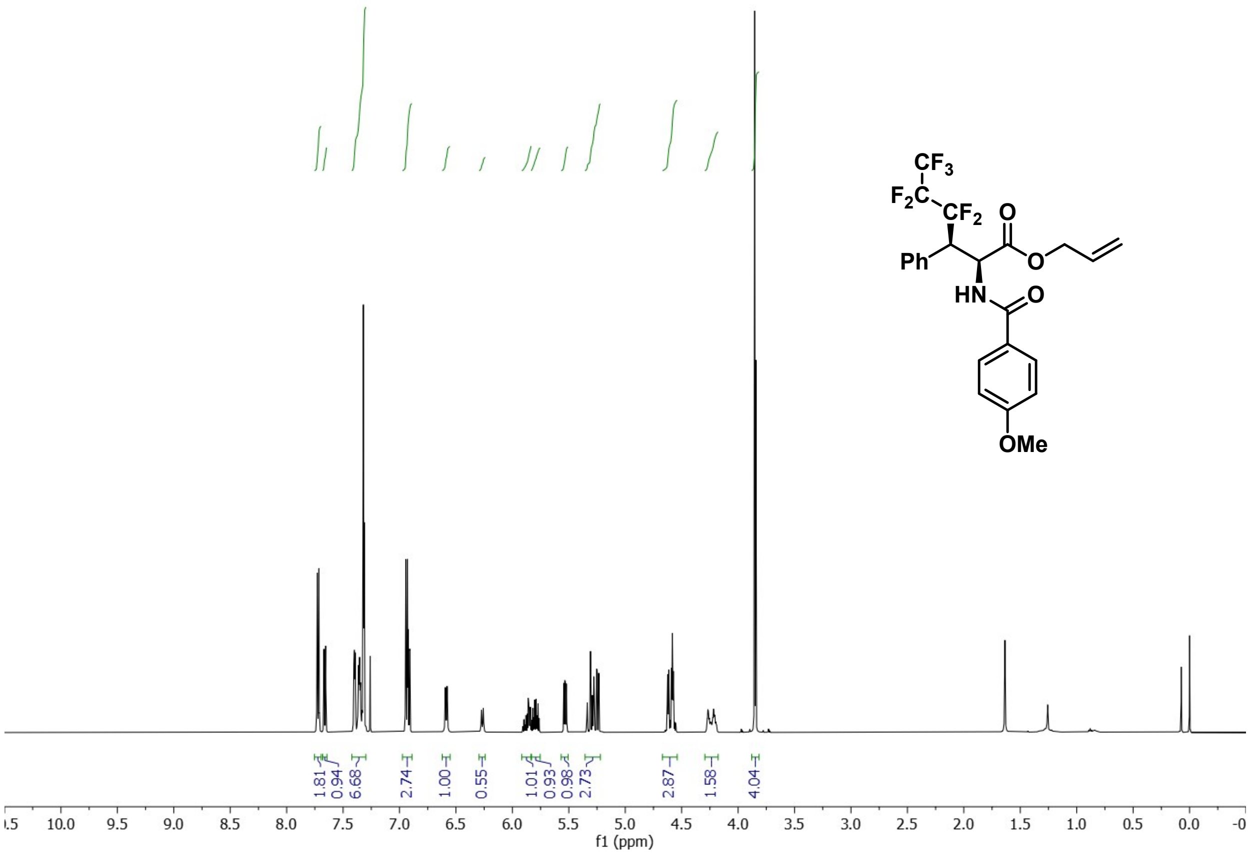

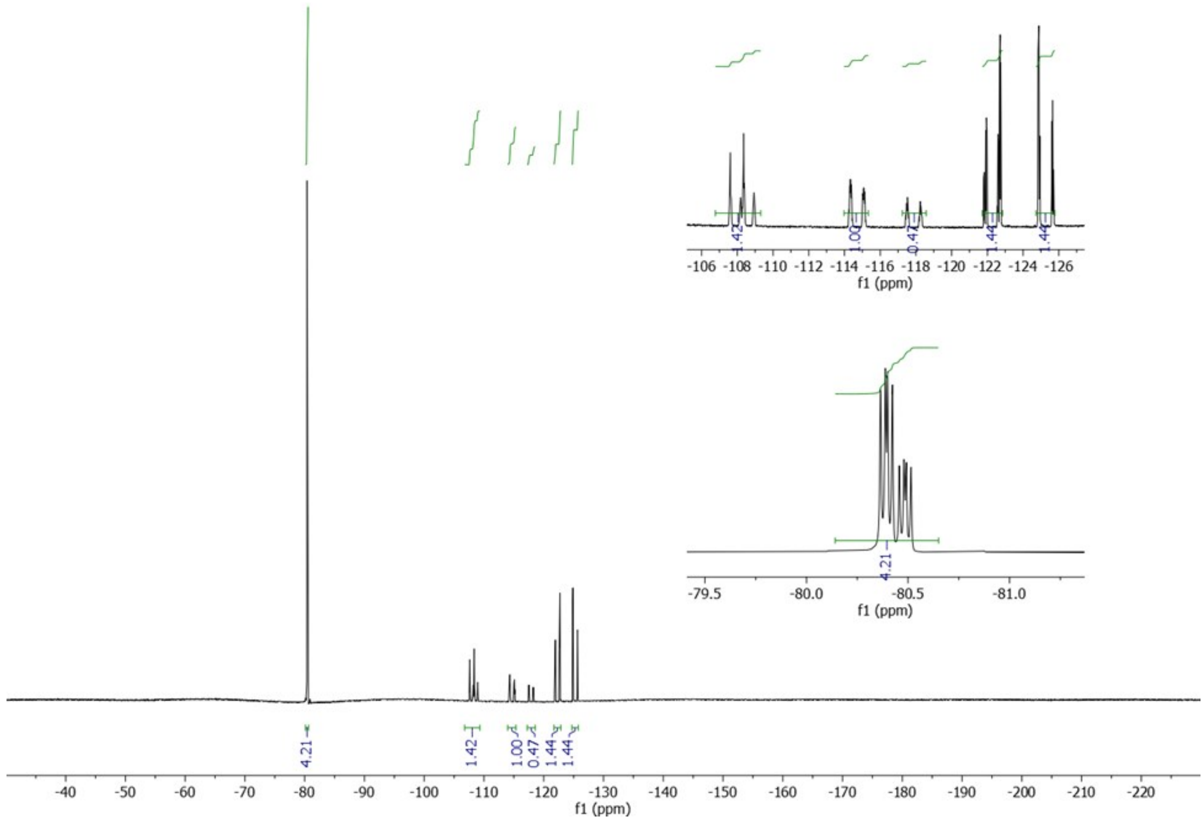
4.21
1.42
1.00
0.47
1.44
1.44
-40 -50 -60 -70 -80 -90 -100 -110 -120 -130 -140 -150 -160 -170 -180 -190 -200 -210 -220
f1 (ppm)
-106 -108 -110 -112 -114 -116 -118 -120 -122 -124 -126
-79.5 -80.0 -80.5 -81.0

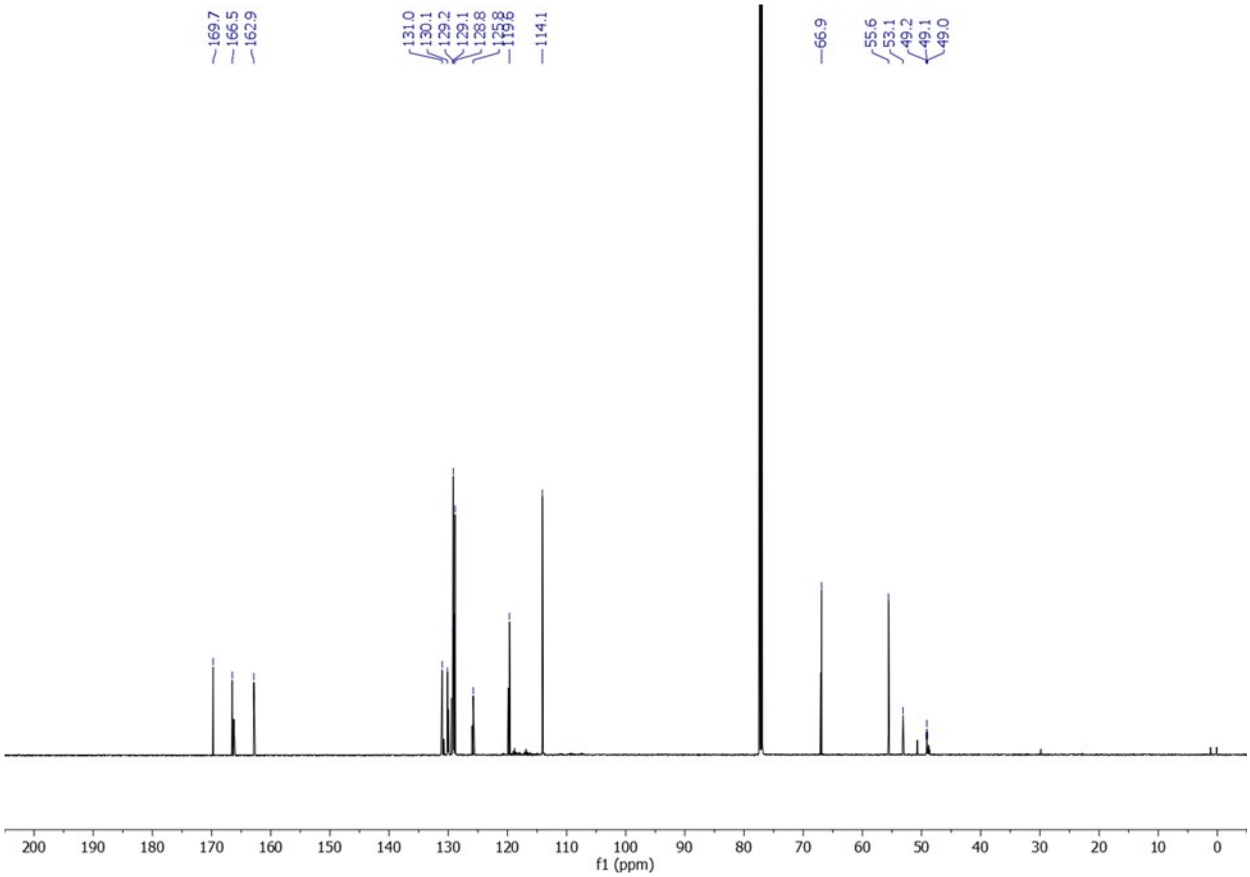
169.7
166.5
162.9
131.0
130.1
129.2
129.1
128.8
125.8
119.6
114.1
66.9
55.6
53.1
49.2
49.1
49.0
200 190 180 170 160 150 140 130 120 110 100 90 80 70 60 50 40 30 20 10 0
f1 (ppm)

**Allyl (2*R*,3*R*)-3-cyclohexyl-4,4,4-trifluoro-2-(4-methoxybenzamido)butanoate *anti*-4n**.

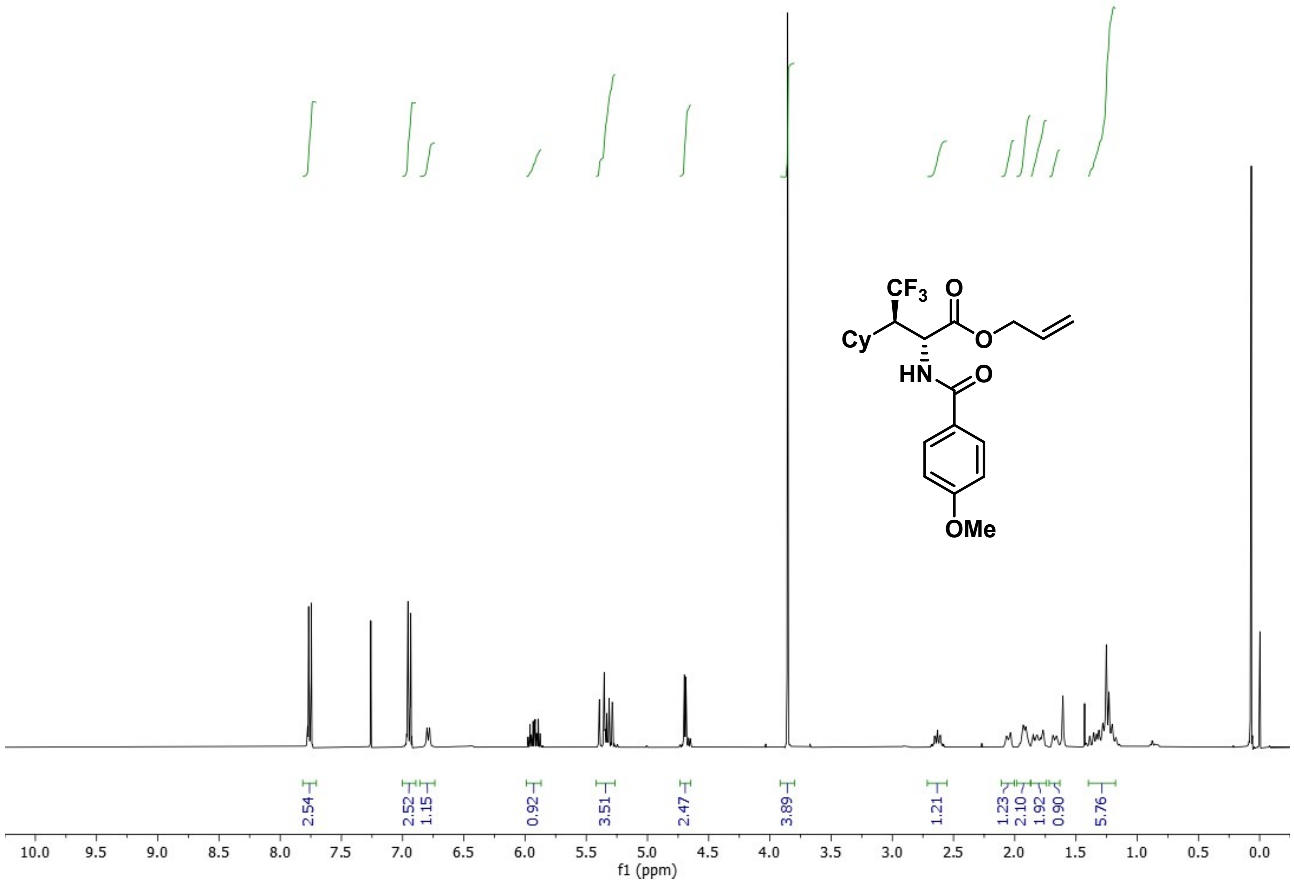

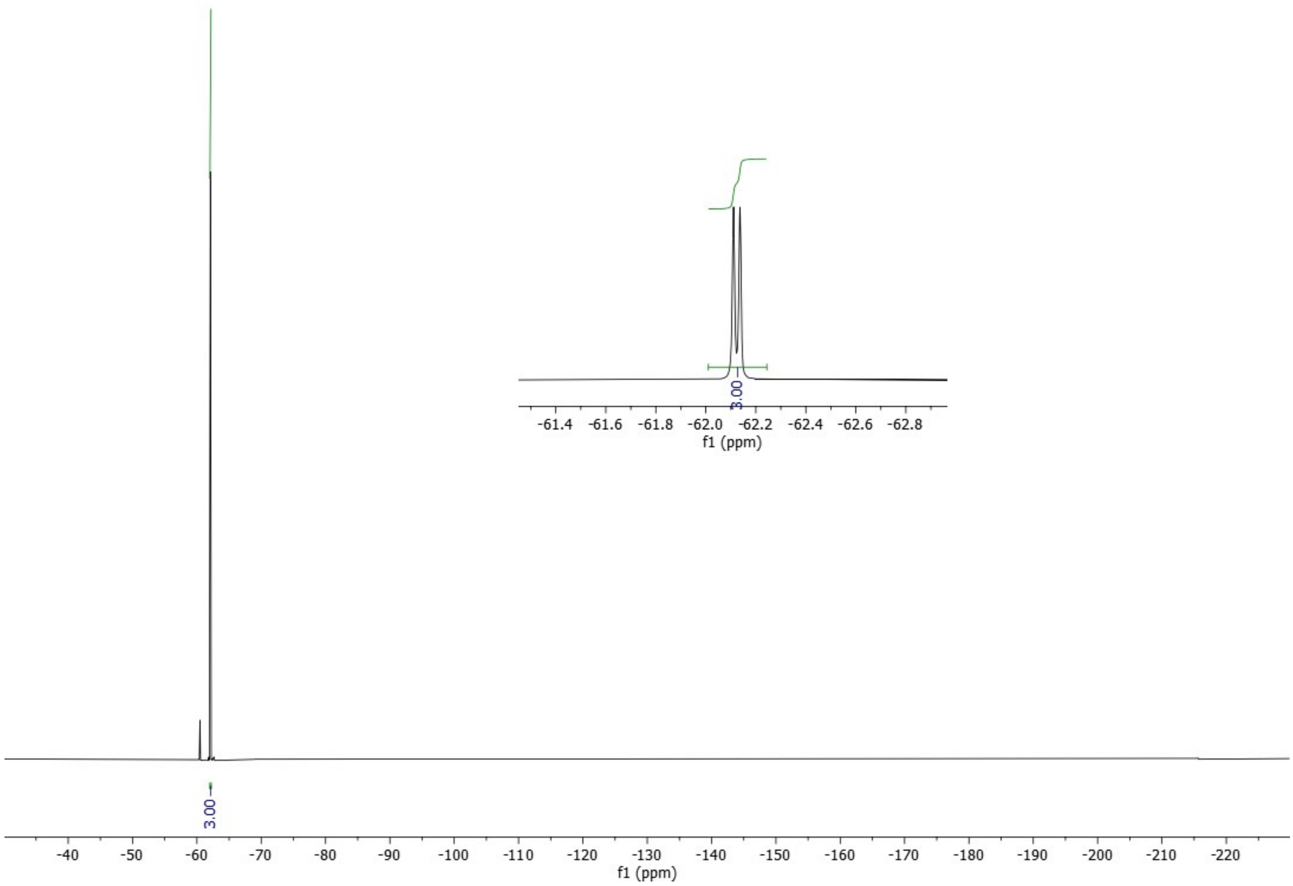
3.00
-61.4
-61.6
-61.8
-62.0
-62.2
-62.4
-62.6
-62.8
f1 (ppm)
3.00
-40
-50
-60
-70
-80
-90
-100
-110
-120
-130
-140
-150
-160
-170
-180
-190
-200
-210
-220
f1 (ppm)

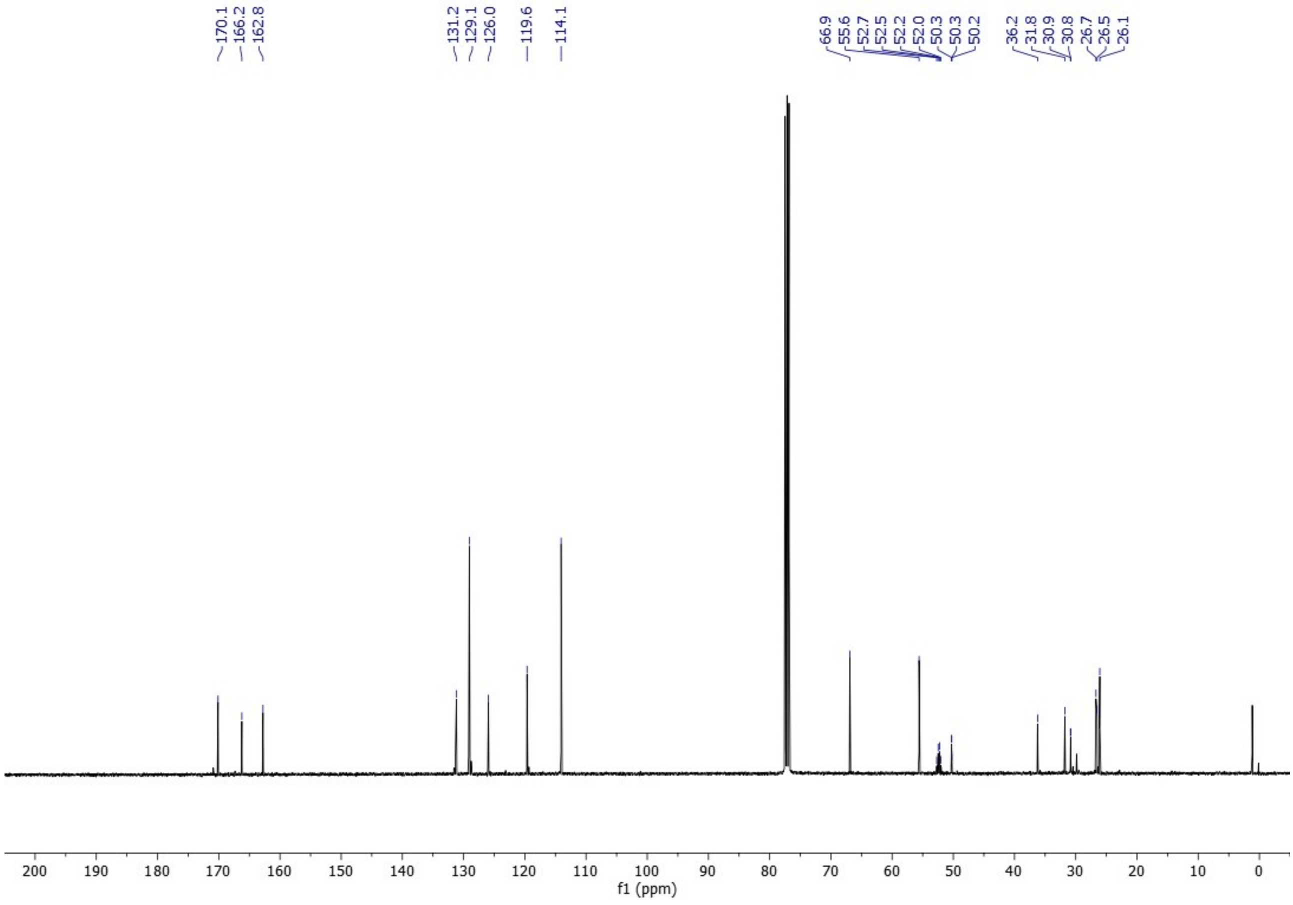
170.1
166.2
162.8
131.2
129.1
126.0
119.6
114.1
66.9
55.6
52.7
52.5
52.2
52.0
50.3
50.3
50.2
36.2
31.8
30.9
30.8
26.7
26.5
26.1
200
190
180
170
160
150
140
130
120
110
100
90
80
70
60
50
40
30
20
10
0
f1 (ppm)

**Allyl (2*S*,3*R*)-3-cyclohexyl-4,4,4-trifluoro-2-(4-methoxybenzamido)butanoate *syn*-4n.**

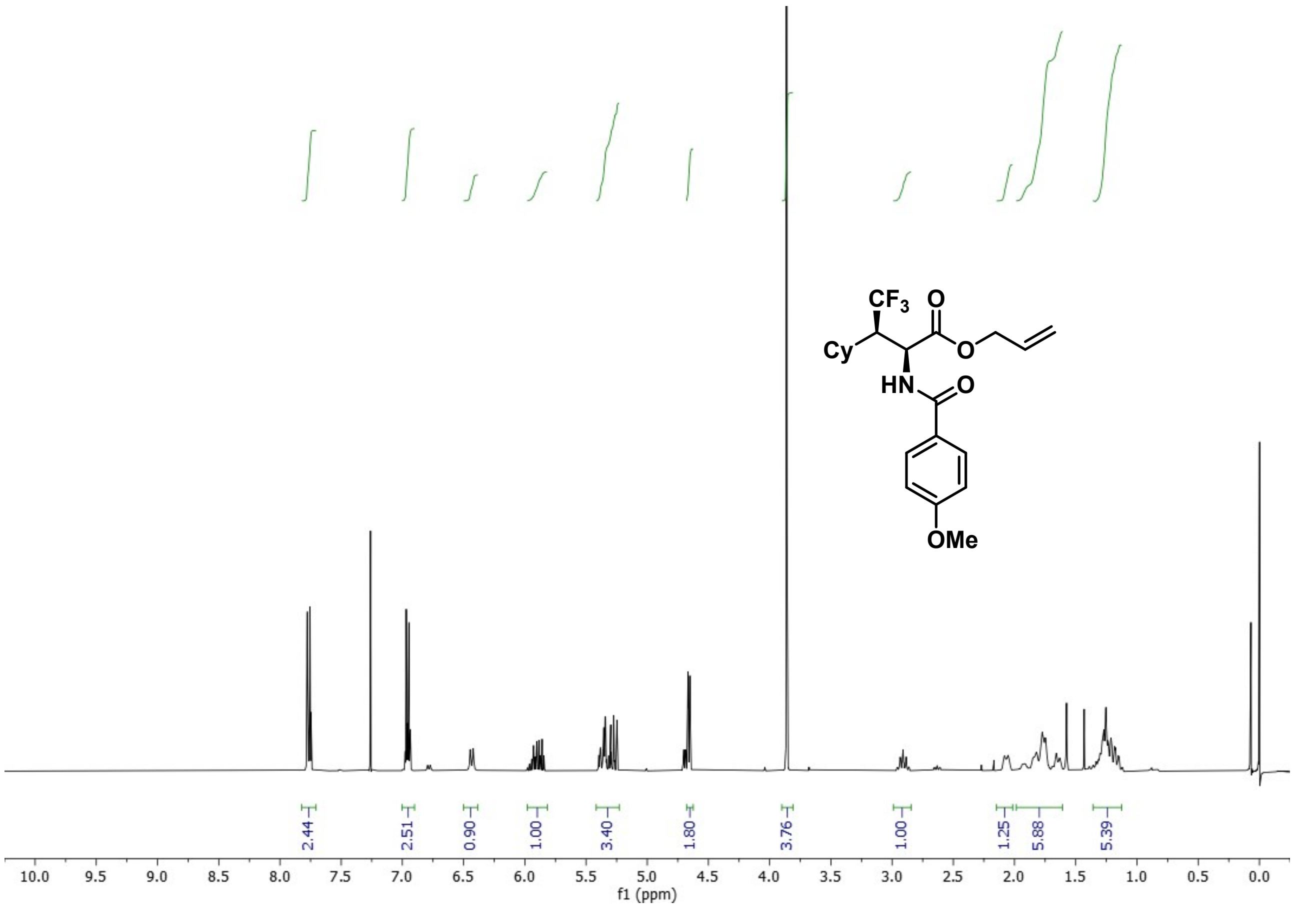

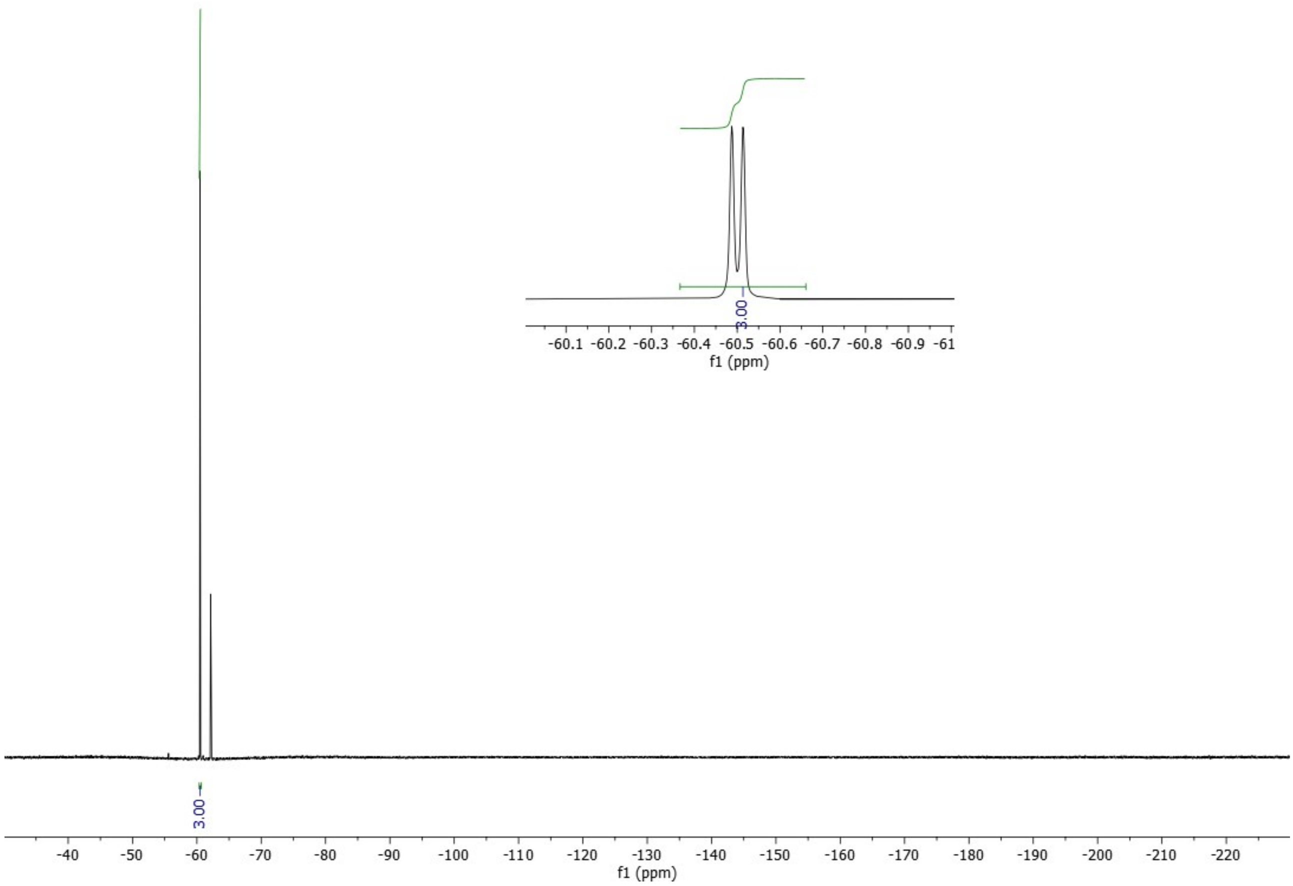
3.00
-60.1 -60.2 -60.3 -60.4 -60.5 -60.6 -60.7 -60.8 -60.9 -61
f1 (ppm)
3.00
-40 -50 -60 -70 -80 -90 -100 -110 -120 -130 -140 -150 -160 -170 -180 -190 -200 -210 -220
f1 (ppm)

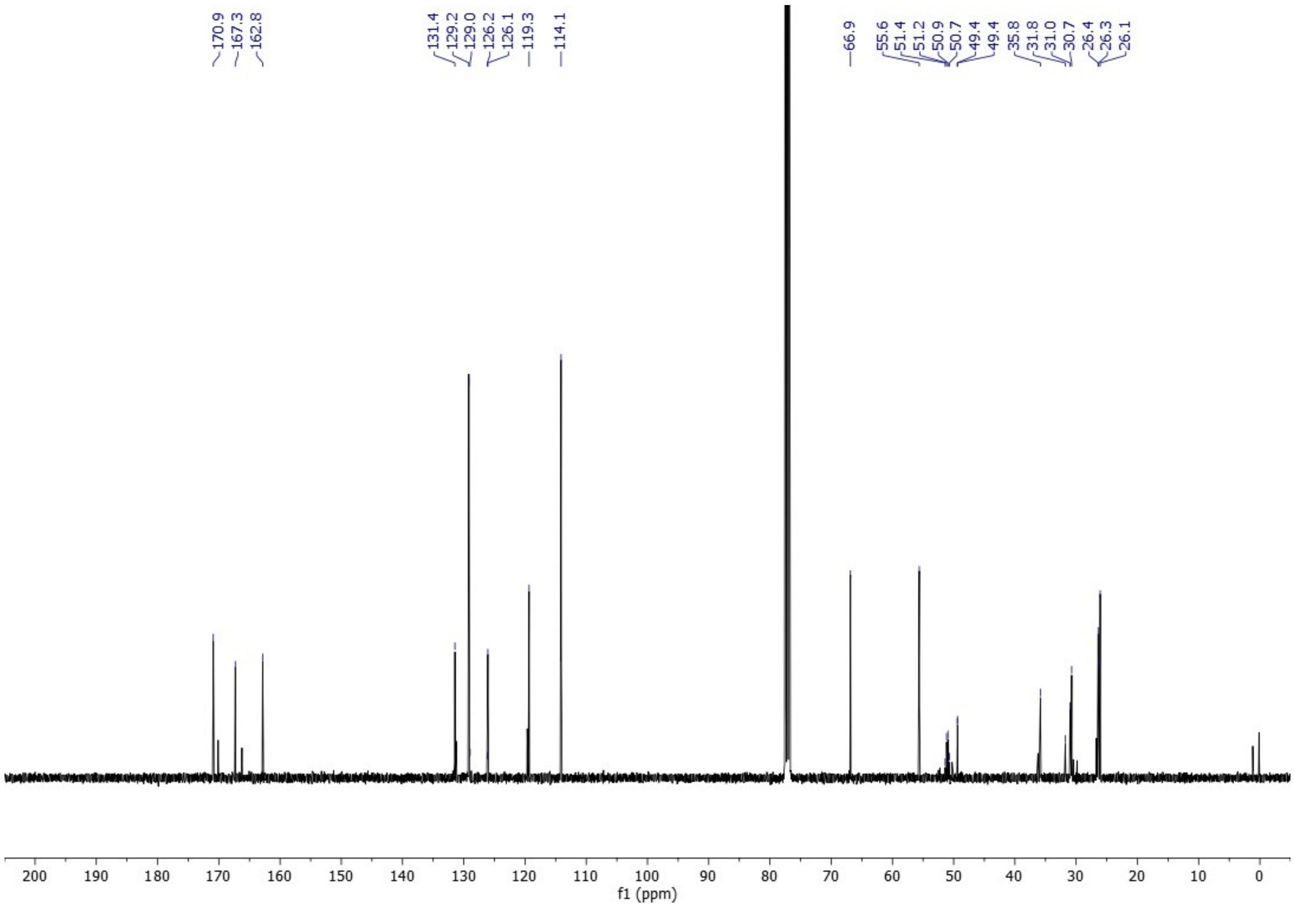
170.9
167.3
162.8
131.4
129.2
129.0
126.2
126.1
119.3
114.1
66.9
55.6
51.4
51.2
50.9
50.7
49.4
49.4
35.8
31.8
31.0
30.7
26.4
26.3
26.1
200 190 180 170 160 150 140 130 120 110 100 90 80 70 60 50 40 30 20 10 0
f1 (ppm)

**Allyl (2*R*,3*R*)-2-(4-methoxybenzamido)-3-(trifluoromethyl)pentanoate *anti*-4o**.

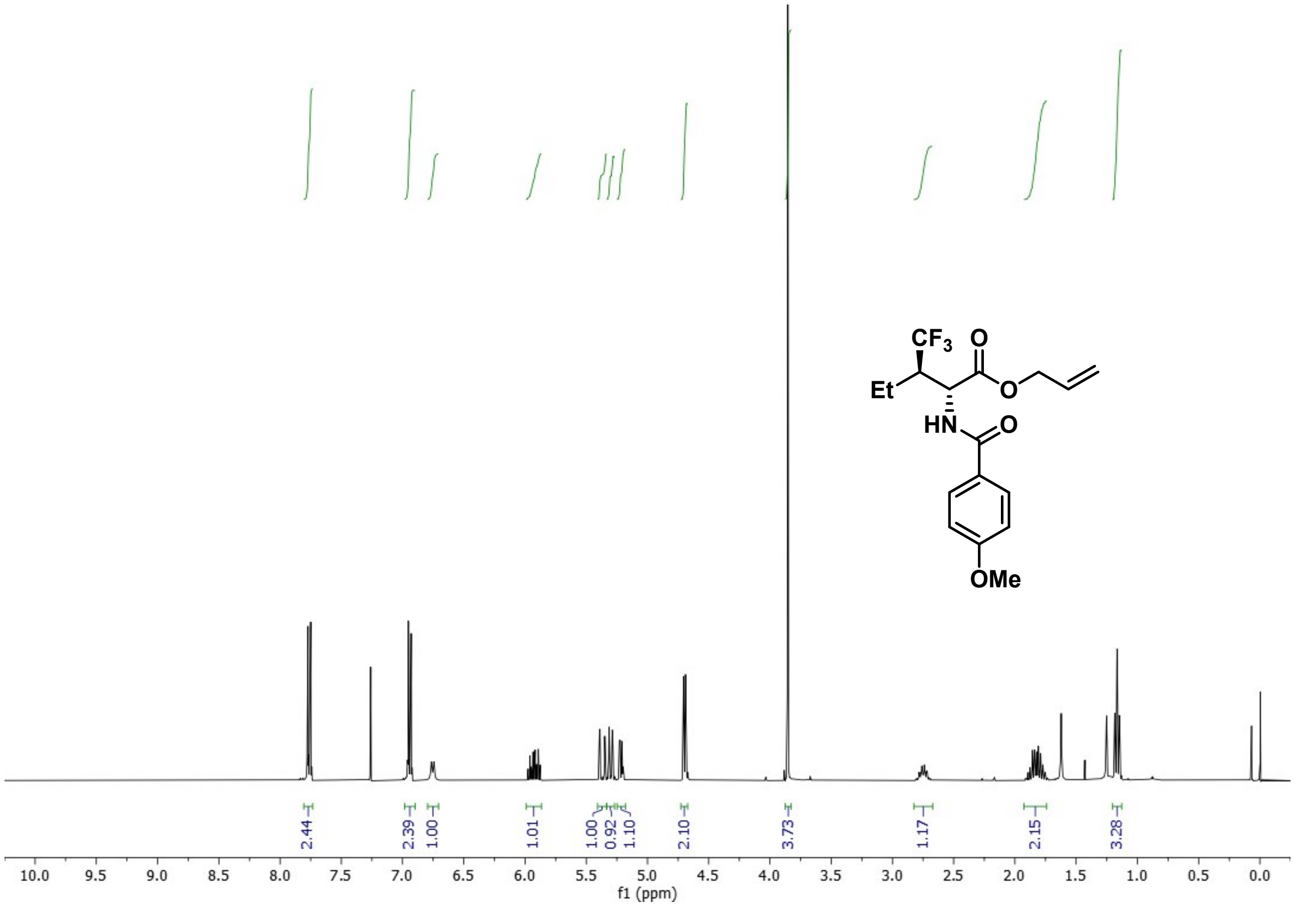

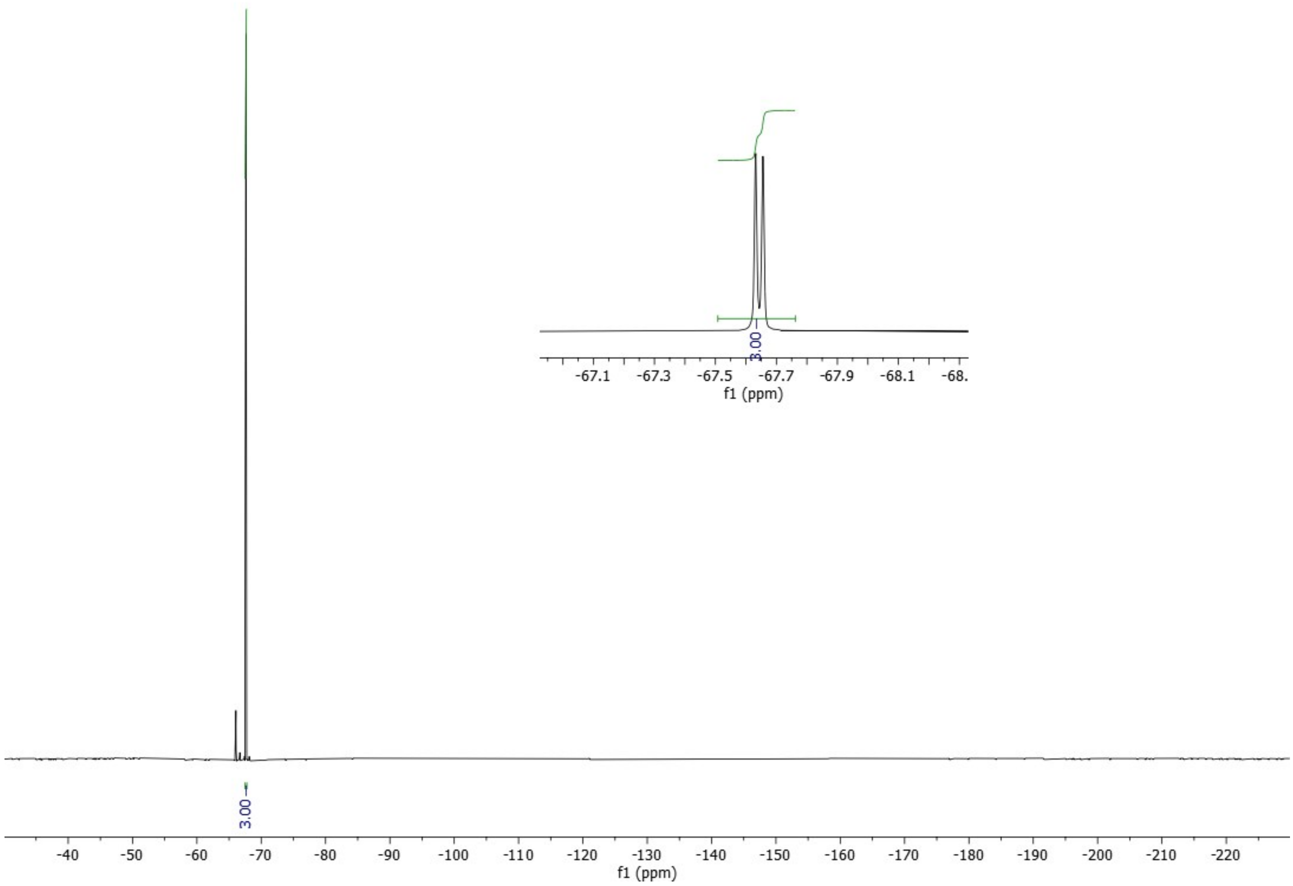
3.00
-67.1
-67.3
-67.5
-67.7
-67.9
-68.1
f1 (ppm)
3.00
-40
-50
-60
-70
-80
-90
-100
-110
-120
-130
-140
-150
-160
-170
-180
-190
-200
-210
-220
f1 (ppm)

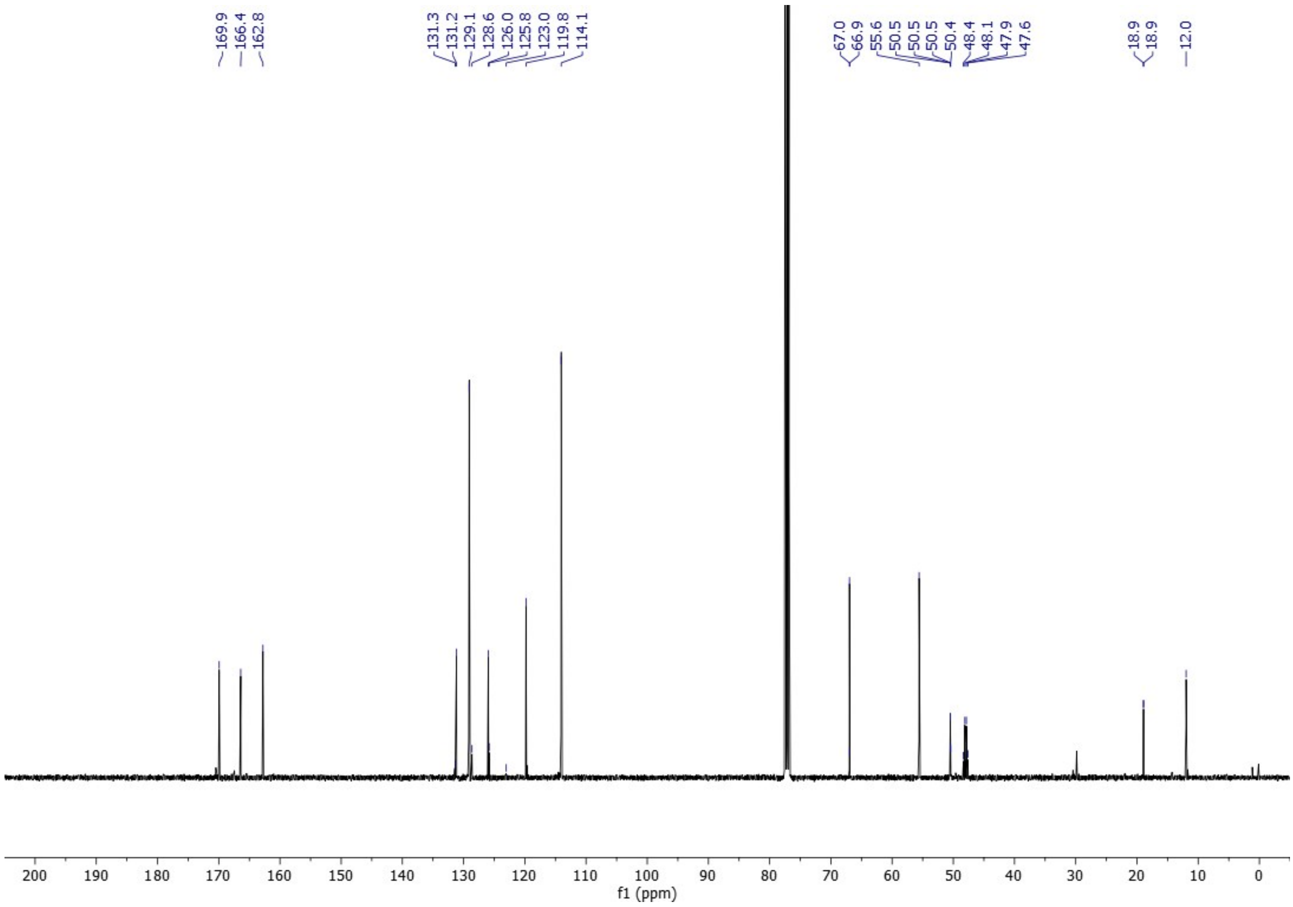
169.9
166.4
162.8
131.3
131.2
129.1
128.6
126.0
125.8
123.0
119.8
114.1
67.0
66.9
55.6
50.5
50.5
50.5
50.4
48.4
48.1
47.9
47.6
18.9
18.9
12.0
200
190
180
170
160
150
140
130
120
110
100
90
80
70
60
50
40
30
20
10
0
f1 (ppm)

**Allyl (2*S*,3*R*)-2-(4-methoxybenzamido)-3-(trifluoromethyl)pentanoate *syn*-4o**.

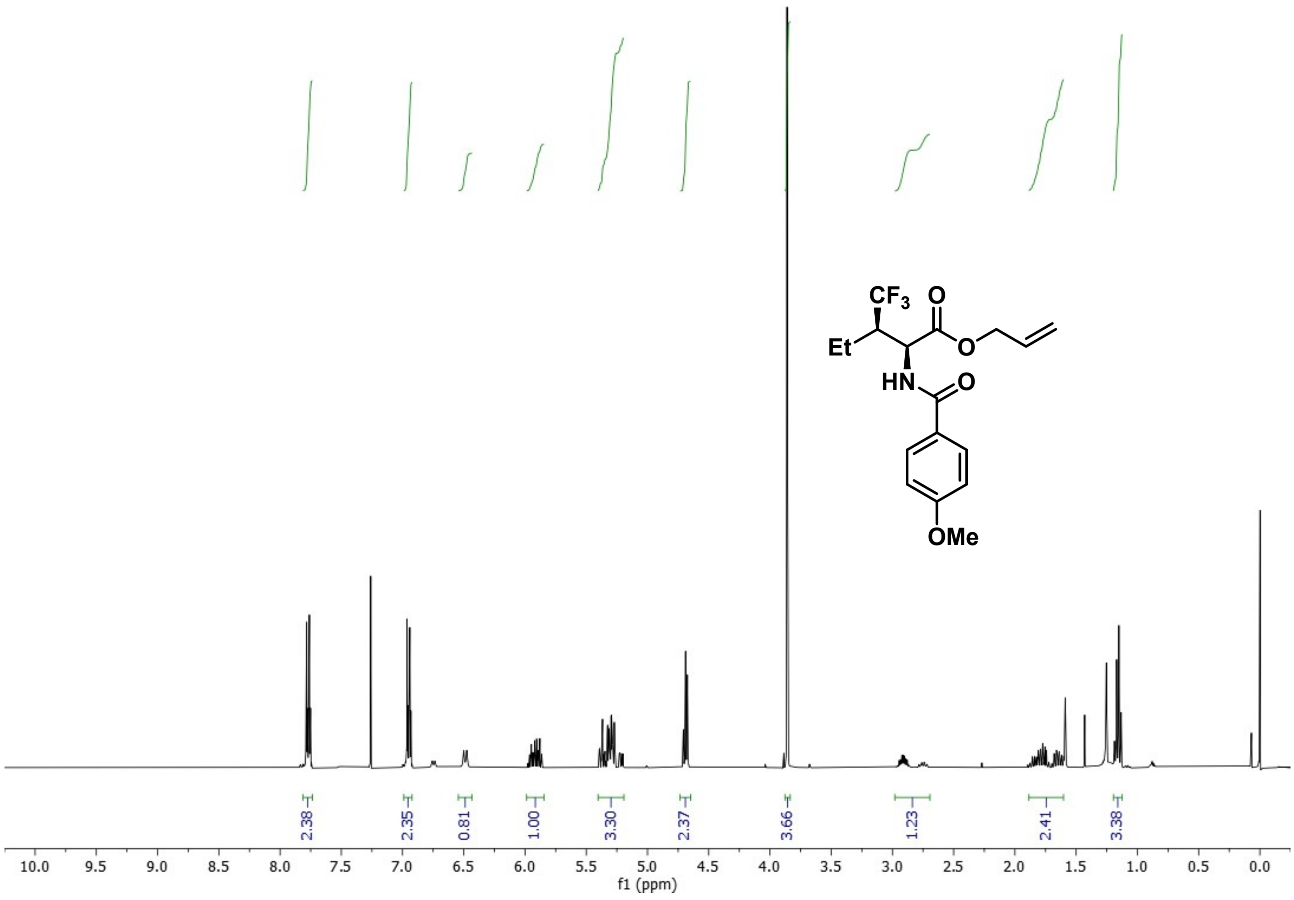

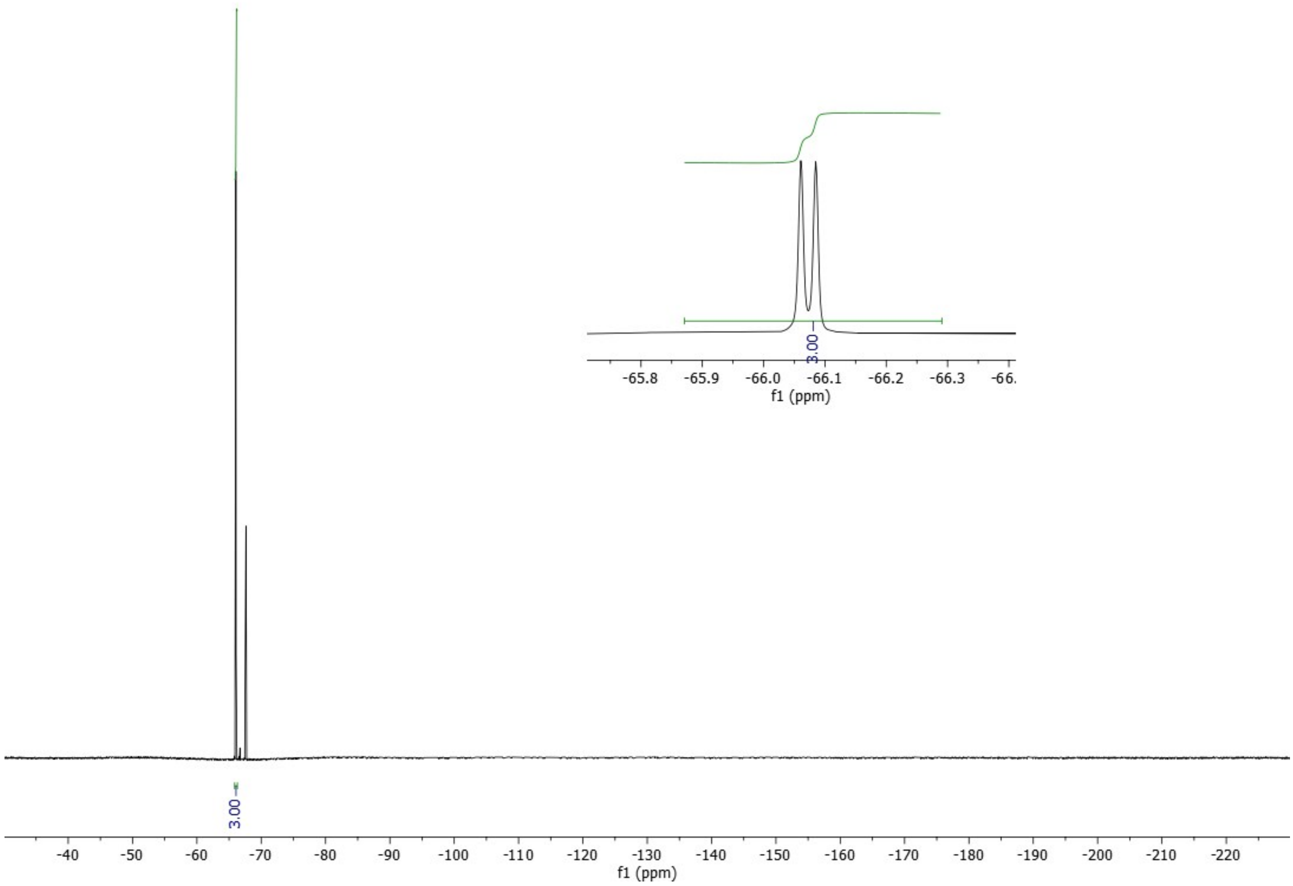

3.00
-65.8 -65.9 -66.0 -66.1 -66.2 -66.3
f1 (ppm)
3.00
-40 -50 -60 -70 -80 -90 -100 -110 -120 -130 -140 -150 -160 -170 -180 -190 -200 -210 -220
f1 (ppm)


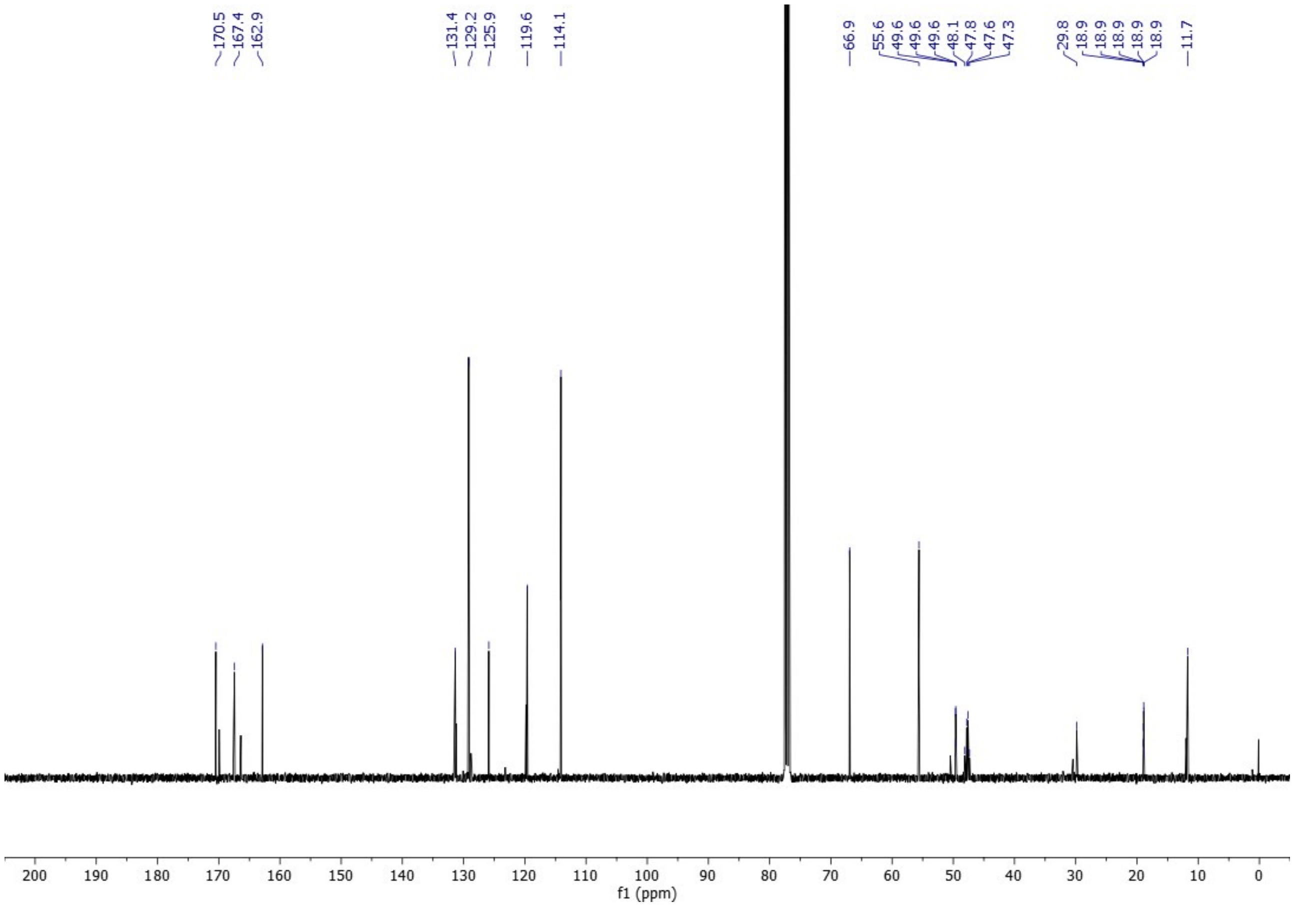

170.5
167.4
162.9
131.4
129.2
125.9
119.6
114.1
66.9
55.6
49.6
49.6
49.6
48.1
47.8
47.6
47.3
29.8
18.9
18.9
18.9
18.9
18.9
11.7
200 190 180 170 160 150 140 130 120 110 100 90 80 70 60 50 40 30 20 10 0
f1 (ppm)

**Allyl (2*R*,3*R*)-4,4,4-trifluoro-2-(4-methoxybenzamido)-3-methylbutanoate *anti*-4p**.

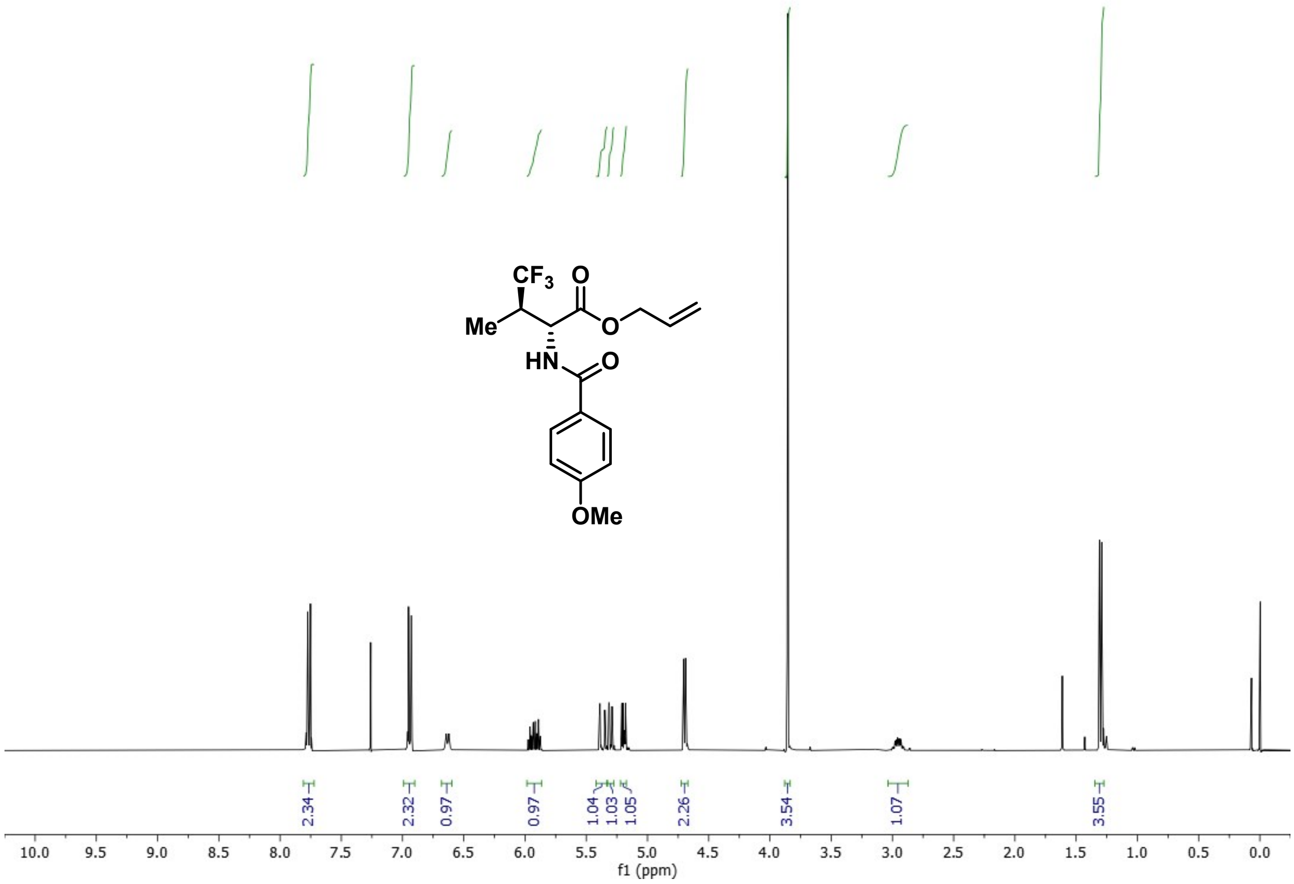

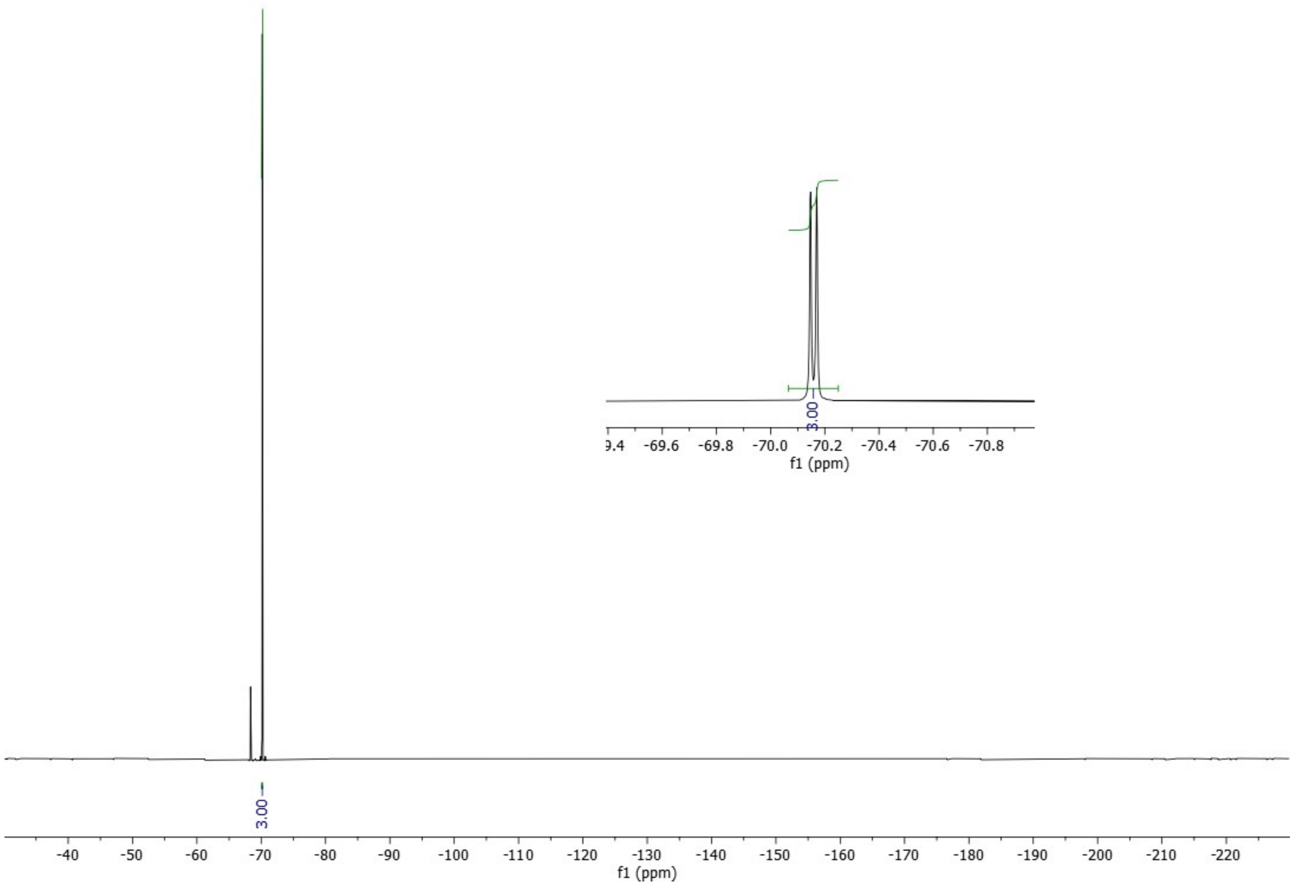

3.00
-40
-50
-60
-70
-80
-90
-100
-110
-120
-130
-140
-150
-160
-170
-180
-190
-200
-210
-220
f1 (ppm)
-69.6
-69.8
-70.0
-70.2
-70.4
-70.6
-70.8


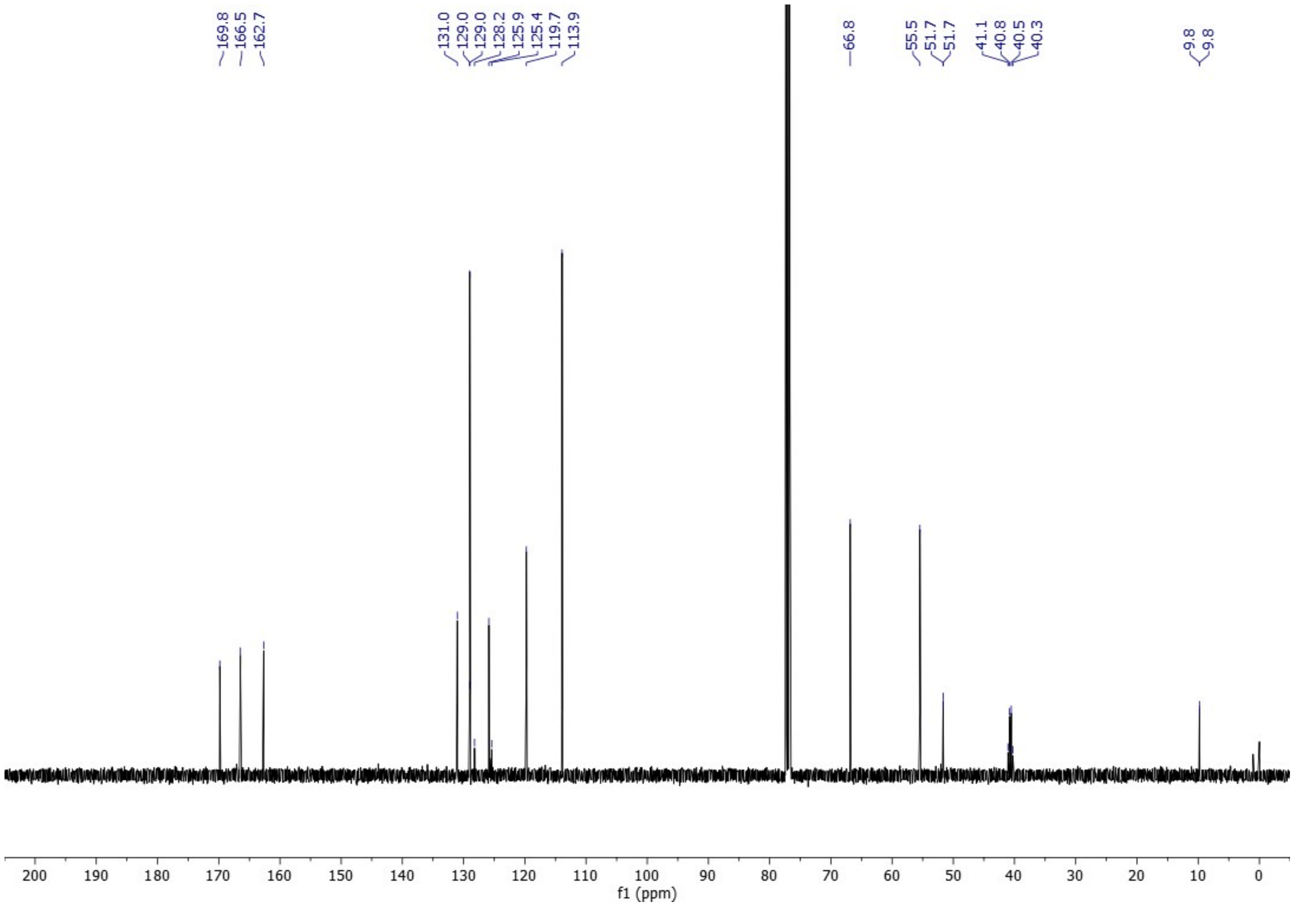

169.8
166.5
162.7
131.0
129.0
129.0
128.2
125.9
125.4
119.7
113.9
66.8
55.5
51.7
51.7
41.1
40.8
40.5
40.3
9.8
9.8
200
190
180
170
160
150
140
130
120
110
100
90
80
70
60
50
40
30
20
10
0
f1 (ppm)

**Allyl (2*S*,3*R*)-4,4,4-trifluoro-2-(4-methoxybenzamido)-3-methylbutanoate *syn*-4p**.

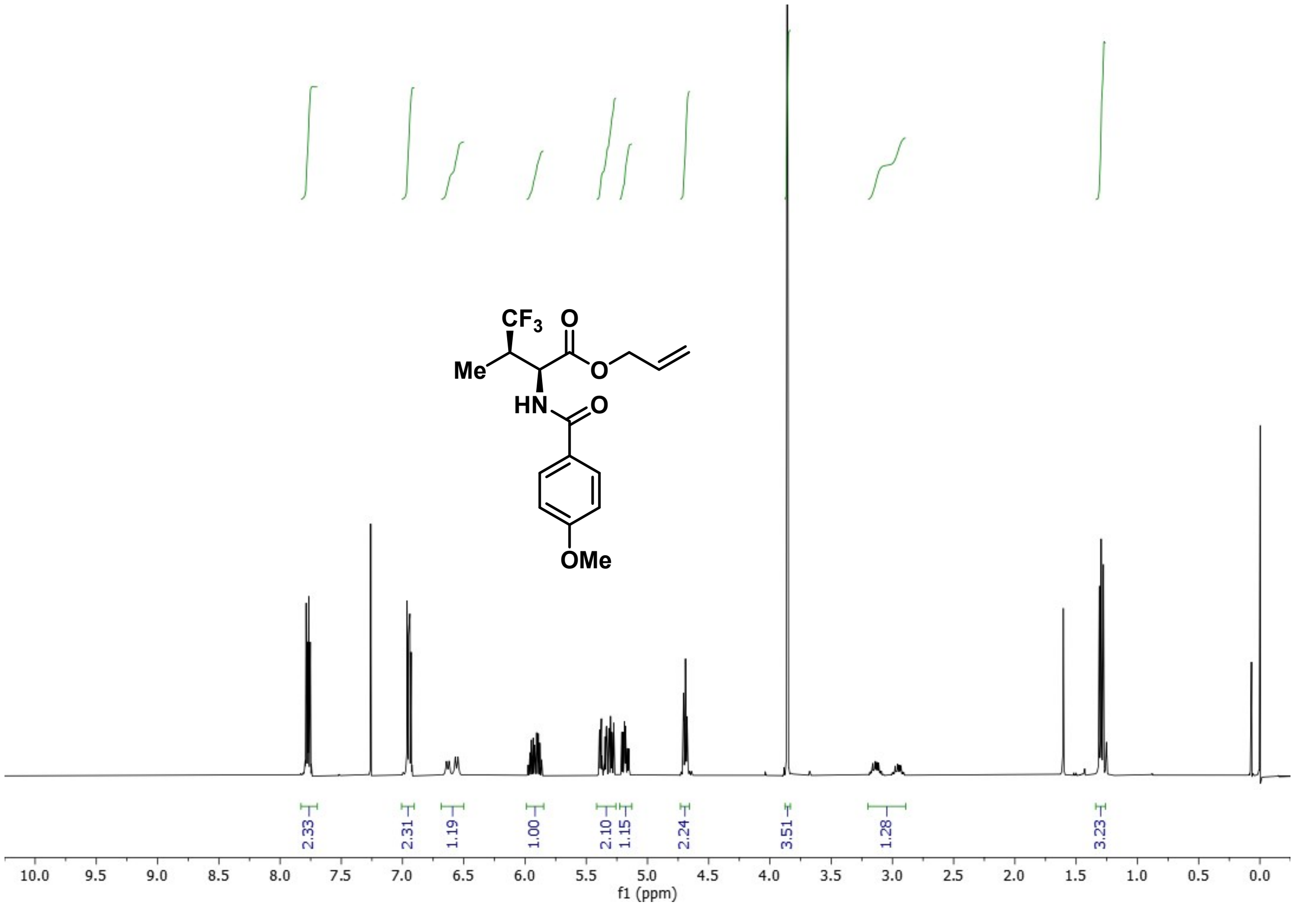

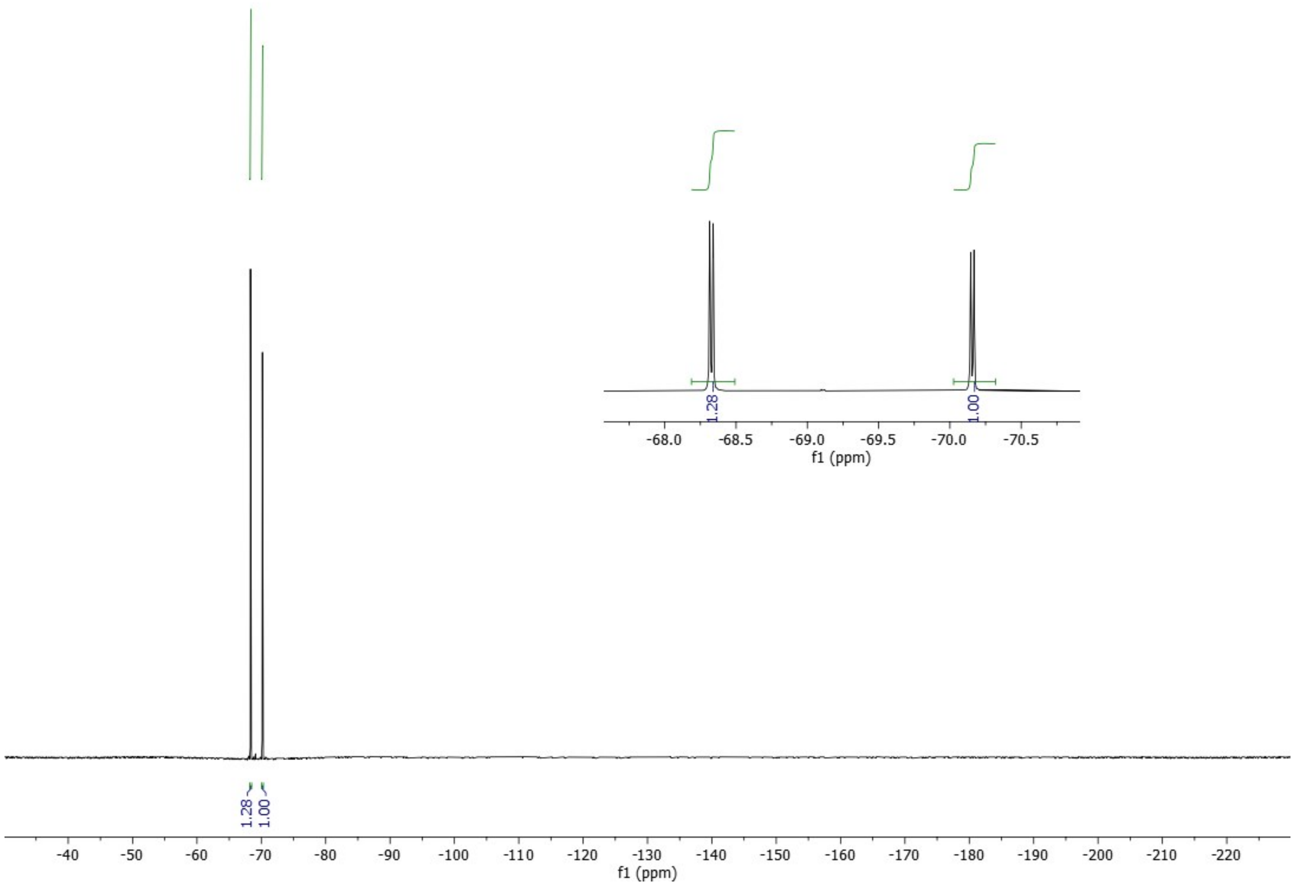
1.28
1.00
-68.0
-68.5
-69.0
-69.5
-70.0
-70.5
f1 (ppm)
-40
-50
-60
-70
-80
-90
-100
-110
-120
-130
-140
-150
-160
-170
-180
-190
-200
-210
-220

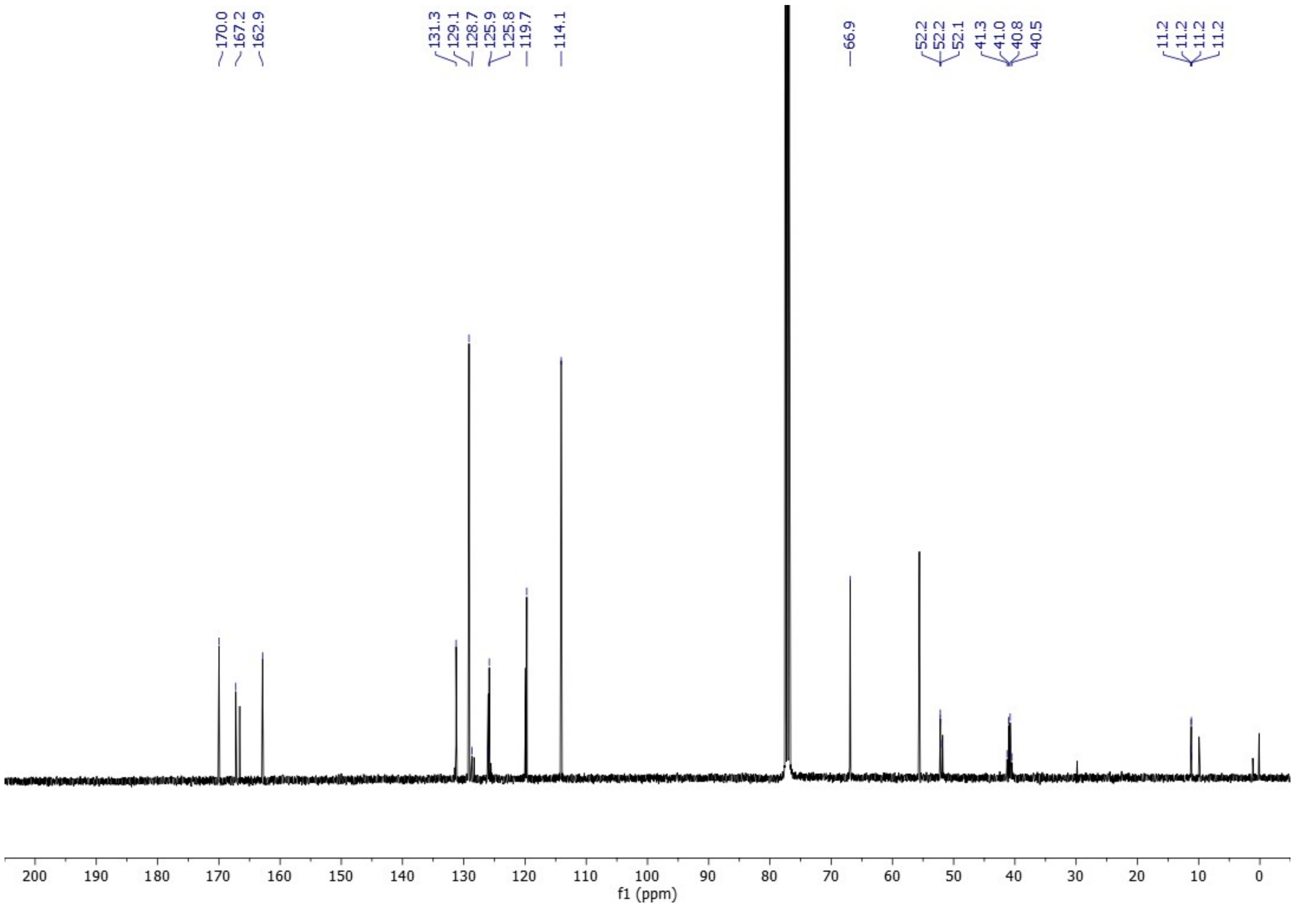
170.0
167.2
162.9
131.3
129.1
128.7
125.9
125.8
119.7
114.1
66.9
52.2
52.2
52.1
41.3
41.0
40.8
40.5
11.2
11.2
11.2
11.2
200
190
180
170
160
150
140
130
120
110
100
90
80
70
60
50
40
30
20
10
0
f1 (ppm)

**Methyl (2*R*,3*R*)-4,4,4-trifluoro-2-(4-methoxybenzamido)-3-phenylbutanoate *anti*-4q.**

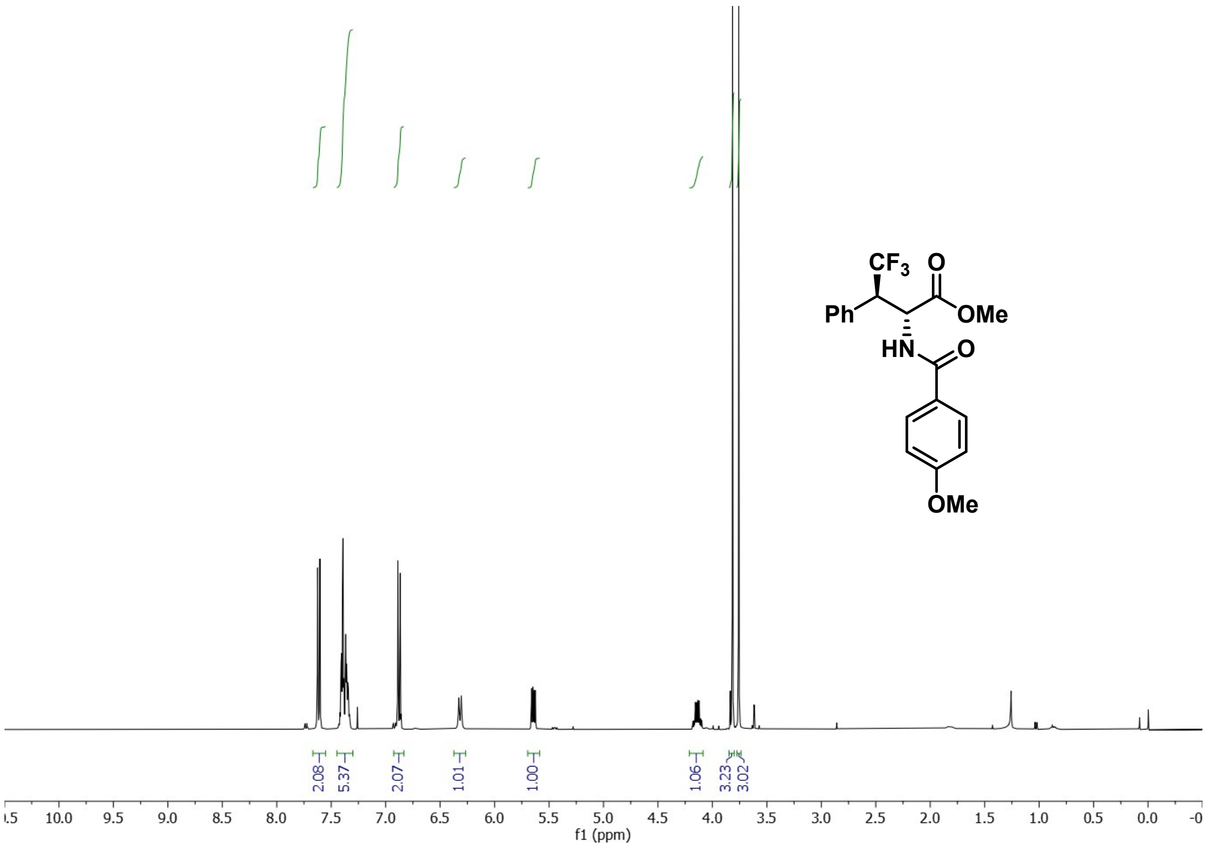


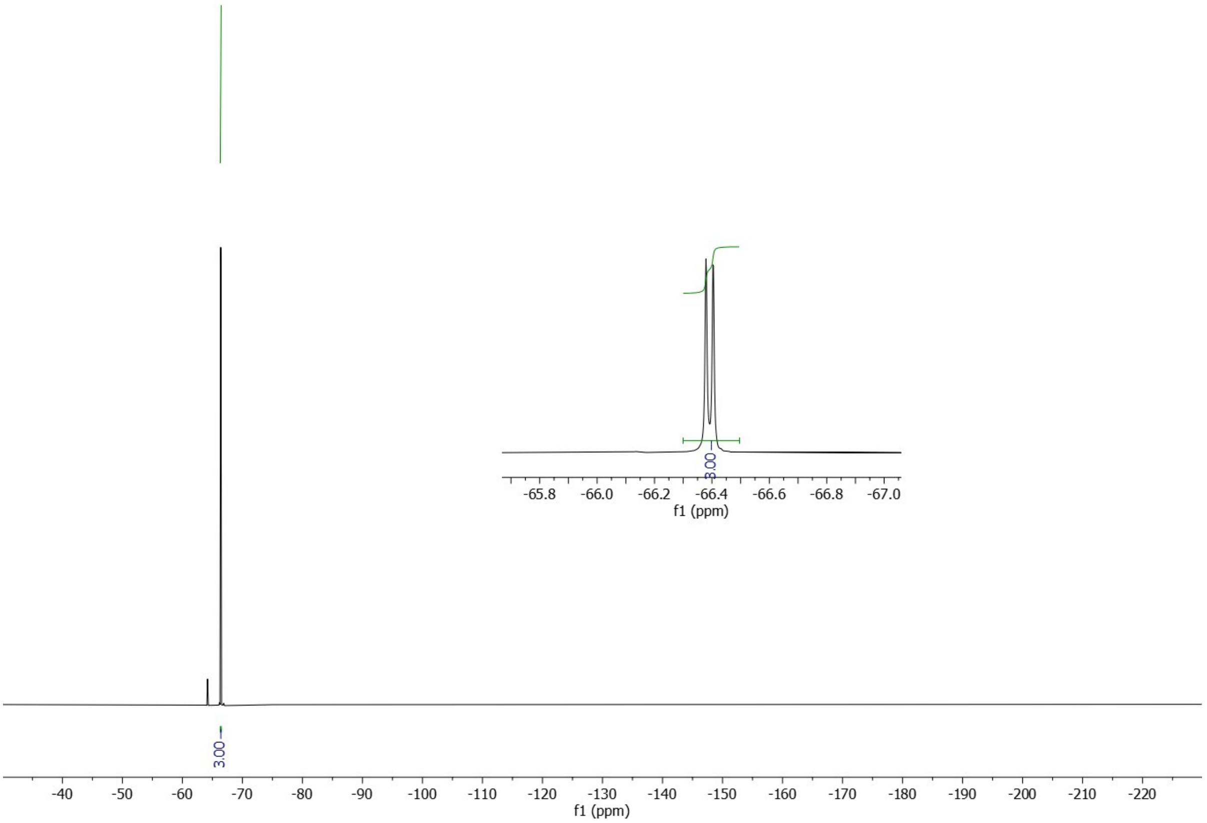

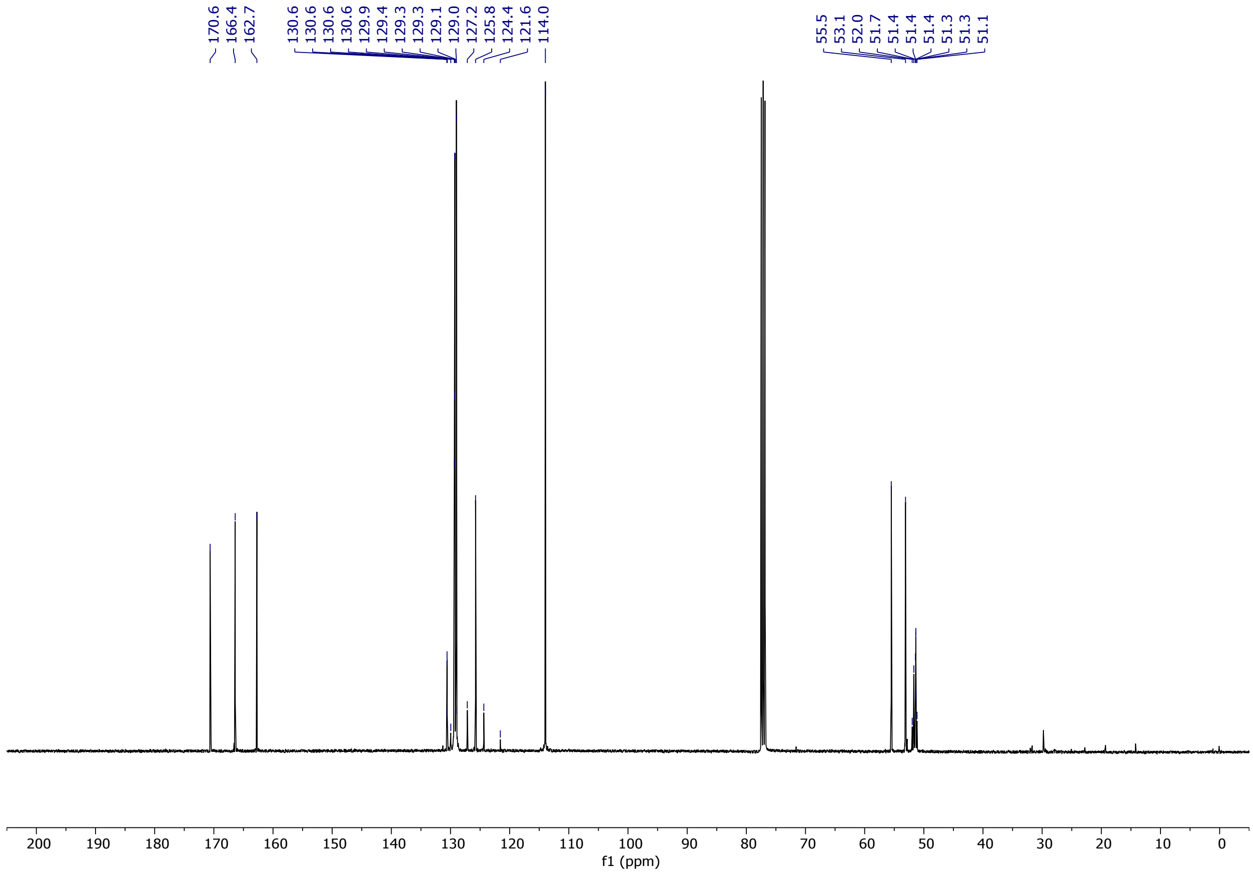
170.6
166.4
162.7
130.6
130.6
130.6
130.6
129.9
129.4
129.3
129.3
129.1
129.0
127.2
125.8
124.4
121.6
114.0
55.5
53.1
52.0
51.7
51.4
51.4
51.4
51.3
51.3
51.1
200 190 180 170 160 150 140 130 120 110 100 90 80 70 60 50 40 30 20 10 0
f1 (ppm)

**Methyl (2*S*,3*R*)-4,4,4-trifluoro-2-(4-methoxybenzamido)-3-phenylbutanoate (in $CD_2Cl_2$)**
*syn*-**4q**.

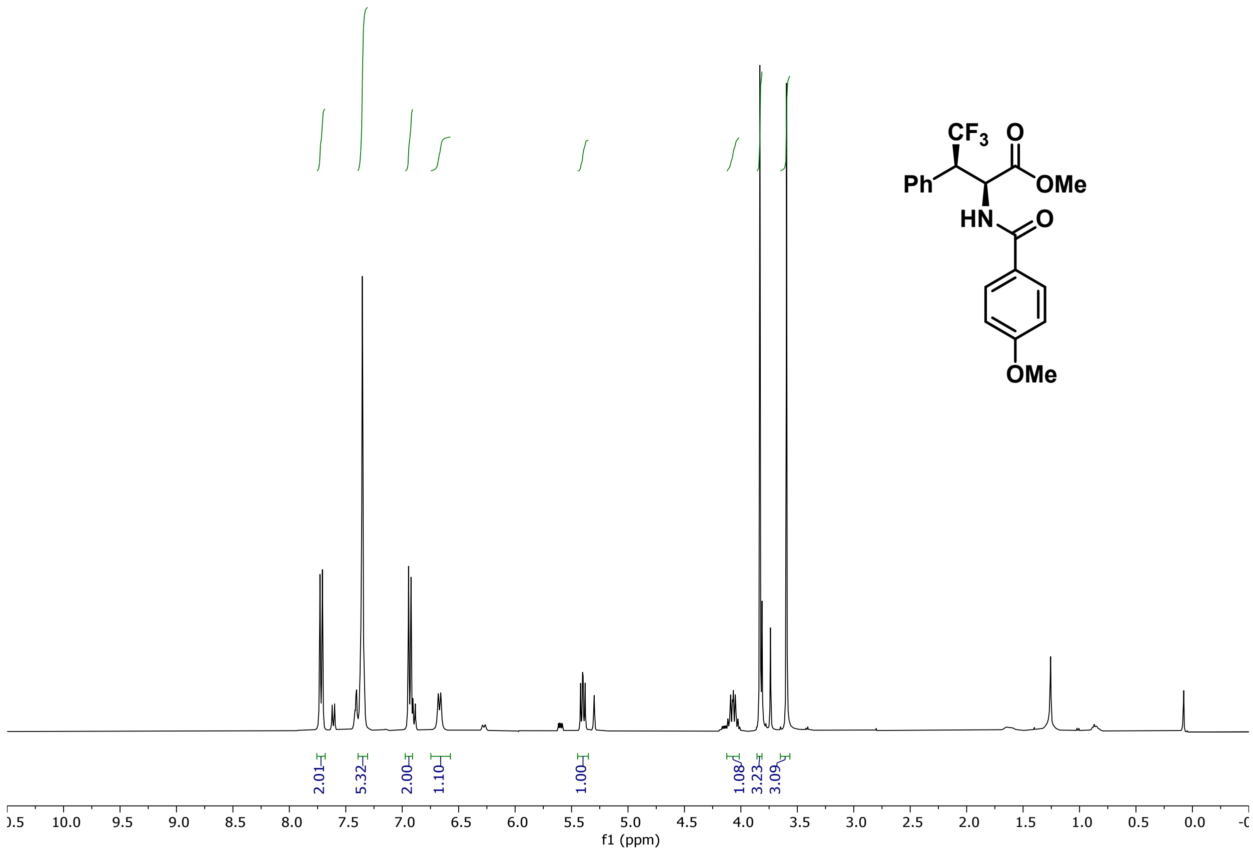

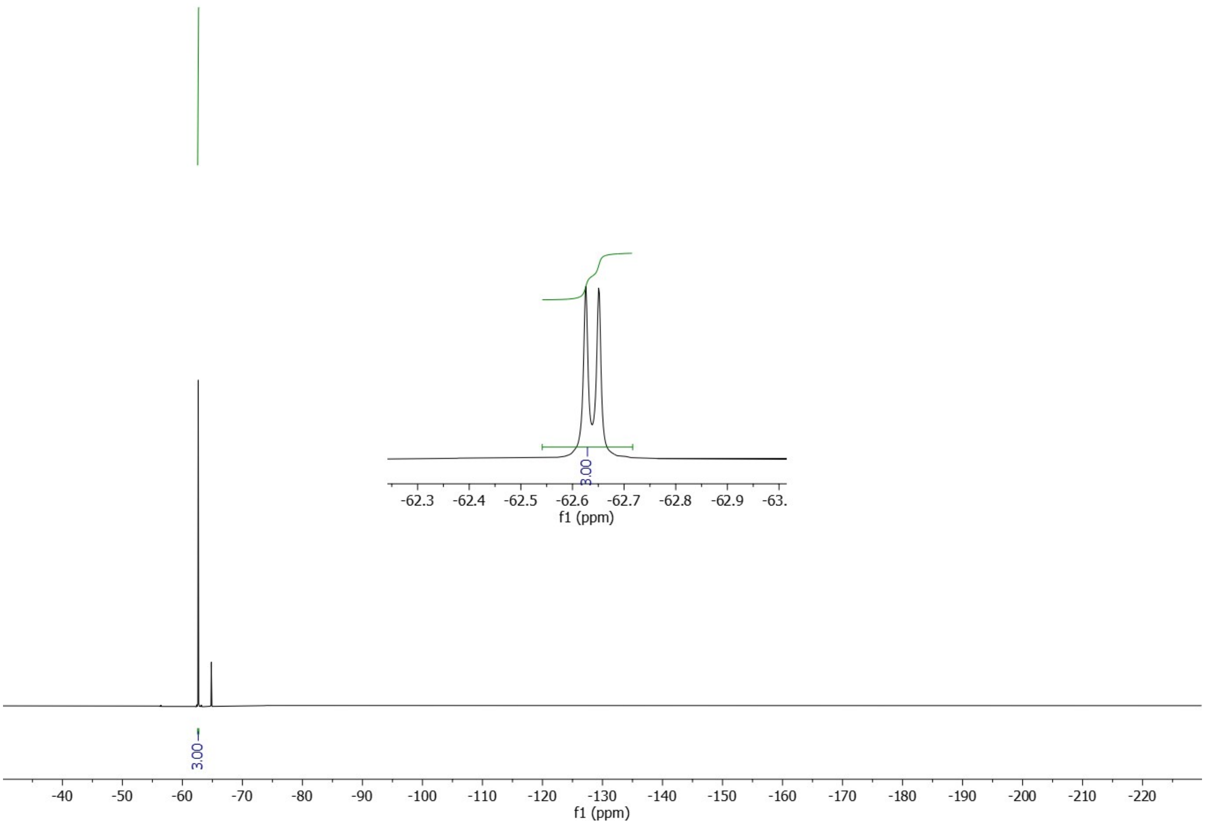
3.00
-62.3 -62.4 -62.5 -62.6 -62.7 -62.8 -62.9 -63.
f1 (ppm)
-40 -50 -60 -70 -80 -90 -100 -110 -120 -130 -140 -150 -160 -170 -180 -190 -200 -210 -220
f1 (ppm)

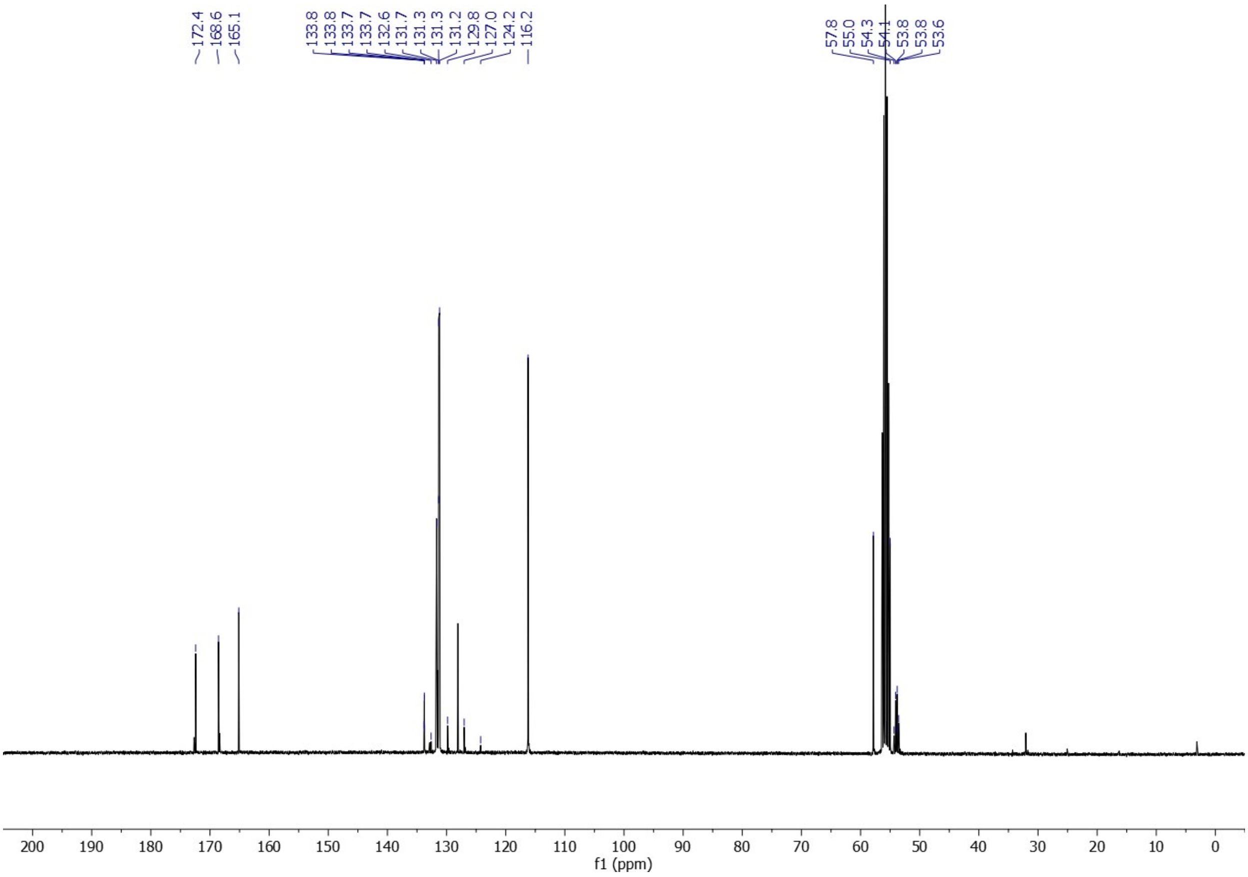
172.4
168.6
165.1
133.8
133.8
133.7
133.7
132.6
131.7
131.3
131.3
131.2
129.8
127.0
124.2
116.2
57.8
55.0
54.3
54.1
53.8
53.8
53.6
200 190 180 170 160 150 140 130 120 110 100 90 80 70 60 50 40 30 20 10 0
f1 (ppm)

**Benzyl (2*R*,3*R*)-4,4,4-trifluoro-2-(4-methoxybenzamido)-3-phenylbutanoate *anti*-4r.**

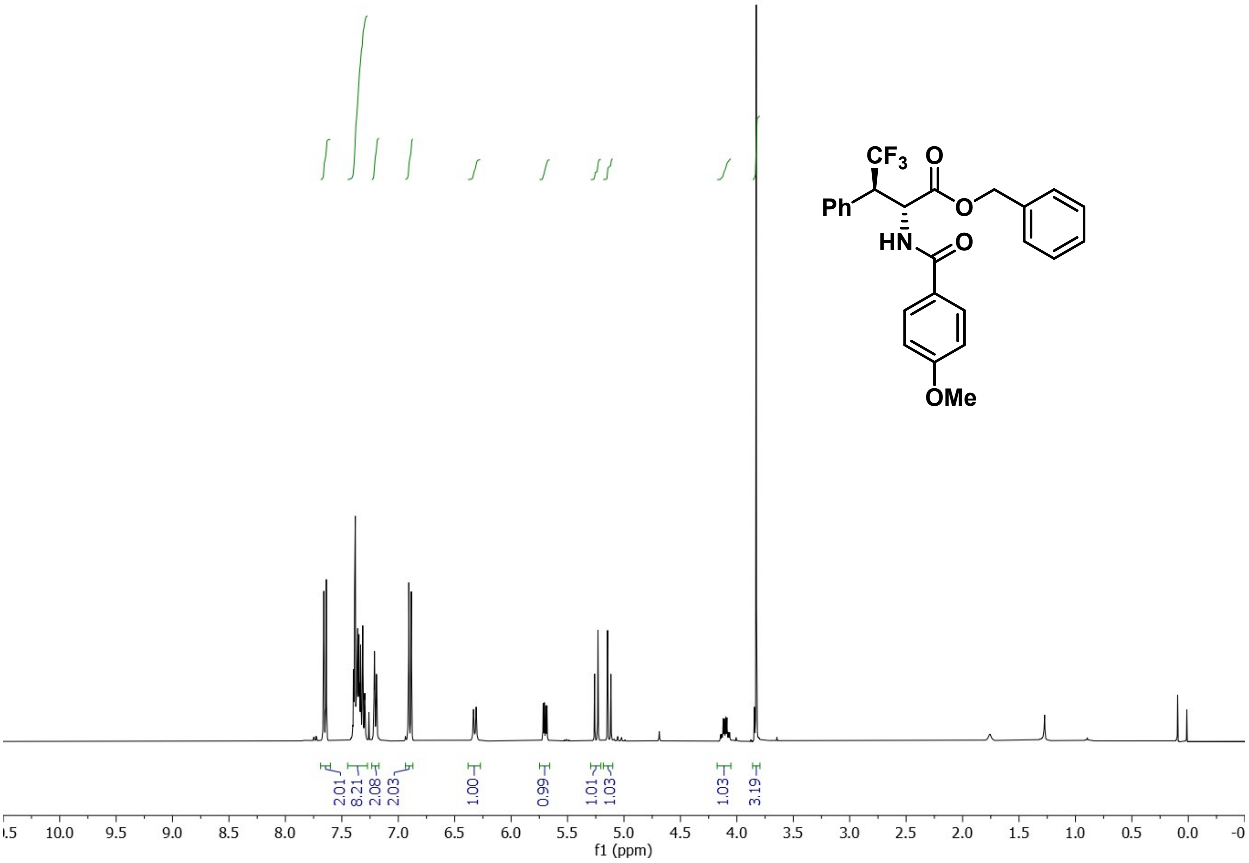


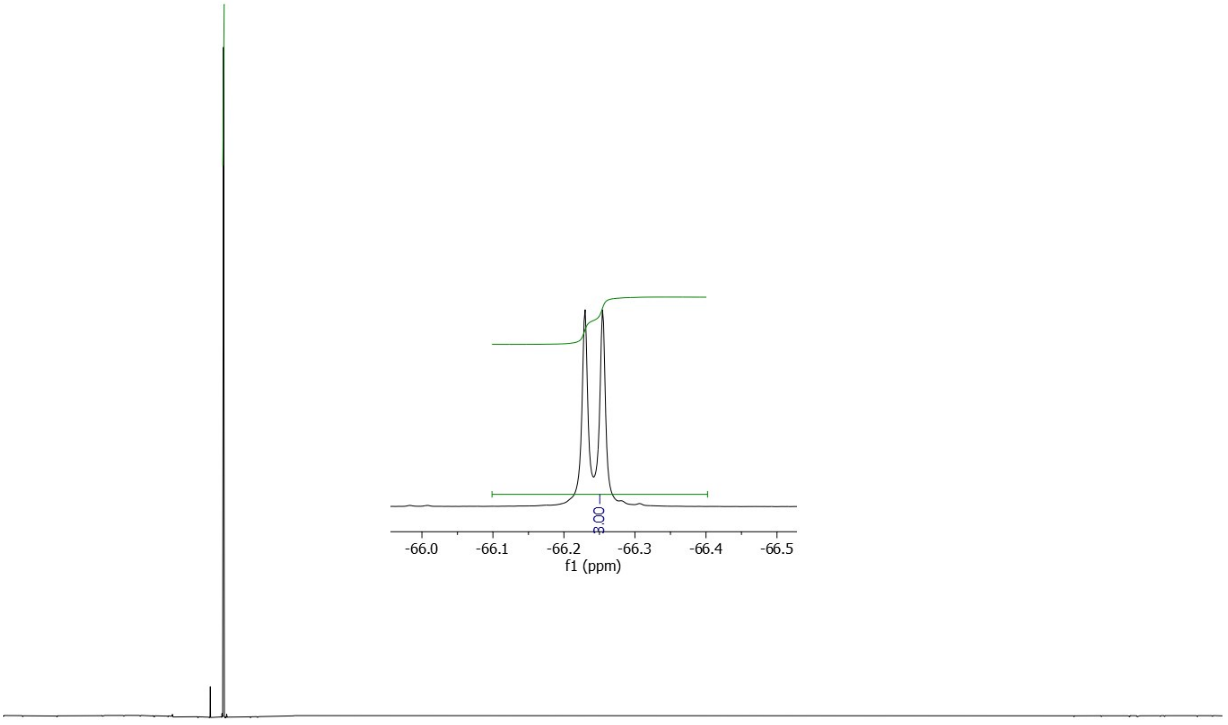

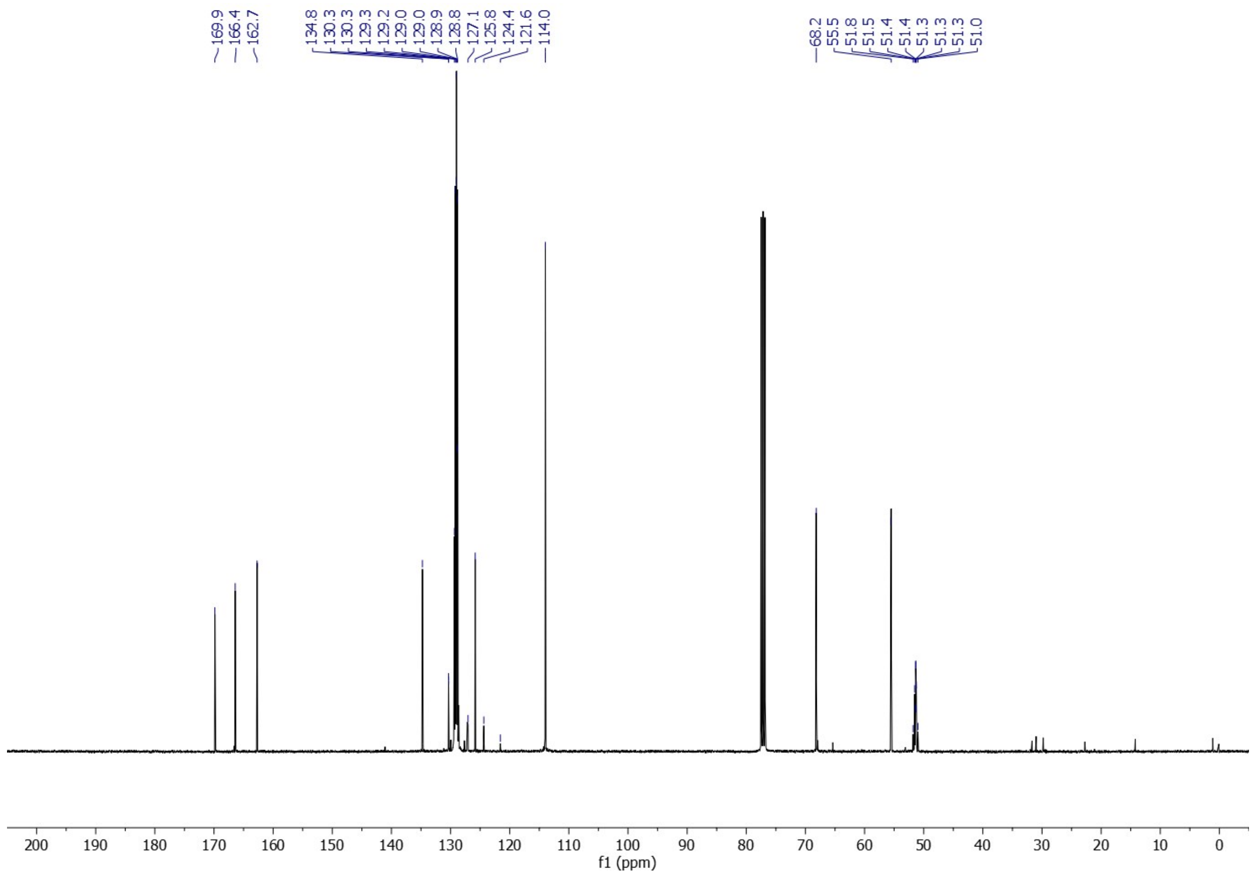
169.9
166.4
162.7
134.8
130.3
130.3
129.3
129.2
129.0
129.0
128.9
128.8
127.1
125.8
124.4
121.6
114.0
68.2
55.5
51.8
51.5
51.4
51.4
51.3
51.3
51.3
51.0
200
190
180
170
160
150
140
130
120
110
100
90
80
70
60
50
40
30
20
10
0
f1 (ppm)

**Benzyl (2*S*,3*R*)-4,4,4-trifluoro-2-(4-methoxybenzamido)-3-phenylbutanoate *syn*-4r.**

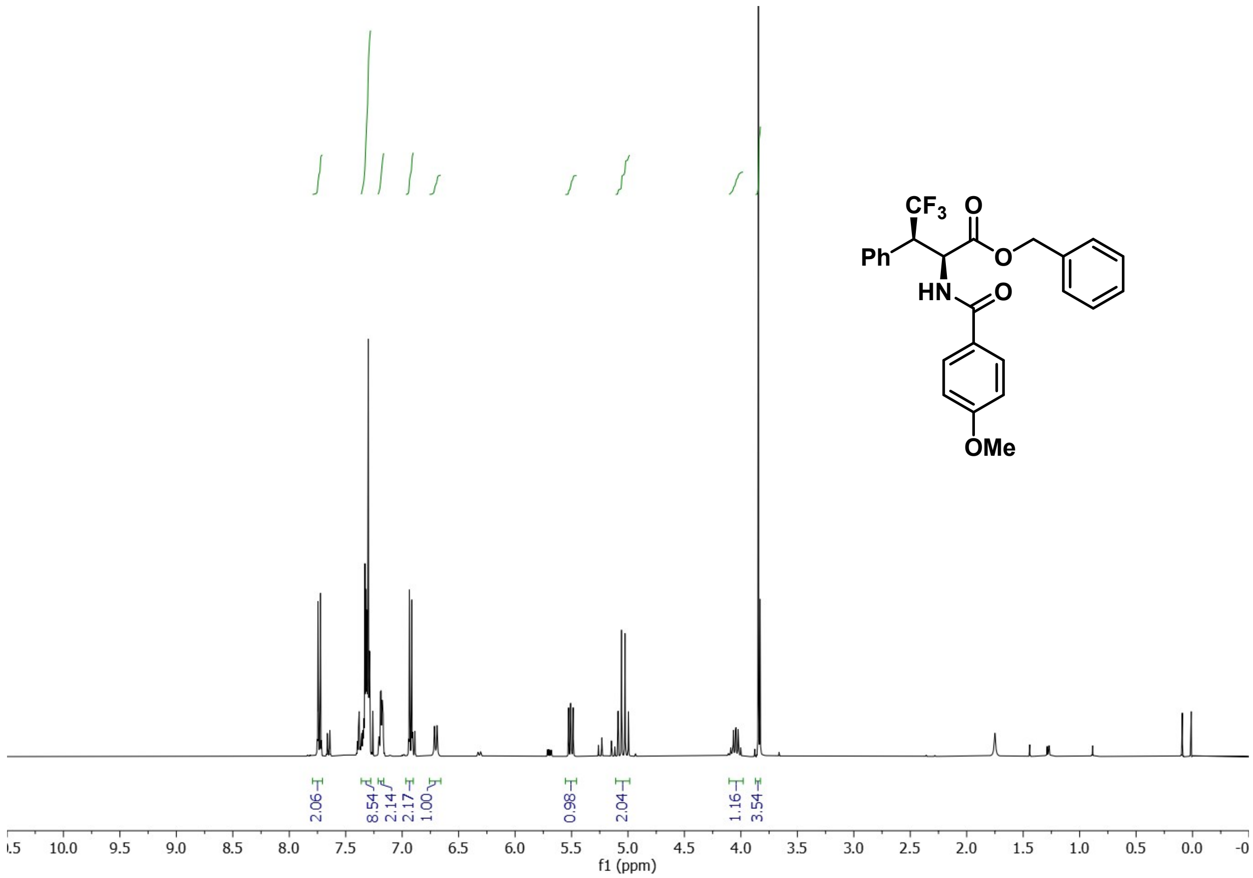


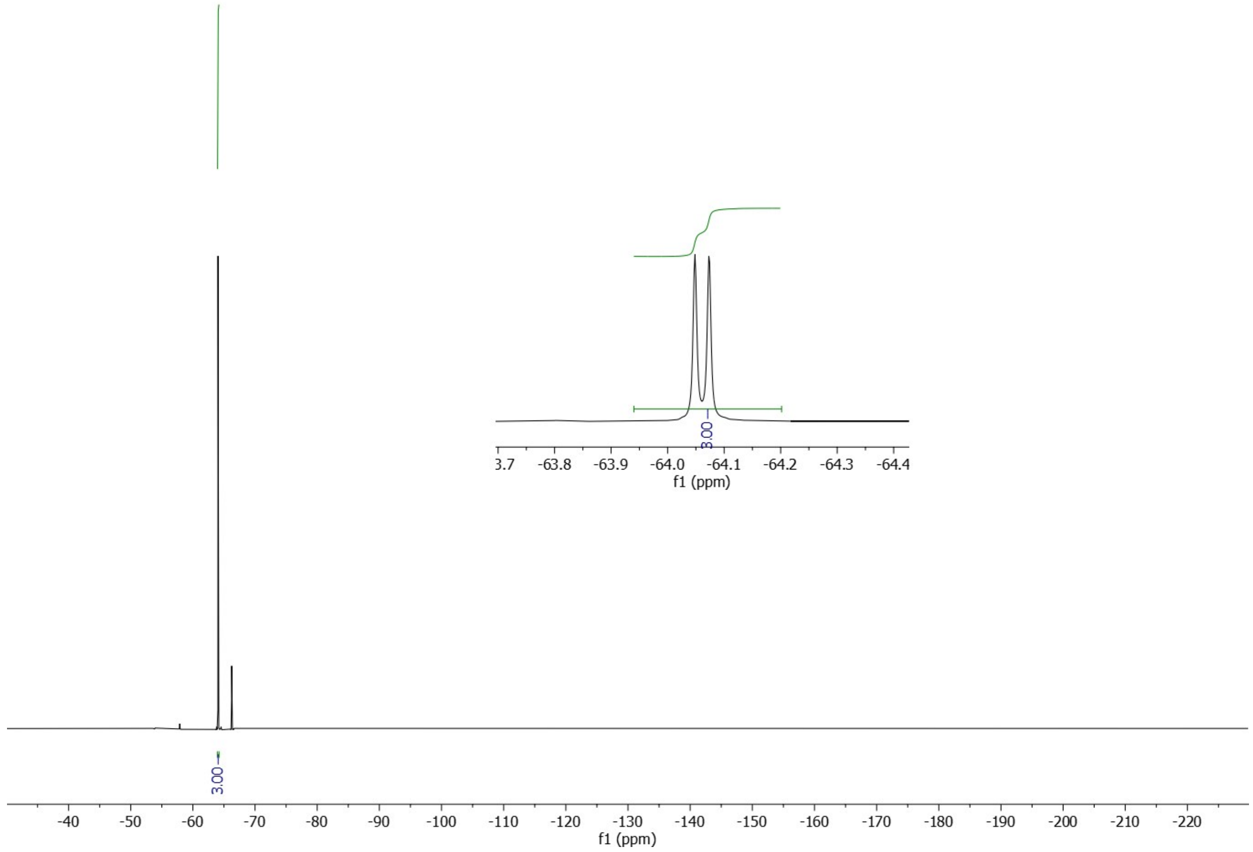

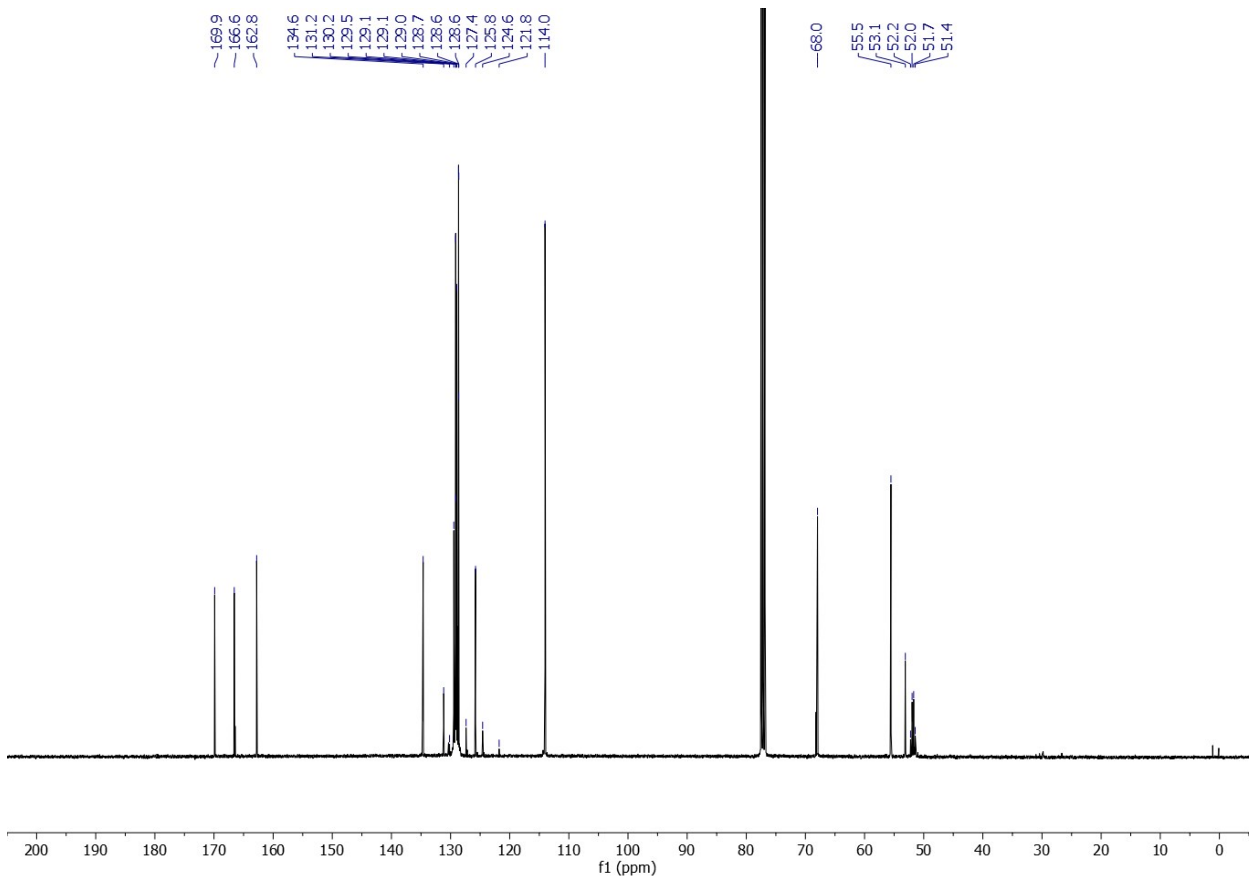
169.9
166.6
162.8
134.6
131.2
130.2
129.5
129.1
129.1
129.0
128.7
128.6
128.6
127.4
125.8
124.6
121.8
114.0
68.0
55.5
53.1
52.2
52.0
51.7
51.4
200
190
180
170
160
150
140
130
120
110
100
90
80
70
60
50
40
30
20
10
0
f1 (ppm)

**Isobutyl (2*R*,3*R*)-4,4,4-trifluoro-2-(4-methoxybenzamido)-3-phenylbutanoate *anti*-4s**.

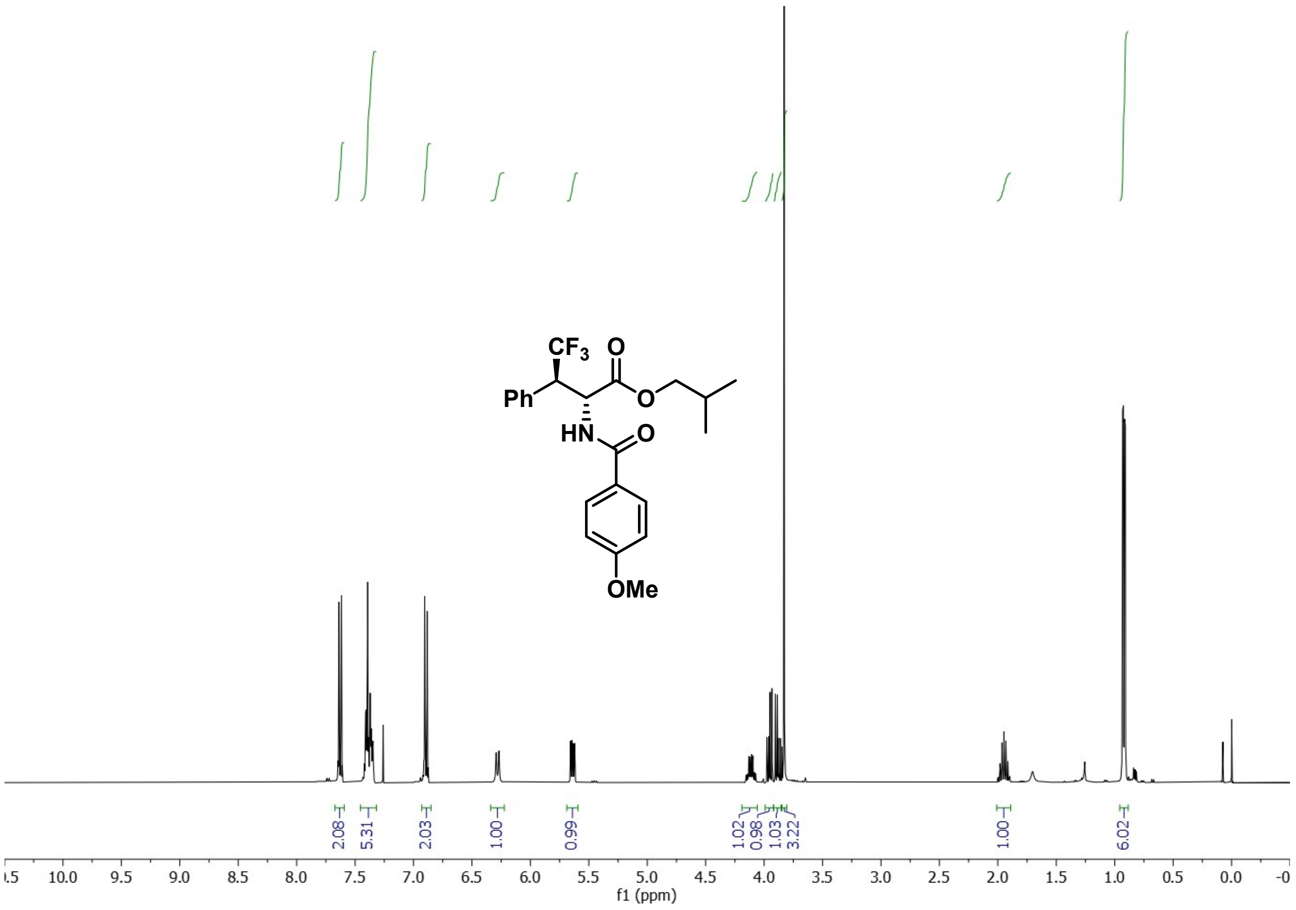


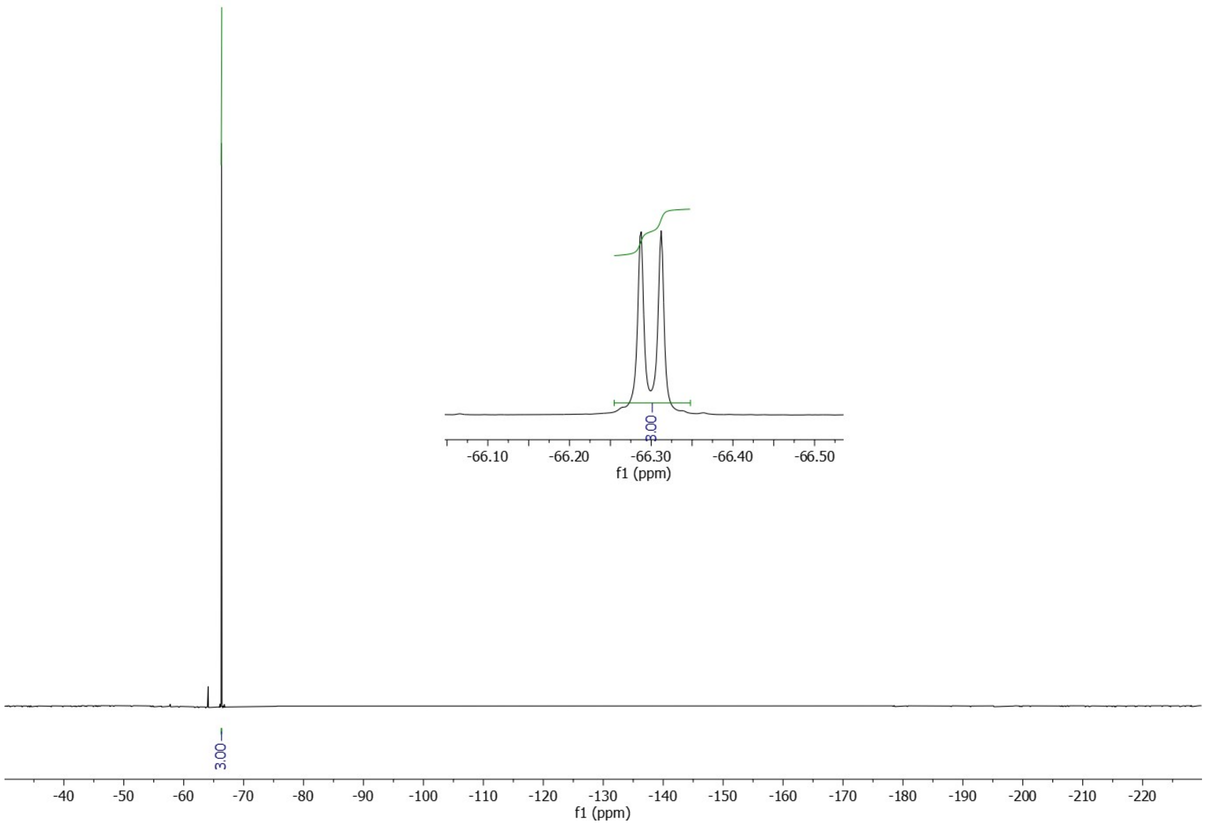

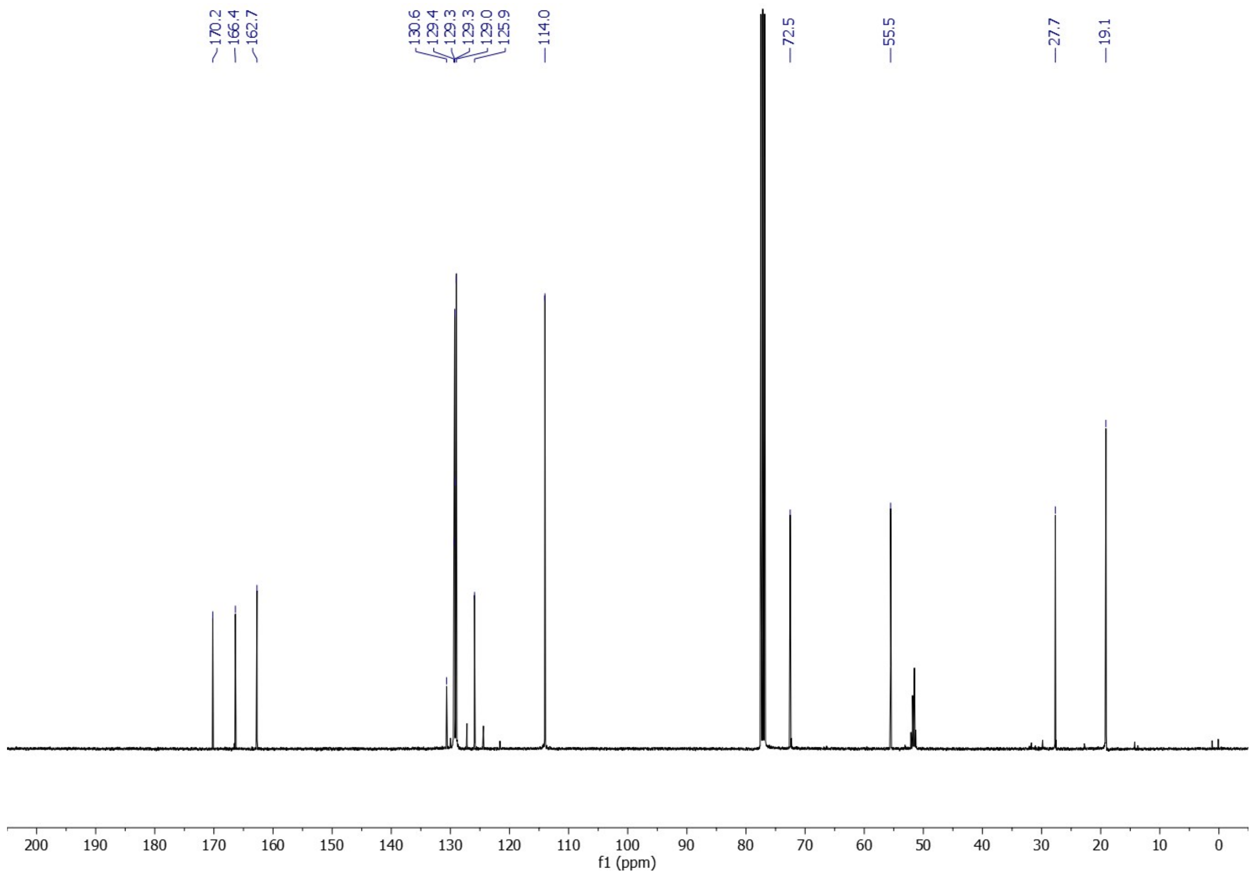
170.2
166.4
162.7
130.6
129.4
129.3
129.3
129.0
125.9
114.0
72.5
55.5
27.7
19.1
200 190 180 170 160 150 140 130 120 110 100 90 80 70 60 50 40 30 20 10 0
f1 (ppm)

**Isobutyl (2*S*,3*R*)-4,4,4-trifluoro-2-(4-methoxybenzamido)-3-phenylbutanoate *syn*-4s**.

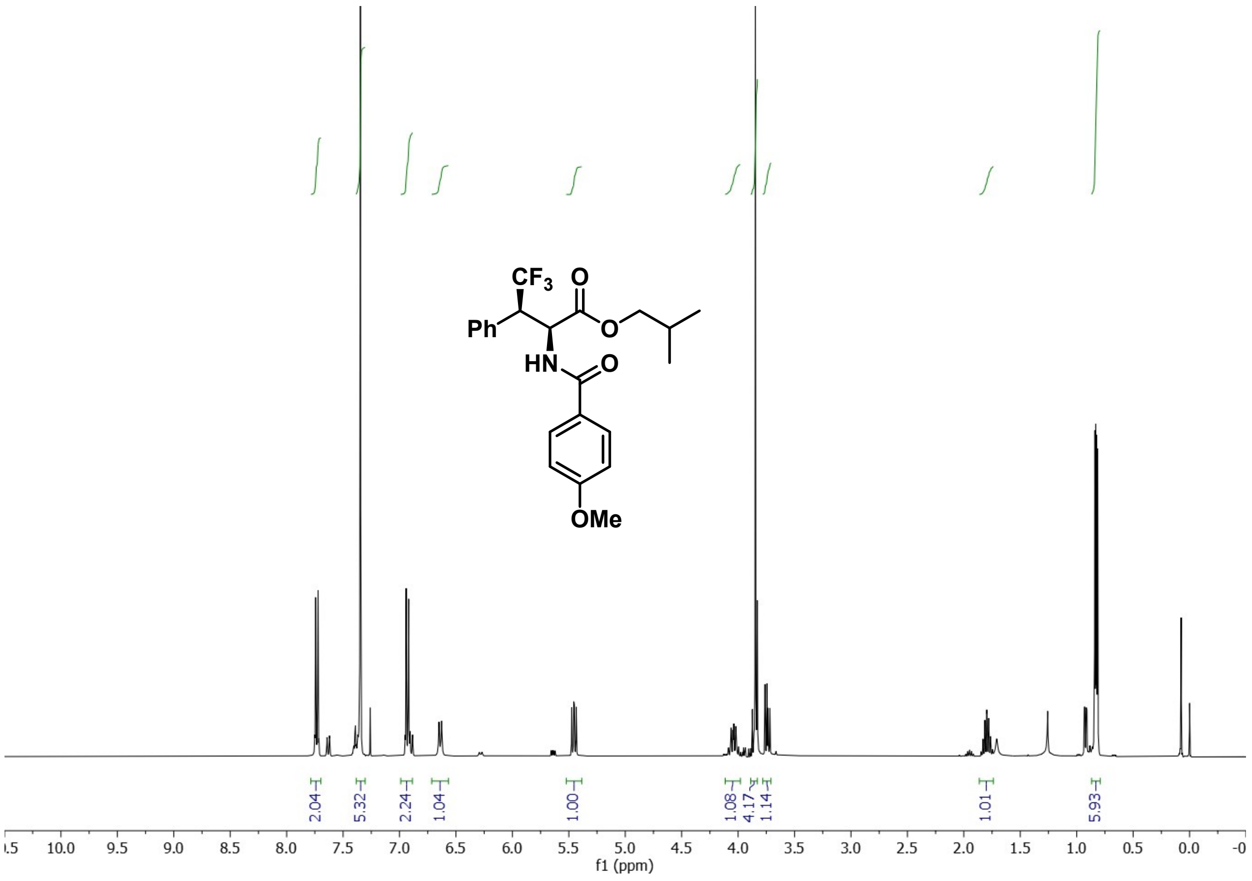


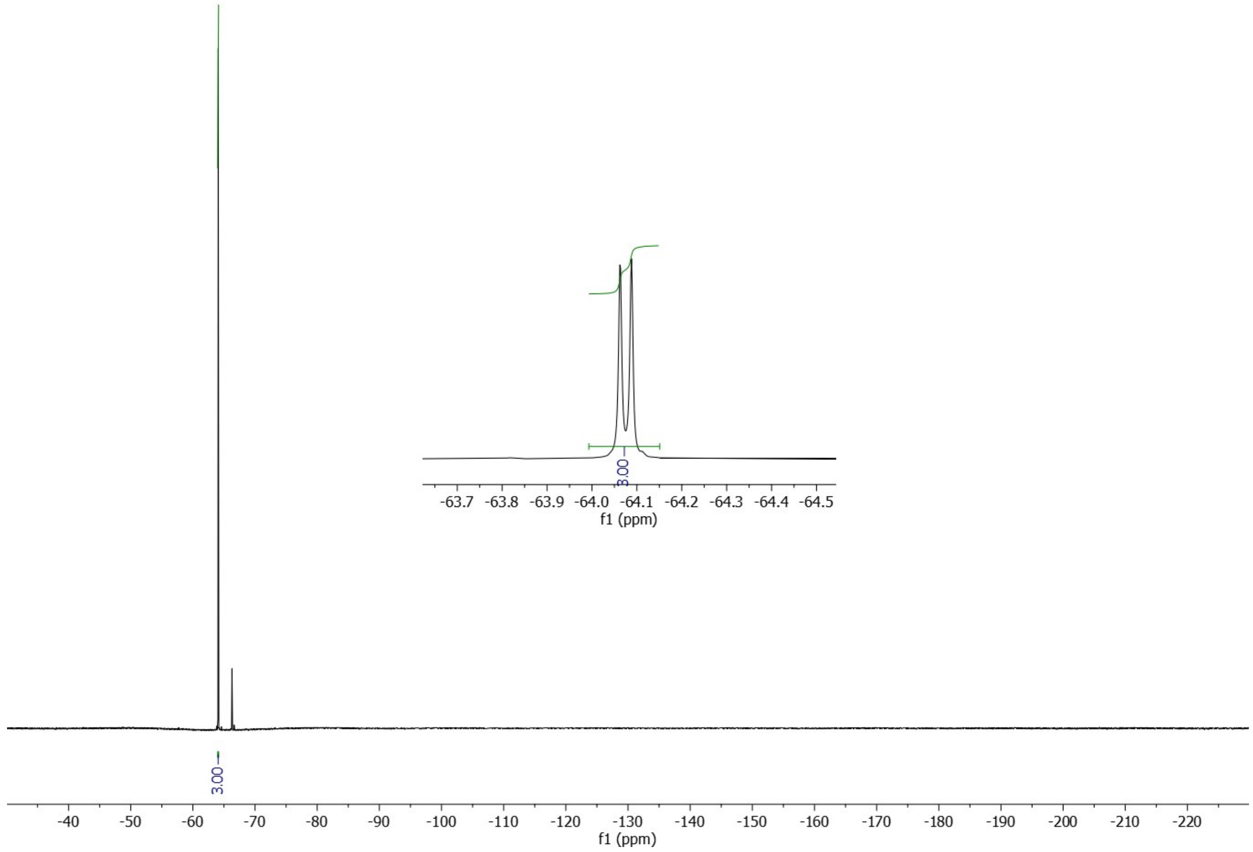

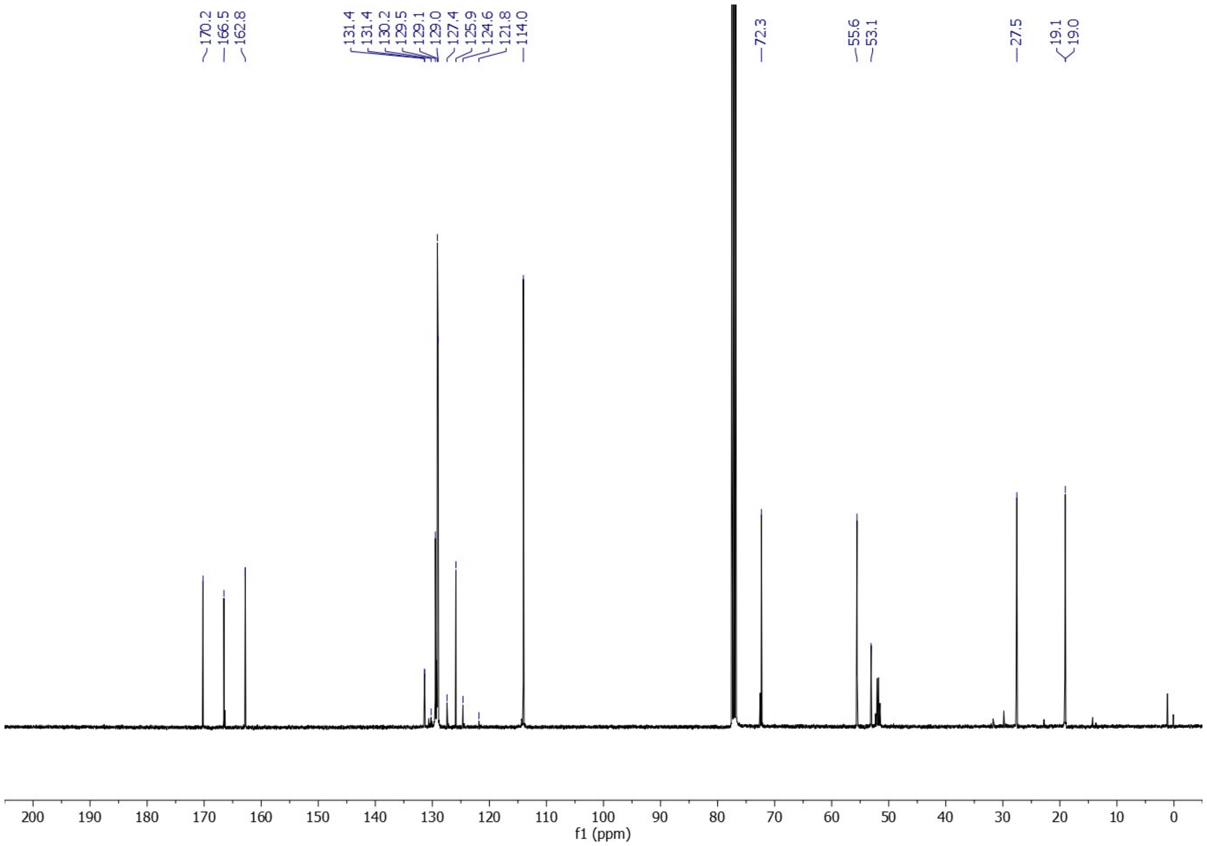
170.2
166.5
162.8
131.4
131.4
130.2
129.5
129.1
129.0
127.4
125.9
124.6
121.8
114.0
72.3
55.6
53.1
27.5
19.1
19.0
200 190 180 170 160 150 140 130 120 110 100 90 80 70 60 50 40 30 20 10 0
f1 (ppm)

***N*-((2*R*,3*R*)-3-(3,5-difluorophenyl)-4,4,4-trifluoro-1-hydroxybutan-2-yl)-4-methoxybenzamide *anti*-5.**

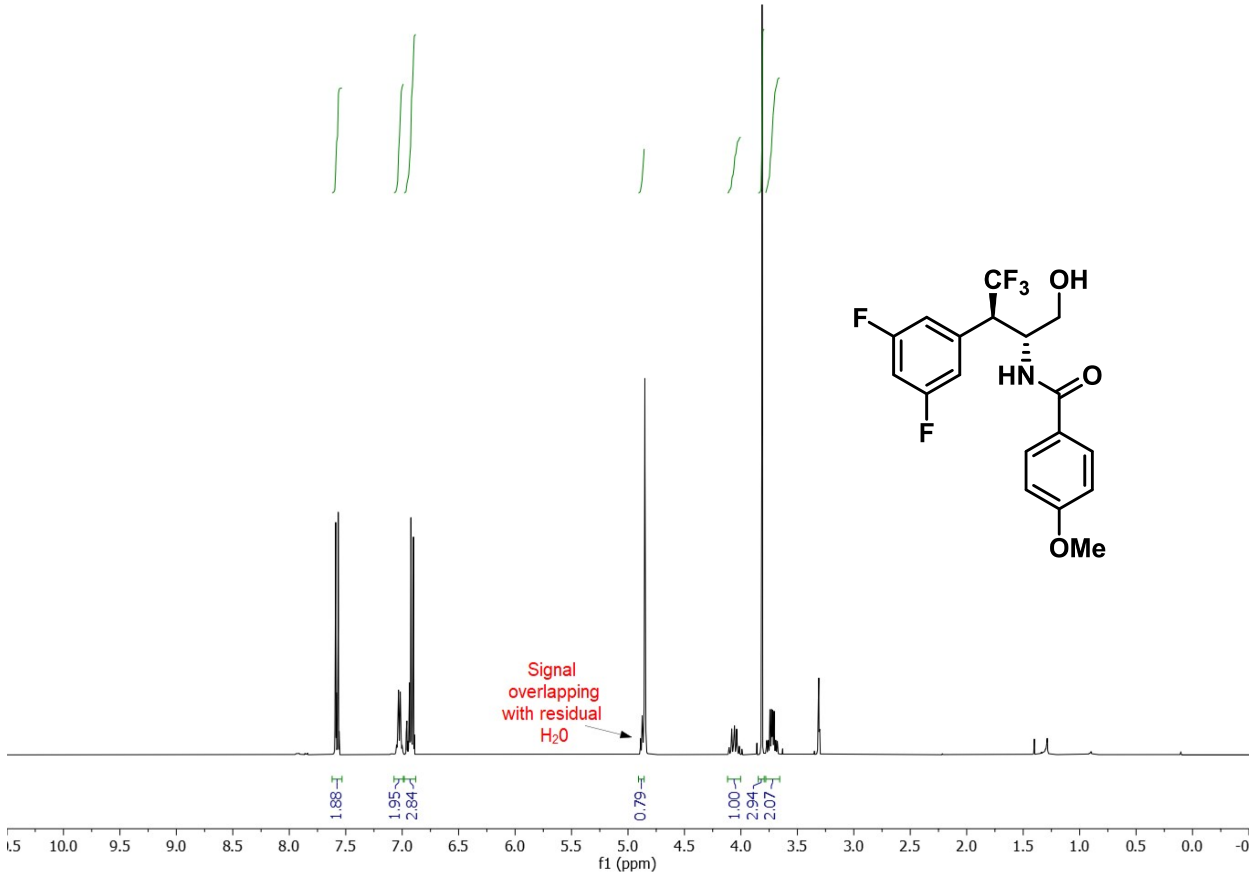

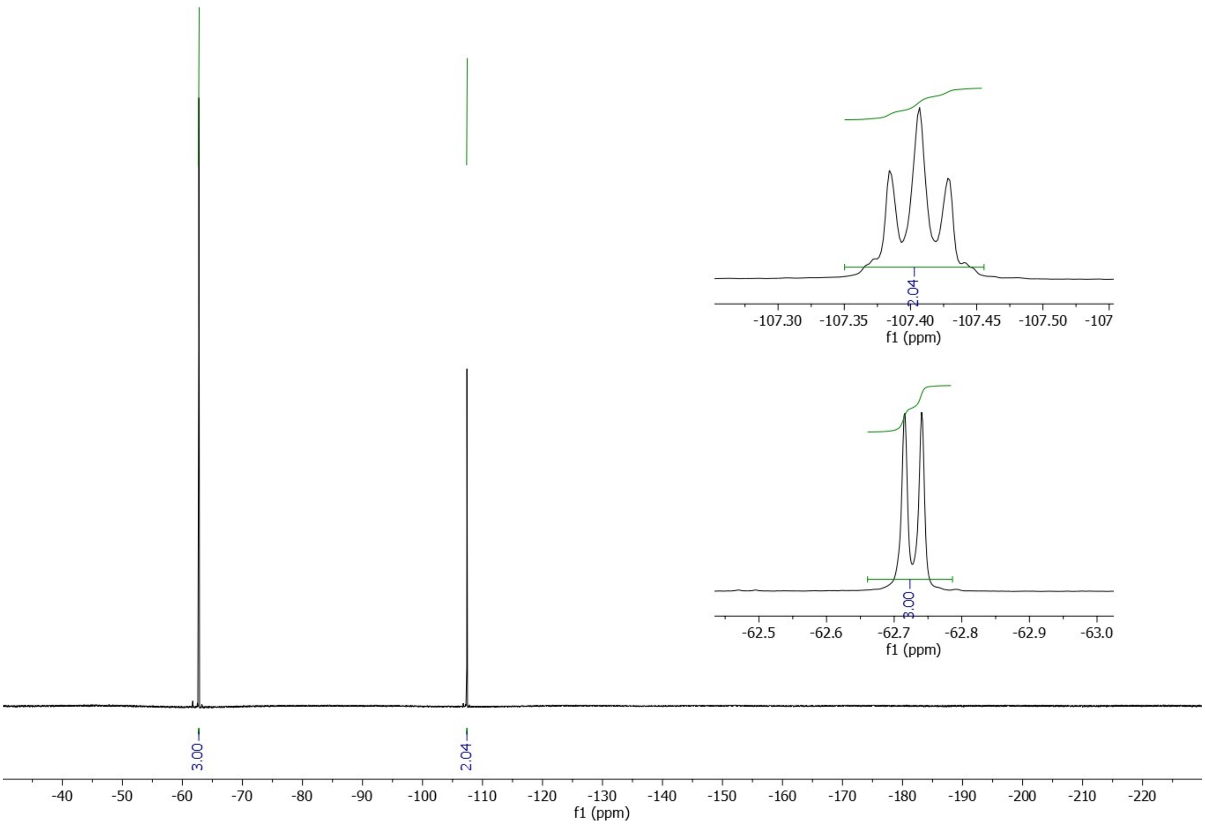

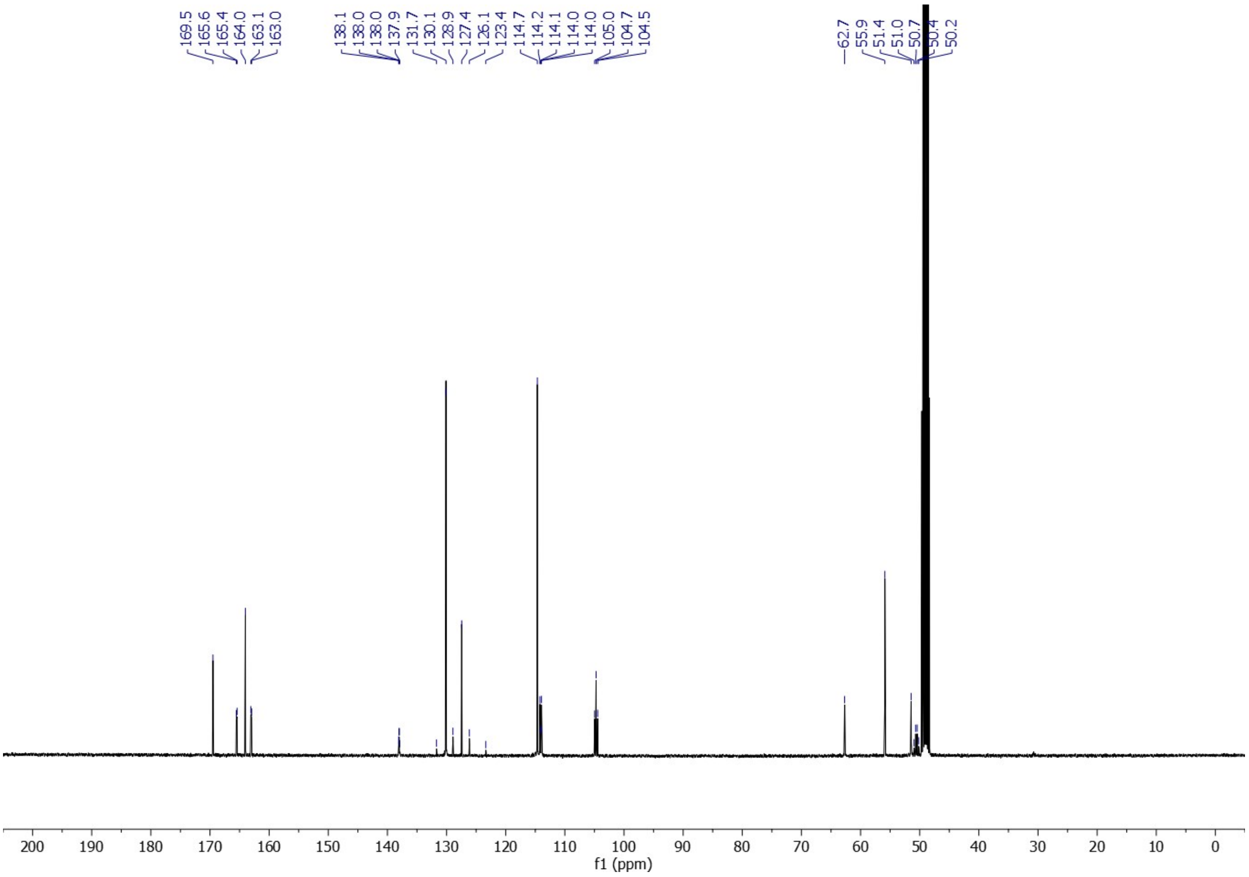

***N*-((2*S*,3*R*)-3-(3,5-difluorophenyl)-4,4,4-trifluoro-1-hydroxybutan-2-yl)-4-methoxybenzamide *syn*-5.**

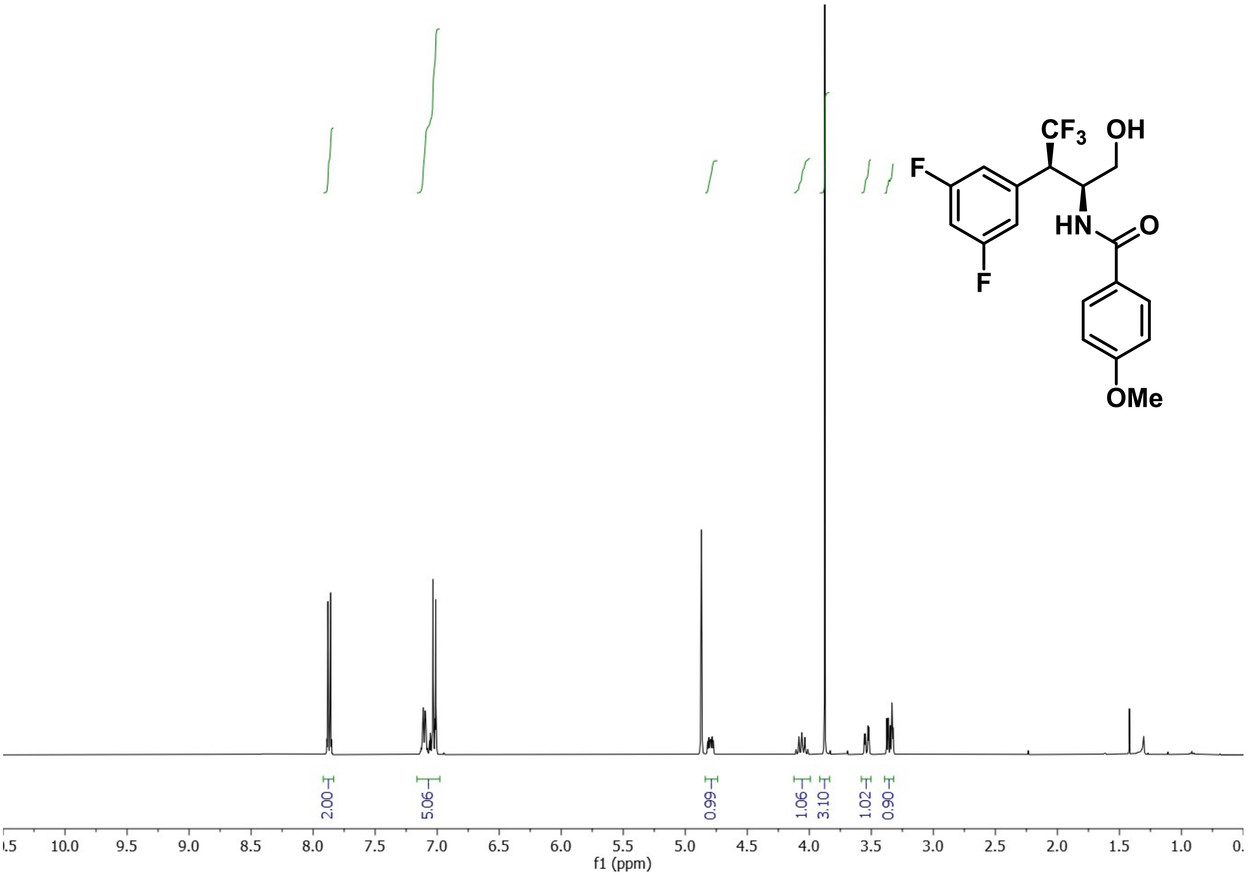

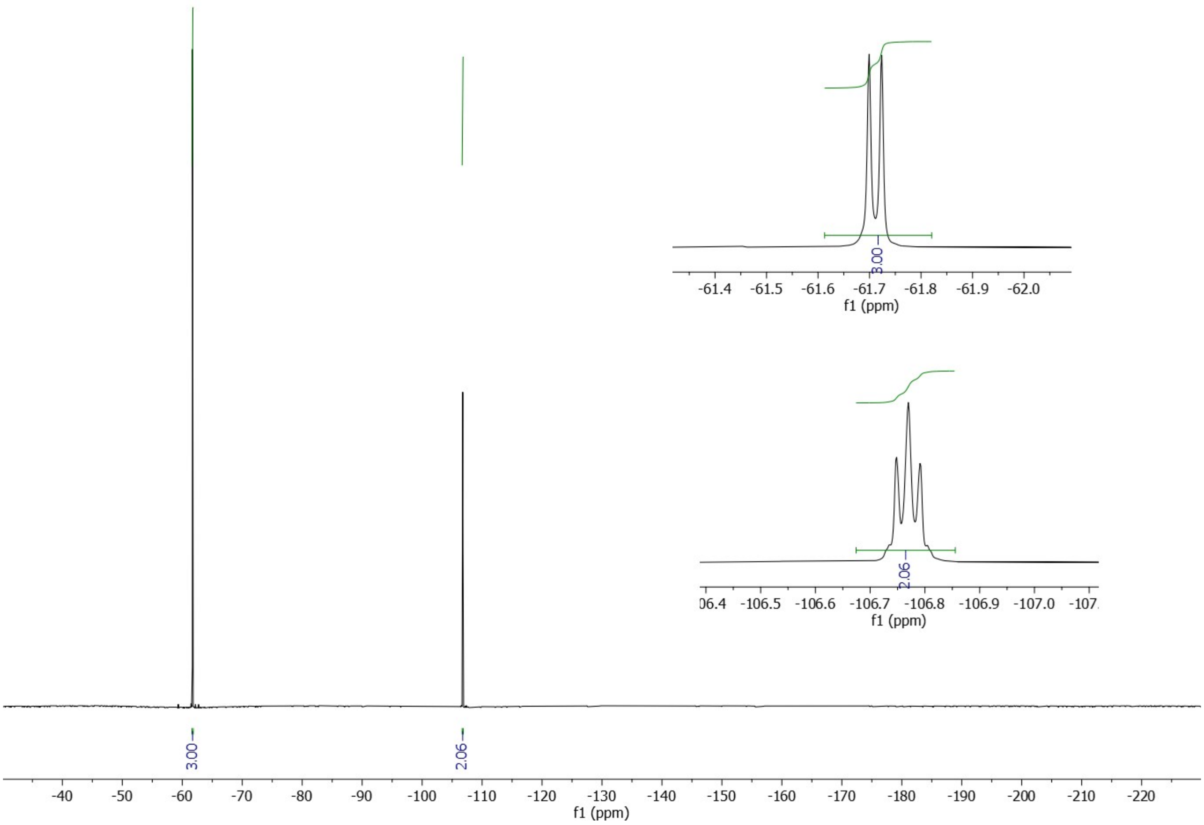

3.00
2.06
-40 -50 -60 -70 -80 -90 -100 -110 -120 -130 -140 -150 -160 -170 -180 -190 -200 -210 -220
f1 (ppm)
3.00
-61.4 -61.5 -61.6 -61.7 -61.8 -61.9 -62.0
f1 (ppm)
2.06
-106.5 -106.6 -106.7 -106.8 -106.9 -107.0
f1 (ppm)


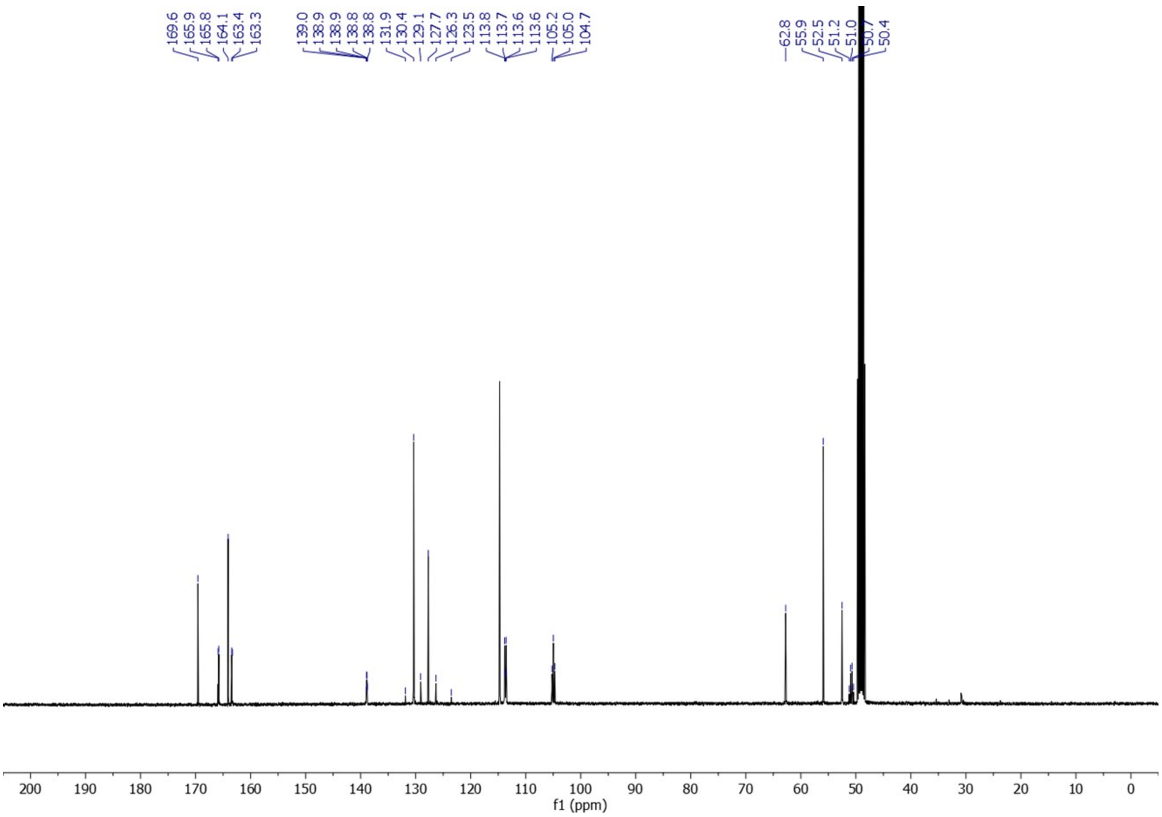

169.6 165.9 165.8 164.1 163.4 163.3
139.0 138.9 138.9 138.8 138.8 131.9 130.4 129.1 127.7 126.3 123.5 113.8 113.7 113.6 113.6 105.2 105.0 104.7
62.8 55.9 52.5 51.2 51.0 50.7 50.4
200 190 180 170 160 150 140 130 120 110 100 90 80 70 60 50 40 30 20 10 0
f1 (ppm)

**(2*R*,3*R*)-3-(3,5-Difluorophenyl)-4,4,4-trifluoro-1-hydroxybutan-2-aminium chloride *anti*-6 (in $CD_3OD$).**

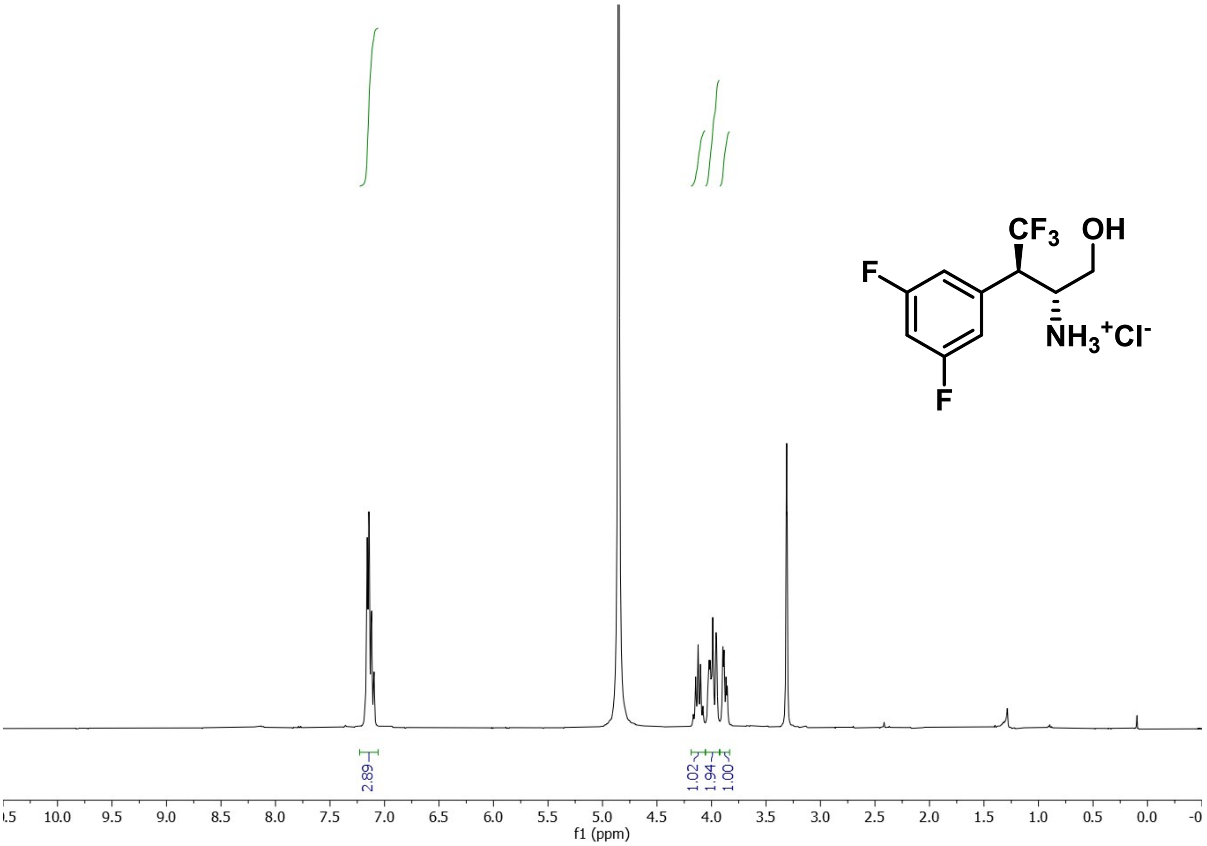

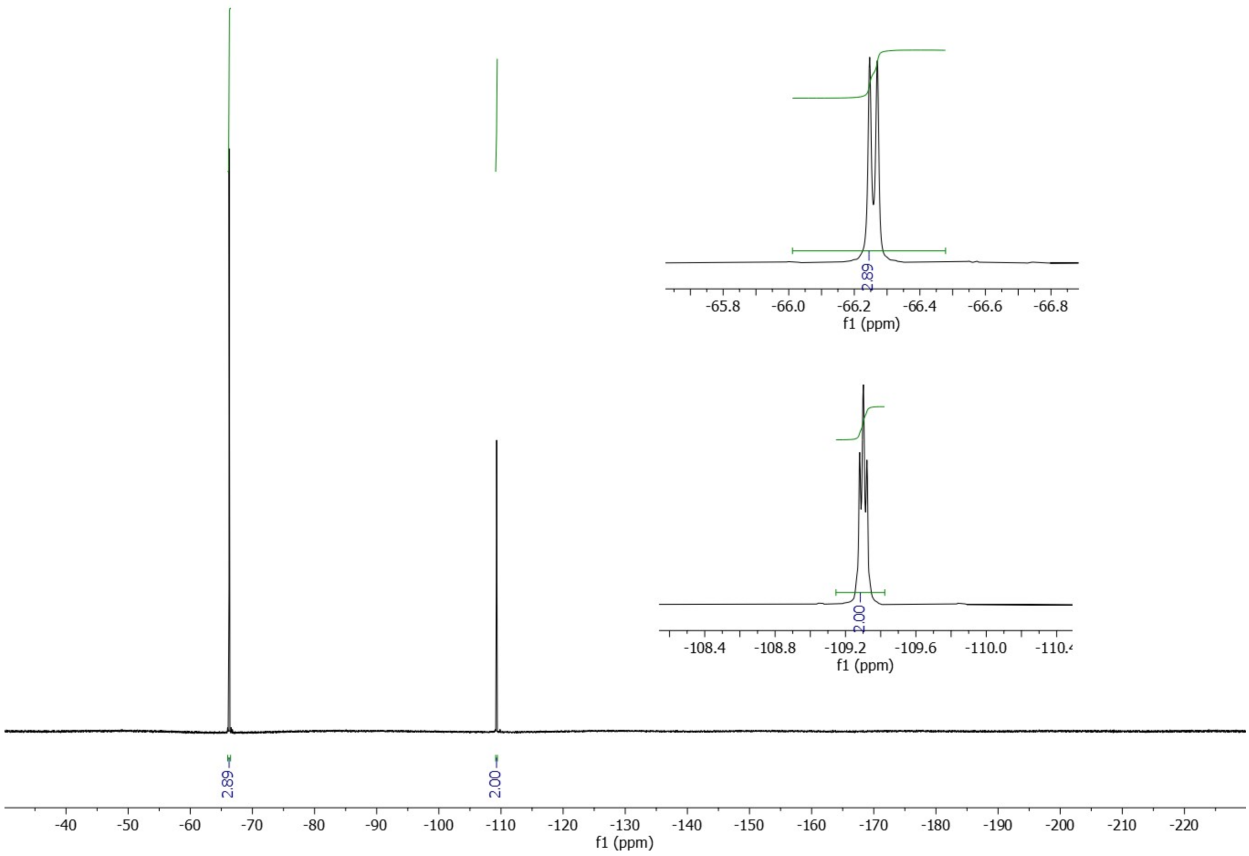
2.89
-65.8
-66.0
-66.2
-66.4
-66.6
-66.8
f1 (ppm)
2.00
-108.4
-108.8
-109.2
-109.6
-110.0
2.89
2.00
-40
-50
-60
-70
-80
-90
-100
-110
-120
-130
-140
-150
-160
-170
-180
-190
-200
-210
-220
f1 (ppm)

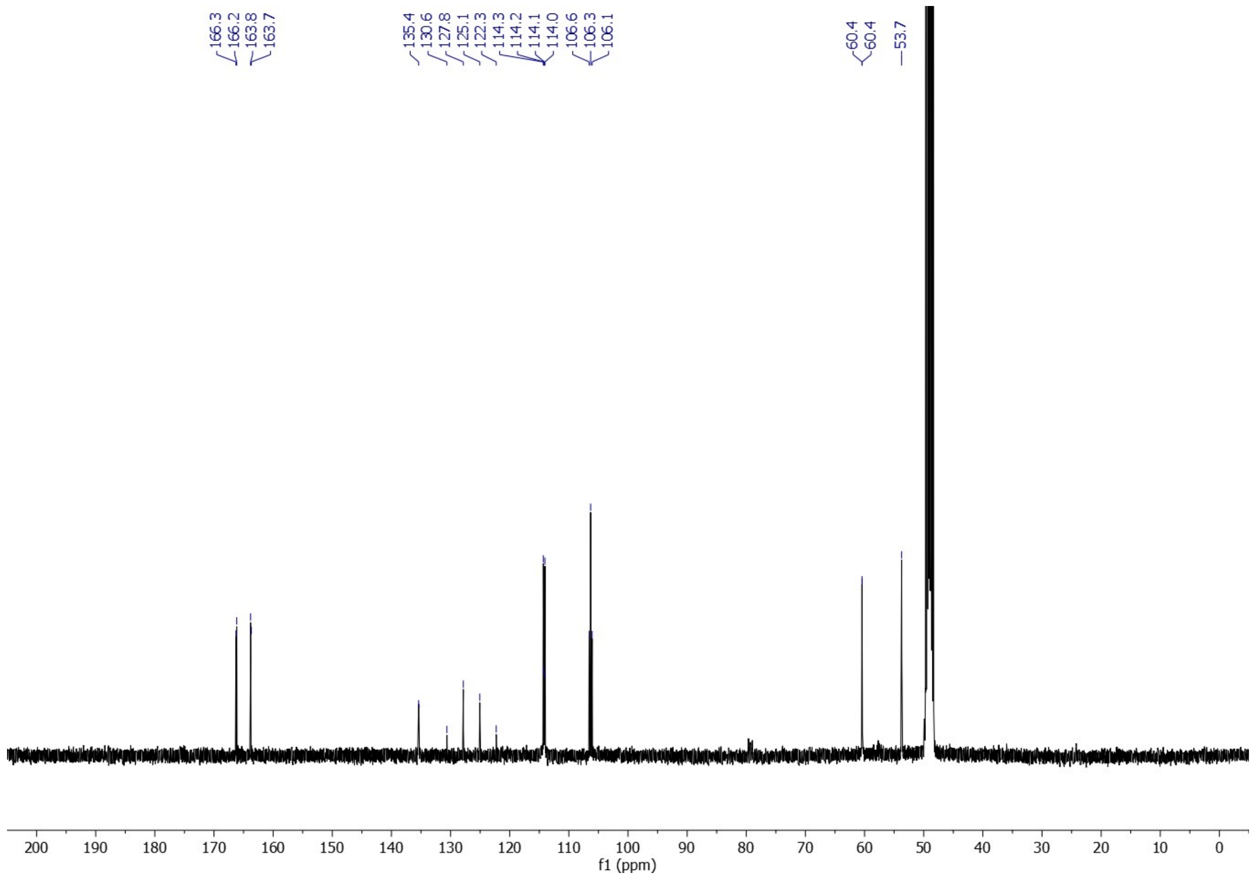
166.3
166.2
163.8
163.7
135.4
130.6
127.8
125.1
122.3
114.3
114.2
114.1
114.0
106.6
106.3
106.1
60.4
60.4
53.7
200
190
180
170
160
150
140
130
120
110
100
90
80
70
60
50
40
30
20
10
0
f1 (ppm)

**(2*S*,3*R*)-3-(3,5-difluorophenyl)-4,4,4-trifluoro-1-hydroxybutan-2-aminium chloride *syn*-6. (in $CD_3OD$).**

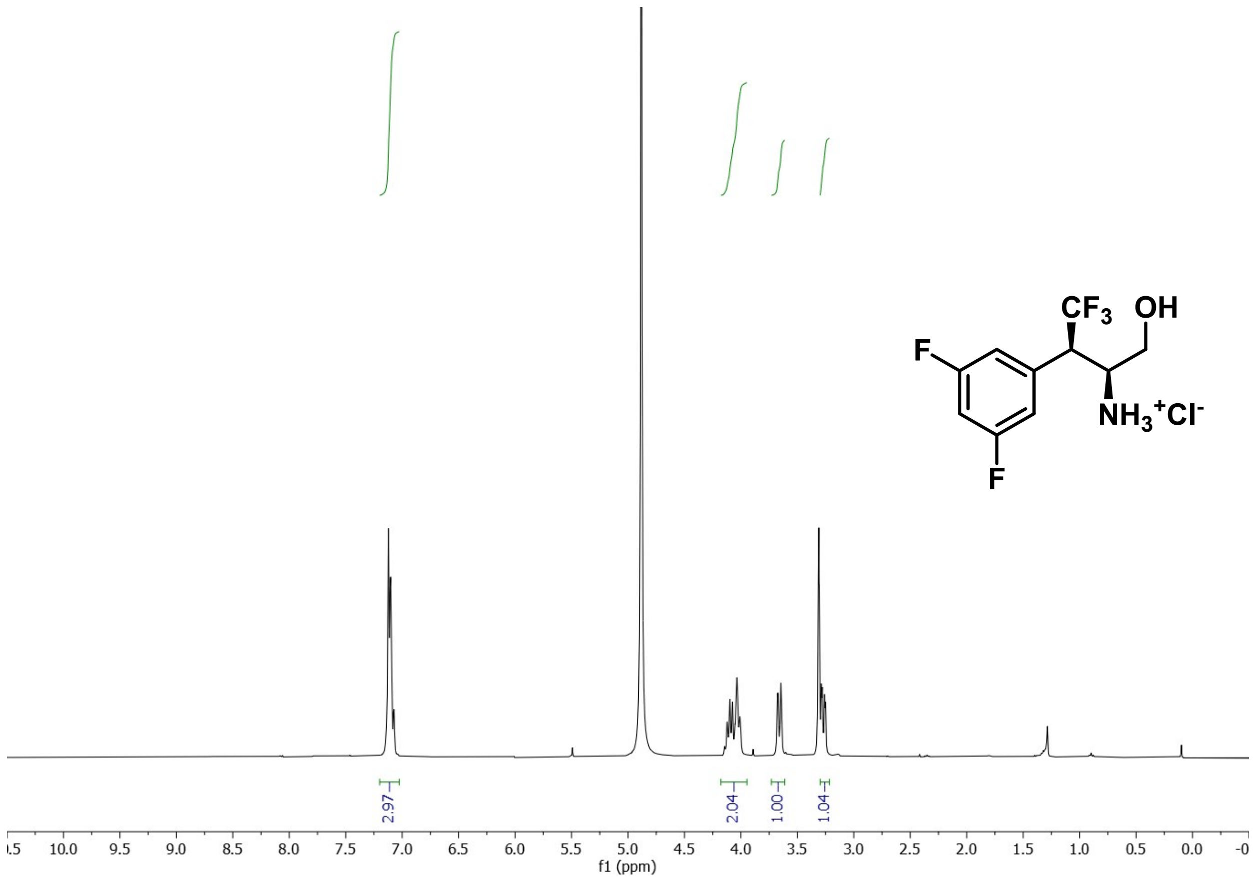

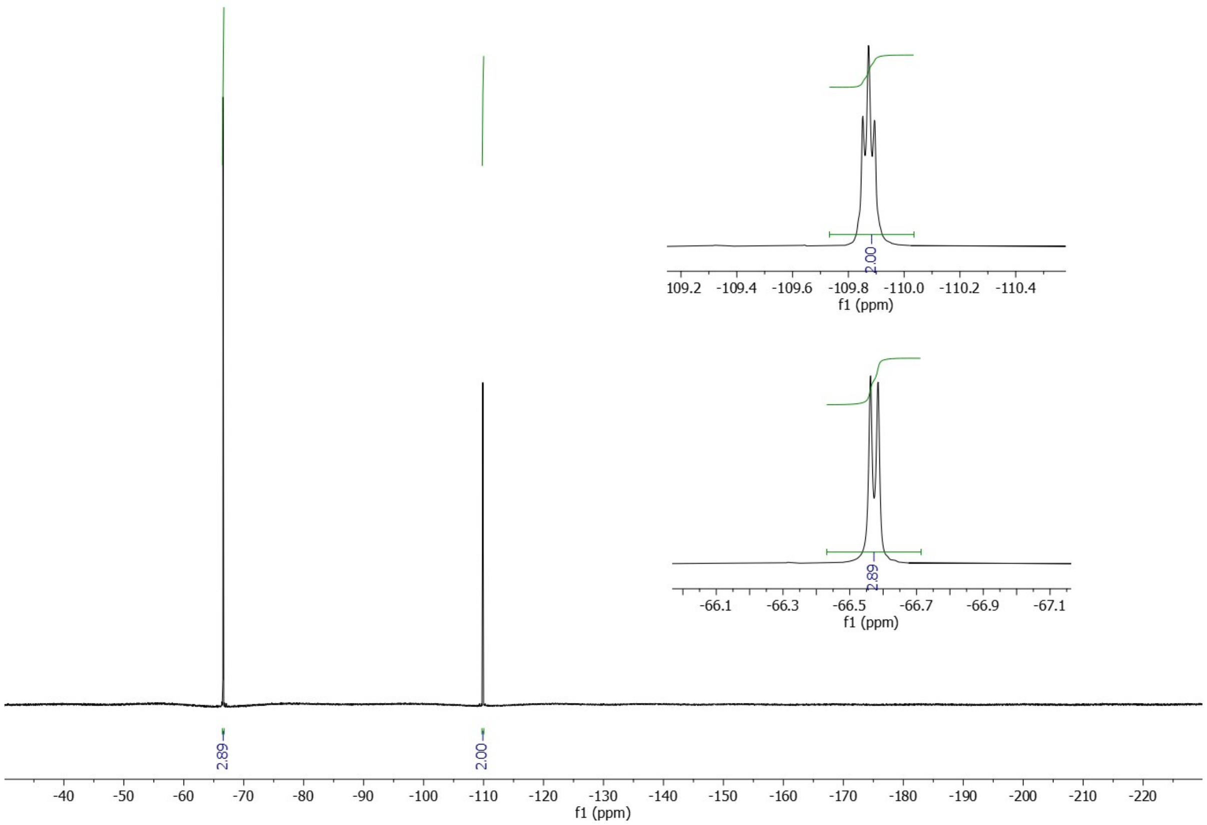

109.2 -109.4 -109.6 -109.8 -110.0 -110.2 -110.4
f1 (ppm)
2.00
-66.1 -66.3 -66.5 -66.7 -66.9 -67.1
f1 (ppm)
2.89
-40 -50 -60 -70 -80 -90 -100 -110 -120 -130 -140 -150 -160 -170 -180 -190 -200 -210 -220
f1 (ppm)


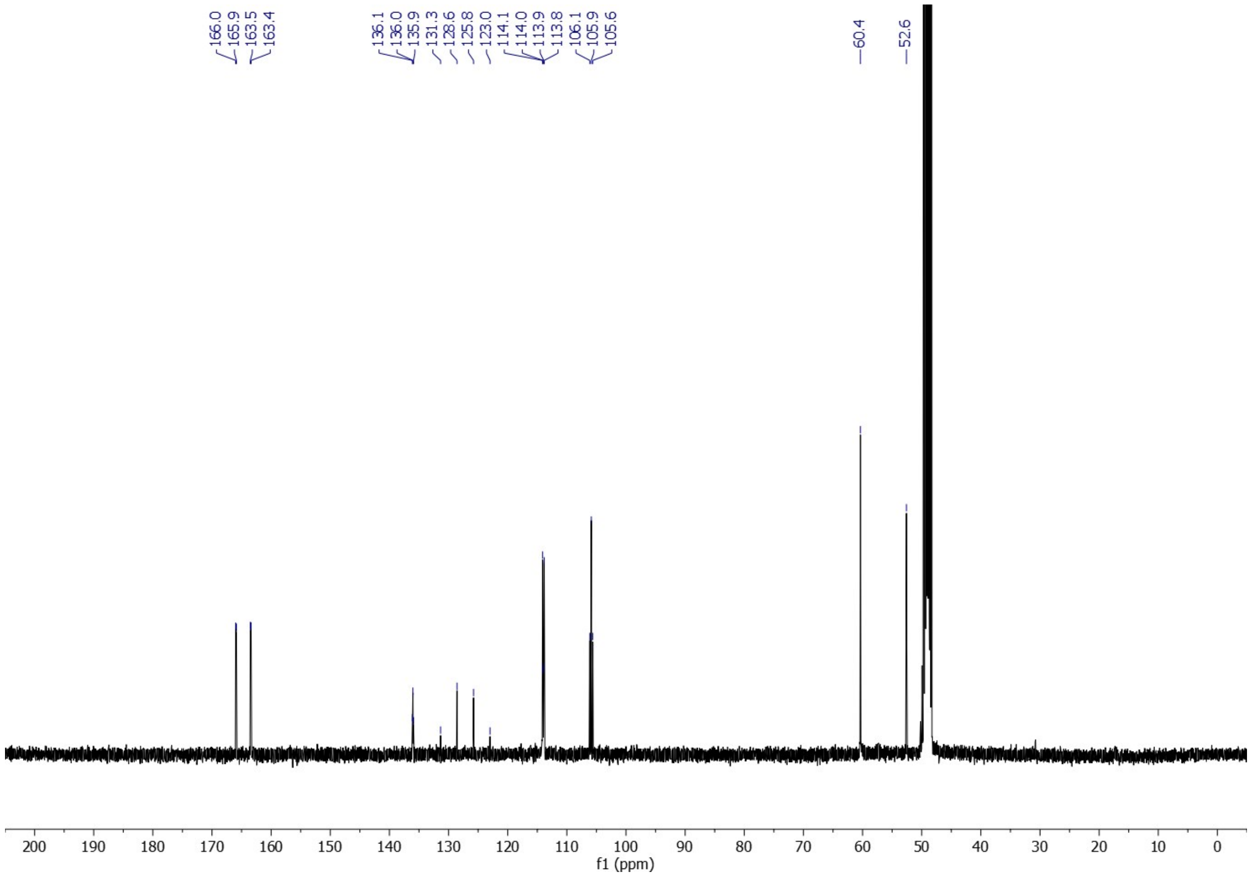

166.0
165.9
163.5
163.4
136.1
136.0
135.9
131.3
128.6
125.8
123.0
114.1
114.0
113.9
113.8
106.1
105.9
105.6
60.4
52.6
200 190 180 170 160 150 140 130 120 110 100 90 80 70 60 50 40 30 20 10 0
f1 (ppm)

## [illegible] Traces of products 4 and 5.

**[illegible]-2-(4-methoxybenzamido)-3-phenylbutanoate *rac*-4d.**

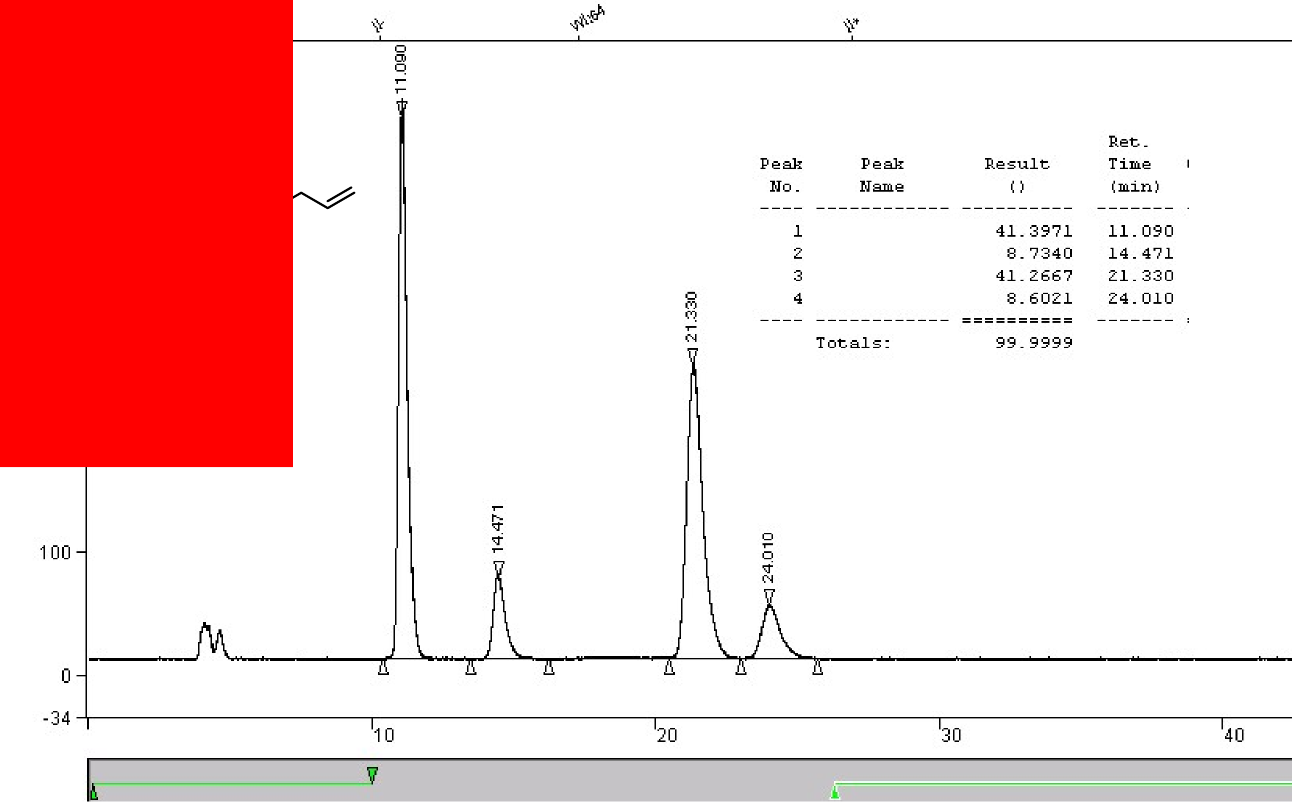


| Peak No. | Peak Name | Result () | Ret. Time (min) |
|---|---|---|---|
| 1 | | 41.3971 | 11.090 |
| 2 | | 8.7340 | 14.471 |
| 3 | | 41.2667 | 21.330 |
| 4 | | 8.6021 | 24.010 |
| | Totals: | 99.9999 | |

**Allyl (2*R*,3*R*)-4,4,4-trifluoro-2-(4-methoxybenzamido)-3-phenylbutanoate *anti*-4d.**

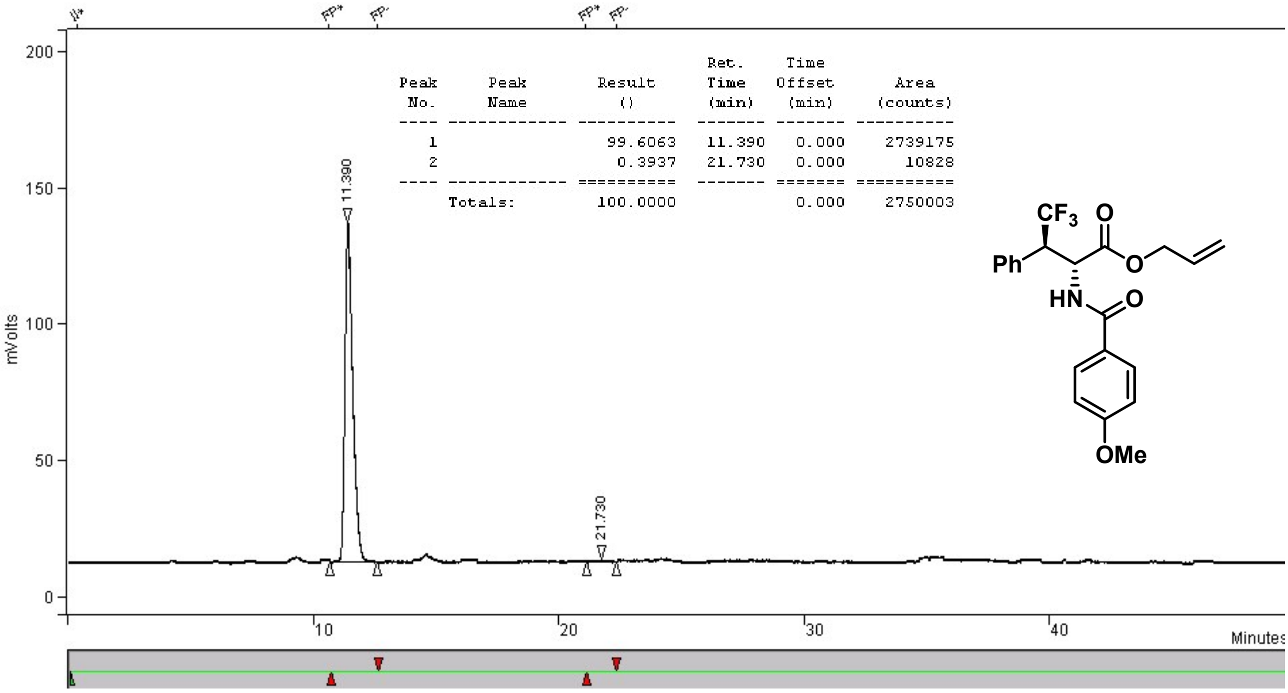


| Peak No. | Peak Name | Result () | Ret. Time (min) | Time Offset (min) | Area (counts) |
|---|---|---|---|---|---|
| 1 | | 99.6063 | 11.390 | 0.000 | 2739175 |
| 2 | | 0.3937 | 21.730 | 0.000 | 10828 |
| | Totals: | 100.0000 | | 0.000 | 2750003 |

**[illegible]rifluoro-2-(4-methoxybenzamido)-3-phenylbutanoate *syn*-4d.**

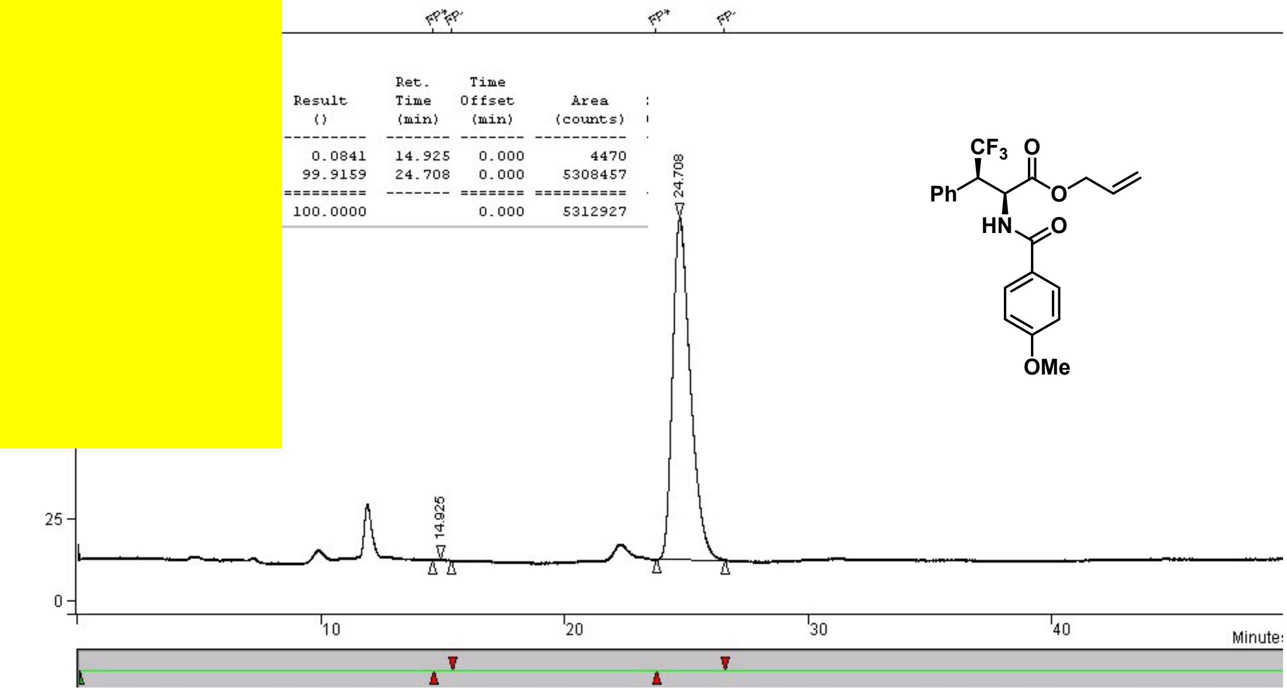


| [illegible] | Result () | Ret. Time (min) | Time Offset (min) | Area (counts) |
|---|---|---|---|---|
| [illegible] | 0.0841 | 14.925 | 0.000 | 4470 |
| [illegible] | 99.9159 | 24.708 | 0.000 | 5308457 |
| [illegible] | 100.0000 | | 0.000 | 5312927 |

**Allyl (2*S*,3*S*)-4,4,4-trifluoro-2-(4-methoxybenzamido)-3-phenylbutanoate *ent-anti*-4d.**

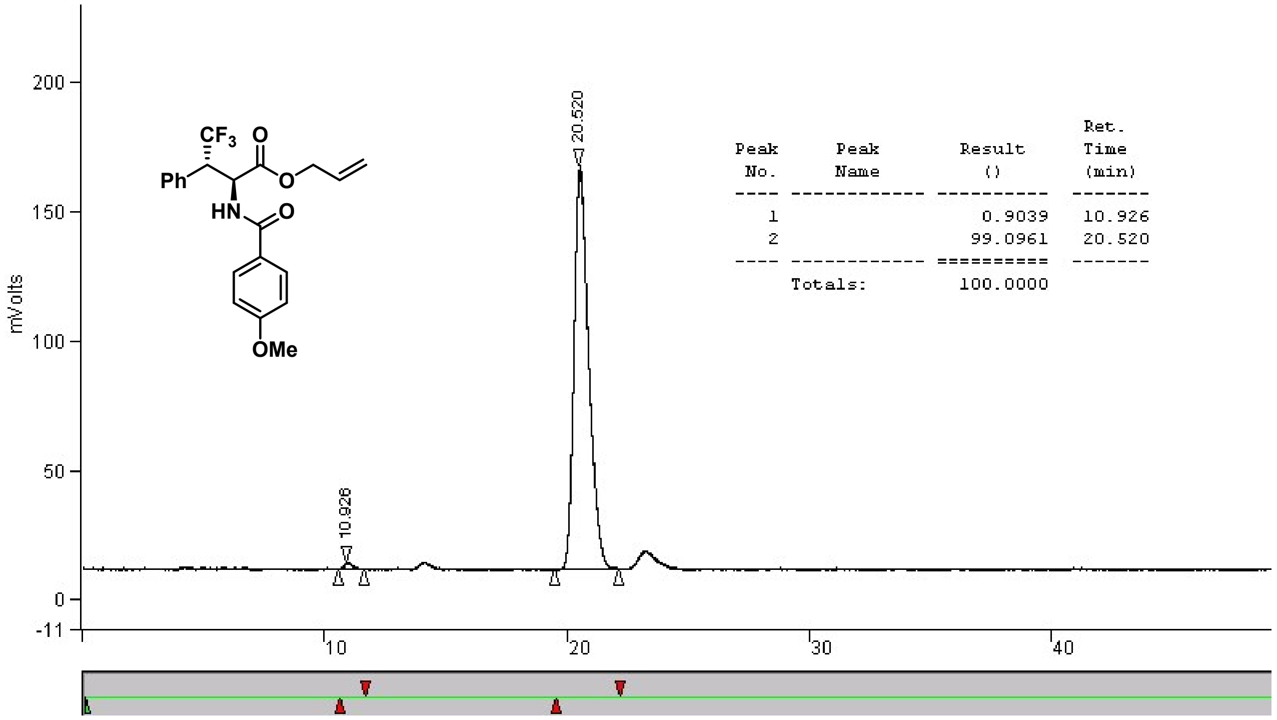


| Peak No. | Peak Name | Result () | Ret. Time (min) |
|---|---|---|---|
| 1 | | 0.9039 | 10.926 |
| 2 | | 99.0961 | 20.520 |
| | Totals: | 100.0000 | |

**Allyl (2*R*,3*S*)-4,4,4-trifluoro-2-(4-methoxybenzamido)-3-phenylbutanoate *ent-syn*-4d.**

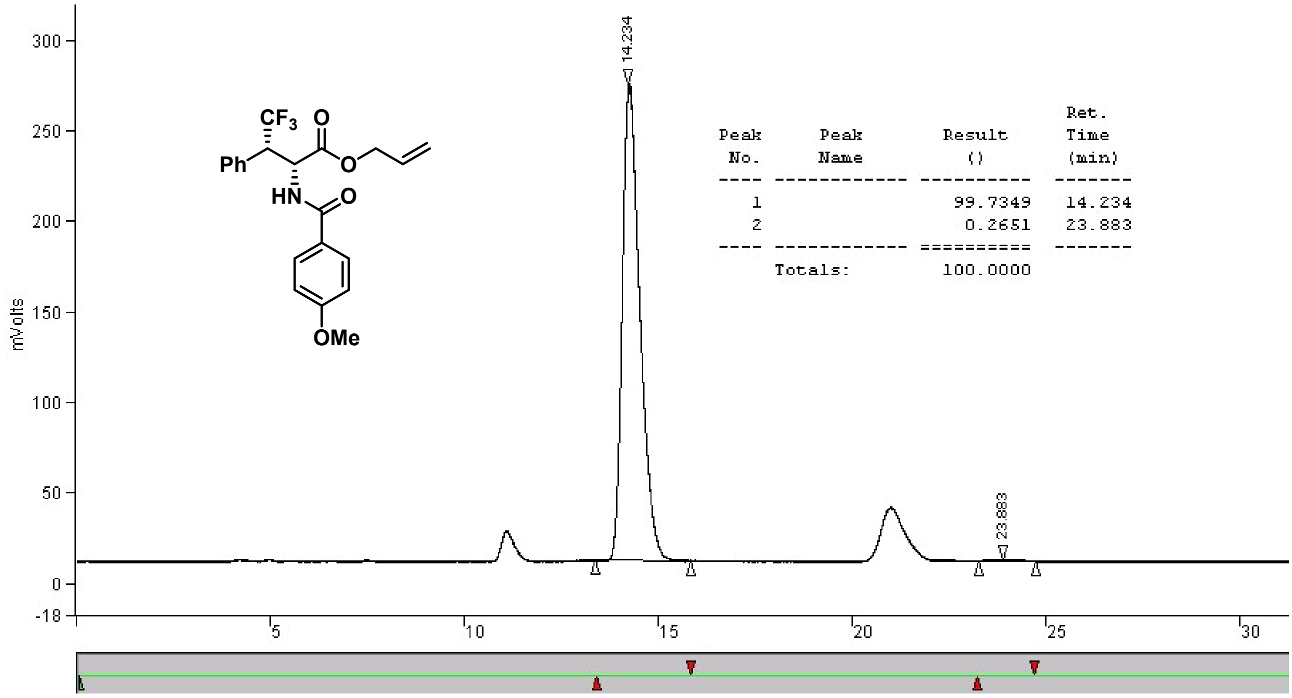


| Peak No. | Peak Name | Result () | Ret. Time (min) |
|---|---|---|---|
| 1 | | 99.7349 | 14.234 |
| 2 | | 0.2651 | 23.883 |
| | Totals: | 100.0000 | |

**Allyl 3-(4-bromophenyl)-4,4,4-trifluoro-2-(4-methoxybenzamido)butanoate *rac*-4e**

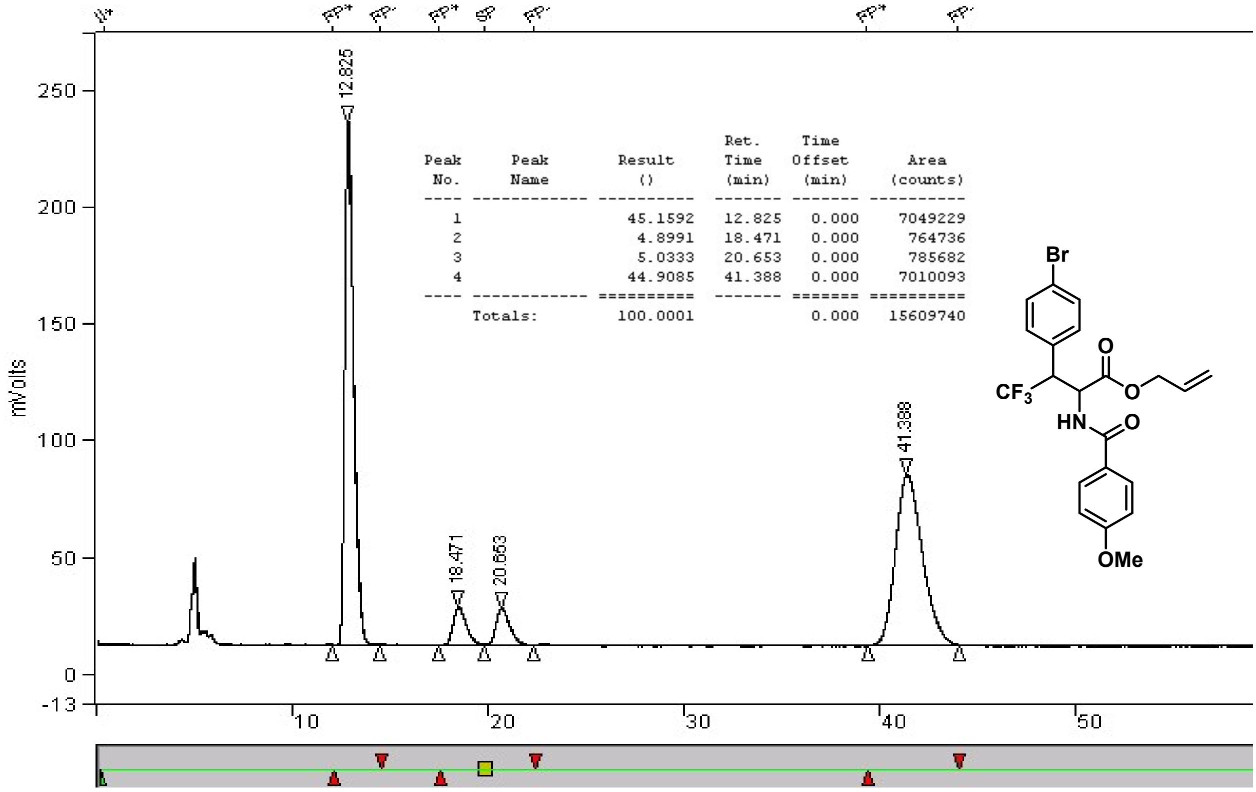


| Peak No. | Peak Name | Result () | Ret. Time (min) | Time Offset (min) | Area (counts) |
|---|---|---|---|---|---|
| 1 | | 45.1592 | 12.825 | 0.000 | 7049229 |
| 2 | | 4.8991 | 18.471 | 0.000 | 764736 |
| 3 | | 5.0333 | 20.653 | 0.000 | 785682 |
| 4 | | 44.9085 | 41.388 | 0.000 | 7010093 |
| | Totals: | 100.0001 | | 0.000 | 15609740 |

**Allyl (2*R*,3*R*)-3-(4-bromophenyl)-4,4,4-trifluoro-2-(4-methoxybenzamido)butanoate *anti*-4e.**

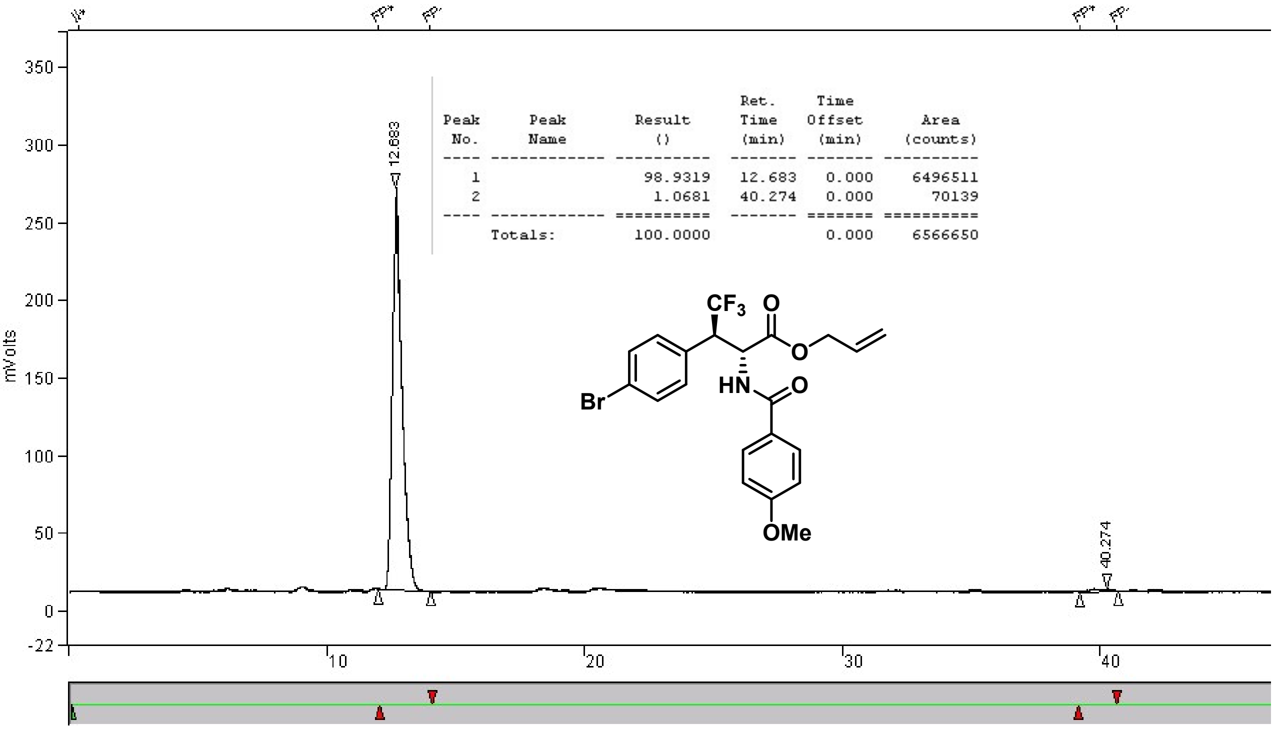


| Peak No. | Peak Name | Result () | Ret. Time (min) | Time Offset (min) | Area (counts) |
|---|---|---|---|---|---|
| 1 | | 98.9319 | 12.683 | 0.000 | 6496511 |
| 2 | | 1.0681 | 40.274 | 0.000 | 70139 |
| | Totals: | 100.0000 | | 0.000 | 6566650 |

**Allyl (2*S*,3*R*)-3-(4-bromophenyl)-4,4,4-trifluoro-2-(4-methoxybenzamido)butanoate *syn*-4e.**

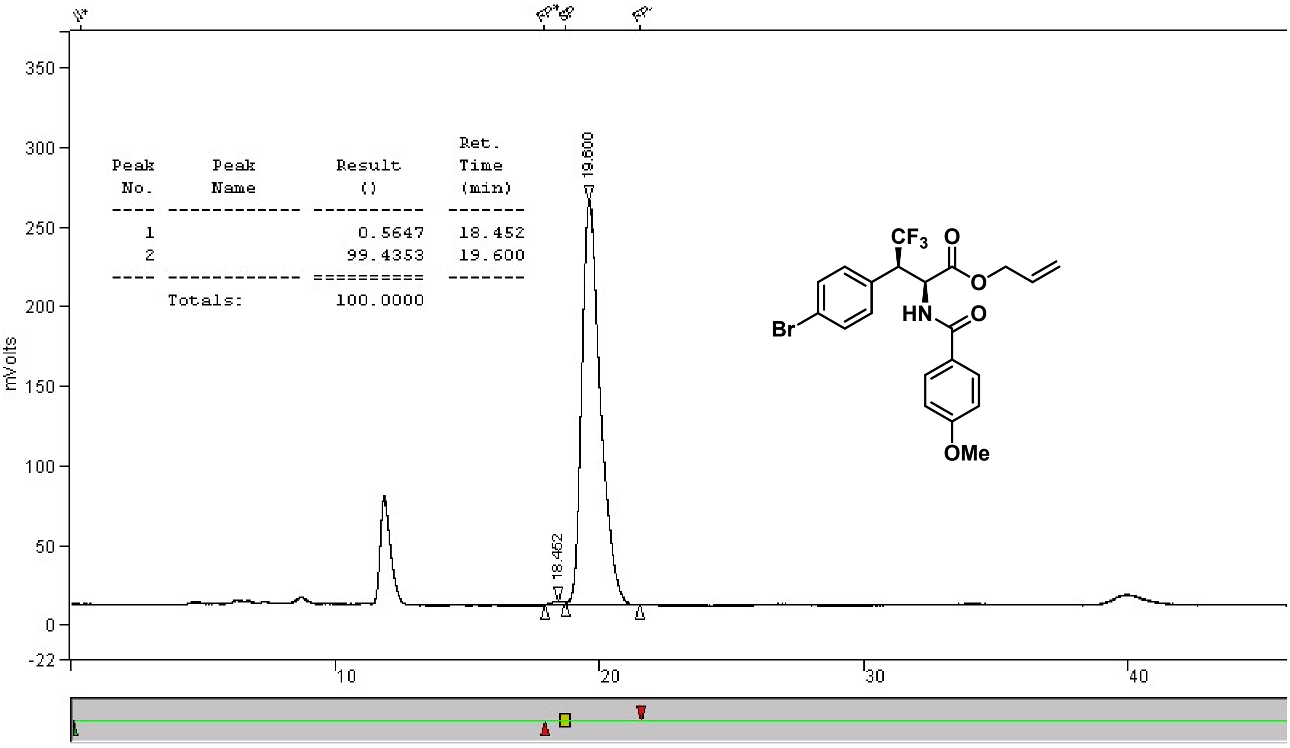


| Peak No. | Peak Name | Result () | Ret. Time (min) |
|---|---|---|---|
| 1 | | 0.5647 | 18.452 |
| 2 | | 99.4353 | 19.600 |
| | Totals: | 100.0000 | |

**Allyl 3-(4-chlorophenyl)-4,4,4-trifluoro-2-(4-methoxybenzamido)butanoate *rac*-4f.**

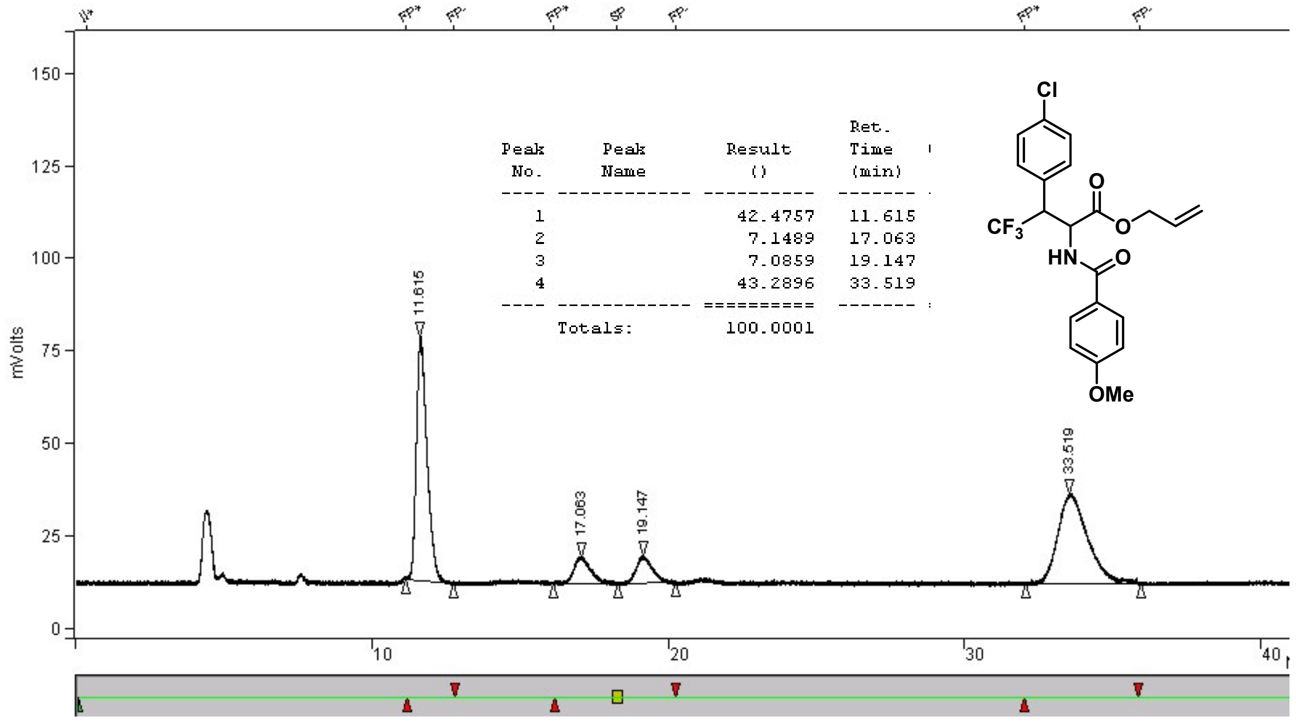


| Peak No. | Peak Name | Result () | Ret. Time (min) |
|---|---|---|---|
| 1 | | 42.4757 | 11.615 |
| 2 | | 7.1489 | 17.063 |
| 3 | | 7.0859 | 19.147 |
| 4 | | 43.2896 | 33.519 |
| | Totals: | 100.0001 | |

**Allyl (2*R*,3*R*)-3-(4-chlorophenyl)-4,4,4-trifluoro-2-(4-methoxybenzamido)butanoate *anti*-4f.**

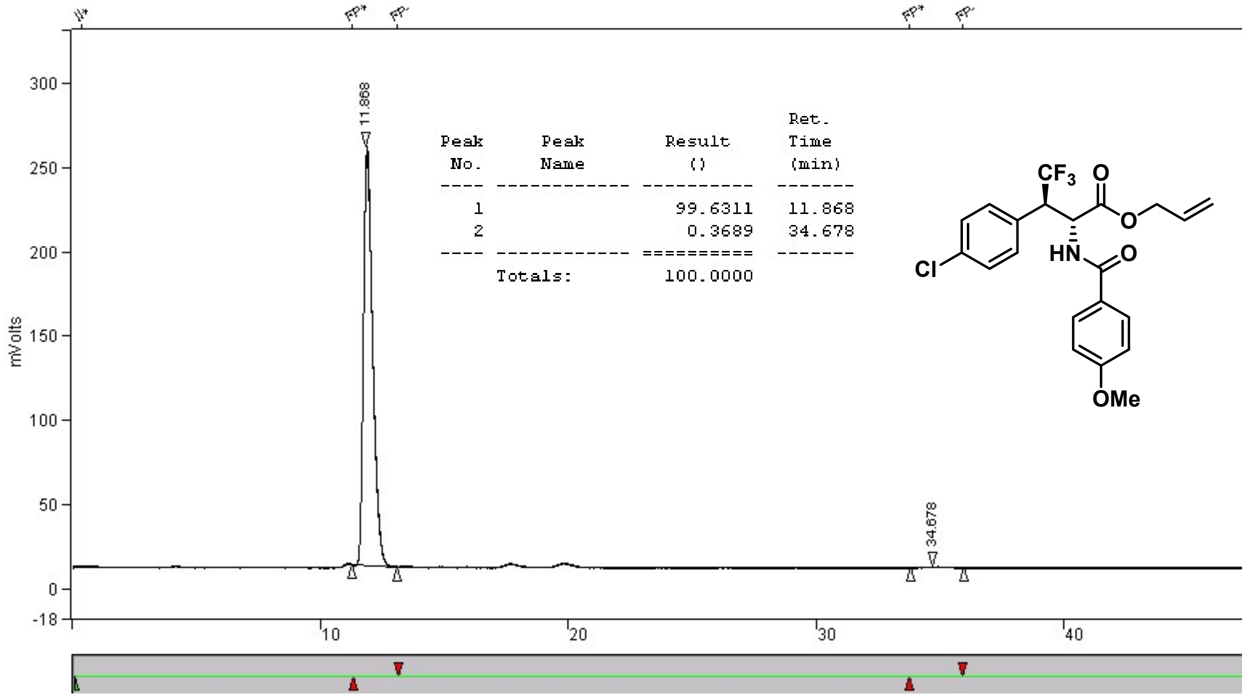


| Peak No. | Peak Name | Result () | Ret. Time (min) |
|---|---|---|---|
| 1 | | 99.6311 | 11.868 |
| 2 | | 0.3689 | 34.678 |
| | Totals: | 100.0000 | |

**Allyl (2*R*,3*R*)-3-(4-chlorophenyl)-4,4,4-trifluoro-2-(4-methoxybenzamido)butanoate *syn*-4f.**

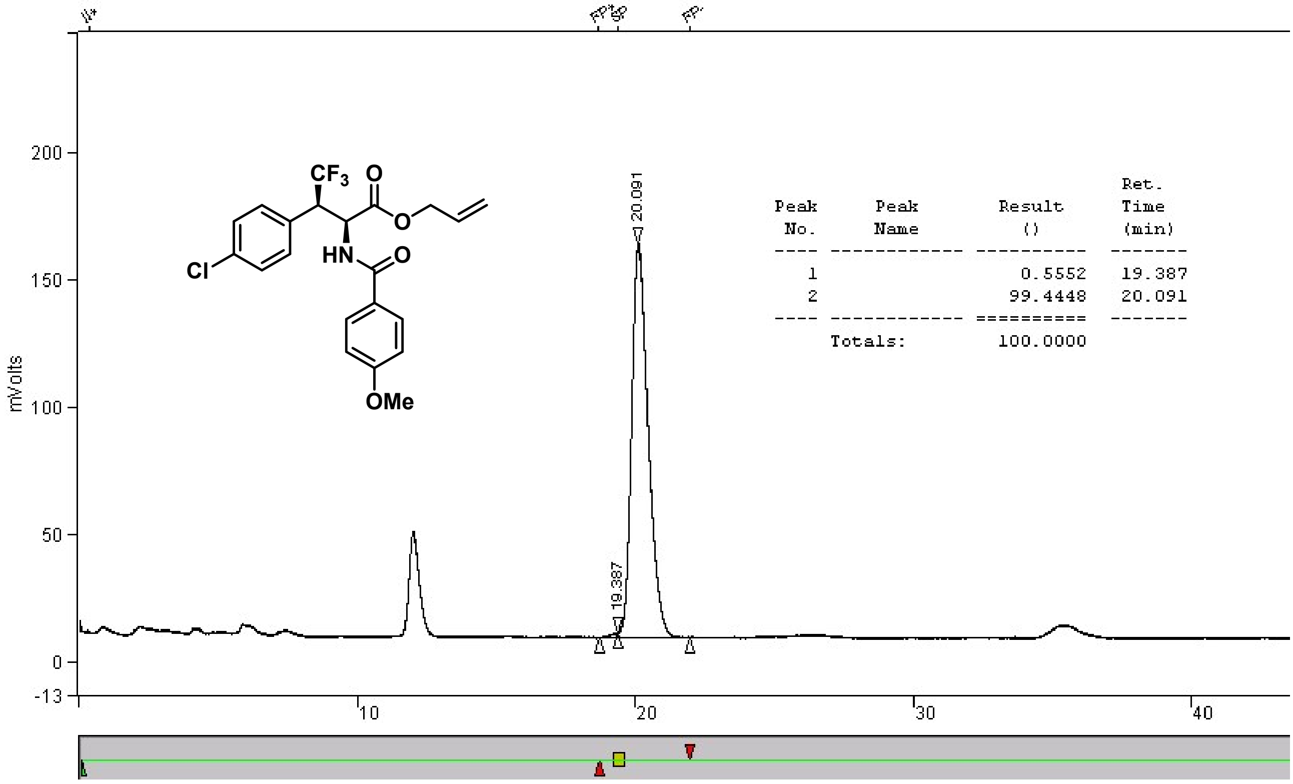


| Peak No. | Peak Name | Result () | Ret. Time (min) |
|---|---|---|---|
| 1 | | 0.5552 | 19.387 |
| 2 | | 99.4448 | 20.091 |
| | Totals: | 100.0000 | |

**Allyl 4,4,4-trifluoro-2-(4-methoxybenzamido)-3-(m-tolyl)butanoate *rac*-4g.**

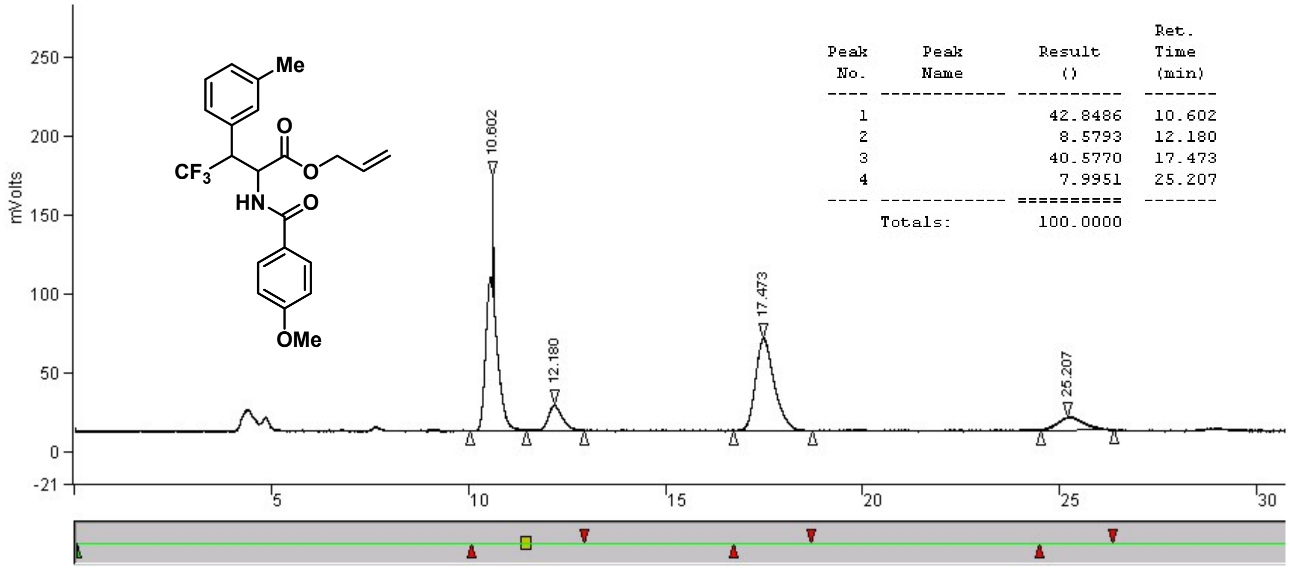


| Peak No. | Peak Name | Result () | Ret. Time (min) |
|---|---|---|---|
| 1 | | 42.8486 | 10.602 |
| 2 | | 8.5793 | 12.180 |
| 3 | | 40.5770 | 17.473 |
| 4 | | 7.9951 | 25.207 |
| | Totals: | 100.0000 | |

**Allyl (2*R*,3*R*)-4,4,4-trifluoro-2-(4-methoxybenzamido)-3-(m-tolyl)butanoate *anti*-4g.**

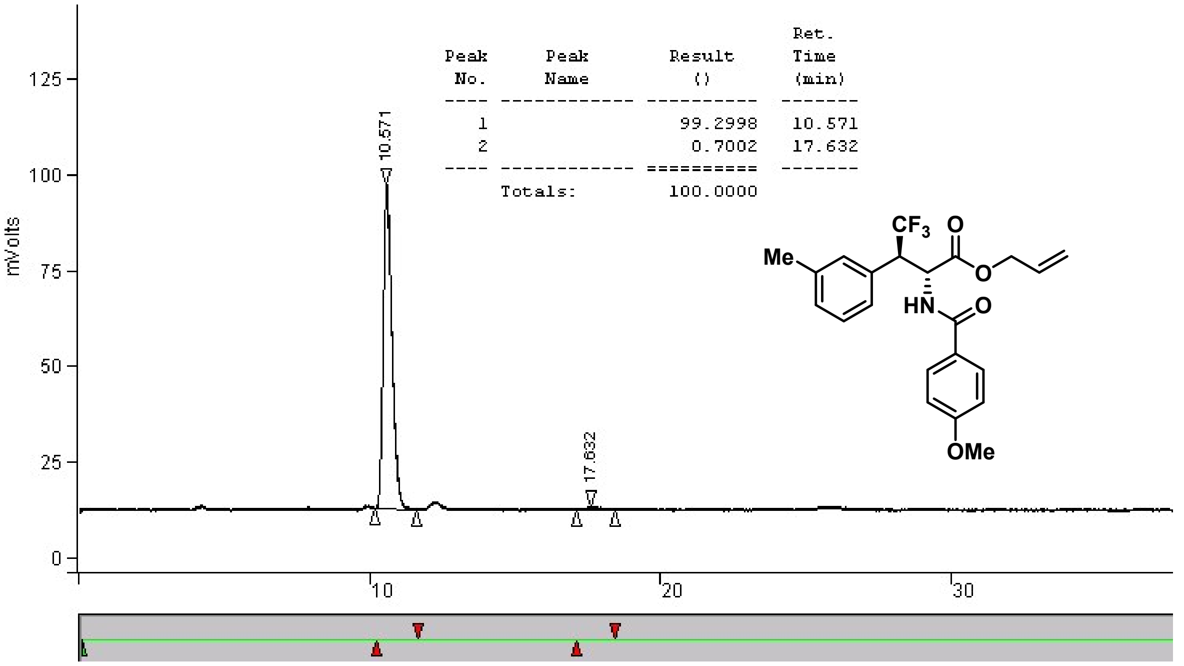


| Peak No. | Peak Name | Result () | Ret. Time (min) |
| --- | --- | --- | --- |
| 1 | | 99.2998 | 10.571 |
| 2 | | 0.7002 | 17.632 |
| | Totals: | 100.0000 | |

**Allyl (2*R*,3*R*)-4,4,4-trifluoro-2-(4-methoxybenzamido)-3-(m-tolyl)butanoate *syn*-4g.**

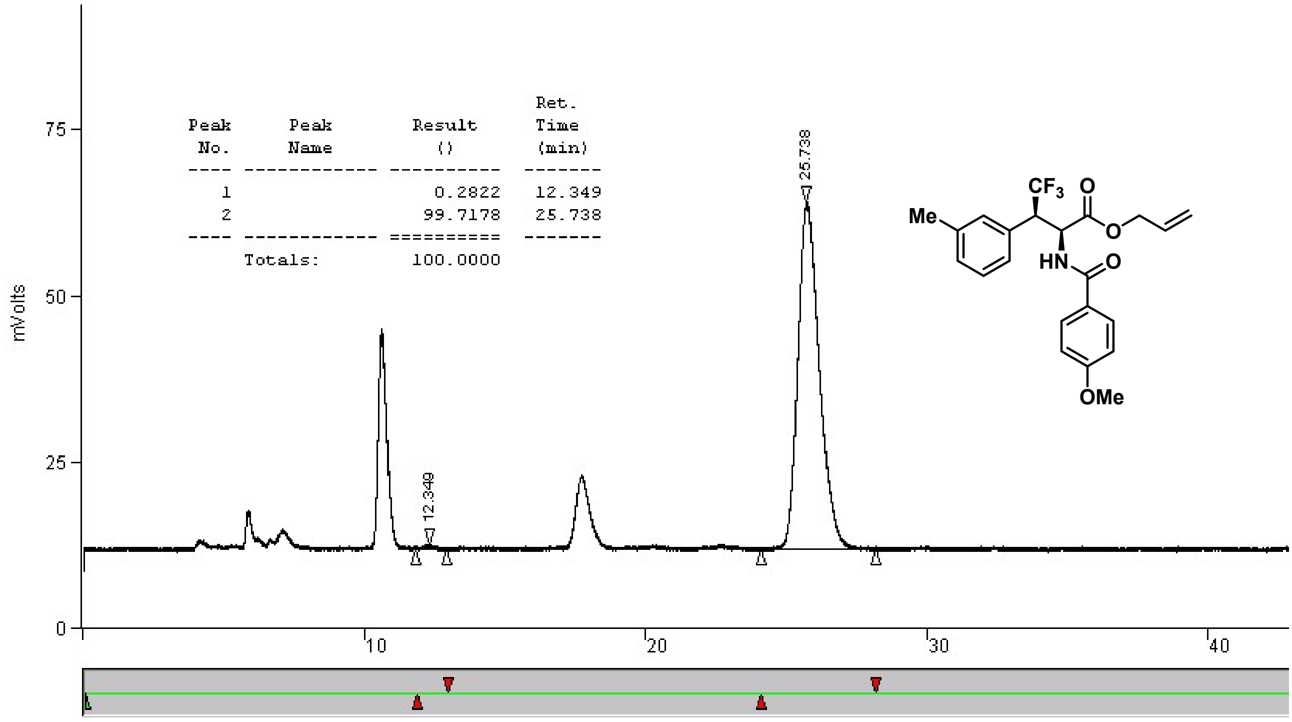


| Peak No. | Peak Name | Result () | Ret. Time (min) |
| --- | --- | --- | --- |
| 1 | | 0.2822 | 12.349 |
| 2 | | 99.7178 | 25.738 |
| | Totals: | 100.0000 | |

**Allyl 4,4,4-trifluoro-3-(4-fluorophenyl)-2-(4-methoxybenzamido)butanoate *rac*-4h.**

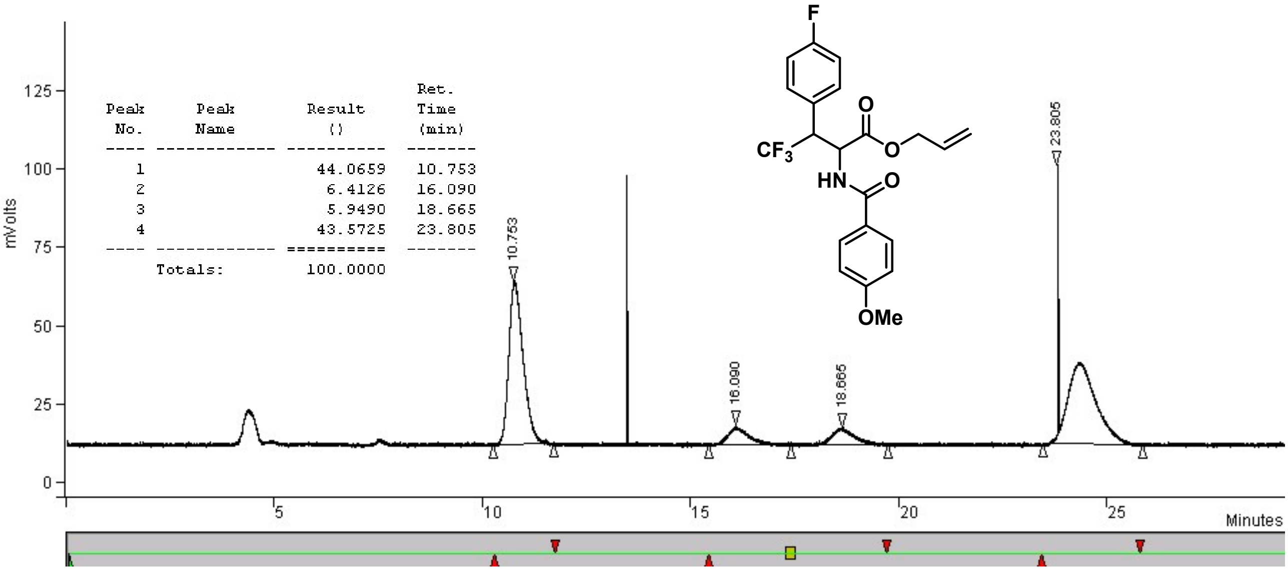


**Allyl (2*R*,3*R*)-4,4,4-trifluoro-3-(4-fluorophenyl)-2-(4-methoxybenzamido)butanoate *anti*-4h.**

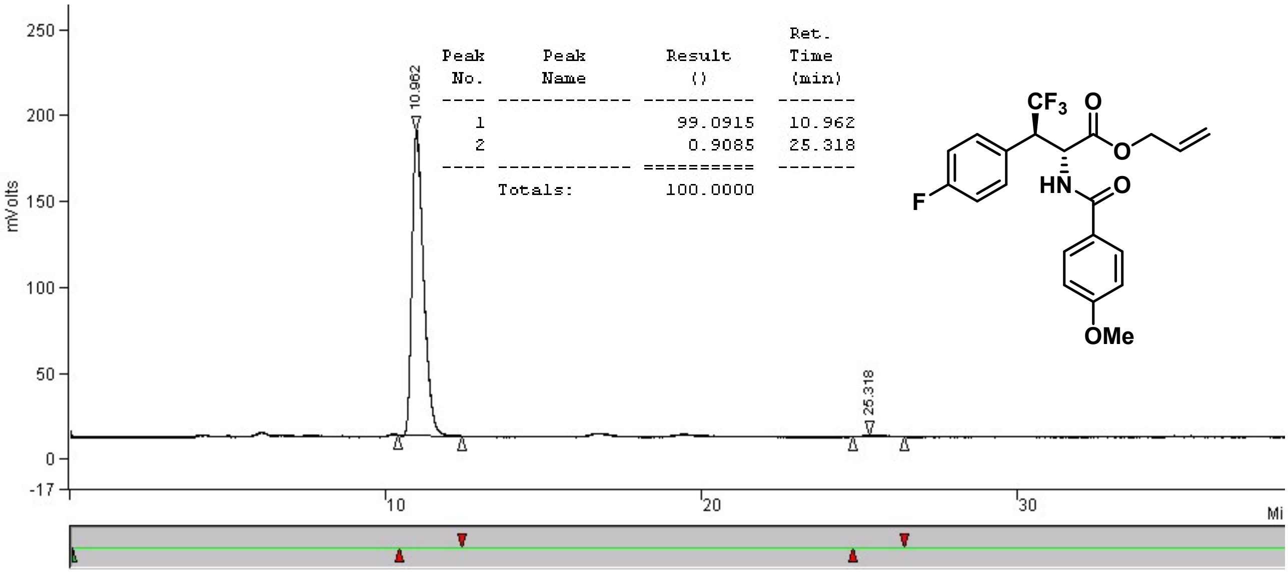

**Allyl (2*R*,3*R*)-4,4,4-trifluoro-3-(4-fluorophenyl)-2-(4-methoxybenzamido)butanoate *syn*-4h.**

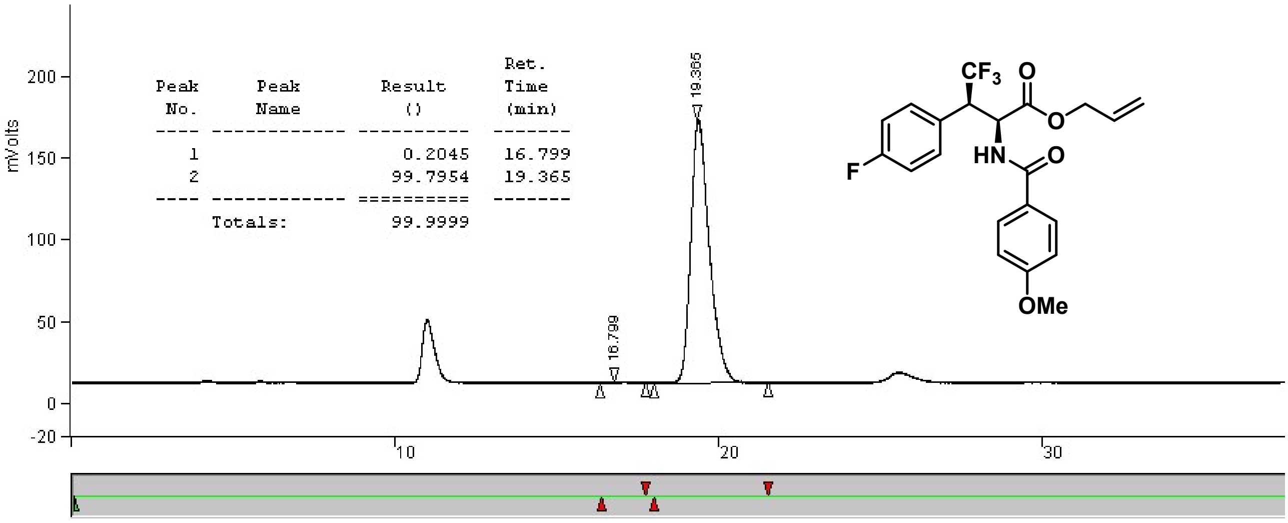


**Allyl (2*R*,3*R*)-4,4,4-trifluoro-2-(4-methoxybenzamido)-3-(4-methoxyphenyl)butanoate *rac*-4i.**

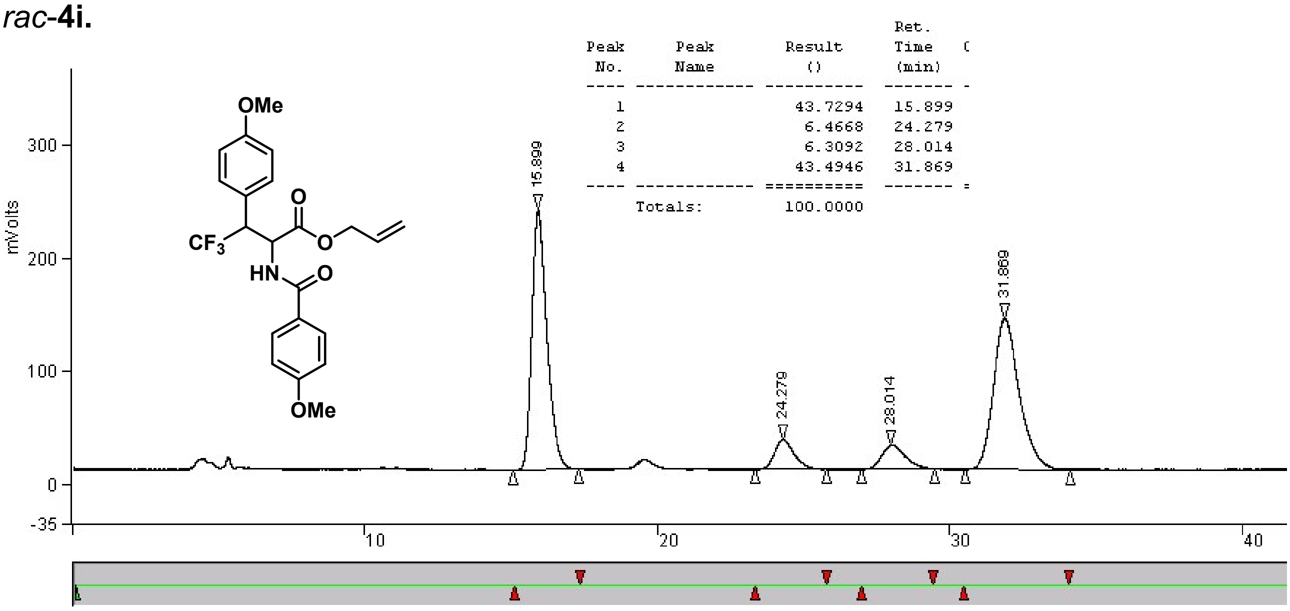

**Allyl(2*R*,3*R*)-4,4,4-trifluoro-2-(4-methoxybenzamido)-3-(4-methoxyphenyl)butanoate** *anti*-**4i**.

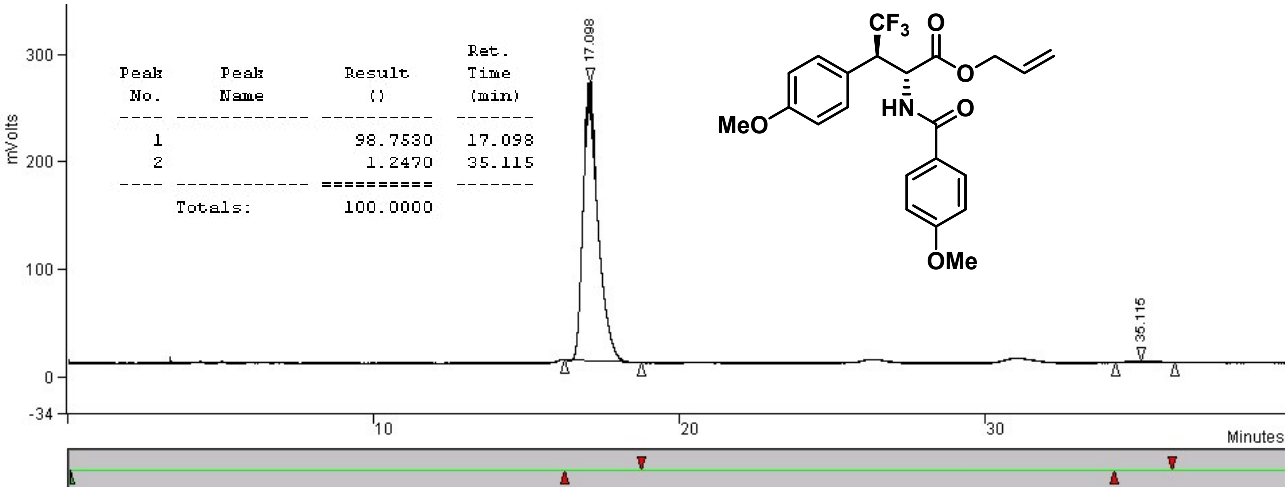


**Allyl(2*R*,3*R*)-4,4,4-trifluoro-2-(4-methoxybenzamido)-3-(4-methoxyphenyl)butanoate** *syn*-**4i**.

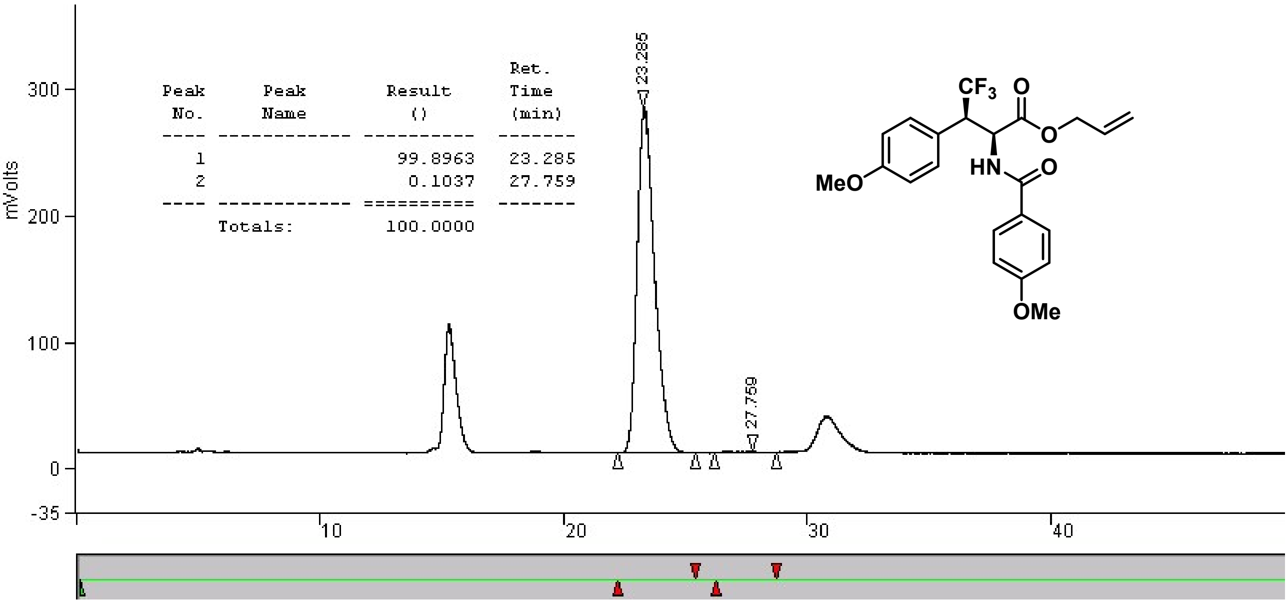

**Allyl 3-(3,5-difluorophenyl)-4,4,4-trifluoro-2-(4-methoxybenzamido)butanoate *rac*-4j.**

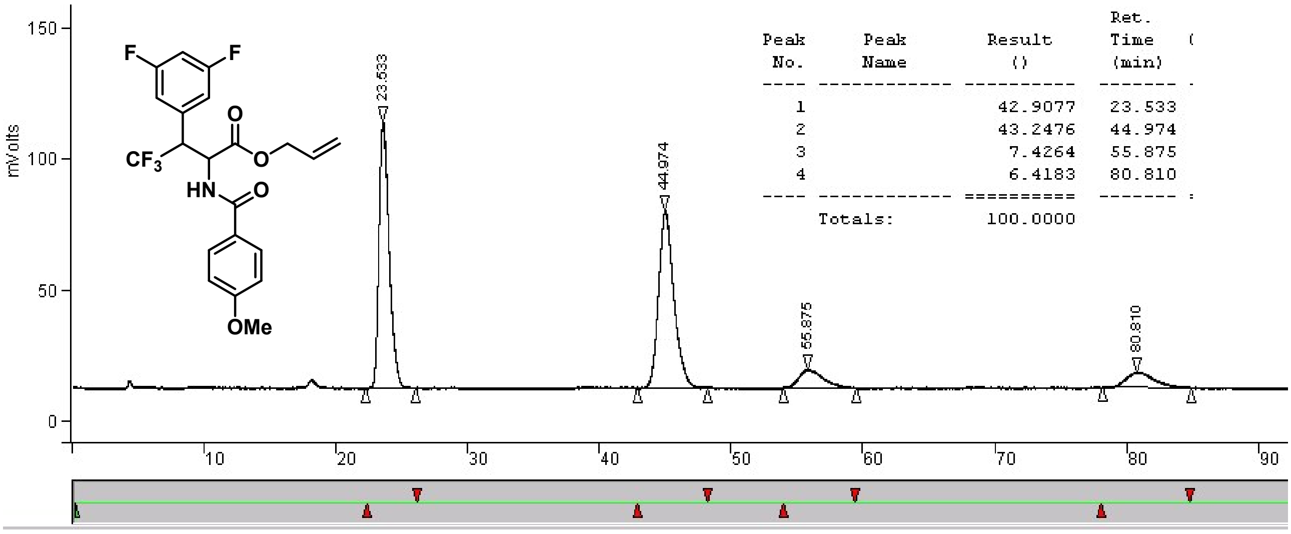


**Allyl (2*R*,3R)-3-(3,5-difluorophenyl)-4,4,4-trifluoro-2-(4-methoxybenzamido)butanoate *anti*-4j.**

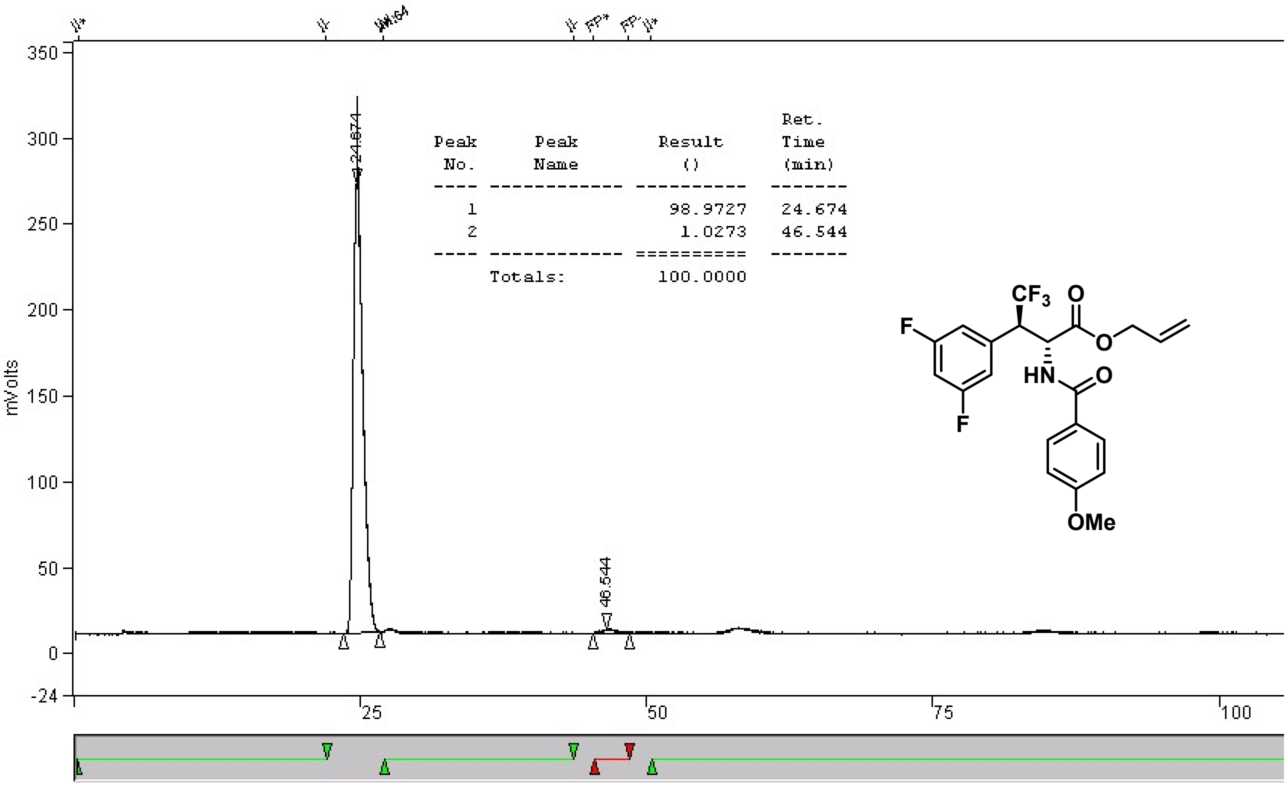

**Allyl (2*R*,3R)-3-(3,5-difluorophenyl)-4,4,4-trifluoro-2-(4-methoxybenzamido)butanoate *syn*-4j.**

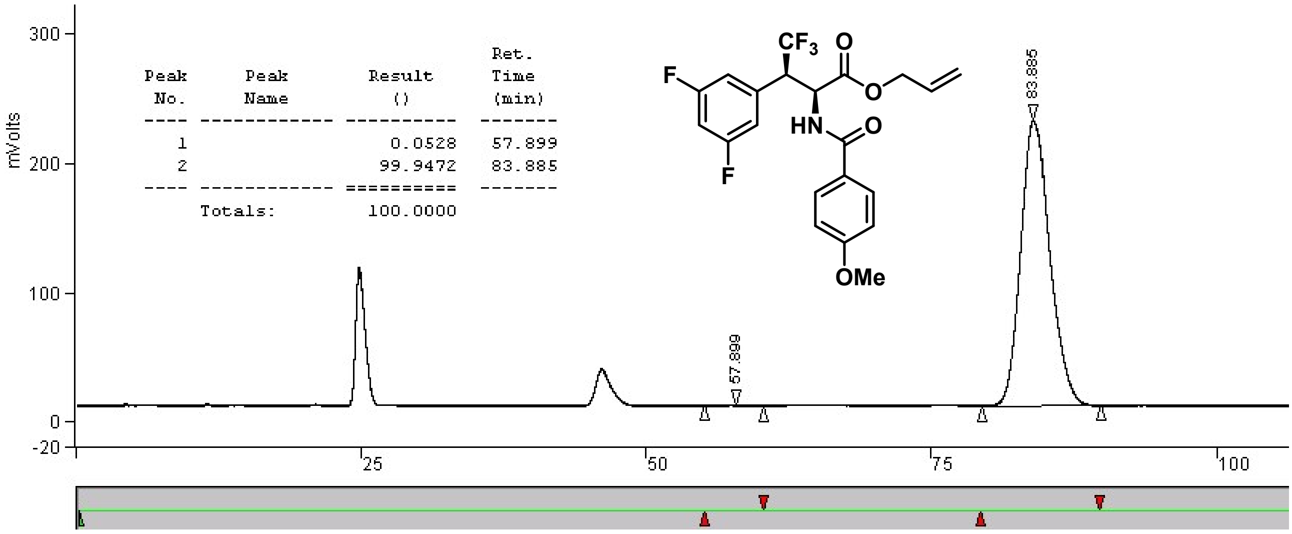


**Allyl -4,4,4-trifluoro-2-(4-methoxybenzamido)-3-(thiophen-2-yl)butanoate *rac*-4k.**

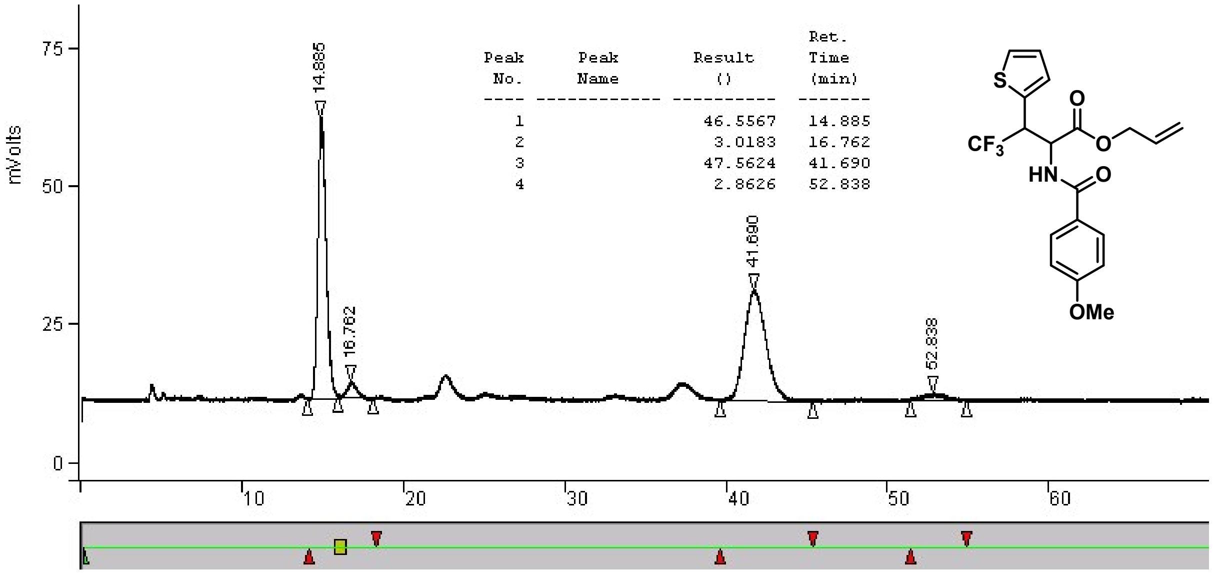

**Allyl (2*R*,3*R*)-4,4,4-trifluoro-2-(4-methoxybenzamido)-3-(thiophen-2-yl)butanoate *anti*-4k.**

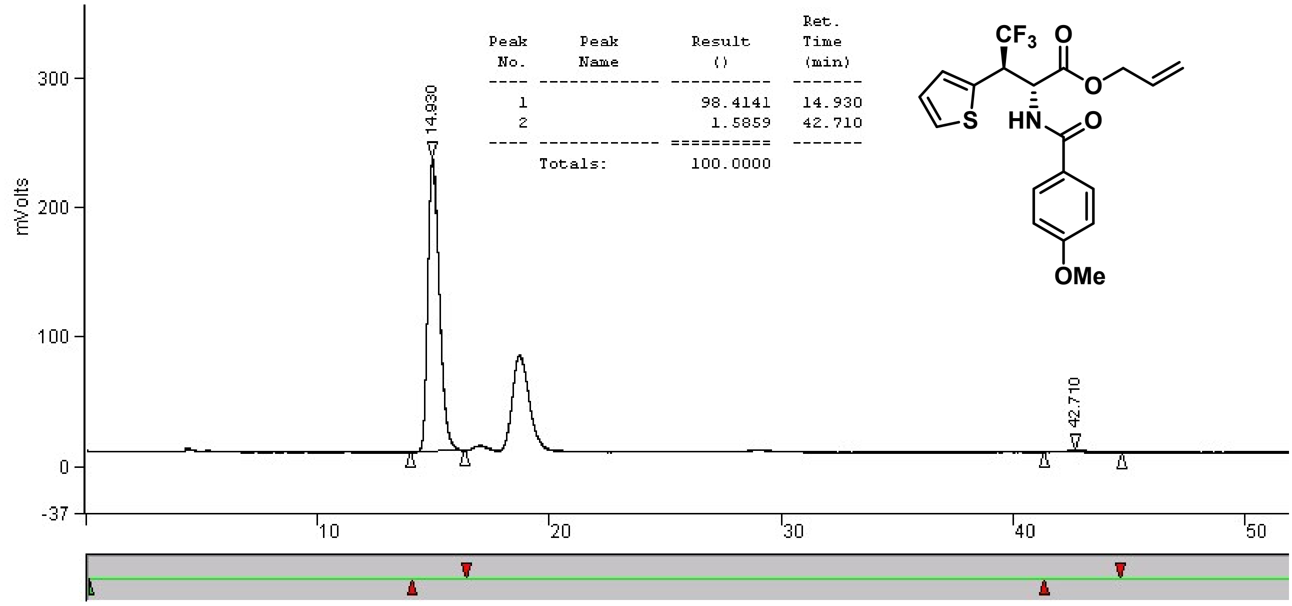


| Peak No. | Peak Name | Result () | Ret. Time (min) |
|---|---|---|---|
| 1 | | 98.4141 | 14.930 |
| 2 | | 1.5859 | 42.710 |
| | Totals: | 100.0000 | |

**Allyl (2*R*,3*R*)-4,4,4-trifluoro-2-(4-methoxybenzamido)-3-(thiophen-2-yl)butanoate *syn*-4k.**

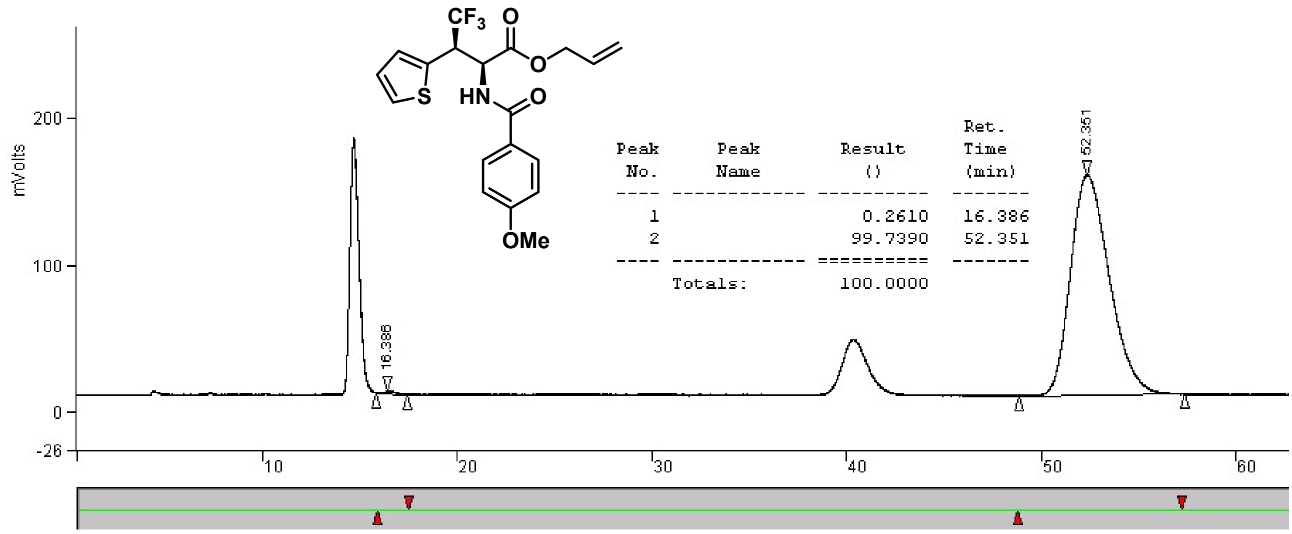


| Peak No. | Peak Name | Result () | Ret. Time (min) |
|---|---|---|---|
| 1 | | 0.2610 | 16.386 |
| 2 | | 99.7390 | 52.351 |
| | Totals: | 100.0000 | |

**Allyl 4,4,4-trifluoro-2-(4-methoxybenzamido)-3-(1-tosyl-1H-indol-3-yl)butanoate *rac*-4l.**

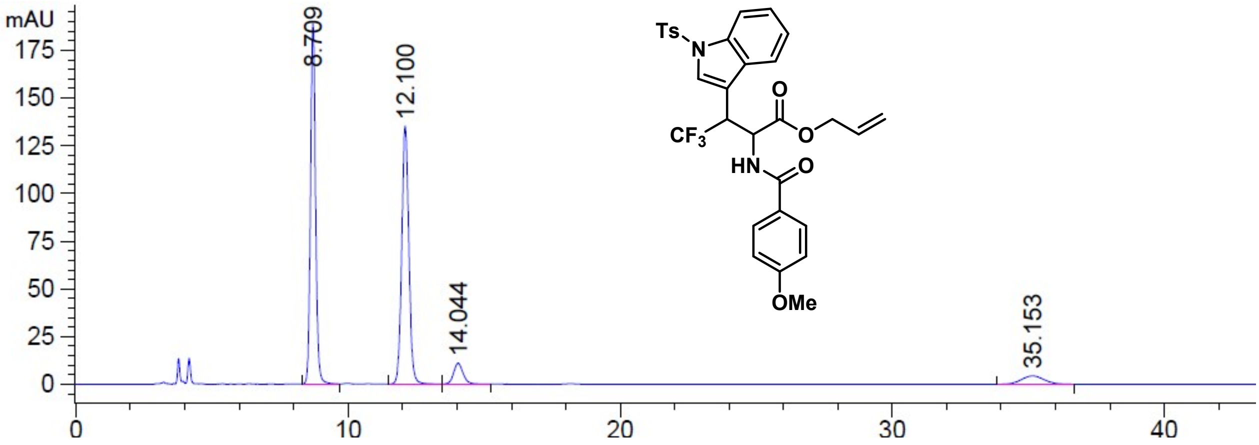


| Peak # | RetTime [min] | Type | Width [min] | Area [mAU*s] | Height [mAU] | Area % |
|---|---|---|---|---|---|---|
| 1 | 8.709 | BB | 0.1993 | 2450.37231 | 188.96262 | 45.0587 |
| 2 | 12.100 | BB | 0.2785 | 2461.34082 | 135.14580 | 45.2604 |
| 3 | 14.044 | BB | 0.3701 | 265.93655 | 10.92214 | 4.8902 |
| 4 | 35.153 | BB | 0.7109 | 260.52960 | 4.39941 | 4.7908 |

**Allyl (2*R*,3*R*)-4,4,4-trifluoro-2-(4-methoxybenzamido)-3-(1-tosyl-1H-indol-3-yl)butanoate *anti*-4l.**

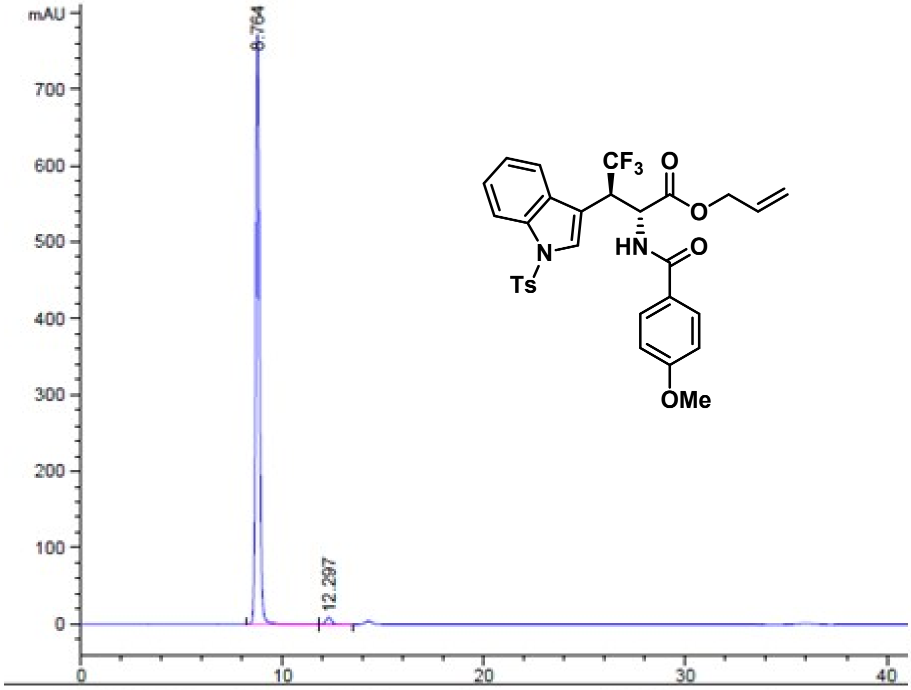


| Peak # | RetTime [min] | Type | Width [min] | Area [mAU*s] | Height [mAU] | Area % |
|---|---|---|---|---|---|---|
| 1 | 8.764 | BV | 0.2006 | 1.00975e4 | 771.98810 | 98.2654 |
| 2 | 12.297 | VB | 0.2917 | 178.24515 | 9.38069 | 1.7346 |
| Totals : | | | | 1.02757e4 | 781.36879 | |

**Allyl (2*S*,3*R*)-4,4,4-trifluoro-2-(4-methoxybenzamido)-3-(1-tosyl-1H-indol-3-yl)butanoate *syn*-4l.**

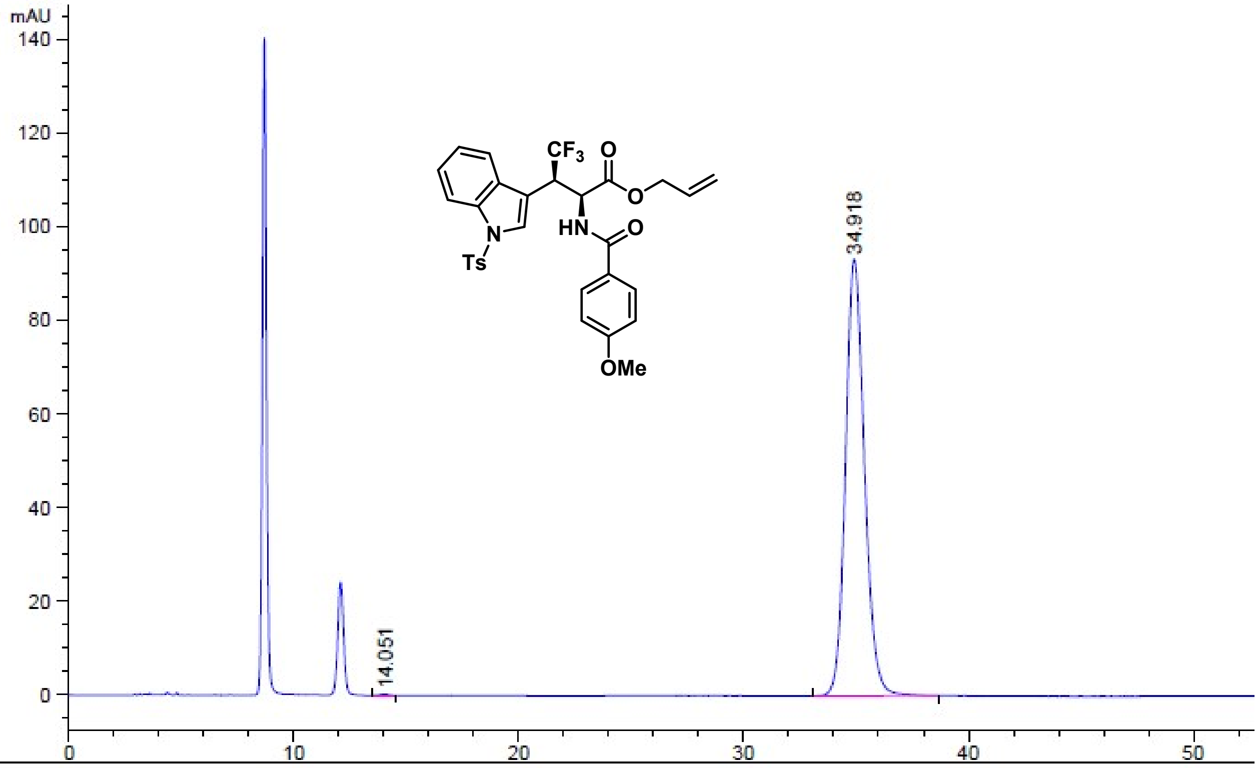


| Peak # | RetTime [min] | Type | Width [min] | Area [mAU*s] | Height [mAU] | Area % |
|---|---|---|---|---|---|---|
| 1 | 14.051 | BB | 0.3227 | 5.76035 | 2.24958e-1 | 0.1051 |
| 2 | 34.918 | BB | 0.9046 | 5472.81055 | 93.26136 | 99.8949 |

Totals : 5478.57090 93.48632

**Allyl 4,4,5,5,6,6,6-heptafluoro-2-(4-methoxybenzamido)-3-phenylhexanoate *rac*-4m.**

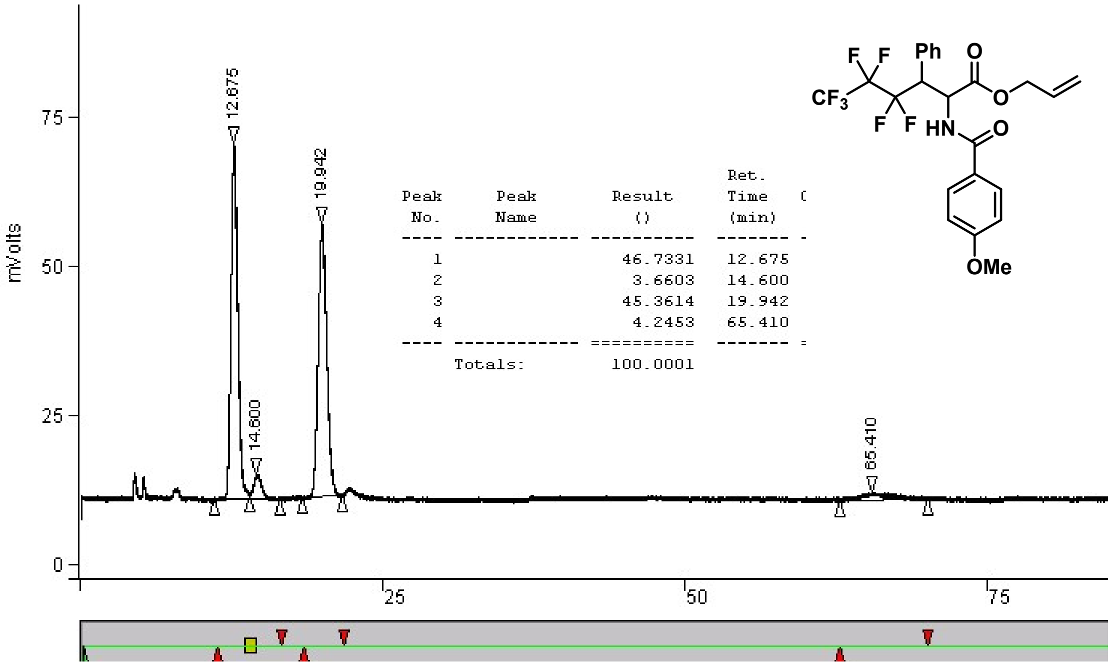


**Allyl (2*R*,3*R*)-4,4,5,5,6,6,6-heptafluoro-2-(4-methoxybenzamido)-3-phenylhexanoate *anti*-4m.**

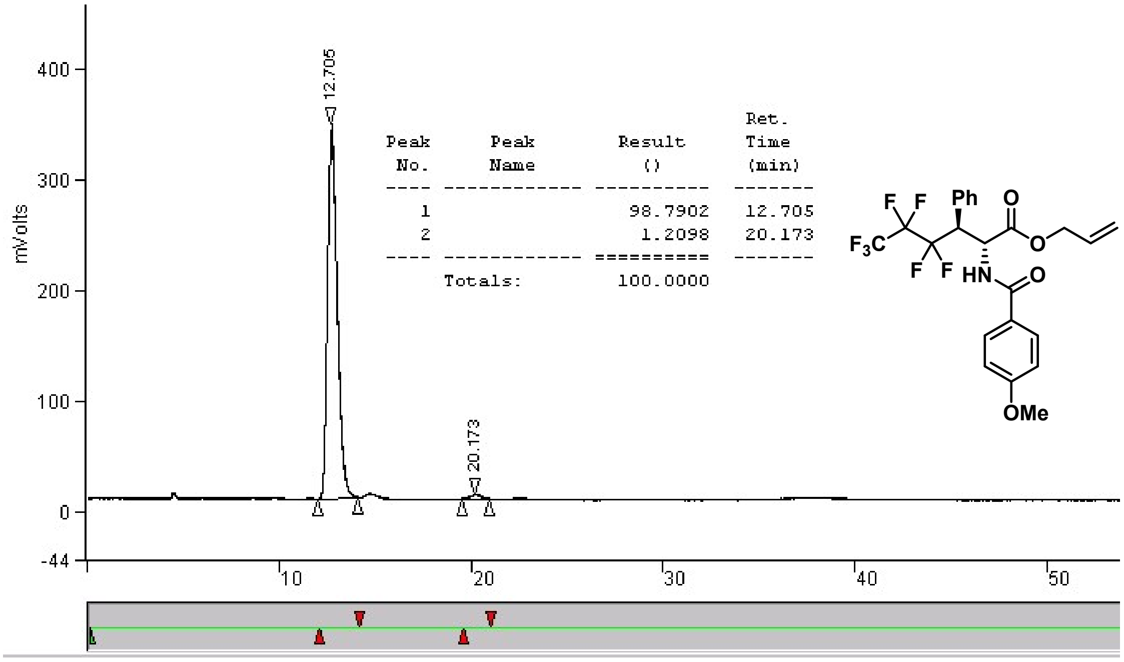

**Allyl (2*S*,3*R*)-4,4,5,5,6,6,6-heptafluoro-2-(4-methoxybenzamido)-3-phenylhexanoate *syn*-4m**.

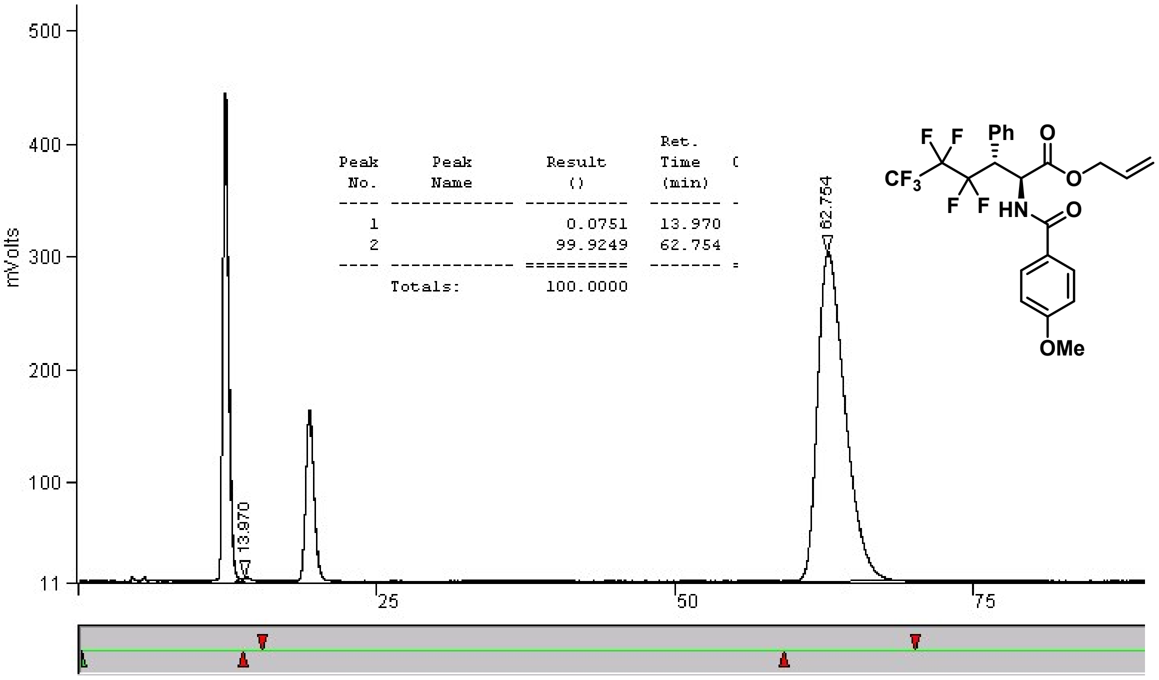


| Peak No. | Peak Name | Result () | Ret. Time (min) |
|---|---|---|---|
| 1 | | 0.0751 | 13.970 |
| 2 | | 99.9249 | 62.754 |
| | Totals: | 100.0000 | |

**Allyl-3-cyclohexyl-4,4,4-trifluoro-2-(4-methoxybenzamido)butanoate *rac*-4n**.

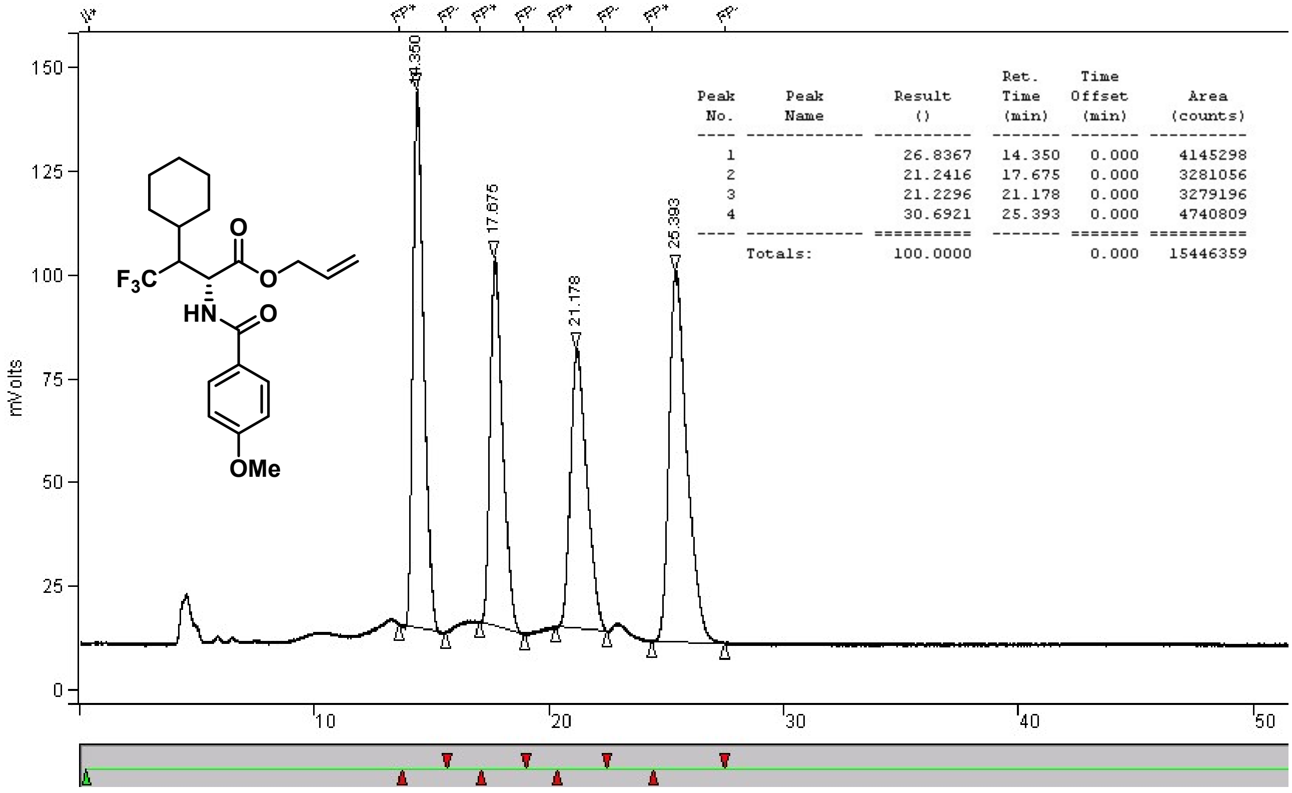


| Peak No. | Peak Name | Result () | Ret. Time (min) | Time Offset (min) | Area (counts) |
|---|---|---|---|---|---|
| 1 | | 26.8367 | 14.350 | 0.000 | 4145298 |
| 2 | | 21.2416 | 17.675 | 0.000 | 3281056 |
| 3 | | 21.2296 | 21.178 | 0.000 | 3279196 |
| 4 | | 30.6921 | 25.393 | 0.000 | 4740809 |
| | Totals: | 100.0000 | | 0.000 | 15446359 |

**Allyl (2*R*,3*R*)-3-cyclohexyl-4,4,4-trifluoro-2-(4-methoxybenzamido)butanoate *anti*-4n.**

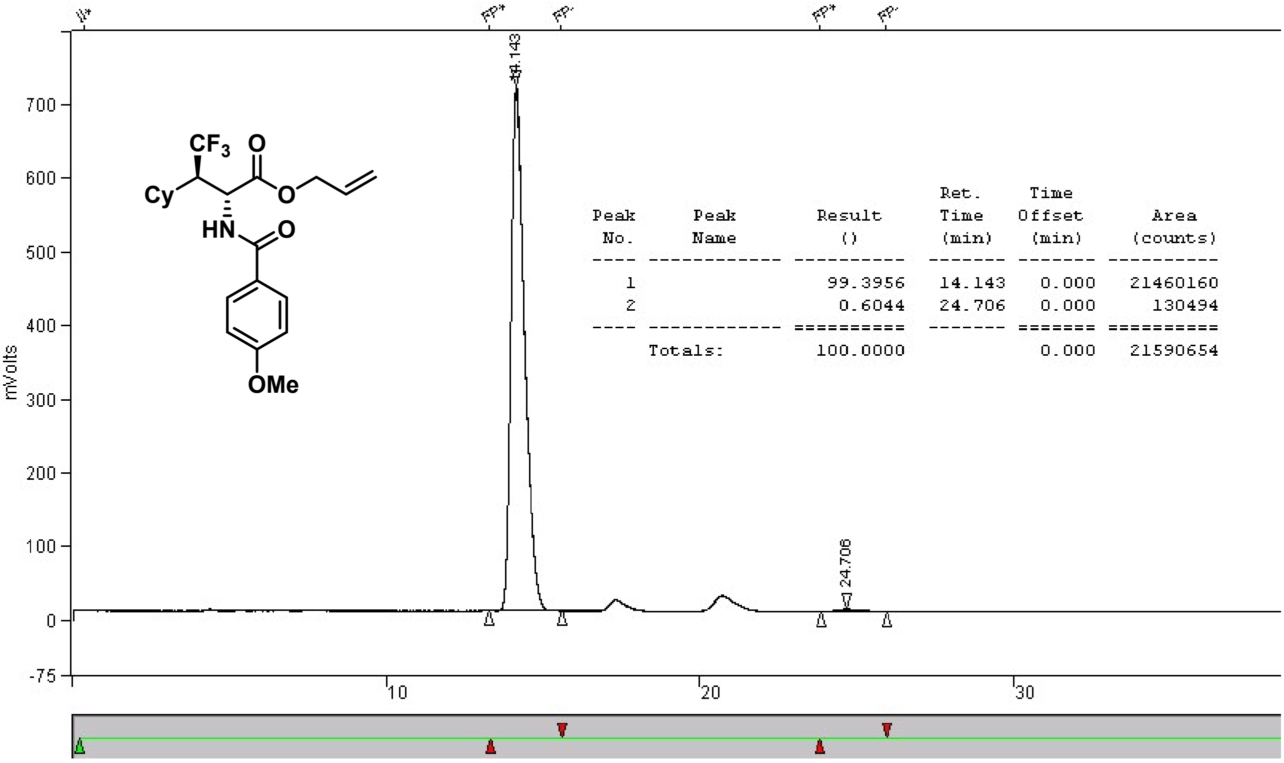


| Peak No. | Peak Name | Result () | Ret. Time (min) | Time Offset (min) | Area (counts) |
|---|---|---|---|---|---|
| 1 | | 99.3956 | 14.143 | 0.000 | 21460160 |
| 2 | | 0.6044 | 24.706 | 0.000 | 130494 |
| | Totals: | 100.0000 | | 0.000 | 21590654 |

**Allyl (2*S*,3*R*)-3-cyclohexyl-4,4,4-trifluoro-2-(4-methoxybenzamido)butanoate *syn*-4n.**

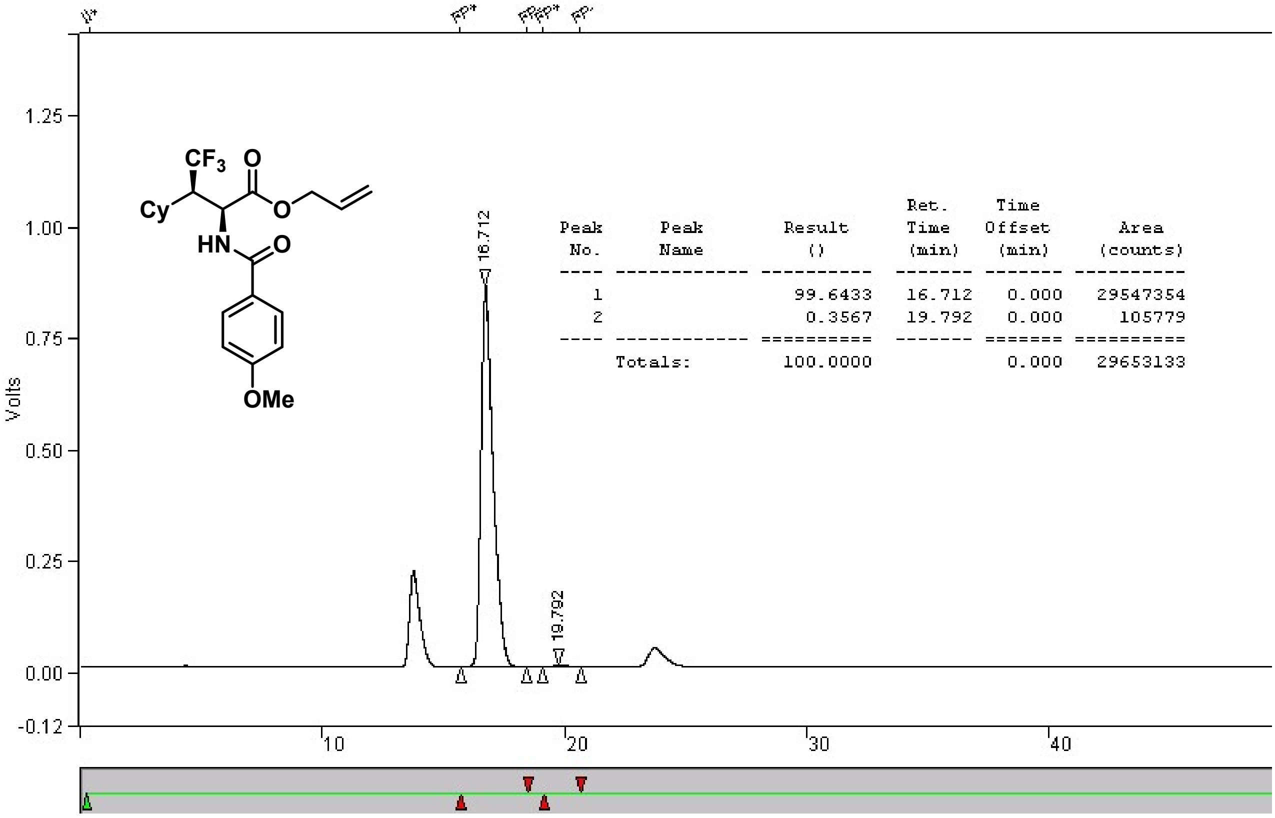


| Peak No. | Peak Name | Result () | Ret. Time (min) | Time Offset (min) | Area (counts) |
|---|---|---|---|---|---|
| 1 | | 99.6433 | 16.712 | 0.000 | 29547354 |
| 2 | | 0.3567 | 19.792 | 0.000 | 105779 |
| | Totals: | 100.0000 | | 0.000 | 29653133 |

**Allyl-2-(4-methoxybenzamido)-3-(trifluoromethyl)pentanoate *rac*-4o.**

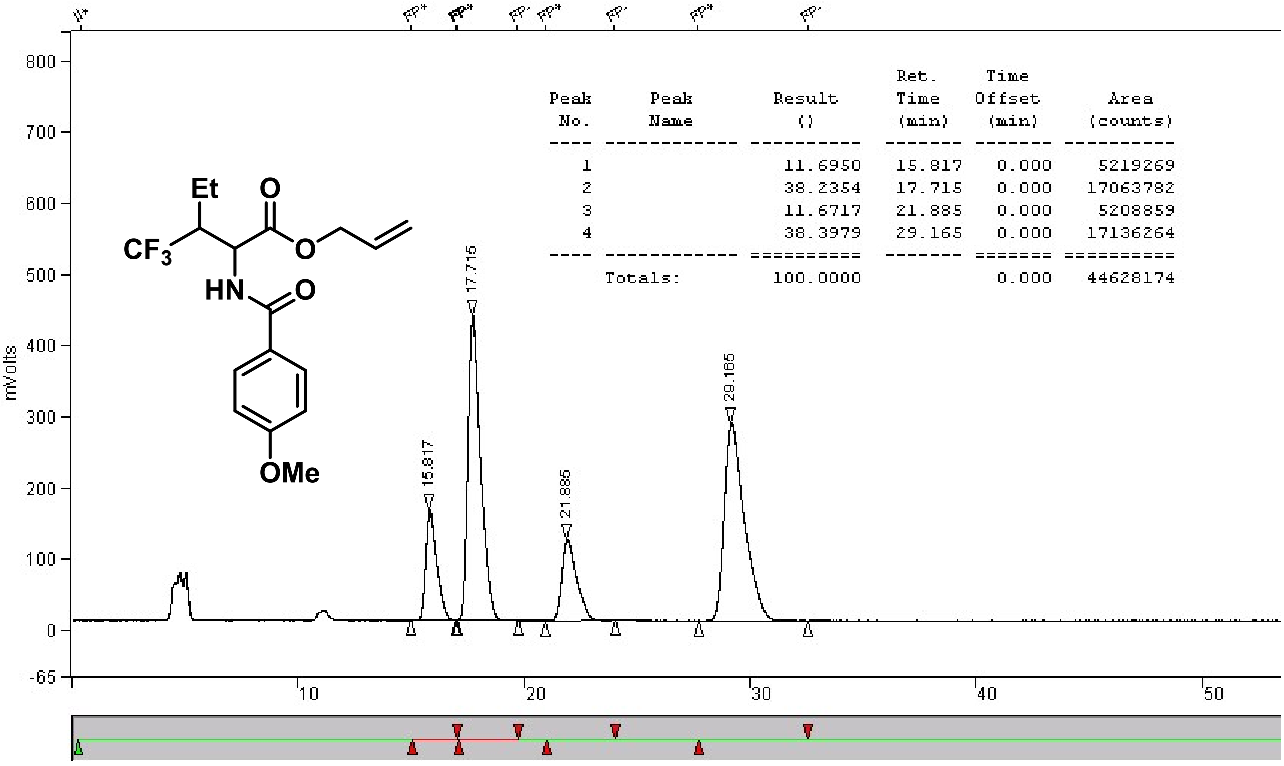


| Peak No. | Peak Name | Result () | Ret. Time (min) | Time Offset (min) | Area (counts) |
|---|---|---|---|---|---|
| 1 | | 11.6950 | 15.817 | 0.000 | 5219269 |
| 2 | | 38.2354 | 17.715 | 0.000 | 17063782 |
| 3 | | 11.6717 | 21.885 | 0.000 | 5208859 |
| 4 | | 38.3979 | 29.165 | 0.000 | 17136264 |
| | Totals: | 100.0000 | | 0.000 | 44628174 |

**Allyl (2*R*,3*R*)-2-(4-methoxybenzamido)-3-(trifluoromethyl)pentanoate *anti*-4o.**

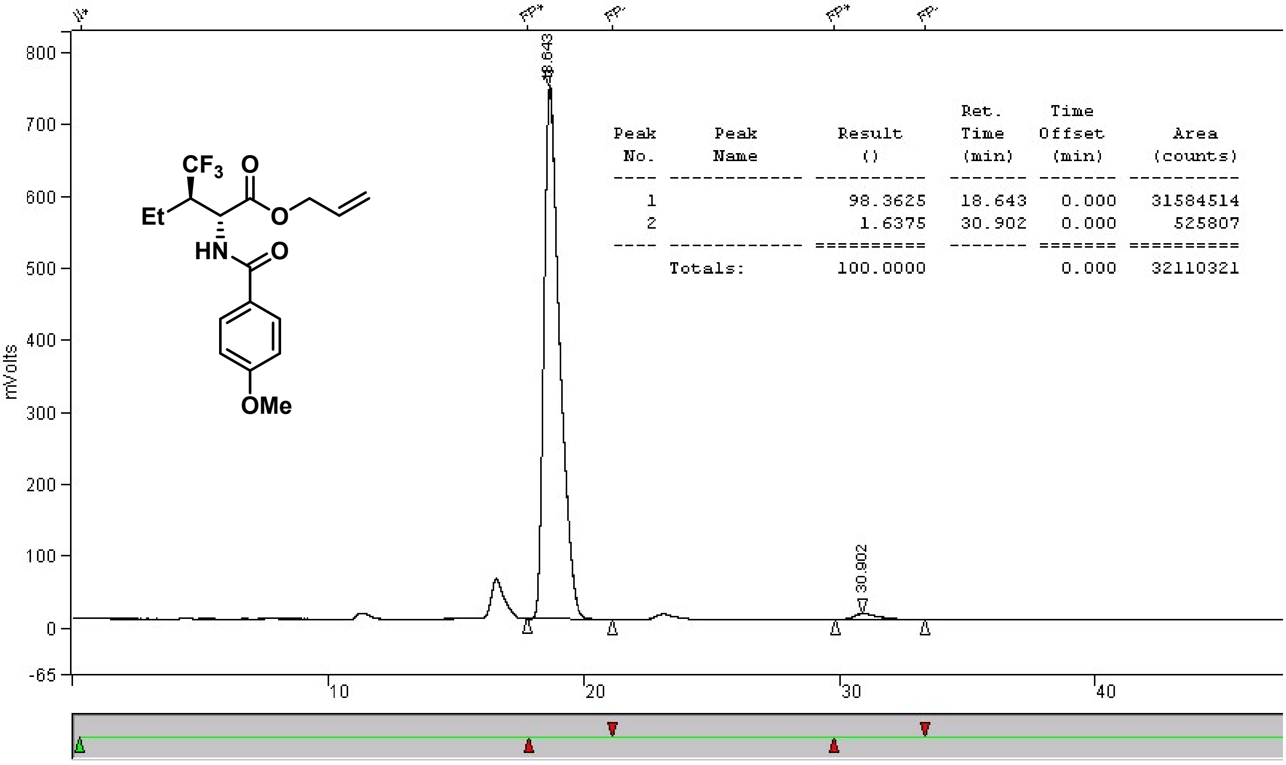


| Peak No. | Peak Name | Result () | Ret. Time (min) | Time Offset (min) | Area (counts) |
|---|---|---|---|---|---|
| 1 | | 98.3625 | 18.643 | 0.000 | 31584514 |
| 2 | | 1.6375 | 30.902 | 0.000 | 525807 |
| | Totals: | 100.0000 | | 0.000 | 32110321 |

**Allyl (2*S*,3*R*)-2-(4-methoxybenzamido)-3-(trifluoromethyl)pentanoate *syn*-4o.**

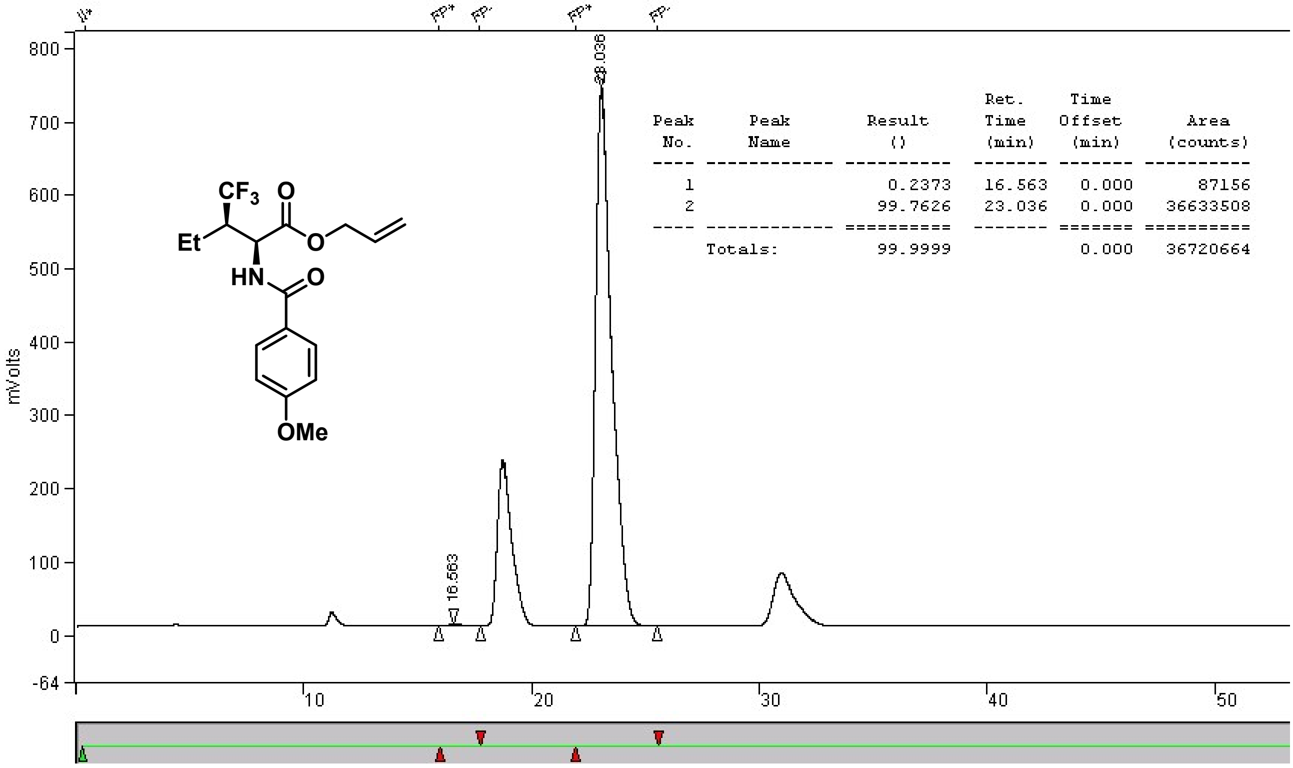


| Peak No. | Peak Name | Result () | Ret. Time (min) | Time Offset (min) | Area (counts) |
|---|---|---|---|---|---|
| 1 | | 0.2373 | 16.563 | 0.000 | 87156 |
| 2 | | 99.7626 | 23.036 | 0.000 | 36633508 |
| | Totals: | 99.9999 | | 0.000 | 36720664 |

**Allyl-4,4,4-trifluoro-2-(4-methoxybenzamido)-3-methylbutanoate *rac*-4p.**

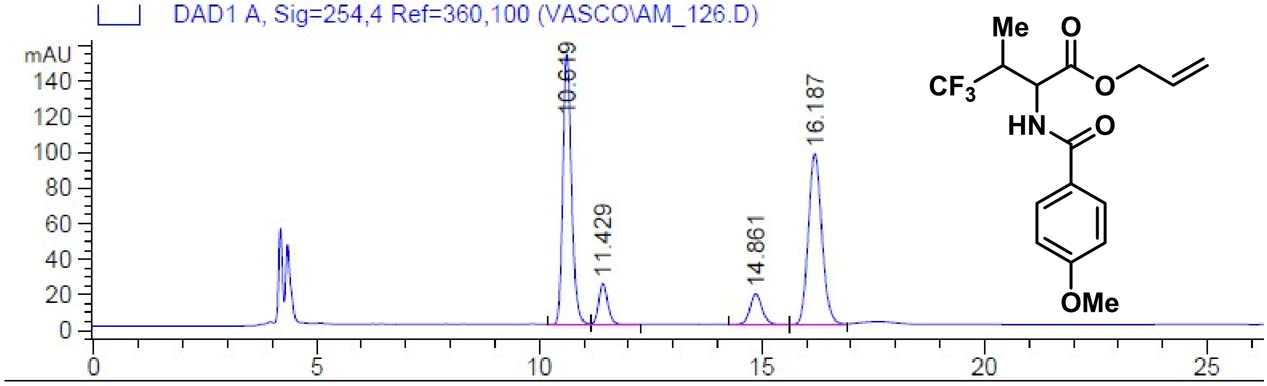


| Peak # | RetTime [min] | Type | Width [min] | Area [mAU*s] | Height [mAU] | Area % |
|---|---|---|---|---|---|---|
| 1 | 10.619 | BV | 0.2052 | 2043.43494 | 151.62614 | 43.1297 |
| 2 | 11.429 | VB | 0.2175 | 327.56653 | 23.09250 | 6.9138 |
| 3 | 14.861 | BV | 0.2935 | 329.91904 | 17.21866 | 6.9634 |
| 4 | 16.187 | VV | 0.3279 | 2036.96057 | 95.85818 | 42.9931 |

**Allyl (2*R*,3*R*)-4,4,4-trifluoro-2-(4-methoxybenzamido)-3-methylbutanoate *anti*-4p.**

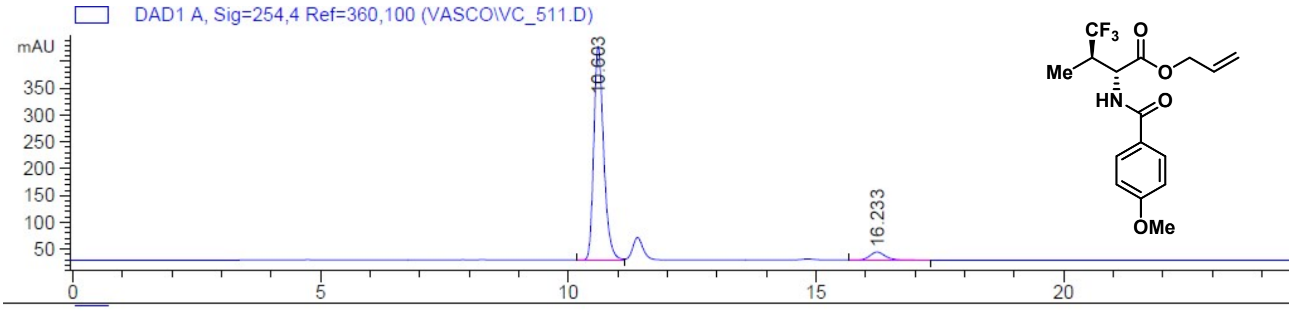


| Peak # | RetTime [min] | Type | Width [min] | Area [mAU*s] | Height [mAU] | Area % |
|---|---|---|---|---|---|---|
| 1 | 10.603 | BV | 0.2116 | 5527.60693 | 398.97031 | 94.5727 |
| 2 | 16.233 | BB | 0.3304 | 317.21564 | 14.77696 | 5.4273 |

**Allyl (2*S*,3*R*)-4,4,4-trifluoro-2-(4-methoxybenzamido)-3-methylbutanoate *syn*-4p.**

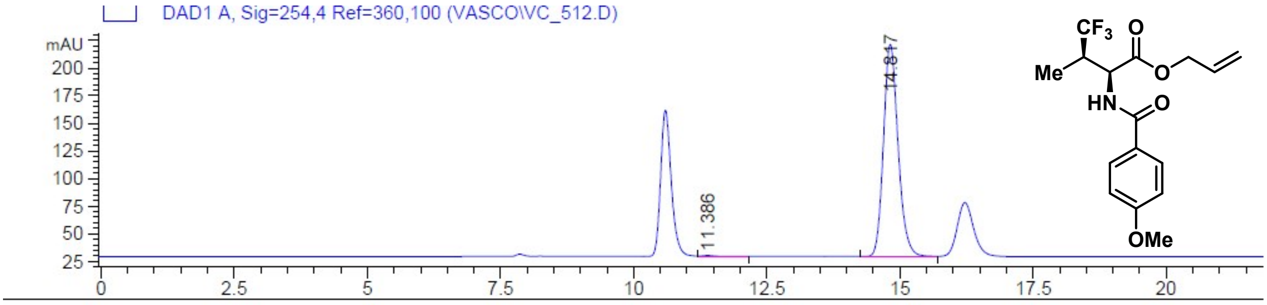


| Peak # | RetTime [min] | Type | Width [min] | Area [mAU*s] | Height [mAU] | Area % |
|---|---|---|---|---|---|---|
| 1 | 11.386 | VB | 0.2484 | 13.73116 | 7.99236e-1 | 0.3736 |
| 2 | 14.817 | BV | 0.2923 | 3661.44995 | 192.17696 | 99.6264 |

**Methyl 4,4,4-trifluoro-2-(4-methoxybenzamido)-3-phenylbutanoate *rac*-4q.**

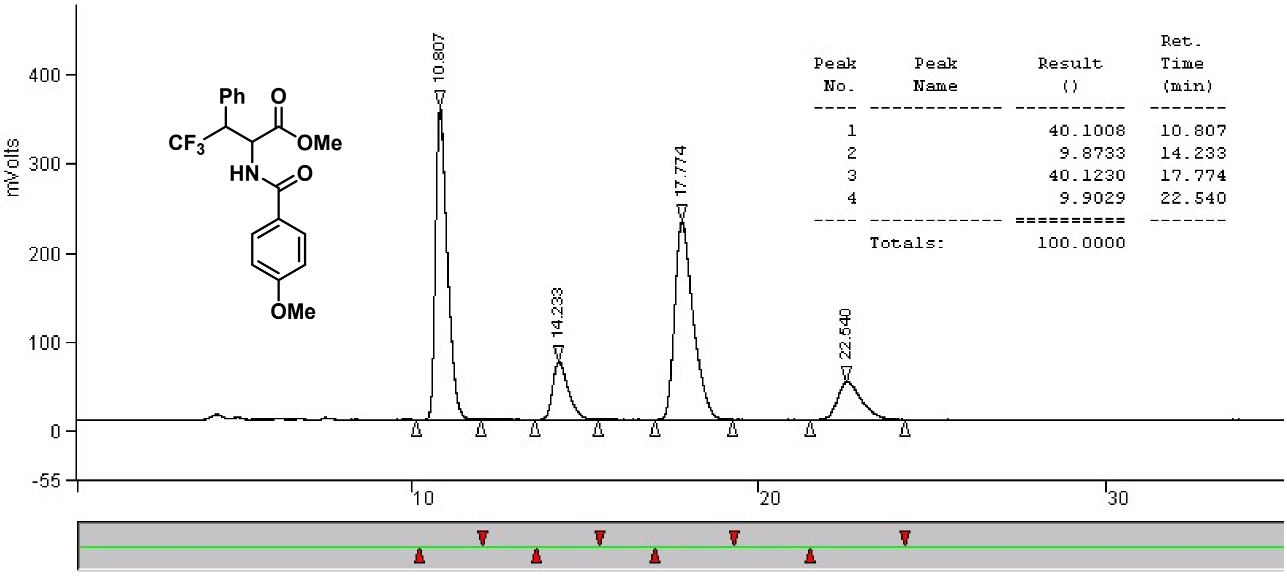


**Methyl (2*R*,3*R*)-4,4,4-trifluoro-2-(4-methoxybenzamido)-3-phenylbutanoate *anti*-4q.**

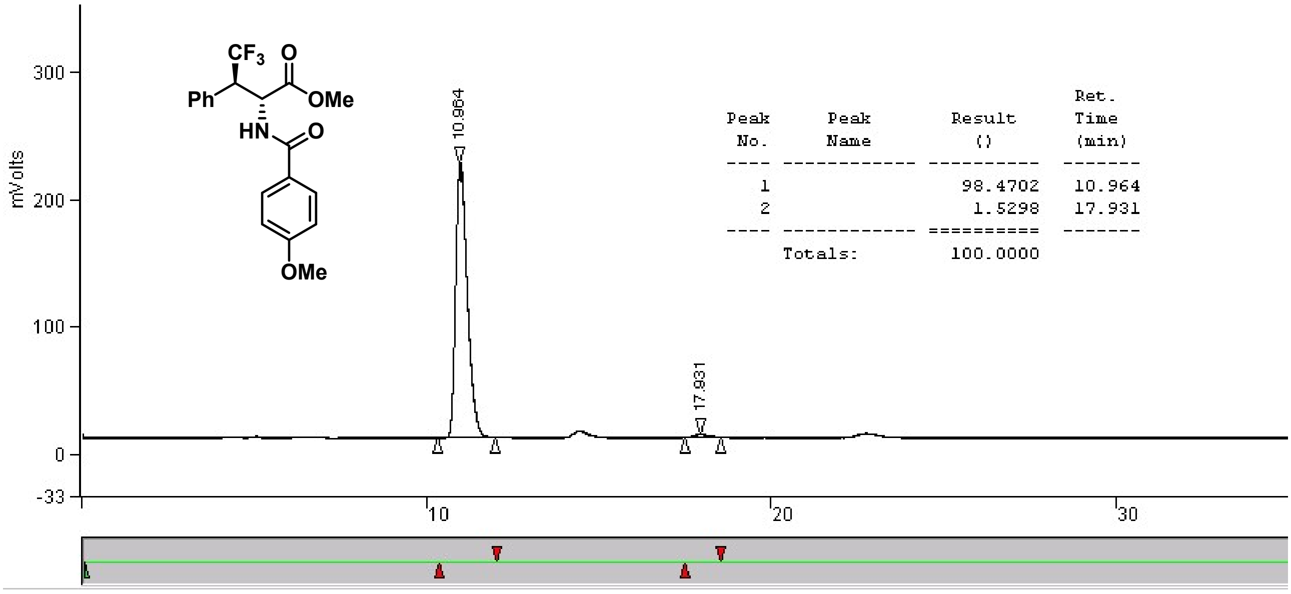

**Methyl (2*S*,3*R*)-4,4,4-trifluoro-2-(4-methoxybenzamido)-3-phenylbutanoate *syn*-4q.**

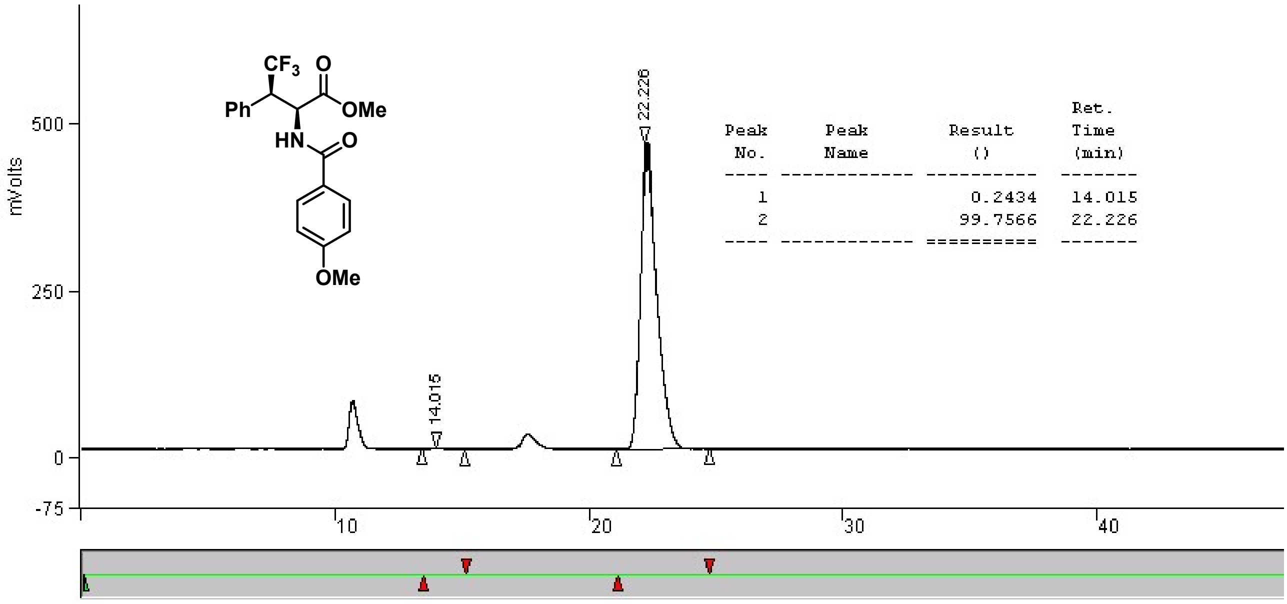


| Peak No. | Peak Name | Result () | Ret. Time (min) |
|---|---|---|---|
| 1 | | 0.2434 | 14.015 |
| 2 | | 99.7566 | 22.226 |

**Benzyl 4,4,4-trifluoro-2-(4-methoxybenzamido)-3-phenylbutanoate *rac*-4r.**

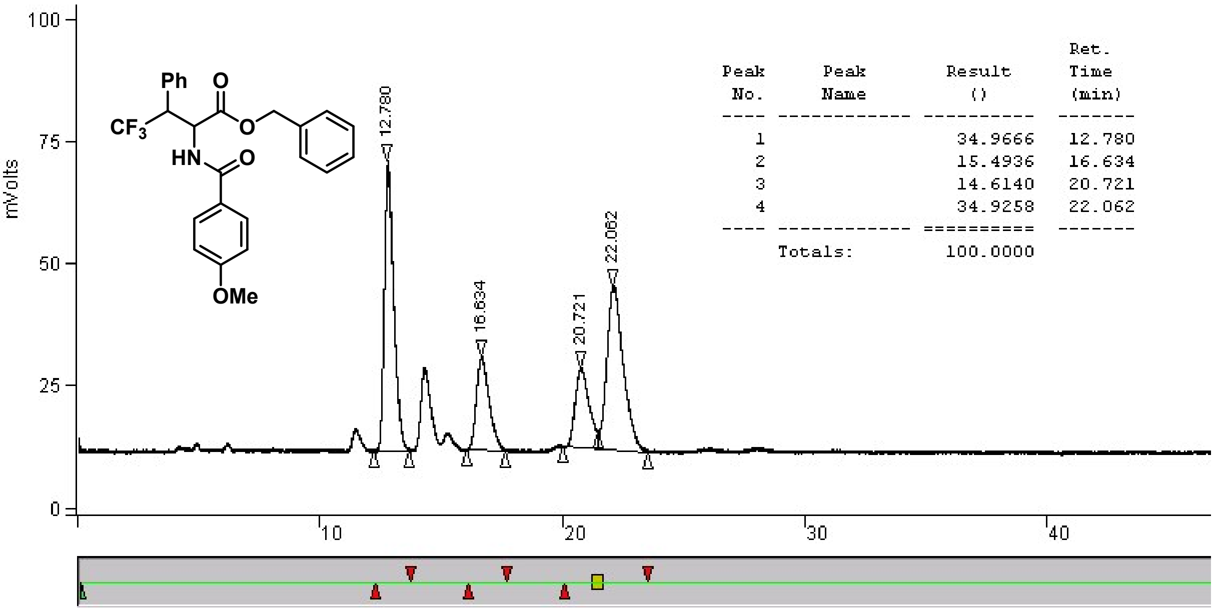


| Peak No. | Peak Name | Result () | Ret. Time (min) |
|---|---|---|---|
| 1 | | 34.9666 | 12.780 |
| 2 | | 15.4936 | 16.634 |
| 3 | | 14.6140 | 20.721 |
| 4 | | 34.9258 | 22.062 |
| | Totals: | 100.0000 | |

**Benzyl (2*R*,3*R*)-4,4,4-trifluoro-2-(4-methoxybenzamido)-3-phenylbutanoate *anti*-4r.**

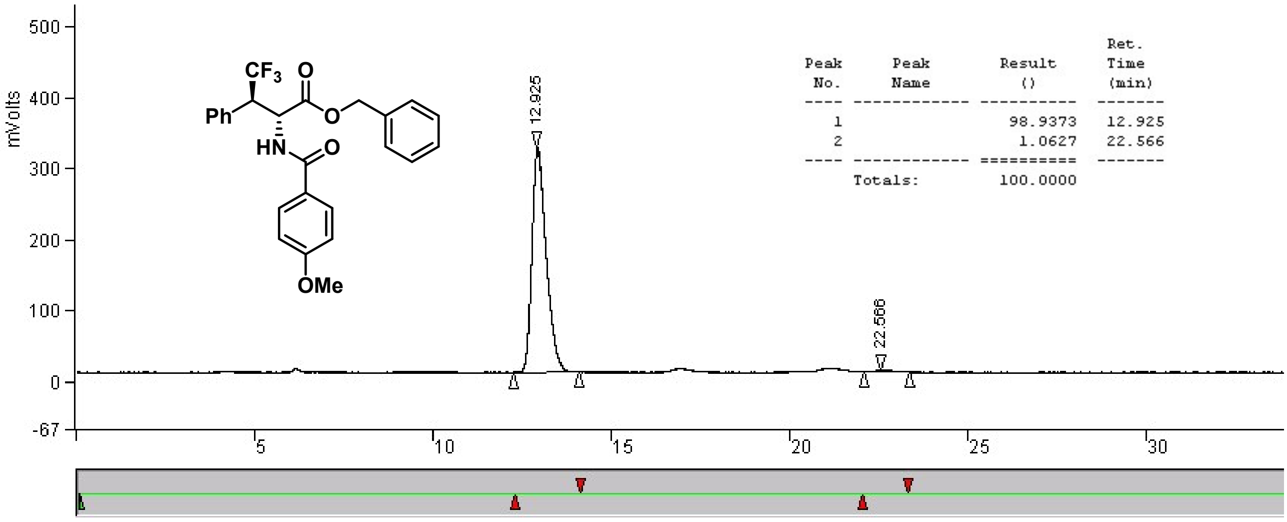


**Benzyl (2*S*,3*R*)-4,4,4-trifluoro-2-(4-methoxybenzamido)-3-phenylbutanoate *syn*-4r.**

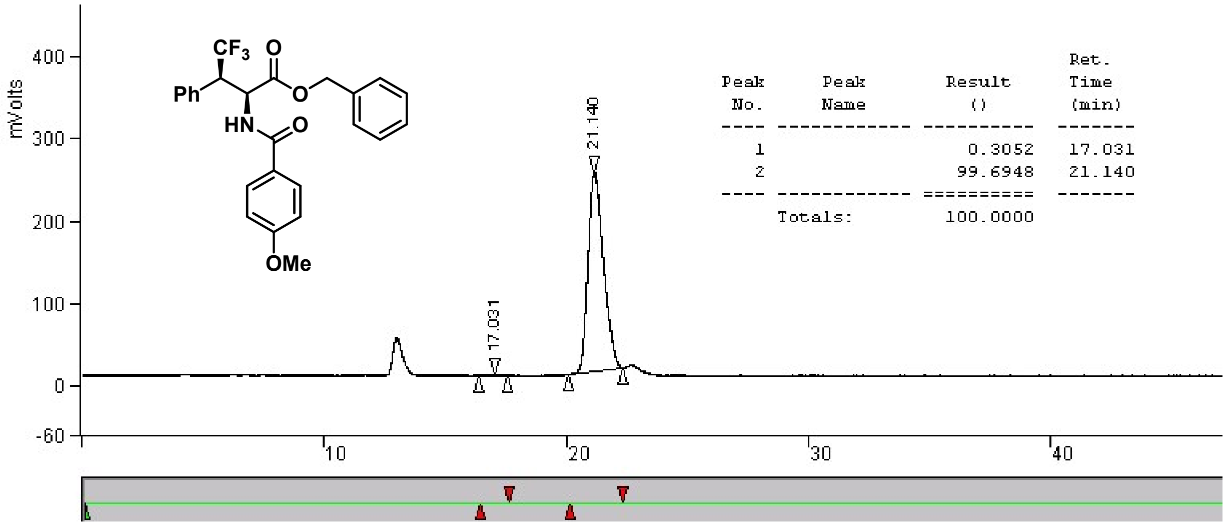

**Isobutyl 4,4,4-trifluoro-2-(4-methoxybenzamido)-3-phenylbutanoate *rac*-4s.**

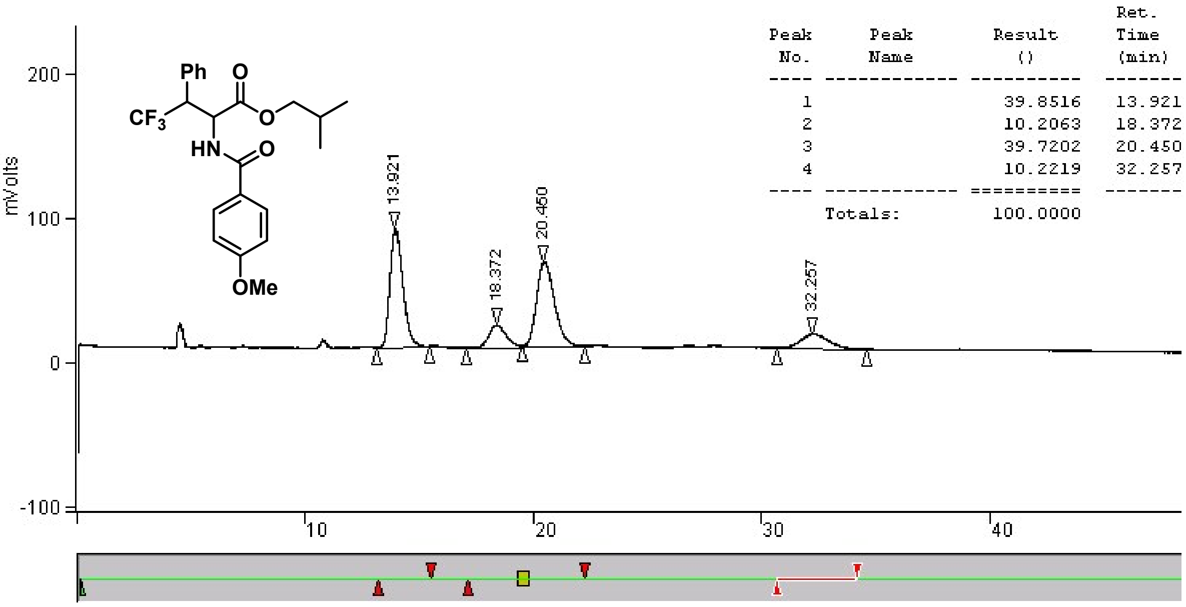


| Peak No. | Peak Name | Result () | Ret. Time (min) |
|---|---|---|---|
| 1 | | 39.8516 | 13.921 |
| 2 | | 10.2063 | 18.372 |
| 3 | | 39.7202 | 20.450 |
| 4 | | 10.2219 | 32.257 |
| | Totals: | 100.0000 | |

**Isobutyl (2*R*,3*R*)-4,4,4-trifluoro-2-(4-methoxybenzamido)-3-phenylbutanoate *anti*-4s.**

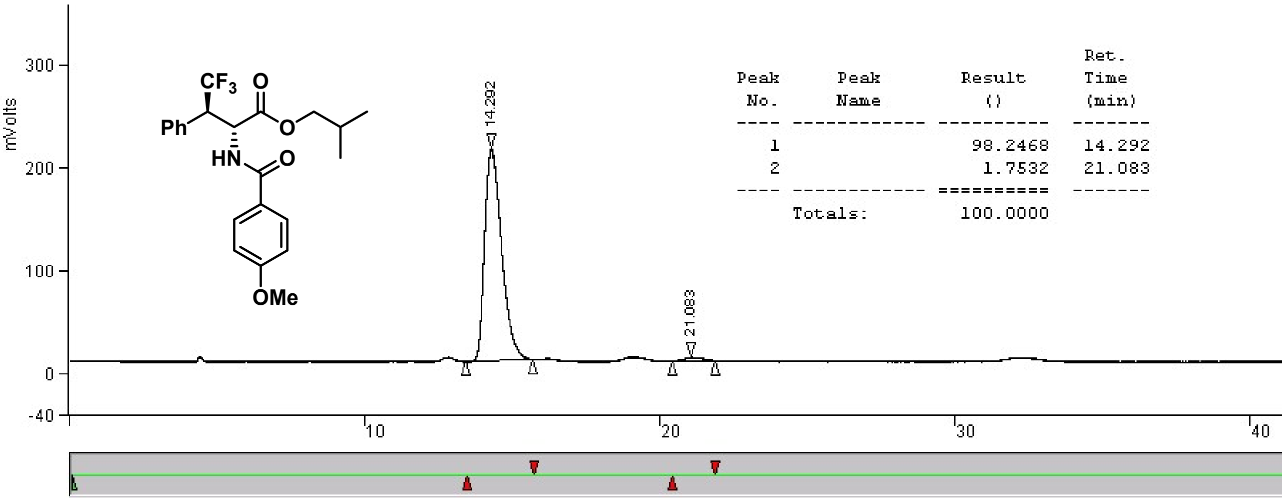


| Peak No. | Peak Name | Result () | Ret. Time (min) |
|---|---|---|---|
| 1 | | 98.2468 | 14.292 |
| 2 | | 1.7532 | 21.083 |
| | Totals: | 100.0000 | |

**Isobutyl (2*S*,3*R*)-4,4,4-trifluoro-2-(4-methoxybenzamido)-3-phenylbutanoate *syn*-4s.**

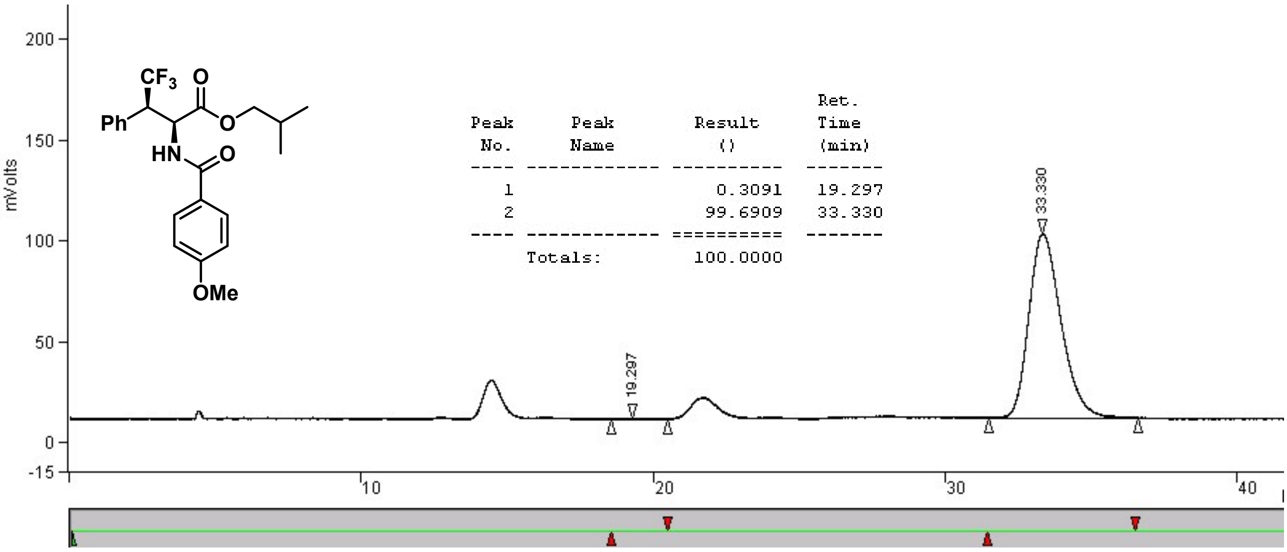


***N*-3-(3,5-difluorophenyl)-4,4,4-trifluoro-1-hydroxybutan-2-yl)-4-methoxybenzamide *rac*-5.**

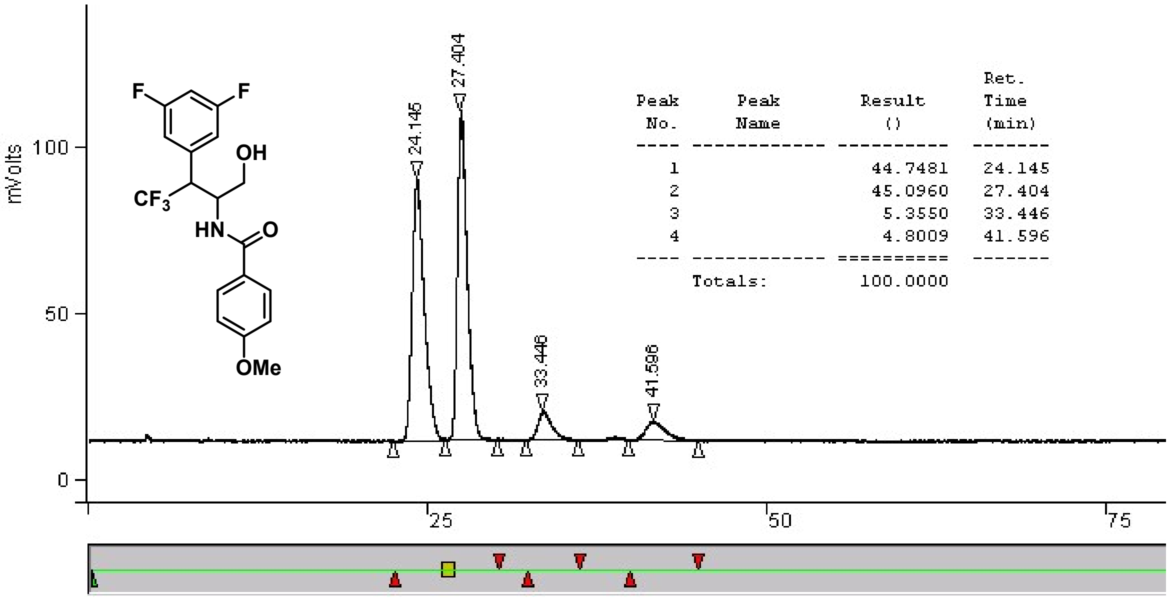

***N*-((2*R*,3*R*)-3-(3,5-difluorophenyl)-4,4,4-trifluoro-1-hydroxybutan-2-yl)-4-methoxybenzamide** *anti*-**5**.

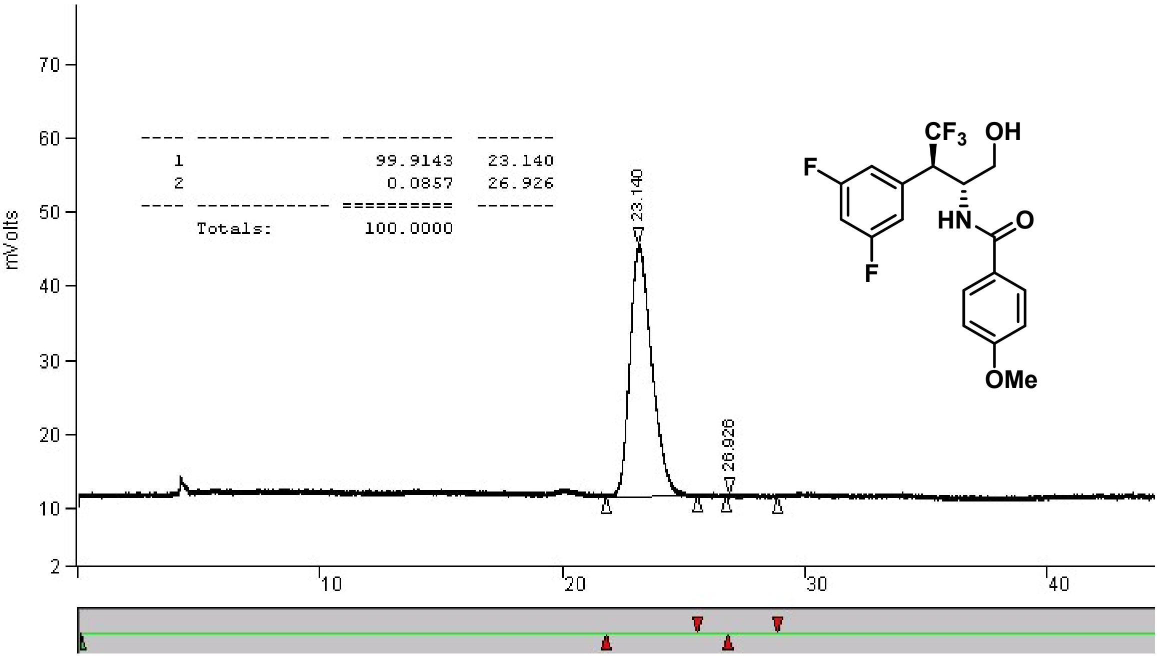


***N*-((2*S*,3*R*)-3-(3,5-difluorophenyl)-4,4,4-trifluoro-1-hydroxybutan-2-yl)-4-methoxybenzamide** *syn*-**5**.

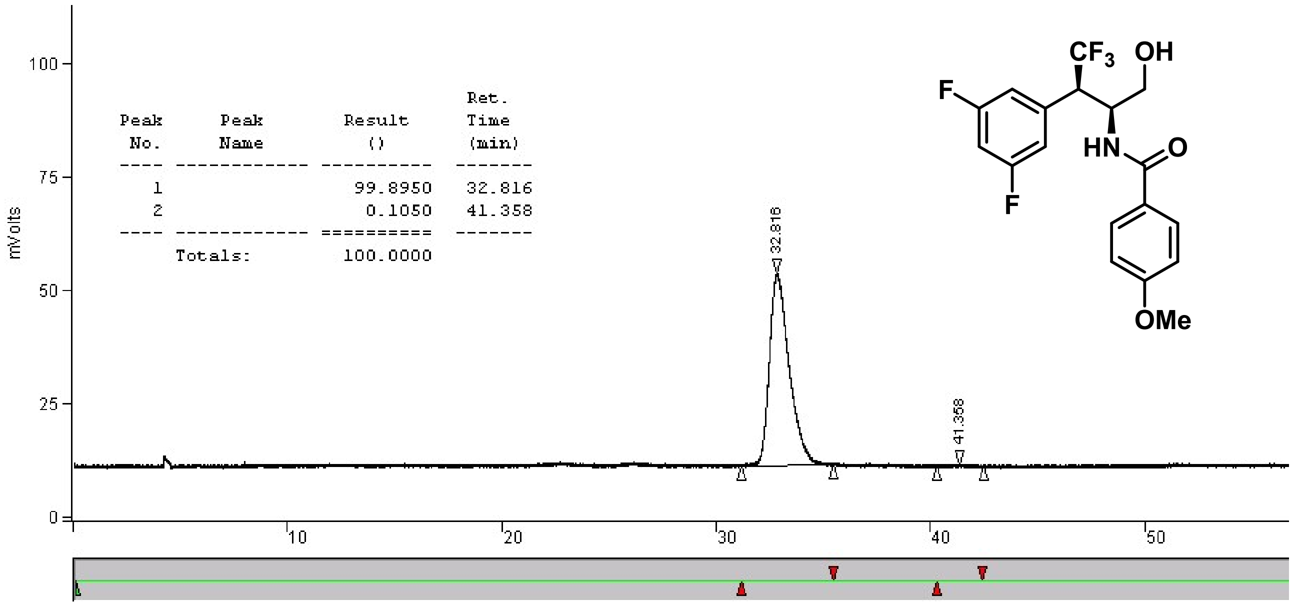

Supplement: SC-012-D1SC01442K-s001 [file SC-012-D1SC01442K-s001.pdf]
